# Evaluating Cubic Equations of State with Various α Functions for Viscosity Predictions of 124 Industrial Important Fluids Based on Residual Entropy Scaling

**DOI:** 10.1021/acsomega.5c01157

**Published:** 2025-06-27

**Authors:** Xiong Xiao, Xiaoxian Yang

**Affiliations:** † Fluid Science & Resources Division, Department of Chemical Engineering, The University of Western Australia, Crawley, WA 6009, Australia; ‡ 38869 Chemnitz University of Technology, Applied Thermodynamics, 09107 Chemnitz, Germany

## Abstract

Accurate prediction of viscosity remains a challenge in industry due to the lack of reliable simple universal models. This work investigates the accuracy of four cubic equations of state (EOS) with different α functions for pure fluid viscosity predictions based on the residual entropy scaling (RES). The cubic EOS are Peng–Robinson (PR), Soave–Redlich–Kwong (SRK), Patel-Teja-Valderrama (PTV), and Yang-Frotscher-Richter (YFR), and the α functions are those proposed by Twu et al., Coquelet et al., Mahmoodi and Sedigh, and Heyen et al. The investigation utilizes the approximately 54,000 experimental viscosity data of 124 pure fluids at pressures below 60 MPa, mainly obtained from the NIST TDE (ThermoData Engine) database. Compared to a previous study ( ACS Omega 2025, 10, 6124)39989818 10.1021/acsomega.4c10815PMC11840589, which focused on the Soave α function, the adoption of modified α functions led to improved viscosity predictions for 39 out of the 124 studied substances. Notable enhancements are observed for R1234ze­(E) and SO_2_ with PTV-Heyen and for CO_2_ with PR-Twu. The absolute average deviation (AAD) between experimental values and model predictions is 3.1% (PR, SRK, and YFR), 3.0% (PTV), 3.4% (PR-Twu and SRK-Coquelet), 3.2% (PTV-Heyen and SRK-MS), 3.5% (PR-Coquelet), and 3.3% (PR-MS). As a reference, the AAD of the various reference models implemented in REFPROP 10.0 is 2.7%. This work demonstrates the potential of integrating optimized α functions to improve the predictive capabilities for viscosity within the cubic EOS + RES framework for certain specific pure fluids. Additionally, a recommended combination of cubic EOS and α function is provided for each fluid studied.

## Graphical abstract

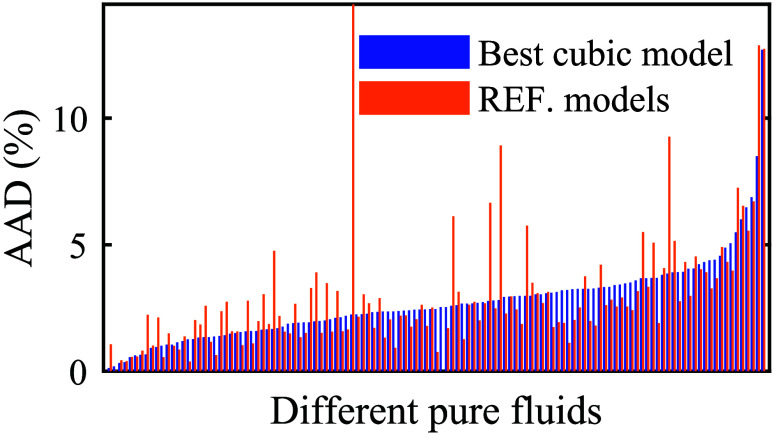

## 1 Introduction

Viscosity is a crucial property for Reynolds number calculations, pressure drop estimations in compressors, and the sizing of pumps and pipelines.^1,2^ Despite its importance as a transport property, accurately predicting viscosity remains challenging for both pure fluids and mixtures under diverse conditions. Existing empirical models may fail to achieve consistent accuracy across different fluids and mixtures.^3–7^ As an example, for a refrigerant mixture of CO_2_ + *R*32, calculated viscosities from the default models implemented in the NIST REFPROP database version 10.0 ^8^ can deviate by up to 8% from experimental values.^9^ These discrepancies result in high uncertainties during equipment design, necessitating overdesign to account for these inaccuracies, a costly requirement for large-scale industrial processes. To address these challenges, the residual entropy scaling (RES) emerged as a promising approach to predict transport properties such as viscosity, thermal conductivity, and self-diffusion coefficient.^10–15^ RES builds on the relationship between transport properties and residual entropy, a thermodynamic property that can be calculated with an equation of state (EOS). Linking transport properties to thermodynamic properties, the EOS + RES framework provides a unified and systematic method to predict transport properties across a wide range of fluids, requiring only a small number of parameters.

Based on the work of Bell et al.^11,16,17^ and the highly accurate multiparameter EOS in the REFPROP 10.0, Yang and colleagues^18–24^ developed a RES approach to model viscosity and thermal conductivity for a wide range of fluids. Particularly, the work of Martinek et al.^21^ demonstrated that the EOS + RES framework could achieve an accuracy comparable to the various state-of-the-art viscosity models implemented in REFPROP 10.0 while utilizing simpler formulations with fewer parameters. However, this approach is limited to the approximate 150 pure fluids available in REFPROP 10.0. In 2025, Yang^25^ developed a RES approach combined with four cubic EOS [Peng–Robinson (PR),^26,27^ Soave–Redlich–Kwong (SRK),^28,29^ Patel-Teja-Valderrama (PTV),^30,31^ and Yang-Frotscher-Richter (YFR)^32^ EOS] to calculate the viscosity and thermal conductivity of approximate 150 fluids. Another application of the cubic EOS + RES framework was the prediction of the viscosity of choline-chloride-based deep eutectic solvents (DESs) at temperatures up to 100 °C and pressures up to 1000 bar by Macías-Salinas and Pereda-Cruz.^33^ These researches have laid the foundation for the applications of EOS + RES framework to more fluids. For viscosity, Yang’s approach is limited at pressures below 60 MPa, and achieved an AAD from experimental data to model predictions of 3.1%. This result is slightly higher but comparable to 2.7%^21^ obtained by the state-of-the-art viscosity models in REFPROP 10.0.

The predictive capability of cubic EOS is influenced by the choice of the α function, an empirical temperature-dependent correlation that modifies the attractive term in cubic EOS to improve thermodynamic property predictions. Yang’s study^25^ exclusively utilized the traditional Soave α function across the four cubic EOS. To explore potential improvements, this work investigates the performance of different α functions within the cubic EOS + RES framework for viscosity prediction. The available combinations of cubic EOS and α functions are presented as follows. Bell et al.^34^ fitted the parameters of the Twu α function^35^ in PR EOS^26,27^ for 2570 pure fluids using experimental data from the NIST TDE (ThermoDataEngine) database.^36^ Mean average percentage deviation of approximately 7% for vapor pressure, 1% for latent heat of vaporization, and 1% for saturation specific heat were achieved.^34^ Mahmoodi and Sedigh^37^ summarized the existing α functions and proposed a new generalized and consistent α function for the cubic EOS of PR and SRK, designed to improve the accuracy of thermodynamic property predictions and address consistency issues in the derivatives of α functions against temperature. Additionally, the Heyen α function has been incorporated into the PTV EOS, resulting in the PTV-Heyen EOS.^38–40^ The Heyen function’s parameters are expressed in terms of acentric factors ω to improve the description of saturated thermodynamic properties of heavy hydrocarbons and other polar substances.

Based on the research mentioned above, this work evaluates the performance of different α functions in the cubic EOS + RES framework for viscosity prediction of 124 industrial important fluids. The results are compared with other models: the work of Yang,^25^ which employed the traditional Soave α function, and the state-of-the-art viscosity reference models implemented in REFPROP 10.0 (REF. models). By identifying the best-performing models for each pure fluid, this research establishes a robust foundation that will serve for developing viscosity models for over 600 pure fluids in the future. Part of this effort is funded by the KETEC (Research Platform Refrigeration and Energy Technology) project.^41^

## 2 Models

In this work, all the thermodynamic property calculations were performed via OilMixProp 1.0 software package,^42^ a tool designed to calculate key thermophysical properties of user-defined oils, common fluids, and their mixtures. The up-to-date code can be accessed free of charge by contacting developers of OilMixProp 1.0. The developed method in this work can be implemented in other thermophysical property software packages, including ThermoFAST.^43^

### 2.1 Viscosity Model

The molar residual entropy *s* ^res^ is defined as the difference between the entropy of a fluid at a given temperature *T* and density ρ and its ideal gas entropy in the same state

1 $$s^{\mathrm{res}}\left(T,\rho \right)=s\left(T,\rho \right)-s^{\mathrm{ig}}\left(T,\rho \right)$$

To facilitate correlation, the molar residual entropy is reduced to a dimensionless quantity *s* ^+^

2 $$s^{+}=-s^{\mathrm{res}}/R$$

where *R* is the universal gas constant (*R* = 8.314462618 J·mol^–1^·K^–1^).

The concept of the plus-scaled residual viscosity η_res_^+^ in eq 5 was first introduced by Bell based on isomorph theory.^11,16^ The calculation begins with determining the reduced residual viscosity, which is the difference between the viscosity of the fluid η­(*T*, ρ) and the dilute gas viscosity η_ρ→0_(*T*)­

3 $$\eta_{r}=\eta -\eta_{\rho \to 0}\left(T\right)$$

4 $$\eta_{\rho \to 0}/\left(\mathrm{Pa\cdot s}\right)=\sum_{i=0}^{4}a_{\eta ,i}{\left(T/K\right)}^{i}$$

5 $$\eta_{\mathrm{res}}^{+}=\frac{\eta_{r}}{\rho_{N}^{2/3}\sqrt{mk_{B}T}}\times {\left(s^{+}\right)}^{2/3}$$

Here the variable *m* represents the mass of one molecule, expressed in kilograms. The Boltzmann constant is given as *k*_B_ = 1.380649 × 10^–23^ J·K^–1^. The symbol ρ_N_ denotes the number density in units of m^–3^ and should not be confused with the molar density ρ, which is expressed in units of mol·m^–3^. The coefficients *a*_η,*i*_ (where *i* = 0, 1, 2, 3, and 4) are fitted to REFPROP 10.0 calculations for each pure fluid. These coefficients were determined for temperatures ranging from the triple point to either 1000 K or the maximum calculable temperature for the fluid. The values of *a*_η,*i*_ were originally published in the Supporting Information (SI) of Martinek et al.^21^ and are integrated into the OilMixProp 1.0 software package.^42^ Additionally, they are provided in the SI of this paper.

The relationship between the plus-scaled residual viscosity and the dimensionless residual entropy is established through correlation. Prior studies have demonstrated the effectiveness of employing a polynomial function,^21,25^ expressed as

6 $$\ln\left(\eta_{\mathrm{res}}^{+}+1\right)=n_{\eta ,f1}\cdot {{(s}^{+})}^{1.8}+n_{\eta ,f2}\cdot {{(s}^{+})}^{2.4}+n_{\eta ,f3}\cdot {{(s}^{+})}^{2.8}$$

7 $$\ln\left(\eta_{\mathrm{res}}^{+}+1\right)=n_{\eta ,g1}\cdot {{(s}^{+}/\xi_{\eta })}^{1.8}+n_{\eta ,g2}\cdot {{(s}^{+}/\xi_{\eta })}^{2.4}+n_{\eta ,g3}\cdot {{(s}^{+}/\xi_{\eta })}^{2.8}$$

The parameters *n*_η,f*i*_ and *n*_η,g*i*_ (*i* = 1, 2, 3) represent fluid-specific parameters for individual fluids and group-specific parameters for categories of similar fluids, respectively. Each pure fluid is assigned to a fluid group classified by Yang et al.^19^ and is associated with a corresponding fluid-specific scaling factor ξ_η_. Fluid-specific parameters *n*_η,f*i*_ are fitted only for fluids with sufficiently high-quality data. Only when such parameters are unavailable, the model utilizes group-specific parameters *n*_η,f*i*_ in conjunction with the fluid-specific scaling factor ξ_η_. The fitted parameters and η_res_^+^ vs *s* ^+^ curve of each model for each pure fluid investigated in this work are provided in the SI.

The eight fluid groups are broadly categorized as follows:

1. LG (Light Gases): Light gases exhibiting quantum effects at low temperatures, primarily hydrogen (including its spin isomers) and helium.
2. G (Gaseous Fluids): Noble gases and other gaseous fluids.
3. LHC (Light Hydrocarbons): This category includes light hydrocarbons such as straight-chain alkanes with up to five carbon atoms (C_1_–C_5_), branched alkanes and cycloalkanes with up to six carbon atoms (e.g., 2,2-dimethylbutane, cyclohexane), as well as related compounds of industrial relevance, including halogenated refrigerants (e.g., R11, R134a, *R*32) and small inorganic or heteroatomic molecules (e.g., CO_2_, COS, NF_3_).
4. B (Benzene-like Fluids and Small Molecules): Aromatic hydrocarbons and their derivatives (e.g., benzene, toluene, xylenes, chlorobenzene, ethylbenzene), as well as selected small nonaromatic compounds of industrial relevance, such as acetone and 1-butyne.
5. MHC (Medium Hydrocarbons): Medium hydrocarbons, including alkenes (e.g., 1-butene), straight-chain and branched alkanes with six to ten carbon atoms (C_6_–C_10_), and cyclic hydrocarbons in the same molecular weight range.
6. HHC (Heavy Hydrocarbons): Heavy hydrocarbons, high molecular weight compounds, and oils, typically with more than 16 carbon atoms (C_16_ and above), including long-chain alkanes (e.g., C_22_), cyclic siloxanes (e.g., D4, D5), methylated siloxanes (e.g., MDM), and fatty acid derivatives (e.g., methyl linoleate, methyl palmitate, methyl stearate).
7. LA (Lightly Associating Fluids): Fluids with weak intermolecular associations, such as methanol.
8. SA (Strongly Associating Fluids): Fluids with strong intermolecular associations, such as water.

### 2.2 Cubic EOS Model

#### 2.2.1 General Models

Cubic EOS were developed based on the ideal gas EOS by introducing correction terms to account for molecular interactions and finite molecular volume. The general form of cubic EOS expresses pressure as a function of molar volume and temperature, incorporating an attractive term to account for intermolecular forces and a repulsive term to correct for the finite size of molecules. A generalized 3-parameters cubic EOS is given as follows^30,31^

8 $$p=\frac{\mathrm{RT}}{v-b}-\frac{a}{v^{2}+\left(b+c\right)v-\mathrm{bc}}$$

Here *p* is pressure, *v* refers to molar volume, *a* represents the magnitude of intermolecular attractions, *b* accounts for excluded volume effects, and *c* is an additional parameter introduced in the 3-parameter cubic EOS to improve flexibility in representing different fluid behaviors.

Parameters *a*, *b*, and *c* are computed as

9 $$a=\alpha \left(T_{r},\omega ,Z_{c}\right)\cdot \Omega_{a}\frac{R^{2}T_{c}^{2}}{p_{c}}$$

10 $$b=\Omega_{b}\frac{{\mathrm{RT}}_{c}}{p_{c}}$$

11 $$c=\Omega_{c}\frac{{\mathrm{RT}}_{c}}{p_{c}}$$

Here *T*_r_, ω, and *Z*_c_ denote to the reduced temperature (*T*_r_ = *T*/*T*_c_), acentric factor, and critical compressibility factor. Critical point information (*T*_c_, *p*_c_, and *Z*_c_) for each fluid is sourced from REFPROP 10.0^8^ and is provided in the SIof this paper. The α function α­(*T*_*r*_, ω, *Z*_c_) is a temperature-dependent correction factor that modifies the attractive term in the cubic EOS to improve the thermodynamic property predictions.

For further calculations, the following terms are defined

12 $$d={\left[\mathrm{bc}+\frac{{\left(b+c\right)}^{2}}{4}\right]}^{1/2},q=\frac{b+c}{2}+d,m=\frac{b+c}{2}-d$$

The molar residual entropy *s* ^res^ is given by

13 $$s^{\mathrm{res}}=R\ln\frac{v-b}{v}-\frac{1}{2d}\frac{da}{dT}\ln\frac{v+m}{v+q}$$

where d*a*/d*T* is derived from the expression for *a* in eq 9.

This study evaluates four cubic EOS: PR,^26,27^ SRK,^28,29^ PTV,^30,31^ and YFR.^32^ Other cubic models, including Redlich–Kwong (RK),^29^ Wilson-Redlich–Kwong (WRK),^44^ and Peng–Robinson-Stryjek-Vera (PRSV)^45^ were excluded from this work due to, as noted by Yang,^25^ they either showed comparatively poor performance in the EOS + RES framework or lacked the necessary parameters for the 124 pure fluids analyzed in this work.

Each of the 4 cubic EOS employs a specific formulation for the α function α­(*T*_r_, ω, *Z*_c_) and utilizes characteristic constants Ω_a_, Ω_b_, and Ω_c_ for parameter calculation. The details are summarized in Table 1, where the α function follows the Soave formulation for the PR,^26,27^ PTV,^30,31^ SRK,^28,29^ and YFR^32^ EOS.

**Table 1:** Overview of the Evaluated Cubic Equations of State

EOS	Ω_a_	Ω_b_	Ω_c_	α (the Soave α function)
PR^26,27^	0.45724	0.07779	0.07779	14 $\alpha ={\left[1+m\cdot \left(1-{T_{r}}^{1/2}\right)\right]}^{2}$
				15 $m=0.37464+1.54226\omega -0.26992\omega^{2}\left(\omega \leq 0.49\right)$
				16 $m=0.37964+1.48503\omega -0.164423\omega^{2}+0.016666\omega^{3}\left(\omega >0.49\right)$
				
PTV^30,31^	0.66121 – 0.761057·*Z*_c_	0.02207 + 0.20868·*Z*_c_	0.57765 – 1.87080·*Z*_c_	17 $\alpha ={\left[1+m\left(1-{T_{r}}^{1/2}\right)\right]}^{2}$
				18 $m=0.46283+3.58230\omega Z_{C}+8.19417{\left(\omega Z_{c}\right)}^{2}$
				
SRK^28,29^	0.42748	0.08664	0	19 $\alpha ={\left[1+m\left(1-{T_{r}}^{1/2}\right)\right]}^{2}$
				20 $m=0.48508+1.55171\omega -0.15613\omega^{2}$
				
YFR^32^	*n*_Ω*a*,1_ = −0.174696	*n*_Ω*b*,1_ = 0.048371	*n*_Ω*c*,1_ = −0.434682	21 $\alpha ={\left[1+m\left(1-{T_{r}}^{1/2}\right)\right]}^{2}$
	*n*_Ω*a*,2_ = 0.156625	*n*_Ω*b*,2_ = −0.043334	*n*_Ω*c*,2_ = 0.389505	22 $m=2.779200Z_{c}+5.208803\omega Z_{c}-0.314477$
	*n*_Ω*a*,3_ = −1.158565	*n*_Ω*b*,3_ = 0.319103	*n*_Ω*c*,3_ = −2.872362	
	*n*_Ω*a*,4_ = 0.784751	*n*_Ω*b*,4_ = −0.012341	*n*_Ω*c*,4_ = 0.889348	
	*n*_*x*,1_ · exp(−*T*_r_^4^) + *n*_*x*,2_ · exp(−T_r_^3^) + *n*_*x*,3_·*Z*_*c*_ + *n*_*x*,4_			
	d*x*/d*T* = 0,(x = Ω*_a_*, Ω*_b_* and Ω*_c_*)			

#### 2.2.2 α Functions

As formulated in eq 9, the attractive term *a* can be regarded as the product of an α function and $\Omega_{a}\frac{R^{2}T_{c}^{2}}{p_{c}}$ where Ω*_a_* is EOS-dependent. Extensive research efforts have been dedicated to refining the α function to enhance its accuracy in thermodynamic property descriptions.^34,35,37,46^ In 1991, Twu et al. introduced an α function with parameters *C*_0_, *C*_1_, and *C*_2_ that can be tailored to meet critical consistency conditions.^35^ Its formula (eq 19) is presented in Table 2. Values of *C*_0_, *C*_1_, and *C*_2_ were fitted by Bell et al. for PR EOS for over 2500 pure fluids, which were determined via a multiproperty fitting process.^34^ Here this model is referred as PR-Twu. For the 124 fluids investigated in this work, if a substance is not included in the table of Bell et al.,^34^ the original PR EOS is employed for the calculations. These fluids are helium, nitrogen trifluoride, and R1224yd­(Z).

**Table 2:** List of the α Functions Studied in This Work

thermodynamic model	α functions
PR-Twu^26,27,34,35^	23 $\alpha ={\left(\frac{T}{T_{C}}\right)}^{C_{2}\left(C_{1}-1\right)}\exp\left\{C_{0}\left[1-{\left(\frac{T}{T_{C}}\right)}^{C_{1}C_{2}}\right]\right\}$
	values for over 2500 pure fluids is available in the work of Bell et al.^34^
	
PR-Coquelet^26,27,37,46^	24 $\alpha \left(T_{r}\right)=\exp\left[C_{1}\left(1-T_{r}\right)\right]{\left[1+C_{2}{\left(1-\sqrt{T_{r}}\right)}^{2}+C_{3}{\left(1-\sqrt{T_{r}}\right)}^{3}\right]}^{2}$
	25 $C_{1}=0.40464+1.3361\omega -0.07987\omega^{2}$
	26 $C_{2}=-0.08139+0.96493\omega -0.85454\omega^{2}$
	27 $C_{3}=0.31953+0.001007\omega -0.88858\omega^{2}$
	
SRK-Coquelet^28,29,37,46^	28 $\alpha \left(T_{r}\right)=\exp\left[C_{1}\left(1-T_{r}\right)\right]{\left[1+C_{2}{\left(1-\sqrt{T_{r}}\right)}^{2}+C_{3}{\left(1-\sqrt{T_{r}}\right)}^{3}\right]}^{2}$
	29 $C_{1}=0.53591+1.4492\omega -0.13969\omega^{2}$
	30 $C_{2}=-0.2741+0.59006\omega -0.54412\omega^{2}$
	31 $C_{3}=0.54293+0.53258\omega -1.4927\omega^{2}$
	
PR-MS^26,27,37^	32 $\alpha \left(T_{r}\right)=\exp\left\{2C_{1}\left(1-\sqrt{T_{r}}\right)-{\left[C_{2}\left(1-\sqrt{T_{r}}\right)\right]}^{2}+\frac{2}{3}{\left[C_{3}\left(1-\sqrt{T_{r}}\right)\right]}^{3}\right\}$
	33 $C_{1}=0.36818+1.4801\omega -0.14407\omega^{2}$
	34 $C_{2}=0.19422+1.9061\omega -0.46577\omega^{2}$
	35 $C_{3}=1.514\exp\left\{-{\left[\left(\omega -1.6645\right)/1.5474\right]}^{4}\right\}$
	
SRK-MS^28,29,37^	36 $\alpha \left(T_{r}\right)=\exp\left\{2C_{1}\left(1-\sqrt{T_{r}}\right)-{\left[C_{2}\left(1-\sqrt{T_{r}}\right)\right]}^{2}+\frac{2}{3}{\left[C_{3}\left(1-\sqrt{T_{r}}\right)\right]}^{3}\right\}$
	37 $C_{1}=0.47941+1.673\omega -0.23356\omega^{2}$
	38 $C_{2}=0.53827+2.1529\omega -0.7143\omega^{2}$
	39 $C_{3}=1.7482\exp\left\{-{\left[\left(\omega -1.456\right)/1.4542\right]}^{4}\right\}$
	
PTV-Heyen^38–40^	40 $\alpha \left(T\right)=\exp\left[H_{1}{(1-T_{r}}^{H_{2}}\right)]$
	41 $H_{1}=\frac{0.4653+1.26\omega -0.3928\omega^{2}}{H_{2}}$
	42 $H_{2}=\frac{-0.2981-1.9574\omega +0.1789\omega^{2}}{0.4563+1.26\omega -0.3928\omega^{2}}+1.4563+1.26\omega -0.3928\omega^{2}$

In 2004 Coquelet et al.^46^ combined Mathiase-Copeman^47^ and Trebblee-Bishnoi^48^ models to propose a new α function based on the reduced temperature. The correlation is given in eq 20 (Table 2). In 2017, Mahmoodi and Sedigh established an α function based on a Taylor series of the Soave relation [eq 28].^37^ Note that while originally referred to as the “PM” α function in their publication, it is abbreviated as the “MS” α function in this work for consistency with the authors’ last names. For this model, parameter *C*_3_ needs to be positive and smaller than 1.25|*C*_1_|. Mahmoodi and Sedigh provided correlations for the *C*_1_ to *C*_3_ parameters, enabling their application to PR and SRK EOS.^37^ The correlated relations of Coquelet and MS α function parameters are summarized in eqs 21 to 23 and 25 to 27 for the Coquelet model, and eqs 29 to 31 and 33 to 35 for the MS model in Table 2.

The Heyen α function can be combined with the PTV EOS, named as PTV-Heyen EOS.^38,39^ The parameters of the α function are expressed in terms of acentric factors and are presented in eqs 36 to 38 (Table 2). Forero G and Velásquez J proposed different *H* values for polar substances.^39^ In this work, the *H* parameters were computed using eqs^41^ and 38, as these values proved to yield more reliable results when combined with the RES for viscosity calculations.

## 3 Results and Discussion

Two statistical terms are utilized in this work to evaluate the results: AAD and BIAS. While AAD is an acronym, BIAS is a standard statistical term that quantifies the systematic deviation between experimental and calculated values. For a given experimental data η_*i*,exp_ and model calculation result η_*i*,calc_, AAD and BIAS can be computed as

43 $$\mathrm{AAD}=\frac{100\sum_{i=1}^{N_{\eta }}\left|\frac{\eta_{i,\exp}-\eta_{i,\mathrm{calc}}}{\eta_{i,\exp}}\right|}{N_{\eta }}$$

44 $$\mathrm{BIAS}=\frac{100\sum_{i=1}^{N_{\eta }}\frac{\eta_{i,\exp}-\eta_{i,\mathrm{calc}}}{\eta_{i,\exp}}}{N_{\eta }}$$

Here *N*_η_ is the total number of viscosity data points for a given substance. AAD measures the scatter of the data, whereas BIAS quantifies the extent of the model to systematically overpredict or underpredict experimental values.

The viscosity data set includes approximately 54,000 experimental viscosity data points for 124 pure fluids, primarily sourced from the NIST TDE database.^36^ In this work, the experimental data processing and parameter regression approach closely follow the methodology outlined in previous studies of viscosity.^19,20,25^ The data processing incorporates multiple filtering criteria, including limitation of EOS, phase consistency checks, outlier removal, and pressure constraints to ensure data reliability. A pressure limit of 60 MPa is set as a filter, as suggested by Yang,^25^ for data processing, because this threshold balances the trade-off between achieving a low AAD and retaining a high percentage of analyzable data. For consistency, this upper pressure limit of 60 MPa is applied to the computations of all models, including the state-of-the-art models in REFPROP 10.0 (REF. model).

The parameter regression approach involves fitting fluid-specific and group-specific parameters in eqs 6 and 7 within the residual entropy scaling framework. Model parameters are anchored to experimental data; the reference list for all the experimental data used in this work is available in the SI (Reference list.docx).

### 3.1 Model Performance

A model performance overview for all processed experimental data of all studied fluids is provided in Figure 1. The results indicate that the performance ranking follows the order: REF. models, cubic EOS (with the traditional Soave α function) + RES, and cubic EOS-other α function + RES. The REF. models achieve the lowest AAD (2.7%), indicating high overall agreement with experimental viscosity data. However, they exhibit the largest absolute BIAS values among all the models (more than +0.6%).

**Figure 1:**
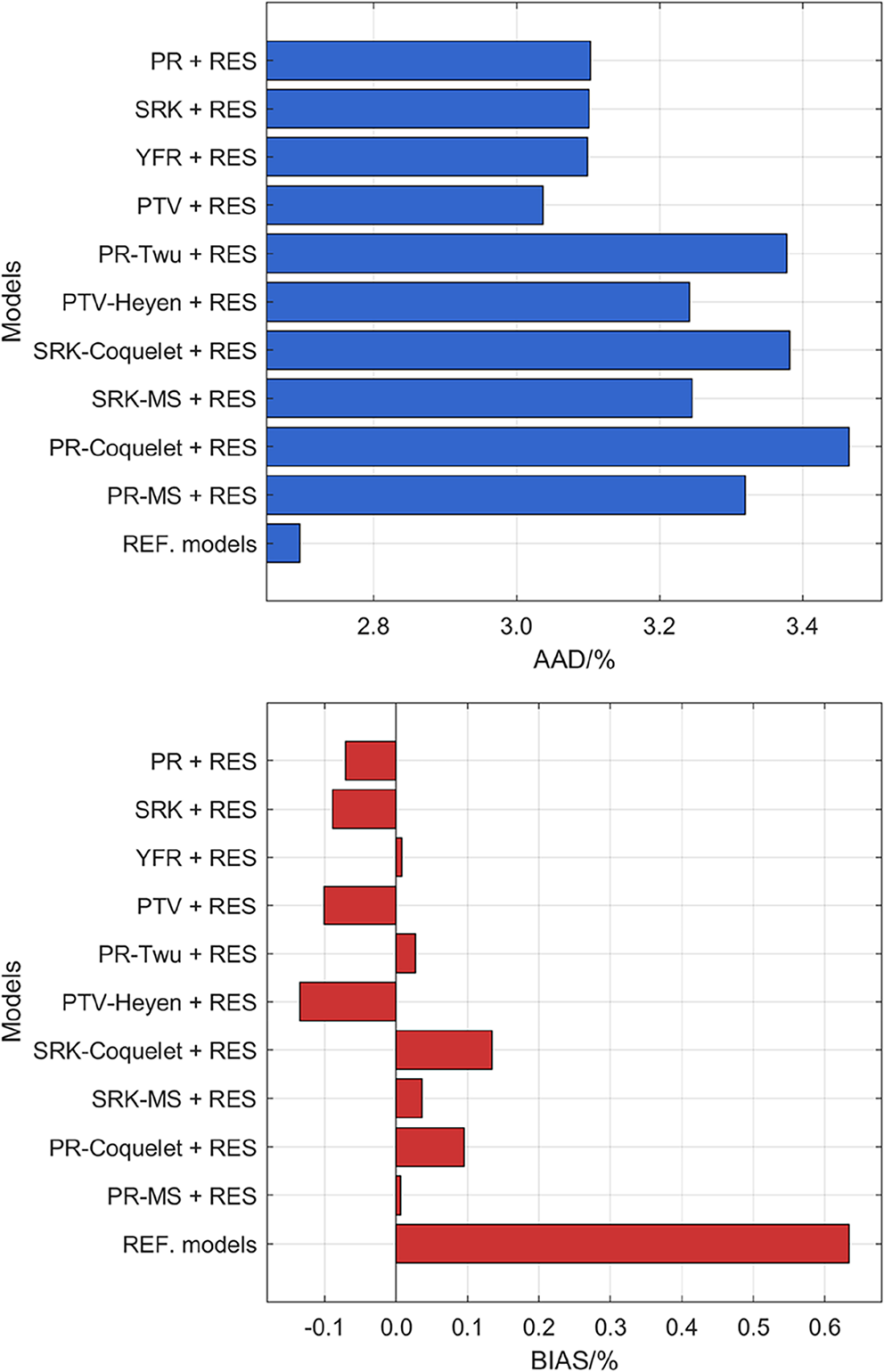
AAD and BIAS for all processed viscosity data computed from different models.

Among the cubic EOS models, the PTV + RES approach yielded the lowest AAD (3.0%), followed by the YFR + RES, SRK + RES, and PR + RES approaches (3.1%). However, the YFR + RES approach demonstrates the best predictive performance for largest number of pure fluids (18 fluids as summarized in the second column of Table 3). Originally developed by Yang et al.^32^ for liquid density calculations, the YFR EOS performs well when integrated with RES, making it particularly effective for industrially relevant fluids such as 1-butene, fluorine, helium, krypton, pentane, and R134a. In Yang’s work^25^ of examining viscosities based on residual entropy scaling and cubic EOS, the RES model was also extended to approximately 26 additional pure fluids—those having multiparameter EOS in REFPROP 10.0 but lacking experimental viscosity data. This anchoring strategy was not adopted in the present study, as our focus was limited to fluids with available experimental viscosity data. In addition, reproducibility of Yang’ work^25^ was carried out. Due to the removing of very few repeated data, the resulting AAD and BIAS values differ slightly from those reported by Yang.^25^

**Table 3:** Summary of the Best Viscosity Model (Based on AAD) for Each Substance^a^

models	best-performing substances (including REFPROP models)	best-performing substances (excluding REFPROP models)
PR + RES	DEA, R11, R124, R21, R41	BENZENE, BUTANE, CO, DEA, MSTEARAT, NEOPENTN, R11, R124, R21, R41
SRK + RES	ACETONE, ACETYLENE, C6F14, CF3I, MM, MOLEATE, R114, R115, R22	ACETONE, ACETYLENE, C11, C12, C1CC6, C6F14, CF3I, CYCLOPEN, DECANE, DMC, EBENZENE, ETHYLENE, HEPTANE, IOCTANE, MLINOLEA, MM, MOLEATE, MXYLENE, NONANE, OCTANE, OXYLENE, PXYLENE, R114, R115, R1233ZDE, R22, RE347MCC, TOLUENE
YFR + RES	1BUTENE, C2BUTENE, C5F12, CHLOROBENZENE, CYCLOPRO, D5, ETHYLENEOXIDE, FLUORINE, HELIUM, IBUTENE, KRYPTON, PENTANE, PROPYLENEOXIDE, R134A, R236EA, R245CA, RC318, RE245FA2	13BUTADIENE, 1BUTENE, 22DIMETHYLBUTANE, 3METHYLPENTANE, AMMONIA, C2BUTENE, C5F12, CHLOROBENZENE, CYCLOPRO, D5, ETHYLENEOXIDE, FLUORINE, H2S, HELIUM, IBUTENE, KRYPTON, MLINOLEN, NEON, PENTANE, PROPYLENEOXIDE, PROPYNE, R1224YDZ, R125, R1336MZZZ, R134A, R142B, R143A, R152A, R23, R236EA, R245CA, R245FA, R40, RC318, RE245FA2
PTV + RES	CYCLOHEX, DEE, N2O, R113, R141B, R161, XENON	CYCLOHEX, DEE, HEXANE, HYDROGEN, IHEXANE, N2O, Overall, R113, R141B, R161, R218, R236FA, XENON
PR-Twu + RES	D4, ETHANOL, METHANOL, R150	CO2, D4, ETHANE, ETHANOL, HCL, METHANOL, R150, *R*32
PTV-Heyen + RES	R12, R1234ZEE, R227EA, SO2	CHLORINE, D2O, DME, ISOBUTAN, METHANE, NITROGEN, R12, R1234YF, R1234ZEE, R227EA, SO2, WATER
SRK-Coquelet + RES	EGLYCOL, IPENTANE, OXYGEN	C16, C22, EGLYCOL, IPENTANE, OXYGEN
SRK-MS + RES	MEA, MPALMITA, R116, R123	23DIMETHYLBUTANE, ARGON, MEA, MPALMITA, R116, R123, R13
PR-Coquelet + RES	MDM, NF3, R14	MDM, NF3, R14, SF6
PR-MS + RES	PROPANE, PROPYLEN	D2, PROPANE, PROPYLEN
REF. models	13BUTADIENE, 22DIMETHYLBUTANE, 23DIMETHYLBUTANE, 3METHYLPENTANE, AMMONIA, ARGON, BENZENE, BUTANE, C11, C12, C16, C1CC6, C22, CHLORINE, CO, CO2, CYCLOPEN, D2, D2O, DECANE, DMC, DME, EBENZENE, ETHANE, ETHYLENE, H2S, HCL, HEPTANE, HEXANE, HYDROGEN, IHEXANE, IOCTANE, ISOBUTAN, METHANE, MLINOLEA, MLINOLEN, MSTEARAT, MXYLENE, NEON, NEOPENTN, NITROGEN, NONANE, OCTANE, OXYLENE, Overall, PROPYNE, PXYLENE, R1224YDZ, R1233ZDE, R1234YF, R125, R13, R1336MZZZ, R142B, R143A, R152A, R218, R23, R236FA, R245FA, *R*32, R40, RE347MCC, SF6, TOLUENE, WATER	NA

a Note: Substance names follow the REFPROP 10.0 convention. The corresponding IUPAC names and CAS Registry Numbers are provided in the Supporting Information.

Compared to the traditional Soave α function, the modified α functions (Twu, Coquelet, MS and Heyen) do not always lead to improved viscosity predictions. One possible reason is that these α functions were primarily developed to enhance vapor–liquid equilibrium (VLE) predictions. They were not specifically designed for density and residual entropy calculations, which are essential for the cubic EOS + RES framework. Despite their lower overall performance, cubic EOS with modified α functions still provide improved predictions for specific substances. As indicated in the second column of Table 3, PTV-Heyen + RES has the lowest AAD for R1234ze­(E), SO_2_, and R12; SRK-MS + RES and PR-MS + RES yields best viscosity description for MEA and propane, respectively. Additional bar charts showing the AAD, BIAS, and successful calculation rates (i.e., the ratio of analyzable data to the total available data for each model and fluid) are provided in the SI. Relative deviations of the analyzable experimental data from both the RES model and the models in REFPROP 10.0 for each pure fluid are also included in the SI.

The best cubic EOS + RES model (evaluated by the lowest AAD, without considering REF. models) for each pure fluid is listed in the third column of Table 3. The exclusion of REF. models is primarily due to their limitations in practical applications. While REF. models provide overall the most accurate viscosity predictions, they are relatively hard to be implemented in major commercial process simulation software such as HYSYS^49^ due to their weak documentation for reproducibility. Additionally, the formulations of REF. models are relatively complex and not straightforward to use without access to proprietary software like REFPROP 10.0. These factors make cubic EOS + RES models, which are widely supported and computationally efficient, more attractive for industrial application and academic development.

Among the cubic EOS + RES models, SRK + RES demonstrates strong predictive capabilities for a wide range of hydrocarbons (e.g., decane, nonane, and toluene), and refrigerants such as R1233zd­(E) and R115. YFR + RES stands out for its effectiveness in predicting viscosities for noble gases (e.g., helium and krypton), unsaturated hydrocarbons (e.g., 1-butene and 2-methyl-1-propene), and a variety of refrigerants, including R134a and R125. This model’s strong performance is attributed to its foundation in liquid density calculations, which aligns well with the thermodynamic behavior of these substances. The replacement of Soave to other α functions improved predictions for 39 substances. Among them, PTV-Heyen + RES predicts 12 substances best, including R1234yf, SO_2_, and water, while PR-Twu + RES is notably effective for CO_2_, a fluid of significant industrial importance.

The grouping of fluids, i.e., clustering fluids with similar thermodynamic and intermolecular interaction characteristics, was primarily introduced to improve the quality of model fitting. Our analysis reveals that REFPROP models perform best for most of fluids in groups 1, 3, 4, 5, and 8, while different RES-based approaches excel in other groups: PTV + RES for group 2, PR + RES for group 6, and PR-Twu + RES for group 7. Notably, the REFPROP models demonstrate a marked advantage for group 8 fluids (i.e., strongly associating fluids such as water) with an AAD of 0.9%, in contrast to at least 2.4% of the best-performing RES-based model (PTV-Heyen + RES). Similarly, REFPROP models (2.2% AAD) outperform the best RES model (SRK + RES with 3.2% AAD) for fluids in group 5. The observed variations among fluid groups indicate the necessity of further refining the RES-based approaches for specific group fluids, particularly for groups 5 and 8.

Table 3 provides a practical reference for model selection in simulations. For each fluid, the most accurate model—evaluated by the lowest AAD when REF. models are included—is listed in the second column. It is recommended that simulation tools assign viscosity models based on this column to ensure optimal accuracy. When REF. models are unavailable, the third column offers alternative model choices using only cubic EOS + RES approaches, which are widely supported in commercial software. In practical simulations involving fluid mixtures, the PTV model is recommended, as it demonstrates the most consistent performance across all substances and is therefore suitable for general-purpose application.

### 3.2 Case Studies of CO_2_, R1234zee, MEA, and SO_2_

A detailed analysis is presented for four fluids of significant industrial relevance: CO_2_, R1234ze­(E), MEA, and SO_2_. These fluids were selected based on two criteria. First, each plays an important role in practical applications—CO_2_ in carbon capture and energy systems, R1234ze­(E) and SO_2_ in refrigeration, and MEA in chemical absorption and gas processing. Second, these fluids represent cases where the use of cubic EOS combined with modified α functions (e.g., Twu, Heyen, MS) resulted in markedly improved viscosity predictions compared to models using the traditional Soave α function.

#### 3.2.1 CO_2_ with PR-Twu + RES

The performance of all studied models for viscosity prediction of CO_2_ is provided in Figure 2. It can be seen that the PR-Twu + RES model demonstrates commendable performance, offering a simpler and computationally efficient alternative to the reference model^50^ in REFPROP 10.0 for pressures below 60 MPa. This model achieves a smaller BIAS (in absolute values), indicating that its predictions, on average, are closer to experimental values in a systematic manner. However, it has a higher AAD, meaning that individual deviations from experimental data vary more widely. This suggests that while the model may provide a more balanced estimate overall, it exhibits greater variability in accuracy across different data points.

**Figure 2:**
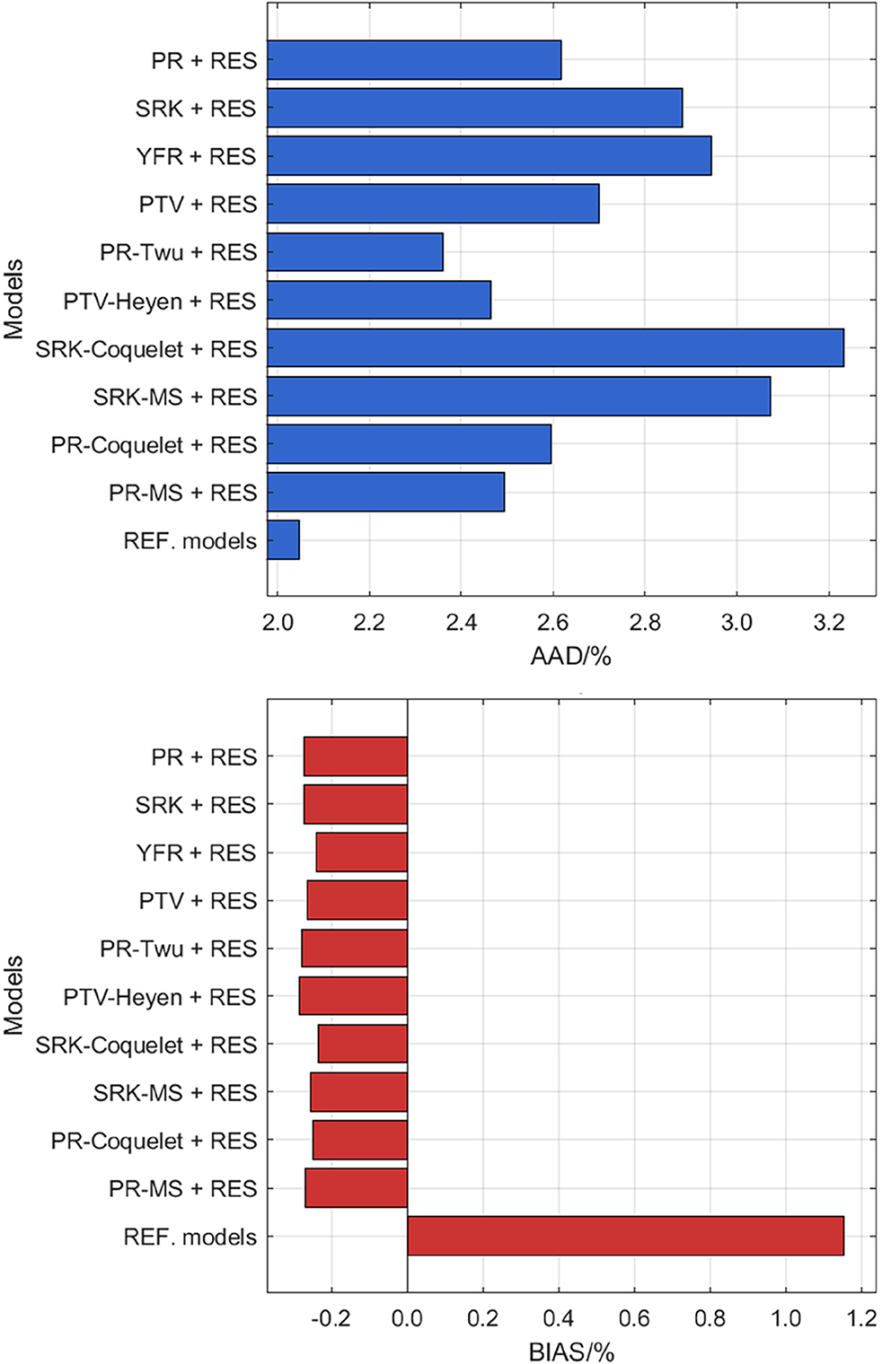
AAD and BIAS for the viscosity data of CO_2_ computed from different models.

Relative deviations of the experimental data of CO_2_ from the PR-Twu + RES model and the REF. model^50^ are illustrated in Figure 3. It can be seen that PR-Twu + RES exhibits different performance across different phases. While the model of PR-Twu + RES may not perform as well as the reference model in the gas phase (small *s* ^+^ up to 0.60), it demonstrates similar predictive capabilities near the critical point (middle *s* ^+^ between 0.31 and 2.78) and the liquid phase (high *s* ^+^ between 1.55 and 4.28). It should be noted that the *s* ^+^ ranges associated with each phase may overlap, as reduced entropy alone is not a definitive indicator of phase state, which also depends on the specific temperature, pressure, and fluid properties. Importantly, in the higher *s* ^+^ region, the deviations of PR-Twu + RES are relatively well-distributed and do not exhibit excessive scattering, with almost all deviations within 10%. Considering that the reference model^50^ is finely tuned with multiple adjustable parameters, the same-quality performance of PR-Twu + RES, achieved with a simpler framework, highlights its practical utility. The balance of accuracy, consistency, and computational efficiency suggests that PR-Twu + RES should be recommended, particularly for applications requiring reliable and straightforward viscosity predictions for CO_2_.

**Figure 3:**
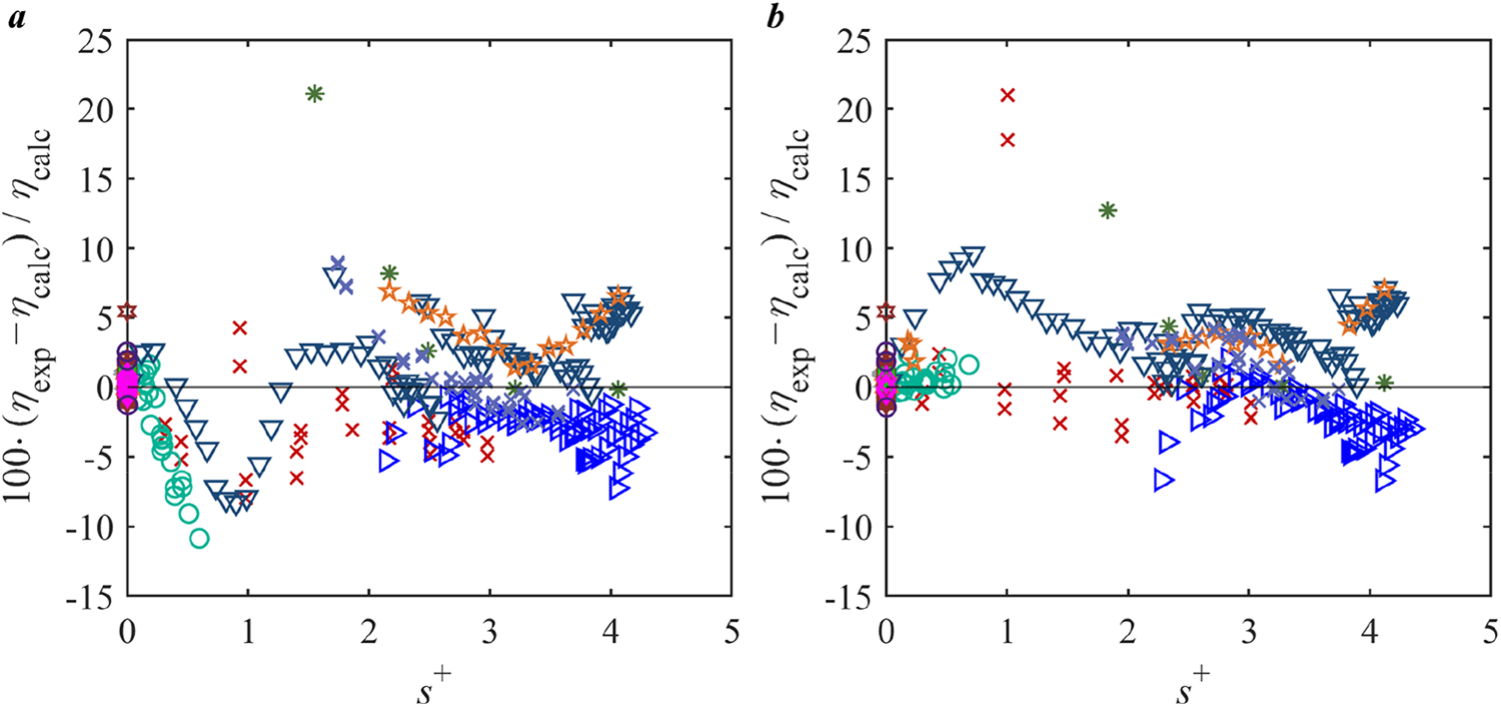
Deviation of the viscosity literature data of CO_2_ from *a*, PR-Twu + RES model; *b*, REF. correlation.^50^ Legend: single phase: Black Square Gururaja et al.;^51^ Purple Diamond Open Borisov et al.;^52^ Red Cross Kurin and Golubev;^53^ Green Circle Open Timrot and Traktueva;^54^ Orange Triangle Up Open Kestin et al.;^55^ Blue Triangle Right-Pointing Open Ulybin and Makarushkin;^56^ Gray Triangle Left-Pointing Open Kestin et al.;^57^ Purple Plus Harris et al.;^58^ Blue Triangle Down Open Diller and Ball;^59^ Brown Six Pointed Star Lusternik and Kuznetsov;^60^ Green Box Hobley et al.;^61^ Pink Diamond Open Hunter et al.;^62^ Blue Cross Padua et al.;^63^ Blue Circle Open Mal’tsev et al;^64^ liquid in equilibrium with gas: green star Rabe;^65^ Brown Outlined White Star Diller and Ball.^59^

#### 3.2.2 R1234ze­(E) with PTV-Heyen + RES

The performance of all studied models for viscosity prediction of R1234ze­(E) is provided in Figure 4, and relative deviations of the experimental data from the PTV-Heyen + RES model and the reference model^66^ in REFPROP 10.0 are illustrated in Figure 5. As shown in Figure 4, PTV-Heyen + RES achieves lower AAD compared to the reference model,^66^ indicating its ability to provide accurate viscosity predictions for R1234ze­(E) while using a simpler formation and fewer parameters. Figure 5 clearly shows that the primary limitation of the PTV-Heyen + RES model for R1234ze­(E) is the inaccurate prediction of dilute gas viscosity (small *s* ^+^). The underestimation of gas-phase viscosity is not fully offset by the positive deviations observed in the liquid phase, leading to a relatively large absolute BIAS value. The reference model^66^ systematically overestimates liquid-phase viscosities while underestimating gas-phase viscosities. This discrepancy is largely due to the fact that the reference model^66^ was primarily fitted to the liquid data from Meng et al.,^67^ which leads to larger deviations for the other two liquid phase data sets. In the gas phase, the reference model cannot describe the viscosities well either, especially for those data in the dilute gas region.

**Figure 4:**
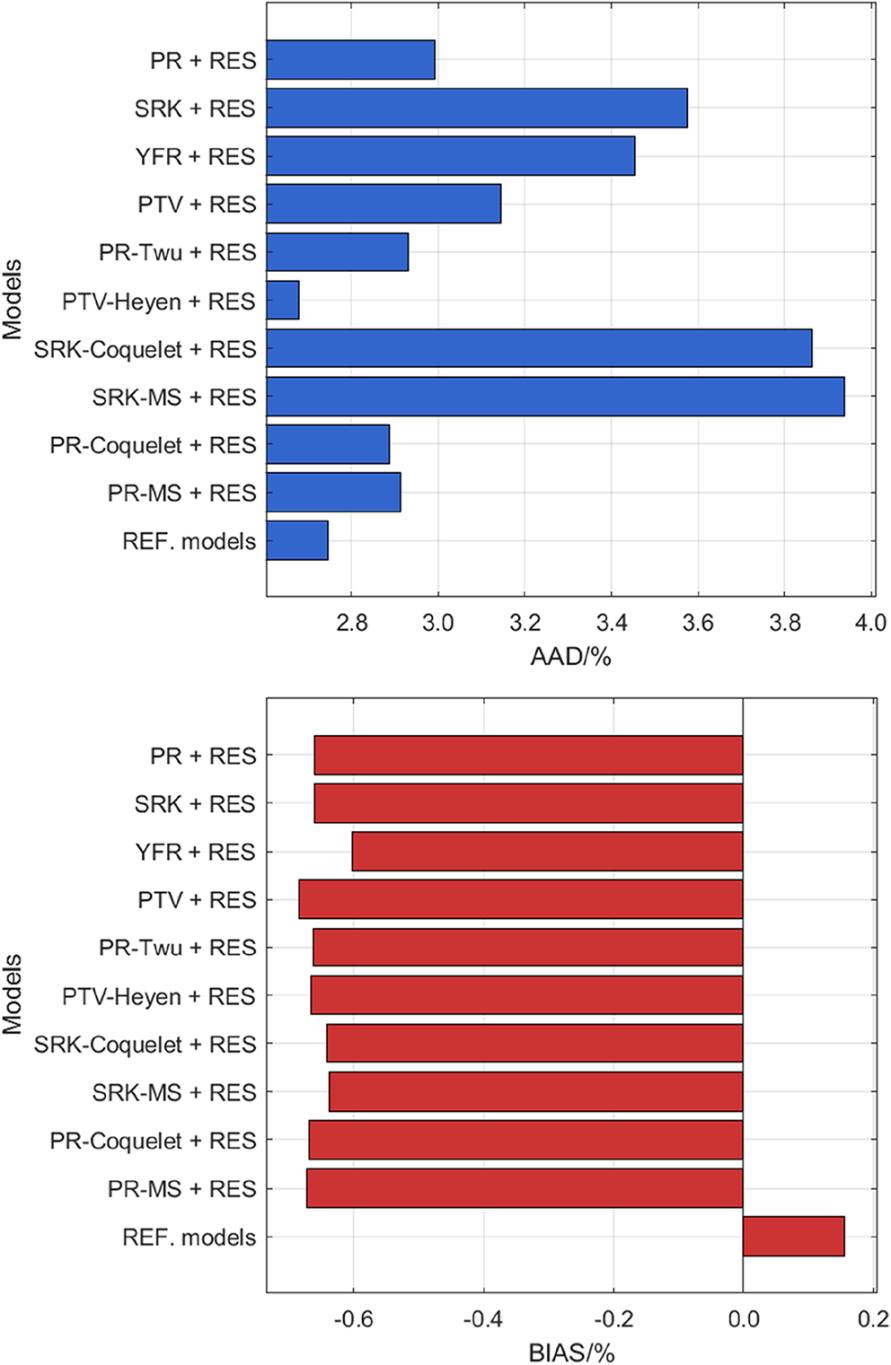
AAD and BIAS for the viscosity data of R1234ze­(E) computed from different models.

**Figure 5:**
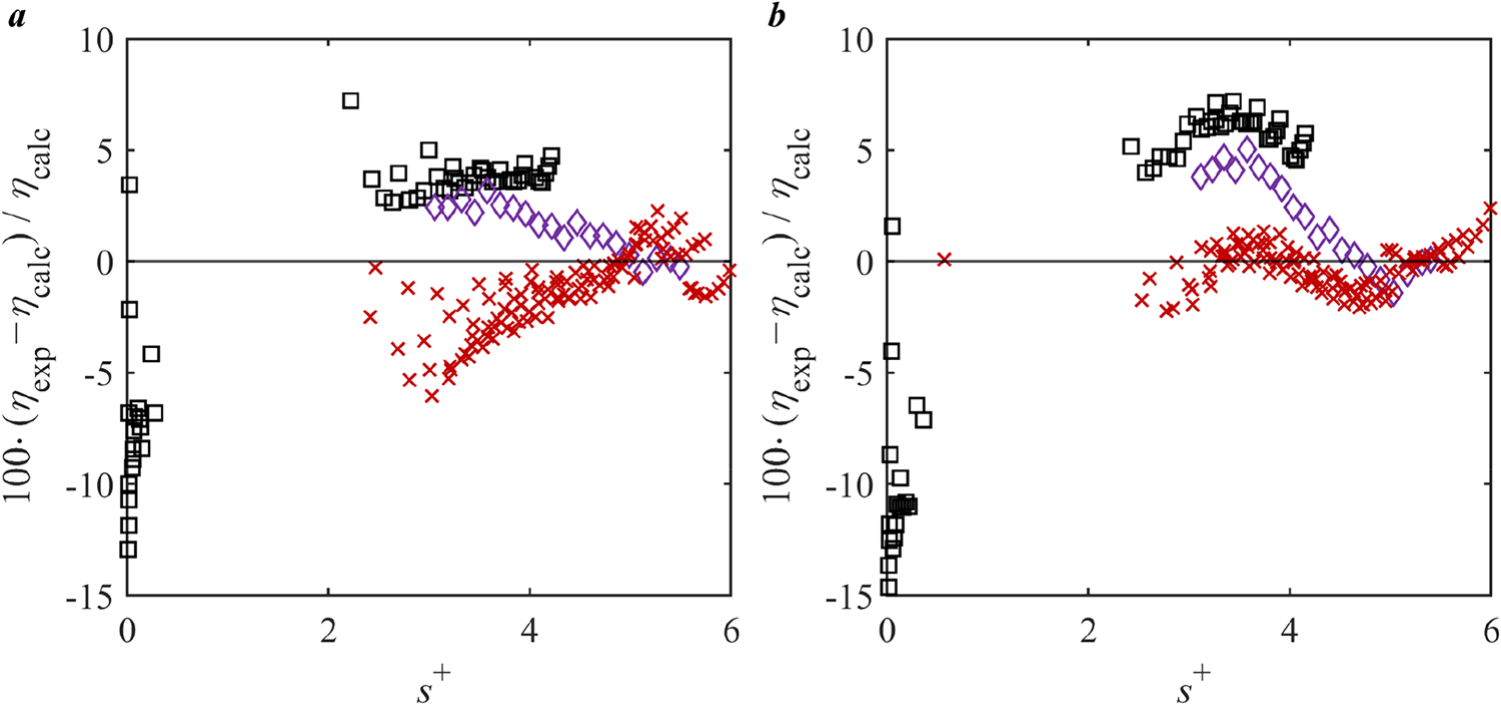
Deviation of the viscosity literature data of R1234ze­(E) from *a*, PTV-Heyen + RES model; *b*, REF. correlation.^66^ Legend: single phase: Black Square Grebenkov et al.;^68^ Red Cross Meng et al.;^67^ liquid in equilibrium with gas: Purple Diamond Open Cousins and Laesecke.^69^

Given these observations, a priority in the future work should be improving the dilute gas viscosity model of R1234ze­(E) to enhance overall accuracy. Despite its limitations, the PTV-Heyen + RES model demonstrates strong performance in the liquid phase, with nearly all literature data predicted within 5% deviation. Therefore, it remains a recommended choice for R1234ze­(E) viscosity computations, provided that refinements are made to the dilute gas viscosity predictions.

#### 3.2.3 MEA with SRK-MS + RES

The performance of all studied models for viscosity prediction of MEA (monoethanolamine) is provided in Figure 6, and relative deviations of the experimental data from the SRK-MS + RES model and the reference model^70^ in REFPROP 10.0 are illustrated in Figure 7. In Figure 6, it is evident that many models outperform the reference model for MEA viscosity predictions. Notably, SRK-MS + RES achieves the best performance with the lowest AAD among all models. In Figure 7, the deviations for SRK-MS + RES are mostly distributed within 5% from the literature data, while the reference model^70^ exhibits a wider spread of deviations with more pronounced scattering. Such behavior is also reflected in the BIAS plot in Figure 6 that the reference model^70^ is of a significantly higher BIAS value than SRK-MS + RES. The strong performance of SRK-MS + RES underscores the advantage of cubic EOS + RES framework for predicting viscosities of lightly associating fluids (categorized as Group 7).

**Figure 6:**
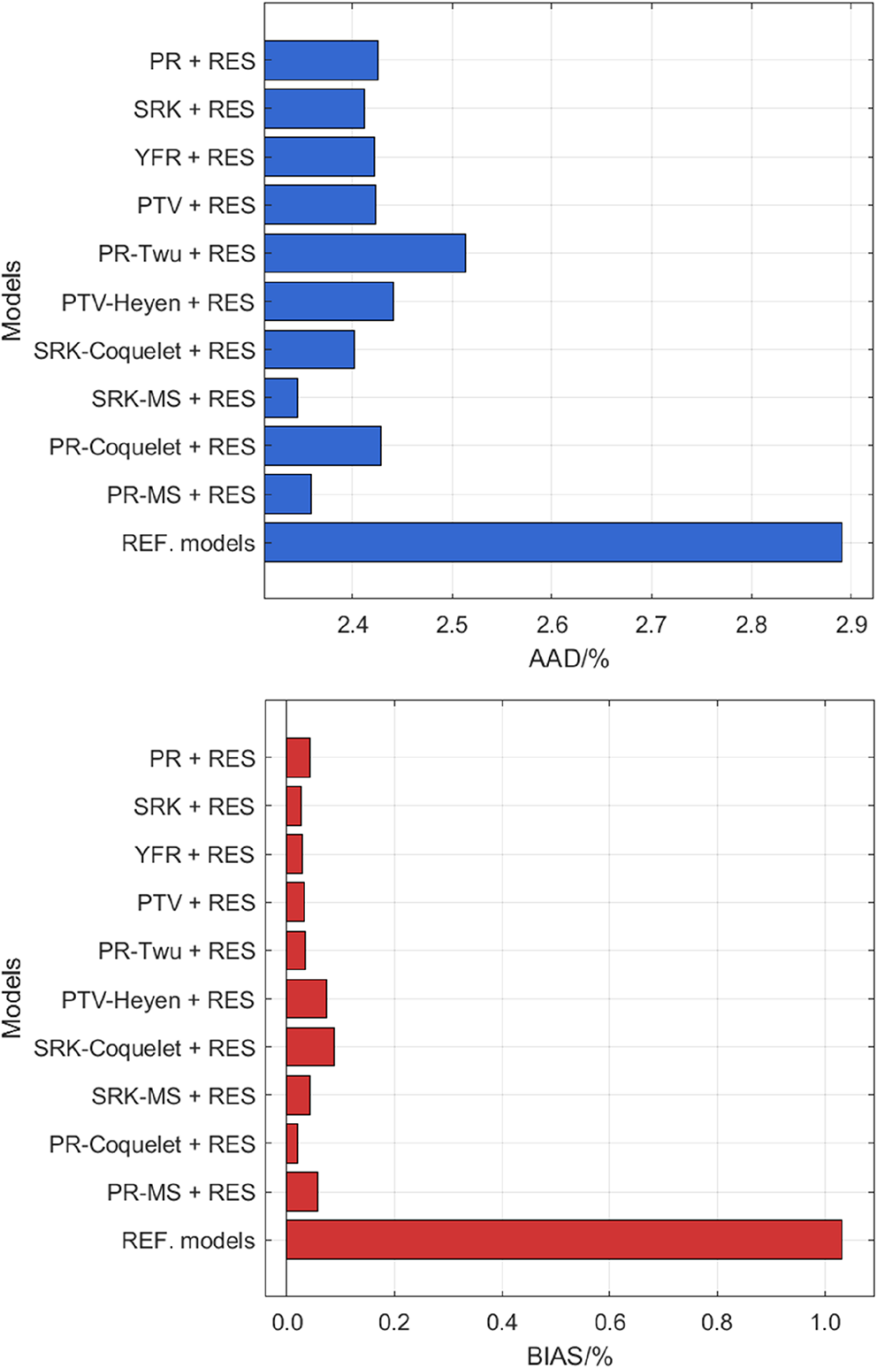
AAD and BIAS for the viscosity data of MEA computed from different models.

**Figure 7:**
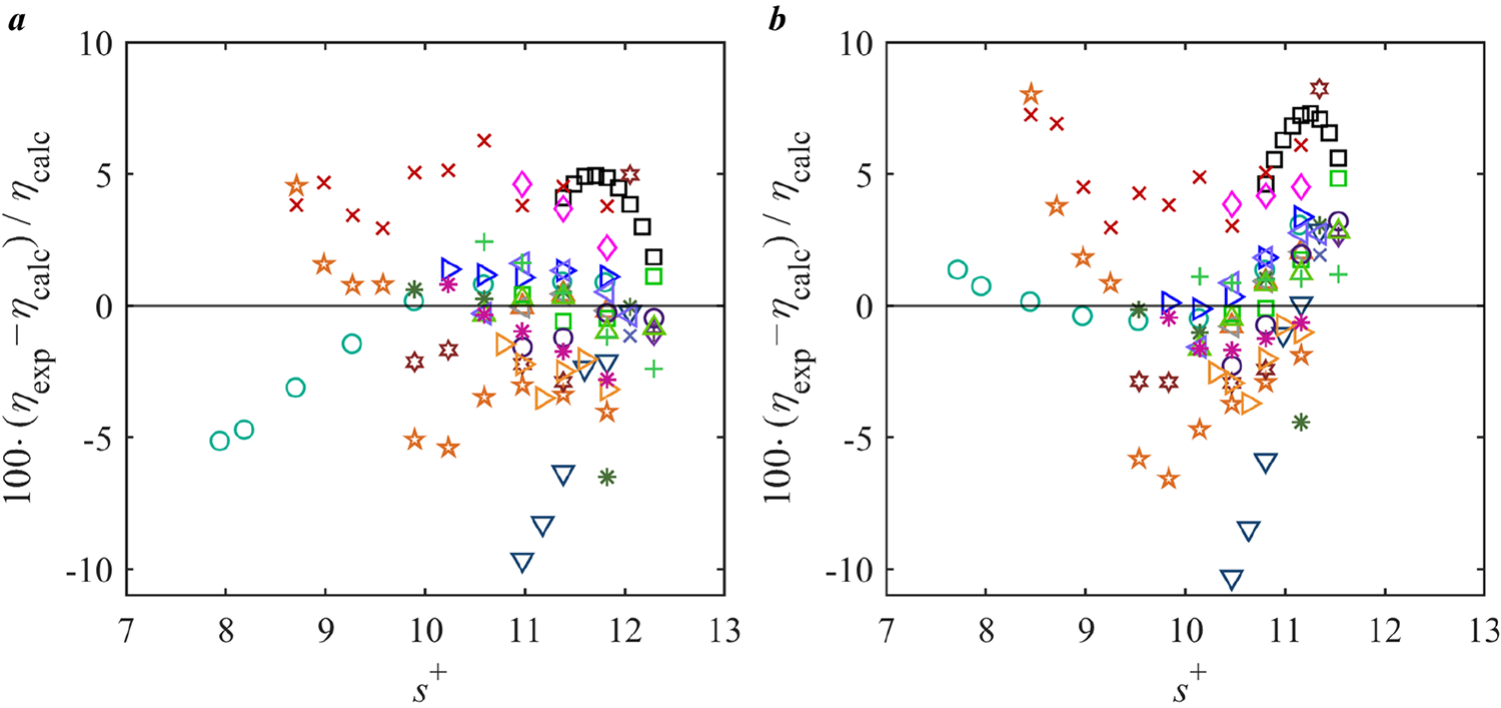
Deviation of the viscosity literature data of MEA from *a*, SRK-MS + RES model; *b*, REF. correlation.^70^ Legend: single phase: Black Square Matthews et al.;^71^ Red Cross Anonymous;^72^ Green Open Circle DiGuilio et al.;^73^ Orange Triangle Up Open Lee and Lin;^74^ Blue Triangle Right-Pointing Open Song et al.;^75^ Gray Triangle Left-Pointing open Lee et al.;^76^ Purple Plus Tsierkezos and Molinou;^77^ Dark Green Eight Spoked Asterisk Maham et al.;^78^ Blue Triangle Down Open Islam et al.;^79^ Brown Star Open Aguila-Hernandezet al.;^80^ Brown Six Pointed Star Amundsen et al.;^81^ Green Box Garcia-Abuin et al.;^82^ Pink Diamond Open Song et al.;^83^ Blue Cross Blanco et al.;^84^ Purple Open Circle Blanco et al.;^85^ Green Triangle Up Open Li et al.;^86^ Brown Triangle Right-Pointing Open Tian et al.;^87^ Blue Triangle Left-Pointing Open Shaikh et al.^88^ Green Plus Xu et al.;^89^ Pink Eight Spoked Asterisk Shen et al.;^90^ liquid in equilibrium with gas: Blue Diamond Open Anonymous.^91^

#### 3.2.4 SO_2_ with PTV-Heyen + RES

The performance of all studied models for viscosity prediction of SO_2_ is provided in Figure 8, and relative deviations of the experimental data from the PTV-Heyen + RES model and the reference model^70^ in REFPROP 10.0 are illustrated in Figure 9. From Figure 8, it is noticeable that all the cubic EOS + RES models exhibit significantly better performance than the reference model^70^ in terms of AAD. Among the models, PTV-Heyen + RES achieves the best performance, with an AAD of 3.7% and a BIAS of 0.35%. While the low BIAS indicates that the model does not exhibit a systematic overestimation or underestimation, the AAD value of 3.7% remains relatively high, suggesting that further refinements are necessary. In Figure 9, the PTV-Heyen + RES model demonstrates a relatively balanced distribution of deviations across the entire *s* ^+^ range, capturing the liquid-phase behavior of SO_2_ with reasonable accuracy. However, noticeable fluctuations in deviations are observed in the low *s* ^+^ area (corresponding to the gas phase), where the model exhibits larger prediction errors up to 30% in the dilute gas region. The reference model^70^ shows higher scattering across the data, leading to higher AAD values overall. A refined dilute gas viscosity correlation could reduce the deviations of both models for better viscosity prediction.

**Figure 8:**
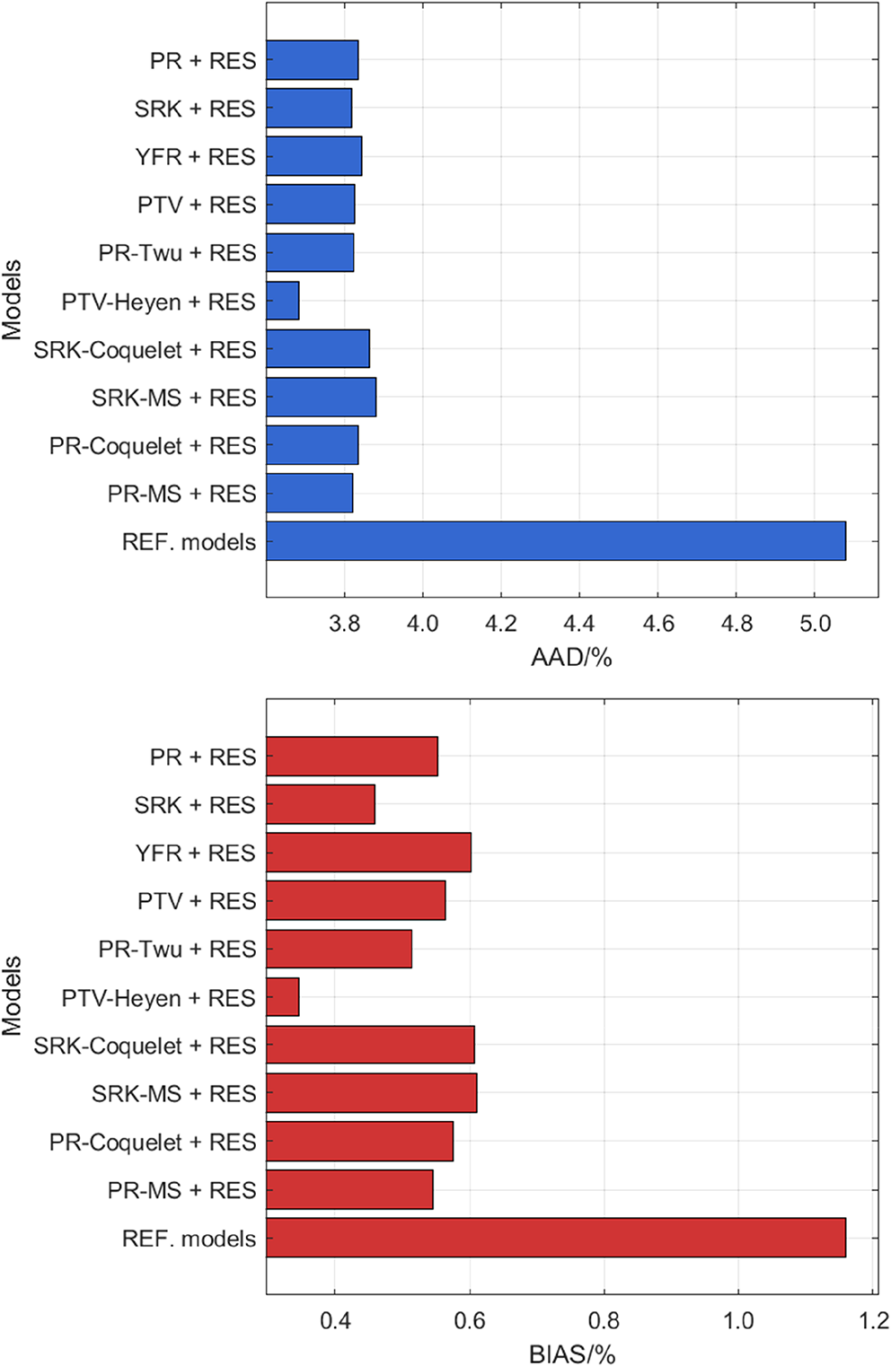
AAD and BIAS for the viscosity data of SO_2_ computed from different models.

**Figure 9:**
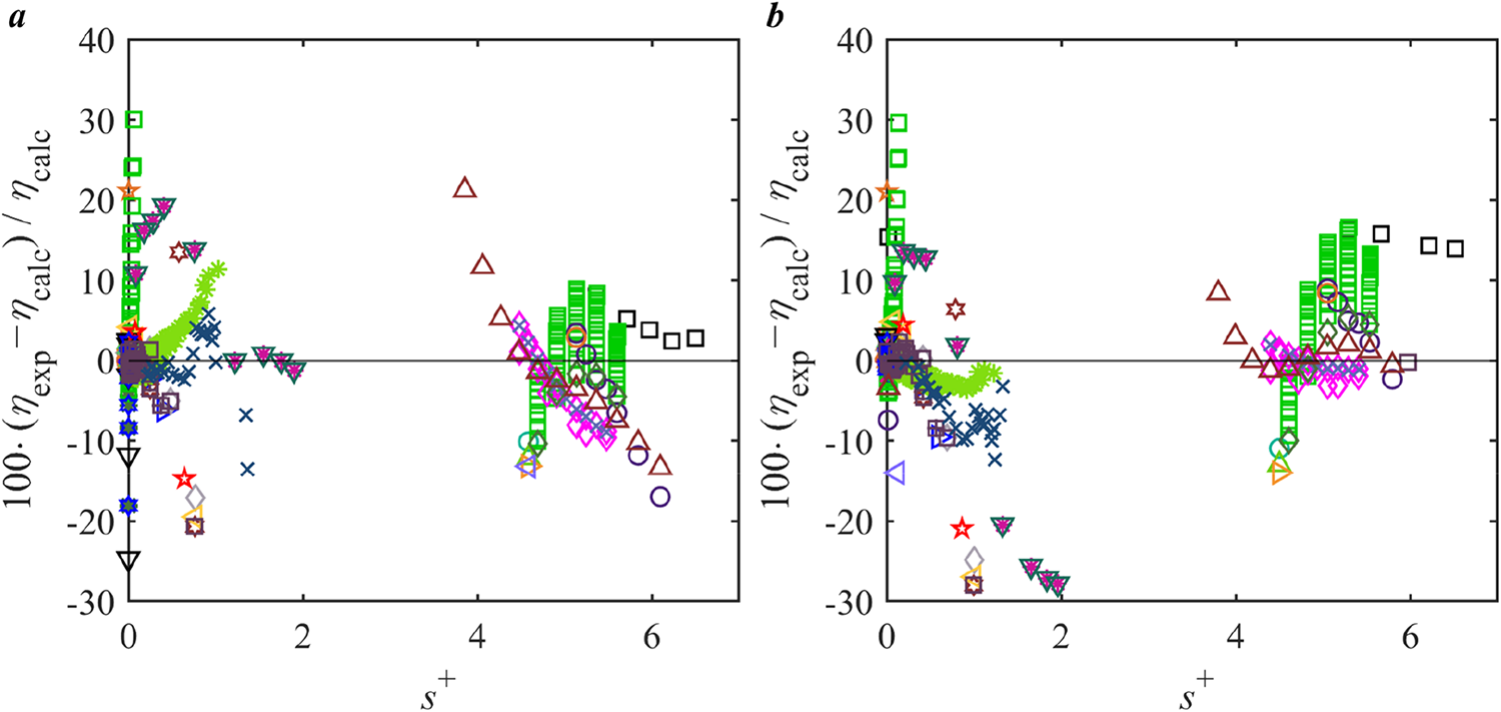
Deviation of the viscosity literature data of SO_2_ from *a*, PTV-Heyen + RES model; *b*, REF. correlation.^70^ Legend: single phase: Purple Diamond Open Vogel;^92^ Orange Triangle Up Open Trautz and Weizel;^93^ Gray Left-Pointing Open Jung and Schmick;^94^ Dark Green Eight Spoked Asterisk Trautz and Zink;^95^ Brown Star Open Trautz and Winterkorn;^96^ Green Box Stakelbeck;^97^ Orange Triangle Right-Pointing Open Wobser and Mueller;^98^ Blue Triangle Left-Pointing Open Shimotake and Thodos;^99^ Green Plus Chakraborti and Gray;^100^ Blue Triangle Down Open Pal and Barua;^101^ Blue Six Pointed Star Bhattacharyya et al.;^102^ Pink Cross Chang et al.;^103^ Green Triangle Up Open Kestin et al.;^104^ Green Triangle Right-Pointing Open Iwasaki and Takahashi;^105^ Yellow Triangle Left-Pointing Open Clifford et al.;^106^ Green Eight Spoked Asterisk Scholz and Kley;^107^ Blue Six Pointed Star Takahashi et al.;^108^ gas in equilibrium with liquid: Red Cross Smith;^109^ Blue Triangle Right-Pointing Open Trautz and Weizel;^93^ Purple Plus Titani;^110^ Dark Blue Triangle Down Open Trautz and Zink;^95^ Brown Six Pointed Star Trautz and Winterkorn;^96^ Red Eight Spoked Asterisk Chakraborti and Gray;^100^ Blue Outlined White Star Pal and Barua;^101^ Orange Box Bhattacharyya et al.;^102^ Gray Diamond Open Bhattacharyya;^111^ Brown Open Circle Chang et al.;^103^ Yellow Plus Clifford et al.;^106^ Black Triangle Down Open Scholz and Kley;^107^ liquid in equilibrium with gas: Black SquareScheuer;^112^ Green Open Circle Lewis;^113^ Pink Diamond Open Awbery and Griffiths;^114^ Blue Cross Luchinski;^115^ Purple Open Circle Adams and Rogers**;** ^116^ Green Triangle Up Open Cupp and Rogers;^117^ Red Outlined White Star Scholz and Kley;^107^ Purple Box Square Salvi;^118^ Olive Diamond Open Liessmann et al.^119^

### 3.3 Residual Entropy Calculation of Different Models

This section analyses how deviations in residual entropy calculations across different EOS affect the accuracy of viscosity predictions. Figure 10 illustrates the relative deviations of entropy scaling parameter *s* ^+^ calculated by the ten cubic EOS from reference EOS^120–123^ for the four fluids studied in Section 3.2. Similar plots for each pure studied in this work are provided in the SI. For CO_2_, the maximum deviation reaches up to 20% for PR-Twu EOS near the critical region, potentially impacting the viscosity description of the supercritical region. For R1234ze­(E) and SO_2_, the deviations follow a consistent sequence across the models, transitioning from positive to negative in the order from SRK EOS to PTV EOS, suggesting systematic behavior in their entropy predictions. In contrast, for MEA, the curve trends differ significantly over temperature, with SRK-MS EOS and PR-MS EOS deviating from the patterns observed in other models. These deviations reveal how thermodynamic property scaling impacts transport property modeling, with *s* ^+^ differences affecting residual viscosity correlations and viscosity predictions. The distinct trends for MEA highlight the need for refined EOS formulations to better capture unique fluid interactions.

**Figure 10:**
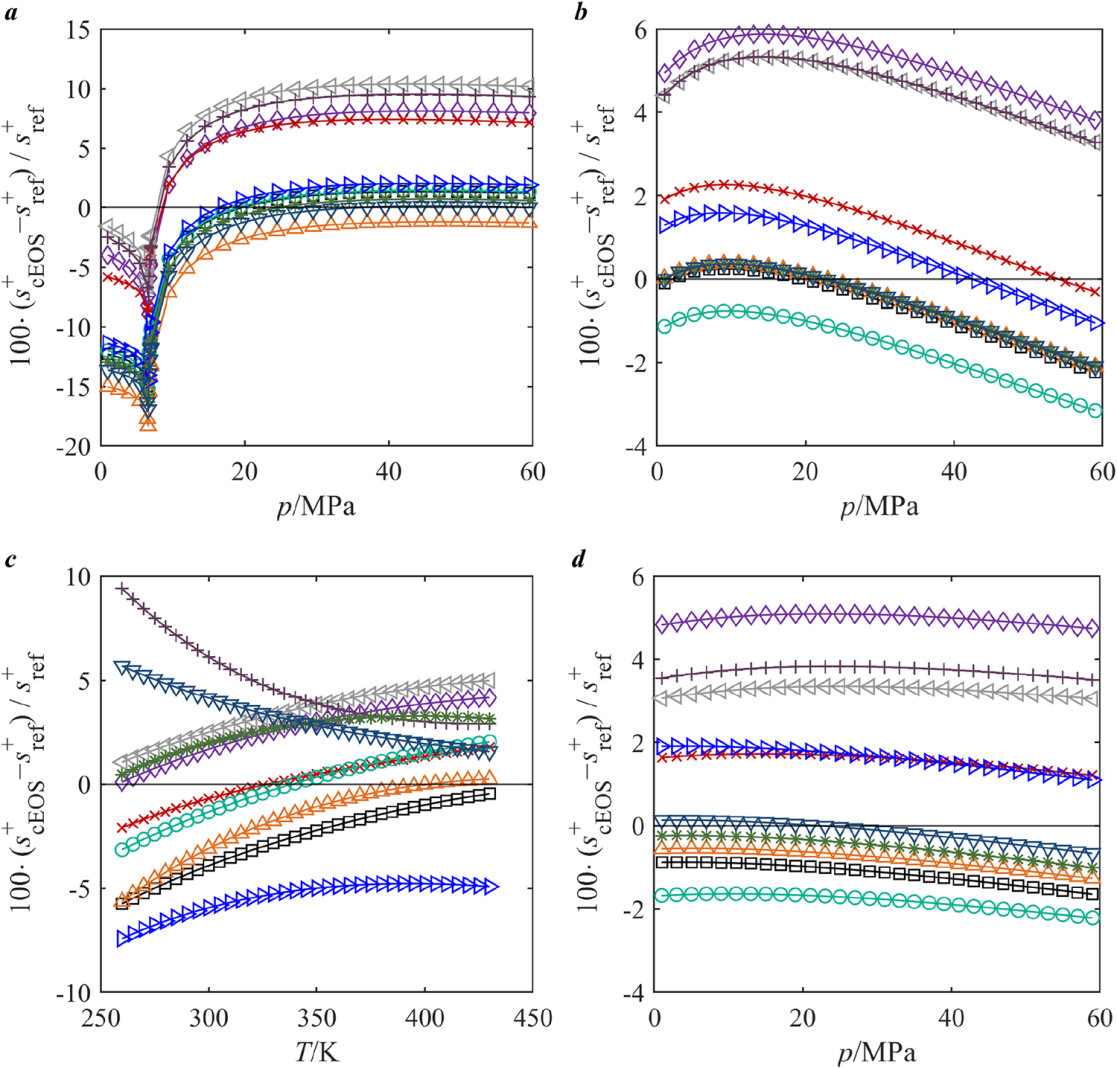
Relative deviations of residual entropy calculations of the ten cubic EOS from reference EOS^120–123^ implemented in REFPROP 10.0.^8^ The selected substances are *a*, CO_2_; *b*, R1234ze­(E); *c*, MEA; and *d*, SO_2_. The plots for CO_2_, R1234ze­(E), and SO_2_ are at 300 K as a function of pressure, while the plot for MEA is at 0.1 MPa as a function of temperature due to the availability of literature data only at 0.1 MPa. Legend for different models (with lines): Black Square PR EOS; Purple Diamond Open SRK EOS; Red Cross YFR EOS; Green Open circle PTV EOS; Orange Triangle Up Open PR-Twu EOS; Blue Triangle Right-Pointing Open PTV-Heyen EOS; Gray Triangle Left-Pointing Open SRK-Coqulet EOS; Purple Plus SRK-MS EOS; Dark Green Eight Spoked Asterisk PR-Coquelet EOS; Blue Triangle Down Open PR-MS EOS.

These findings suggest that inaccuracies in viscosity predictions across different models may indeed stem from deviations in residual entropy calculations. Systematic differences in s^+^ among the cubic EOS constrain the effectiveness of viscosity modeling using the RES approach. Consequently, future α function development could prioritize minimizing s^+^ deviations from the reference EOS to enhance predictive accuracy.

To further assess the potential link between vapor pressure accuracy and residual entropy scaling performance, Figure 11 presents the relative deviations in saturated vapor pressure predicted by the ten cubic EOS combinations, benchmarked against the Span and Wagner reference EOS^120^ for CO_2_. As anticipated, the PR-Twu EOS, featuring component-specific α-function parameters, yields superior vapor pressure predictions compared to those based on generalized α-functions (e.g., PTV-Heyen EOS). This raises the expectation that improved saturation properties might translate into better residual entropy scaling, especially given the importance of vapor pressure in capturing phase behavior. However, a comparison with residual entropy deviations (i.e., Figure 10a) reveals a noticeable disconnect: models with better vapor pressure agreement do not necessarily produce more accurate residual entropy values across the single-phase region.

**Figure 11:**
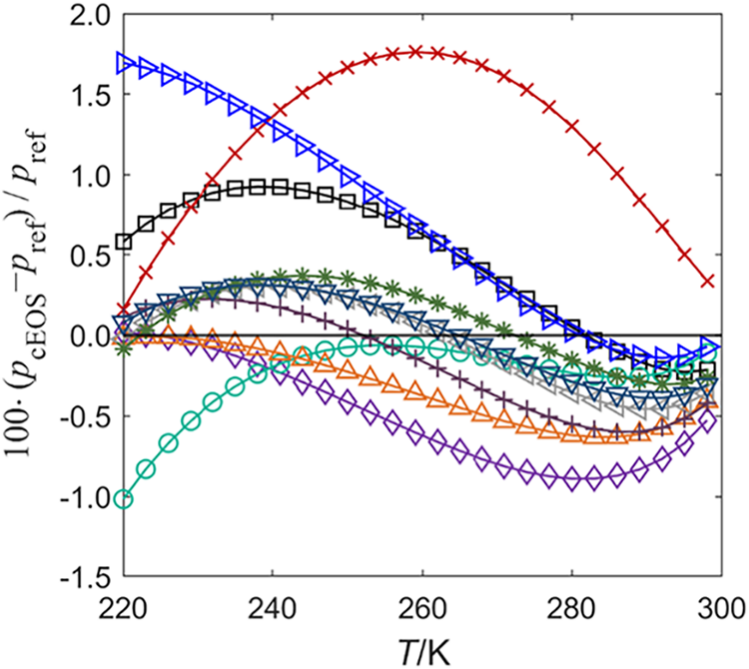
Relative deviations in saturated vapor pressure predictions from ten cubic EOS models compared to the reference EOS^120^ (Span and Wagner) implemented in REFPROP 10.0^8^ for CO_2_. The plot shows deviations as a function of temperature. Legend for different models (with lines): Black Square PR EOS; Purple Diamond Open SRK EOS; Red Cross YFR EOS; Green Open Circle PTV EOS; Orange Triangle Up Open PR-Twu EOS; Blue Triangle Right-Pointing Open PTV-Heyen EOS; Gray Triangle Left-Pointing Open SRK-Coqulet EOS; Purple Plus SRK-MS EOS; Dark Green Eight Spoked Asterisk PR-Coquelet EOS; Blue Triangle Down Open PR-MS EOS.

This decoupling reveals a critical point. Vapor pressure is a one-dimensional function of temperature and is only relevant along the saturation curve, whereas residual entropy is a two-dimensional function of both temperature and pressure, particularly sensitive to the EOS representation in compressed and supercritical states. For example, density plays a key role in shaping residual entropy landscapes and has a greater influence on single-phase transport properties than saturation data alone. Therefore, while vapor pressure accuracy remains a useful diagnostic of α-function quality, it is not sufficient on its own to ensure reliable viscosity scaling. A robust evaluation of EOS performance for entropy-based viscosity models must also consider thermodynamic properties such as density, isobaric heat capacity, and speed of sound. These properties collectively determine the residual entropy predictions in the regions where viscosity data are most critical.

## 4 Conclusions

This study evaluated the performance of cubic EOS with various α functions for viscosity predictions based on RES approach. The analyzed cubic EOS include the PR, SRK, PTV, and YFR EOS. The incorporation of four α functions, Twu, Coquelet, MS, and Heyen rather than the traditional Soave α function demonstrated improvements for specific fluids. For example, the PR-Twu + RES model achieved commendable accuracy for CO_2_, while the PTV-Heyen + RES model exhibited strong performance for R1234ze­(E) and SO_2_. However, increased deviations were observed for 85 and 84 fluids, respectively, for these two models, highlighting the limitations of such modifications in achieving broad predictive accuracy.

The overall AAD from experimental values was 3.1% (PR, SRK, and YFR), 3.0% (PTV), 3.4% (PR-Twu and SRK-Coquelet), 3.2% (PTV-Heyen and SRK-MS), 3.5% (PR-Coquelet), 3.3% (PR-MS), and 2.7% (REF. models). This work reveals the inherent limitations of cubic EOS + RES models in achieving consistent accuracy, possibly stemming from the challenges in residual entropy predictions. Near the critical region, the calculated residual entropy can deviate by up to 20% from the reference EOS. Outside the critical region, in the typical gas and liquid phases, a systematic deviation of approximately 2–5% persists. These discrepancies indicate the necessity for further refinement in modeling approaches to enhance predictive accuracy across a broader range of fluid systems.

Future research will focus on expanding the scope of viscosity modeling in four key areas. First, the analysis will be extended to a broader range of fluids beyond the 124 examined in this study, allowing for a more comprehensive assessment of the generalizability of cubic EOS-based approaches. Second, a more detailed investigation into the influence of different α functions on viscosity predictions across a wider selection of fluids will provide insights into the optimal selection and parametrization of these functions. Third, the impact of volume translation on viscosity modeling will be systematically examined, both within the existing data set of 124 fluids and across an expanded fluid database. Fourth, the study of mixtures will be undertaken to evaluate the applicability and limitations of cubic EOS-based models in predicting viscosity for multicomponent systems. These efforts will enhance the understanding of the applicability and limitations of cubic EOS in viscosity modeling, ultimately contributing to the refinement of thermophysical property predictions.

## Supplementary Material







## Data Availability

The full-resolution dataset, including all individual EOS results and high-quality figures (PR, SRK, PTV, YFR, and modified models), is publicly available at: https://doi.org/10.6084/m9.figshare.28357856.v3.

## Notes

Tables of fluid constants and fluid names with CAS Registry Number (ZIP)
Bar charts illustrating deviations and successful calculation rates for each component; fitted results of η_res_^+^ versus *s* ^+^, viscosity deviation plots for each fluid across different models, and residual entropy comparison plots for each fluid (ZIP)
Details on the extensive tables and figures (PDF)
