# Supplementary figures and images for: Evaluating Cubic Equations of State with Various α Functions for Viscosity Predictions of 124 Industrial Important Fluids Based on Residual Entropy Scaling (part 2 of 4)
Source: ACS Omega. 2025 Jun 27;10(27):29021–36. doi: 10.1021/acsomega.5c01157 (PMC12268422; doi:10.1021/acsomega.5c01157)

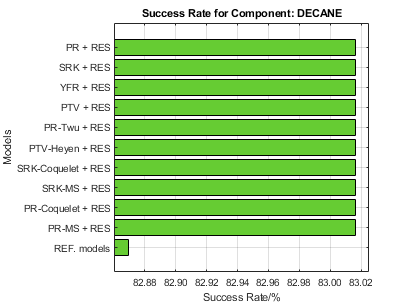

Supplement: Supplementary file 1 [file ao5c01157_si_001.zip › Supporting Information package 1/Figures/Bar_chart_summary/DECANE_SuccessRate.png]

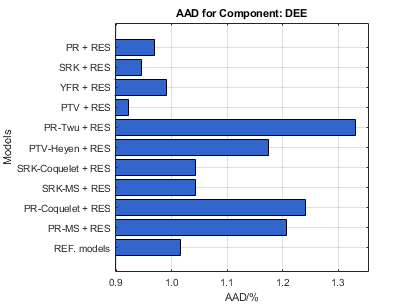

Supplement: Supplementary file 1 [file ao5c01157_si_001.zip › Supporting Information package 1/Figures/Bar_chart_summary/DEE_AAD.png]

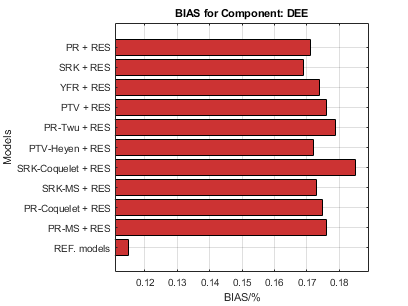

Supplement: Supplementary file 1 [file ao5c01157_si_001.zip › Supporting Information package 1/Figures/Bar_chart_summary/DEE_BIAS.png]

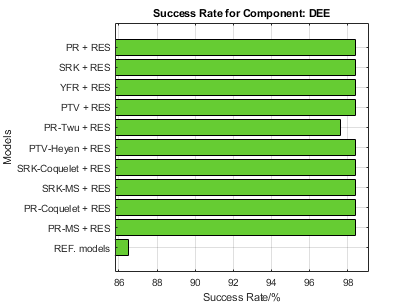

Supplement: Supplementary file 1 [file ao5c01157_si_001.zip › Supporting Information package 1/Figures/Bar_chart_summary/DEE_SuccessRate.png]

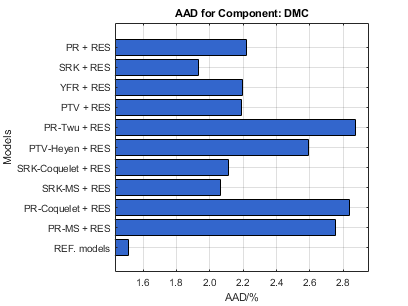

Supplement: Supplementary file 1 [file ao5c01157_si_001.zip › Supporting Information package 1/Figures/Bar_chart_summary/DMC_AAD.png]

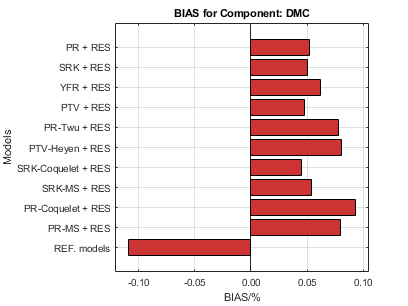

Supplement: Supplementary file 1 [file ao5c01157_si_001.zip › Supporting Information package 1/Figures/Bar_chart_summary/DMC_BIAS.png]

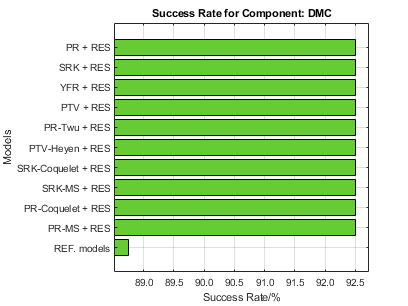

Supplement: Supplementary file 1 [file ao5c01157_si_001.zip › Supporting Information package 1/Figures/Bar_chart_summary/DMC_SuccessRate.png]

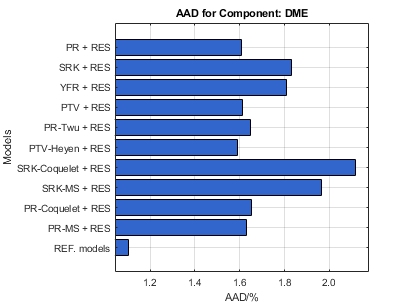

Supplement: Supplementary file 1 [file ao5c01157_si_001.zip › Supporting Information package 1/Figures/Bar_chart_summary/DME_AAD.png]

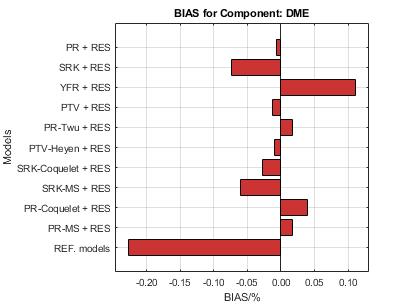

Supplement: Supplementary file 1 [file ao5c01157_si_001.zip › Supporting Information package 1/Figures/Bar_chart_summary/DME_BIAS.png]

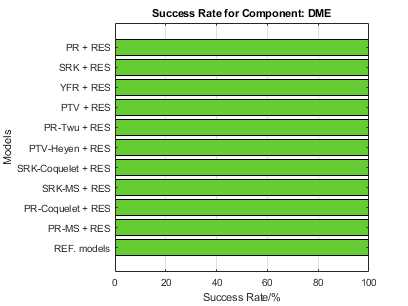

Supplement: Supplementary file 1 [file ao5c01157_si_001.zip › Supporting Information package 1/Figures/Bar_chart_summary/DME_SuccessRate.png]

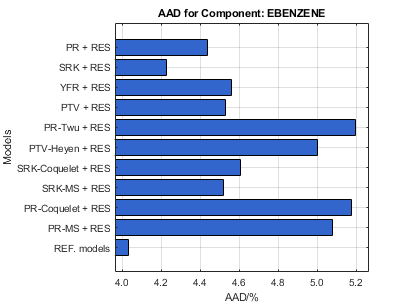

Supplement: Supplementary file 1 [file ao5c01157_si_001.zip › Supporting Information package 1/Figures/Bar_chart_summary/EBENZENE_AAD.png]

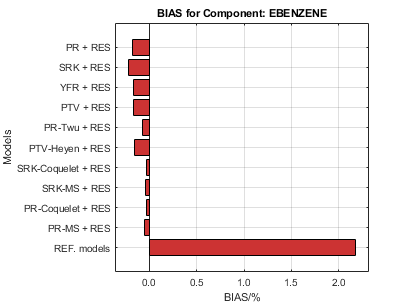

Supplement: Supplementary file 1 [file ao5c01157_si_001.zip › Supporting Information package 1/Figures/Bar_chart_summary/EBENZENE_BIAS.png]

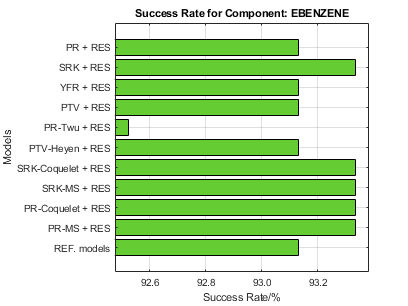

Supplement: Supplementary file 1 [file ao5c01157_si_001.zip › Supporting Information package 1/Figures/Bar_chart_summary/EBENZENE_SuccessRate.png]

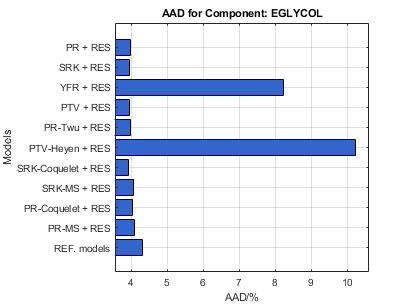

Supplement: Supplementary file 1 [file ao5c01157_si_001.zip › Supporting Information package 1/Figures/Bar_chart_summary/EGLYCOL_AAD.png]

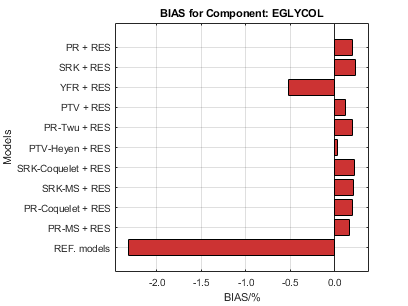

Supplement: Supplementary file 1 [file ao5c01157_si_001.zip › Supporting Information package 1/Figures/Bar_chart_summary/EGLYCOL_BIAS.png]

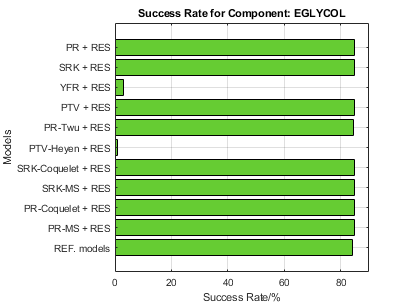

Supplement: Supplementary file 1 [file ao5c01157_si_001.zip › Supporting Information package 1/Figures/Bar_chart_summary/EGLYCOL_SuccessRate.png]

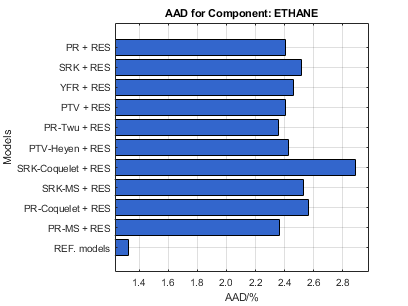

Supplement: Supplementary file 1 [file ao5c01157_si_001.zip › Supporting Information package 1/Figures/Bar_chart_summary/ETHANE_AAD.png]

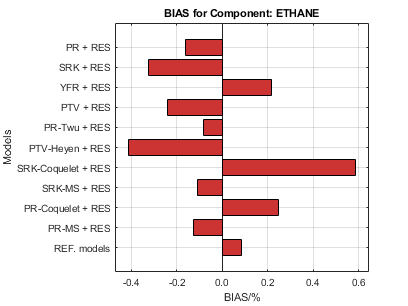

Supplement: Supplementary file 1 [file ao5c01157_si_001.zip › Supporting Information package 1/Figures/Bar_chart_summary/ETHANE_BIAS.png]

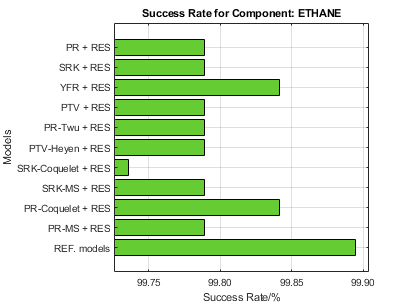

Supplement: Supplementary file 1 [file ao5c01157_si_001.zip › Supporting Information package 1/Figures/Bar_chart_summary/ETHANE_SuccessRate.png]

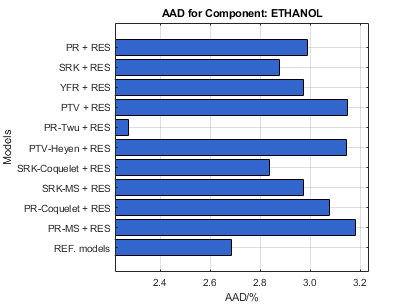

Supplement: Supplementary file 1 [file ao5c01157_si_001.zip › Supporting Information package 1/Figures/Bar_chart_summary/ETHANOL_AAD.png]

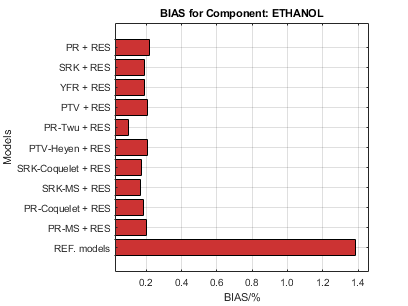

Supplement: Supplementary file 1 [file ao5c01157_si_001.zip › Supporting Information package 1/Figures/Bar_chart_summary/ETHANOL_BIAS.png]

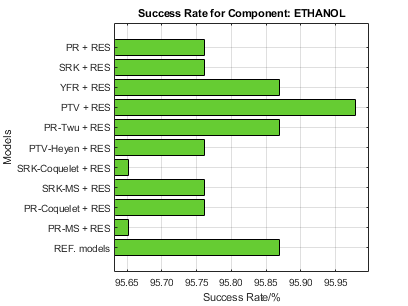

Supplement: Supplementary file 1 [file ao5c01157_si_001.zip › Supporting Information package 1/Figures/Bar_chart_summary/ETHANOL_SuccessRate.png]

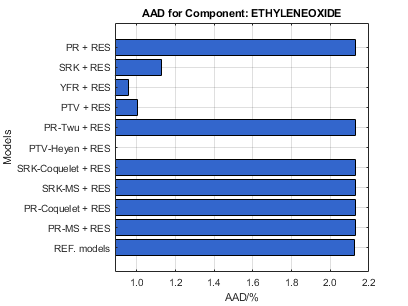

Supplement: Supplementary file 1 [file ao5c01157_si_001.zip › Supporting Information package 1/Figures/Bar_chart_summary/ETHYLENEOXIDE_AAD.png]

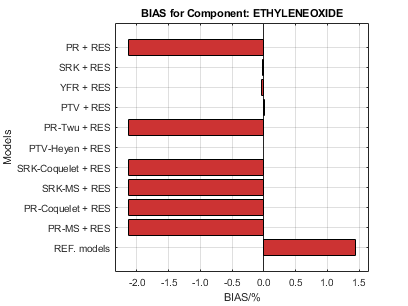

Supplement: Supplementary file 1 [file ao5c01157_si_001.zip › Supporting Information package 1/Figures/Bar_chart_summary/ETHYLENEOXIDE_BIAS.png]

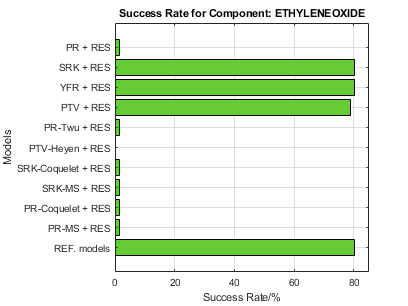

Supplement: Supplementary file 1 [file ao5c01157_si_001.zip › Supporting Information package 1/Figures/Bar_chart_summary/ETHYLENEOXIDE_SuccessRate.png]

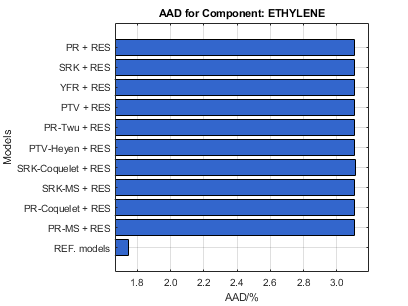

Supplement: Supplementary file 1 [file ao5c01157_si_001.zip › Supporting Information package 1/Figures/Bar_chart_summary/ETHYLENE_AAD.png]

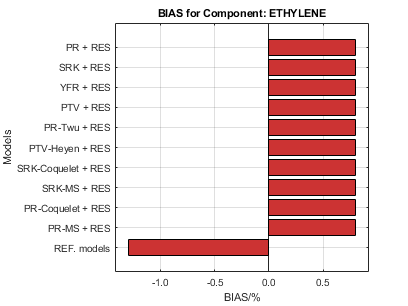

Supplement: Supplementary file 1 [file ao5c01157_si_001.zip › Supporting Information package 1/Figures/Bar_chart_summary/ETHYLENE_BIAS.png]

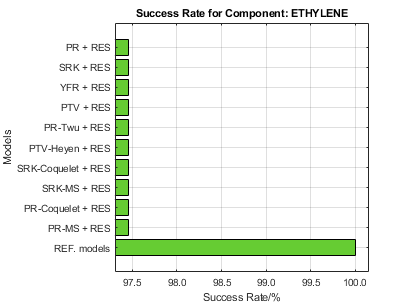

Supplement: Supplementary file 1 [file ao5c01157_si_001.zip › Supporting Information package 1/Figures/Bar_chart_summary/ETHYLENE_SuccessRate.png]

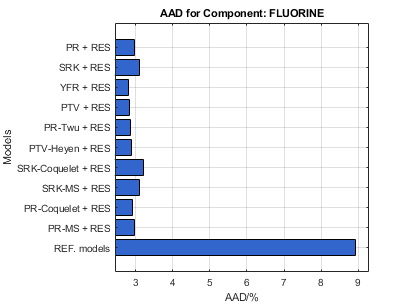

Supplement: Supplementary file 1 [file ao5c01157_si_001.zip › Supporting Information package 1/Figures/Bar_chart_summary/FLUORINE_AAD.png]

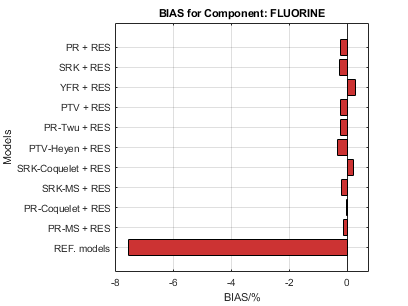

Supplement: Supplementary file 1 [file ao5c01157_si_001.zip › Supporting Information package 1/Figures/Bar_chart_summary/FLUORINE_BIAS.png]

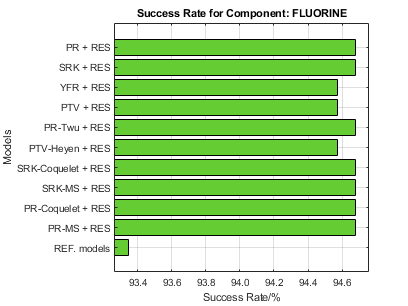

Supplement: Supplementary file 1 [file ao5c01157_si_001.zip › Supporting Information package 1/Figures/Bar_chart_summary/FLUORINE_SuccessRate.png]

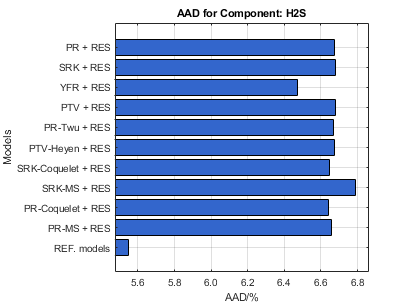

Supplement: Supplementary file 1 [file ao5c01157_si_001.zip › Supporting Information package 1/Figures/Bar_chart_summary/H2S_AAD.png]

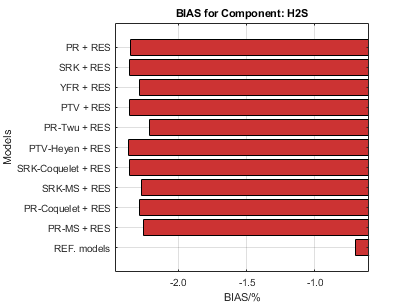

Supplement: Supplementary file 1 [file ao5c01157_si_001.zip › Supporting Information package 1/Figures/Bar_chart_summary/H2S_BIAS.png]

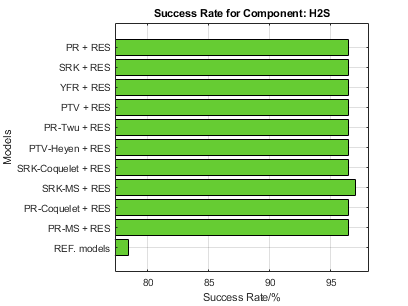

Supplement: Supplementary file 1 [file ao5c01157_si_001.zip › Supporting Information package 1/Figures/Bar_chart_summary/H2S_SuccessRate.png]

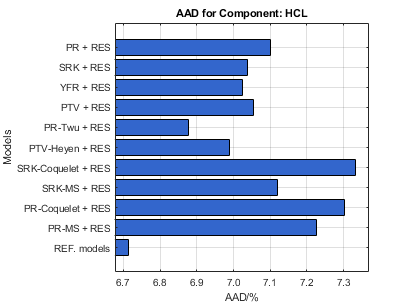

Supplement: Supplementary file 1 [file ao5c01157_si_001.zip › Supporting Information package 1/Figures/Bar_chart_summary/HCL_AAD.png]

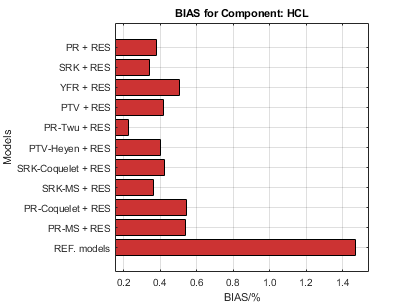

Supplement: Supplementary file 1 [file ao5c01157_si_001.zip › Supporting Information package 1/Figures/Bar_chart_summary/HCL_BIAS.png]

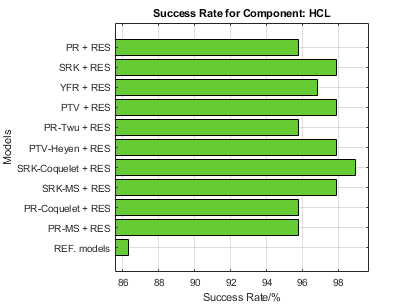

Supplement: Supplementary file 1 [file ao5c01157_si_001.zip › Supporting Information package 1/Figures/Bar_chart_summary/HCL_SuccessRate.png]

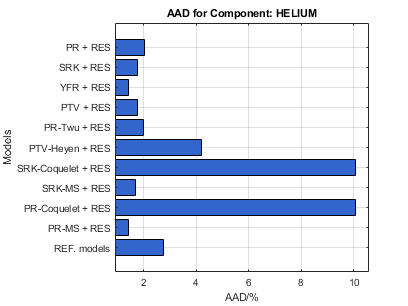

Supplement: Supplementary file 1 [file ao5c01157_si_001.zip › Supporting Information package 1/Figures/Bar_chart_summary/HELIUM_AAD.png]

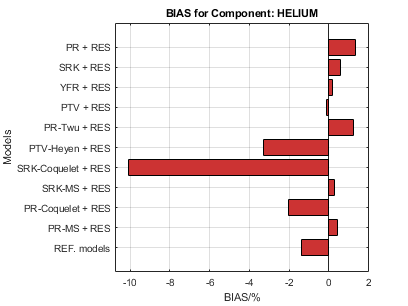

Supplement: Supplementary file 1 [file ao5c01157_si_001.zip › Supporting Information package 1/Figures/Bar_chart_summary/HELIUM_BIAS.png]

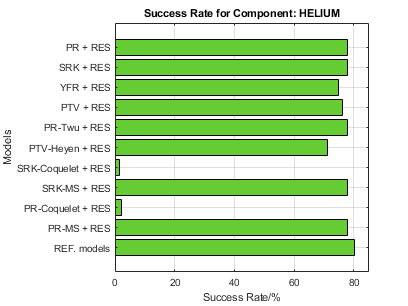

Supplement: Supplementary file 1 [file ao5c01157_si_001.zip › Supporting Information package 1/Figures/Bar_chart_summary/HELIUM_SuccessRate.png]

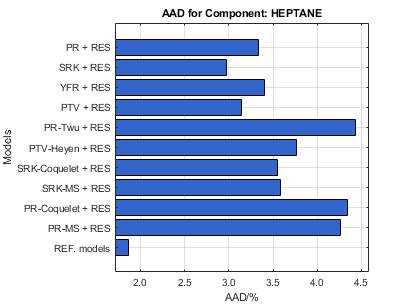

Supplement: Supplementary file 1 [file ao5c01157_si_001.zip › Supporting Information package 1/Figures/Bar_chart_summary/HEPTANE_AAD.png]

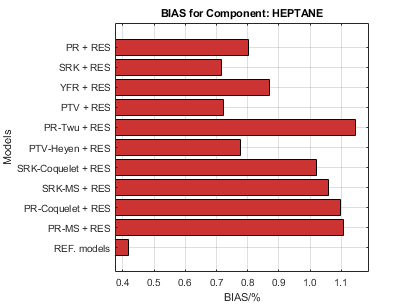

Supplement: Supplementary file 1 [file ao5c01157_si_001.zip › Supporting Information package 1/Figures/Bar_chart_summary/HEPTANE_BIAS.png]

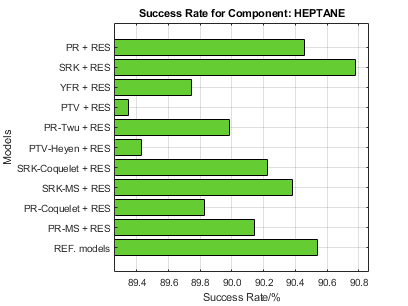

Supplement: Supplementary file 1 [file ao5c01157_si_001.zip › Supporting Information package 1/Figures/Bar_chart_summary/HEPTANE_SuccessRate.png]

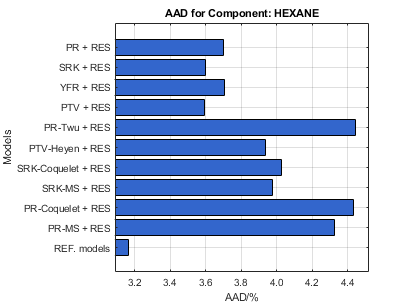

Supplement: Supplementary file 1 [file ao5c01157_si_001.zip › Supporting Information package 1/Figures/Bar_chart_summary/HEXANE_AAD.png]

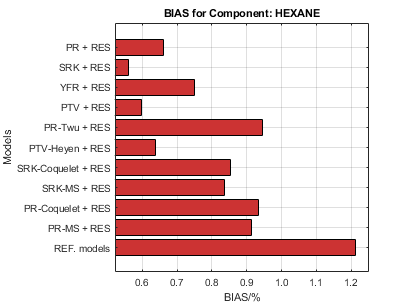

Supplement: Supplementary file 1 [file ao5c01157_si_001.zip › Supporting Information package 1/Figures/Bar_chart_summary/HEXANE_BIAS.png]

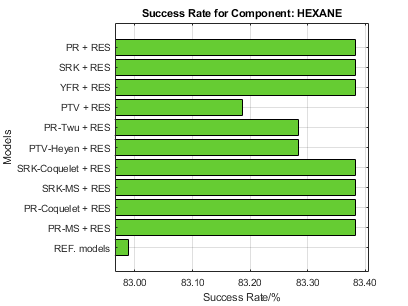

Supplement: Supplementary file 1 [file ao5c01157_si_001.zip › Supporting Information package 1/Figures/Bar_chart_summary/HEXANE_SuccessRate.png]

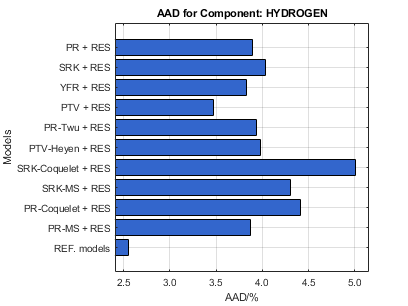

Supplement: Supplementary file 1 [file ao5c01157_si_001.zip › Supporting Information package 1/Figures/Bar_chart_summary/HYDROGEN_AAD.png]

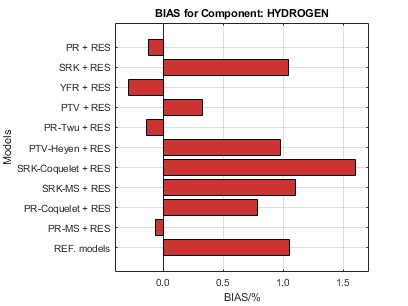

Supplement: Supplementary file 1 [file ao5c01157_si_001.zip › Supporting Information package 1/Figures/Bar_chart_summary/HYDROGEN_BIAS.png]

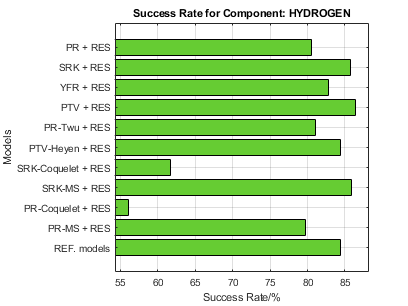

Supplement: Supplementary file 1 [file ao5c01157_si_001.zip › Supporting Information package 1/Figures/Bar_chart_summary/HYDROGEN_SuccessRate.png]

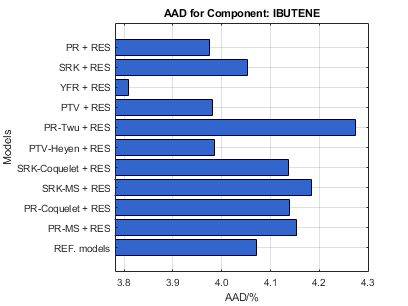

Supplement: Supplementary file 1 [file ao5c01157_si_001.zip › Supporting Information package 1/Figures/Bar_chart_summary/IBUTENE_AAD.png]

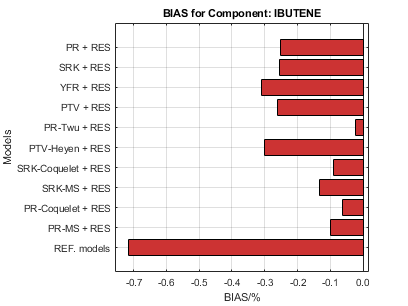

Supplement: Supplementary file 1 [file ao5c01157_si_001.zip › Supporting Information package 1/Figures/Bar_chart_summary/IBUTENE_BIAS.png]

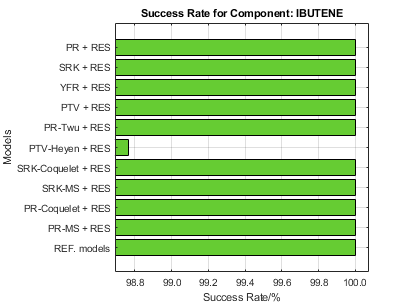

Supplement: Supplementary file 1 [file ao5c01157_si_001.zip › Supporting Information package 1/Figures/Bar_chart_summary/IBUTENE_SuccessRate.png]

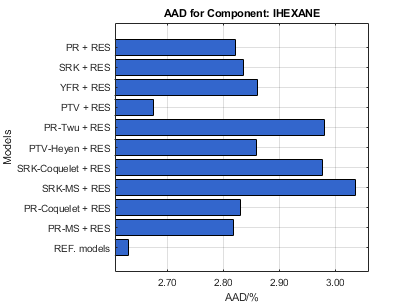

Supplement: Supplementary file 1 [file ao5c01157_si_001.zip › Supporting Information package 1/Figures/Bar_chart_summary/IHEXANE_AAD.png]

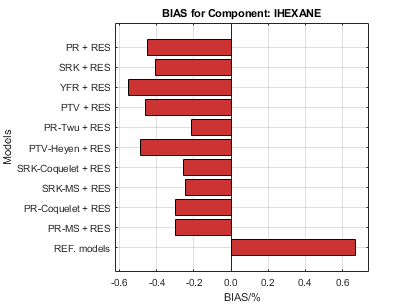

Supplement: Supplementary file 1 [file ao5c01157_si_001.zip › Supporting Information package 1/Figures/Bar_chart_summary/IHEXANE_BIAS.png]

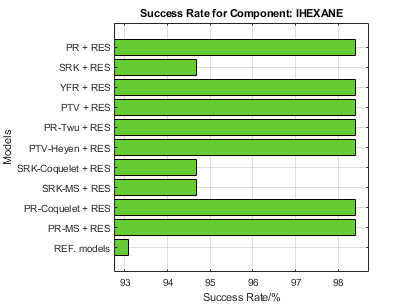

Supplement: Supplementary file 1 [file ao5c01157_si_001.zip › Supporting Information package 1/Figures/Bar_chart_summary/IHEXANE_SuccessRate.png]

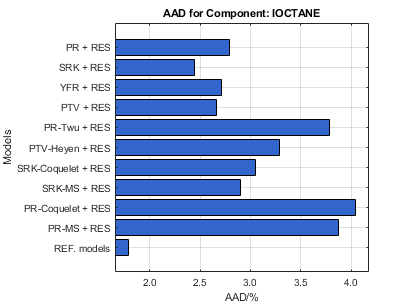

Supplement: Supplementary file 1 [file ao5c01157_si_001.zip › Supporting Information package 1/Figures/Bar_chart_summary/IOCTANE_AAD.png]

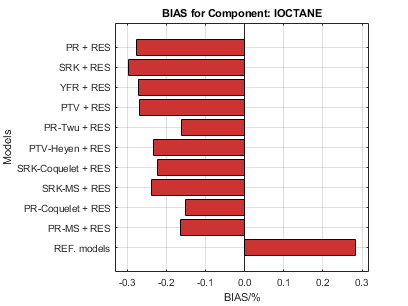

Supplement: Supplementary file 1 [file ao5c01157_si_001.zip › Supporting Information package 1/Figures/Bar_chart_summary/IOCTANE_BIAS.png]

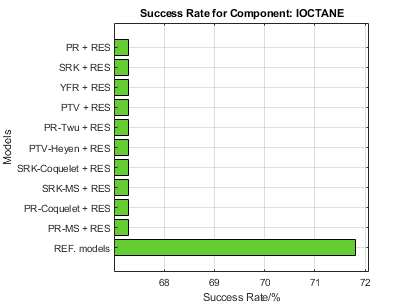

Supplement: Supplementary file 1 [file ao5c01157_si_001.zip › Supporting Information package 1/Figures/Bar_chart_summary/IOCTANE_SuccessRate.png]

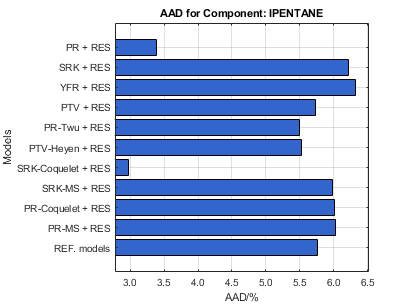

Supplement: Supplementary file 1 [file ao5c01157_si_001.zip › Supporting Information package 1/Figures/Bar_chart_summary/IPENTANE_AAD.png]

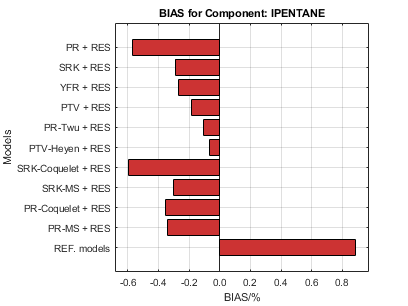

Supplement: Supplementary file 1 [file ao5c01157_si_001.zip › Supporting Information package 1/Figures/Bar_chart_summary/IPENTANE_BIAS.png]

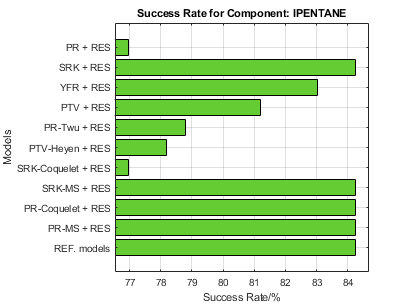

Supplement: Supplementary file 1 [file ao5c01157_si_001.zip › Supporting Information package 1/Figures/Bar_chart_summary/IPENTANE_SuccessRate.png]

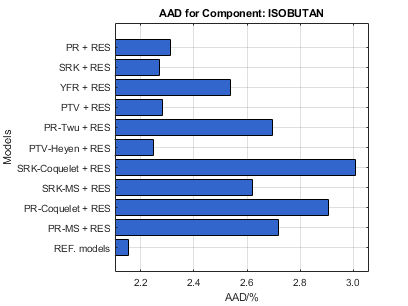

Supplement: Supplementary file 1 [file ao5c01157_si_001.zip › Supporting Information package 1/Figures/Bar_chart_summary/ISOBUTAN_AAD.png]

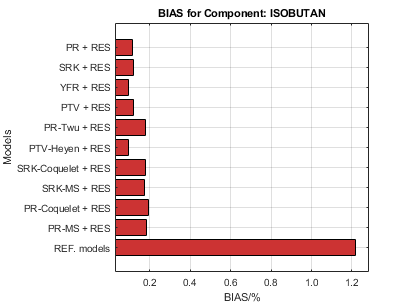

Supplement: Supplementary file 1 [file ao5c01157_si_001.zip › Supporting Information package 1/Figures/Bar_chart_summary/ISOBUTAN_BIAS.png]

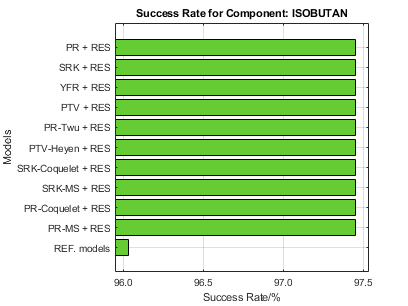

Supplement: Supplementary file 1 [file ao5c01157_si_001.zip › Supporting Information package 1/Figures/Bar_chart_summary/ISOBUTAN_SuccessRate.png]

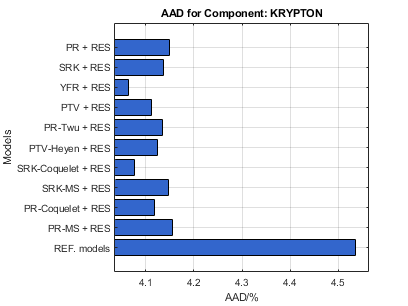

Supplement: Supplementary file 1 [file ao5c01157_si_001.zip › Supporting Information package 1/Figures/Bar_chart_summary/KRYPTON_AAD.png]

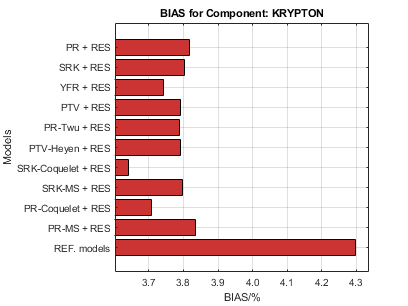

Supplement: Supplementary file 1 [file ao5c01157_si_001.zip › Supporting Information package 1/Figures/Bar_chart_summary/KRYPTON_BIAS.png]

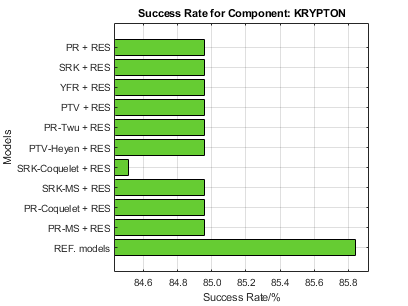

Supplement: Supplementary file 1 [file ao5c01157_si_001.zip › Supporting Information package 1/Figures/Bar_chart_summary/KRYPTON_SuccessRate.png]

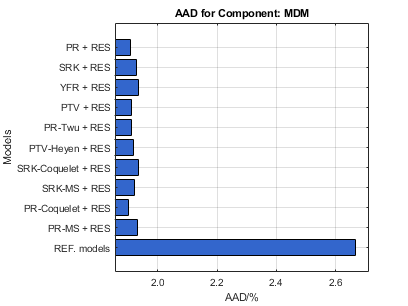

Supplement: Supplementary file 1 [file ao5c01157_si_001.zip › Supporting Information package 1/Figures/Bar_chart_summary/MDM_AAD.png]

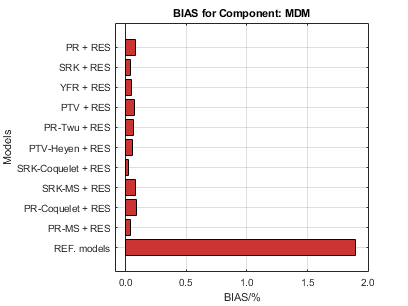

Supplement: Supplementary file 1 [file ao5c01157_si_001.zip › Supporting Information package 1/Figures/Bar_chart_summary/MDM_BIAS.png]

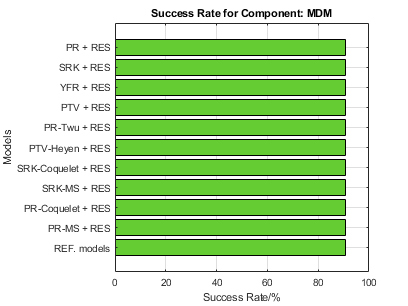

Supplement: Supplementary file 1 [file ao5c01157_si_001.zip › Supporting Information package 1/Figures/Bar_chart_summary/MDM_SuccessRate.png]

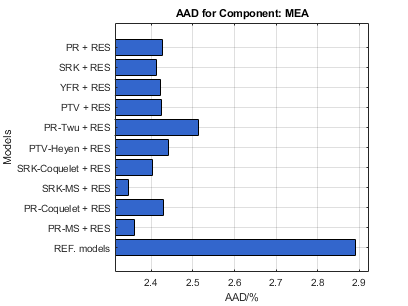

Supplement: Supplementary file 1 [file ao5c01157_si_001.zip › Supporting Information package 1/Figures/Bar_chart_summary/MEA_AAD.png]

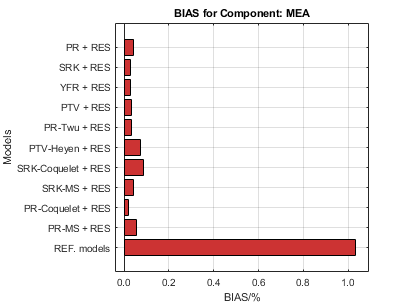

Supplement: Supplementary file 1 [file ao5c01157_si_001.zip › Supporting Information package 1/Figures/Bar_chart_summary/MEA_BIAS.png]

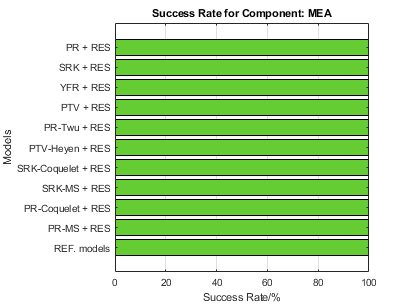

Supplement: Supplementary file 1 [file ao5c01157_si_001.zip › Supporting Information package 1/Figures/Bar_chart_summary/MEA_SuccessRate.png]

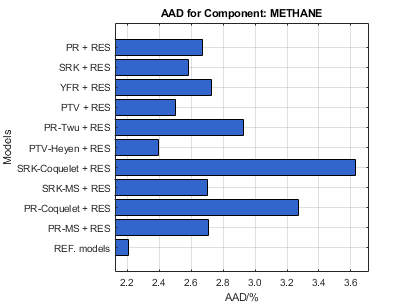

Supplement: Supplementary file 1 [file ao5c01157_si_001.zip › Supporting Information package 1/Figures/Bar_chart_summary/METHANE_AAD.png]

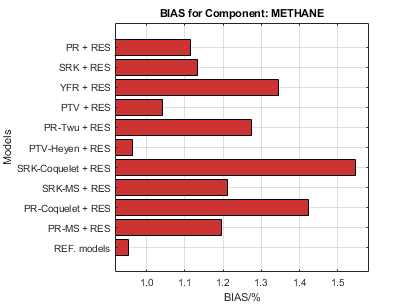

Supplement: Supplementary file 1 [file ao5c01157_si_001.zip › Supporting Information package 1/Figures/Bar_chart_summary/METHANE_BIAS.png]

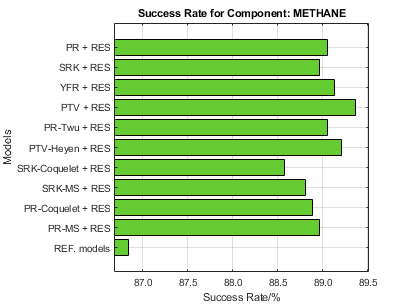

Supplement: Supplementary file 1 [file ao5c01157_si_001.zip › Supporting Information package 1/Figures/Bar_chart_summary/METHANE_SuccessRate.png]

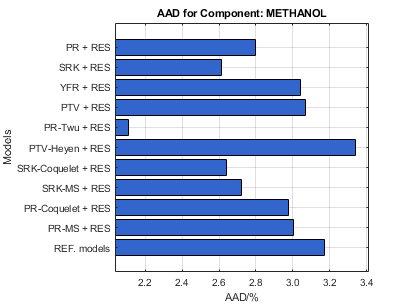

Supplement: Supplementary file 1 [file ao5c01157_si_001.zip › Supporting Information package 1/Figures/Bar_chart_summary/METHANOL_AAD.png]

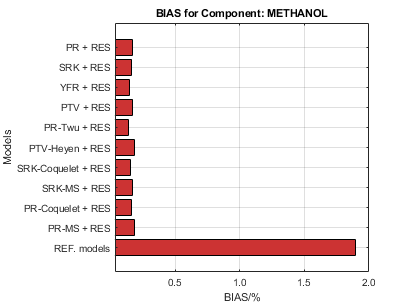

Supplement: Supplementary file 1 [file ao5c01157_si_001.zip › Supporting Information package 1/Figures/Bar_chart_summary/METHANOL_BIAS.png]

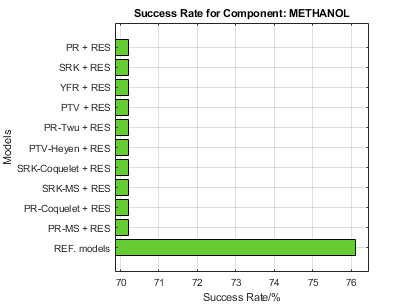

Supplement: Supplementary file 1 [file ao5c01157_si_001.zip › Supporting Information package 1/Figures/Bar_chart_summary/METHANOL_SuccessRate.png]

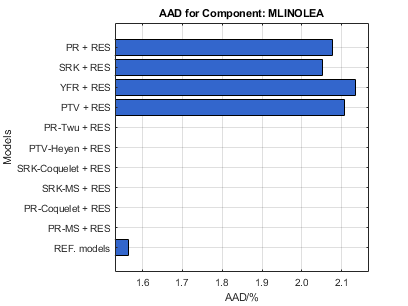

Supplement: Supplementary file 1 [file ao5c01157_si_001.zip › Supporting Information package 1/Figures/Bar_chart_summary/MLINOLEA_AAD.png]

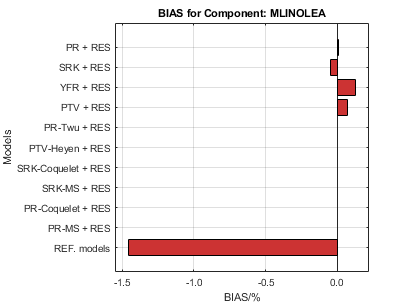

Supplement: Supplementary file 1 [file ao5c01157_si_001.zip › Supporting Information package 1/Figures/Bar_chart_summary/MLINOLEA_BIAS.png]

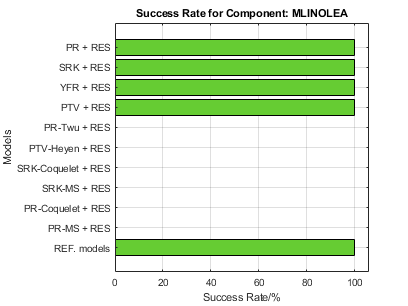

Supplement: Supplementary file 1 [file ao5c01157_si_001.zip › Supporting Information package 1/Figures/Bar_chart_summary/MLINOLEA_SuccessRate.png]

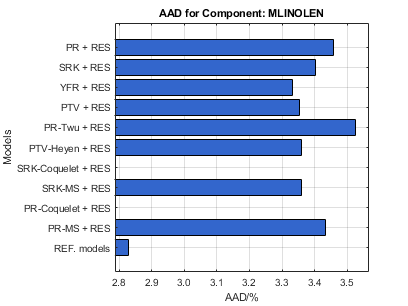

Supplement: Supplementary file 1 [file ao5c01157_si_001.zip › Supporting Information package 1/Figures/Bar_chart_summary/MLINOLEN_AAD.png]

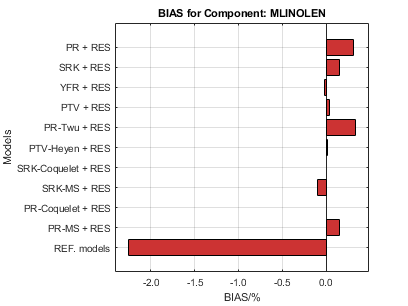

Supplement: Supplementary file 1 [file ao5c01157_si_001.zip › Supporting Information package 1/Figures/Bar_chart_summary/MLINOLEN_BIAS.png]

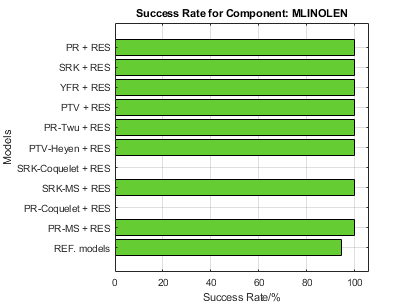

Supplement: Supplementary file 1 [file ao5c01157_si_001.zip › Supporting Information package 1/Figures/Bar_chart_summary/MLINOLEN_SuccessRate.png]

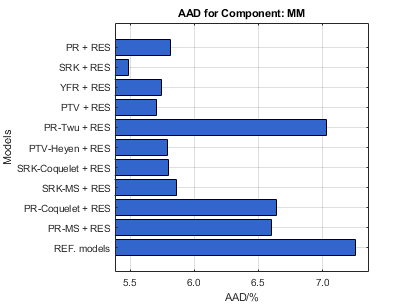

Supplement: Supplementary file 1 [file ao5c01157_si_001.zip › Supporting Information package 1/Figures/Bar_chart_summary/MM_AAD.png]

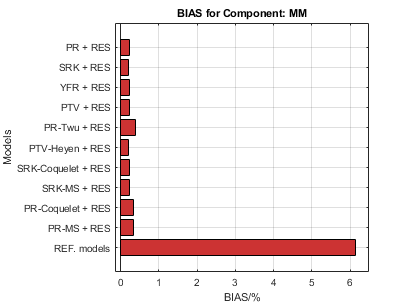

Supplement: Supplementary file 1 [file ao5c01157_si_001.zip › Supporting Information package 1/Figures/Bar_chart_summary/MM_BIAS.png]

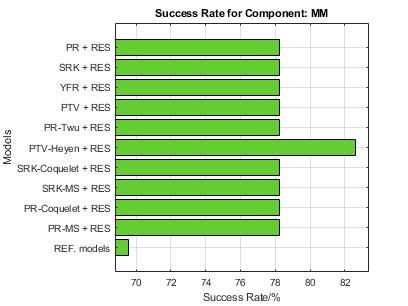

Supplement: Supplementary file 1 [file ao5c01157_si_001.zip › Supporting Information package 1/Figures/Bar_chart_summary/MM_SuccessRate.png]

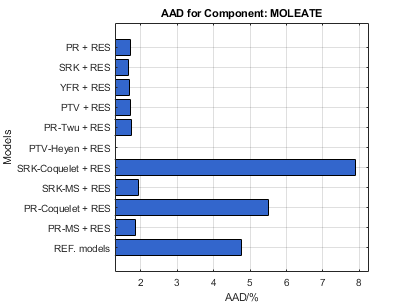

Supplement: Supplementary file 1 [file ao5c01157_si_001.zip › Supporting Information package 1/Figures/Bar_chart_summary/MOLEATE_AAD.png]

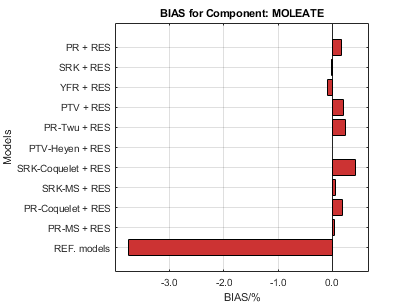

Supplement: Supplementary file 1 [file ao5c01157_si_001.zip › Supporting Information package 1/Figures/Bar_chart_summary/MOLEATE_BIAS.png]

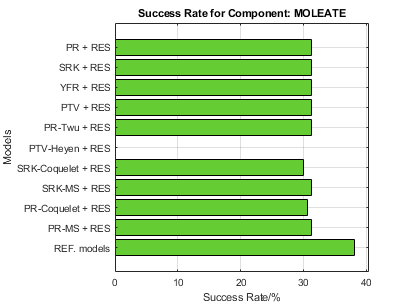

Supplement: Supplementary file 1 [file ao5c01157_si_001.zip › Supporting Information package 1/Figures/Bar_chart_summary/MOLEATE_SuccessRate.png]

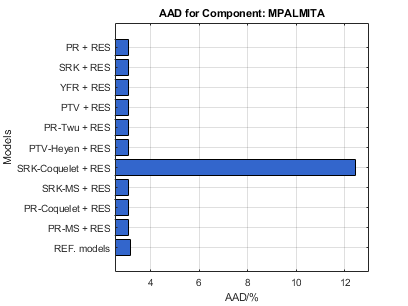

Supplement: Supplementary file 1 [file ao5c01157_si_001.zip › Supporting Information package 1/Figures/Bar_chart_summary/MPALMITA_AAD.png]

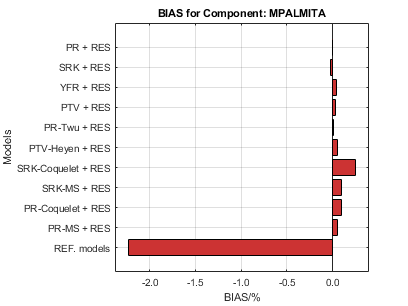

Supplement: Supplementary file 1 [file ao5c01157_si_001.zip › Supporting Information package 1/Figures/Bar_chart_summary/MPALMITA_BIAS.png]

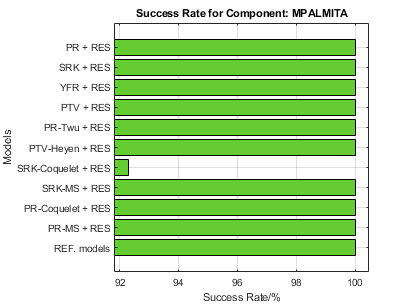

Supplement: Supplementary file 1 [file ao5c01157_si_001.zip › Supporting Information package 1/Figures/Bar_chart_summary/MPALMITA_SuccessRate.png]

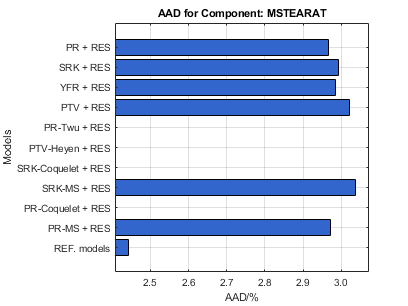

Supplement: Supplementary file 1 [file ao5c01157_si_001.zip › Supporting Information package 1/Figures/Bar_chart_summary/MSTEARAT_AAD.png]

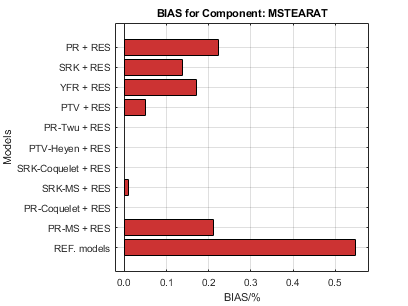

Supplement: Supplementary file 1 [file ao5c01157_si_001.zip › Supporting Information package 1/Figures/Bar_chart_summary/MSTEARAT_BIAS.png]

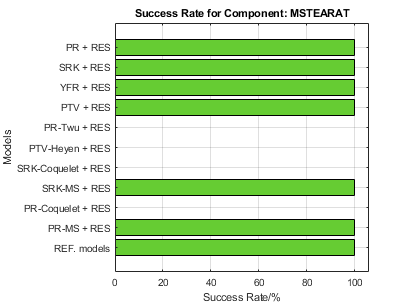

Supplement: Supplementary file 1 [file ao5c01157_si_001.zip › Supporting Information package 1/Figures/Bar_chart_summary/MSTEARAT_SuccessRate.png]

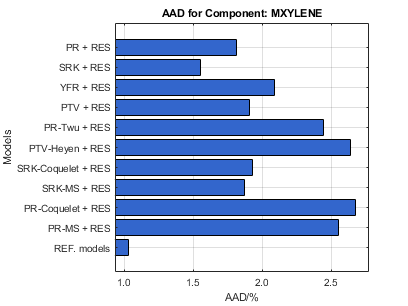

Supplement: Supplementary file 1 [file ao5c01157_si_001.zip › Supporting Information package 1/Figures/Bar_chart_summary/MXYLENE_AAD.png]

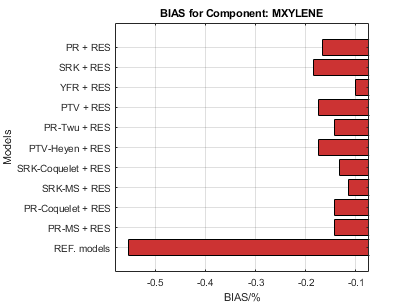

Supplement: Supplementary file 1 [file ao5c01157_si_001.zip › Supporting Information package 1/Figures/Bar_chart_summary/MXYLENE_BIAS.png]

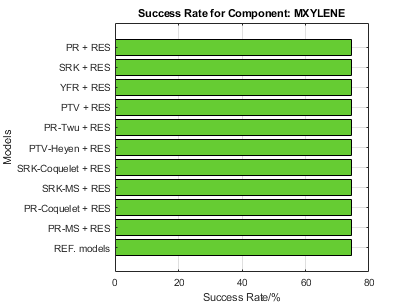

Supplement: Supplementary file 1 [file ao5c01157_si_001.zip › Supporting Information package 1/Figures/Bar_chart_summary/MXYLENE_SuccessRate.png]
