# Supplementary figures and images for: Evaluating Cubic Equations of State with Various α Functions for Viscosity Predictions of 124 Industrial Important Fluids Based on Residual Entropy Scaling (part 1 of 4)
Source: ACS Omega. 2025 Jun 27;10(27):29021–36. doi: 10.1021/acsomega.5c01157 (PMC12268422; doi:10.1021/acsomega.5c01157)

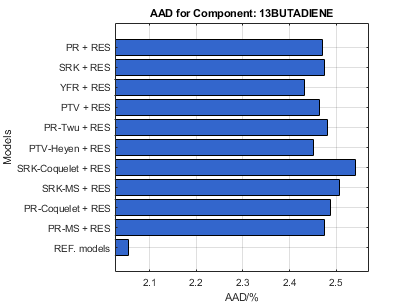

Supplement: Supplementary file 1 [file ao5c01157_si_001.zip › Supporting Information package 1/Figures/Bar_chart_summary/13BUTADIENE_AAD.png]

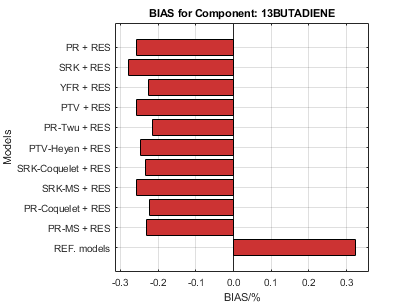

Supplement: Supplementary file 1 [file ao5c01157_si_001.zip › Supporting Information package 1/Figures/Bar_chart_summary/13BUTADIENE_BIAS.png]

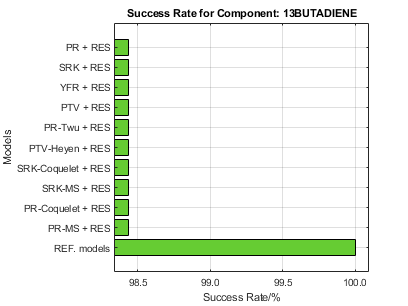

Supplement: Supplementary file 1 [file ao5c01157_si_001.zip › Supporting Information package 1/Figures/Bar_chart_summary/13BUTADIENE_SuccessRate.png]

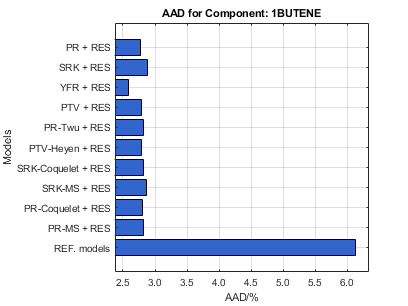

Supplement: Supplementary file 1 [file ao5c01157_si_001.zip › Supporting Information package 1/Figures/Bar_chart_summary/1BUTENE_AAD.png]

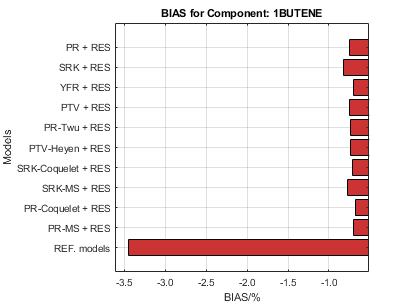

Supplement: Supplementary file 1 [file ao5c01157_si_001.zip › Supporting Information package 1/Figures/Bar_chart_summary/1BUTENE_BIAS.png]

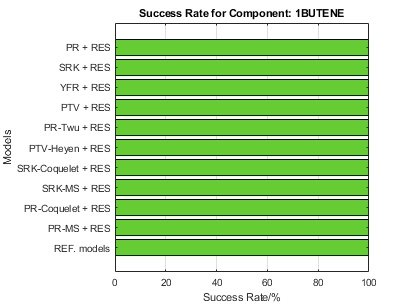

Supplement: Supplementary file 1 [file ao5c01157_si_001.zip › Supporting Information package 1/Figures/Bar_chart_summary/1BUTENE_SuccessRate.png]

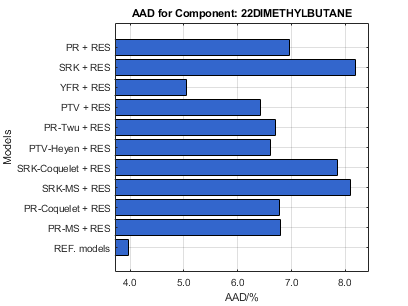

Supplement: Supplementary file 1 [file ao5c01157_si_001.zip › Supporting Information package 1/Figures/Bar_chart_summary/22DIMETHYLBUTANE_AAD.png]

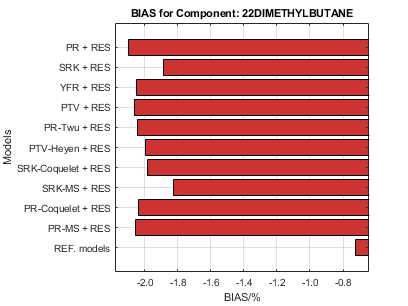

Supplement: Supplementary file 1 [file ao5c01157_si_001.zip › Supporting Information package 1/Figures/Bar_chart_summary/22DIMETHYLBUTANE_BIAS.png]

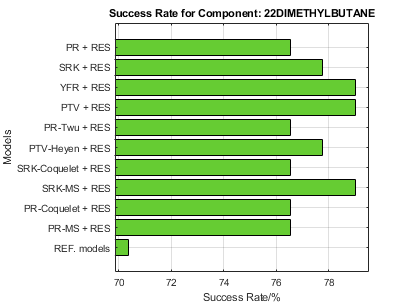

Supplement: Supplementary file 1 [file ao5c01157_si_001.zip › Supporting Information package 1/Figures/Bar_chart_summary/22DIMETHYLBUTANE_SuccessRate.png]

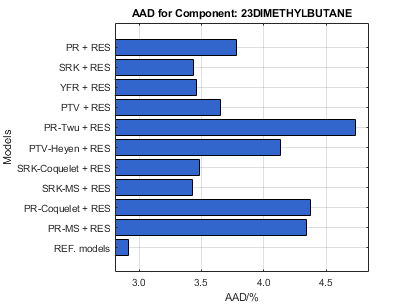

Supplement: Supplementary file 1 [file ao5c01157_si_001.zip › Supporting Information package 1/Figures/Bar_chart_summary/23DIMETHYLBUTANE_AAD.png]

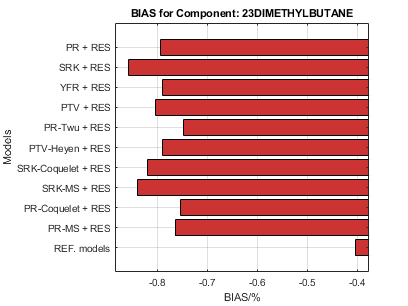

Supplement: Supplementary file 1 [file ao5c01157_si_001.zip › Supporting Information package 1/Figures/Bar_chart_summary/23DIMETHYLBUTANE_BIAS.png]

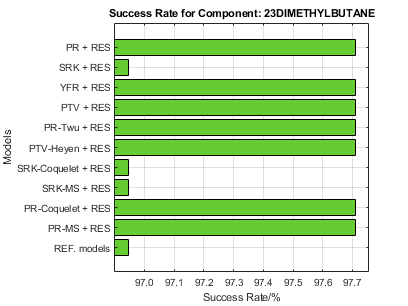

Supplement: Supplementary file 1 [file ao5c01157_si_001.zip › Supporting Information package 1/Figures/Bar_chart_summary/23DIMETHYLBUTANE_SuccessRate.png]

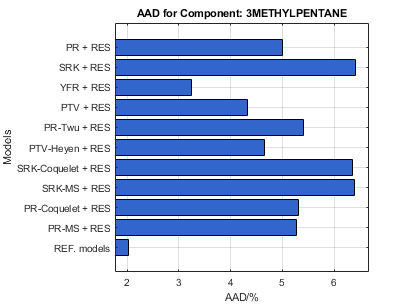

Supplement: Supplementary file 1 [file ao5c01157_si_001.zip › Supporting Information package 1/Figures/Bar_chart_summary/3METHYLPENTANE_AAD.png]

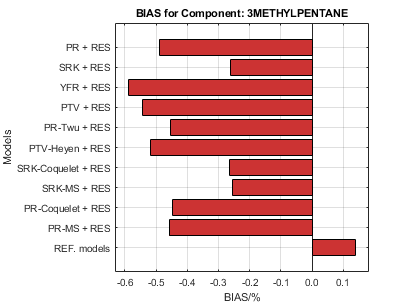

Supplement: Supplementary file 1 [file ao5c01157_si_001.zip › Supporting Information package 1/Figures/Bar_chart_summary/3METHYLPENTANE_BIAS.png]

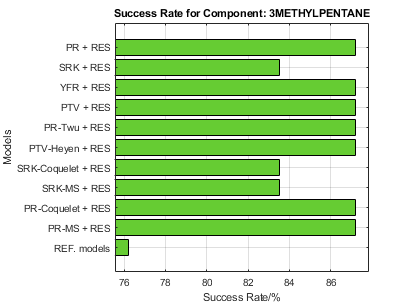

Supplement: Supplementary file 1 [file ao5c01157_si_001.zip › Supporting Information package 1/Figures/Bar_chart_summary/3METHYLPENTANE_SuccessRate.png]

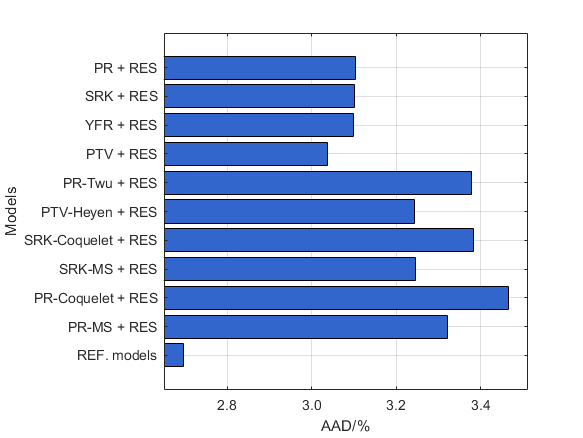

Supplement: Supplementary file 1 [file ao5c01157_si_001.zip › Supporting Information package 1/Figures/Bar_chart_summary/AAD/overall_AAD.png]

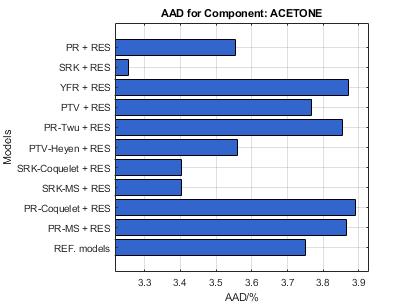

Supplement: Supplementary file 1 [file ao5c01157_si_001.zip › Supporting Information package 1/Figures/Bar_chart_summary/ACETONE_AAD.png]

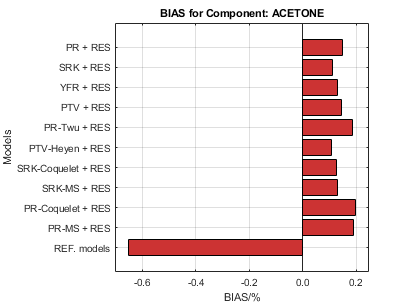

Supplement: Supplementary file 1 [file ao5c01157_si_001.zip › Supporting Information package 1/Figures/Bar_chart_summary/ACETONE_BIAS.png]

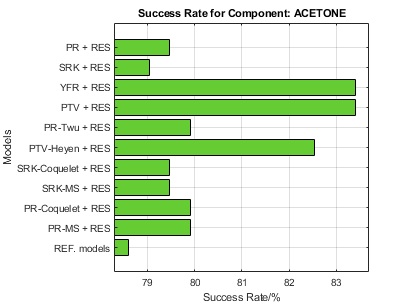

Supplement: Supplementary file 1 [file ao5c01157_si_001.zip › Supporting Information package 1/Figures/Bar_chart_summary/ACETONE_SuccessRate.png]

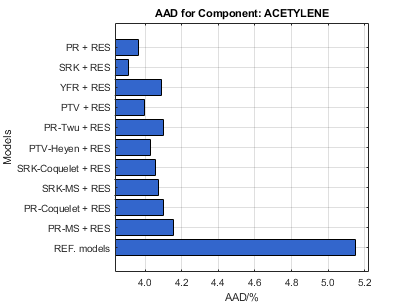

Supplement: Supplementary file 1 [file ao5c01157_si_001.zip › Supporting Information package 1/Figures/Bar_chart_summary/ACETYLENE_AAD.png]

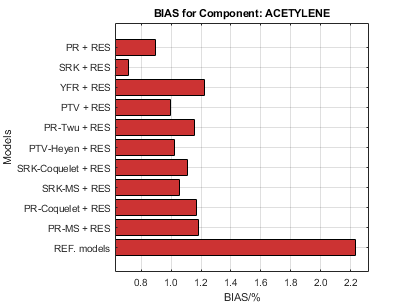

Supplement: Supplementary file 1 [file ao5c01157_si_001.zip › Supporting Information package 1/Figures/Bar_chart_summary/ACETYLENE_BIAS.png]

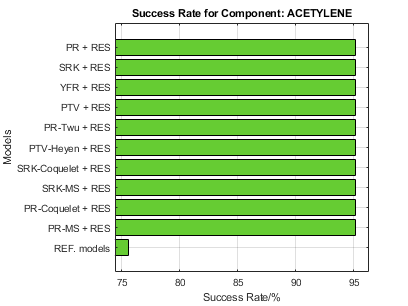

Supplement: Supplementary file 1 [file ao5c01157_si_001.zip › Supporting Information package 1/Figures/Bar_chart_summary/ACETYLENE_SuccessRate.png]

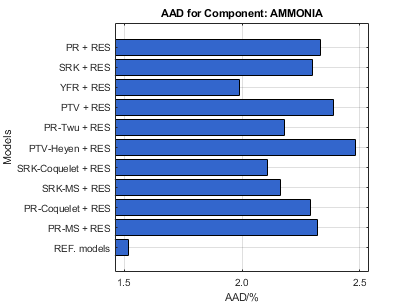

Supplement: Supplementary file 1 [file ao5c01157_si_001.zip › Supporting Information package 1/Figures/Bar_chart_summary/AMMONIA_AAD.png]

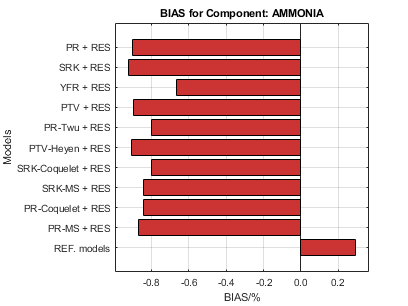

Supplement: Supplementary file 1 [file ao5c01157_si_001.zip › Supporting Information package 1/Figures/Bar_chart_summary/AMMONIA_BIAS.png]

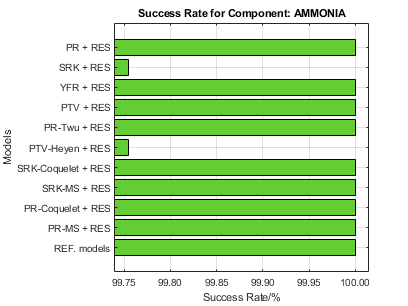

Supplement: Supplementary file 1 [file ao5c01157_si_001.zip › Supporting Information package 1/Figures/Bar_chart_summary/AMMONIA_SuccessRate.png]

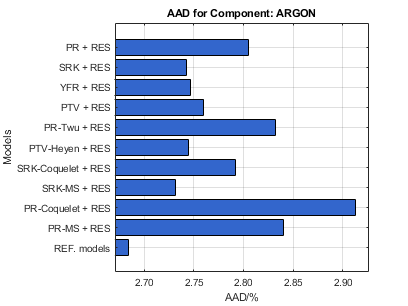

Supplement: Supplementary file 1 [file ao5c01157_si_001.zip › Supporting Information package 1/Figures/Bar_chart_summary/ARGON_AAD.png]

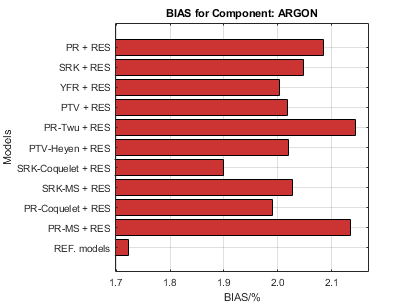

Supplement: Supplementary file 1 [file ao5c01157_si_001.zip › Supporting Information package 1/Figures/Bar_chart_summary/ARGON_BIAS.png]

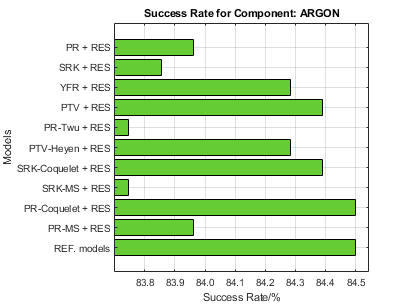

Supplement: Supplementary file 1 [file ao5c01157_si_001.zip › Supporting Information package 1/Figures/Bar_chart_summary/ARGON_SuccessRate.png]

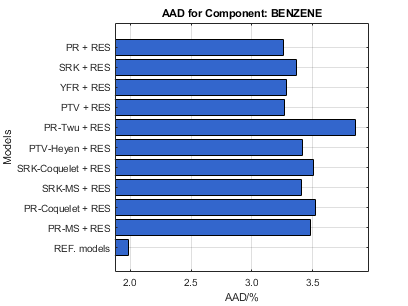

Supplement: Supplementary file 1 [file ao5c01157_si_001.zip › Supporting Information package 1/Figures/Bar_chart_summary/BENZENE_AAD.png]

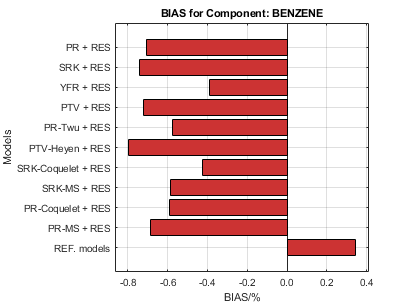

Supplement: Supplementary file 1 [file ao5c01157_si_001.zip › Supporting Information package 1/Figures/Bar_chart_summary/BENZENE_BIAS.png]

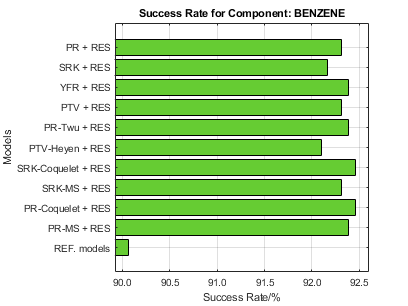

Supplement: Supplementary file 1 [file ao5c01157_si_001.zip › Supporting Information package 1/Figures/Bar_chart_summary/BENZENE_SuccessRate.png]

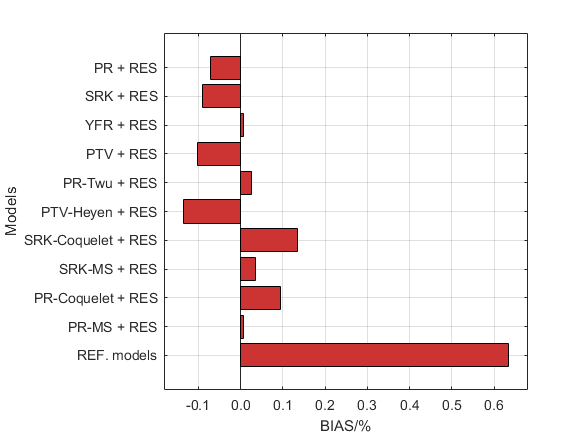

Supplement: Supplementary file 1 [file ao5c01157_si_001.zip › Supporting Information package 1/Figures/Bar_chart_summary/BIAS/overall_BIAS.png]

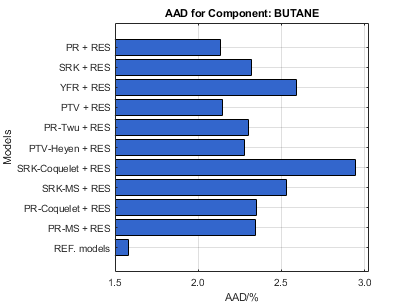

Supplement: Supplementary file 1 [file ao5c01157_si_001.zip › Supporting Information package 1/Figures/Bar_chart_summary/BUTANE_AAD.png]

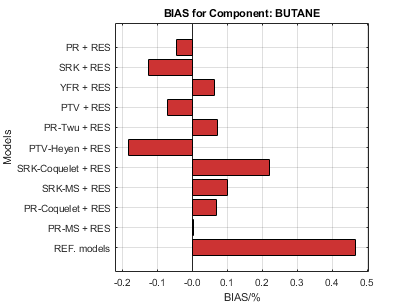

Supplement: Supplementary file 1 [file ao5c01157_si_001.zip › Supporting Information package 1/Figures/Bar_chart_summary/BUTANE_BIAS.png]

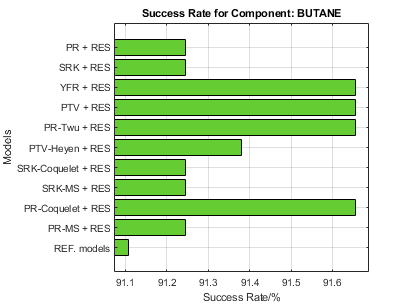

Supplement: Supplementary file 1 [file ao5c01157_si_001.zip › Supporting Information package 1/Figures/Bar_chart_summary/BUTANE_SuccessRate.png]

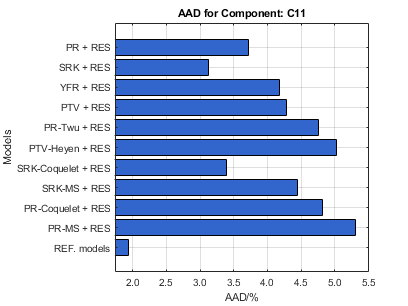

Supplement: Supplementary file 1 [file ao5c01157_si_001.zip › Supporting Information package 1/Figures/Bar_chart_summary/C11_AAD.png]

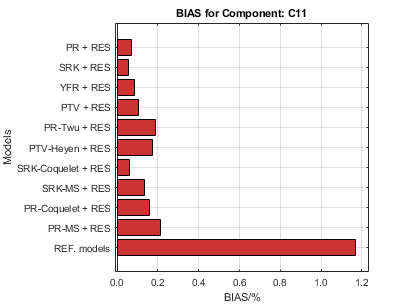

Supplement: Supplementary file 1 [file ao5c01157_si_001.zip › Supporting Information package 1/Figures/Bar_chart_summary/C11_BIAS.png]

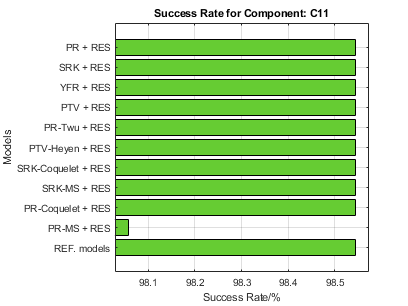

Supplement: Supplementary file 1 [file ao5c01157_si_001.zip › Supporting Information package 1/Figures/Bar_chart_summary/C11_SuccessRate.png]

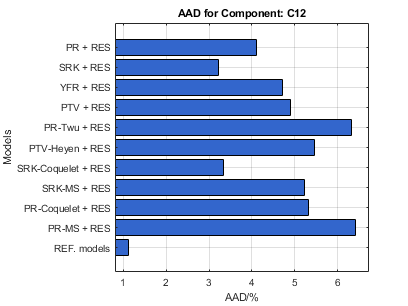

Supplement: Supplementary file 1 [file ao5c01157_si_001.zip › Supporting Information package 1/Figures/Bar_chart_summary/C12_AAD.png]

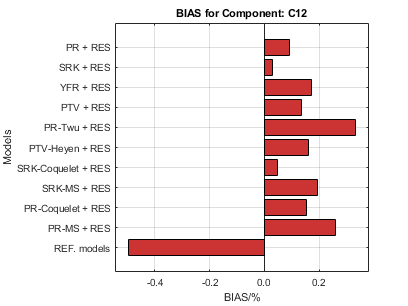

Supplement: Supplementary file 1 [file ao5c01157_si_001.zip › Supporting Information package 1/Figures/Bar_chart_summary/C12_BIAS.png]

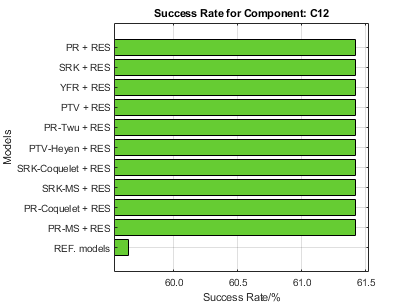

Supplement: Supplementary file 1 [file ao5c01157_si_001.zip › Supporting Information package 1/Figures/Bar_chart_summary/C12_SuccessRate.png]

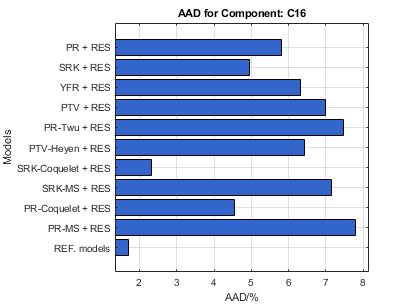

Supplement: Supplementary file 1 [file ao5c01157_si_001.zip › Supporting Information package 1/Figures/Bar_chart_summary/C16_AAD.png]

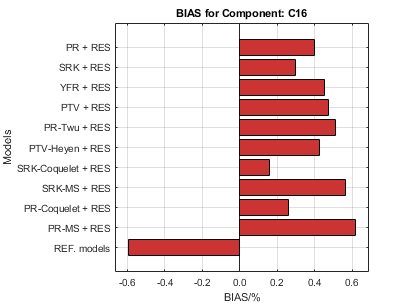

Supplement: Supplementary file 1 [file ao5c01157_si_001.zip › Supporting Information package 1/Figures/Bar_chart_summary/C16_BIAS.png]

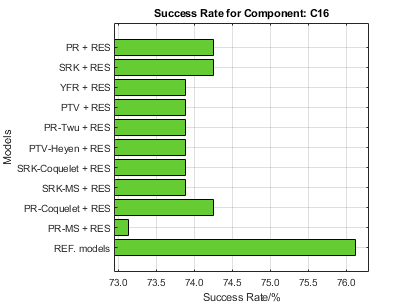

Supplement: Supplementary file 1 [file ao5c01157_si_001.zip › Supporting Information package 1/Figures/Bar_chart_summary/C16_SuccessRate.png]

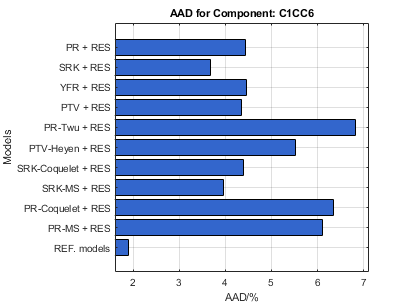

Supplement: Supplementary file 1 [file ao5c01157_si_001.zip › Supporting Information package 1/Figures/Bar_chart_summary/C1CC6_AAD.png]

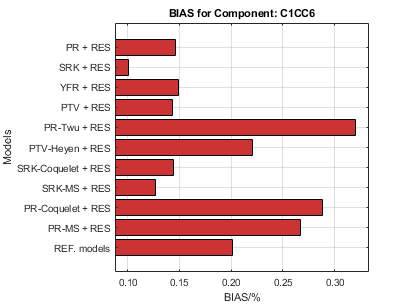

Supplement: Supplementary file 1 [file ao5c01157_si_001.zip › Supporting Information package 1/Figures/Bar_chart_summary/C1CC6_BIAS.png]

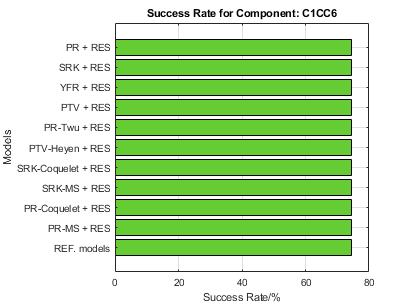

Supplement: Supplementary file 1 [file ao5c01157_si_001.zip › Supporting Information package 1/Figures/Bar_chart_summary/C1CC6_SuccessRate.png]

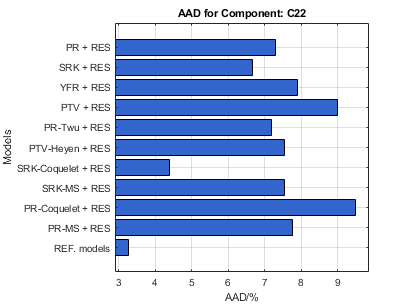

Supplement: Supplementary file 1 [file ao5c01157_si_001.zip › Supporting Information package 1/Figures/Bar_chart_summary/C22_AAD.png]

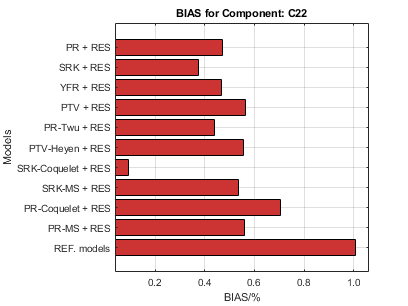

Supplement: Supplementary file 1 [file ao5c01157_si_001.zip › Supporting Information package 1/Figures/Bar_chart_summary/C22_BIAS.png]

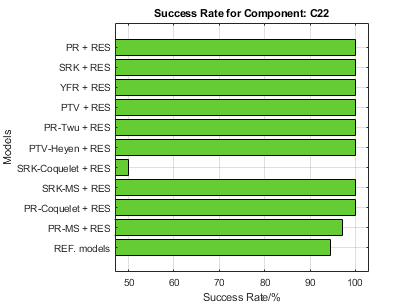

Supplement: Supplementary file 1 [file ao5c01157_si_001.zip › Supporting Information package 1/Figures/Bar_chart_summary/C22_SuccessRate.png]

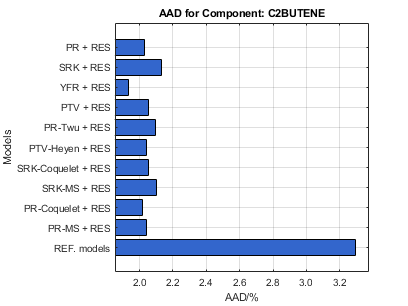

Supplement: Supplementary file 1 [file ao5c01157_si_001.zip › Supporting Information package 1/Figures/Bar_chart_summary/C2BUTENE_AAD.png]

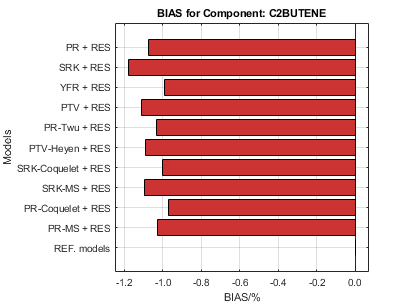

Supplement: Supplementary file 1 [file ao5c01157_si_001.zip › Supporting Information package 1/Figures/Bar_chart_summary/C2BUTENE_BIAS.png]

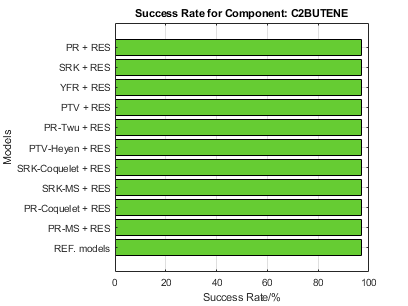

Supplement: Supplementary file 1 [file ao5c01157_si_001.zip › Supporting Information package 1/Figures/Bar_chart_summary/C2BUTENE_SuccessRate.png]

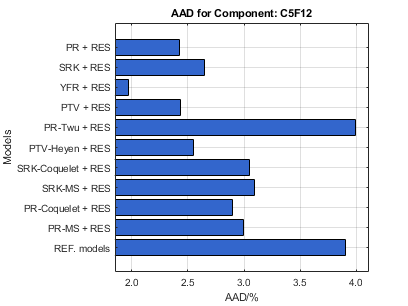

Supplement: Supplementary file 1 [file ao5c01157_si_001.zip › Supporting Information package 1/Figures/Bar_chart_summary/C5F12_AAD.png]

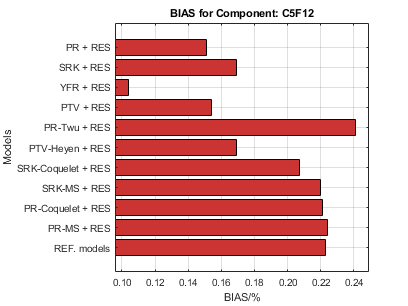

Supplement: Supplementary file 1 [file ao5c01157_si_001.zip › Supporting Information package 1/Figures/Bar_chart_summary/C5F12_BIAS.png]

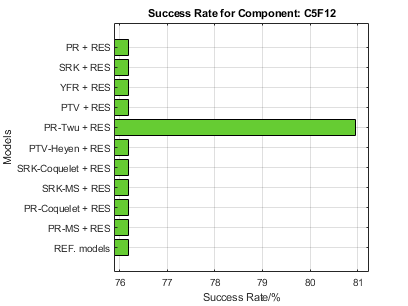

Supplement: Supplementary file 1 [file ao5c01157_si_001.zip › Supporting Information package 1/Figures/Bar_chart_summary/C5F12_SuccessRate.png]

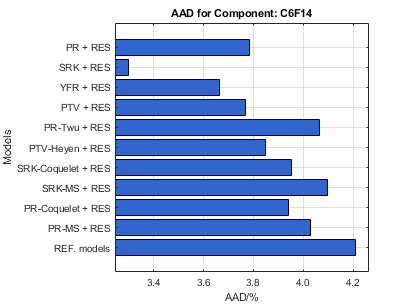

Supplement: Supplementary file 1 [file ao5c01157_si_001.zip › Supporting Information package 1/Figures/Bar_chart_summary/C6F14_AAD.png]

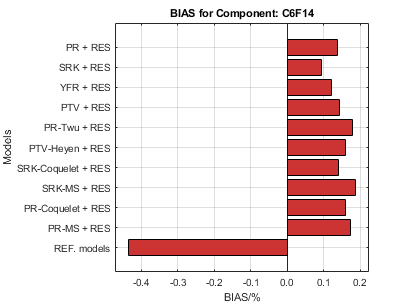

Supplement: Supplementary file 1 [file ao5c01157_si_001.zip › Supporting Information package 1/Figures/Bar_chart_summary/C6F14_BIAS.png]

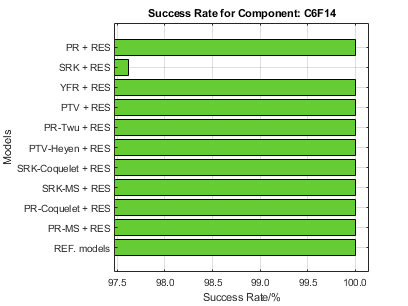

Supplement: Supplementary file 1 [file ao5c01157_si_001.zip › Supporting Information package 1/Figures/Bar_chart_summary/C6F14_SuccessRate.png]

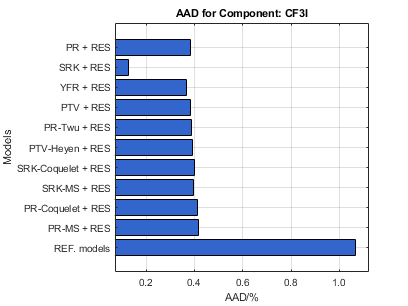

Supplement: Supplementary file 1 [file ao5c01157_si_001.zip › Supporting Information package 1/Figures/Bar_chart_summary/CF3I_AAD.png]

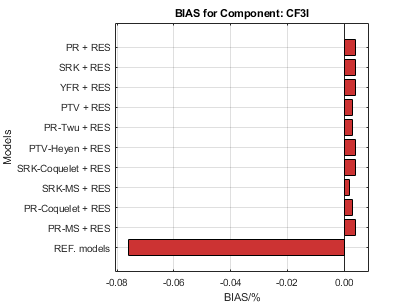

Supplement: Supplementary file 1 [file ao5c01157_si_001.zip › Supporting Information package 1/Figures/Bar_chart_summary/CF3I_BIAS.png]

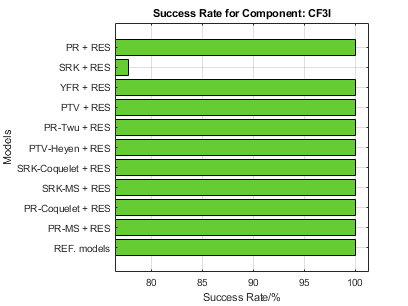

Supplement: Supplementary file 1 [file ao5c01157_si_001.zip › Supporting Information package 1/Figures/Bar_chart_summary/CF3I_SuccessRate.png]

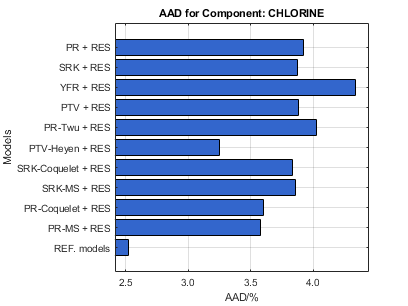

Supplement: Supplementary file 1 [file ao5c01157_si_001.zip › Supporting Information package 1/Figures/Bar_chart_summary/CHLORINE_AAD.png]

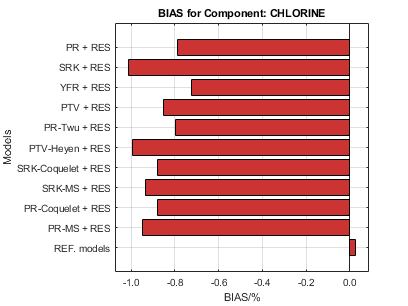

Supplement: Supplementary file 1 [file ao5c01157_si_001.zip › Supporting Information package 1/Figures/Bar_chart_summary/CHLORINE_BIAS.png]

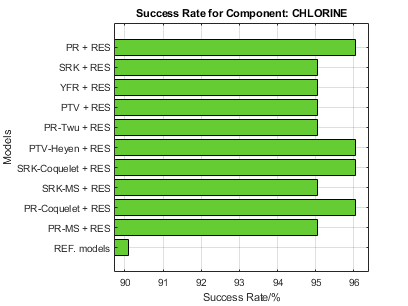

Supplement: Supplementary file 1 [file ao5c01157_si_001.zip › Supporting Information package 1/Figures/Bar_chart_summary/CHLORINE_SuccessRate.png]

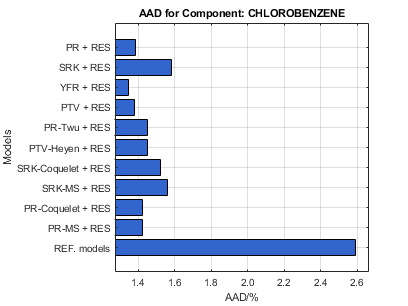

Supplement: Supplementary file 1 [file ao5c01157_si_001.zip › Supporting Information package 1/Figures/Bar_chart_summary/CHLOROBENZENE_AAD.png]

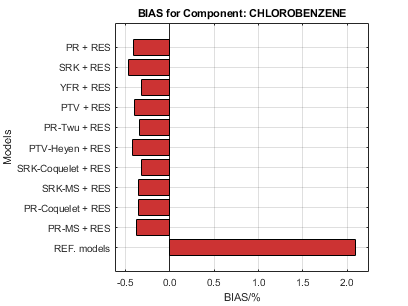

Supplement: Supplementary file 1 [file ao5c01157_si_001.zip › Supporting Information package 1/Figures/Bar_chart_summary/CHLOROBENZENE_BIAS.png]

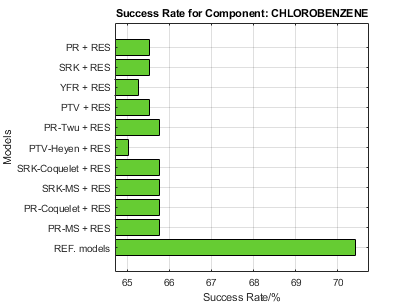

Supplement: Supplementary file 1 [file ao5c01157_si_001.zip › Supporting Information package 1/Figures/Bar_chart_summary/CHLOROBENZENE_SuccessRate.png]

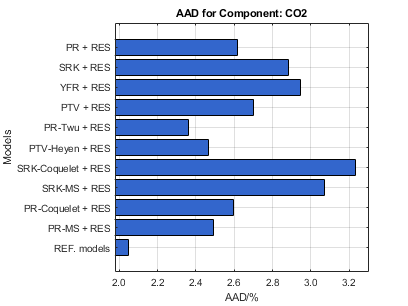

Supplement: Supplementary file 1 [file ao5c01157_si_001.zip › Supporting Information package 1/Figures/Bar_chart_summary/CO2_AAD.png]

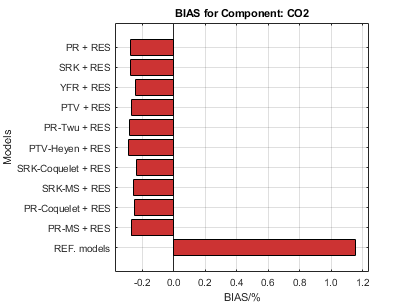

Supplement: Supplementary file 1 [file ao5c01157_si_001.zip › Supporting Information package 1/Figures/Bar_chart_summary/CO2_BIAS.png]

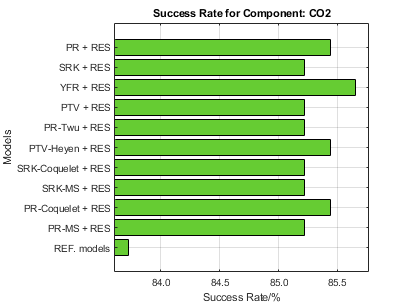

Supplement: Supplementary file 1 [file ao5c01157_si_001.zip › Supporting Information package 1/Figures/Bar_chart_summary/CO2_SuccessRate.png]

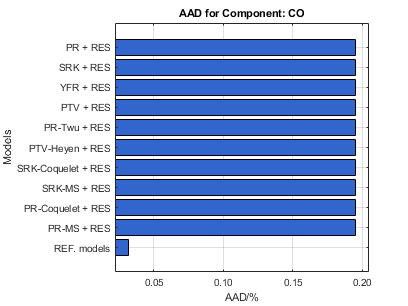

Supplement: Supplementary file 1 [file ao5c01157_si_001.zip › Supporting Information package 1/Figures/Bar_chart_summary/CO_AAD.png]

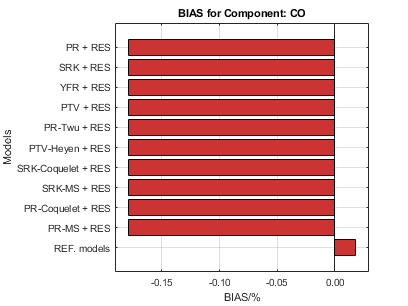

Supplement: Supplementary file 1 [file ao5c01157_si_001.zip › Supporting Information package 1/Figures/Bar_chart_summary/CO_BIAS.png]

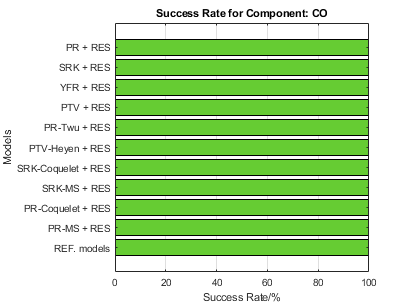

Supplement: Supplementary file 1 [file ao5c01157_si_001.zip › Supporting Information package 1/Figures/Bar_chart_summary/CO_SuccessRate.png]

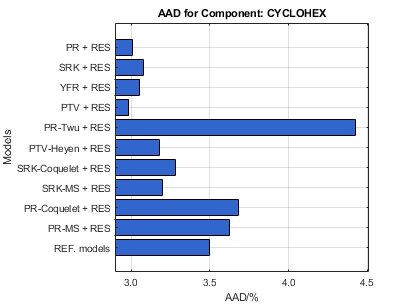

Supplement: Supplementary file 1 [file ao5c01157_si_001.zip › Supporting Information package 1/Figures/Bar_chart_summary/CYCLOHEX_AAD.png]

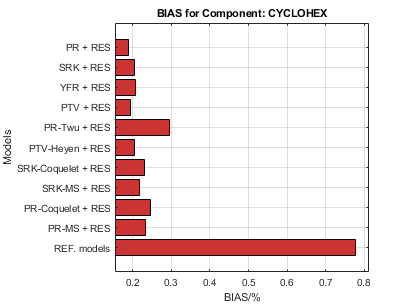

Supplement: Supplementary file 1 [file ao5c01157_si_001.zip › Supporting Information package 1/Figures/Bar_chart_summary/CYCLOHEX_BIAS.png]

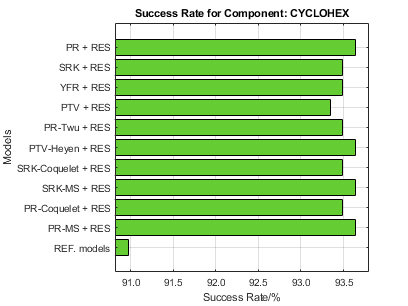

Supplement: Supplementary file 1 [file ao5c01157_si_001.zip › Supporting Information package 1/Figures/Bar_chart_summary/CYCLOHEX_SuccessRate.png]

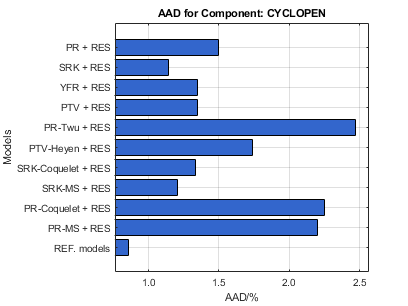

Supplement: Supplementary file 1 [file ao5c01157_si_001.zip › Supporting Information package 1/Figures/Bar_chart_summary/CYCLOPEN_AAD.png]

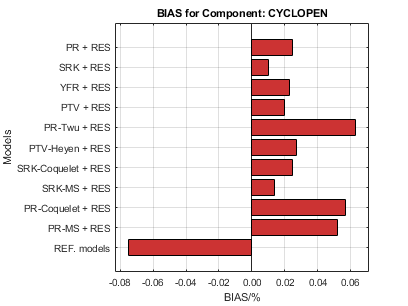

Supplement: Supplementary file 1 [file ao5c01157_si_001.zip › Supporting Information package 1/Figures/Bar_chart_summary/CYCLOPEN_BIAS.png]

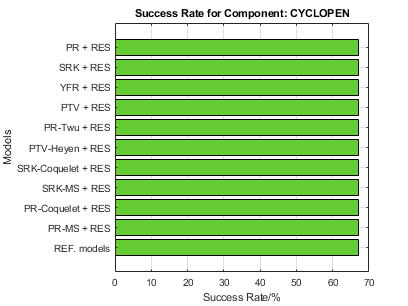

Supplement: Supplementary file 1 [file ao5c01157_si_001.zip › Supporting Information package 1/Figures/Bar_chart_summary/CYCLOPEN_SuccessRate.png]

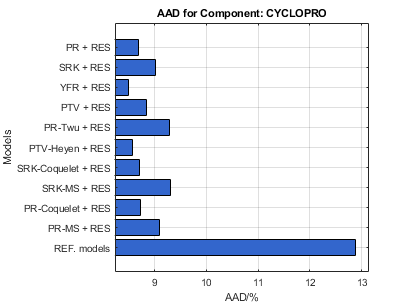

Supplement: Supplementary file 1 [file ao5c01157_si_001.zip › Supporting Information package 1/Figures/Bar_chart_summary/CYCLOPRO_AAD.png]

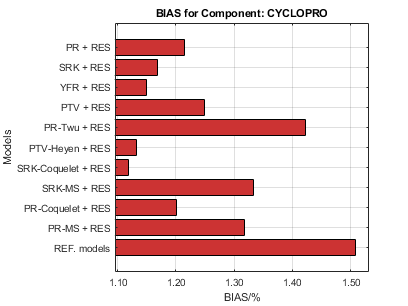

Supplement: Supplementary file 1 [file ao5c01157_si_001.zip › Supporting Information package 1/Figures/Bar_chart_summary/CYCLOPRO_BIAS.png]

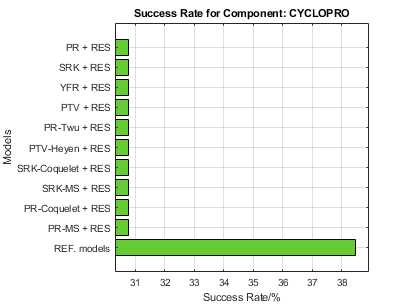

Supplement: Supplementary file 1 [file ao5c01157_si_001.zip › Supporting Information package 1/Figures/Bar_chart_summary/CYCLOPRO_SuccessRate.png]

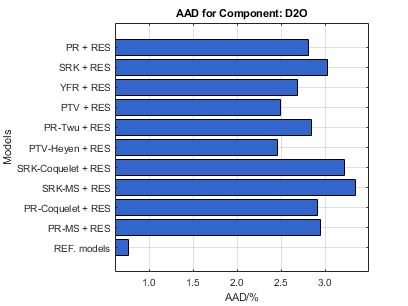

Supplement: Supplementary file 1 [file ao5c01157_si_001.zip › Supporting Information package 1/Figures/Bar_chart_summary/D2O_AAD.png]

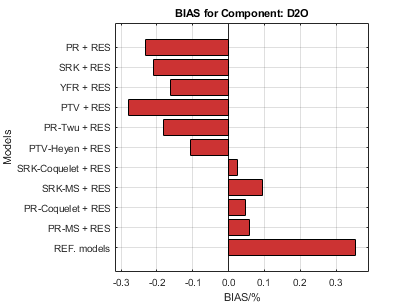

Supplement: Supplementary file 1 [file ao5c01157_si_001.zip › Supporting Information package 1/Figures/Bar_chart_summary/D2O_BIAS.png]

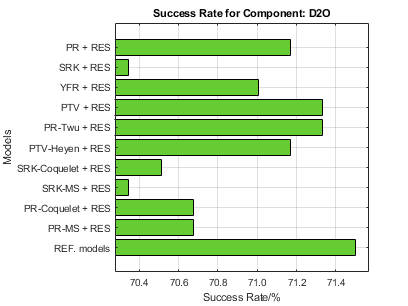

Supplement: Supplementary file 1 [file ao5c01157_si_001.zip › Supporting Information package 1/Figures/Bar_chart_summary/D2O_SuccessRate.png]

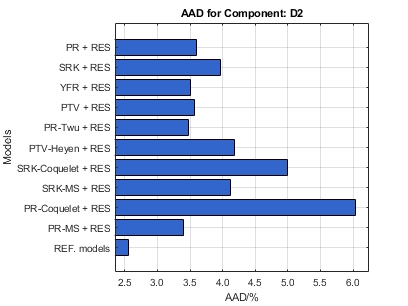

Supplement: Supplementary file 1 [file ao5c01157_si_001.zip › Supporting Information package 1/Figures/Bar_chart_summary/D2_AAD.png]

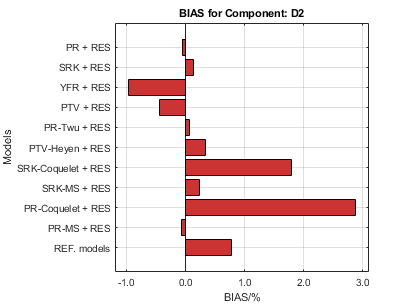

Supplement: Supplementary file 1 [file ao5c01157_si_001.zip › Supporting Information package 1/Figures/Bar_chart_summary/D2_BIAS.png]

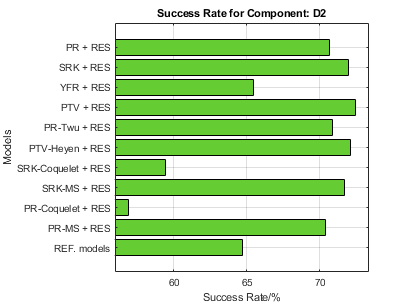

Supplement: Supplementary file 1 [file ao5c01157_si_001.zip › Supporting Information package 1/Figures/Bar_chart_summary/D2_SuccessRate.png]

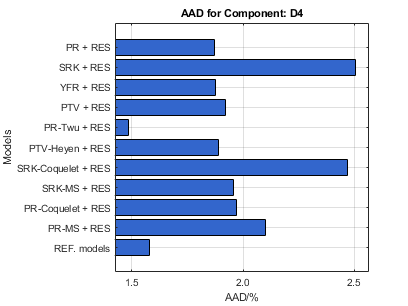

Supplement: Supplementary file 1 [file ao5c01157_si_001.zip › Supporting Information package 1/Figures/Bar_chart_summary/D4_AAD.png]

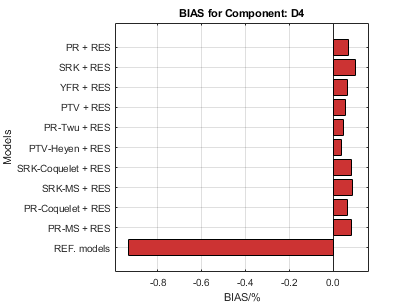

Supplement: Supplementary file 1 [file ao5c01157_si_001.zip › Supporting Information package 1/Figures/Bar_chart_summary/D4_BIAS.png]

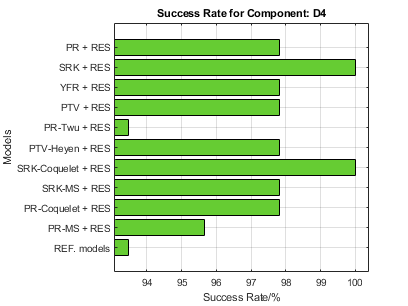

Supplement: Supplementary file 1 [file ao5c01157_si_001.zip › Supporting Information package 1/Figures/Bar_chart_summary/D4_SuccessRate.png]

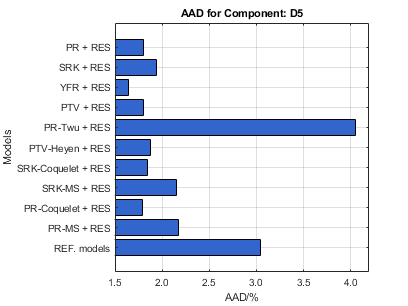

Supplement: Supplementary file 1 [file ao5c01157_si_001.zip › Supporting Information package 1/Figures/Bar_chart_summary/D5_AAD.png]

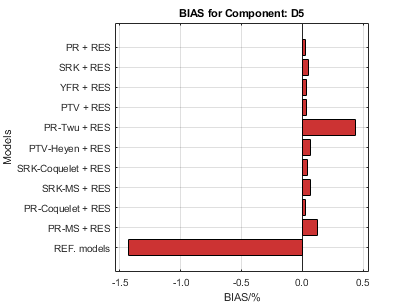

Supplement: Supplementary file 1 [file ao5c01157_si_001.zip › Supporting Information package 1/Figures/Bar_chart_summary/D5_BIAS.png]

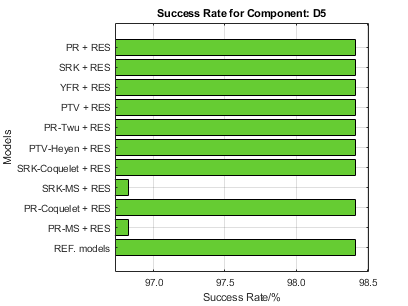

Supplement: Supplementary file 1 [file ao5c01157_si_001.zip › Supporting Information package 1/Figures/Bar_chart_summary/D5_SuccessRate.png]

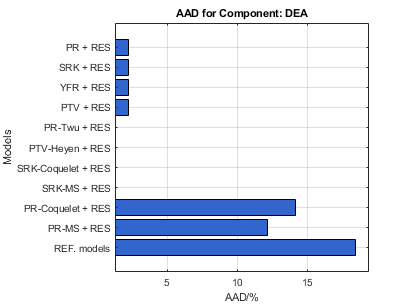

Supplement: Supplementary file 1 [file ao5c01157_si_001.zip › Supporting Information package 1/Figures/Bar_chart_summary/DEA_AAD.png]

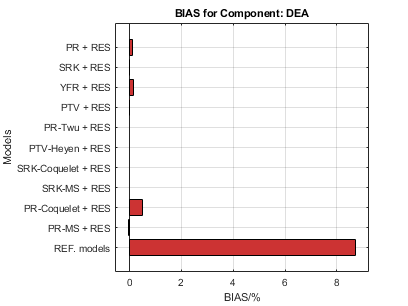

Supplement: Supplementary file 1 [file ao5c01157_si_001.zip › Supporting Information package 1/Figures/Bar_chart_summary/DEA_BIAS.png]

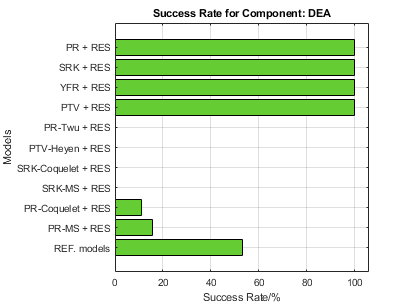

Supplement: Supplementary file 1 [file ao5c01157_si_001.zip › Supporting Information package 1/Figures/Bar_chart_summary/DEA_SuccessRate.png]

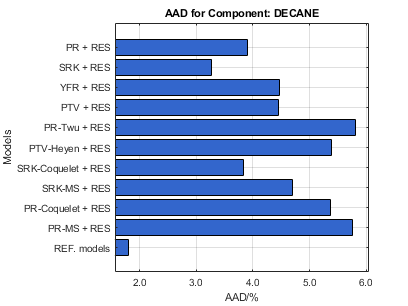

Supplement: Supplementary file 1 [file ao5c01157_si_001.zip › Supporting Information package 1/Figures/Bar_chart_summary/DECANE_AAD.png]

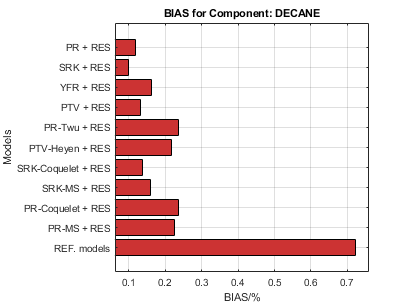

Supplement: Supplementary file 1 [file ao5c01157_si_001.zip › Supporting Information package 1/Figures/Bar_chart_summary/DECANE_BIAS.png]
