# Supplementary figures and images for: Evaluating Cubic Equations of State with Various α Functions for Viscosity Predictions of 124 Industrial Important Fluids Based on Residual Entropy Scaling (part 4 of 4)
Source: ACS Omega. 2025 Jun 27;10(27):29021–36. doi: 10.1021/acsomega.5c01157 (PMC12268422; doi:10.1021/acsomega.5c01157)

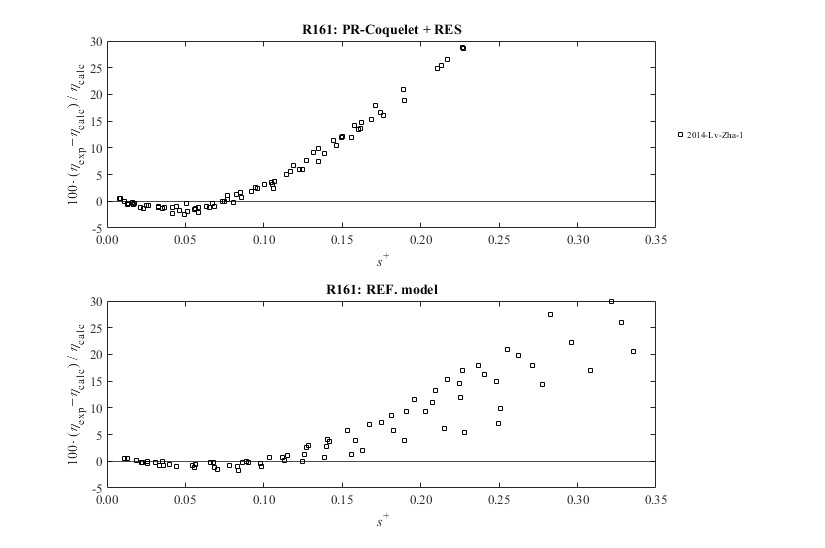

Supplement: Supplementary file 2 [file ao5c01157_si_002.zip › Supporting Information package 2/Figures/Deviation plots/PR-Coquelet/R161.jpeg]

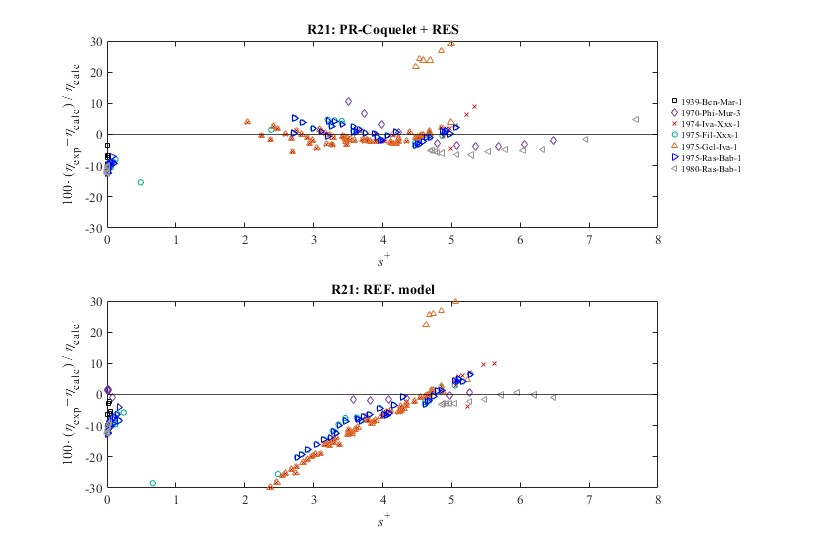

Supplement: Supplementary file 2 [file ao5c01157_si_002.zip › Supporting Information package 2/Figures/Deviation plots/PR-Coquelet/R21.jpeg]

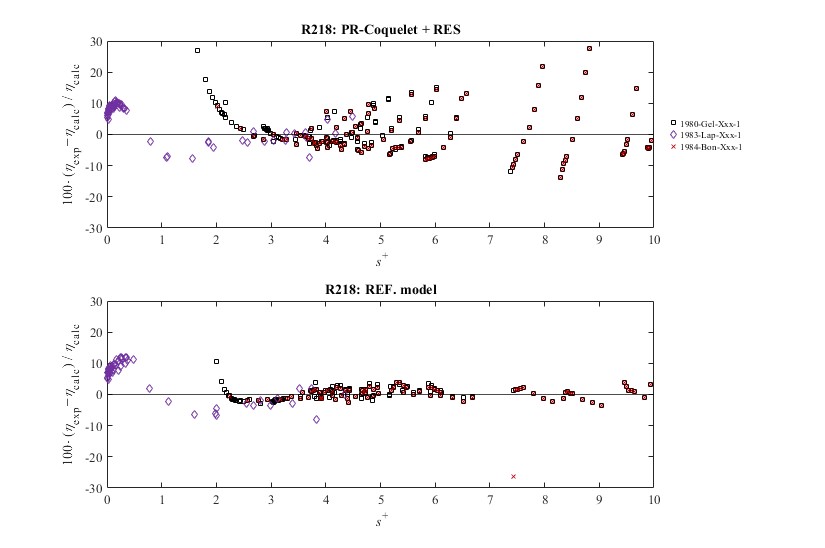

Supplement: Supplementary file 2 [file ao5c01157_si_002.zip › Supporting Information package 2/Figures/Deviation plots/PR-Coquelet/R218.jpeg]

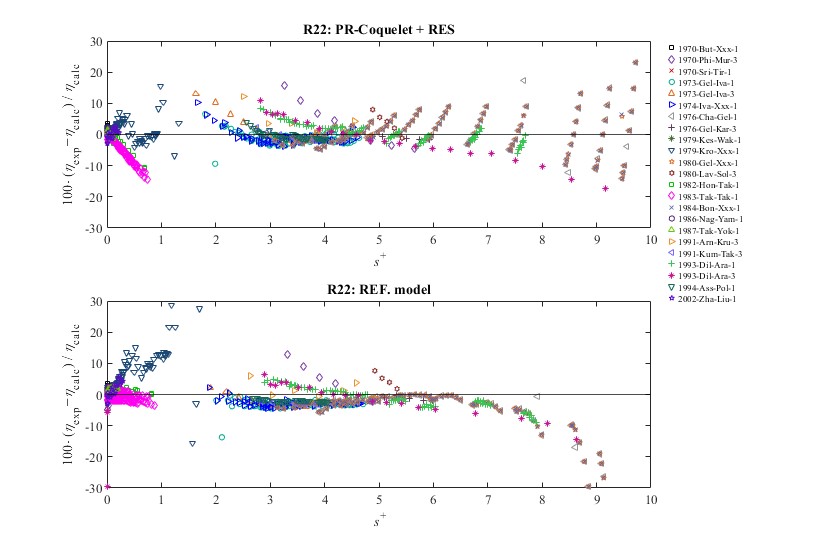

Supplement: Supplementary file 2 [file ao5c01157_si_002.zip › Supporting Information package 2/Figures/Deviation plots/PR-Coquelet/R22.jpeg]

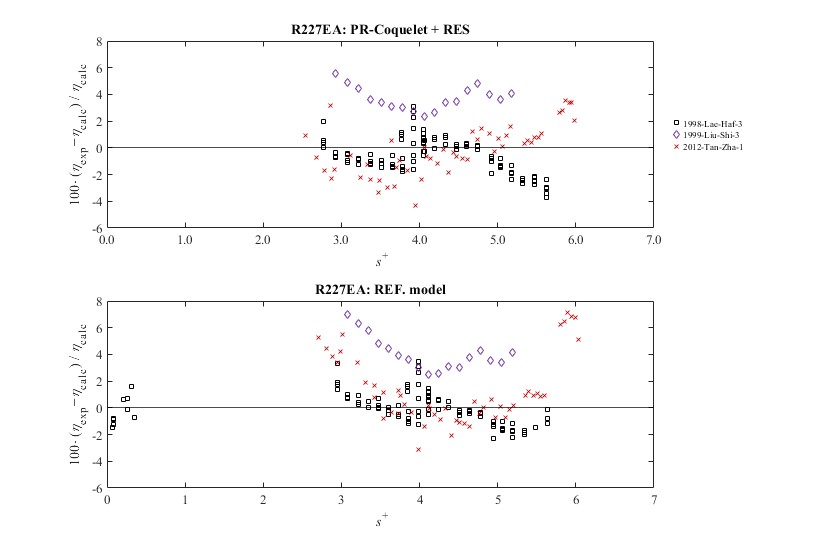

Supplement: Supplementary file 2 [file ao5c01157_si_002.zip › Supporting Information package 2/Figures/Deviation plots/PR-Coquelet/R227EA.jpeg]

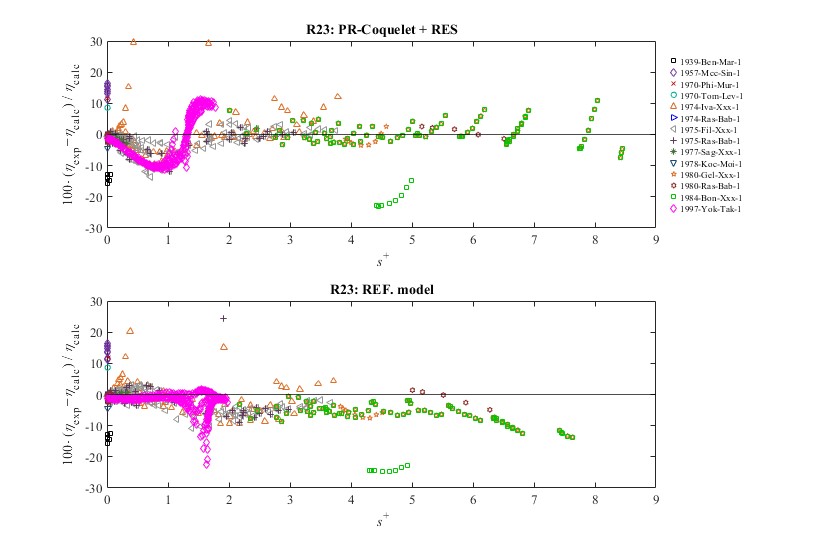

Supplement: Supplementary file 2 [file ao5c01157_si_002.zip › Supporting Information package 2/Figures/Deviation plots/PR-Coquelet/R23.jpeg]

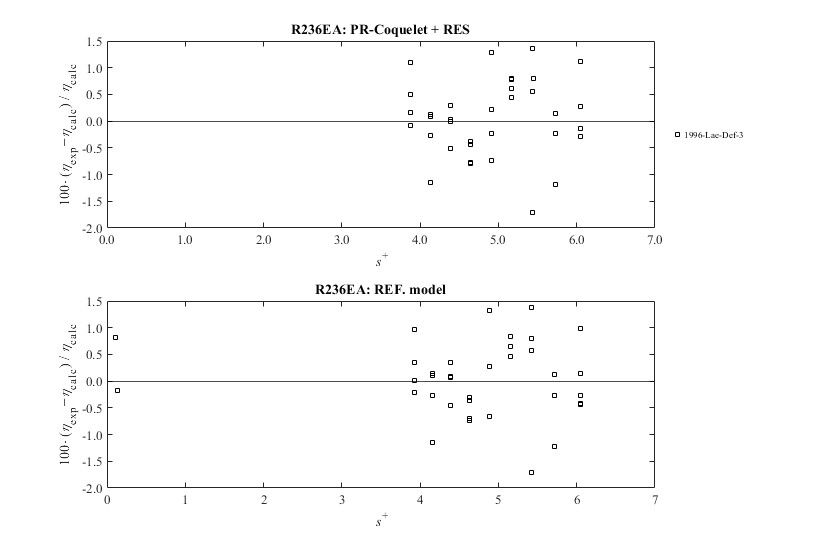

Supplement: Supplementary file 2 [file ao5c01157_si_002.zip › Supporting Information package 2/Figures/Deviation plots/PR-Coquelet/R236EA.jpeg]

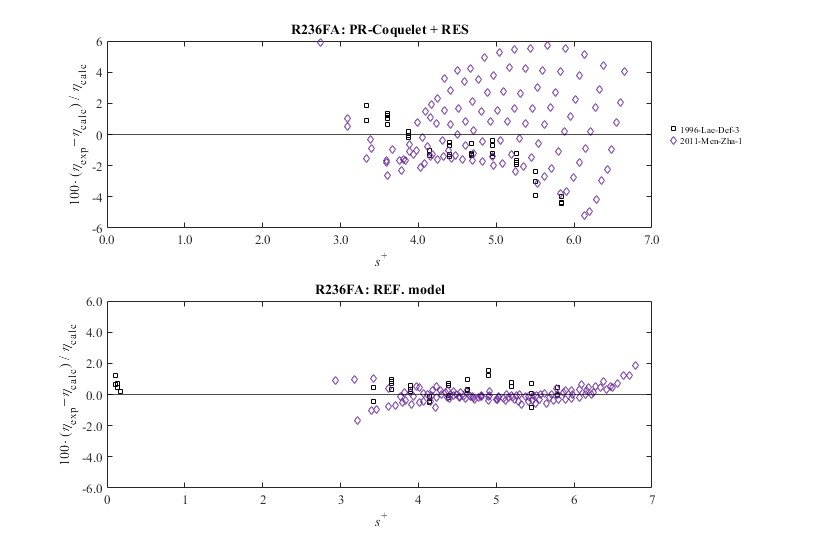

Supplement: Supplementary file 2 [file ao5c01157_si_002.zip › Supporting Information package 2/Figures/Deviation plots/PR-Coquelet/R236FA.jpeg]

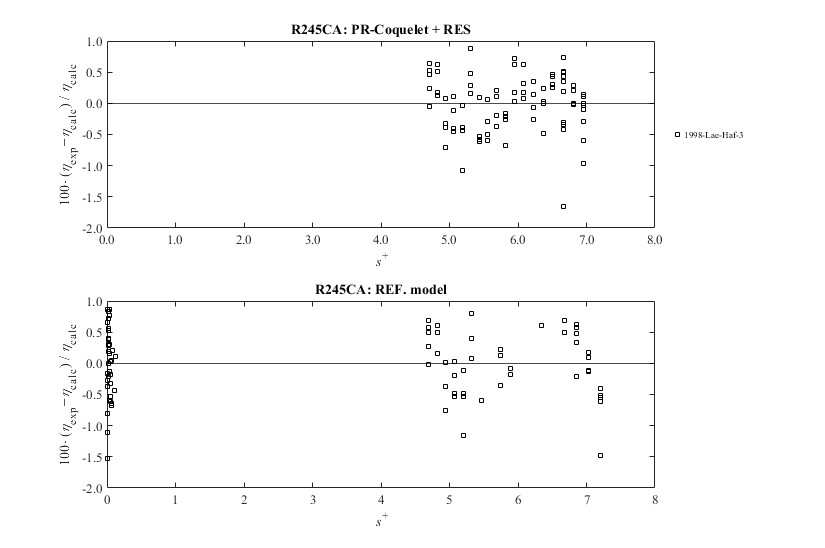

Supplement: Supplementary file 2 [file ao5c01157_si_002.zip › Supporting Information package 2/Figures/Deviation plots/PR-Coquelet/R245CA.jpeg]

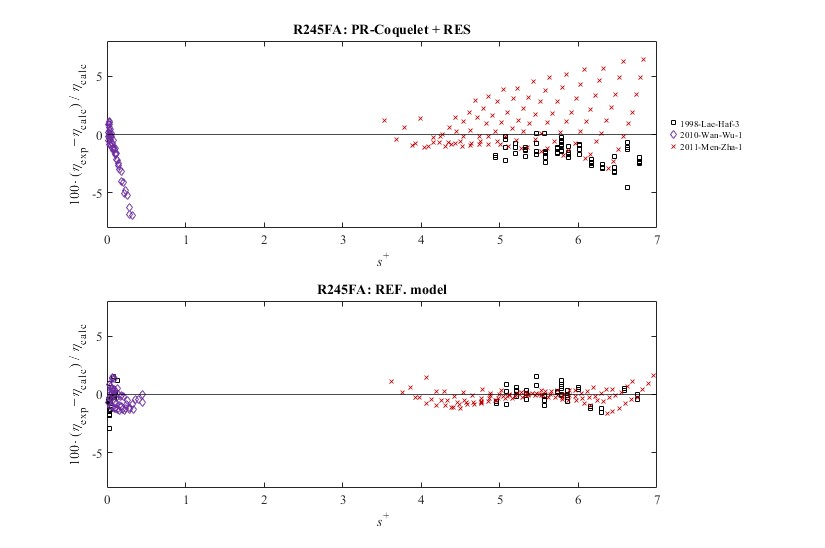

Supplement: Supplementary file 2 [file ao5c01157_si_002.zip › Supporting Information package 2/Figures/Deviation plots/PR-Coquelet/R245FA.jpeg]

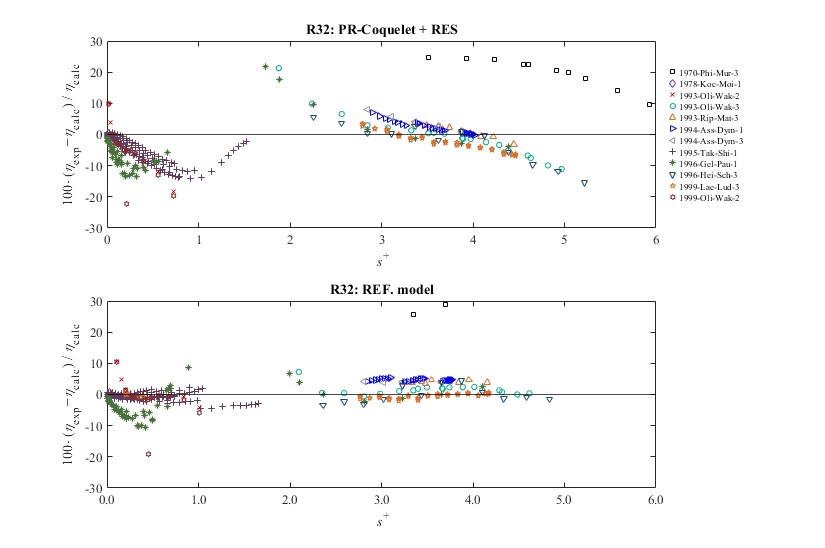

Supplement: Supplementary file 2 [file ao5c01157_si_002.zip › Supporting Information package 2/Figures/Deviation plots/PR-Coquelet/R32.jpeg]

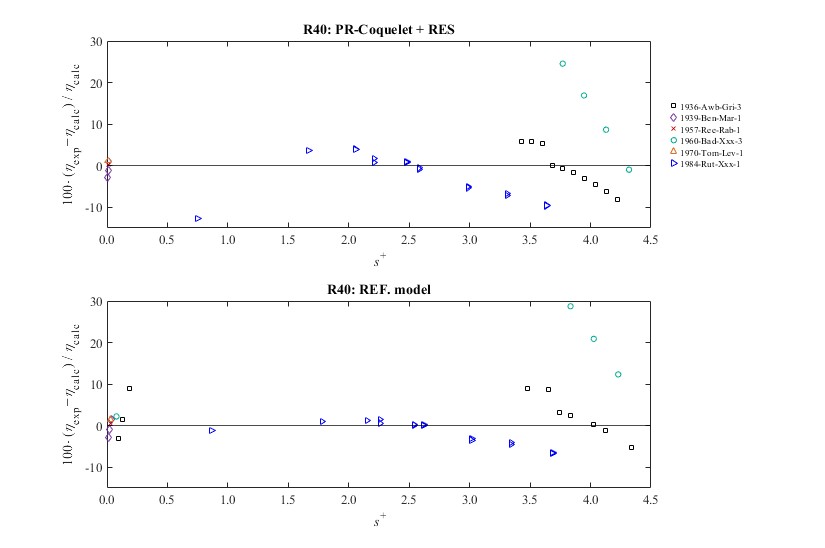

Supplement: Supplementary file 2 [file ao5c01157_si_002.zip › Supporting Information package 2/Figures/Deviation plots/PR-Coquelet/R40.jpeg]

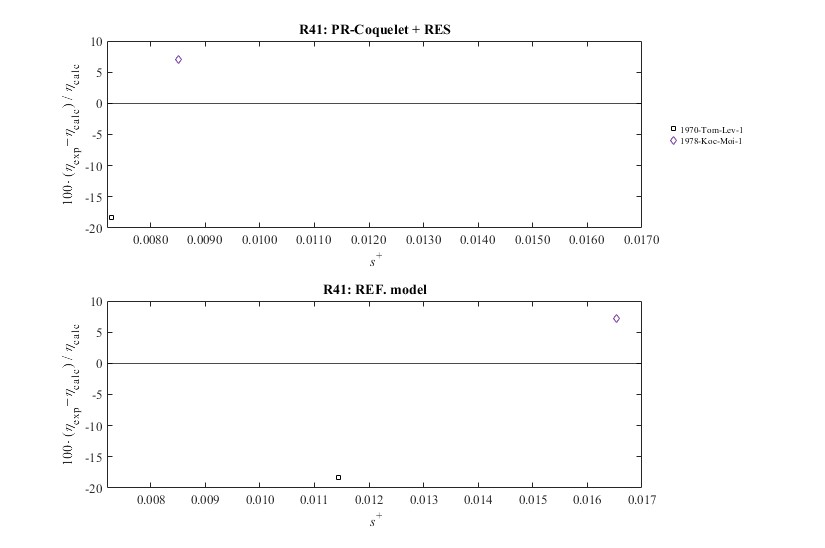

Supplement: Supplementary file 2 [file ao5c01157_si_002.zip › Supporting Information package 2/Figures/Deviation plots/PR-Coquelet/R41.jpeg]

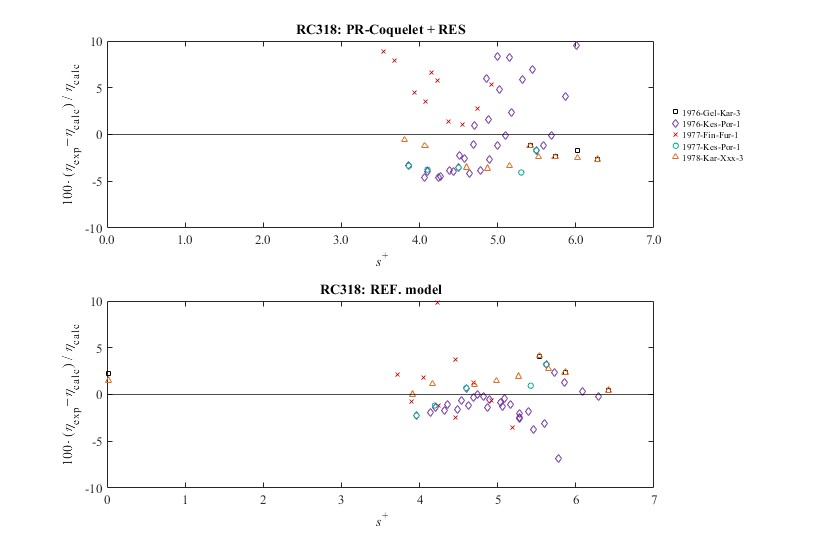

Supplement: Supplementary file 2 [file ao5c01157_si_002.zip › Supporting Information package 2/Figures/Deviation plots/PR-Coquelet/RC318.jpeg]

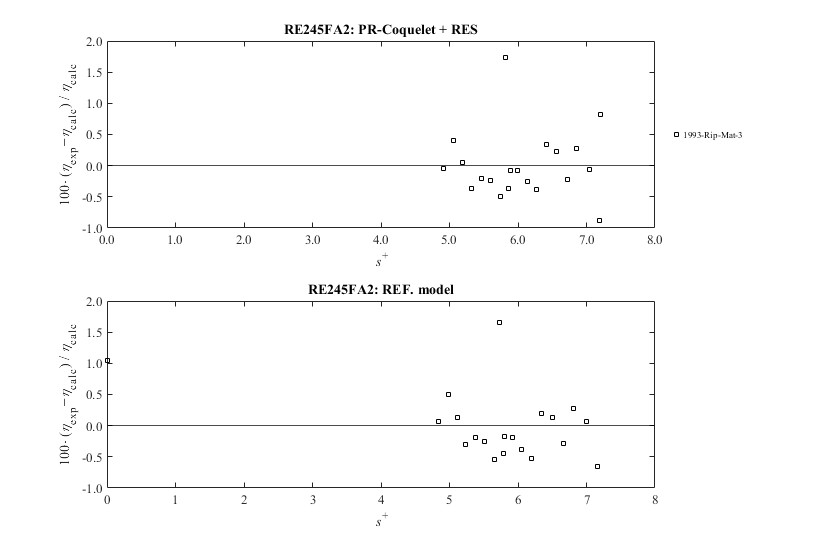

Supplement: Supplementary file 2 [file ao5c01157_si_002.zip › Supporting Information package 2/Figures/Deviation plots/PR-Coquelet/RE245FA2.jpeg]

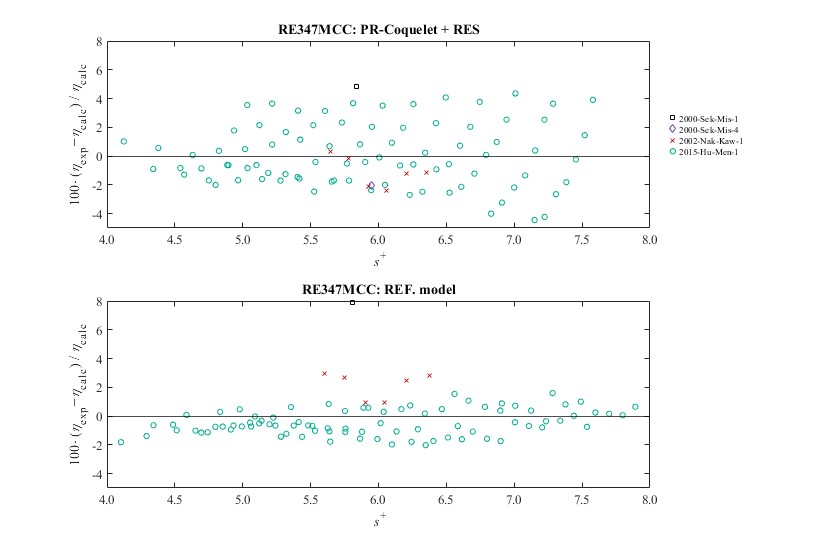

Supplement: Supplementary file 2 [file ao5c01157_si_002.zip › Supporting Information package 2/Figures/Deviation plots/PR-Coquelet/RE347MCC.jpeg]

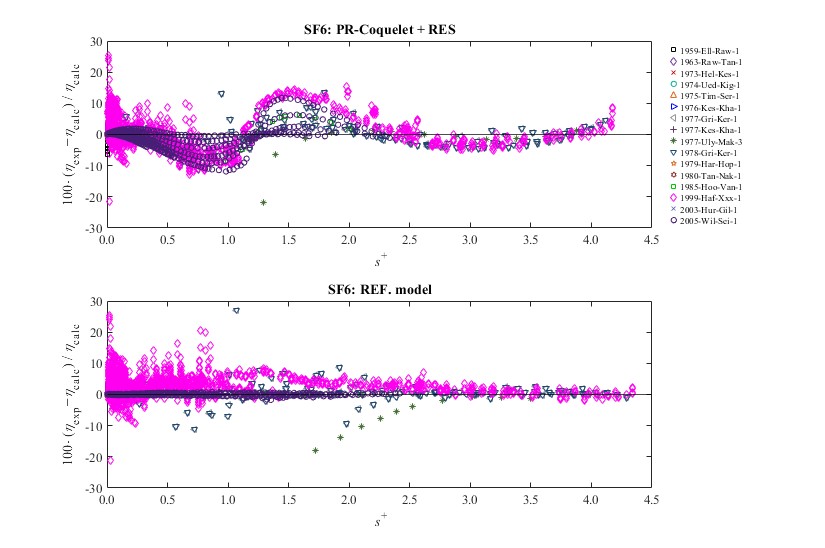

Supplement: Supplementary file 2 [file ao5c01157_si_002.zip › Supporting Information package 2/Figures/Deviation plots/PR-Coquelet/SF6.jpeg]

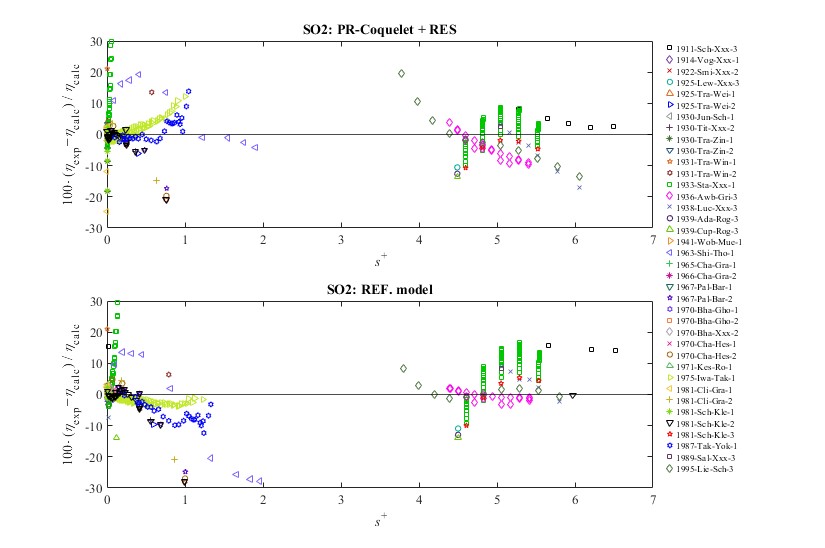

Supplement: Supplementary file 2 [file ao5c01157_si_002.zip › Supporting Information package 2/Figures/Deviation plots/PR-Coquelet/SO2.jpeg]

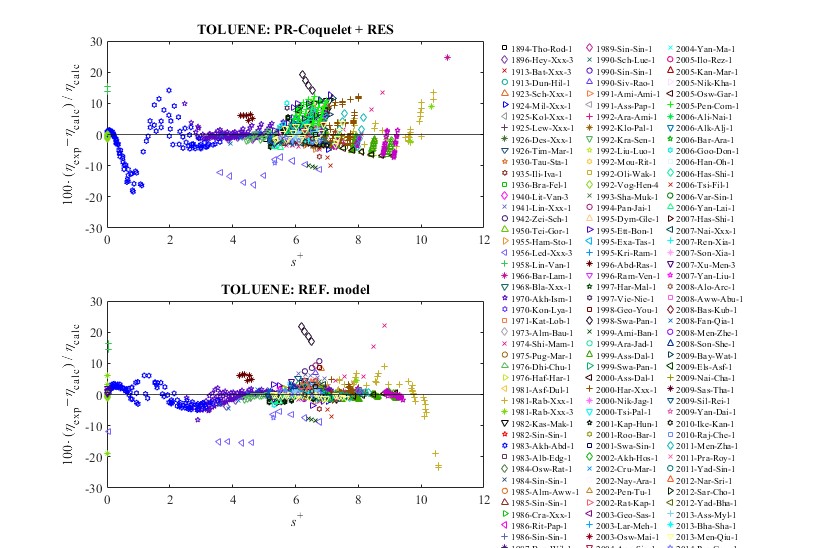

Supplement: Supplementary file 2 [file ao5c01157_si_002.zip › Supporting Information package 2/Figures/Deviation plots/PR-Coquelet/TOLUENE.jpeg]

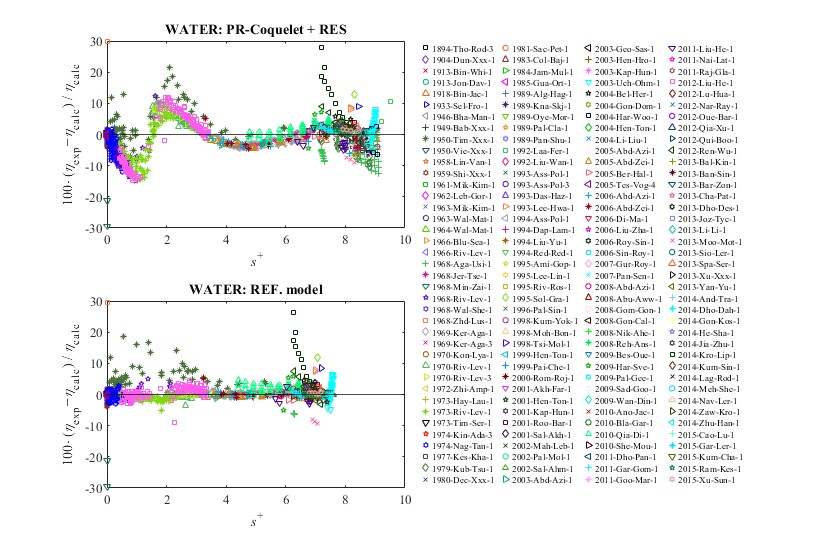

Supplement: Supplementary file 2 [file ao5c01157_si_002.zip › Supporting Information package 2/Figures/Deviation plots/PR-Coquelet/WATER.jpeg]

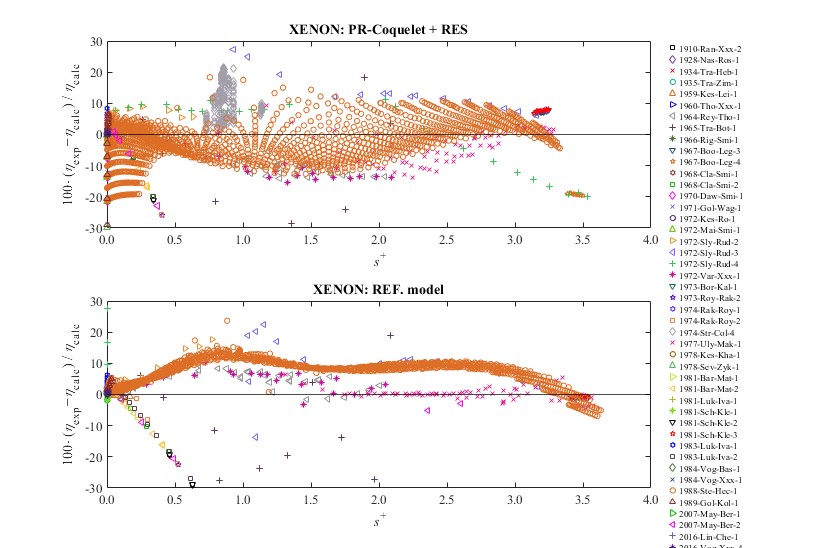

Supplement: Supplementary file 2 [file ao5c01157_si_002.zip › Supporting Information package 2/Figures/Deviation plots/PR-Coquelet/XENON.jpeg]

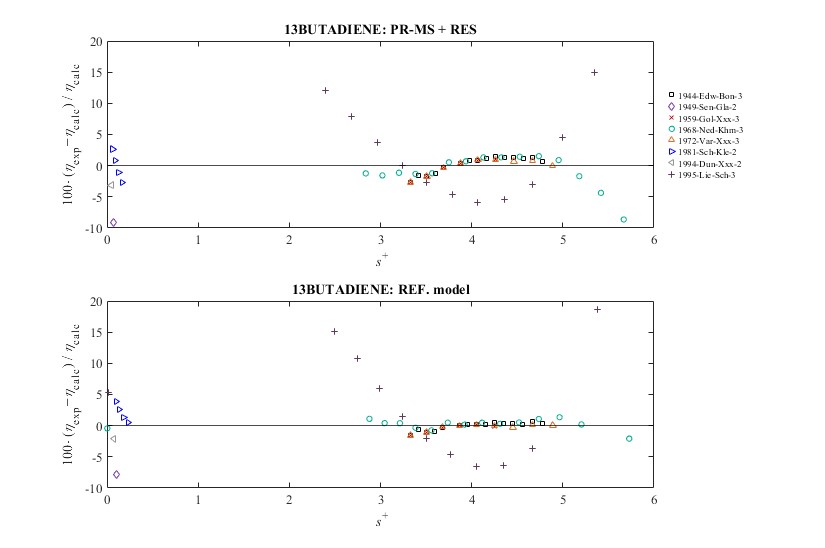

Supplement: Supplementary file 2 [file ao5c01157_si_002.zip › Supporting Information package 2/Figures/Deviation plots/PR-MS/13BUTADIENE.jpeg]

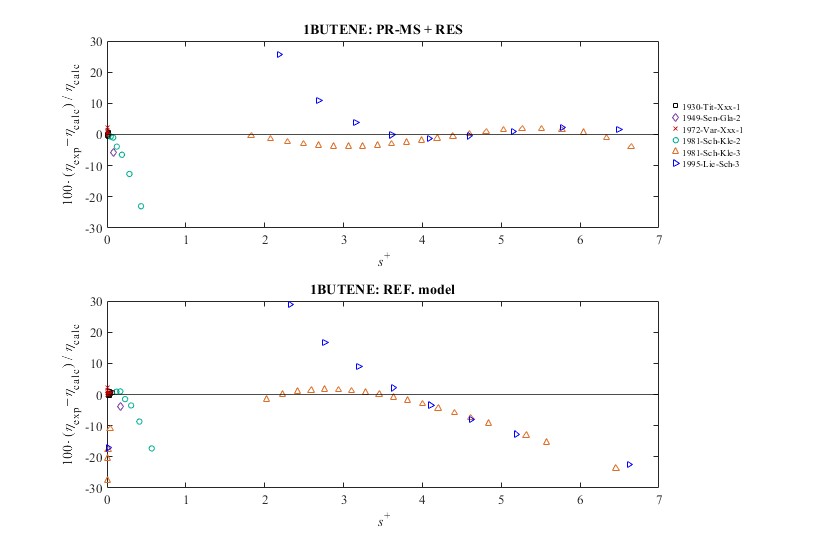

Supplement: Supplementary file 2 [file ao5c01157_si_002.zip › Supporting Information package 2/Figures/Deviation plots/PR-MS/1BUTENE.jpeg]

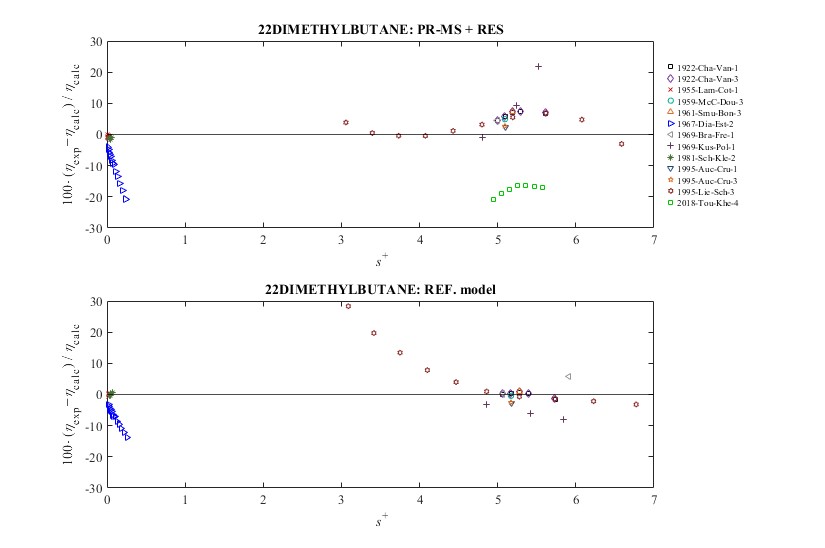

Supplement: Supplementary file 2 [file ao5c01157_si_002.zip › Supporting Information package 2/Figures/Deviation plots/PR-MS/22DIMETHYLBUTANE.jpeg]

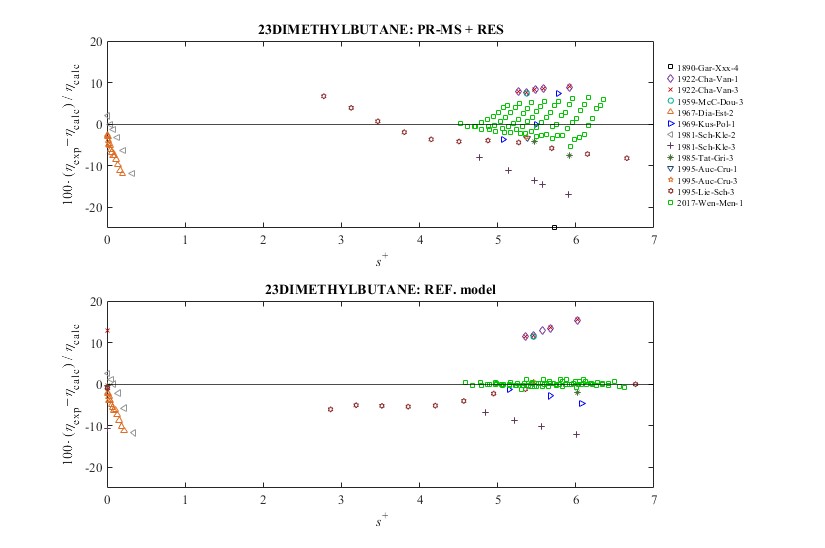

Supplement: Supplementary file 2 [file ao5c01157_si_002.zip › Supporting Information package 2/Figures/Deviation plots/PR-MS/23DIMETHYLBUTANE.jpeg]

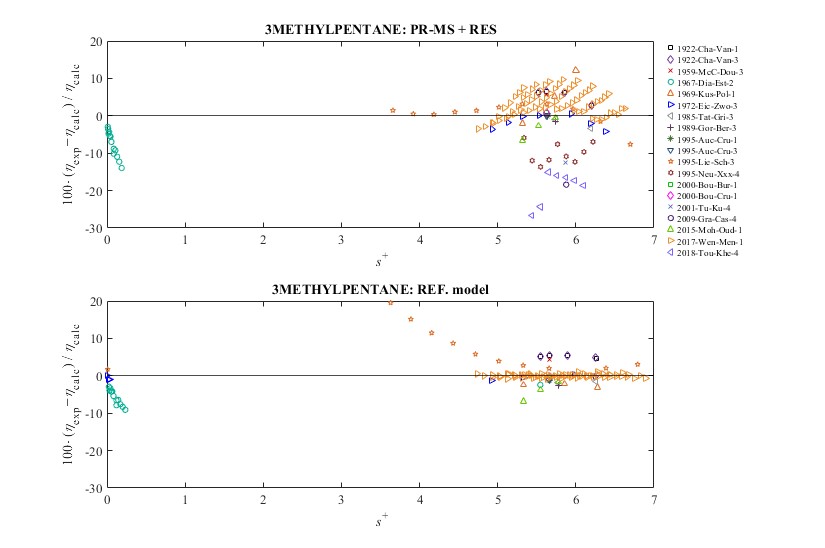

Supplement: Supplementary file 2 [file ao5c01157_si_002.zip › Supporting Information package 2/Figures/Deviation plots/PR-MS/3METHYLPENTANE.jpeg]

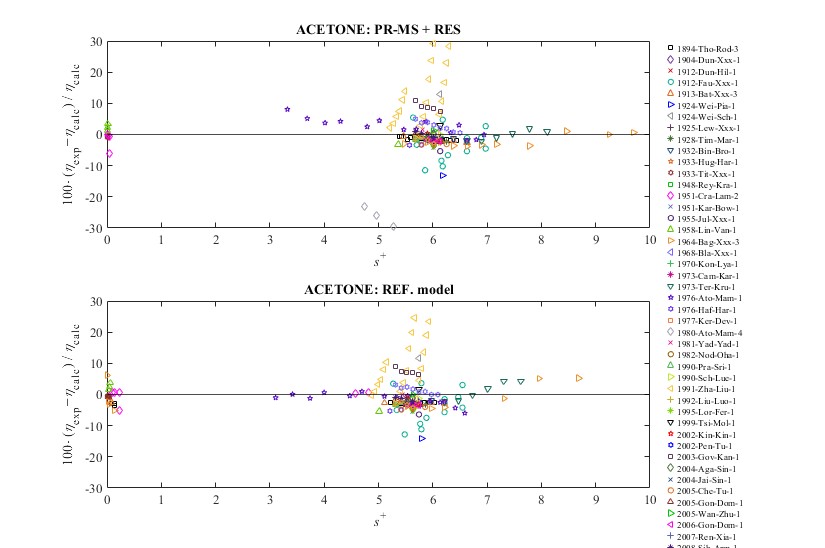

Supplement: Supplementary file 2 [file ao5c01157_si_002.zip › Supporting Information package 2/Figures/Deviation plots/PR-MS/ACETONE.jpeg]

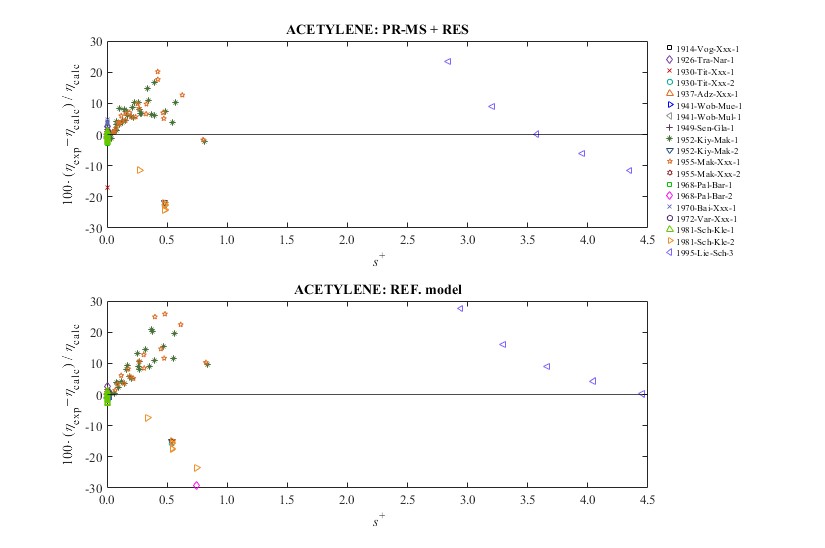

Supplement: Supplementary file 2 [file ao5c01157_si_002.zip › Supporting Information package 2/Figures/Deviation plots/PR-MS/ACETYLENE.jpeg]

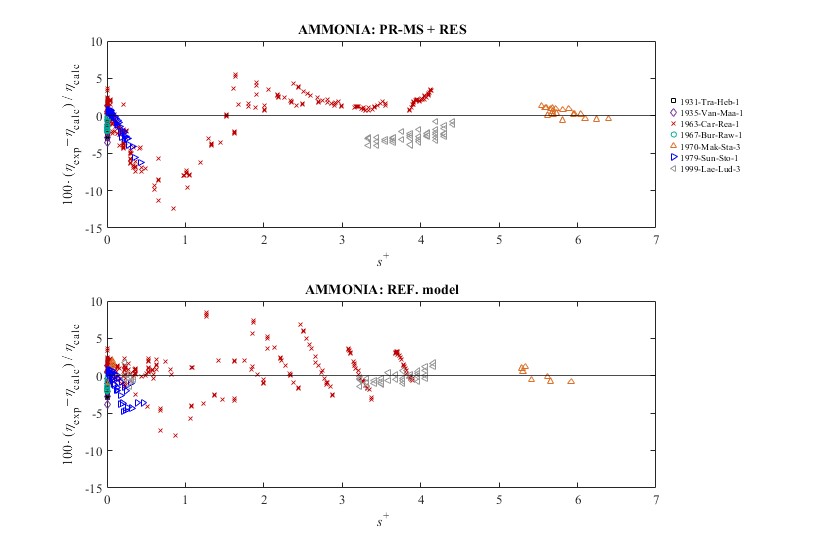

Supplement: Supplementary file 2 [file ao5c01157_si_002.zip › Supporting Information package 2/Figures/Deviation plots/PR-MS/AMMONIA.jpeg]

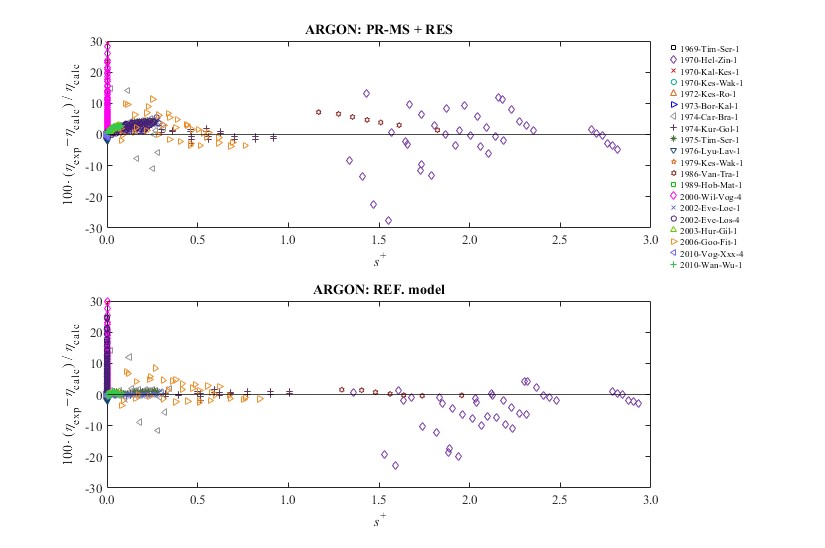

Supplement: Supplementary file 2 [file ao5c01157_si_002.zip › Supporting Information package 2/Figures/Deviation plots/PR-MS/ARGON.jpeg]

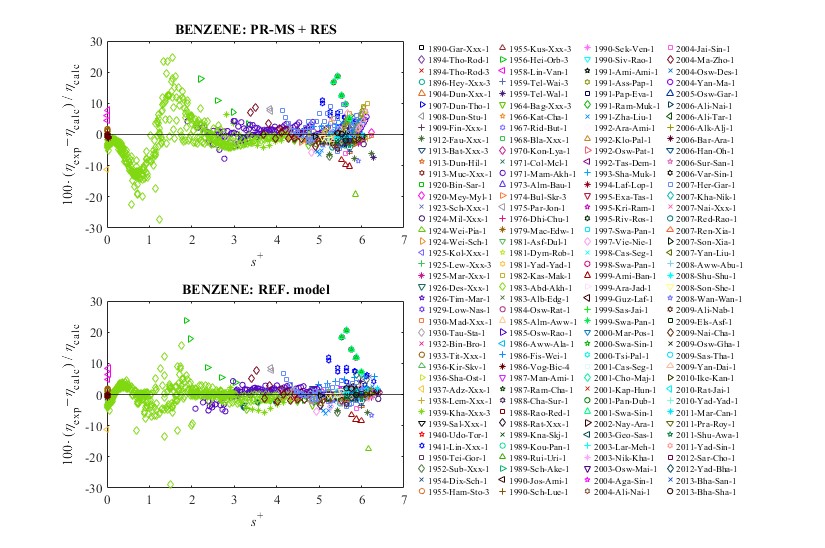

Supplement: Supplementary file 2 [file ao5c01157_si_002.zip › Supporting Information package 2/Figures/Deviation plots/PR-MS/BENZENE.jpeg]

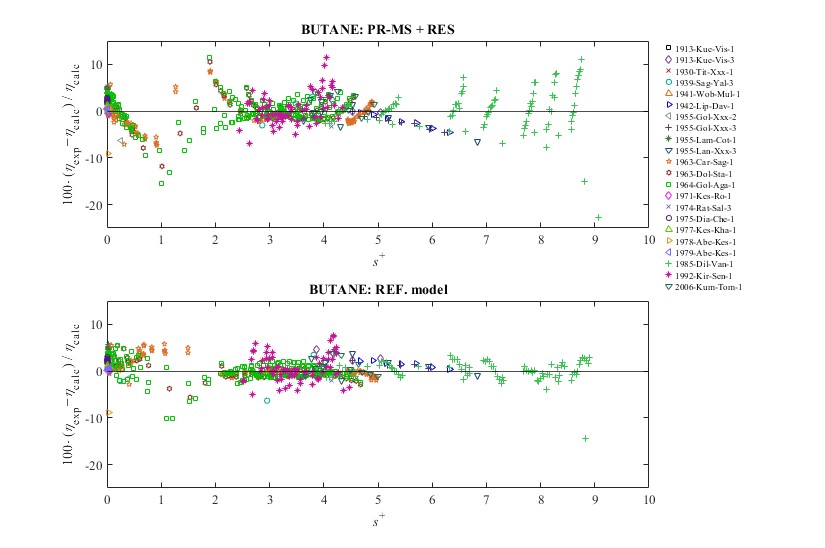

Supplement: Supplementary file 2 [file ao5c01157_si_002.zip › Supporting Information package 2/Figures/Deviation plots/PR-MS/BUTANE.jpeg]

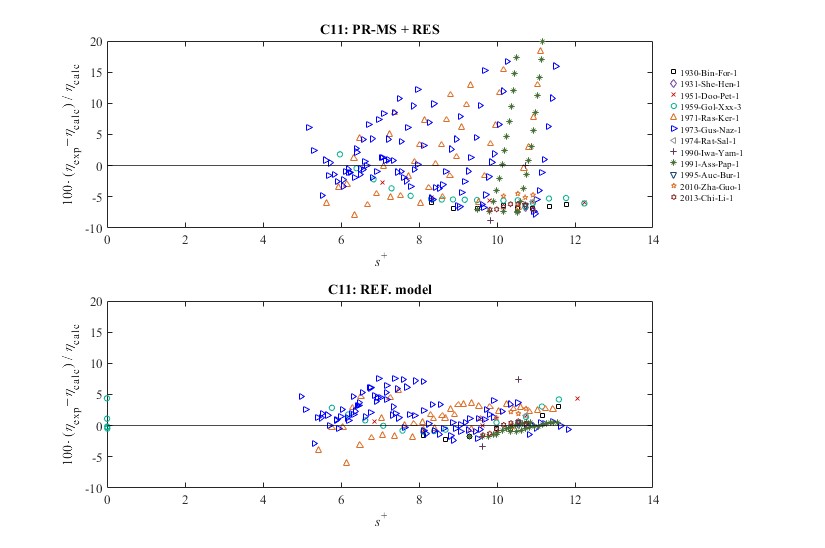

Supplement: Supplementary file 2 [file ao5c01157_si_002.zip › Supporting Information package 2/Figures/Deviation plots/PR-MS/C11.jpeg]

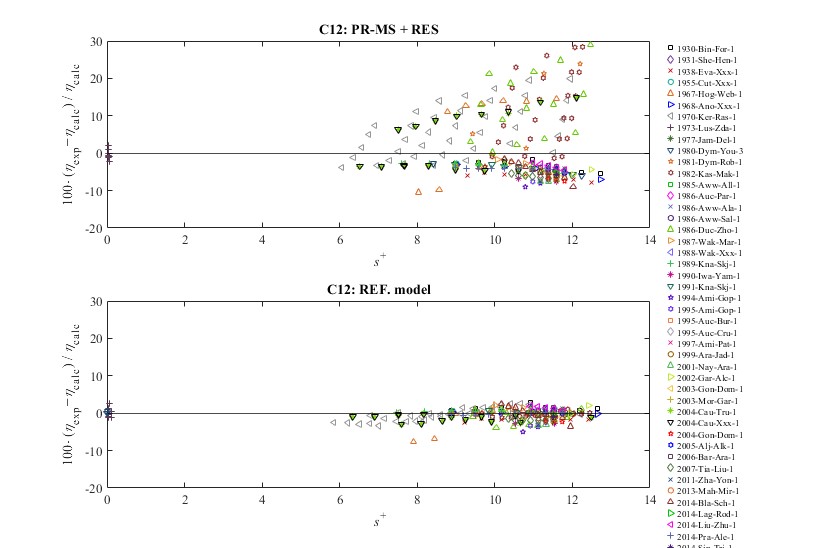

Supplement: Supplementary file 2 [file ao5c01157_si_002.zip › Supporting Information package 2/Figures/Deviation plots/PR-MS/C12.jpeg]

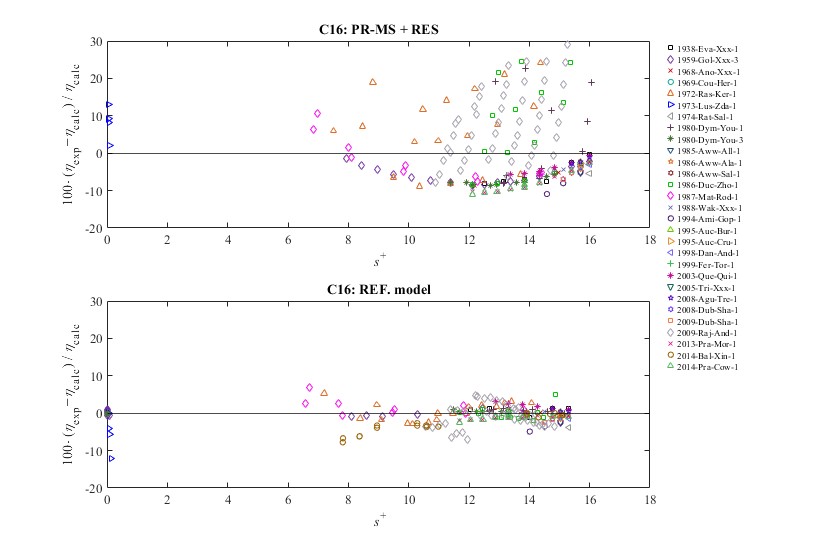

Supplement: Supplementary file 2 [file ao5c01157_si_002.zip › Supporting Information package 2/Figures/Deviation plots/PR-MS/C16.jpeg]

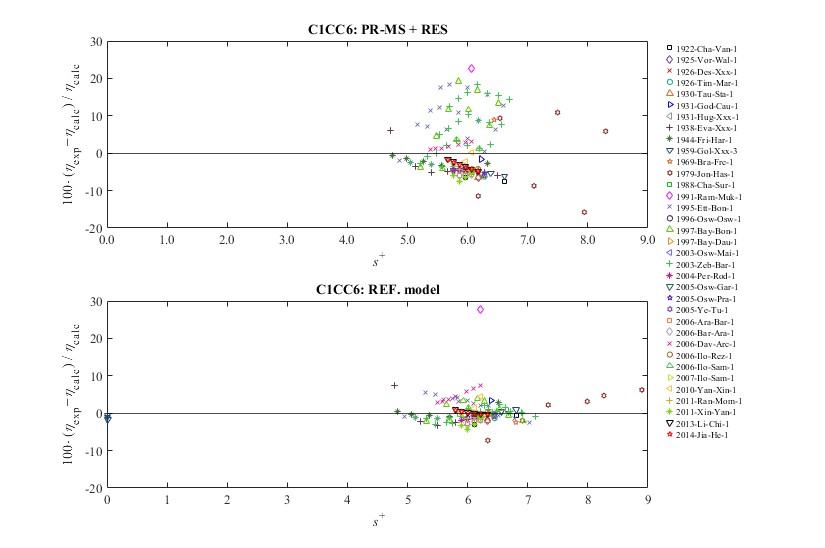

Supplement: Supplementary file 2 [file ao5c01157_si_002.zip › Supporting Information package 2/Figures/Deviation plots/PR-MS/C1CC6.jpeg]

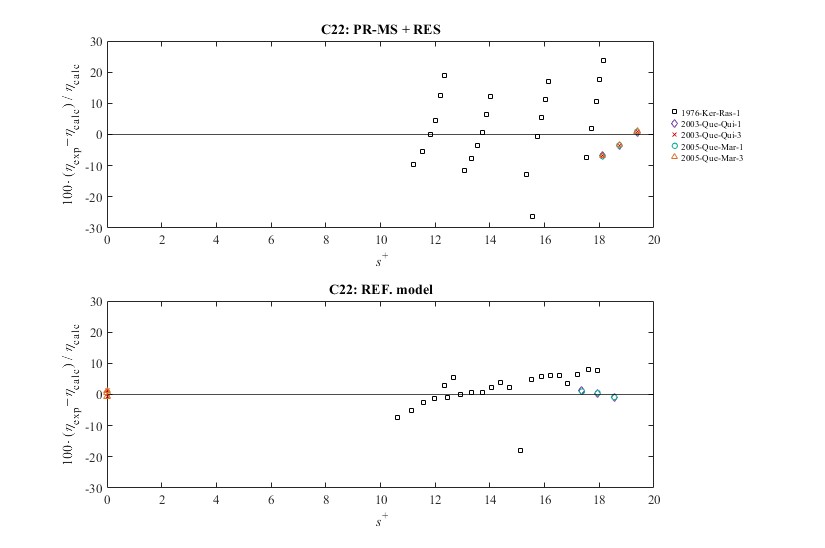

Supplement: Supplementary file 2 [file ao5c01157_si_002.zip › Supporting Information package 2/Figures/Deviation plots/PR-MS/C22.jpeg]

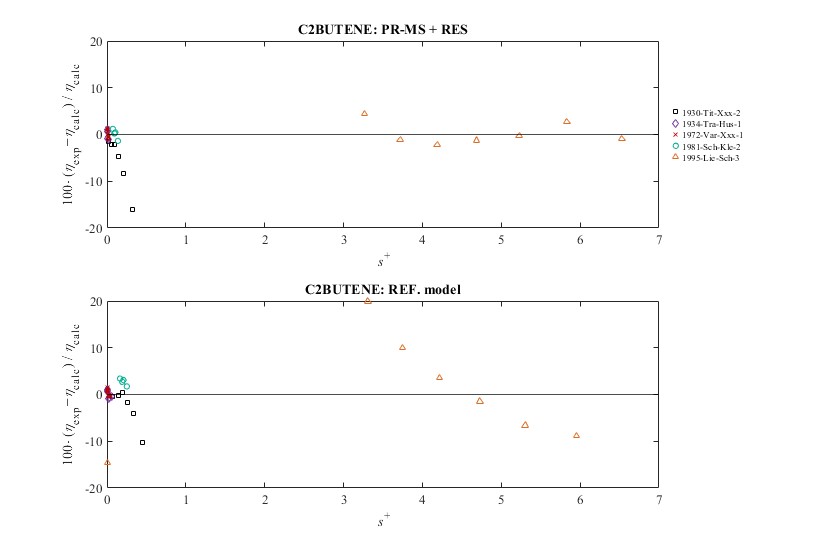

Supplement: Supplementary file 2 [file ao5c01157_si_002.zip › Supporting Information package 2/Figures/Deviation plots/PR-MS/C2BUTENE.jpeg]

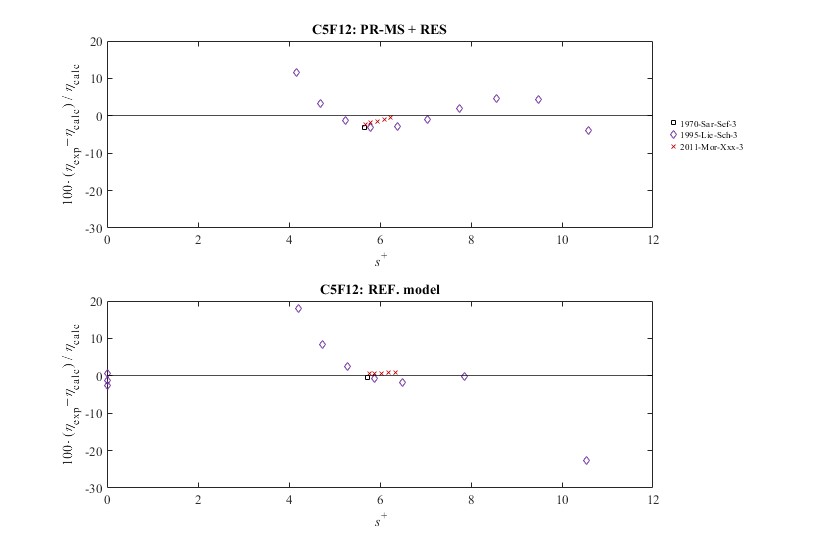

Supplement: Supplementary file 2 [file ao5c01157_si_002.zip › Supporting Information package 2/Figures/Deviation plots/PR-MS/C5F12.jpeg]

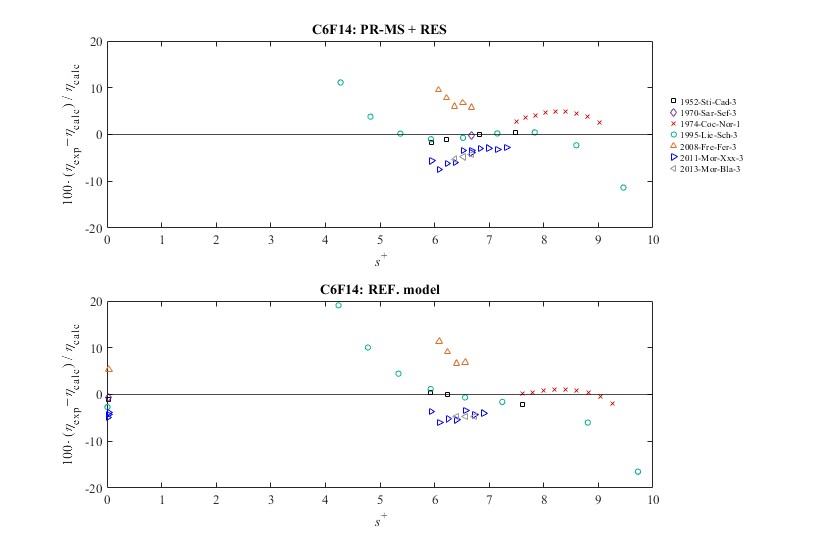

Supplement: Supplementary file 2 [file ao5c01157_si_002.zip › Supporting Information package 2/Figures/Deviation plots/PR-MS/C6F14.jpeg]

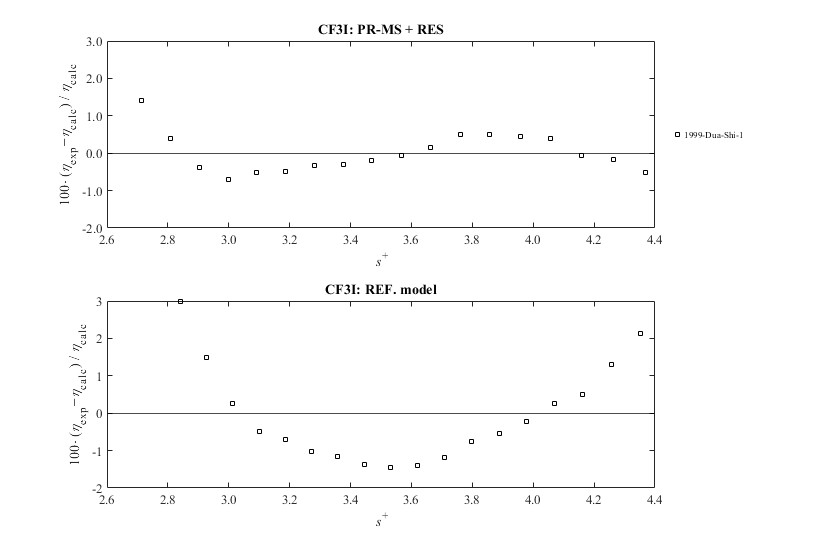

Supplement: Supplementary file 2 [file ao5c01157_si_002.zip › Supporting Information package 2/Figures/Deviation plots/PR-MS/CF3I.jpeg]

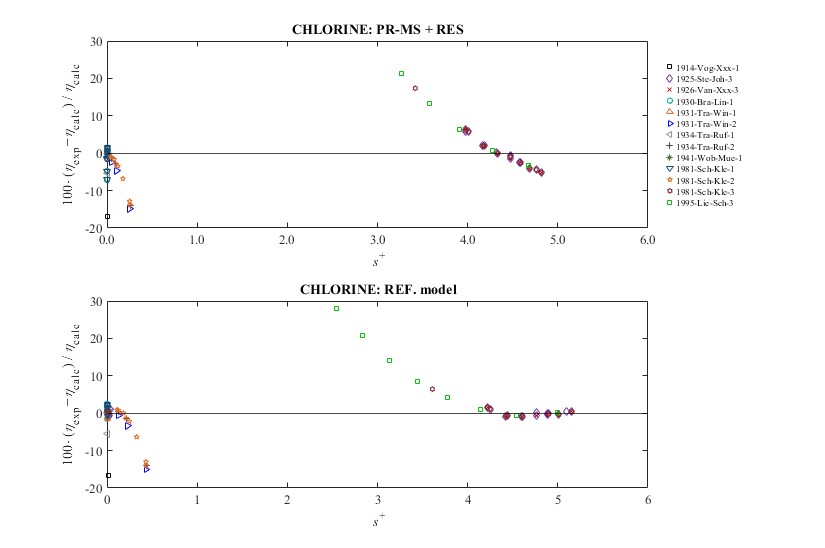

Supplement: Supplementary file 2 [file ao5c01157_si_002.zip › Supporting Information package 2/Figures/Deviation plots/PR-MS/CHLORINE.jpeg]

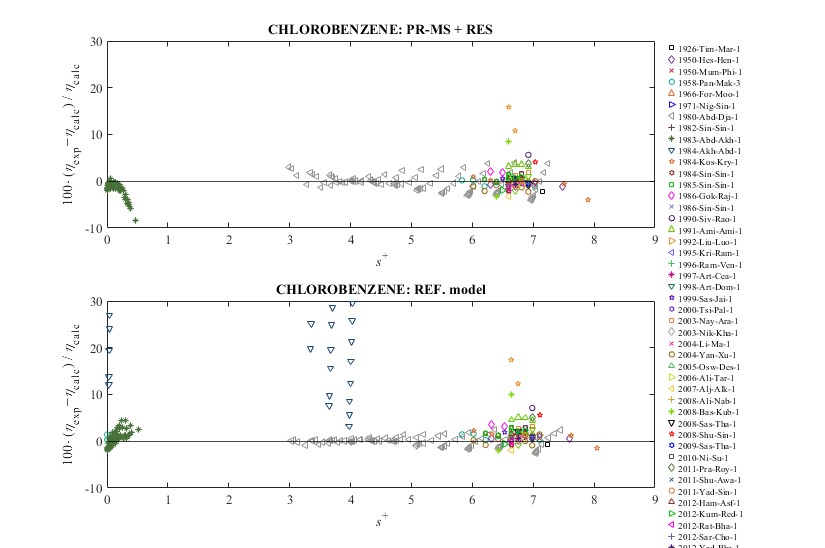

Supplement: Supplementary file 2 [file ao5c01157_si_002.zip › Supporting Information package 2/Figures/Deviation plots/PR-MS/CHLOROBENZENE.jpeg]

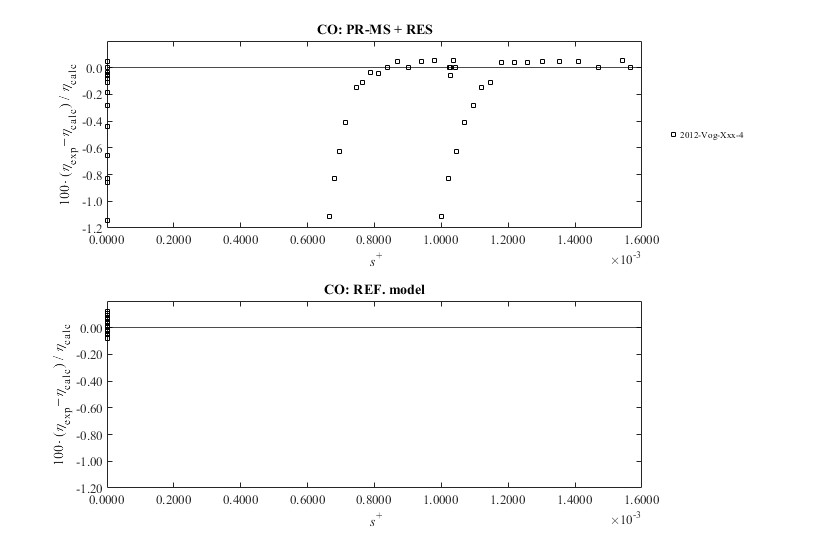

Supplement: Supplementary file 2 [file ao5c01157_si_002.zip › Supporting Information package 2/Figures/Deviation plots/PR-MS/CO.jpeg]

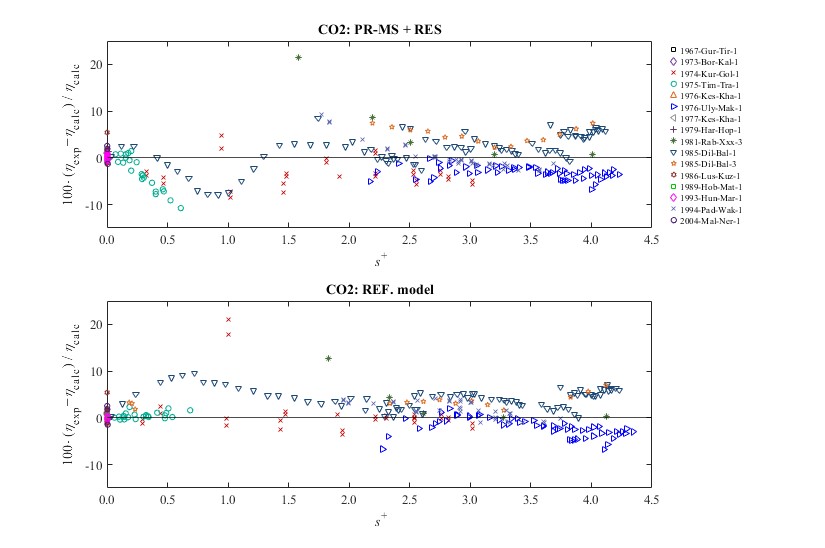

Supplement: Supplementary file 2 [file ao5c01157_si_002.zip › Supporting Information package 2/Figures/Deviation plots/PR-MS/CO2.jpeg]

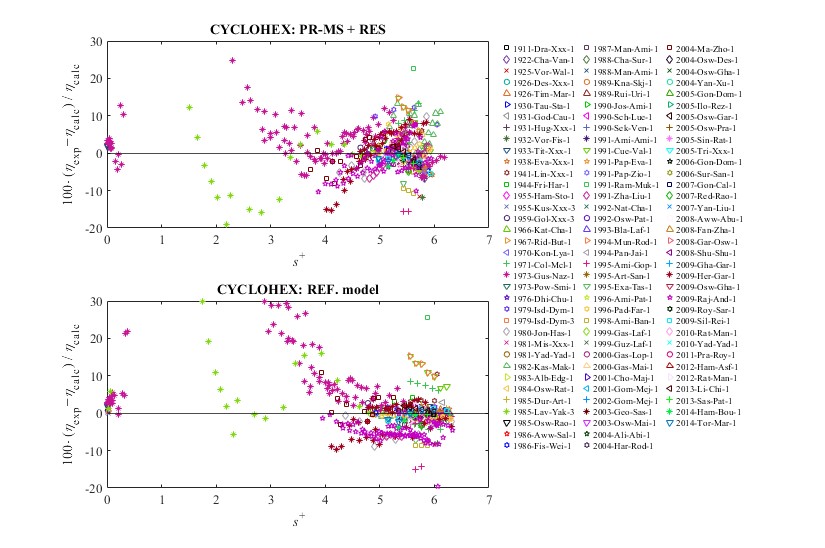

Supplement: Supplementary file 2 [file ao5c01157_si_002.zip › Supporting Information package 2/Figures/Deviation plots/PR-MS/CYCLOHEX.jpeg]

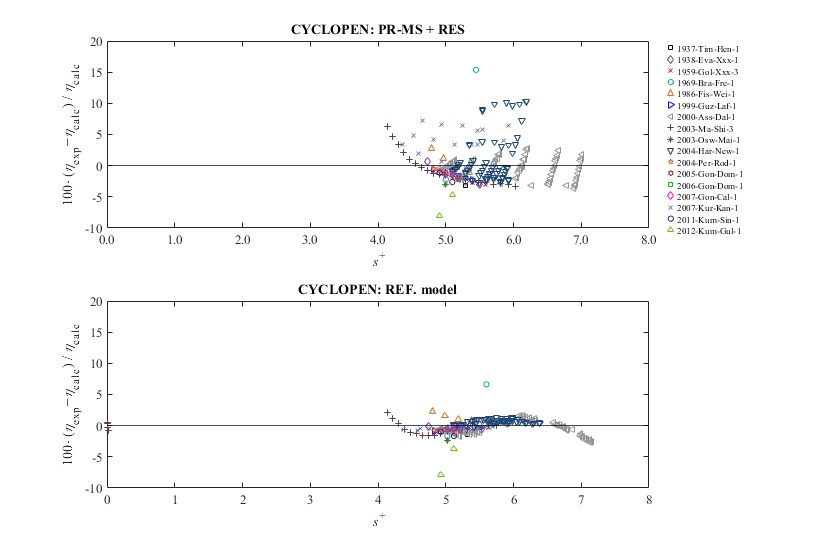

Supplement: Supplementary file 2 [file ao5c01157_si_002.zip › Supporting Information package 2/Figures/Deviation plots/PR-MS/CYCLOPEN.jpeg]

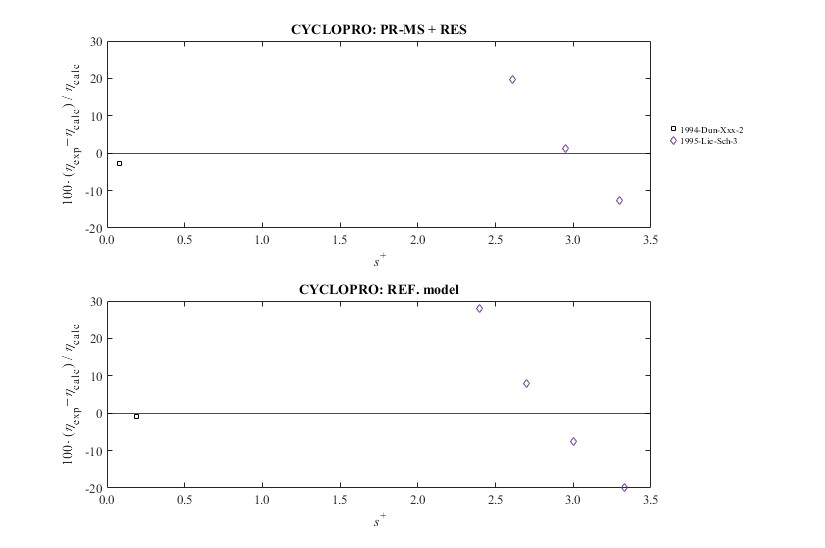

Supplement: Supplementary file 2 [file ao5c01157_si_002.zip › Supporting Information package 2/Figures/Deviation plots/PR-MS/CYCLOPRO.jpeg]

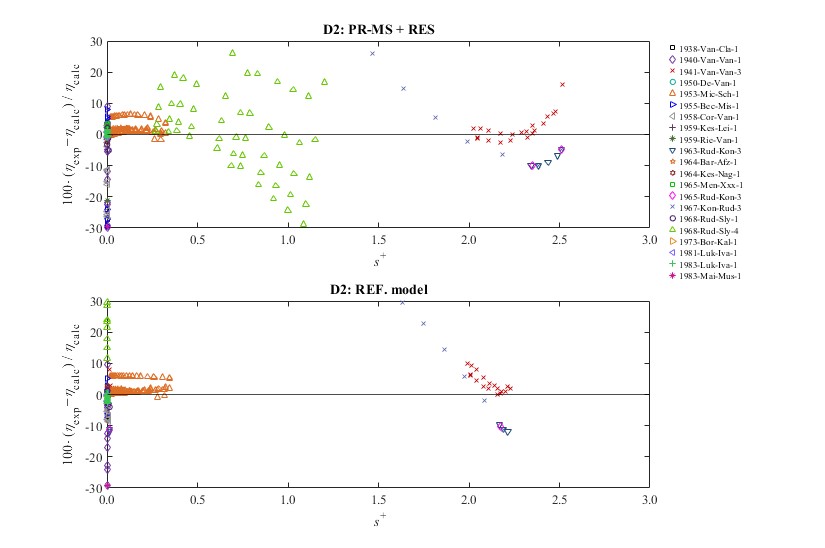

Supplement: Supplementary file 2 [file ao5c01157_si_002.zip › Supporting Information package 2/Figures/Deviation plots/PR-MS/D2.jpeg]

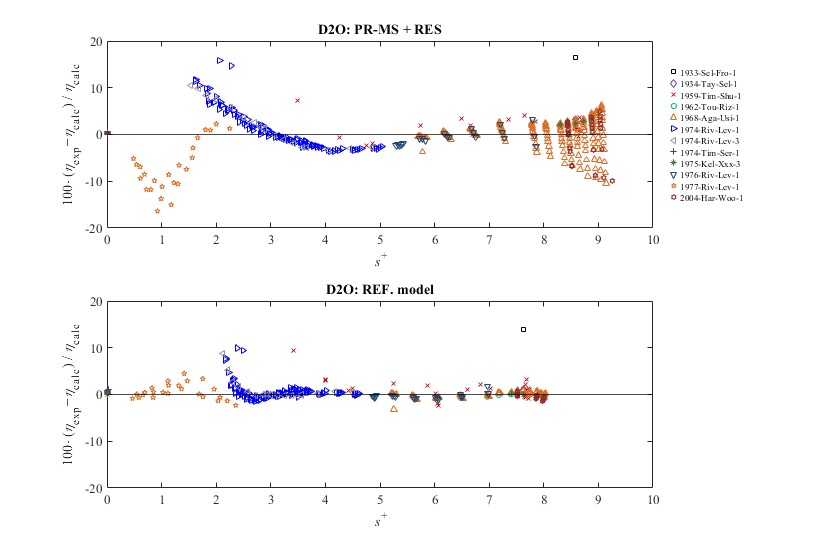

Supplement: Supplementary file 2 [file ao5c01157_si_002.zip › Supporting Information package 2/Figures/Deviation plots/PR-MS/D2O.jpeg]

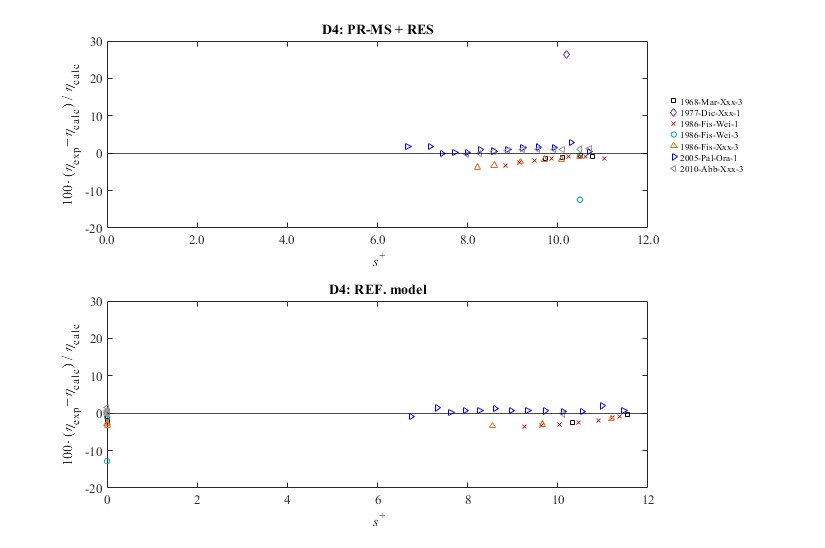

Supplement: Supplementary file 2 [file ao5c01157_si_002.zip › Supporting Information package 2/Figures/Deviation plots/PR-MS/D4.jpeg]

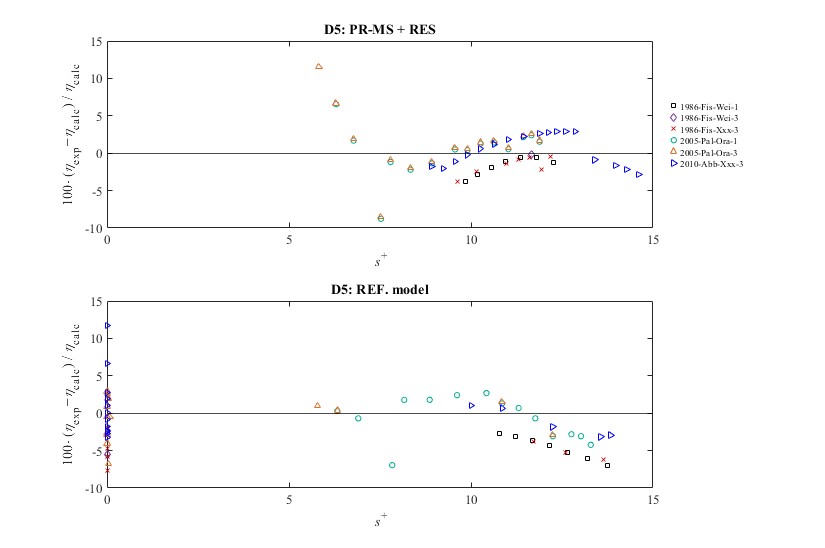

Supplement: Supplementary file 2 [file ao5c01157_si_002.zip › Supporting Information package 2/Figures/Deviation plots/PR-MS/D5.jpeg]

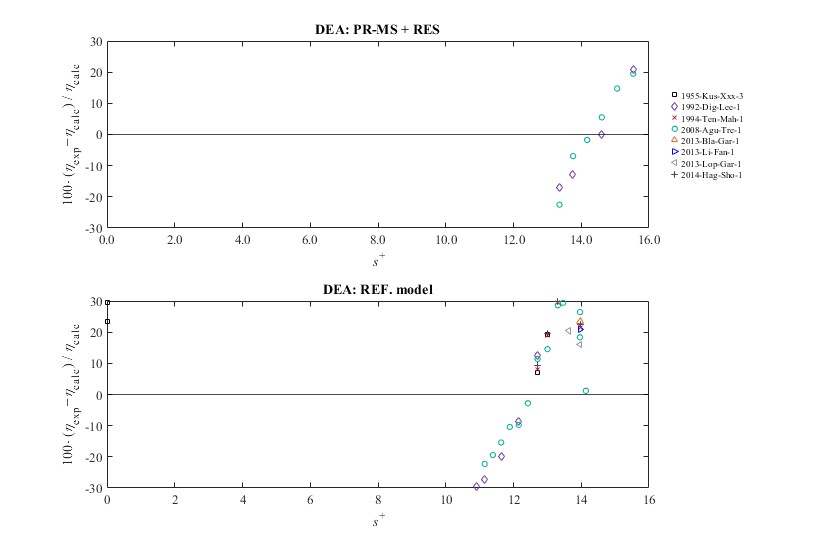

Supplement: Supplementary file 2 [file ao5c01157_si_002.zip › Supporting Information package 2/Figures/Deviation plots/PR-MS/DEA.jpeg]

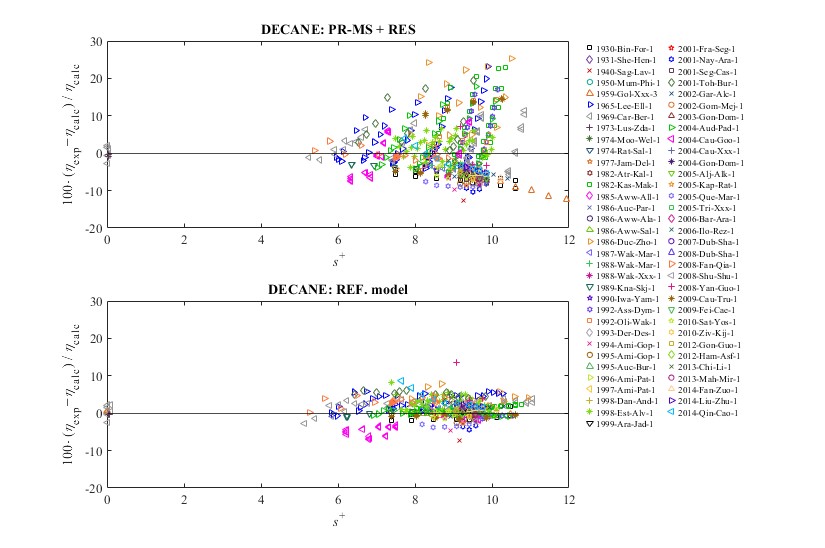

Supplement: Supplementary file 2 [file ao5c01157_si_002.zip › Supporting Information package 2/Figures/Deviation plots/PR-MS/DECANE.jpeg]

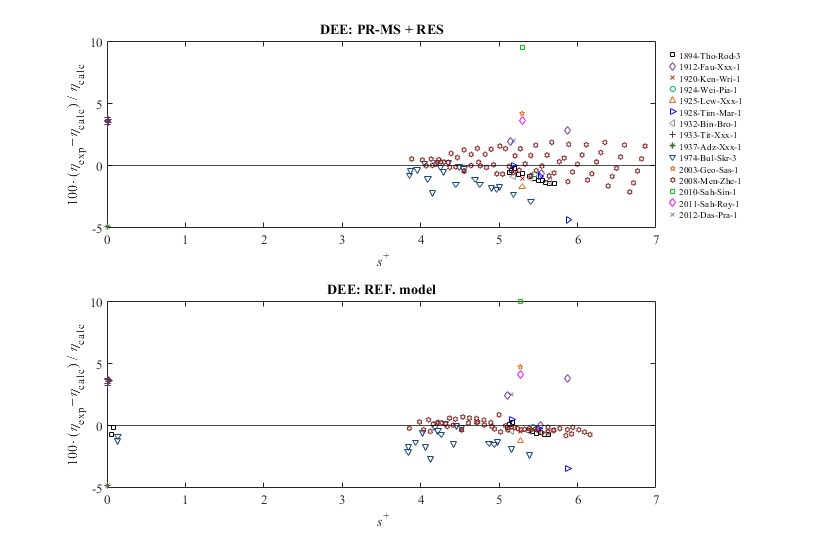

Supplement: Supplementary file 2 [file ao5c01157_si_002.zip › Supporting Information package 2/Figures/Deviation plots/PR-MS/DEE.jpeg]

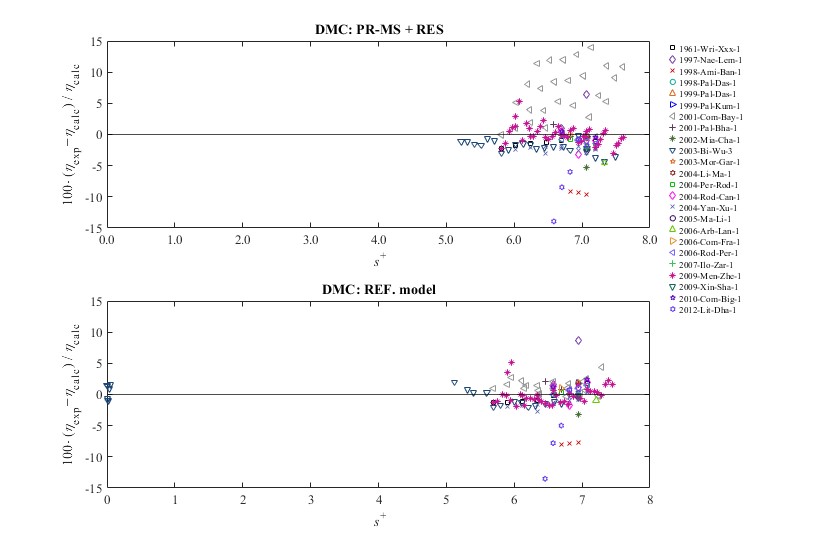

Supplement: Supplementary file 2 [file ao5c01157_si_002.zip › Supporting Information package 2/Figures/Deviation plots/PR-MS/DMC.jpeg]

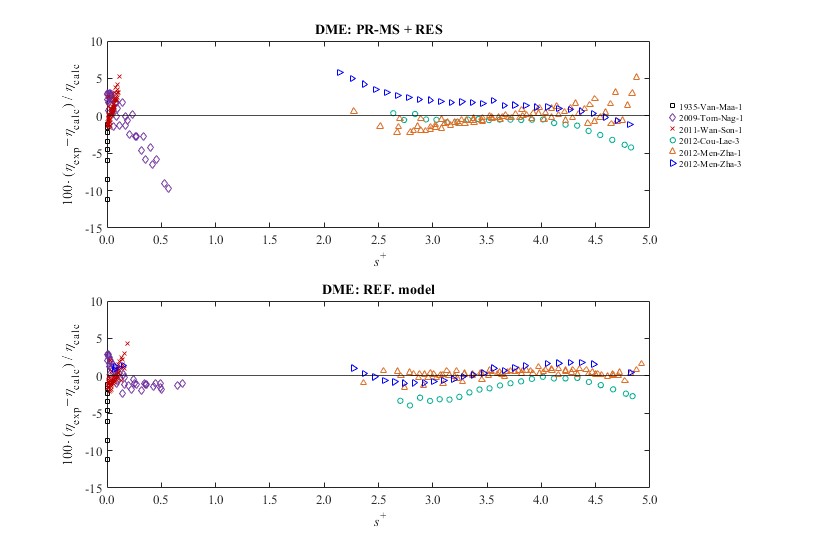

Supplement: Supplementary file 2 [file ao5c01157_si_002.zip › Supporting Information package 2/Figures/Deviation plots/PR-MS/DME.jpeg]

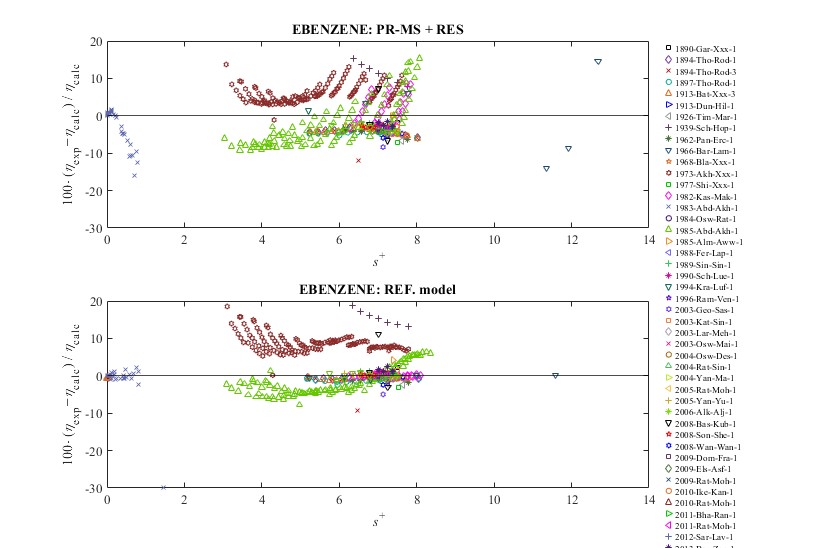

Supplement: Supplementary file 2 [file ao5c01157_si_002.zip › Supporting Information package 2/Figures/Deviation plots/PR-MS/EBENZENE.jpeg]

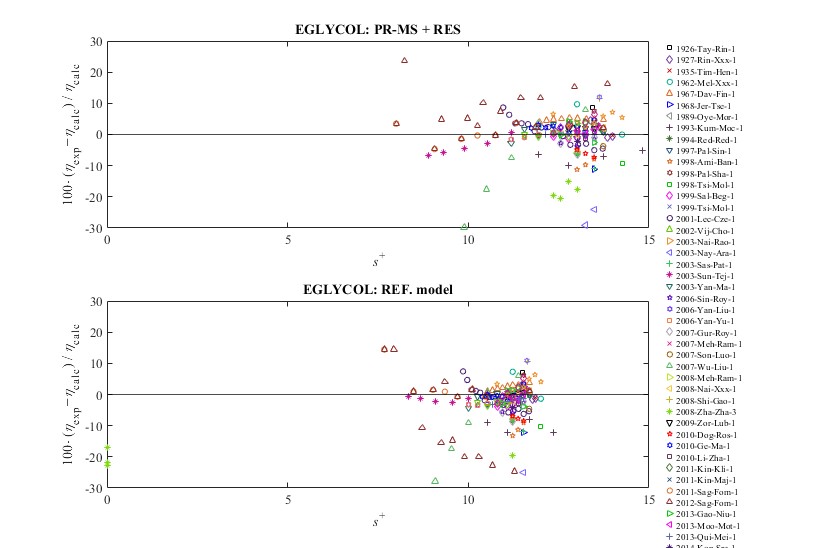

Supplement: Supplementary file 2 [file ao5c01157_si_002.zip › Supporting Information package 2/Figures/Deviation plots/PR-MS/EGLYCOL.jpeg]

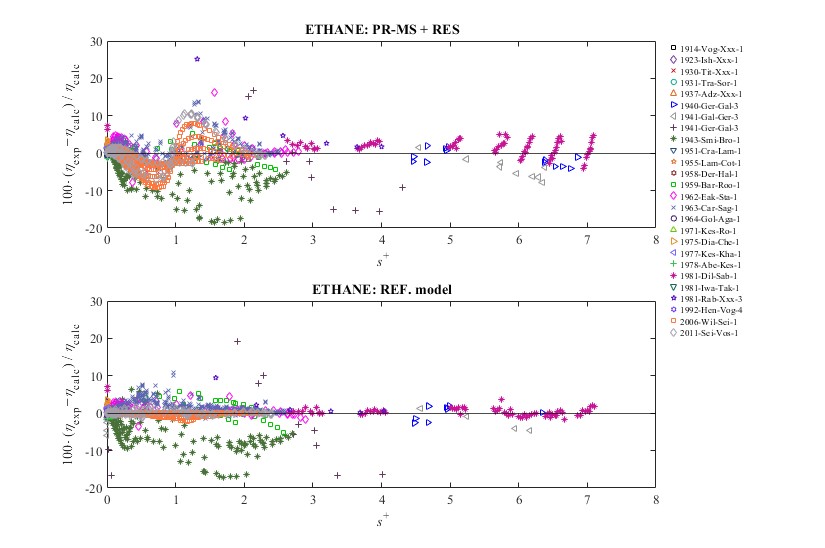

Supplement: Supplementary file 2 [file ao5c01157_si_002.zip › Supporting Information package 2/Figures/Deviation plots/PR-MS/ETHANE.jpeg]

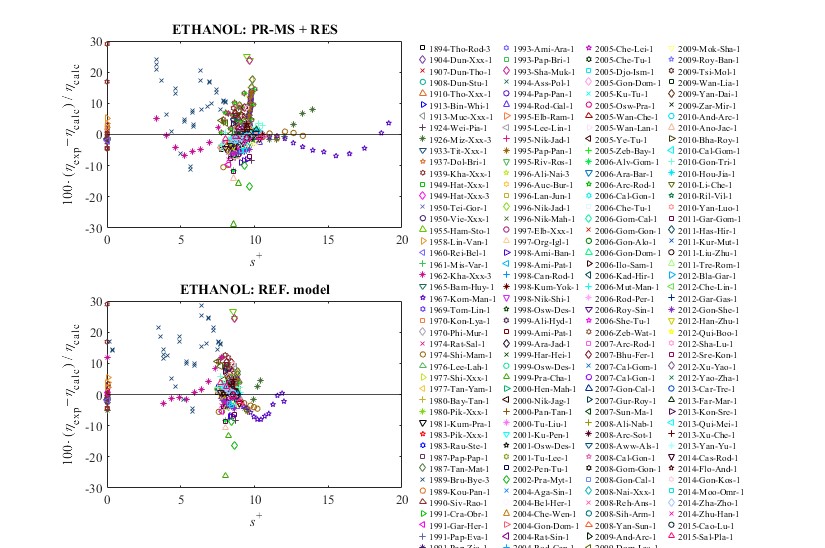

Supplement: Supplementary file 2 [file ao5c01157_si_002.zip › Supporting Information package 2/Figures/Deviation plots/PR-MS/ETHANOL.jpeg]

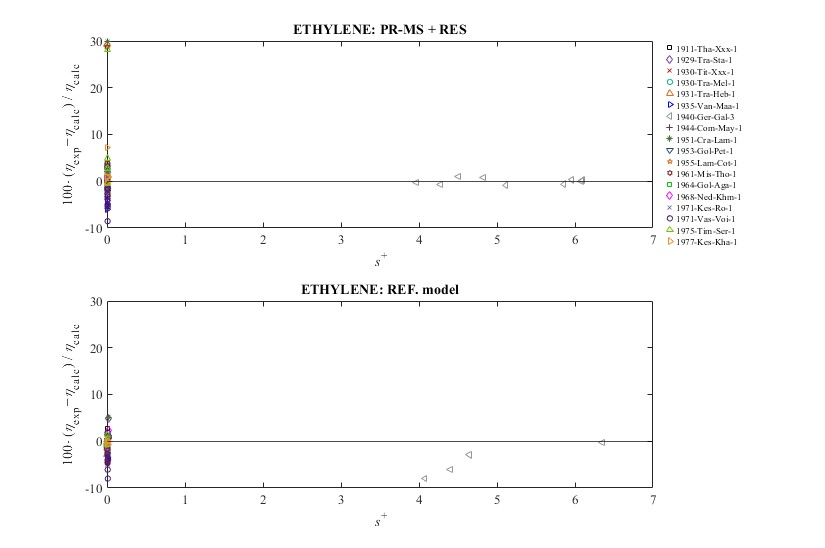

Supplement: Supplementary file 2 [file ao5c01157_si_002.zip › Supporting Information package 2/Figures/Deviation plots/PR-MS/ETHYLENE.jpeg]

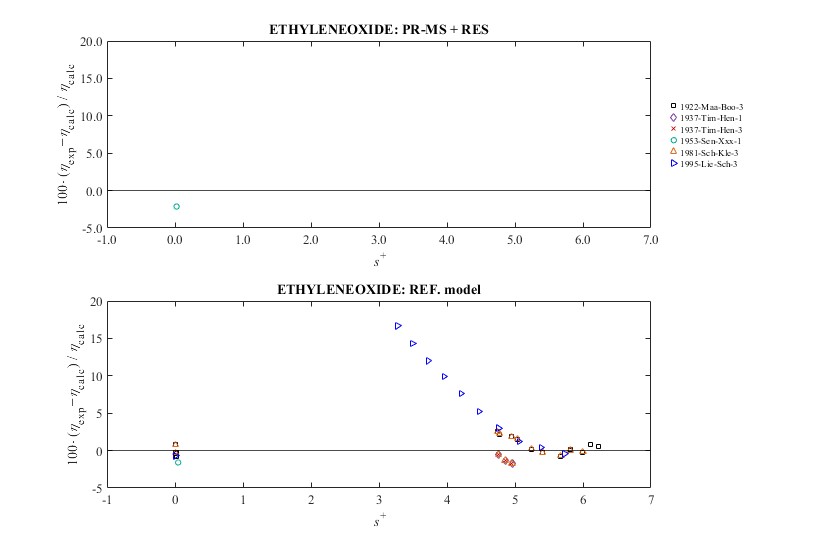

Supplement: Supplementary file 2 [file ao5c01157_si_002.zip › Supporting Information package 2/Figures/Deviation plots/PR-MS/ETHYLENEOXIDE.jpeg]

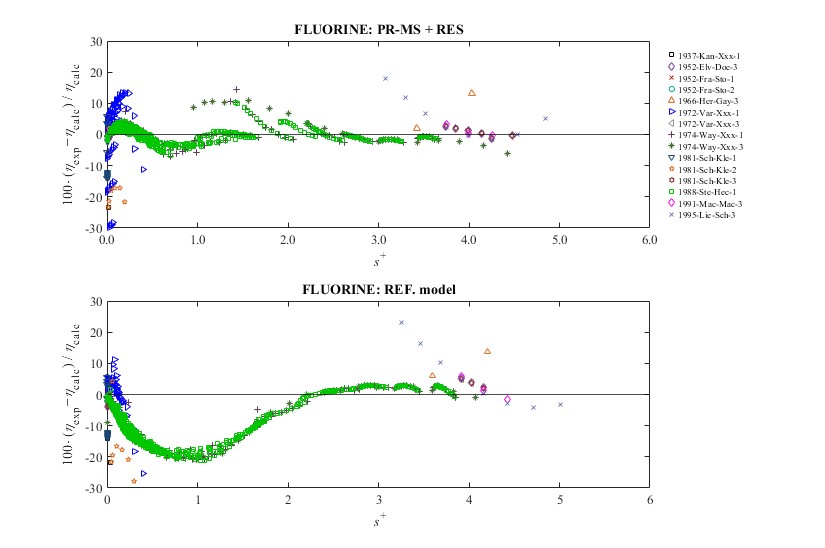

Supplement: Supplementary file 2 [file ao5c01157_si_002.zip › Supporting Information package 2/Figures/Deviation plots/PR-MS/FLUORINE.jpeg]

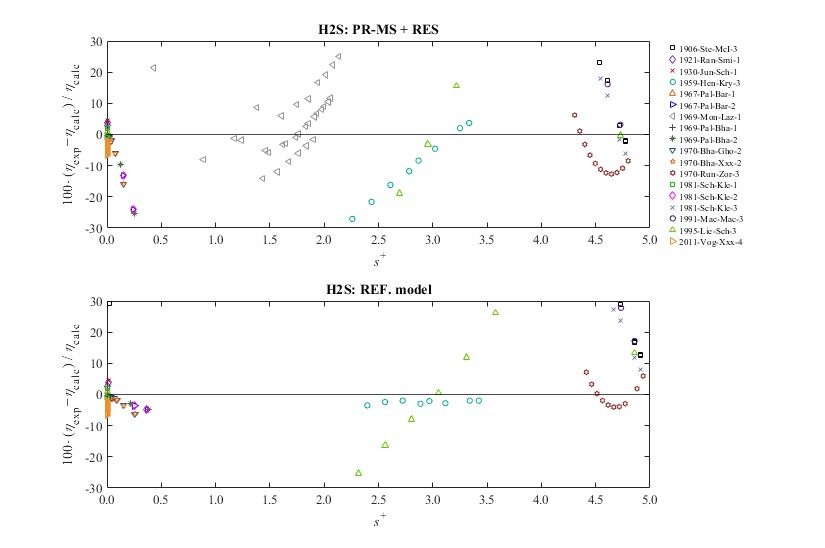

Supplement: Supplementary file 2 [file ao5c01157_si_002.zip › Supporting Information package 2/Figures/Deviation plots/PR-MS/H2S.jpeg]

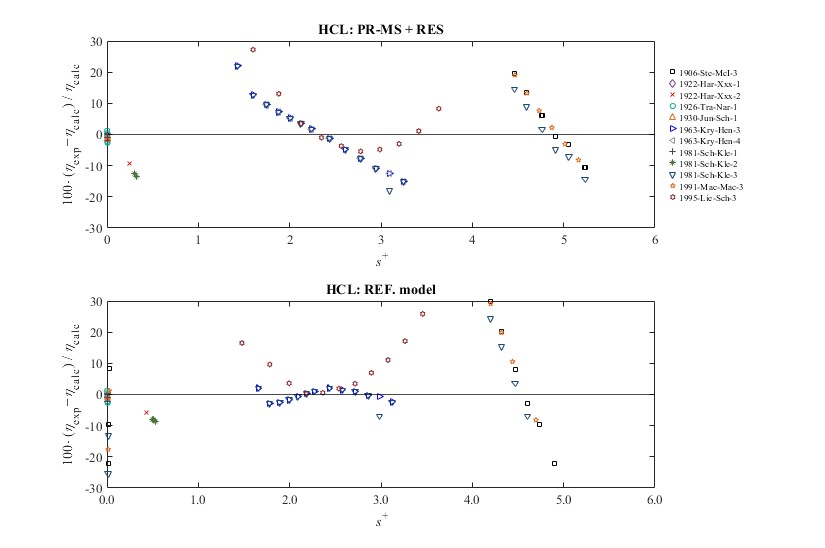

Supplement: Supplementary file 2 [file ao5c01157_si_002.zip › Supporting Information package 2/Figures/Deviation plots/PR-MS/HCL.jpeg]

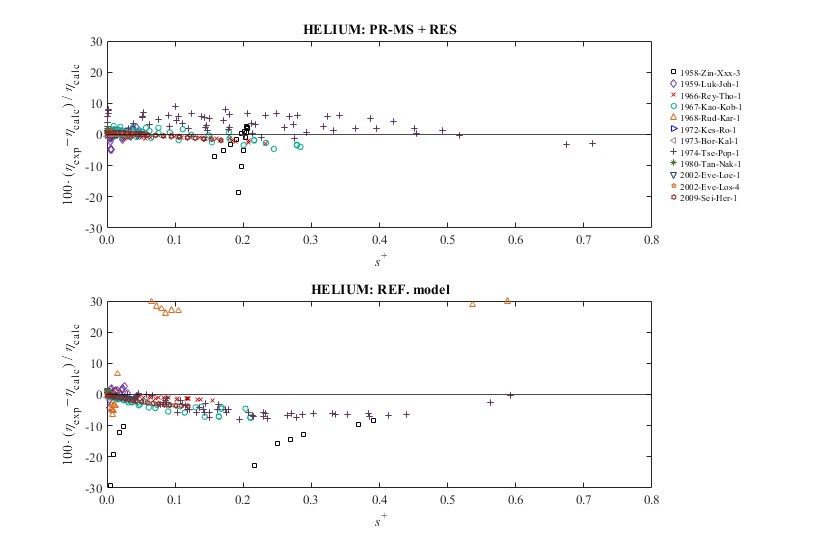

Supplement: Supplementary file 2 [file ao5c01157_si_002.zip › Supporting Information package 2/Figures/Deviation plots/PR-MS/HELIUM.jpeg]

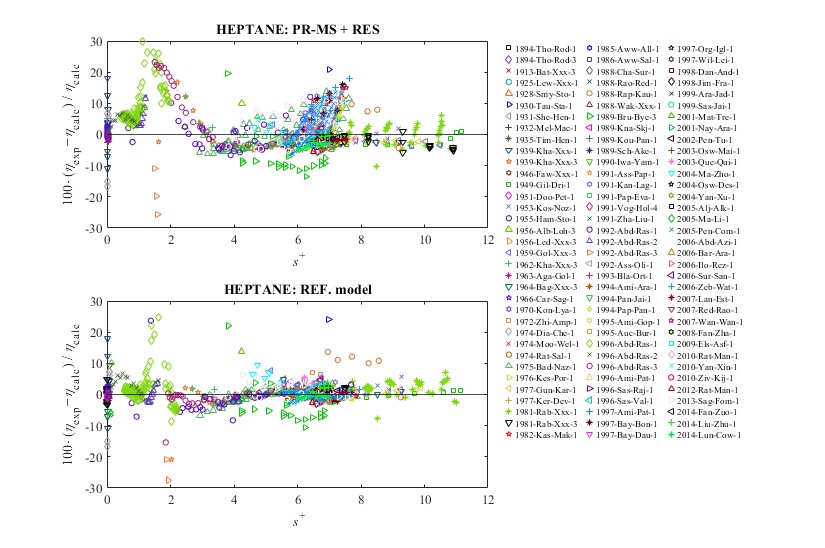

Supplement: Supplementary file 2 [file ao5c01157_si_002.zip › Supporting Information package 2/Figures/Deviation plots/PR-MS/HEPTANE.jpeg]

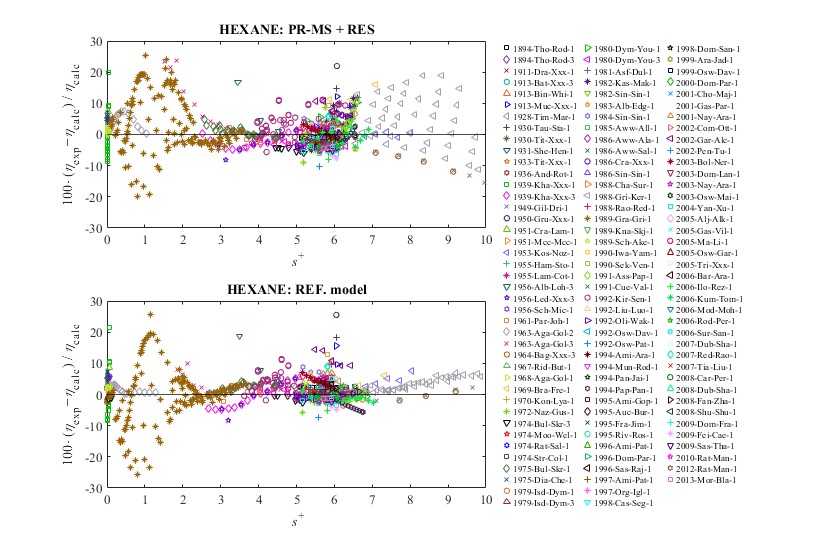

Supplement: Supplementary file 2 [file ao5c01157_si_002.zip › Supporting Information package 2/Figures/Deviation plots/PR-MS/HEXANE.jpeg]

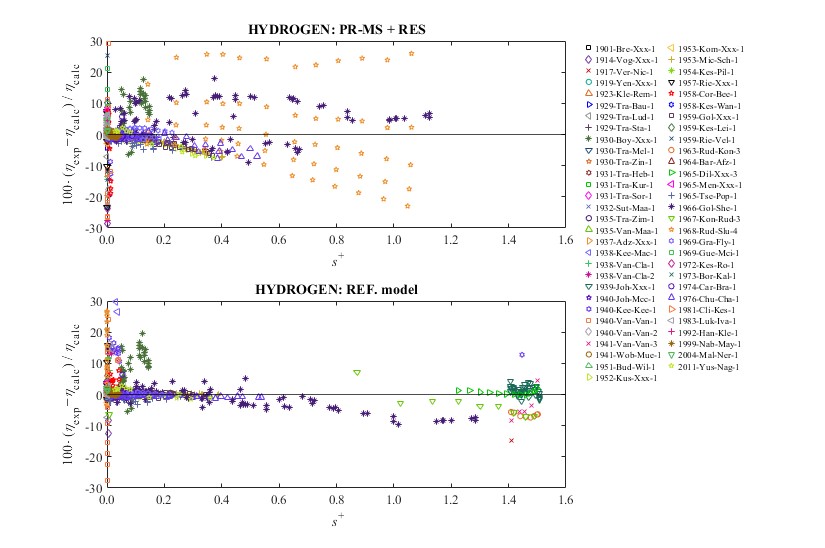

Supplement: Supplementary file 2 [file ao5c01157_si_002.zip › Supporting Information package 2/Figures/Deviation plots/PR-MS/HYDROGEN.jpeg]

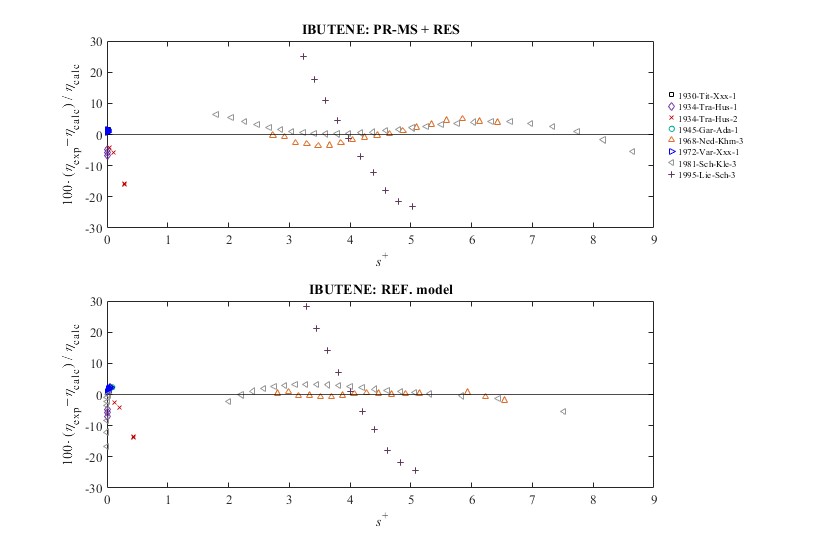

Supplement: Supplementary file 2 [file ao5c01157_si_002.zip › Supporting Information package 2/Figures/Deviation plots/PR-MS/IBUTENE.jpeg]

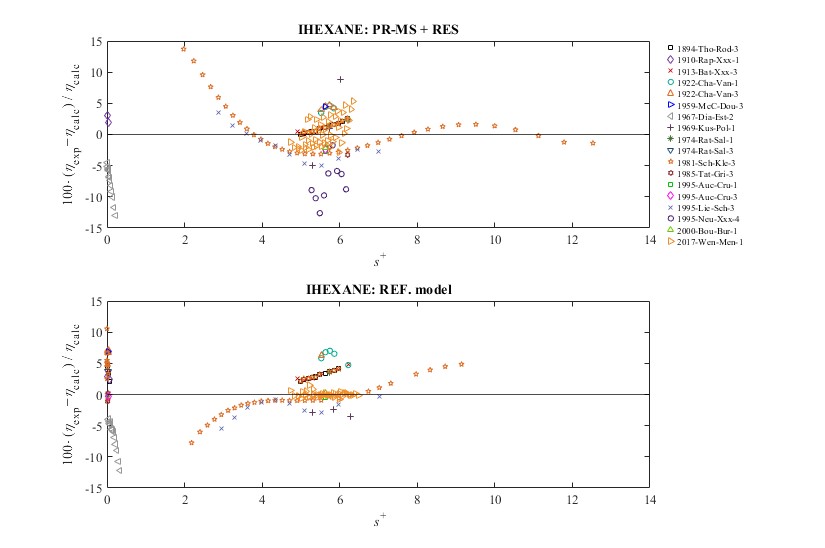

Supplement: Supplementary file 2 [file ao5c01157_si_002.zip › Supporting Information package 2/Figures/Deviation plots/PR-MS/IHEXANE.jpeg]

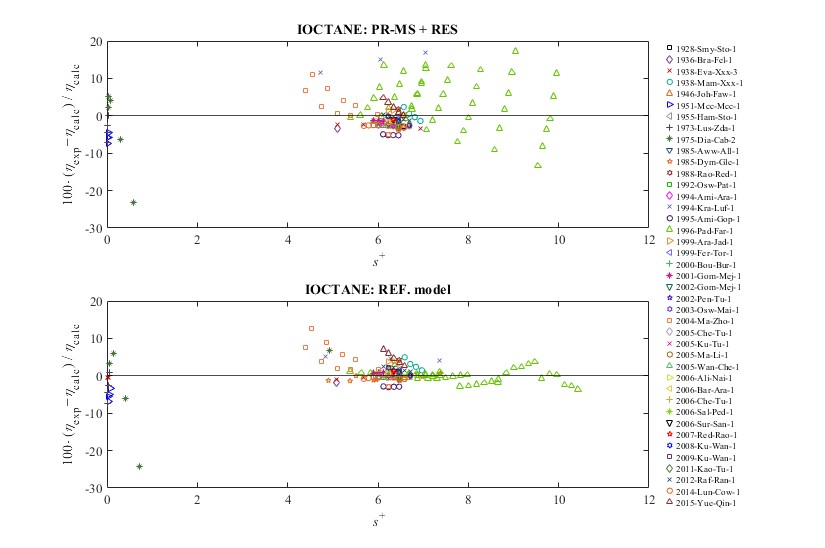

Supplement: Supplementary file 2 [file ao5c01157_si_002.zip › Supporting Information package 2/Figures/Deviation plots/PR-MS/IOCTANE.jpeg]

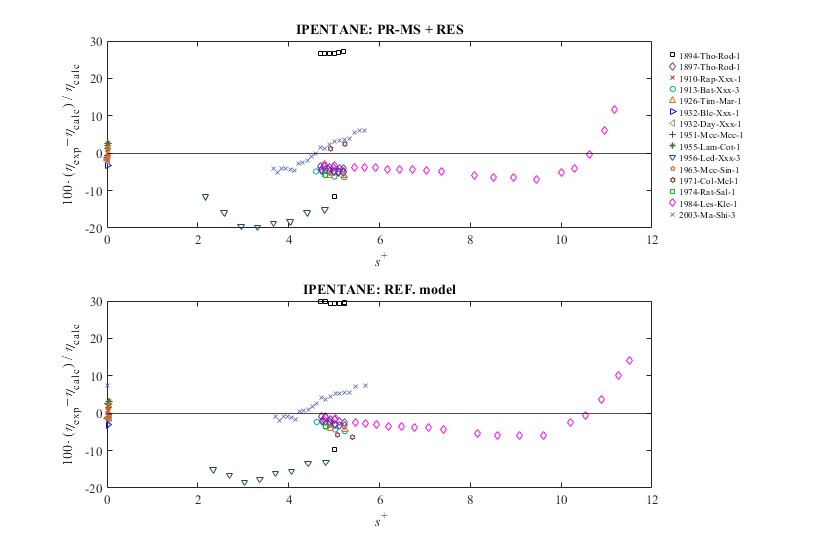

Supplement: Supplementary file 2 [file ao5c01157_si_002.zip › Supporting Information package 2/Figures/Deviation plots/PR-MS/IPENTANE.jpeg]

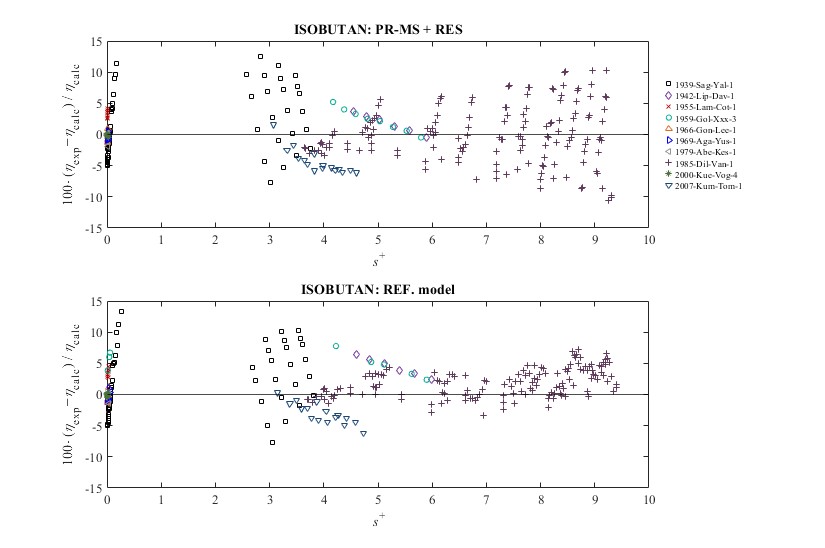

Supplement: Supplementary file 2 [file ao5c01157_si_002.zip › Supporting Information package 2/Figures/Deviation plots/PR-MS/ISOBUTAN.jpeg]

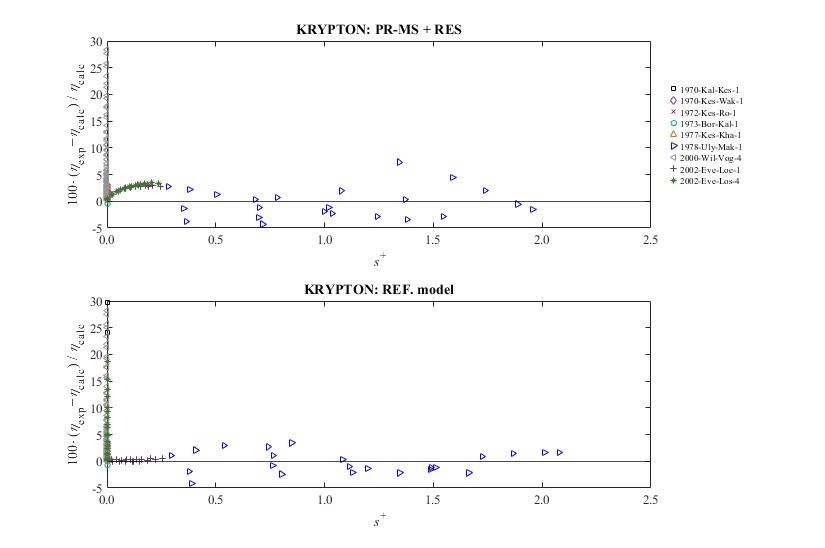

Supplement: Supplementary file 2 [file ao5c01157_si_002.zip › Supporting Information package 2/Figures/Deviation plots/PR-MS/KRYPTON.jpeg]

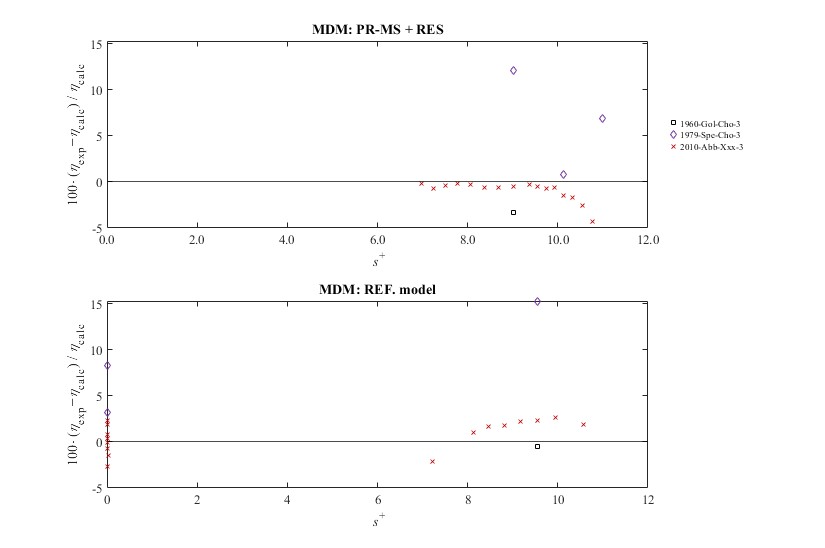

Supplement: Supplementary file 2 [file ao5c01157_si_002.zip › Supporting Information package 2/Figures/Deviation plots/PR-MS/MDM.jpeg]

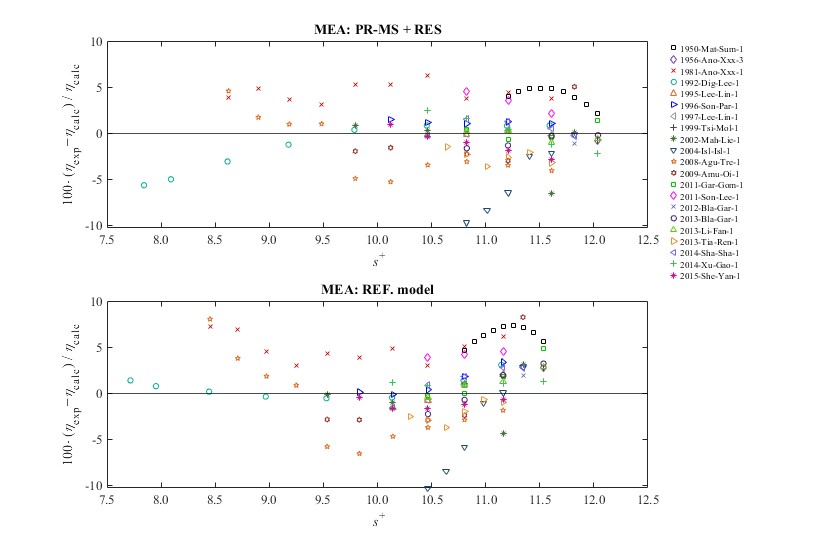

Supplement: Supplementary file 2 [file ao5c01157_si_002.zip › Supporting Information package 2/Figures/Deviation plots/PR-MS/MEA.jpeg]

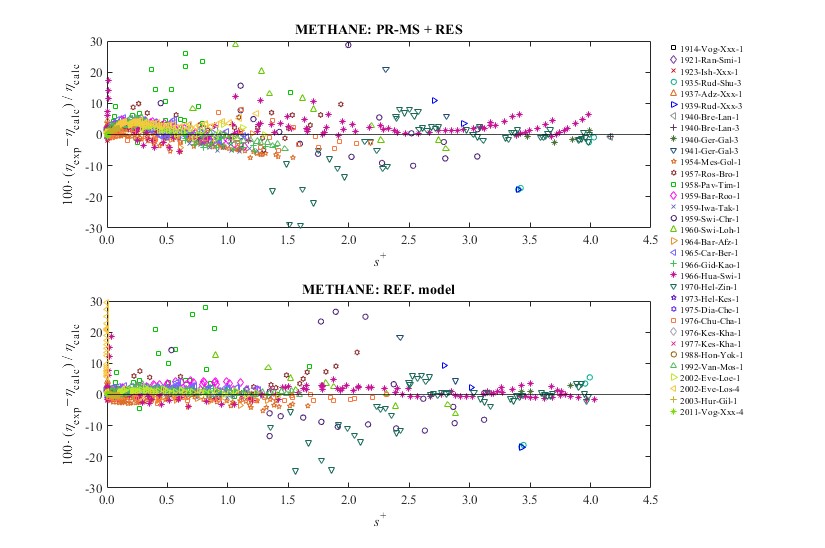

Supplement: Supplementary file 2 [file ao5c01157_si_002.zip › Supporting Information package 2/Figures/Deviation plots/PR-MS/METHANE.jpeg]

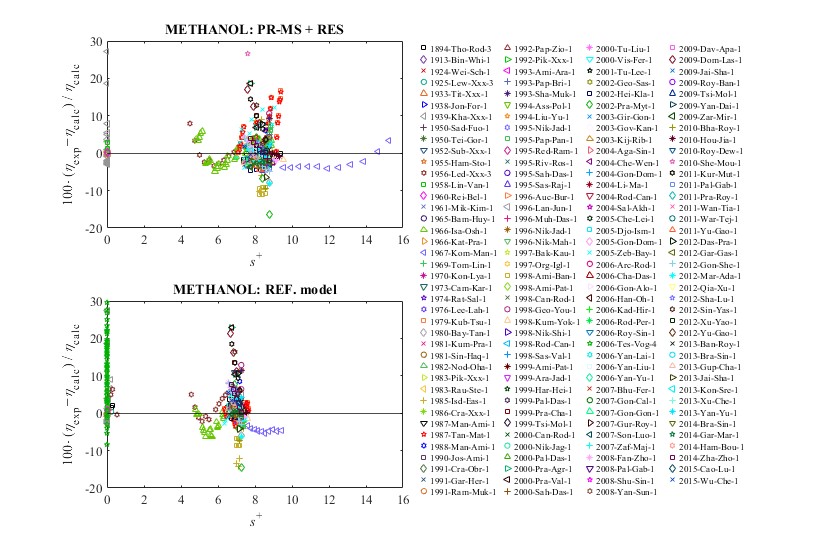

Supplement: Supplementary file 2 [file ao5c01157_si_002.zip › Supporting Information package 2/Figures/Deviation plots/PR-MS/METHANOL.jpeg]

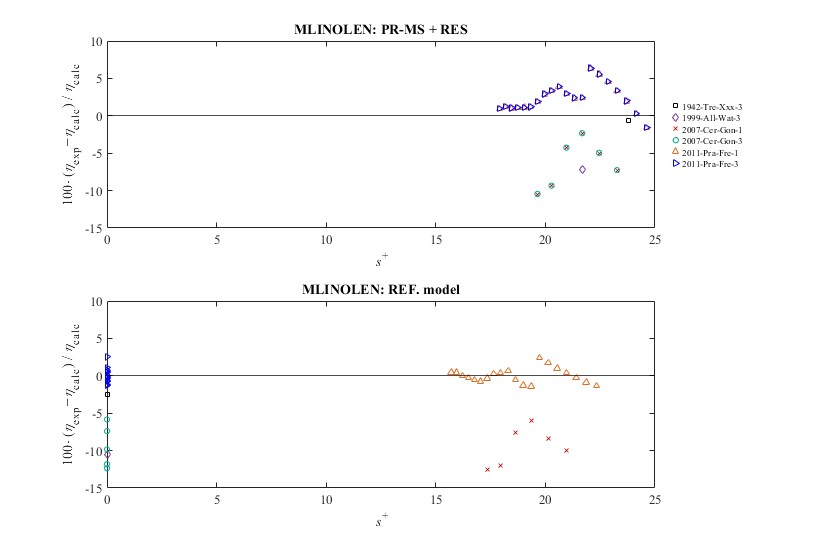

Supplement: Supplementary file 2 [file ao5c01157_si_002.zip › Supporting Information package 2/Figures/Deviation plots/PR-MS/MLINOLEN.jpeg]

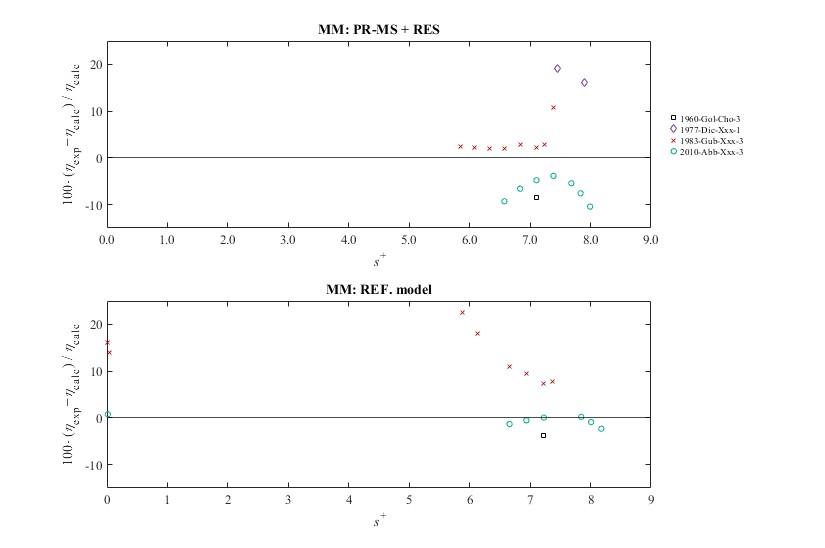

Supplement: Supplementary file 2 [file ao5c01157_si_002.zip › Supporting Information package 2/Figures/Deviation plots/PR-MS/MM.jpeg]

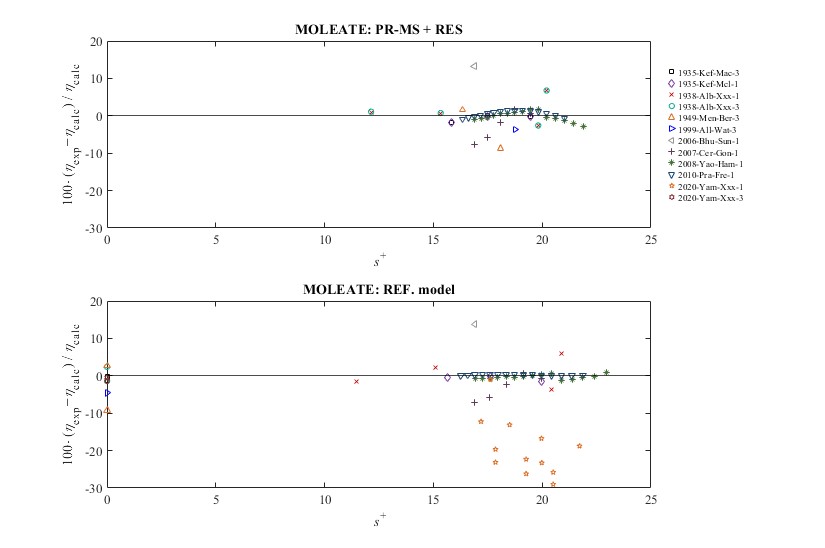

Supplement: Supplementary file 2 [file ao5c01157_si_002.zip › Supporting Information package 2/Figures/Deviation plots/PR-MS/MOLEATE.jpeg]

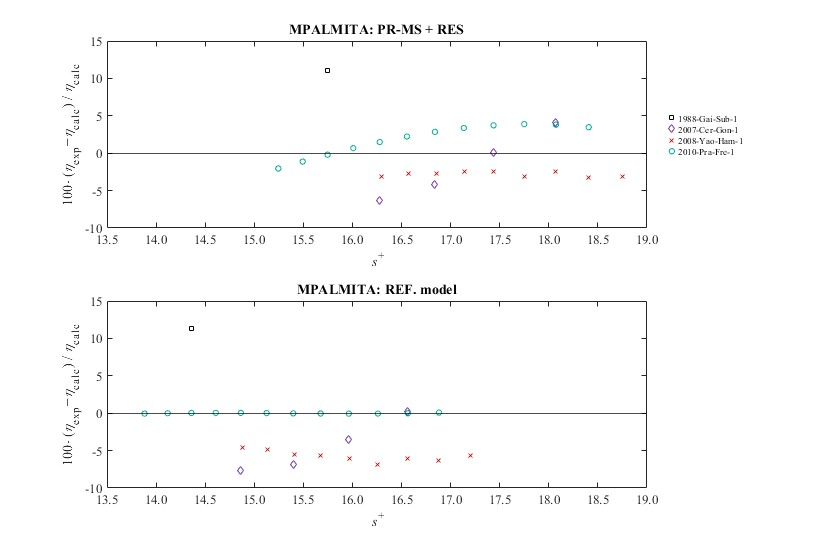

Supplement: Supplementary file 2 [file ao5c01157_si_002.zip › Supporting Information package 2/Figures/Deviation plots/PR-MS/MPALMITA.jpeg]

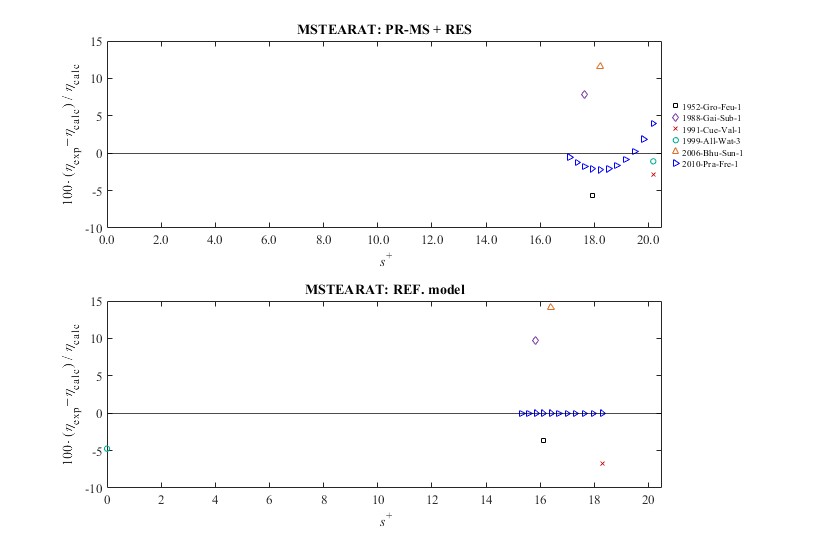

Supplement: Supplementary file 2 [file ao5c01157_si_002.zip › Supporting Information package 2/Figures/Deviation plots/PR-MS/MSTEARAT.jpeg]

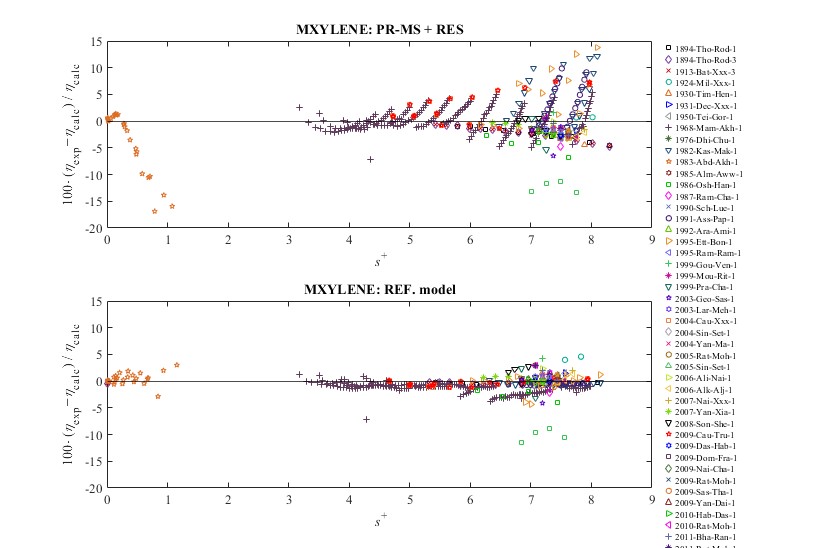

Supplement: Supplementary file 2 [file ao5c01157_si_002.zip › Supporting Information package 2/Figures/Deviation plots/PR-MS/MXYLENE.jpeg]

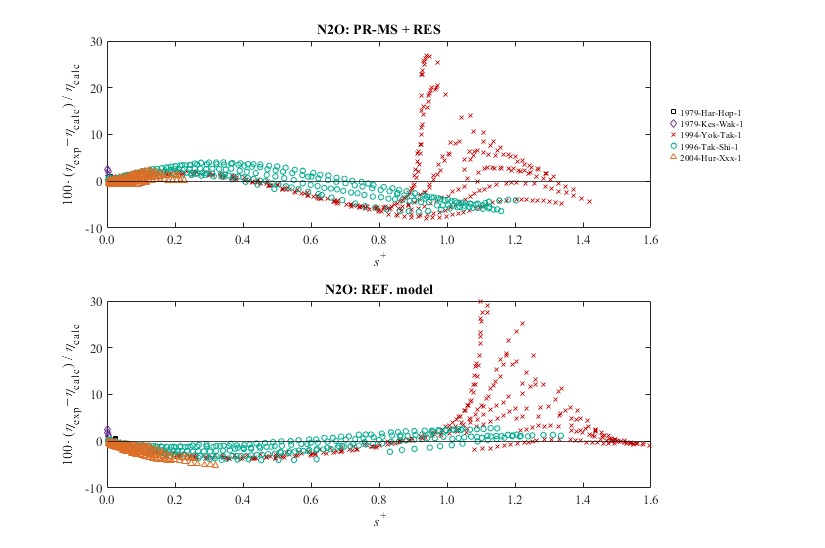

Supplement: Supplementary file 2 [file ao5c01157_si_002.zip › Supporting Information package 2/Figures/Deviation plots/PR-MS/N2O.jpeg]

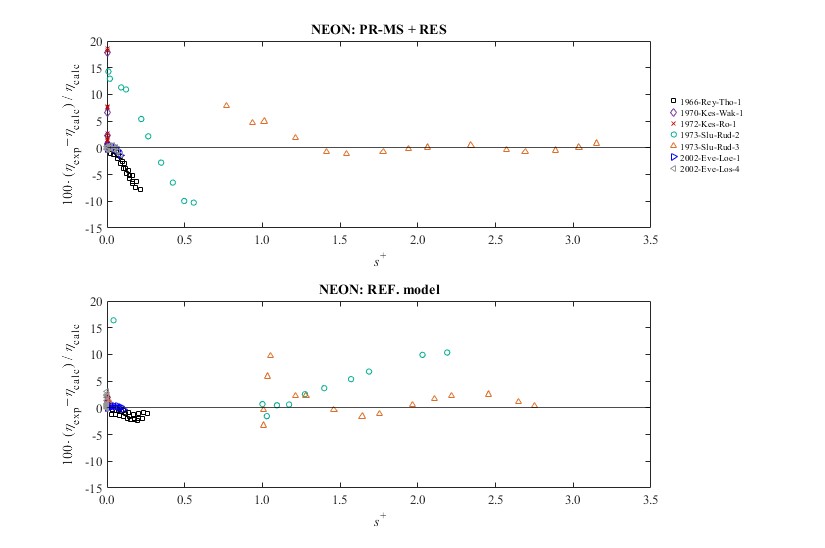

Supplement: Supplementary file 2 [file ao5c01157_si_002.zip › Supporting Information package 2/Figures/Deviation plots/PR-MS/NEON.jpeg]

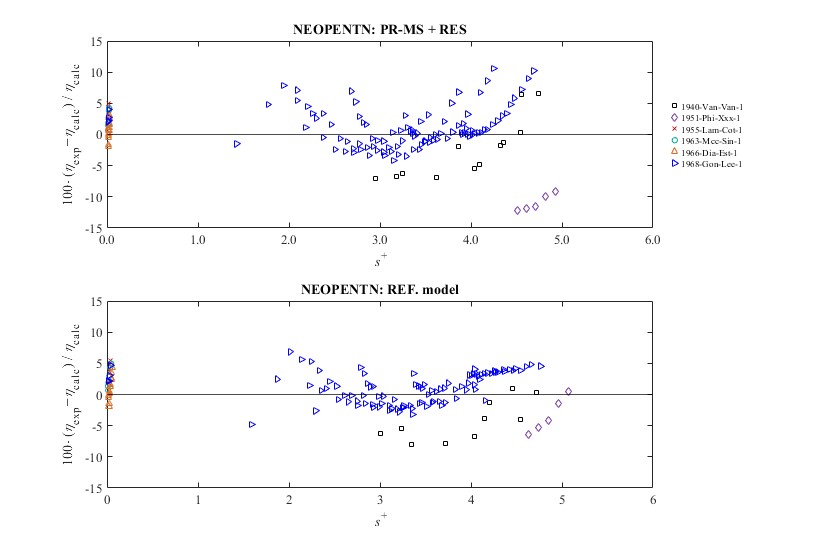

Supplement: Supplementary file 2 [file ao5c01157_si_002.zip › Supporting Information package 2/Figures/Deviation plots/PR-MS/NEOPENTN.jpeg]

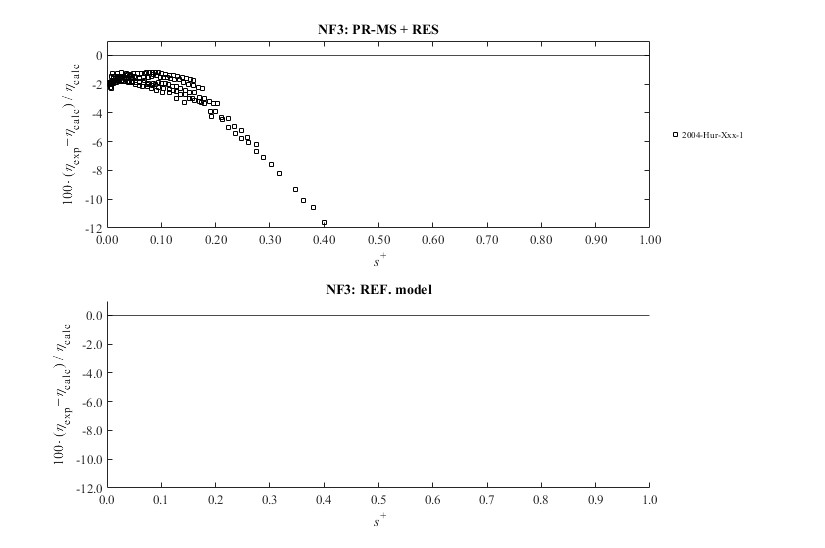

Supplement: Supplementary file 2 [file ao5c01157_si_002.zip › Supporting Information package 2/Figures/Deviation plots/PR-MS/NF3.jpeg]

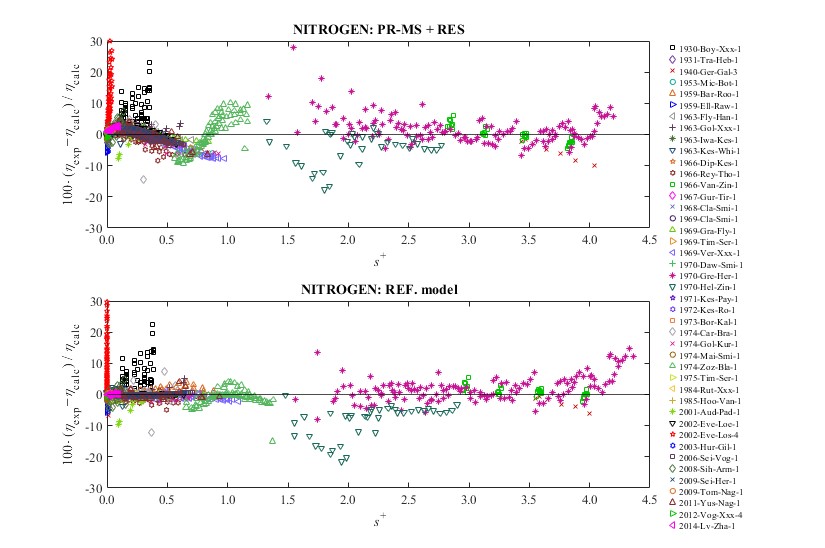

Supplement: Supplementary file 2 [file ao5c01157_si_002.zip › Supporting Information package 2/Figures/Deviation plots/PR-MS/NITROGEN.jpeg]

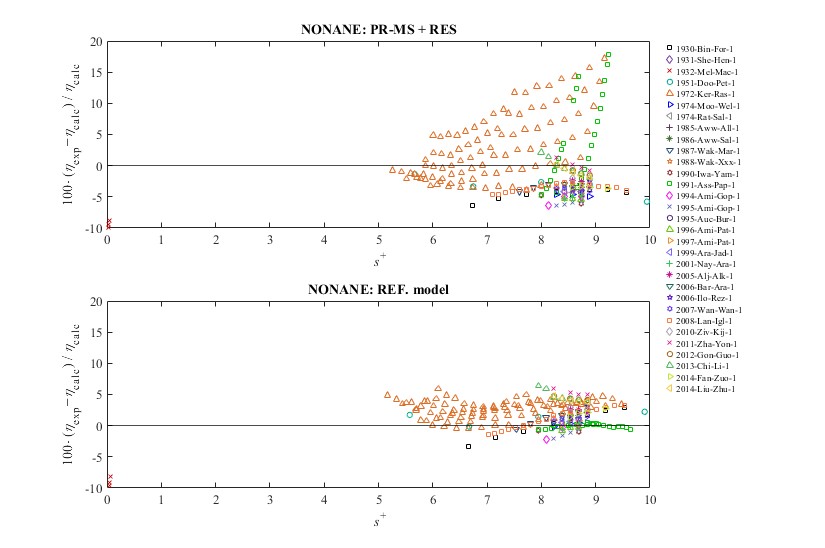

Supplement: Supplementary file 2 [file ao5c01157_si_002.zip › Supporting Information package 2/Figures/Deviation plots/PR-MS/NONANE.jpeg]

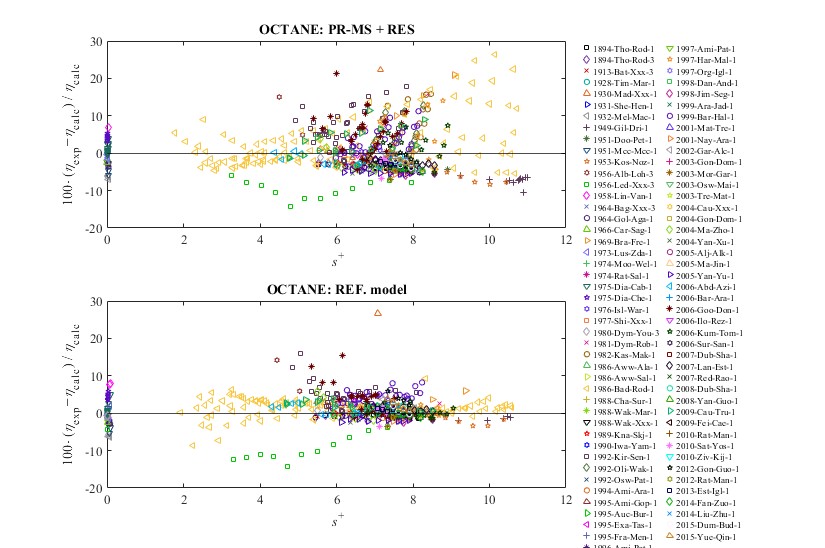

Supplement: Supplementary file 2 [file ao5c01157_si_002.zip › Supporting Information package 2/Figures/Deviation plots/PR-MS/OCTANE.jpeg]

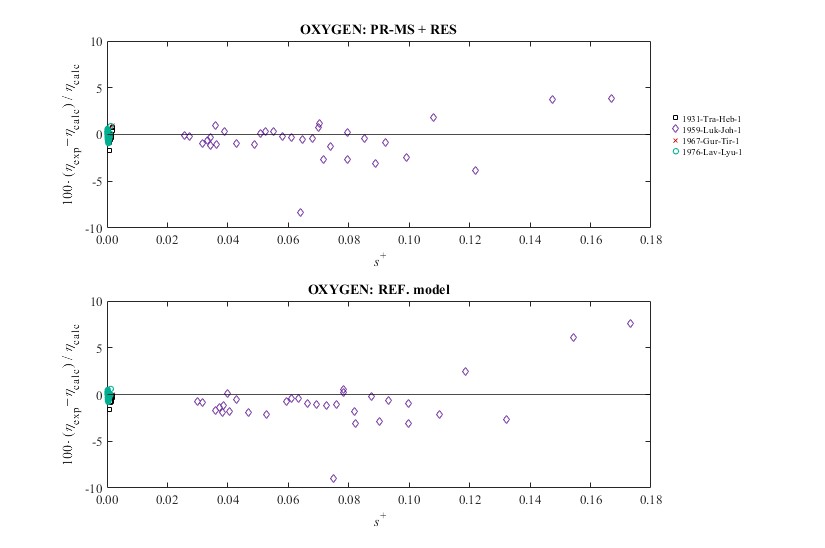

Supplement: Supplementary file 2 [file ao5c01157_si_002.zip › Supporting Information package 2/Figures/Deviation plots/PR-MS/OXYGEN.jpeg]

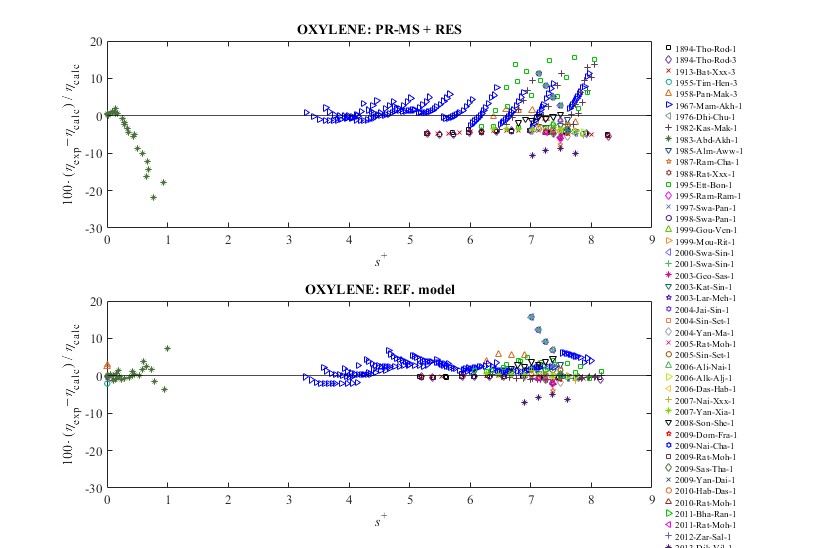

Supplement: Supplementary file 2 [file ao5c01157_si_002.zip › Supporting Information package 2/Figures/Deviation plots/PR-MS/OXYLENE.jpeg]

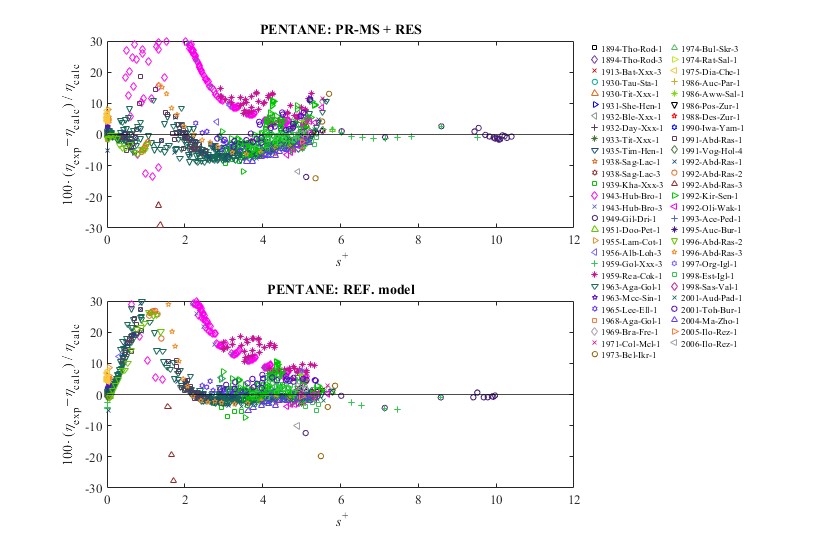

Supplement: Supplementary file 2 [file ao5c01157_si_002.zip › Supporting Information package 2/Figures/Deviation plots/PR-MS/PENTANE.jpeg]

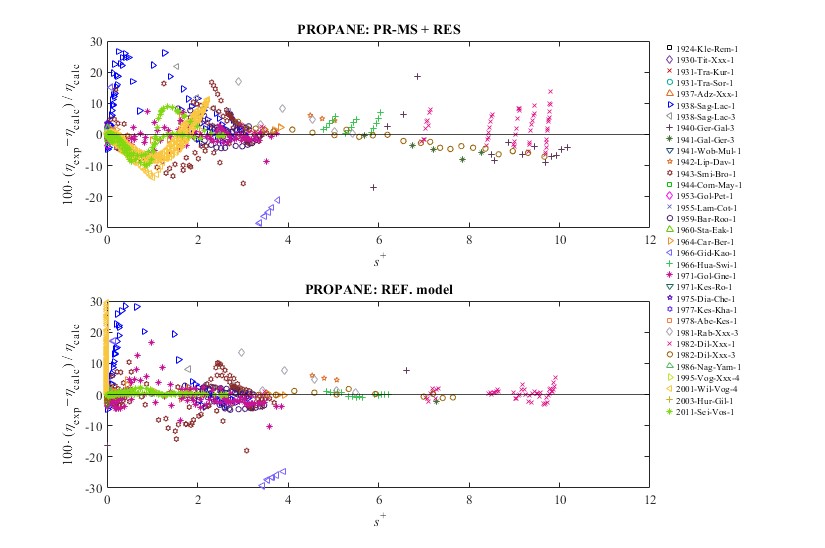

Supplement: Supplementary file 2 [file ao5c01157_si_002.zip › Supporting Information package 2/Figures/Deviation plots/PR-MS/PROPANE.jpeg]

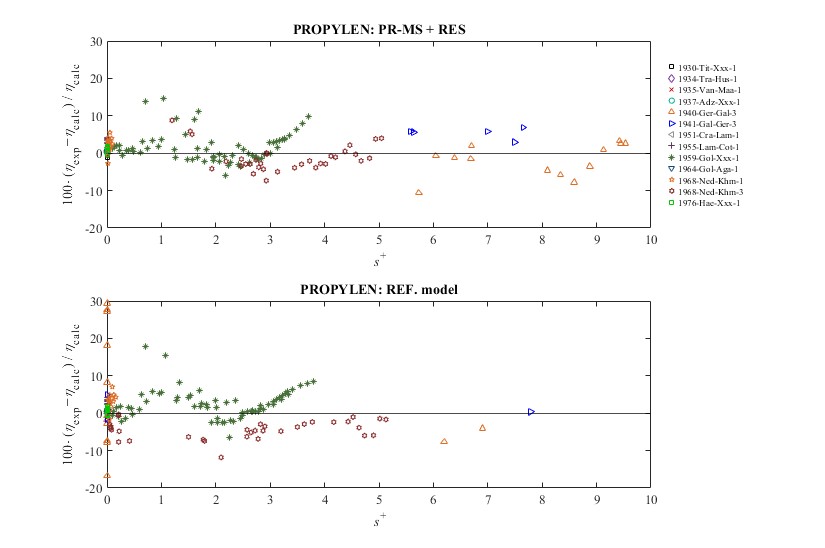

Supplement: Supplementary file 2 [file ao5c01157_si_002.zip › Supporting Information package 2/Figures/Deviation plots/PR-MS/PROPYLEN.jpeg]

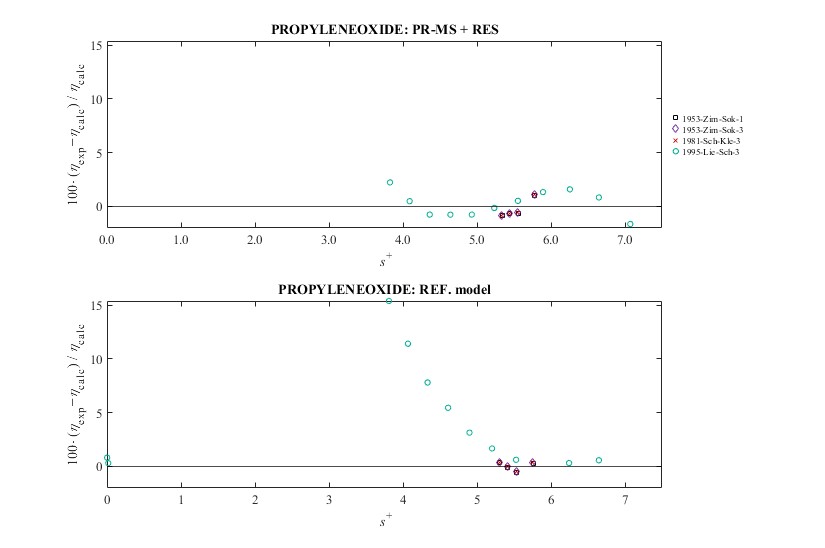

Supplement: Supplementary file 2 [file ao5c01157_si_002.zip › Supporting Information package 2/Figures/Deviation plots/PR-MS/PROPYLENEOXIDE.jpeg]

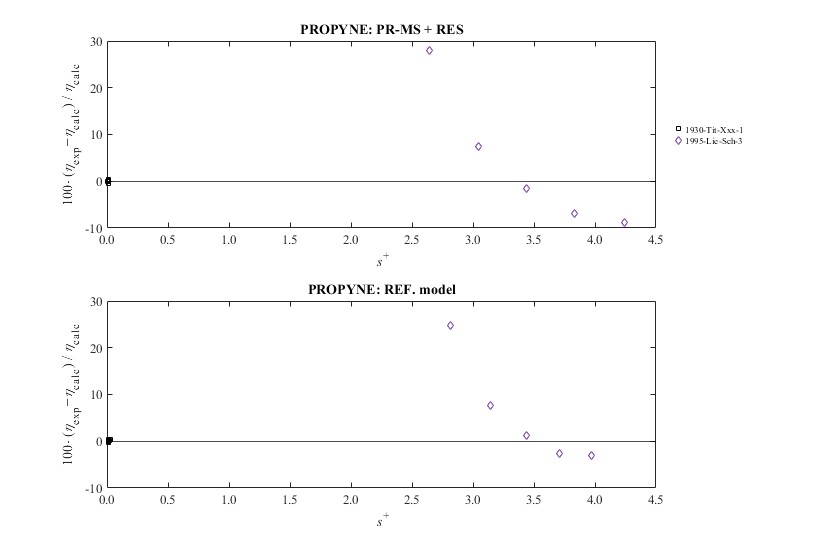

Supplement: Supplementary file 2 [file ao5c01157_si_002.zip › Supporting Information package 2/Figures/Deviation plots/PR-MS/PROPYNE.jpeg]
