# Supplementary figures and images for: Evaluating Cubic Equations of State with Various α Functions for Viscosity Predictions of 124 Industrial Important Fluids Based on Residual Entropy Scaling (part 3 of 4)
Source: ACS Omega. 2025 Jun 27;10(27):29021–36. doi: 10.1021/acsomega.5c01157 (PMC12268422; doi:10.1021/acsomega.5c01157)

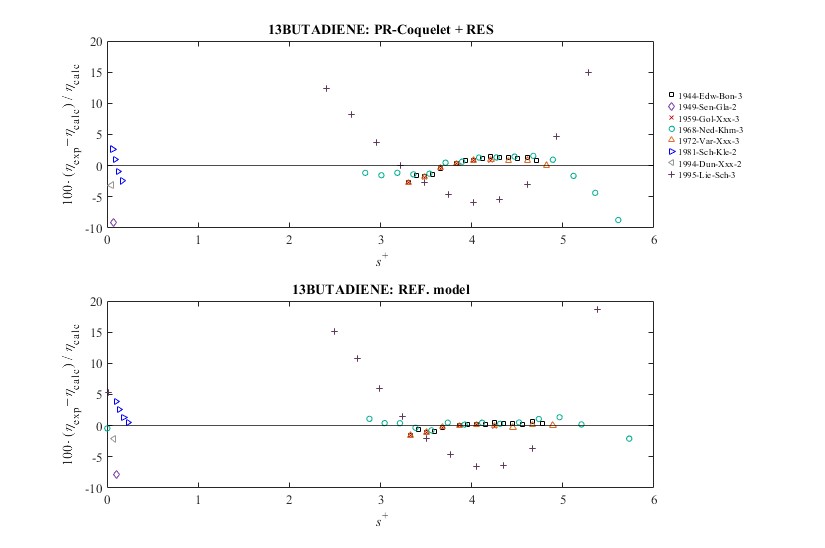

Supplement: Supplementary file 2 [file ao5c01157_si_002.zip › Supporting Information package 2/Figures/Deviation plots/PR-Coquelet/13BUTADIENE.jpeg]

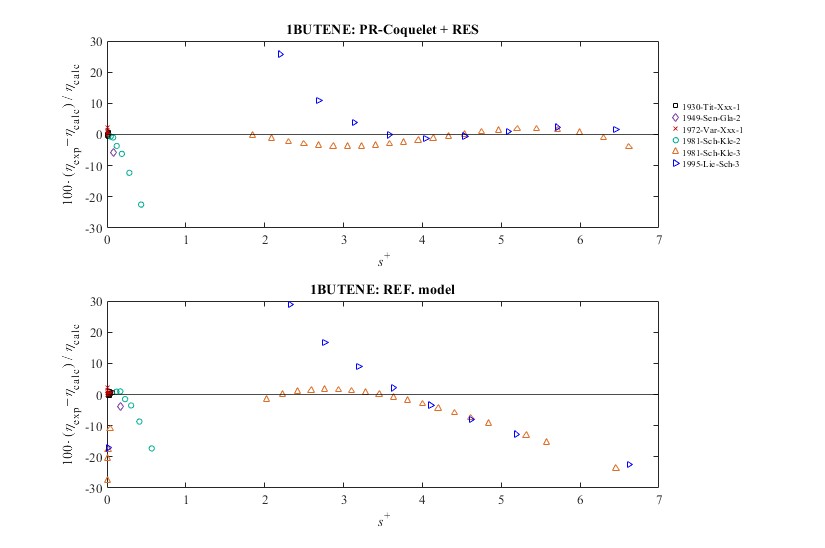

Supplement: Supplementary file 2 [file ao5c01157_si_002.zip › Supporting Information package 2/Figures/Deviation plots/PR-Coquelet/1BUTENE.jpeg]

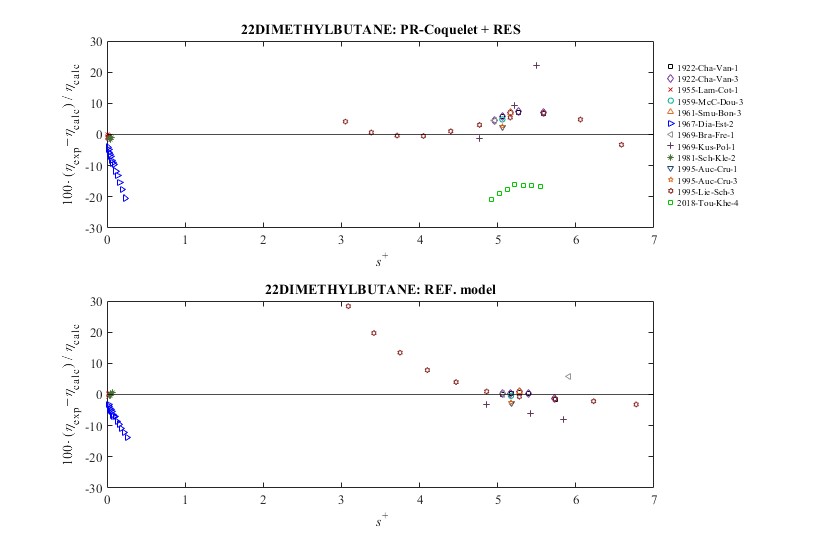

Supplement: Supplementary file 2 [file ao5c01157_si_002.zip › Supporting Information package 2/Figures/Deviation plots/PR-Coquelet/22DIMETHYLBUTANE.jpeg]

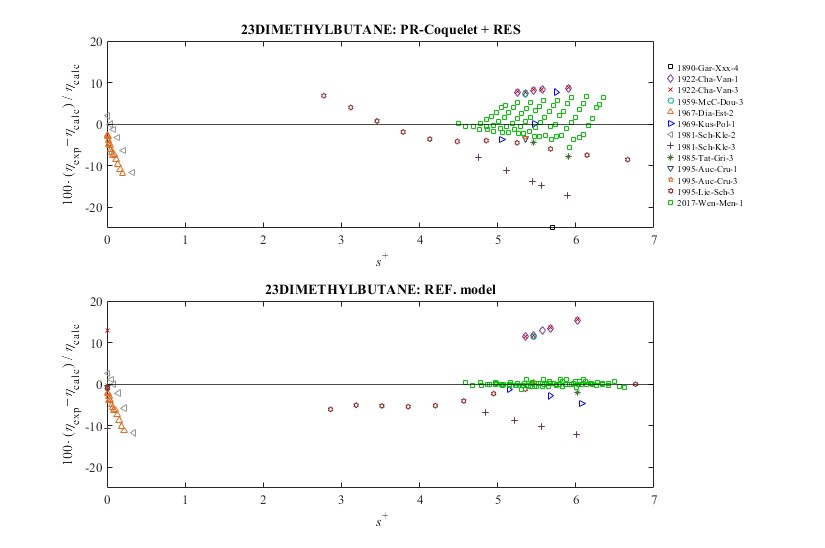

Supplement: Supplementary file 2 [file ao5c01157_si_002.zip › Supporting Information package 2/Figures/Deviation plots/PR-Coquelet/23DIMETHYLBUTANE.jpeg]

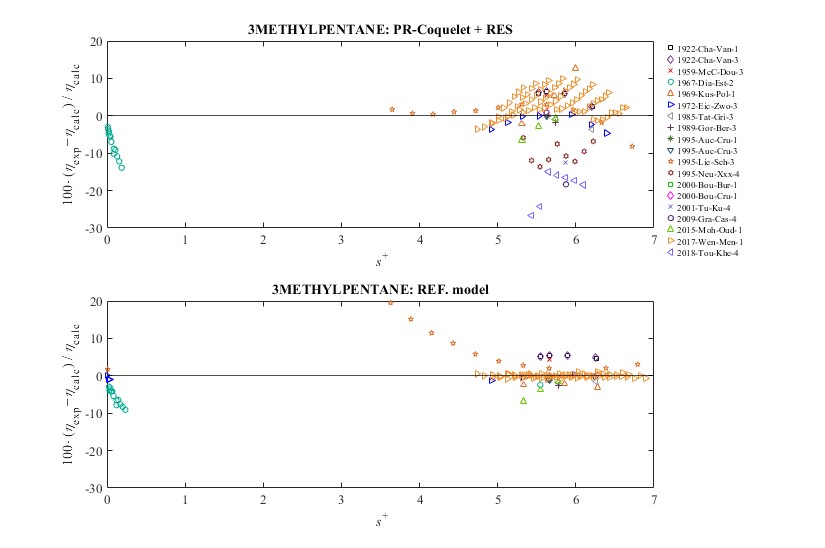

Supplement: Supplementary file 2 [file ao5c01157_si_002.zip › Supporting Information package 2/Figures/Deviation plots/PR-Coquelet/3METHYLPENTANE.jpeg]

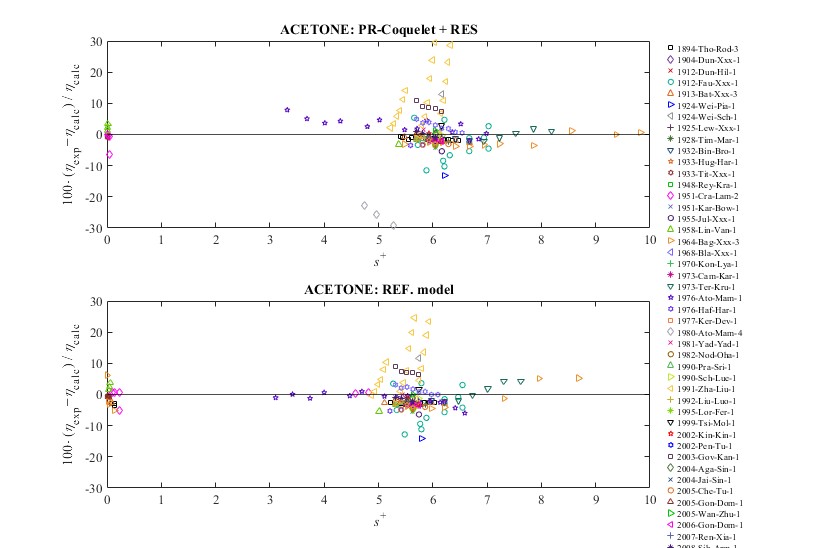

Supplement: Supplementary file 2 [file ao5c01157_si_002.zip › Supporting Information package 2/Figures/Deviation plots/PR-Coquelet/ACETONE.jpeg]

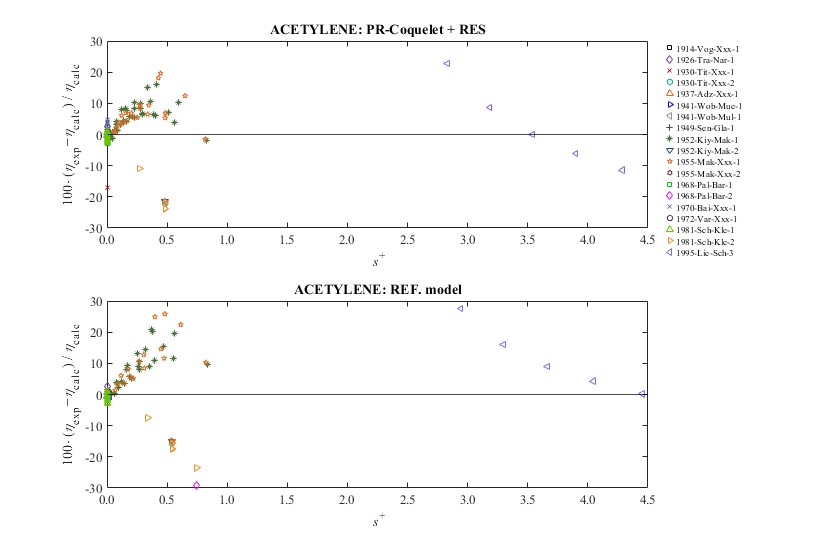

Supplement: Supplementary file 2 [file ao5c01157_si_002.zip › Supporting Information package 2/Figures/Deviation plots/PR-Coquelet/ACETYLENE.jpeg]

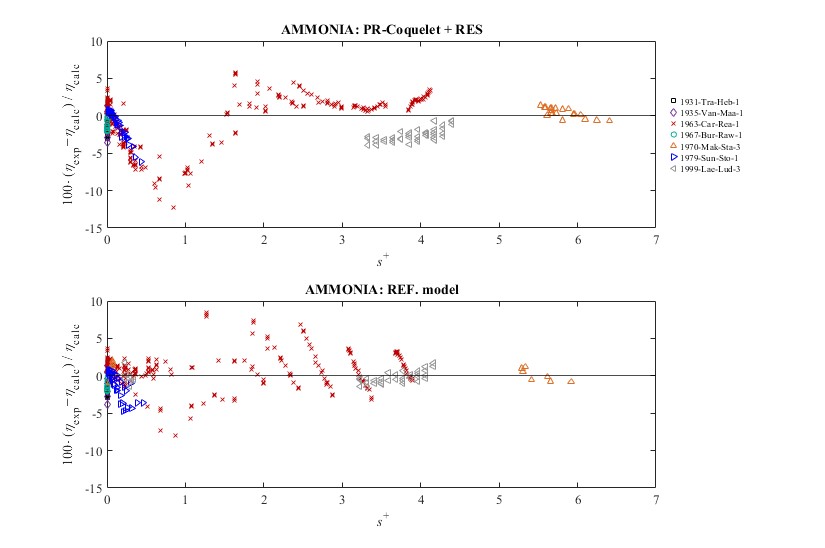

Supplement: Supplementary file 2 [file ao5c01157_si_002.zip › Supporting Information package 2/Figures/Deviation plots/PR-Coquelet/AMMONIA.jpeg]

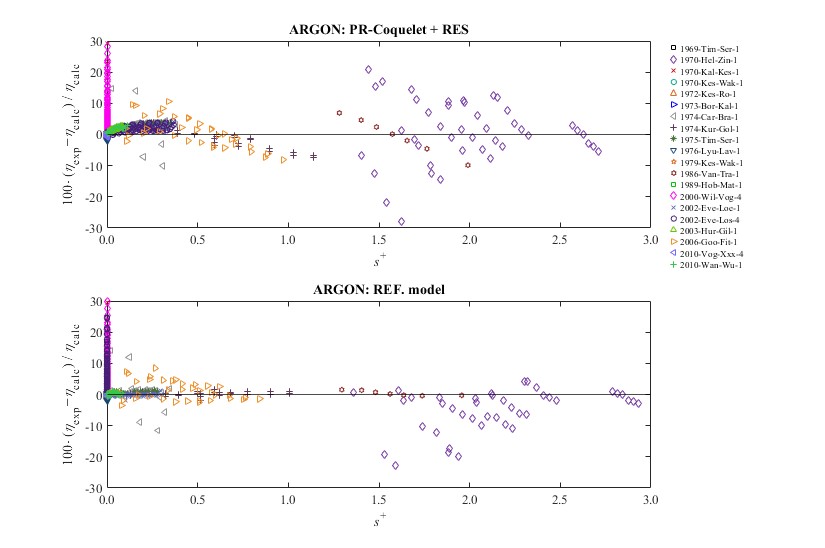

Supplement: Supplementary file 2 [file ao5c01157_si_002.zip › Supporting Information package 2/Figures/Deviation plots/PR-Coquelet/ARGON.jpeg]

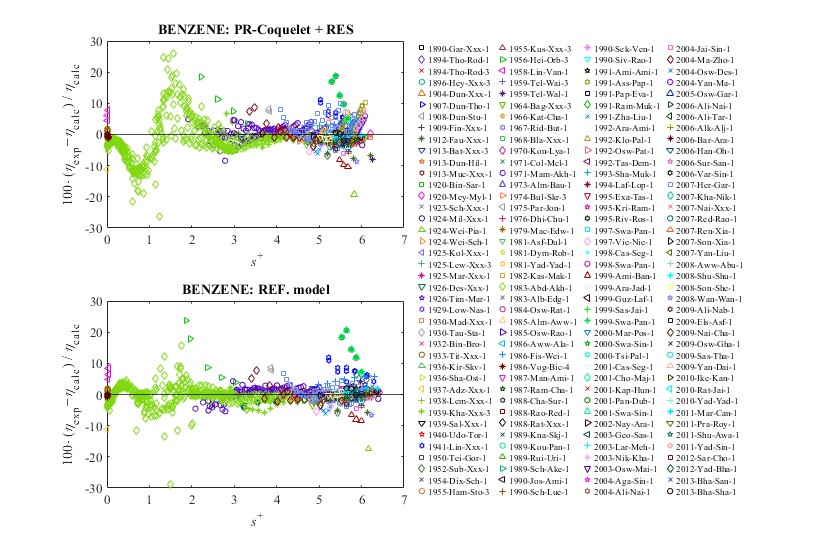

Supplement: Supplementary file 2 [file ao5c01157_si_002.zip › Supporting Information package 2/Figures/Deviation plots/PR-Coquelet/BENZENE.jpeg]

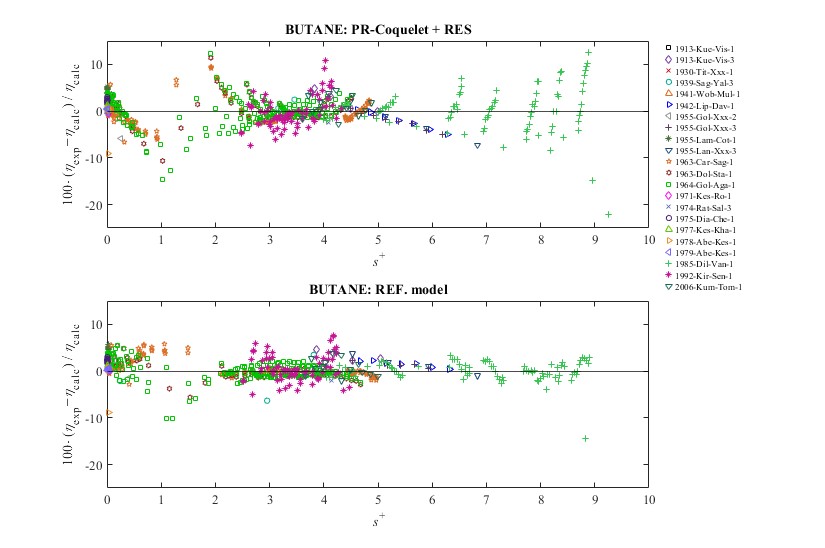

Supplement: Supplementary file 2 [file ao5c01157_si_002.zip › Supporting Information package 2/Figures/Deviation plots/PR-Coquelet/BUTANE.jpeg]

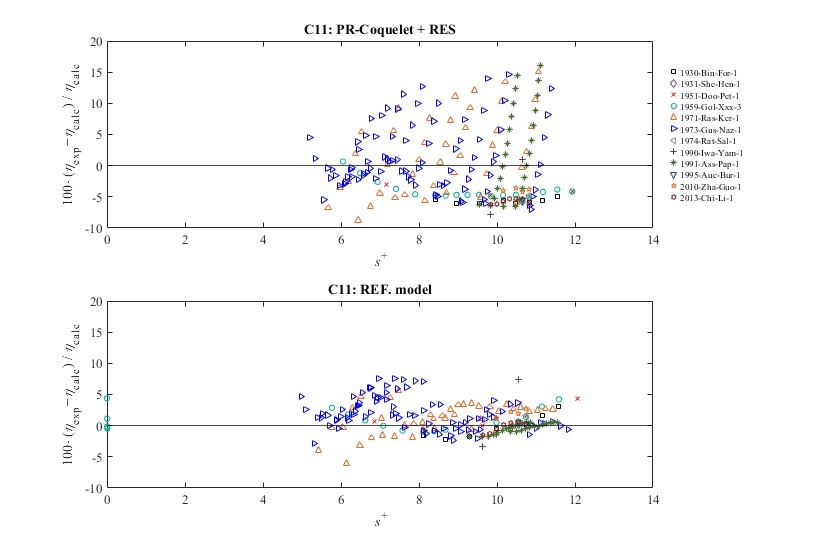

Supplement: Supplementary file 2 [file ao5c01157_si_002.zip › Supporting Information package 2/Figures/Deviation plots/PR-Coquelet/C11.jpeg]

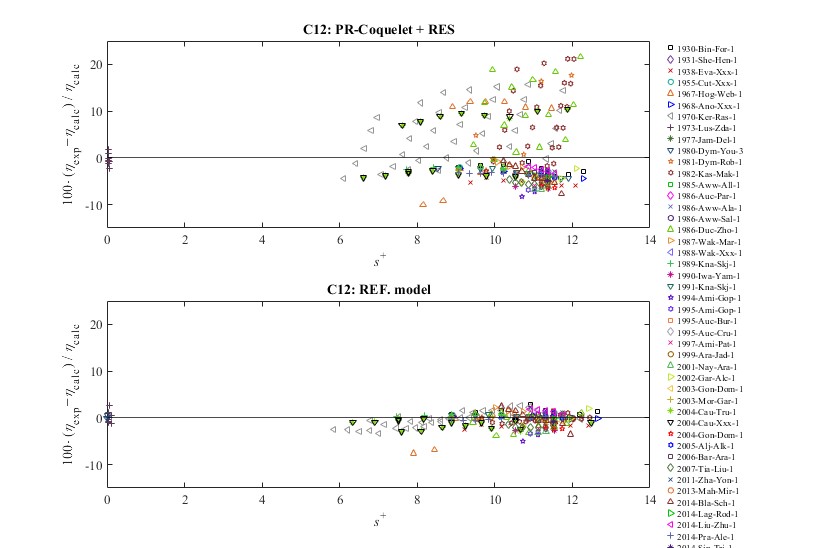

Supplement: Supplementary file 2 [file ao5c01157_si_002.zip › Supporting Information package 2/Figures/Deviation plots/PR-Coquelet/C12.jpeg]

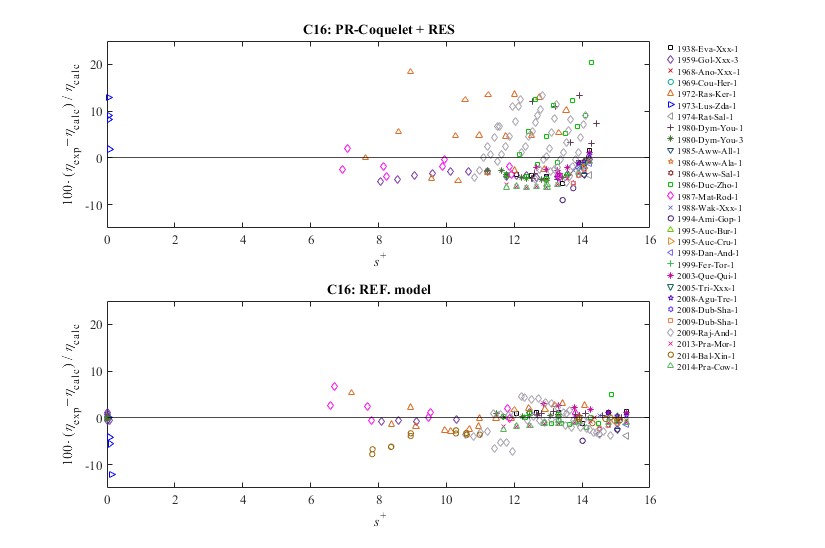

Supplement: Supplementary file 2 [file ao5c01157_si_002.zip › Supporting Information package 2/Figures/Deviation plots/PR-Coquelet/C16.jpeg]

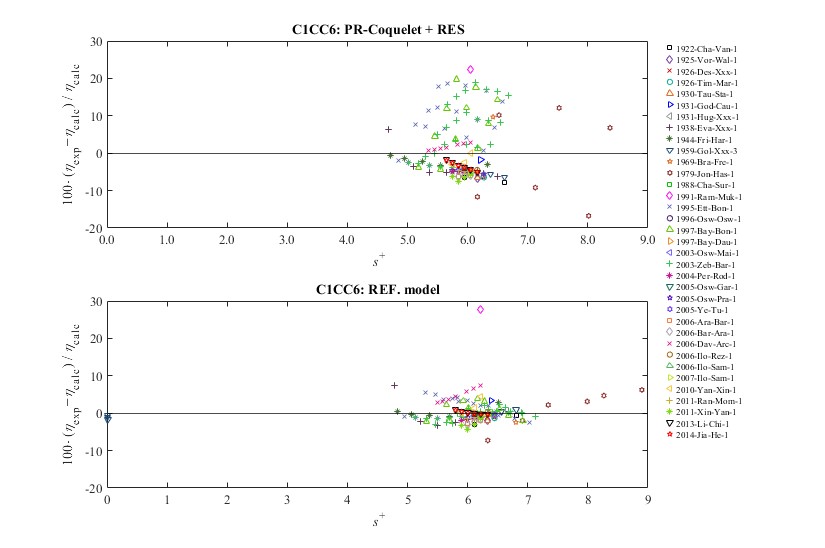

Supplement: Supplementary file 2 [file ao5c01157_si_002.zip › Supporting Information package 2/Figures/Deviation plots/PR-Coquelet/C1CC6.jpeg]

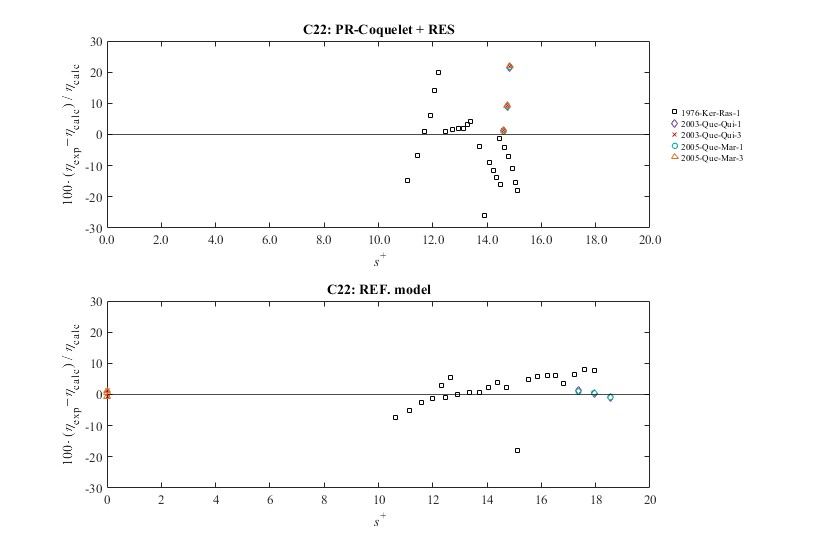

Supplement: Supplementary file 2 [file ao5c01157_si_002.zip › Supporting Information package 2/Figures/Deviation plots/PR-Coquelet/C22.jpeg]

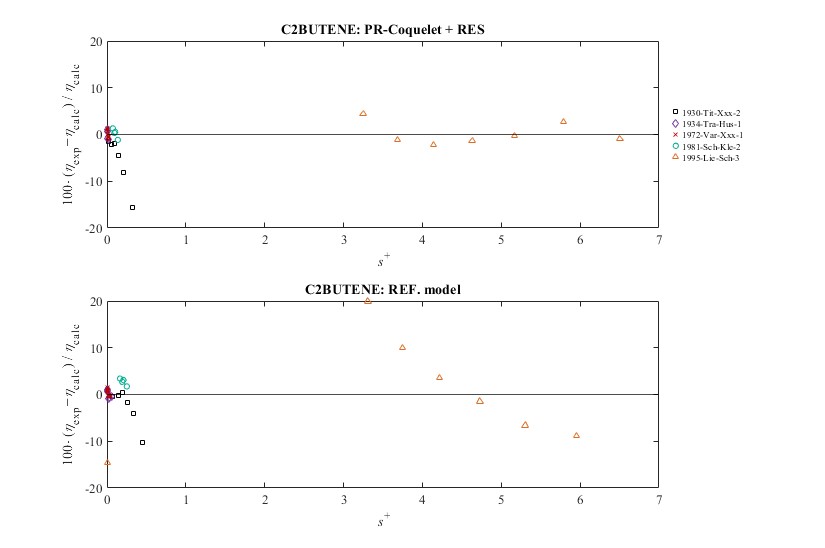

Supplement: Supplementary file 2 [file ao5c01157_si_002.zip › Supporting Information package 2/Figures/Deviation plots/PR-Coquelet/C2BUTENE.jpeg]

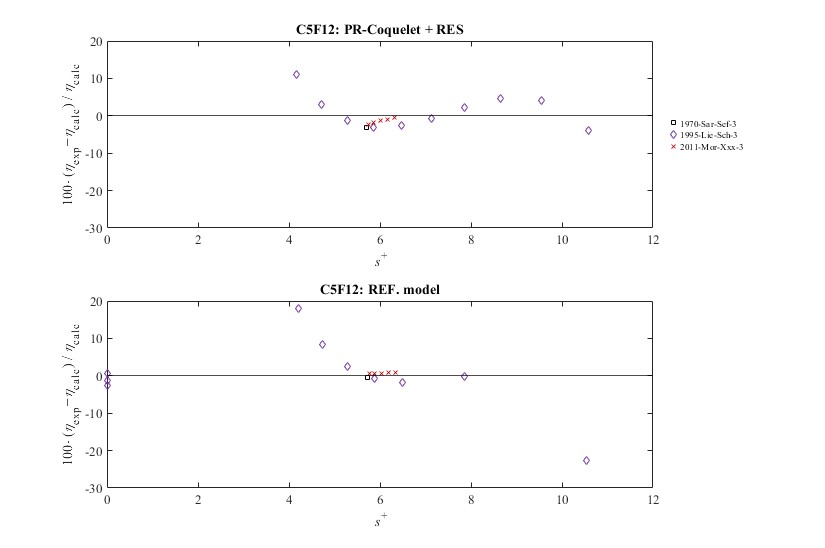

Supplement: Supplementary file 2 [file ao5c01157_si_002.zip › Supporting Information package 2/Figures/Deviation plots/PR-Coquelet/C5F12.jpeg]

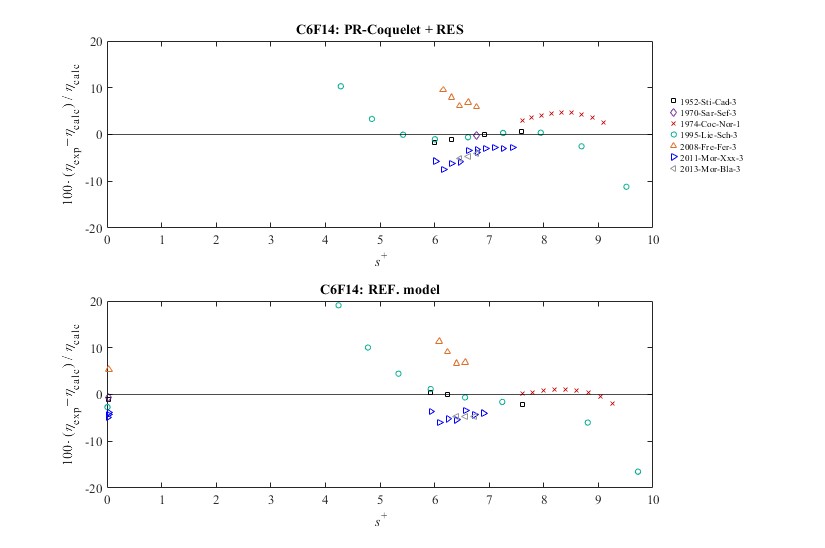

Supplement: Supplementary file 2 [file ao5c01157_si_002.zip › Supporting Information package 2/Figures/Deviation plots/PR-Coquelet/C6F14.jpeg]

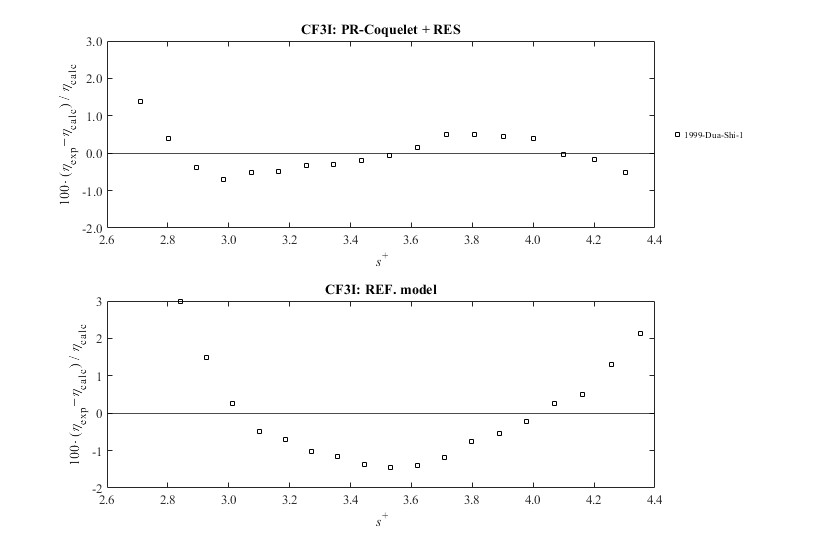

Supplement: Supplementary file 2 [file ao5c01157_si_002.zip › Supporting Information package 2/Figures/Deviation plots/PR-Coquelet/CF3I.jpeg]

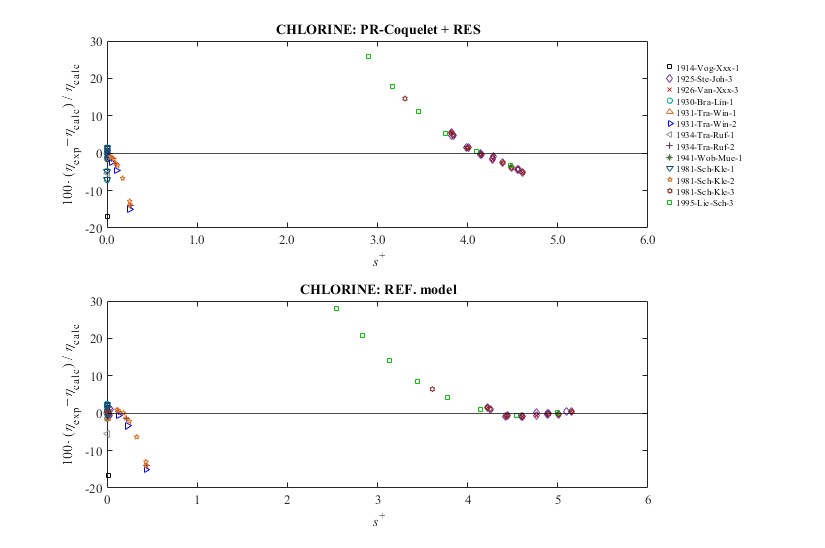

Supplement: Supplementary file 2 [file ao5c01157_si_002.zip › Supporting Information package 2/Figures/Deviation plots/PR-Coquelet/CHLORINE.jpeg]

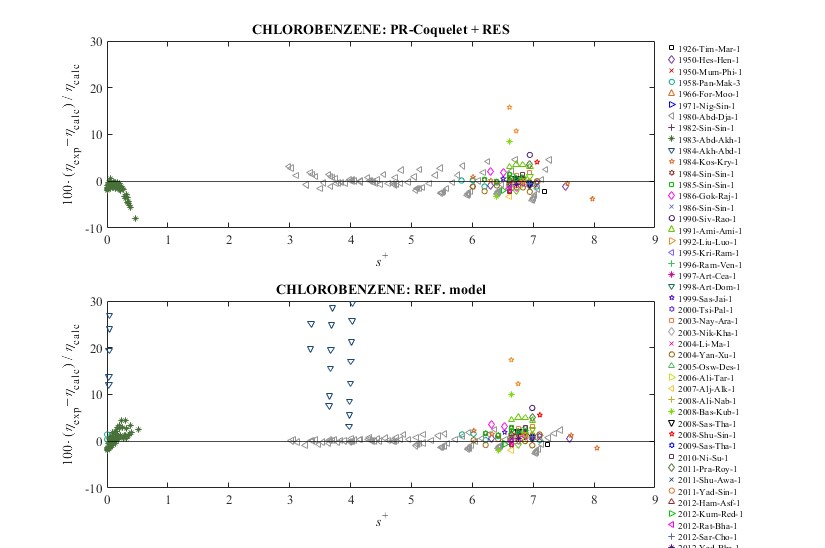

Supplement: Supplementary file 2 [file ao5c01157_si_002.zip › Supporting Information package 2/Figures/Deviation plots/PR-Coquelet/CHLOROBENZENE.jpeg]

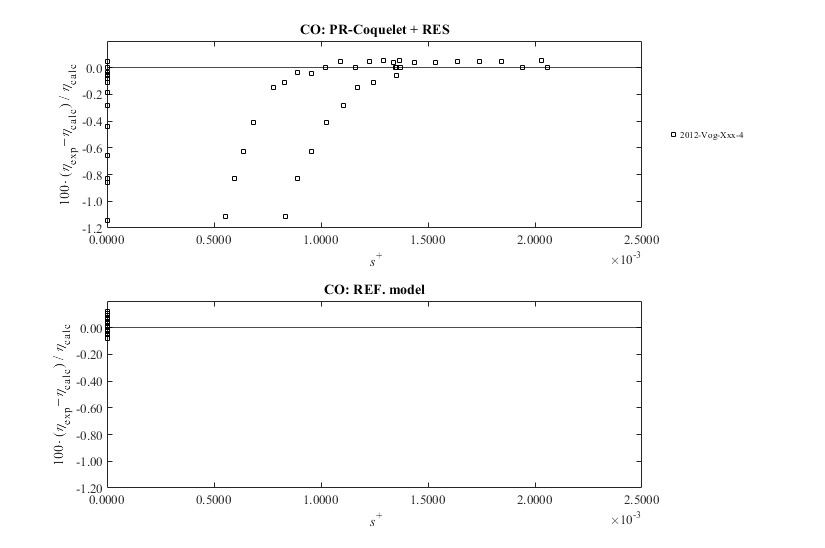

Supplement: Supplementary file 2 [file ao5c01157_si_002.zip › Supporting Information package 2/Figures/Deviation plots/PR-Coquelet/CO.jpeg]

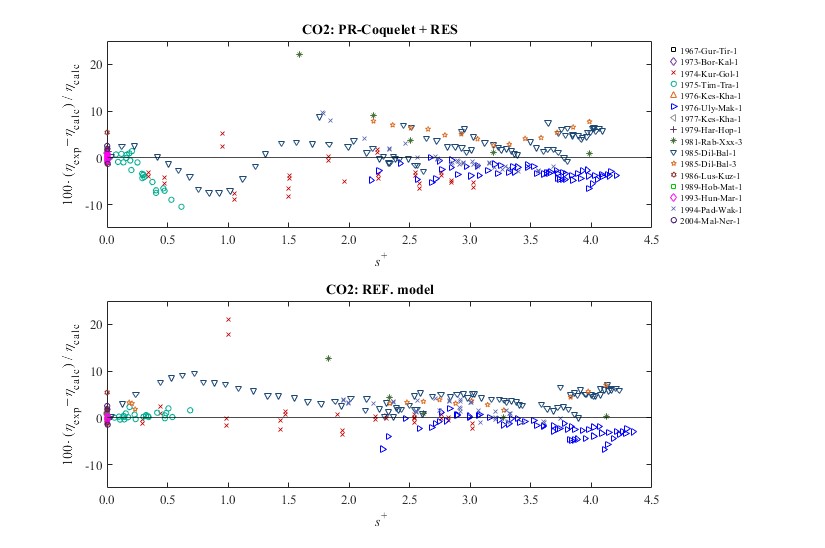

Supplement: Supplementary file 2 [file ao5c01157_si_002.zip › Supporting Information package 2/Figures/Deviation plots/PR-Coquelet/CO2.jpeg]

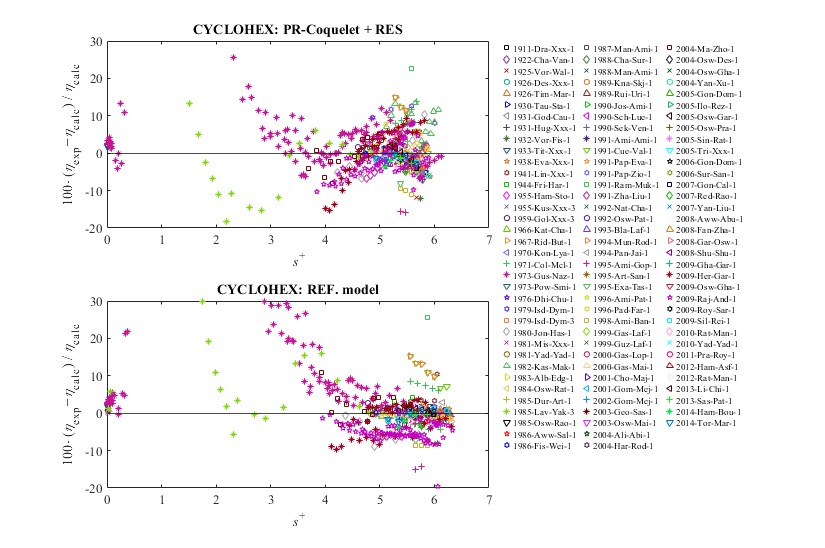

Supplement: Supplementary file 2 [file ao5c01157_si_002.zip › Supporting Information package 2/Figures/Deviation plots/PR-Coquelet/CYCLOHEX.jpeg]

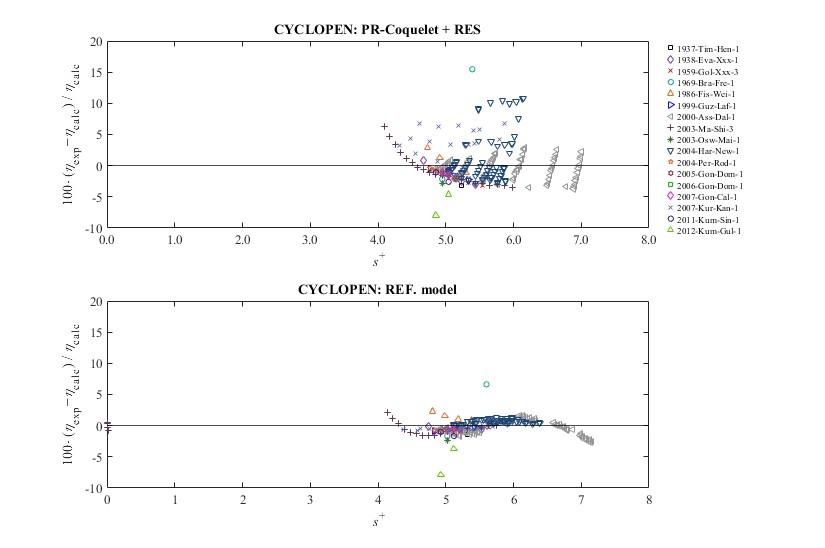

Supplement: Supplementary file 2 [file ao5c01157_si_002.zip › Supporting Information package 2/Figures/Deviation plots/PR-Coquelet/CYCLOPEN.jpeg]

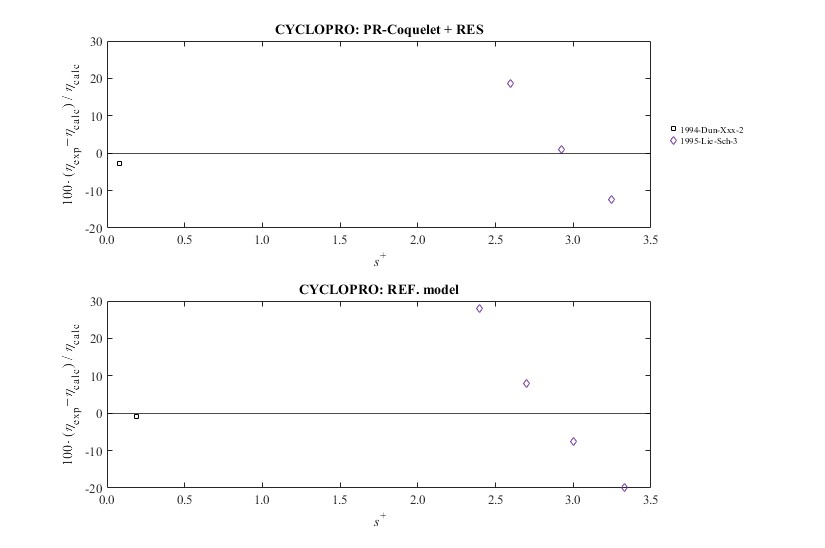

Supplement: Supplementary file 2 [file ao5c01157_si_002.zip › Supporting Information package 2/Figures/Deviation plots/PR-Coquelet/CYCLOPRO.jpeg]

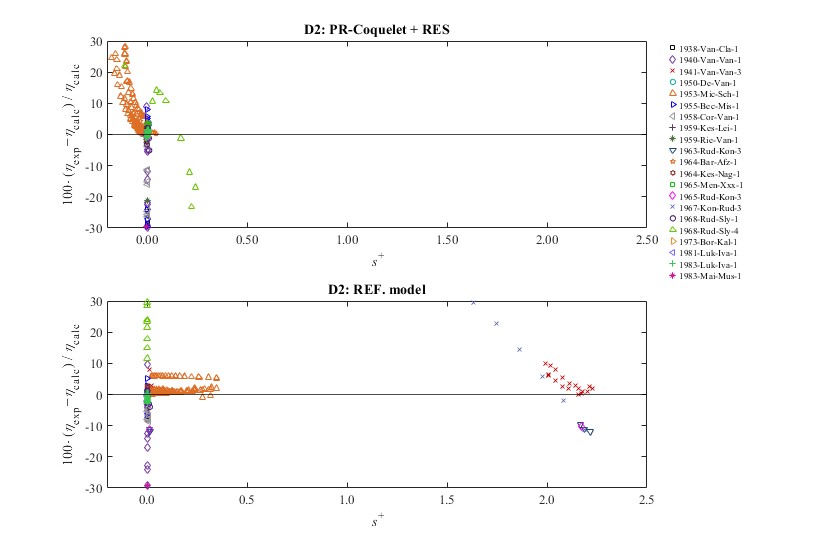

Supplement: Supplementary file 2 [file ao5c01157_si_002.zip › Supporting Information package 2/Figures/Deviation plots/PR-Coquelet/D2.jpeg]

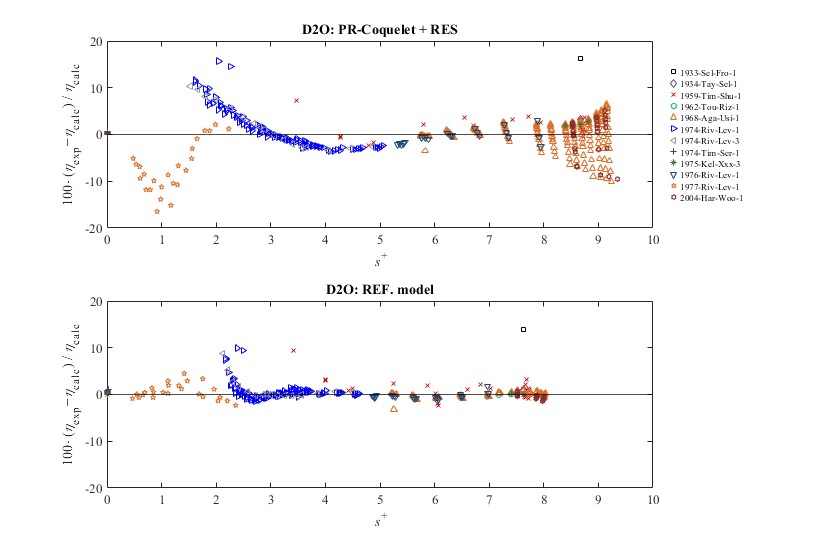

Supplement: Supplementary file 2 [file ao5c01157_si_002.zip › Supporting Information package 2/Figures/Deviation plots/PR-Coquelet/D2O.jpeg]

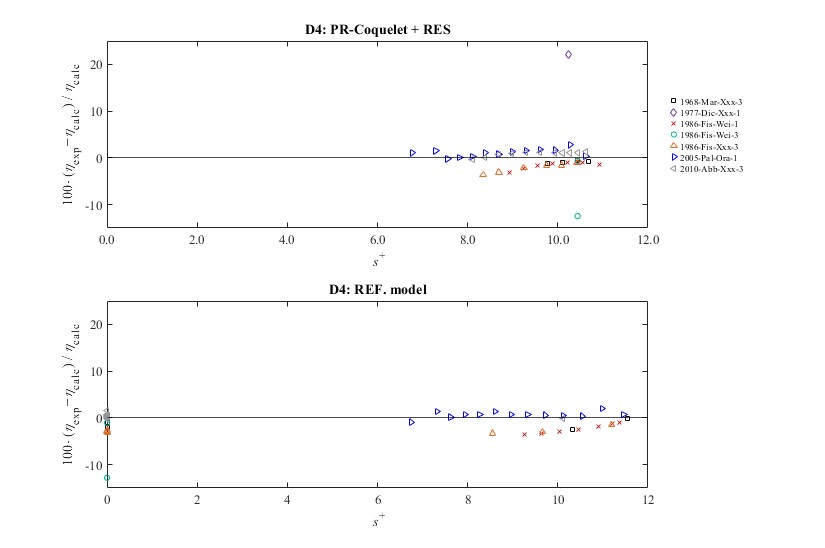

Supplement: Supplementary file 2 [file ao5c01157_si_002.zip › Supporting Information package 2/Figures/Deviation plots/PR-Coquelet/D4.jpeg]

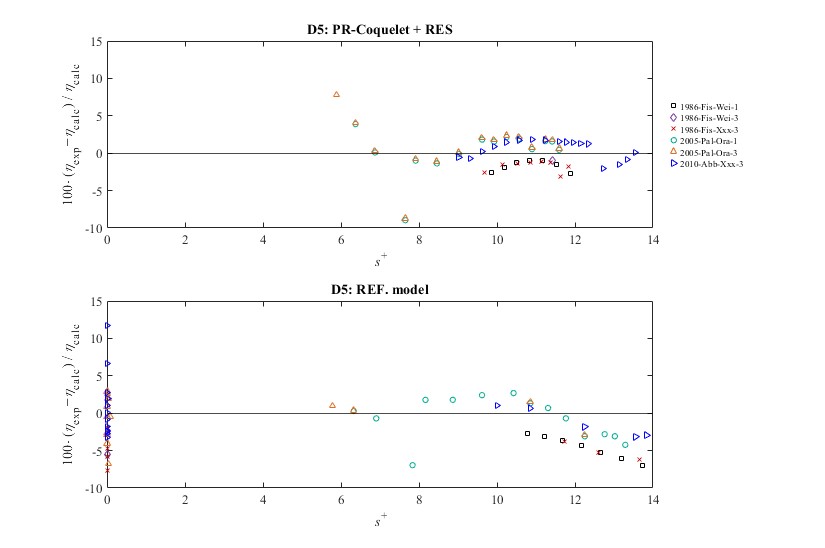

Supplement: Supplementary file 2 [file ao5c01157_si_002.zip › Supporting Information package 2/Figures/Deviation plots/PR-Coquelet/D5.jpeg]

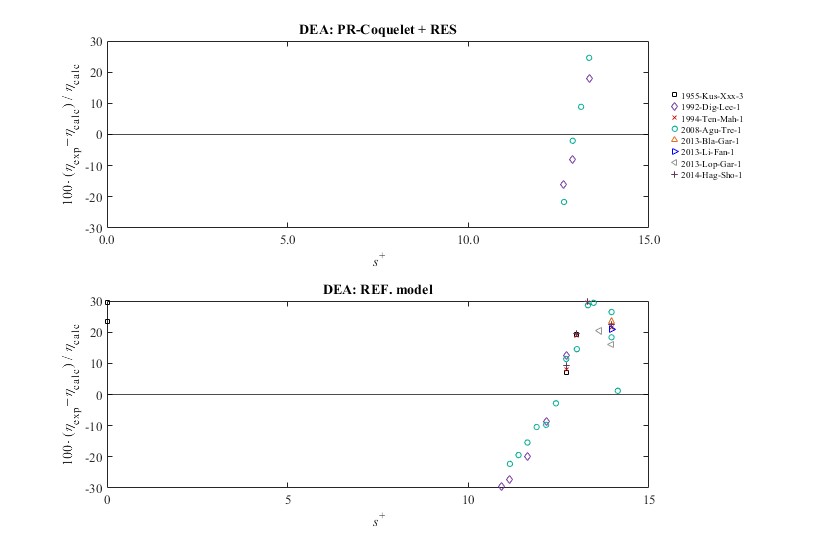

Supplement: Supplementary file 2 [file ao5c01157_si_002.zip › Supporting Information package 2/Figures/Deviation plots/PR-Coquelet/DEA.jpeg]

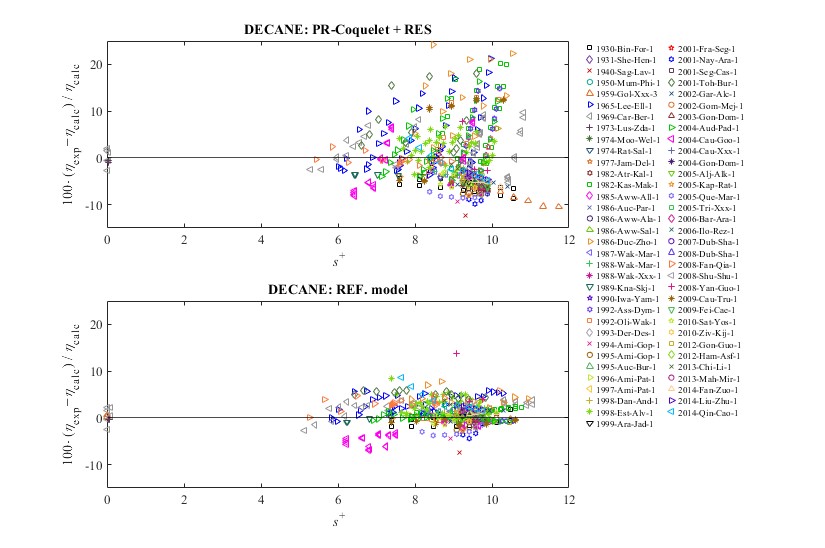

Supplement: Supplementary file 2 [file ao5c01157_si_002.zip › Supporting Information package 2/Figures/Deviation plots/PR-Coquelet/DECANE.jpeg]

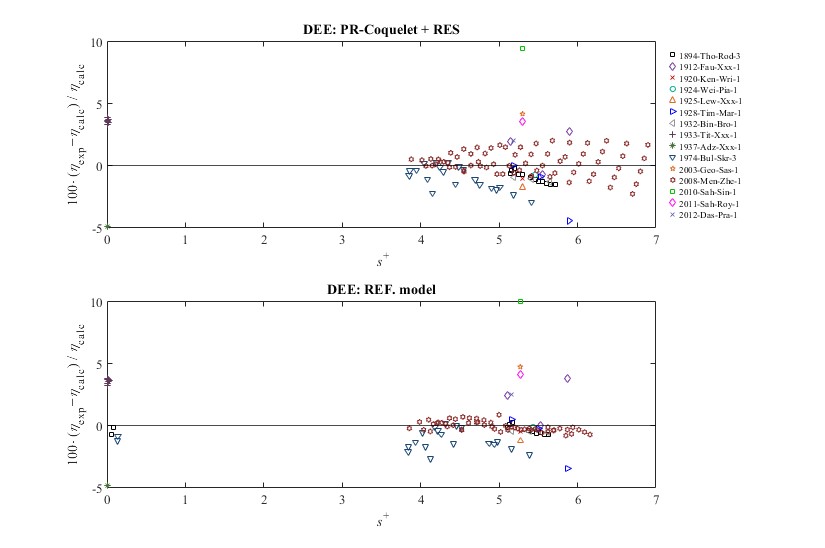

Supplement: Supplementary file 2 [file ao5c01157_si_002.zip › Supporting Information package 2/Figures/Deviation plots/PR-Coquelet/DEE.jpeg]

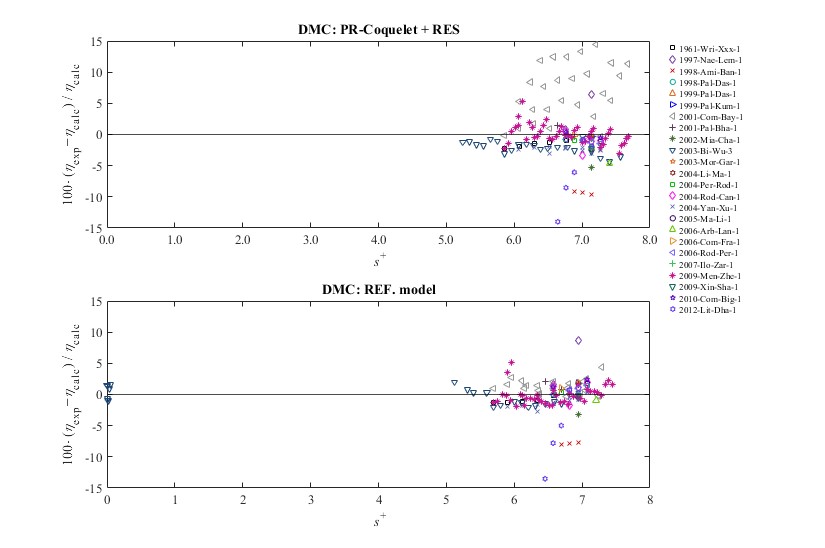

Supplement: Supplementary file 2 [file ao5c01157_si_002.zip › Supporting Information package 2/Figures/Deviation plots/PR-Coquelet/DMC.jpeg]

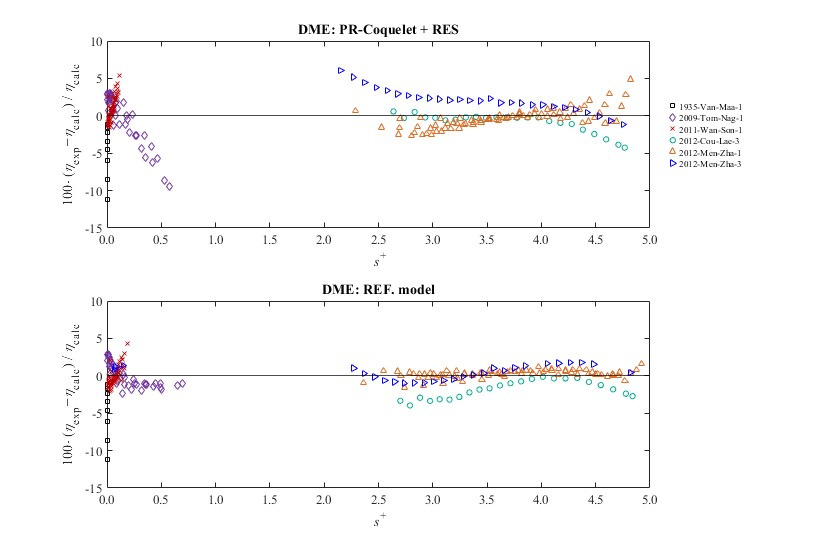

Supplement: Supplementary file 2 [file ao5c01157_si_002.zip › Supporting Information package 2/Figures/Deviation plots/PR-Coquelet/DME.jpeg]

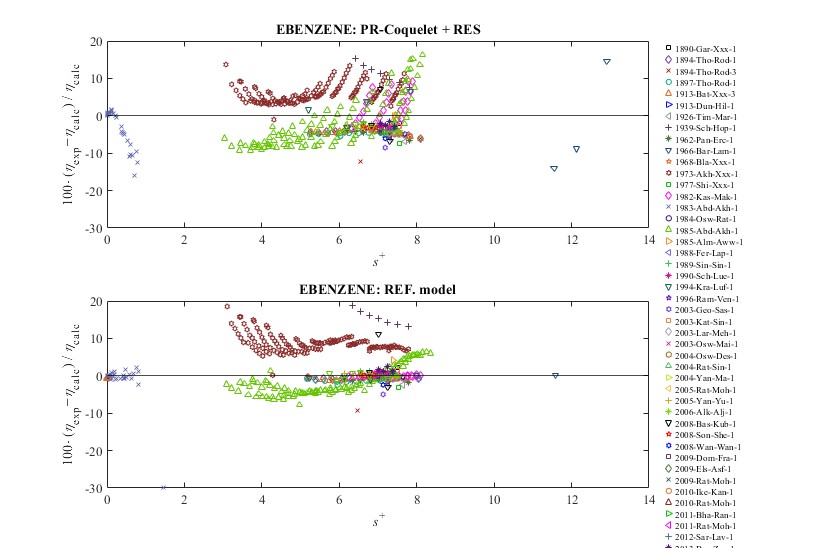

Supplement: Supplementary file 2 [file ao5c01157_si_002.zip › Supporting Information package 2/Figures/Deviation plots/PR-Coquelet/EBENZENE.jpeg]

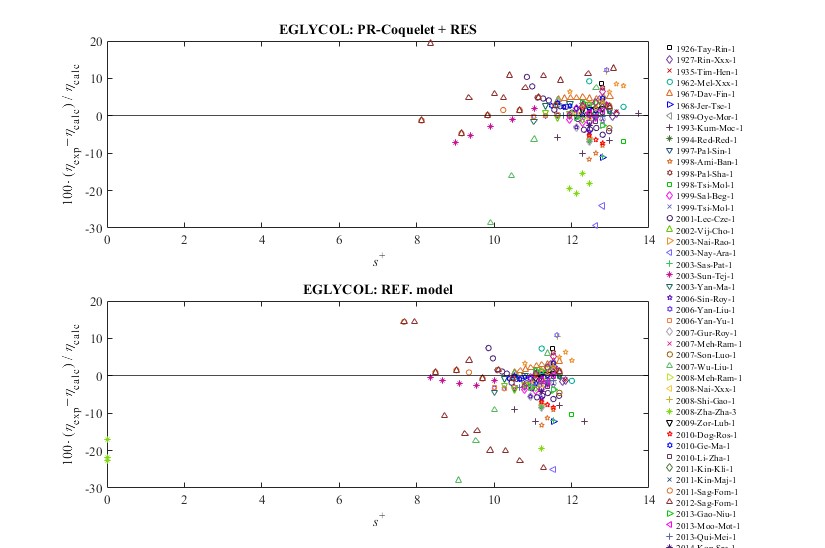

Supplement: Supplementary file 2 [file ao5c01157_si_002.zip › Supporting Information package 2/Figures/Deviation plots/PR-Coquelet/EGLYCOL.jpeg]

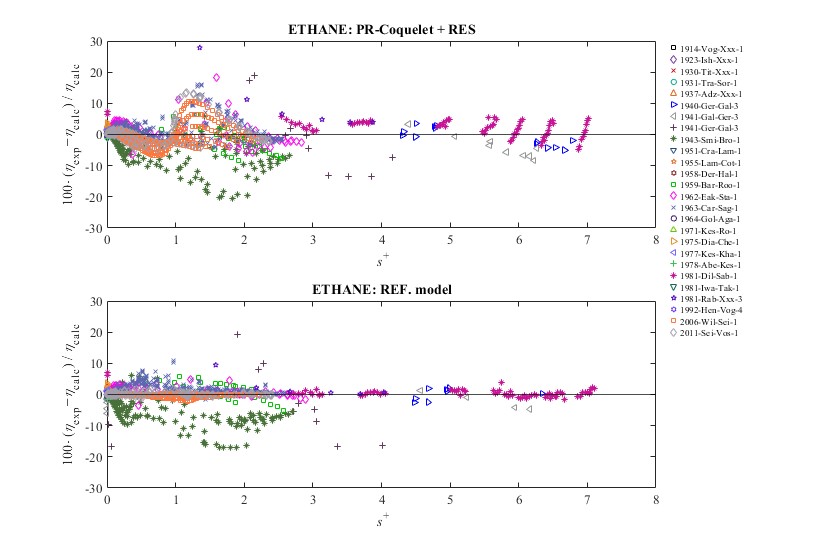

Supplement: Supplementary file 2 [file ao5c01157_si_002.zip › Supporting Information package 2/Figures/Deviation plots/PR-Coquelet/ETHANE.jpeg]

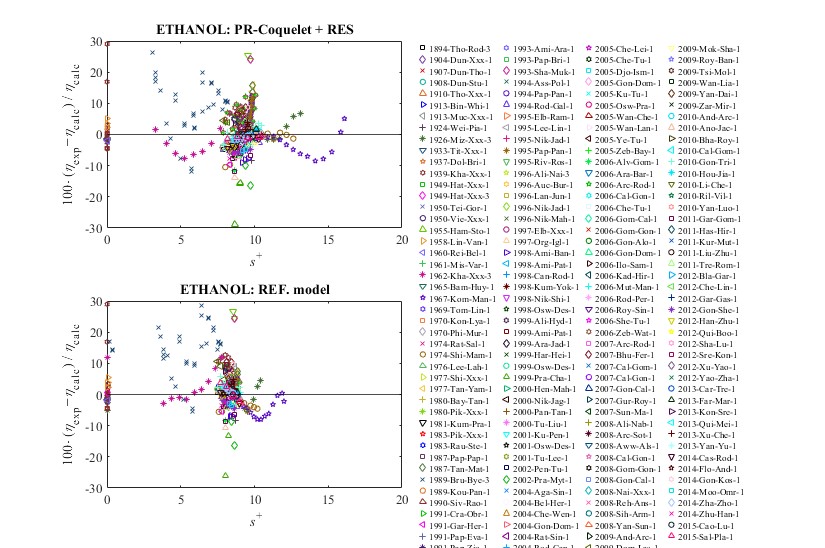

Supplement: Supplementary file 2 [file ao5c01157_si_002.zip › Supporting Information package 2/Figures/Deviation plots/PR-Coquelet/ETHANOL.jpeg]

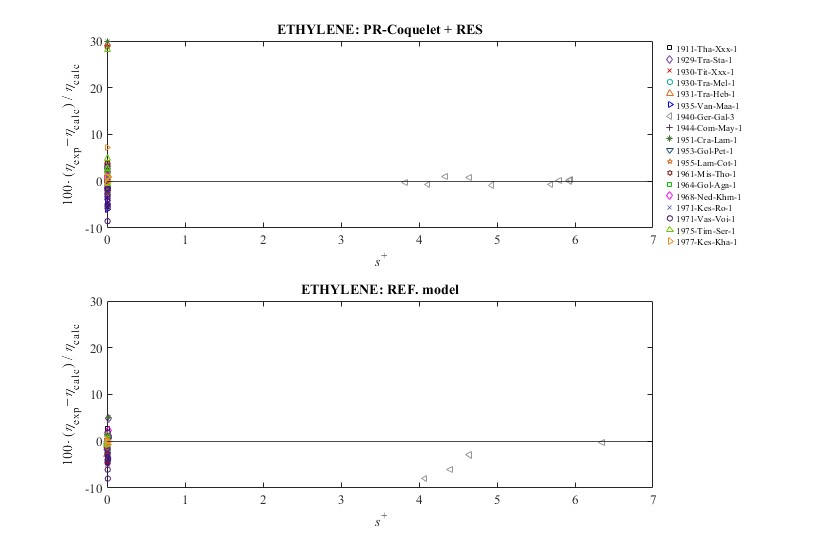

Supplement: Supplementary file 2 [file ao5c01157_si_002.zip › Supporting Information package 2/Figures/Deviation plots/PR-Coquelet/ETHYLENE.jpeg]

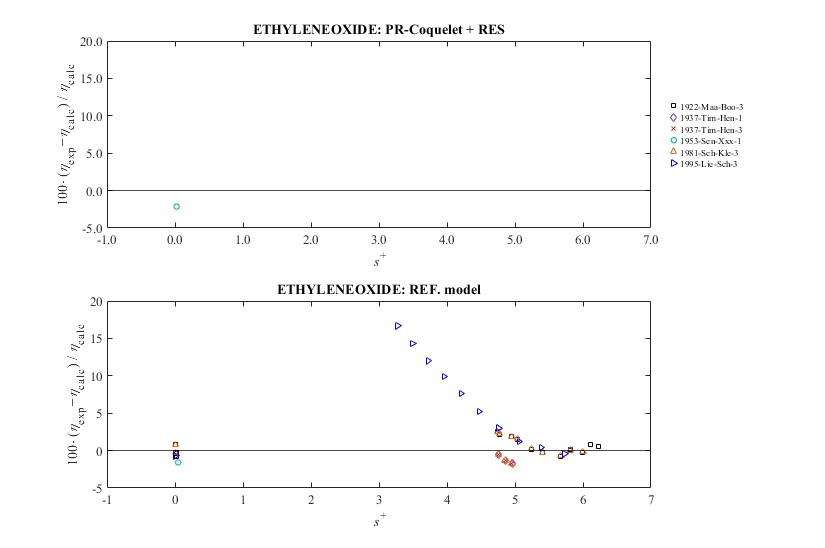

Supplement: Supplementary file 2 [file ao5c01157_si_002.zip › Supporting Information package 2/Figures/Deviation plots/PR-Coquelet/ETHYLENEOXIDE.jpeg]

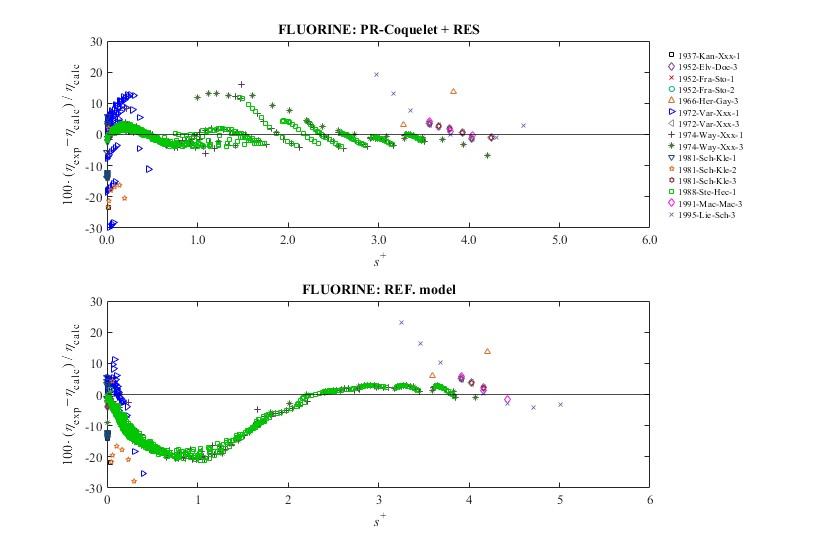

Supplement: Supplementary file 2 [file ao5c01157_si_002.zip › Supporting Information package 2/Figures/Deviation plots/PR-Coquelet/FLUORINE.jpeg]

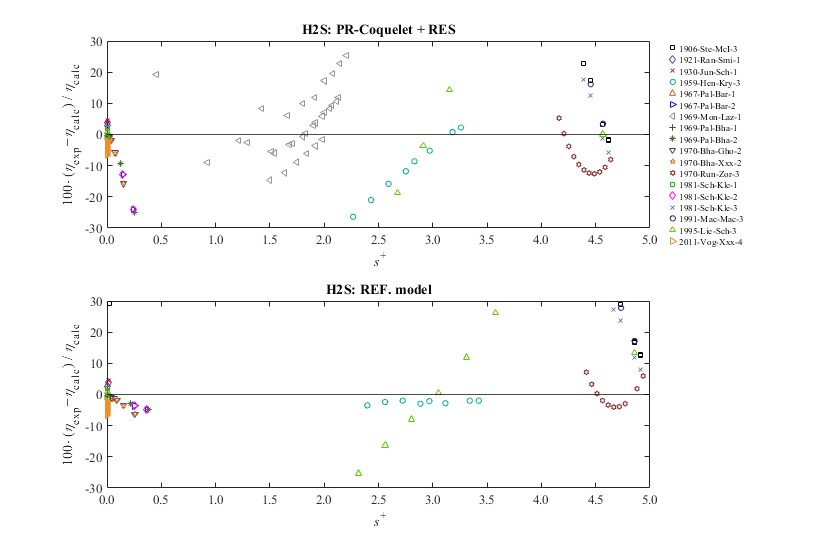

Supplement: Supplementary file 2 [file ao5c01157_si_002.zip › Supporting Information package 2/Figures/Deviation plots/PR-Coquelet/H2S.jpeg]

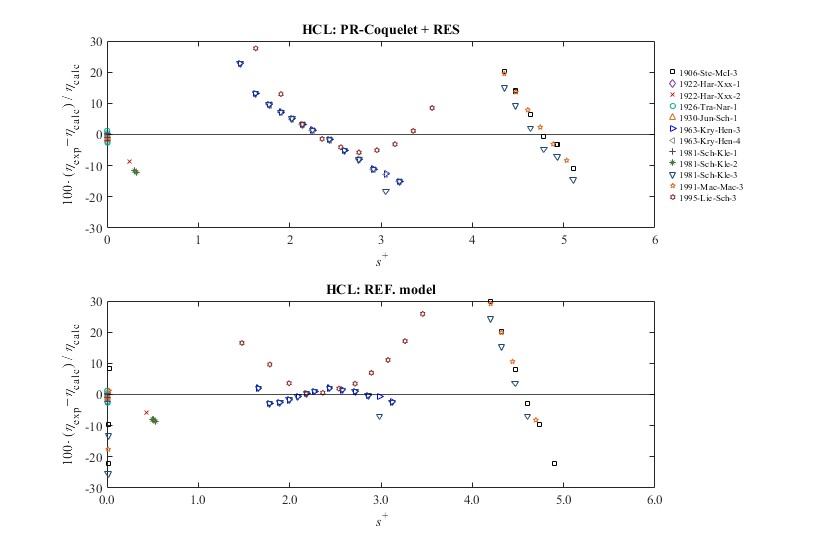

Supplement: Supplementary file 2 [file ao5c01157_si_002.zip › Supporting Information package 2/Figures/Deviation plots/PR-Coquelet/HCL.jpeg]

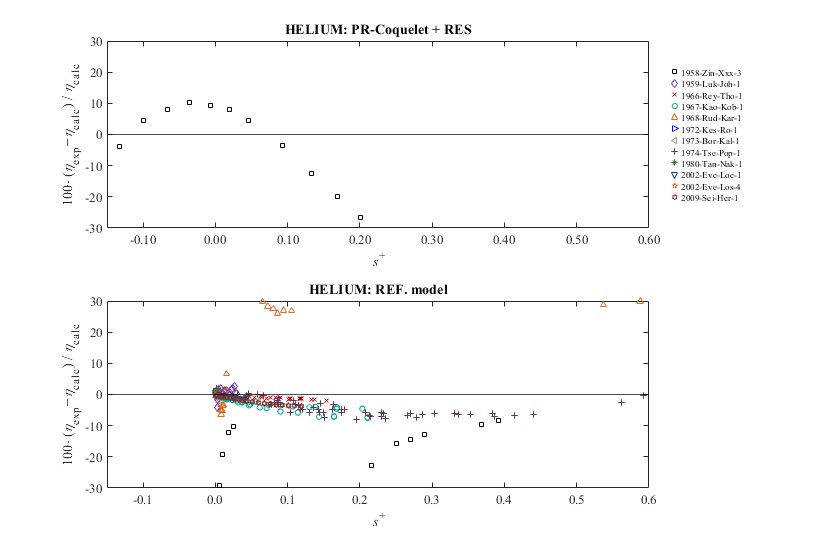

Supplement: Supplementary file 2 [file ao5c01157_si_002.zip › Supporting Information package 2/Figures/Deviation plots/PR-Coquelet/HELIUM.jpeg]

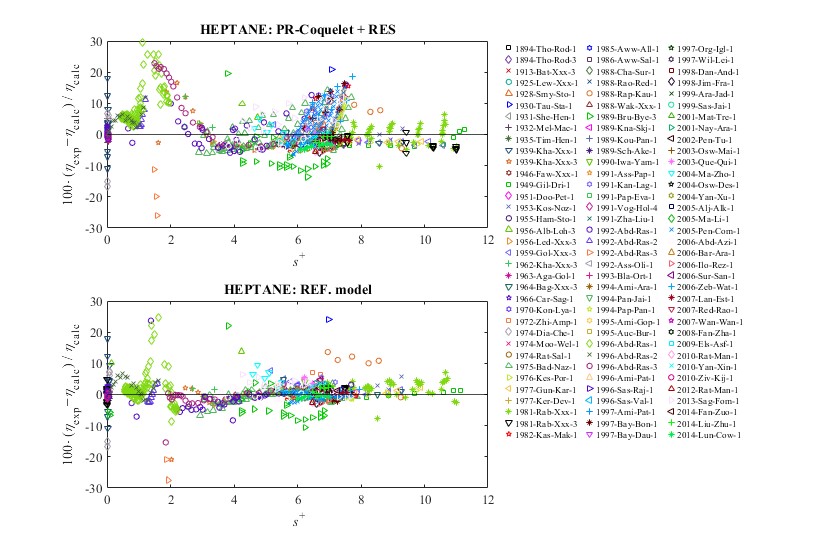

Supplement: Supplementary file 2 [file ao5c01157_si_002.zip › Supporting Information package 2/Figures/Deviation plots/PR-Coquelet/HEPTANE.jpeg]

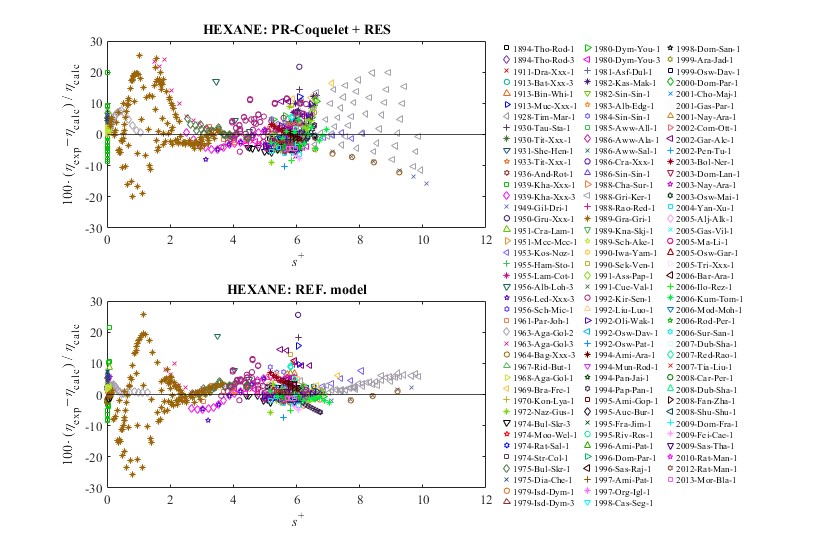

Supplement: Supplementary file 2 [file ao5c01157_si_002.zip › Supporting Information package 2/Figures/Deviation plots/PR-Coquelet/HEXANE.jpeg]

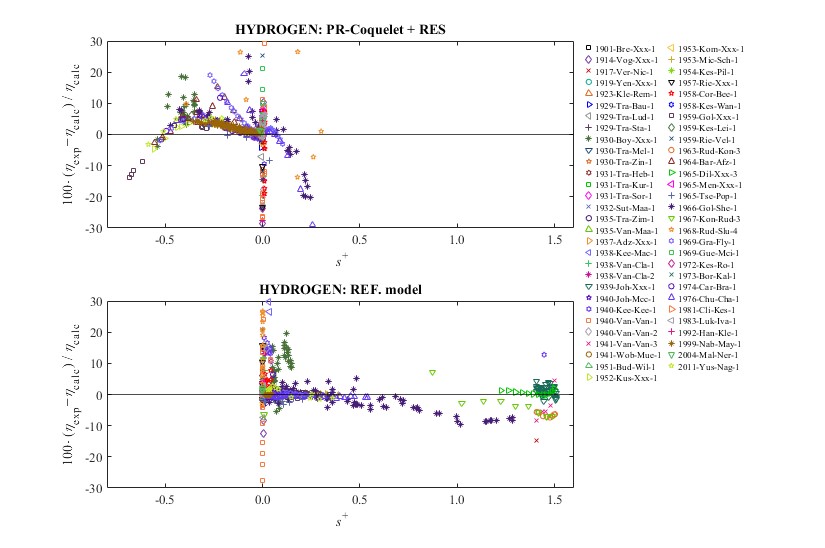

Supplement: Supplementary file 2 [file ao5c01157_si_002.zip › Supporting Information package 2/Figures/Deviation plots/PR-Coquelet/HYDROGEN.jpeg]

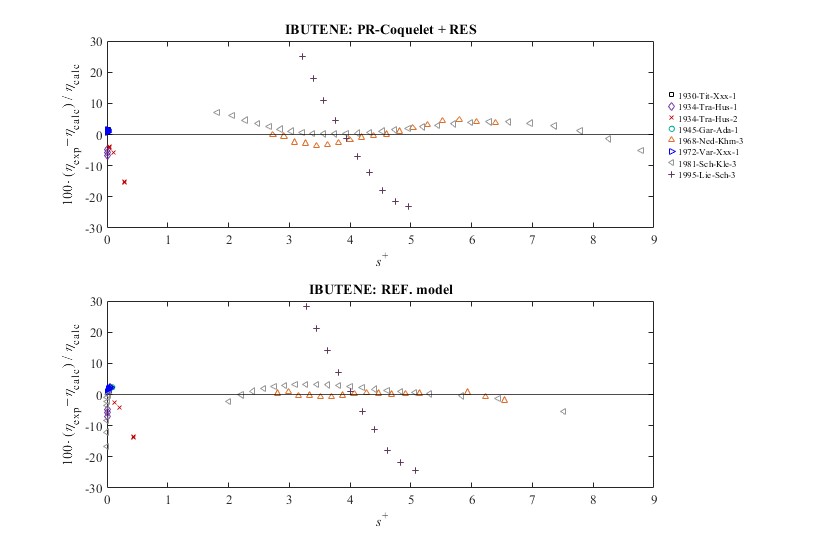

Supplement: Supplementary file 2 [file ao5c01157_si_002.zip › Supporting Information package 2/Figures/Deviation plots/PR-Coquelet/IBUTENE.jpeg]

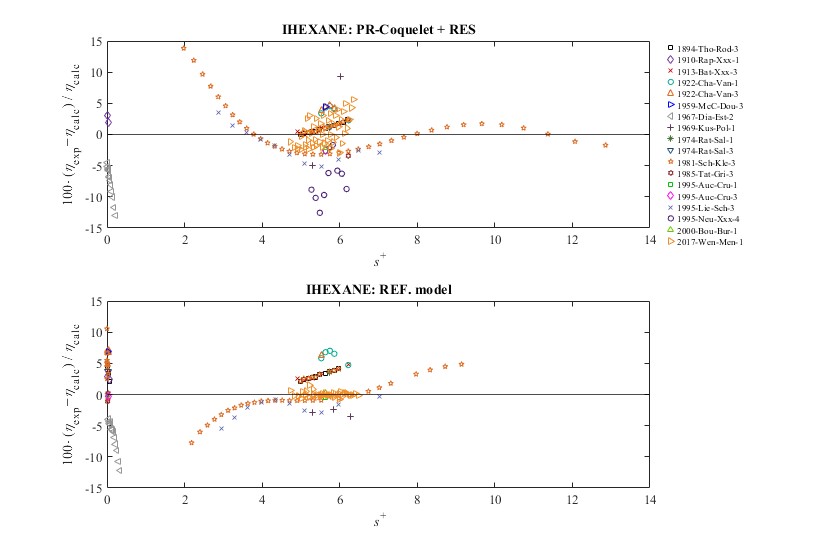

Supplement: Supplementary file 2 [file ao5c01157_si_002.zip › Supporting Information package 2/Figures/Deviation plots/PR-Coquelet/IHEXANE.jpeg]

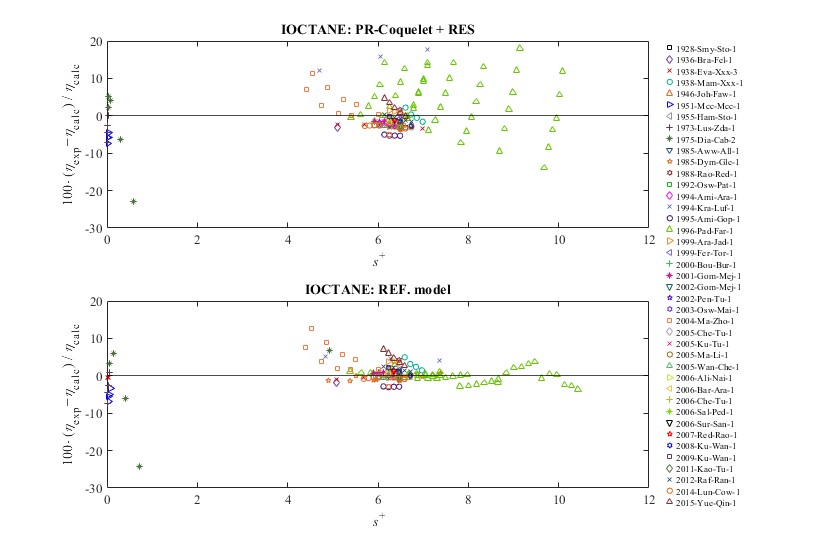

Supplement: Supplementary file 2 [file ao5c01157_si_002.zip › Supporting Information package 2/Figures/Deviation plots/PR-Coquelet/IOCTANE.jpeg]

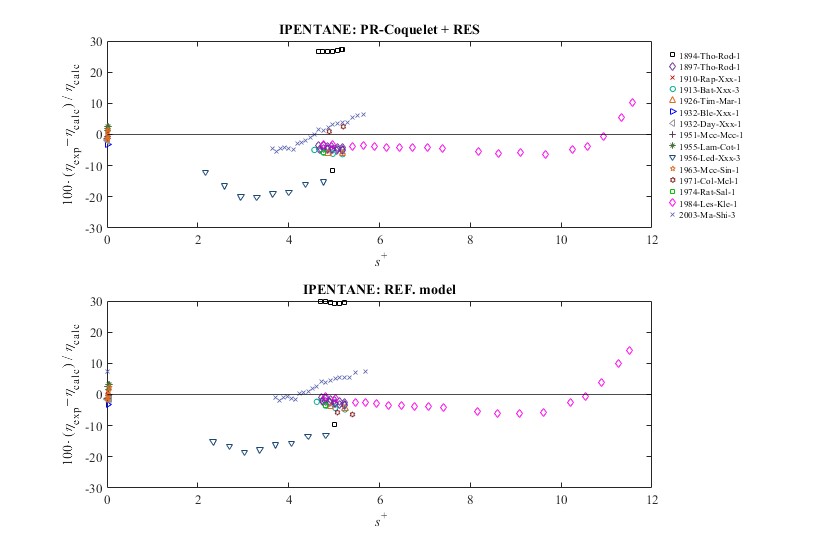

Supplement: Supplementary file 2 [file ao5c01157_si_002.zip › Supporting Information package 2/Figures/Deviation plots/PR-Coquelet/IPENTANE.jpeg]

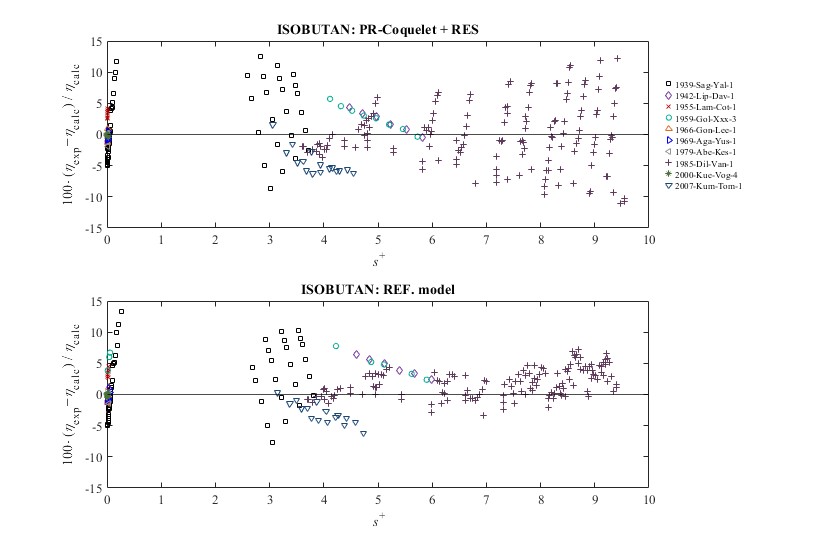

Supplement: Supplementary file 2 [file ao5c01157_si_002.zip › Supporting Information package 2/Figures/Deviation plots/PR-Coquelet/ISOBUTAN.jpeg]

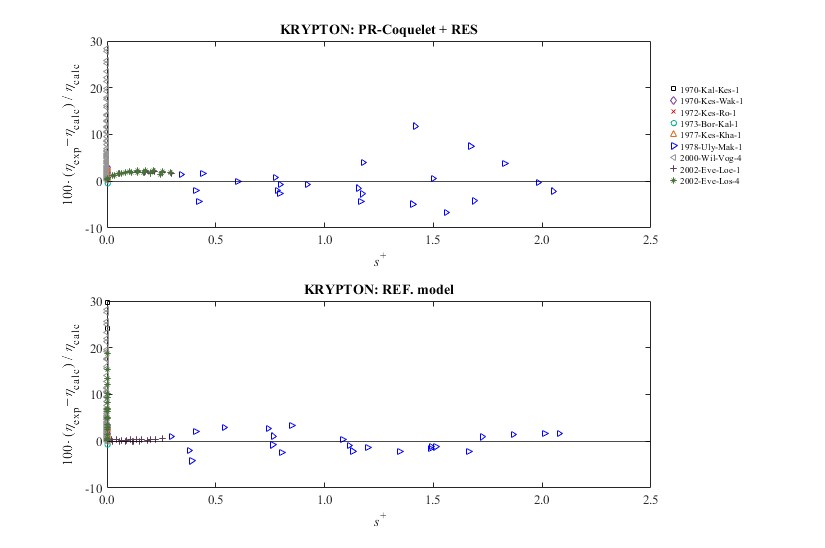

Supplement: Supplementary file 2 [file ao5c01157_si_002.zip › Supporting Information package 2/Figures/Deviation plots/PR-Coquelet/KRYPTON.jpeg]

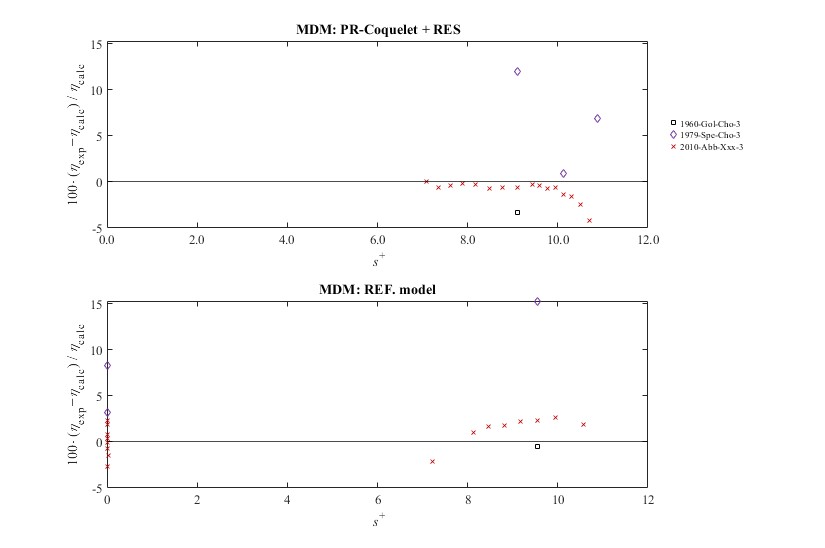

Supplement: Supplementary file 2 [file ao5c01157_si_002.zip › Supporting Information package 2/Figures/Deviation plots/PR-Coquelet/MDM.jpeg]

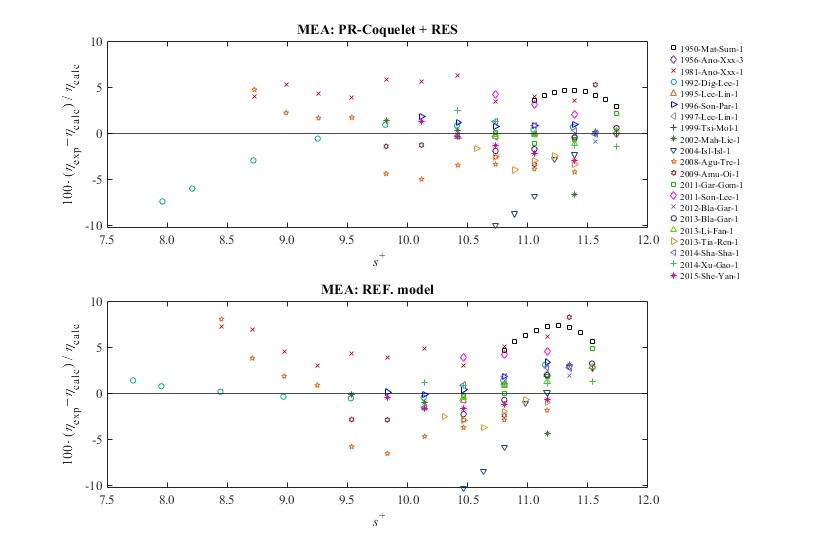

Supplement: Supplementary file 2 [file ao5c01157_si_002.zip › Supporting Information package 2/Figures/Deviation plots/PR-Coquelet/MEA.jpeg]

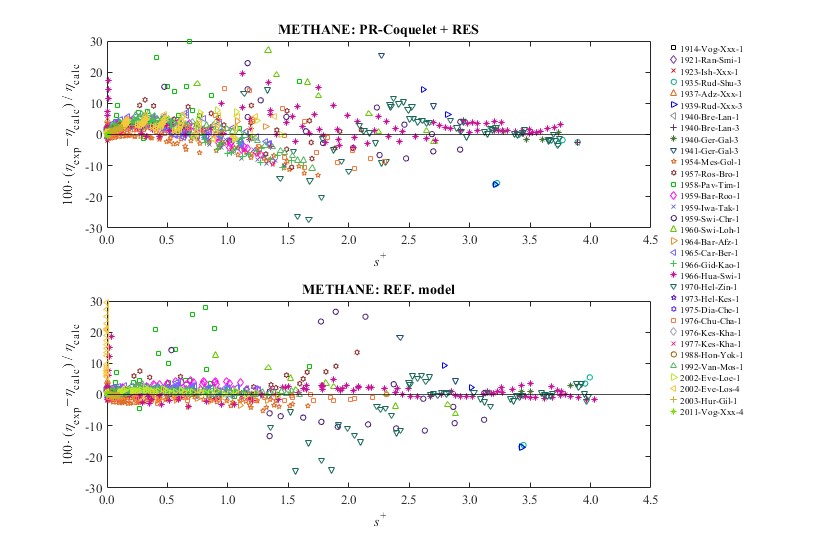

Supplement: Supplementary file 2 [file ao5c01157_si_002.zip › Supporting Information package 2/Figures/Deviation plots/PR-Coquelet/METHANE.jpeg]

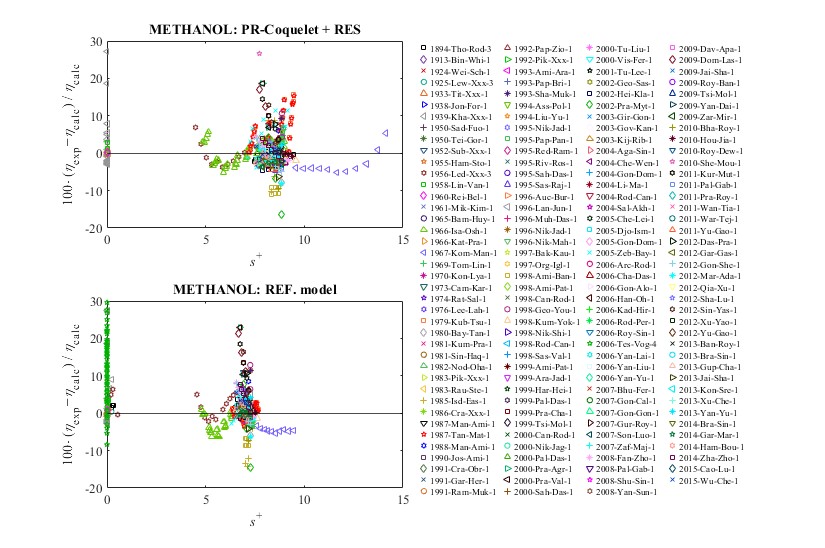

Supplement: Supplementary file 2 [file ao5c01157_si_002.zip › Supporting Information package 2/Figures/Deviation plots/PR-Coquelet/METHANOL.jpeg]

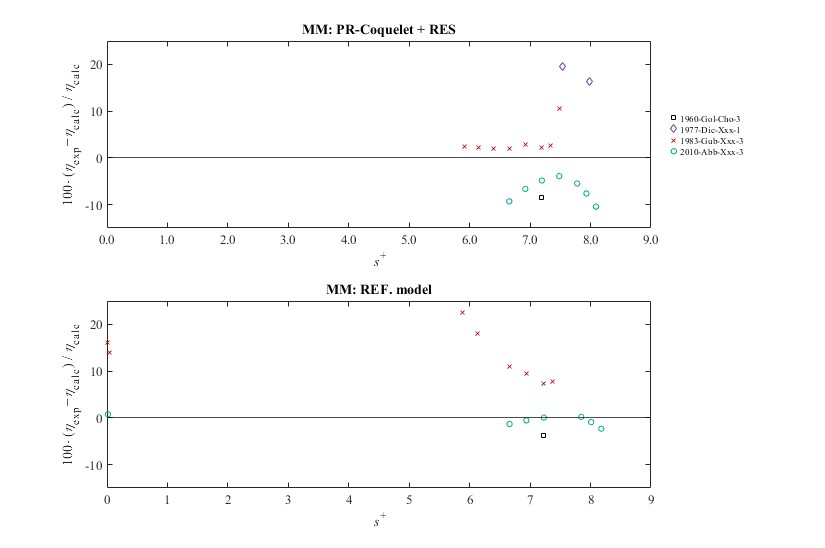

Supplement: Supplementary file 2 [file ao5c01157_si_002.zip › Supporting Information package 2/Figures/Deviation plots/PR-Coquelet/MM.jpeg]

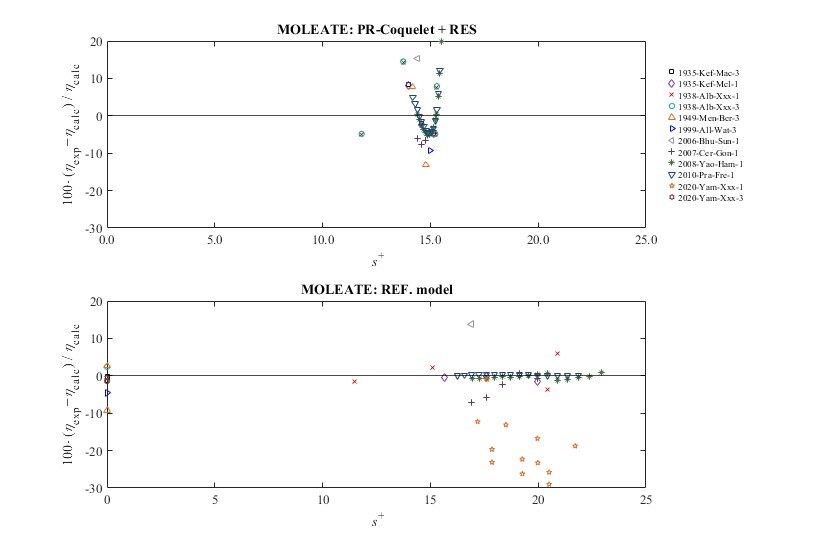

Supplement: Supplementary file 2 [file ao5c01157_si_002.zip › Supporting Information package 2/Figures/Deviation plots/PR-Coquelet/MOLEATE.jpeg]

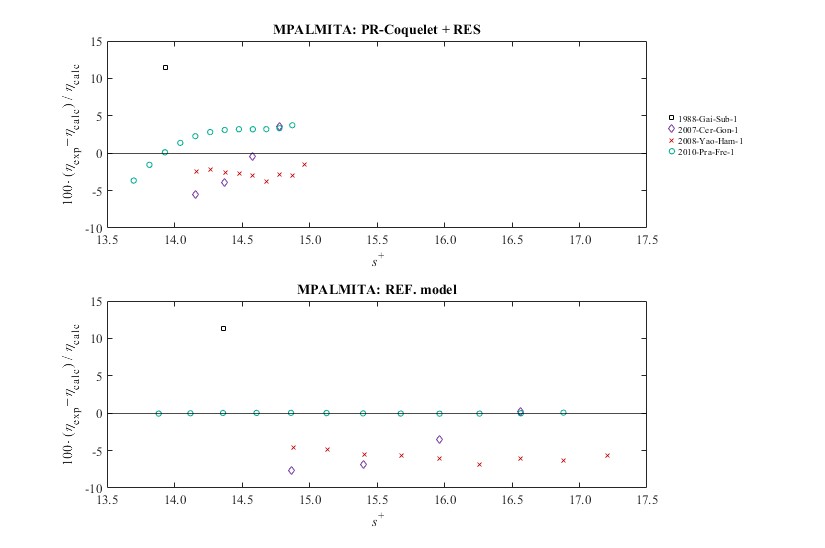

Supplement: Supplementary file 2 [file ao5c01157_si_002.zip › Supporting Information package 2/Figures/Deviation plots/PR-Coquelet/MPALMITA.jpeg]

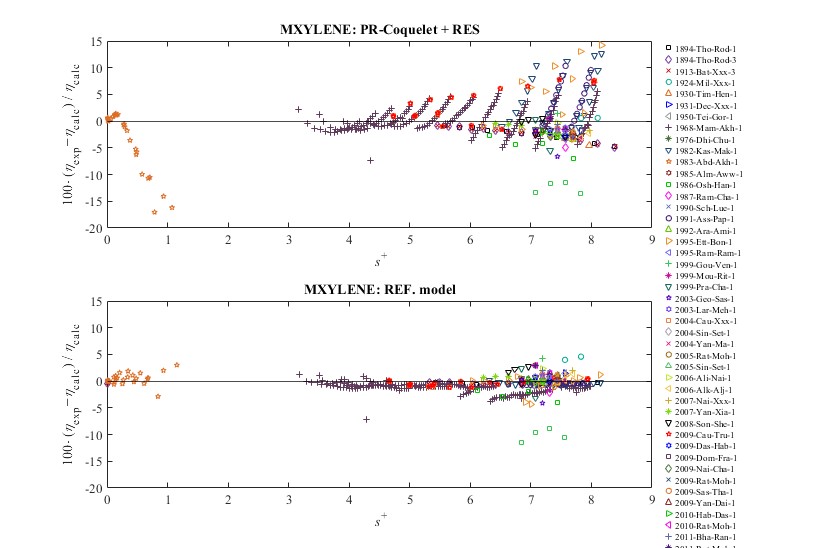

Supplement: Supplementary file 2 [file ao5c01157_si_002.zip › Supporting Information package 2/Figures/Deviation plots/PR-Coquelet/MXYLENE.jpeg]

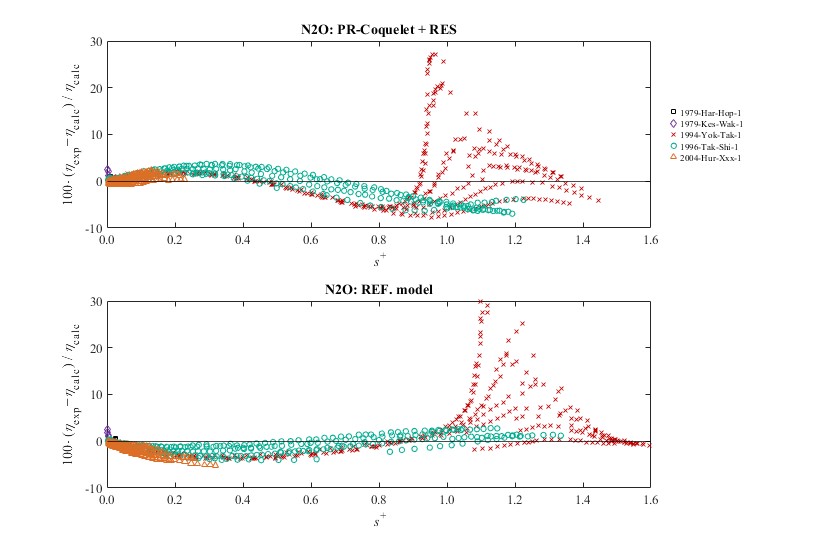

Supplement: Supplementary file 2 [file ao5c01157_si_002.zip › Supporting Information package 2/Figures/Deviation plots/PR-Coquelet/N2O.jpeg]

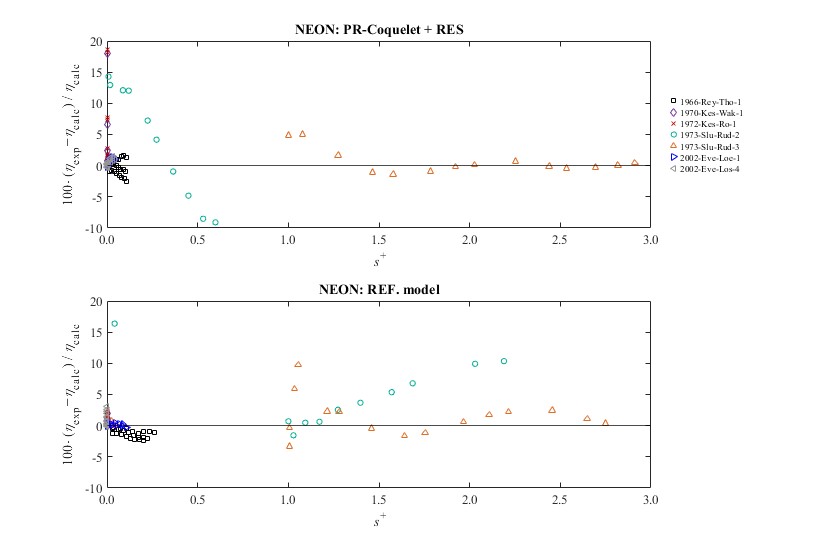

Supplement: Supplementary file 2 [file ao5c01157_si_002.zip › Supporting Information package 2/Figures/Deviation plots/PR-Coquelet/NEON.jpeg]

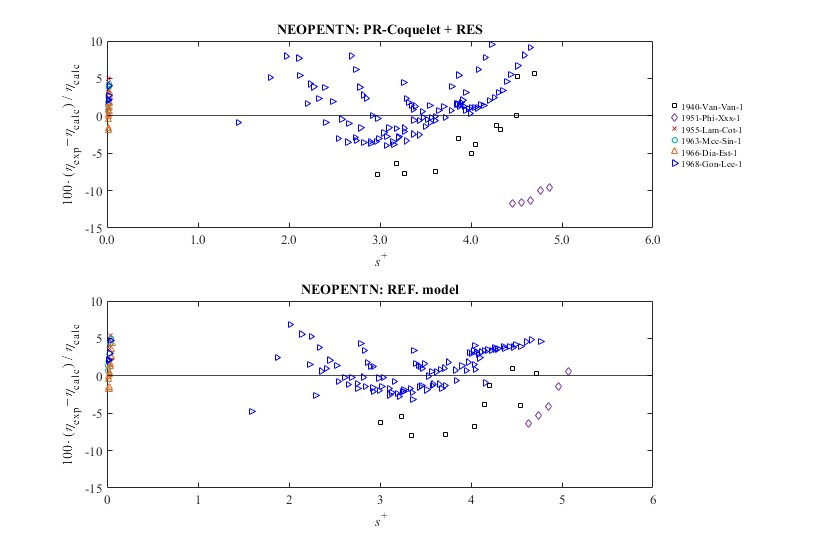

Supplement: Supplementary file 2 [file ao5c01157_si_002.zip › Supporting Information package 2/Figures/Deviation plots/PR-Coquelet/NEOPENTN.jpeg]

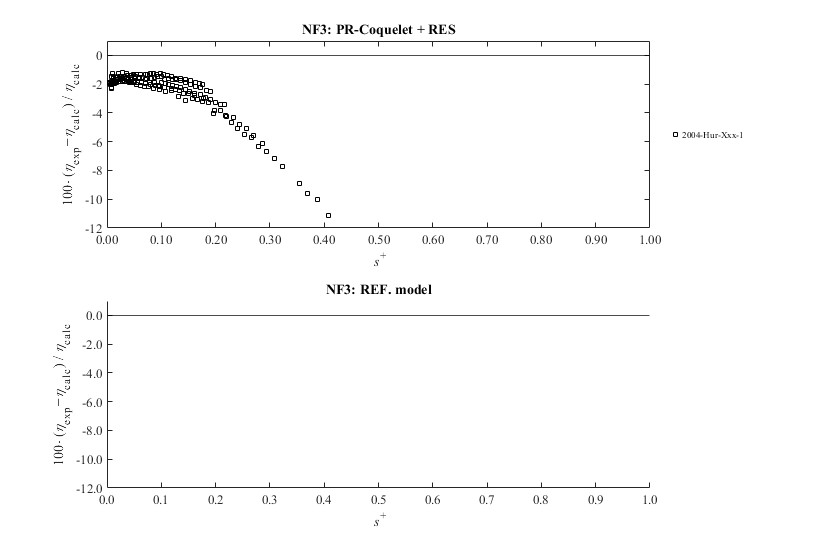

Supplement: Supplementary file 2 [file ao5c01157_si_002.zip › Supporting Information package 2/Figures/Deviation plots/PR-Coquelet/NF3.jpeg]

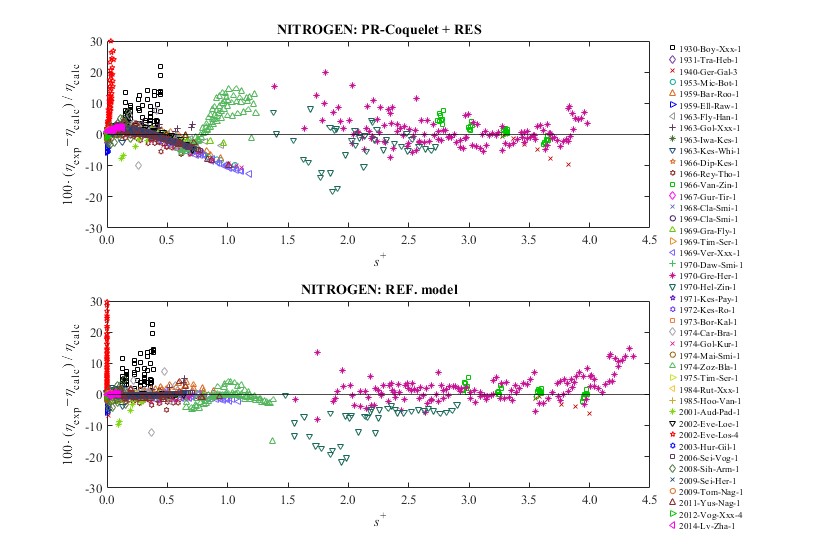

Supplement: Supplementary file 2 [file ao5c01157_si_002.zip › Supporting Information package 2/Figures/Deviation plots/PR-Coquelet/NITROGEN.jpeg]

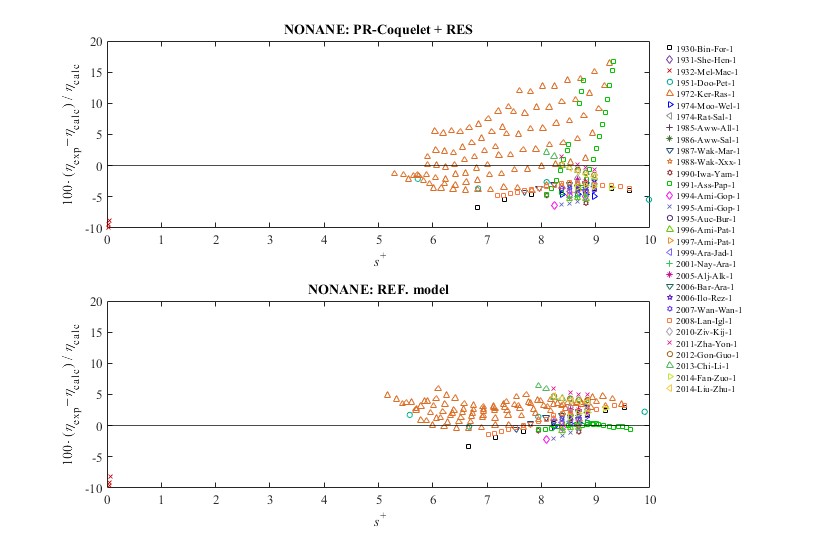

Supplement: Supplementary file 2 [file ao5c01157_si_002.zip › Supporting Information package 2/Figures/Deviation plots/PR-Coquelet/NONANE.jpeg]

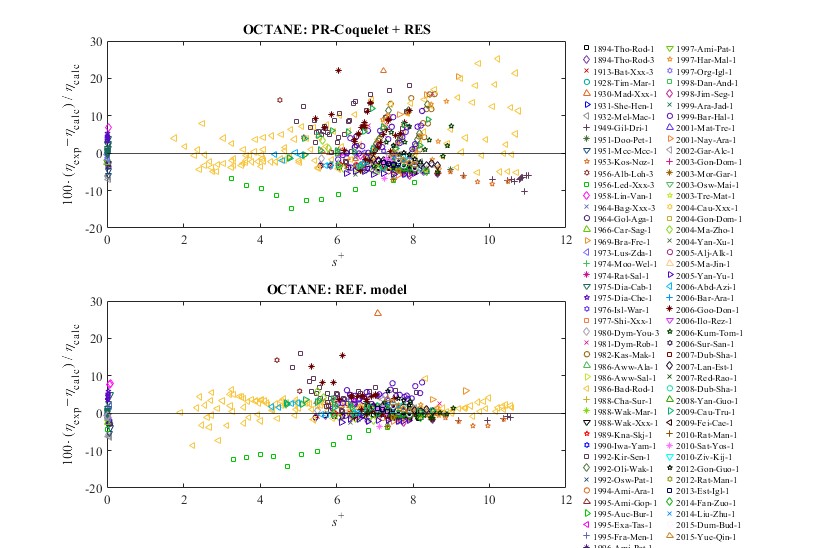

Supplement: Supplementary file 2 [file ao5c01157_si_002.zip › Supporting Information package 2/Figures/Deviation plots/PR-Coquelet/OCTANE.jpeg]

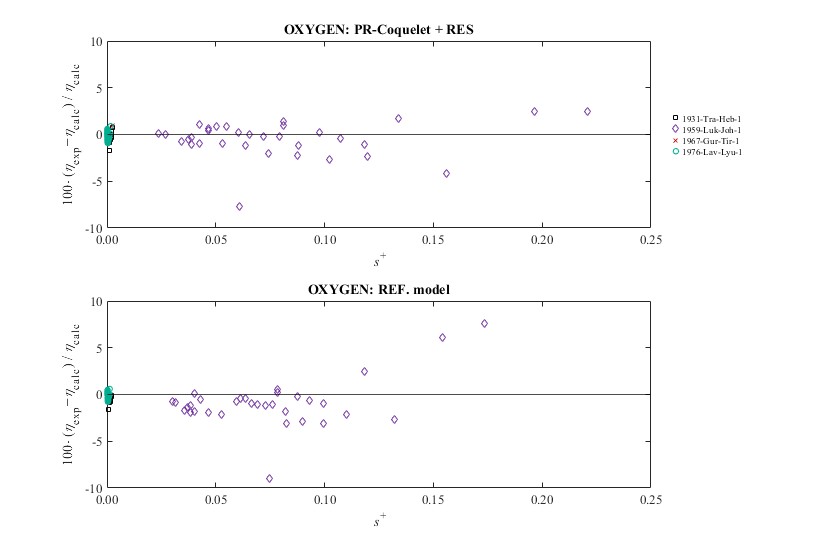

Supplement: Supplementary file 2 [file ao5c01157_si_002.zip › Supporting Information package 2/Figures/Deviation plots/PR-Coquelet/OXYGEN.jpeg]

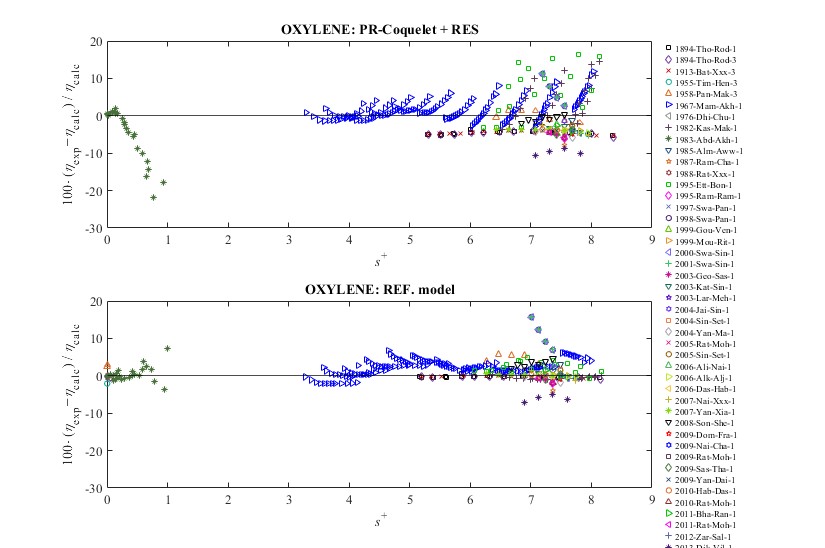

Supplement: Supplementary file 2 [file ao5c01157_si_002.zip › Supporting Information package 2/Figures/Deviation plots/PR-Coquelet/OXYLENE.jpeg]

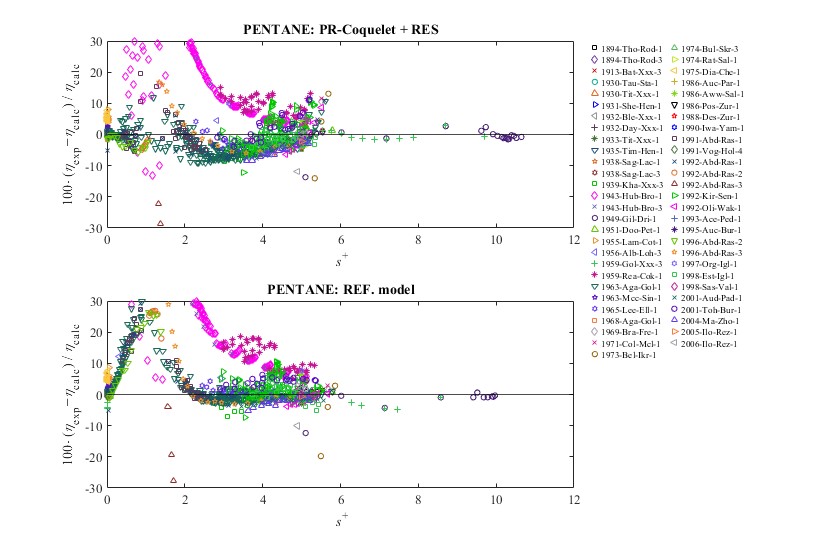

Supplement: Supplementary file 2 [file ao5c01157_si_002.zip › Supporting Information package 2/Figures/Deviation plots/PR-Coquelet/PENTANE.jpeg]

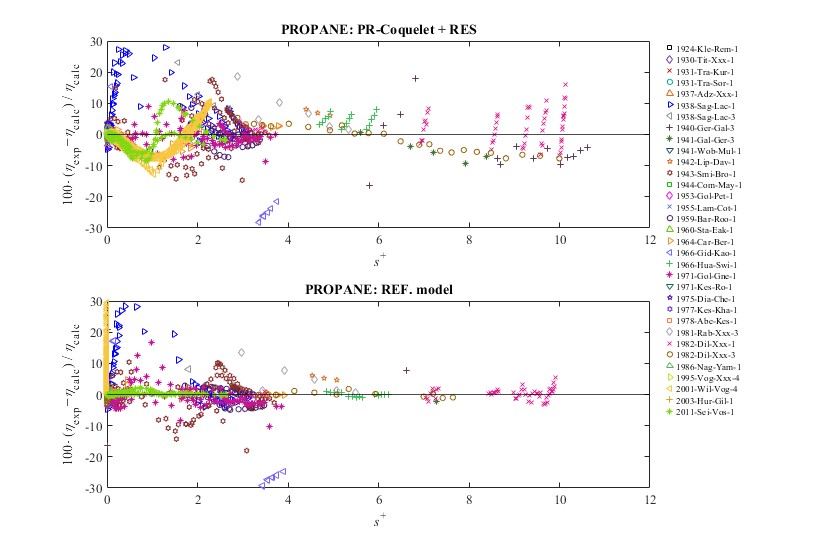

Supplement: Supplementary file 2 [file ao5c01157_si_002.zip › Supporting Information package 2/Figures/Deviation plots/PR-Coquelet/PROPANE.jpeg]

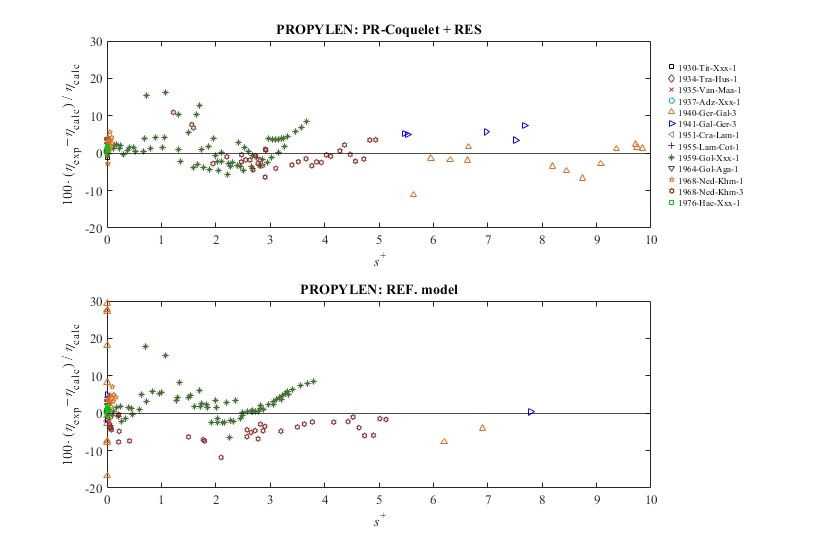

Supplement: Supplementary file 2 [file ao5c01157_si_002.zip › Supporting Information package 2/Figures/Deviation plots/PR-Coquelet/PROPYLEN.jpeg]

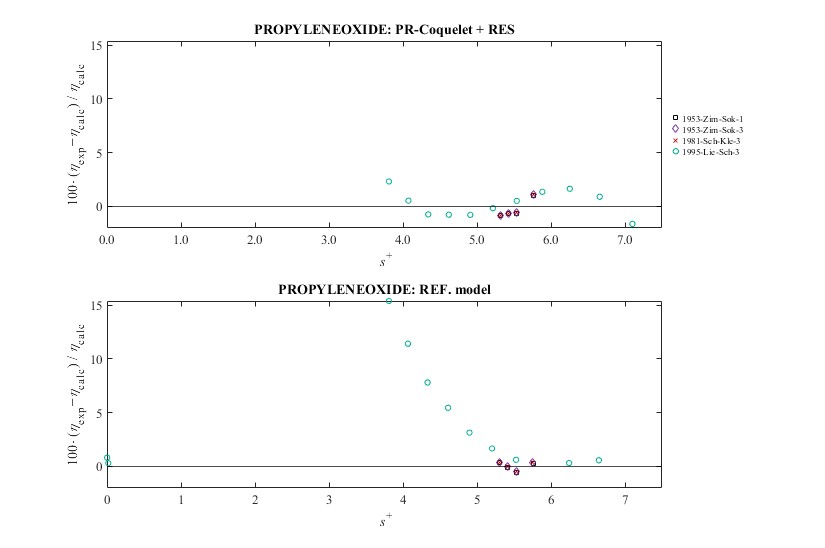

Supplement: Supplementary file 2 [file ao5c01157_si_002.zip › Supporting Information package 2/Figures/Deviation plots/PR-Coquelet/PROPYLENEOXIDE.jpeg]

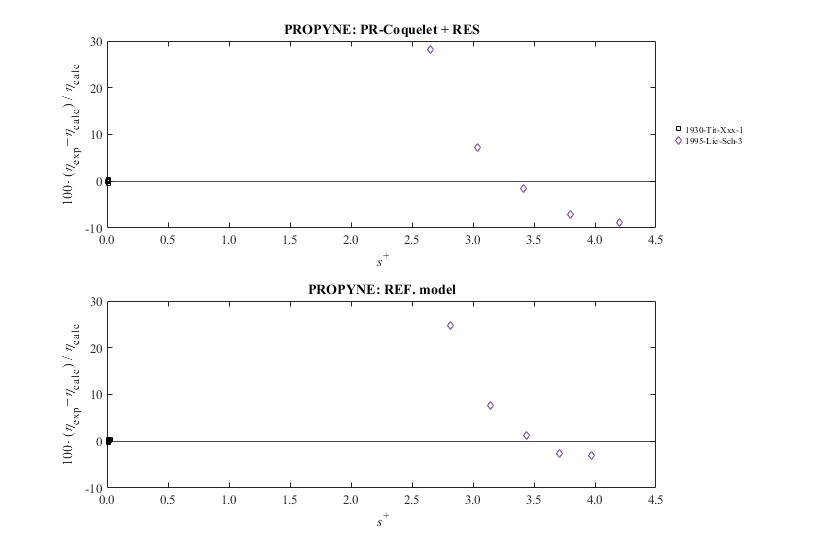

Supplement: Supplementary file 2 [file ao5c01157_si_002.zip › Supporting Information package 2/Figures/Deviation plots/PR-Coquelet/PROPYNE.jpeg]

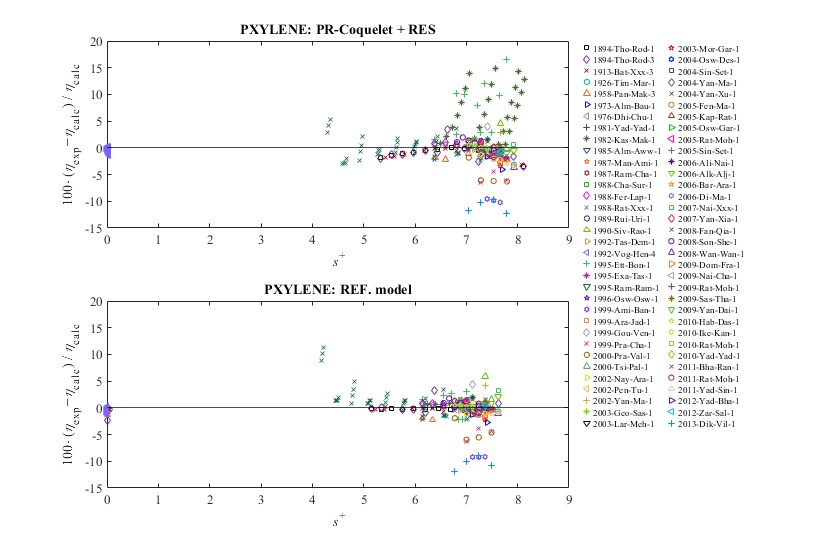

Supplement: Supplementary file 2 [file ao5c01157_si_002.zip › Supporting Information package 2/Figures/Deviation plots/PR-Coquelet/PXYLENE.jpeg]

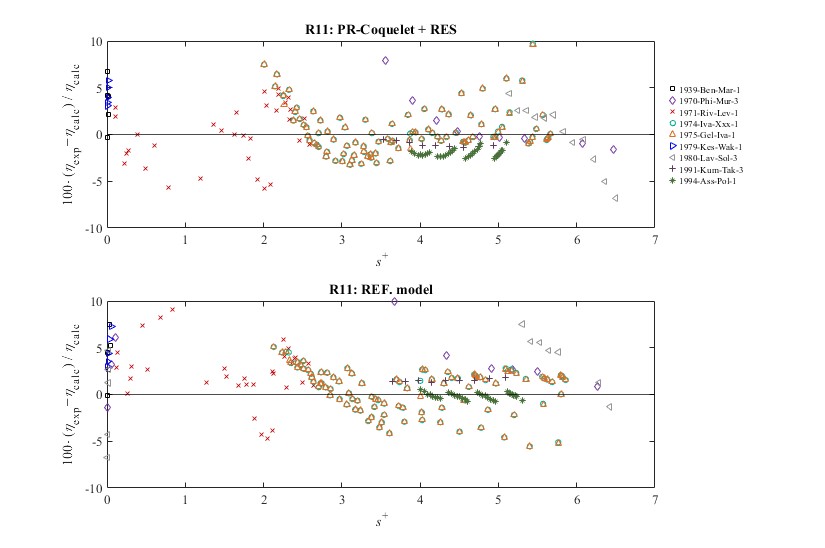

Supplement: Supplementary file 2 [file ao5c01157_si_002.zip › Supporting Information package 2/Figures/Deviation plots/PR-Coquelet/R11.jpeg]

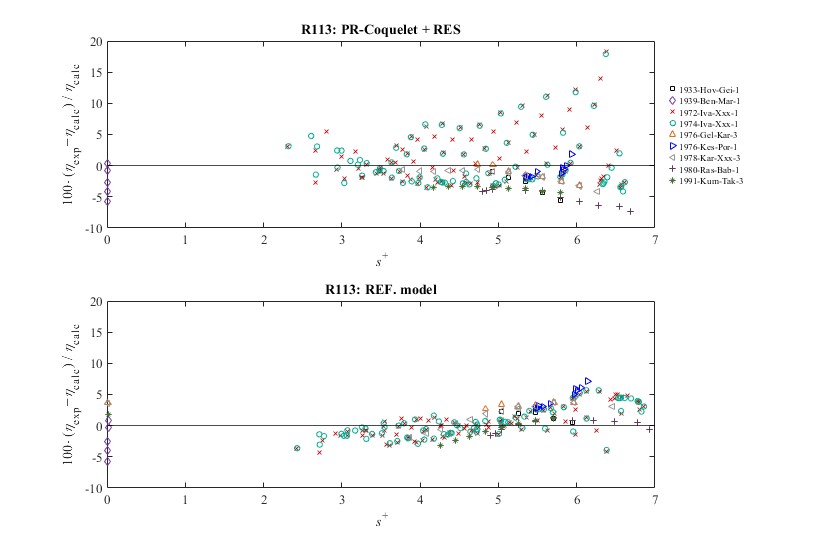

Supplement: Supplementary file 2 [file ao5c01157_si_002.zip › Supporting Information package 2/Figures/Deviation plots/PR-Coquelet/R113.jpeg]

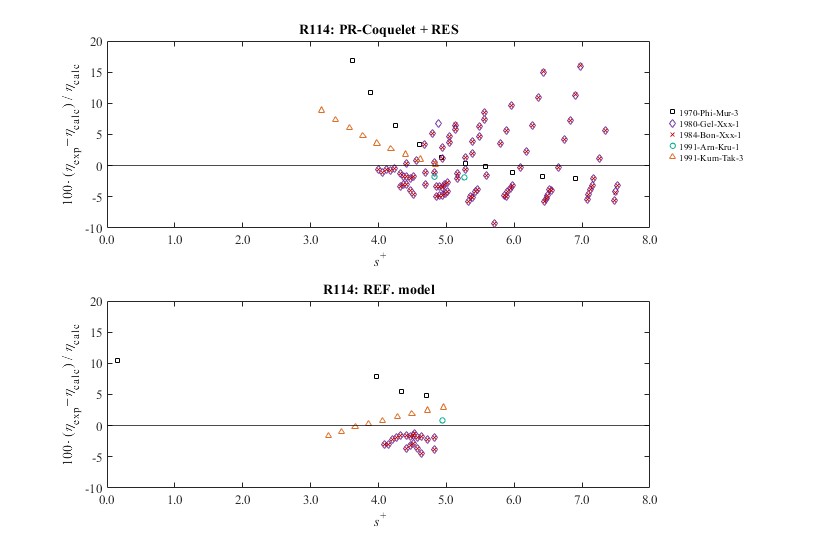

Supplement: Supplementary file 2 [file ao5c01157_si_002.zip › Supporting Information package 2/Figures/Deviation plots/PR-Coquelet/R114.jpeg]

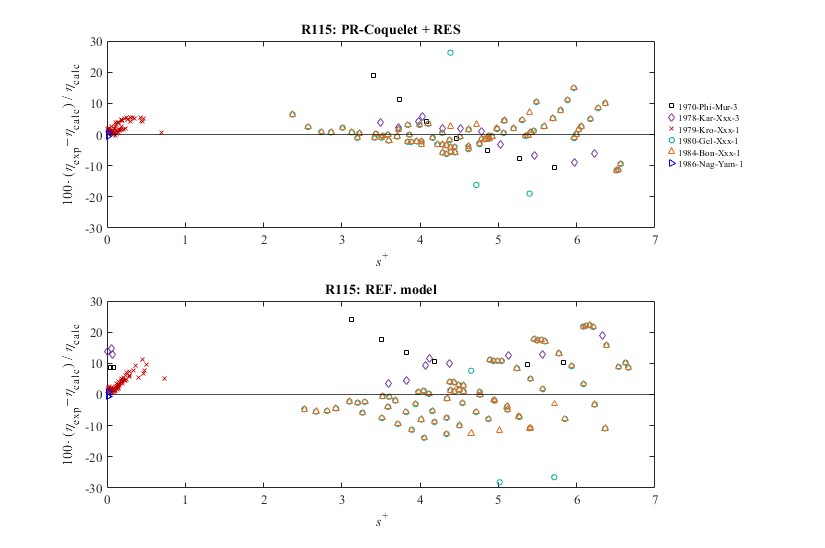

Supplement: Supplementary file 2 [file ao5c01157_si_002.zip › Supporting Information package 2/Figures/Deviation plots/PR-Coquelet/R115.jpeg]

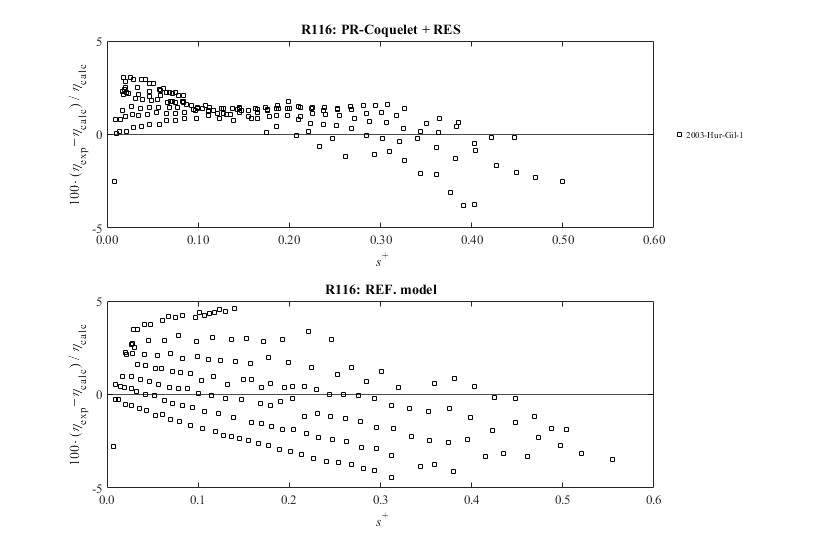

Supplement: Supplementary file 2 [file ao5c01157_si_002.zip › Supporting Information package 2/Figures/Deviation plots/PR-Coquelet/R116.jpeg]

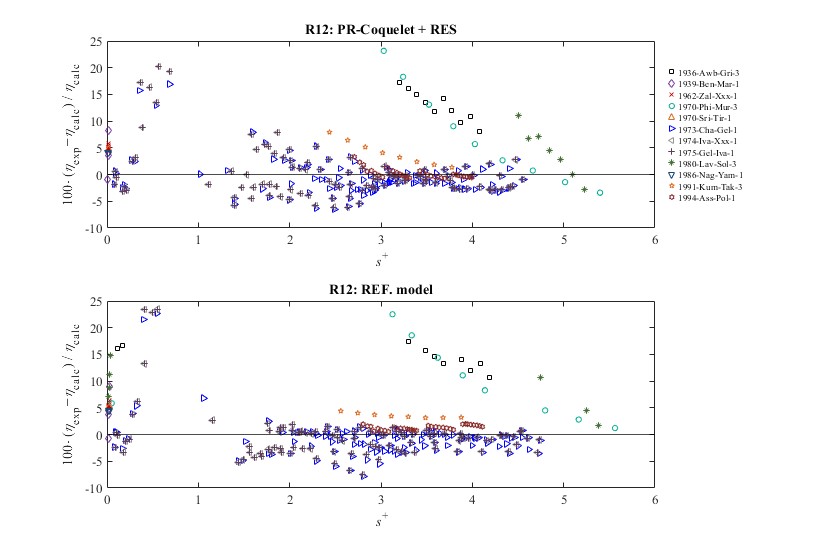

Supplement: Supplementary file 2 [file ao5c01157_si_002.zip › Supporting Information package 2/Figures/Deviation plots/PR-Coquelet/R12.jpeg]

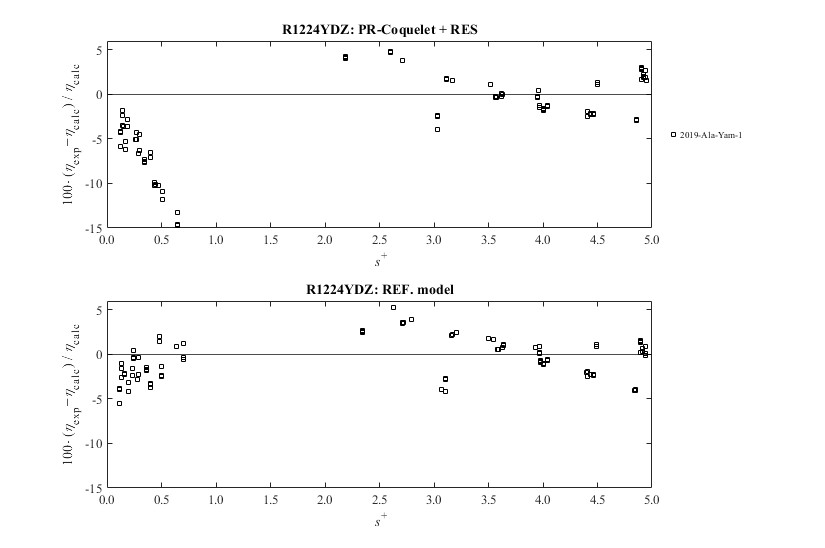

Supplement: Supplementary file 2 [file ao5c01157_si_002.zip › Supporting Information package 2/Figures/Deviation plots/PR-Coquelet/R1224YDZ.jpeg]

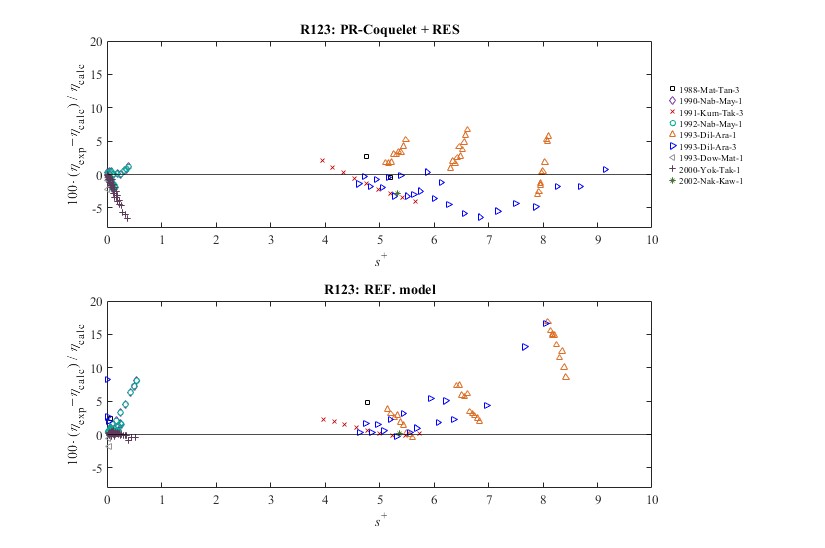

Supplement: Supplementary file 2 [file ao5c01157_si_002.zip › Supporting Information package 2/Figures/Deviation plots/PR-Coquelet/R123.jpeg]

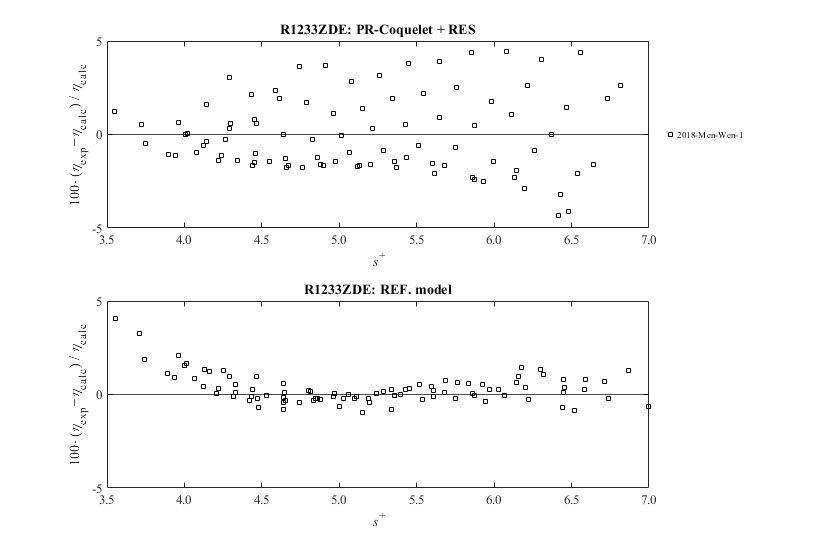

Supplement: Supplementary file 2 [file ao5c01157_si_002.zip › Supporting Information package 2/Figures/Deviation plots/PR-Coquelet/R1233ZDE.jpeg]

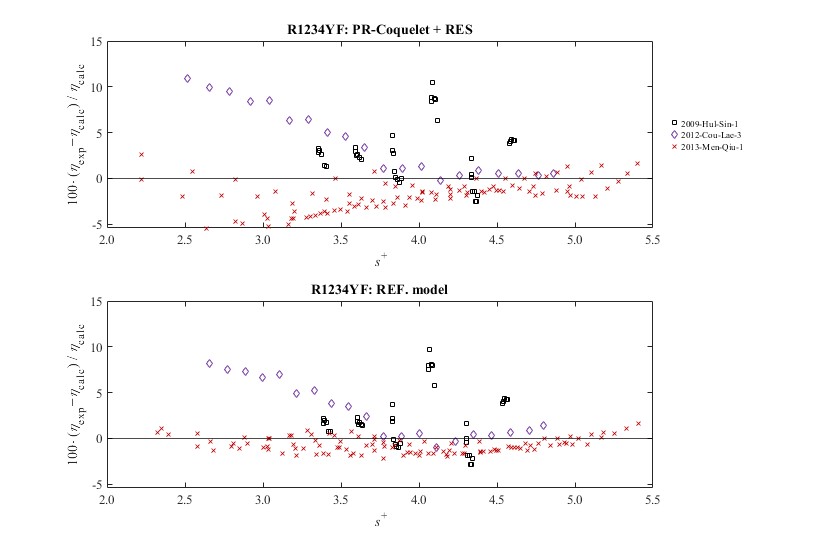

Supplement: Supplementary file 2 [file ao5c01157_si_002.zip › Supporting Information package 2/Figures/Deviation plots/PR-Coquelet/R1234YF.jpeg]

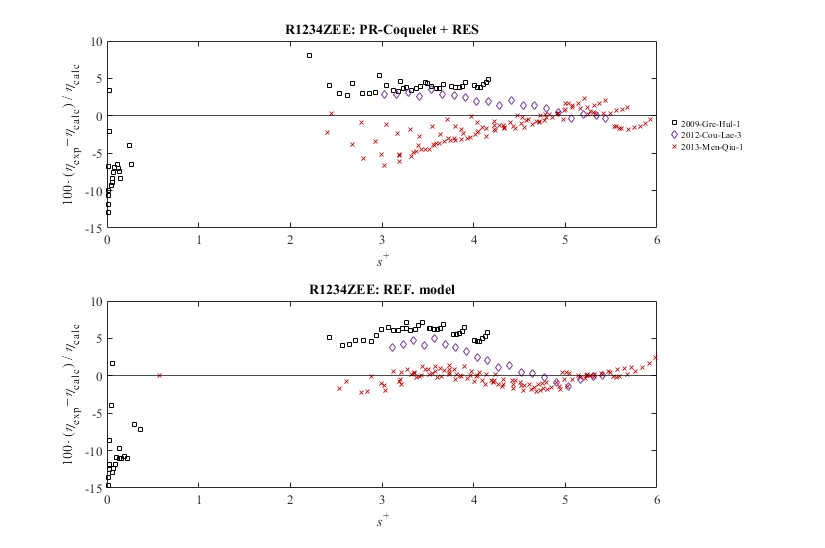

Supplement: Supplementary file 2 [file ao5c01157_si_002.zip › Supporting Information package 2/Figures/Deviation plots/PR-Coquelet/R1234ZEE.jpeg]

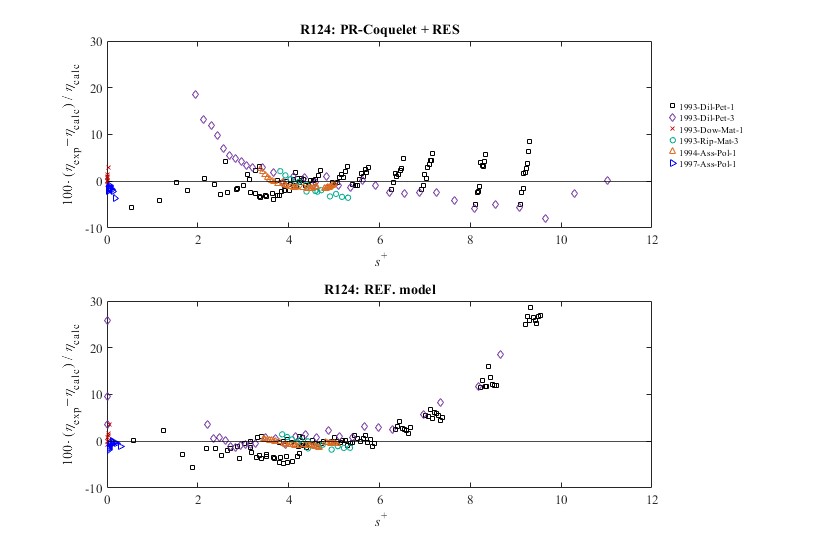

Supplement: Supplementary file 2 [file ao5c01157_si_002.zip › Supporting Information package 2/Figures/Deviation plots/PR-Coquelet/R124.jpeg]

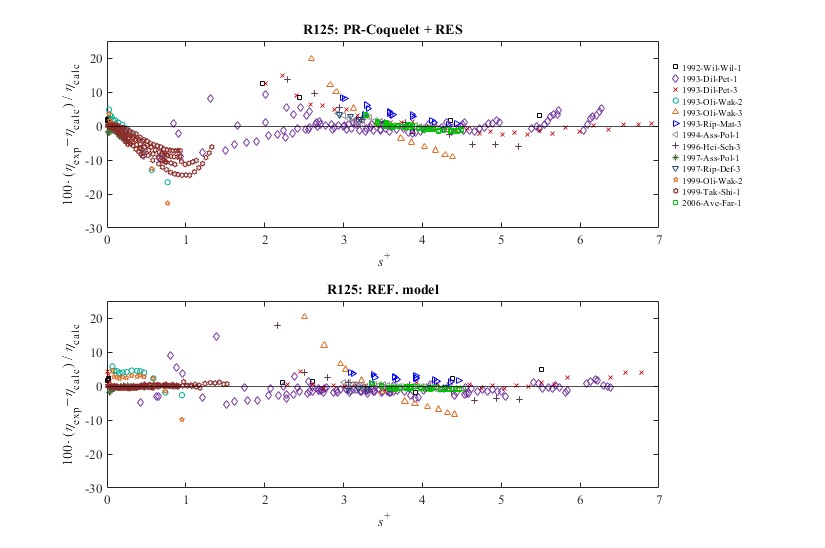

Supplement: Supplementary file 2 [file ao5c01157_si_002.zip › Supporting Information package 2/Figures/Deviation plots/PR-Coquelet/R125.jpeg]

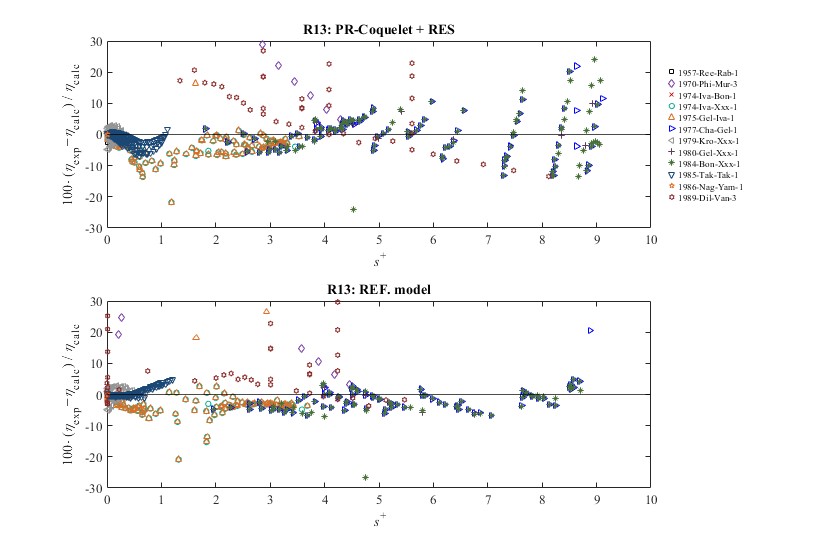

Supplement: Supplementary file 2 [file ao5c01157_si_002.zip › Supporting Information package 2/Figures/Deviation plots/PR-Coquelet/R13.jpeg]

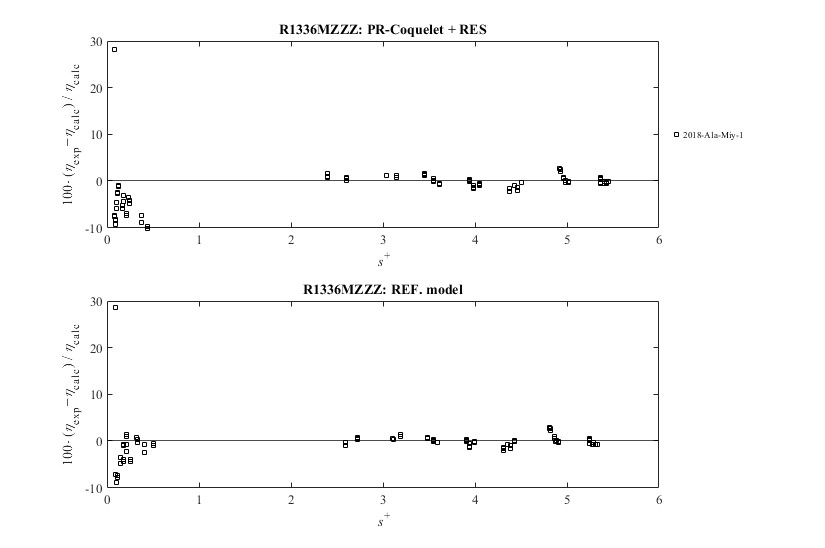

Supplement: Supplementary file 2 [file ao5c01157_si_002.zip › Supporting Information package 2/Figures/Deviation plots/PR-Coquelet/R1336MZZZ.jpeg]

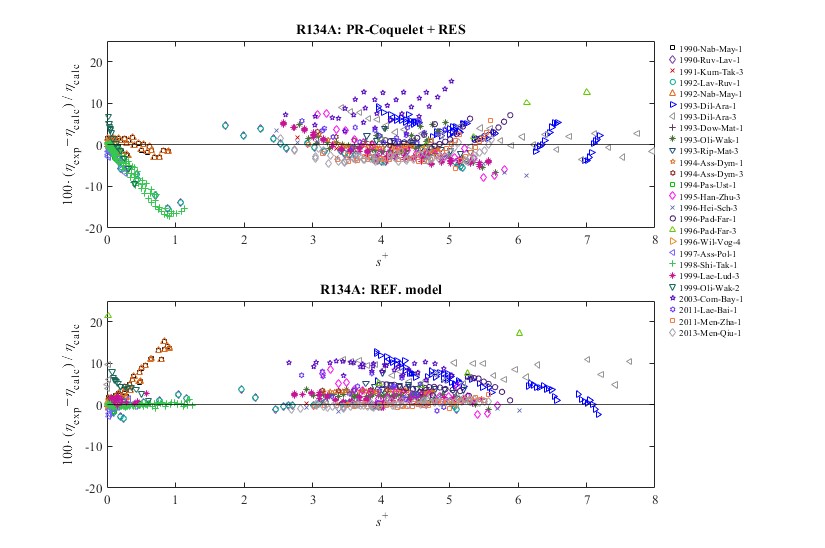

Supplement: Supplementary file 2 [file ao5c01157_si_002.zip › Supporting Information package 2/Figures/Deviation plots/PR-Coquelet/R134A.jpeg]

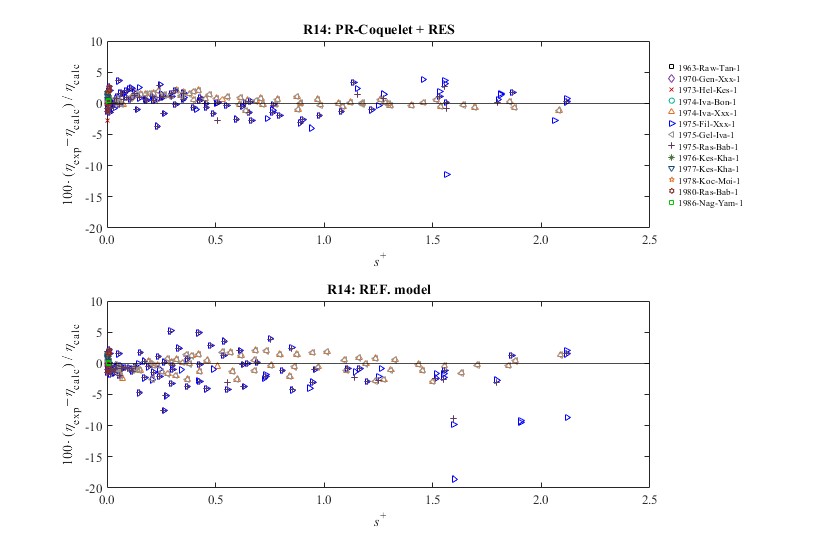

Supplement: Supplementary file 2 [file ao5c01157_si_002.zip › Supporting Information package 2/Figures/Deviation plots/PR-Coquelet/R14.jpeg]

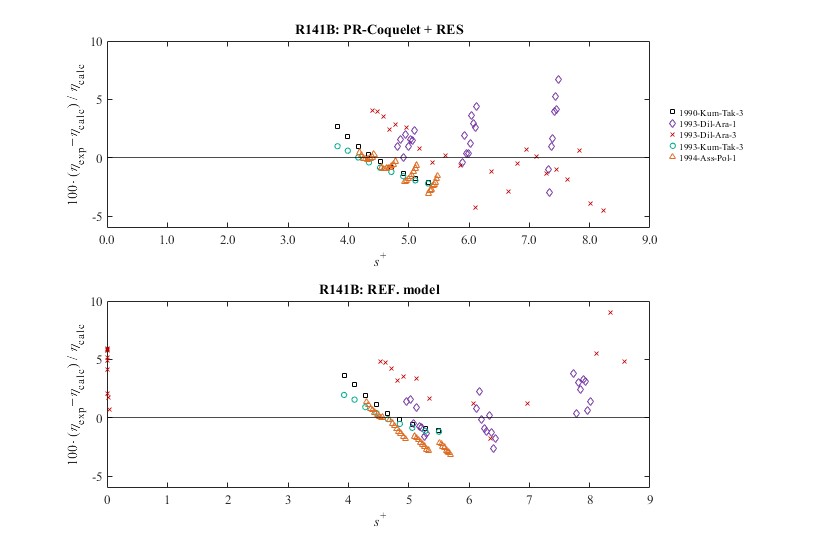

Supplement: Supplementary file 2 [file ao5c01157_si_002.zip › Supporting Information package 2/Figures/Deviation plots/PR-Coquelet/R141B.jpeg]

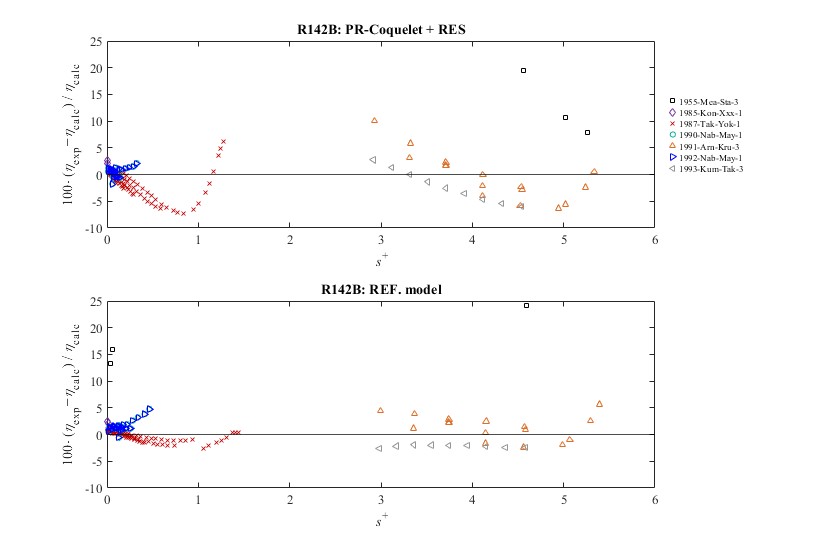

Supplement: Supplementary file 2 [file ao5c01157_si_002.zip › Supporting Information package 2/Figures/Deviation plots/PR-Coquelet/R142B.jpeg]

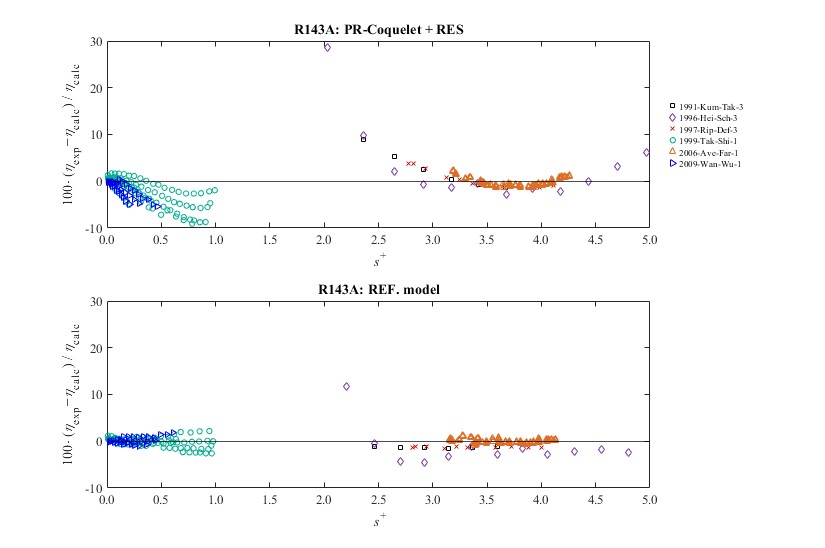

Supplement: Supplementary file 2 [file ao5c01157_si_002.zip › Supporting Information package 2/Figures/Deviation plots/PR-Coquelet/R143A.jpeg]

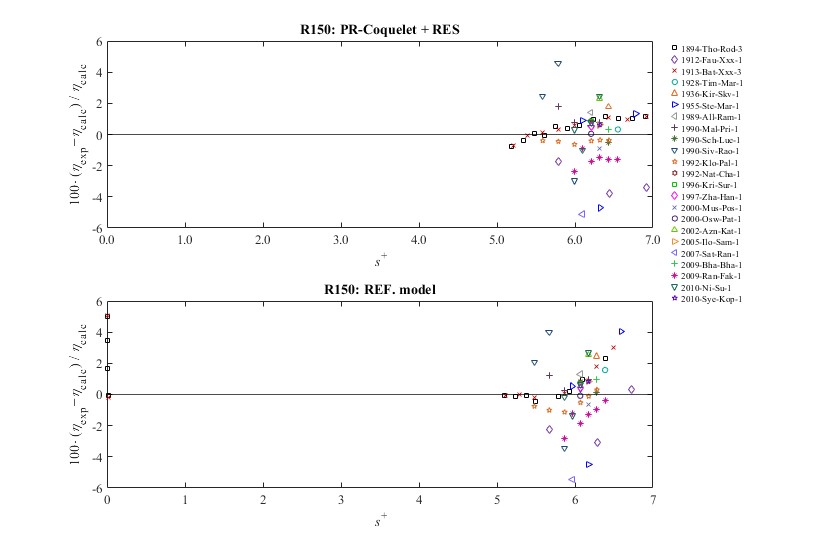

Supplement: Supplementary file 2 [file ao5c01157_si_002.zip › Supporting Information package 2/Figures/Deviation plots/PR-Coquelet/R150.jpeg]

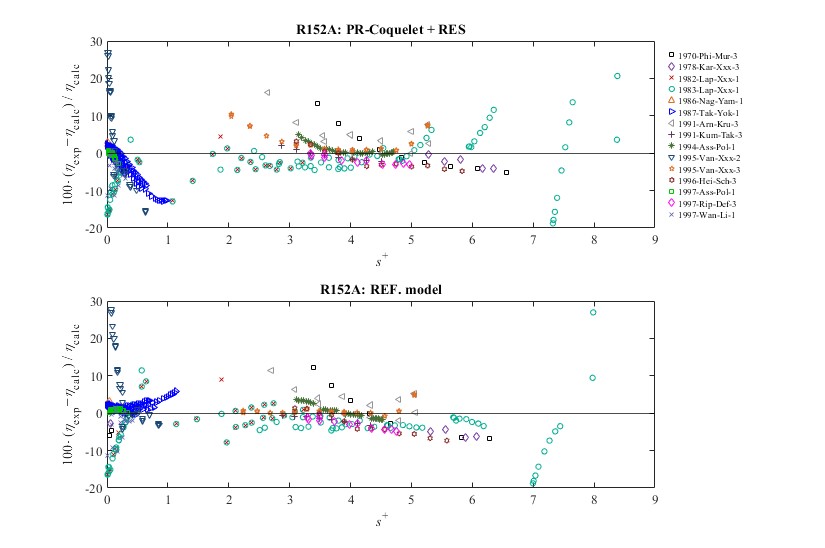

Supplement: Supplementary file 2 [file ao5c01157_si_002.zip › Supporting Information package 2/Figures/Deviation plots/PR-Coquelet/R152A.jpeg]
