# Supplementary material for: Evaluating Cubic Equations of State with Various α Functions for Viscosity Predictions of 124 Industrial Important Fluids Based on Residual Entropy Scaling
Source: ACS Omega. 2025 Jun 27;10(27):29021–36. doi: 10.1021/acsomega.5c01157 (PMC12268422; doi:10.1021/acsomega.5c01157)
Supplement: Supplementary file 1 [file ao5c01157_si_001.zip › Supporting Information package 1/Fluid names.docx]

Table S1. List of REFPROP Names, IUPAC Names, and CAS Numbers for the Components Studied in This Work.

| REFPROP name | IUPAC name | CAS number |
| --- | --- | --- |
| 13BUTADIENE | buta-1,3-diene | 106-99-0 |
| 1BUTENE | 1-butene | 106-98-9 |
| 22DIMETHYLBUTANE | 2,2-dimethylbutane | 75-83-2 |
| 23DIMETHYLBUTANE | 2,3-dimethylbutane | 79-29-8 |
| 3METHYLPENTANE | 3-methylpentane | 96-14-0 |
| ACETONE | propanone | 67-64-1 |
| ACETYLENE | ethyne | 74-86-2 |
| AMMONIA | ammonia | 7664-41-7 |
| ARGON | argon | 7440-37-1 |
| BENZENE | benzene | 71-43-2 |
| BUTANE | n-butane | 106-97-8 |
| C11 | undecane | 1120-21-4 |
| C12 | dodecane | 112-40-3 |
| C16 | hexadecane | 544-76-3 |
| C1CC6 | methylcyclohexane | 108-87-2 |
| C22 | docosane | 629-97-0 |
| C2BUTENE | cis-2-butene | 590-18-1 |
| C5F12 | dodecafluoropentane | 678-26-2 |
| C6F14 | tetradecafluorohexane | 355-42-0 |
| CF3I | trifluoroiodomethane | 2314-97-8 |
| CHLORINE | chlorine | 7782-50-5 |
| CHLOROBENZENE | chlorobenzene | 108-90-7 |
| CO | carbon monoxide | 630-08-0 |
| CO2 | carbon dioxide | 124-38-9 |
| CYCLOHEX | cyclohexane | 110-82-7 |
| CYCLOPEN | cyclopentane | 287-92-3 |
| CYCLOPRO | cyclopropane | 75-19-4 |
| D2 | deuterium | 7782-39-0 |
| D2O | deuterium oxide | 7789-20-0 |
| D4 | octamethylcyclotetrasiloxane | 556-67-2 |
| D5 | decamethylcyclopentasiloxane | 541-02-6 |
| DEA | 2,2’-iminodiethanol | 111-42-2 |
| DECANE | decane | 124-18-5 |
| DEE | diethyl ether | 60-29-7 |
| DMC | dimethyl ester carbonic acid | 616-38-6 |
| DME | methoxymethane | 115-10-6 |
| EBENZENE | phenylethane | 100-41-4 |
| EGLYCOL | 1,2-ethandiol | 107-21-1 |
| ETHANE | ethane | 74-84-0 |
| ETHANOL | ethyl alcohol | 64-17-5 |
| ETHYLENE | ethene | 74-85-1 |
| ETHYLENEOXIDE | ethylene oxide | 75-21-8 |
| FLUORINE | fluorine | 7782-41-4 |
| H2S | hydrogen sulfide | 7783-06-4 |
| HCL | hydrogen chloride | 7647-01-0 |
| HELIUM | helium-4 | 7440-59-7 |
| HEPTANE | heptane | 142-82-5 |
| HEXANE | hexane | 110-54-3 |
| HYDROGEN | hydrogen (normal) | 1333-74-0 |
| IBUTENE | 2-methyl-1-propene | 115-11-7 |
| IHEXANE | 2-methylpentane | 107-83-5 |
| IOCTANE | 2,2,4-trimethylpentane | 540-84-1 |
| IPENTANE | 2-methylbutane | 78-78-4 |
| ISOBUTAN | 2-methylpropane | 75-28-5 |
| KRYPTON | krypton | 7439-90-9 |
| MDM | octamethyltrisiloxane | 107-51-7 |
| MEA | ethanolamine | 141-43-5 |
| METHANE | methane | 74-82-8 |
| METHANOL | methanol | 67-56-1 |
| MLINOLEA | methyl (Z,Z)-9,12-octadecadienoate | 112-63-0 |
| MLINOLEN | methyl (Z,Z,Z)-9,12,15-octadecatrienoate | 301-00-8 |
| MM | hexamethyldisiloxane | 107-46-0 |
| MOLEATE | methyl cis-9-octadecenoate | 112-62-9 |
| MPALMITA | methyl hexadecanoate | 112-39-0 |
| MSTEARAT | methyl octadecanoate | 112-61-8 |
| MXYLENE | 1,3-dimethylbenzene | 108-38-3 |
| N2O | dinitrogen monoxide | 10024-97-2 |
| NEON | neon | 7440-01-9 |
| NEOPENTN | 2,2-dimethylpropane | 463-82-1 |
| NF3 | nitrogen trifluoride | 7783-54-2 |
| NITROGEN | nitrogen | 7727-37-9 |
| NONANE | nonane | 111-84-2 |
| OCTANE | octane | 111-65-9 |
| OXYGEN | oxygen | 7782-44-7 |
| OXYLENE | 1,2-dimethylbenzene | 95-47-6 |
| PENTANE | pentane | 109-66-0 |
| PROPANE | propane | 74-98-6 |
| PROPYLEN | propene | 115-07-1 |
| PROPYLENEOXIDE | 1,2-epoxypropane | 75-56-9 |
| PROPYNE | propyne | 74-99-7 |
| PXYLENE | 1,4-dimethylbenzene | 106-42-3 |
| R11 | trichlorofluoromethane | 75-69-4 |
| R113 | 1,1,2-trichloro-1,2,2-trifluoroethane | 76-13-1 |
| R114 | 1,2-dichloro-1,1,2,2-tetrafluoroethane | 76-14-2 |
| R115 | chloropentafluoroethane | 76-15-3 |
| R116 | hexafluoroethane | 76-16-4 |
| R12 | dichlorodifluoromethane | 75-71-8 |
| R1224YDZ | (Z)-1-chloro-2,3,3,3-tetrafluoropropene | 111512-60-8 |
| R123 | 2,2-dichloro-1,1,1-trifluoroethane | 306-83-2 |
| R1233ZDE | trans-1-chloro-3,3,3-trifluoro-1-propene | 102687-65-0 |
| R1234YF | 2,3,3,3-tetrafluoroprop-1-ene | 754-12-1 |
| R1234ZEE | trans-1,3,3,3-tetrafluoropropene | 29118-24-9 |
| R124 | 1-chloro-1,2,2,2-tetrafluoroethane | 2837-89-0 |
| R125 | pentafluoroethane | 354-33-6 |
| R13 | chlorotrifluoromethane | 75-72-9 |
| R1336MZZZ | (Z)-1,1,1,4,4,4-hexafluoro-2-butene | 692-49-9 |
| R134A | 1,1,1,2-tetrafluoroethane | 811-97-2 |
| R14 | tetrafluoromethane | 75-73-0 |
| R141B | 1,1-dichloro-1-fluoroethane | 1717-00-6 |
| R142B | 1-chloro-1,1-difluoroethane | 75-68-3 |
| R143A | 1,1,1-trifluoroethane | 420-46-2 |
| R150 | 1,2-dichloroethane | 107-06-2 |
| R152A | 1,1-difluoroethane | 75-37-6 |
| R161 | fluoroethane | 353-36-6 |
| R21 | dichlorofluoromethane | 75-43-4 |
| R218 | octafluoropropane | 76-19-7 |
| R22 | chlorodifluoromethane | 75-45-6 |
| R227EA | 1,1,1,2,3,3,3-heptafluoropropane | 431-89-0 |
| R23 | trifluoromethane | 75-46-7 |
| R236EA | 1,1,1,2,3,3-hexafluoropropane | 431-63-0 |
| R236FA | 1,1,1,3,3,3-hexafluoropropane | 690-39-1 |
| R245CA | 1,1,2,2,3-pentafluoropropane | 679-86-7 |
| R245FA | 1,1,1,3,3-pentafluoropropane | 460-73-1 |
| R32 | difluoromethane | 75-10-5 |
| R40 | methyl chloride | 74-87-3 |
| R41 | fluoromethane | 593-53-3 |
| RC318 | octafluorocyclobutane | 115-25-3 |
| RE245FA2 | 2,2,2-trifluoroethyl-difluoromethyl-ether | 1885-48-9 |
| RE347MCC | 1,1,1,2,2,3,3-heptafluoro-3-methoxypropane | 375-03-1 |
| SF6 | sulfur hexafluoride | 2551-62-4 |
| SO2 | sulfur dioxide | 7446-09-5 |
| TOLUENE | methylbenzene | 108-88-3 |
| WATER | water | 7732-18-5 |
| XENON | xenon | 7440-63-3 |
