# Supplementary material for: Evaluating Cubic Equations of State with Various α Functions for Viscosity Predictions of 124 Industrial Important Fluids Based on Residual Entropy Scaling
Source: ACS Omega. 2025 Jun 27;10(27):29021–36. doi: 10.1021/acsomega.5c01157 (PMC12268422; doi:10.1021/acsomega.5c01157)
Supplement: Supplementary file 1 [file ao5c01157_si_001.zip › Supporting Information package 1/README.docx]

Evaluating Cubic Equations of State with Various Alpha Functions for Viscosity Predictions of 124 Industrial Important Fluids Based on Residual Entropy Scaling

Xiong Xiao^1^, Xiaoxian Yang^1,2^*

^1^ *Fluid Science & Resources Division, Department of Chemical Engineering, The University of Western Australia, Crawley, WA 6009, Australia*

^2^ *Chemnitz University of Technology, Applied Thermodynamics, 09107 Chemnitz, Germany*

This document provides details on the extensive tables and figures included in the ‘supporting information.zip’ file published alongside the manuscript.

**Note on image resolution and content scope**

Due to file size limitations, only reduced-resolution images are included in this Supporting Information package. Additionally, to ensure the overall file remains under the size threshold, all deviation and fitting figures for the four base cubic EOS models (PR, SRK, PTV, and YFR) have been omitted. This Supporting Information contains only the summary charts and selected results for modified alpha functions, in compressed resolution.

The full-resolution dataset, including all individual EOS results and high-quality figures (PR, SRK, PTV, YFR, and modified models), is publicly available at:

<https://doi.org/10.6084/m9.figshare.28357856.v3>

Please note that the detailed descriptions below refer to the complete Supporting Information available at the figshare link above, and do not fully reflect the reduced set of content included in the version provided via the ACS Omega’s website. In particular, the PR, SRK, PTV, and YFR model plots are not present in the journal-uploaded version.

# **1. Tables**

## **1.1. Fluid names**

Location: Supporting Information/Fluid names.docx

Contains: Fluid names from REFPROP 10.0 along with the corresponding IUPAC names and CAS numbers.

## **1.2. Parameters for dilute gas viscosity calculation**

Location: Supporting Information/Parameters/Dilute gas for viscosity.txt

Contains: Parameters *a_η,i_* (*i* = 0,1,2,3,4) to be used in the eq 4 in the main manuscript, and the temperature ranges these parameters were fitted to the calculations of REFPROP 10.0.^1^

## **1.3. Fluid constants**

Location: Supporting Information/Parameters/Fluid constants.txt

Contains:

(I) Molar mass *M*, critical point information (*T*_c_, *p*_c_, *ρ*_c_), acentric factor *ω* needed in the cubic EOS calculation.

(II) L-J parameters (the pair-potential energy *ε*/*k*_B_ and the collision diameter *σ*) used for an alternative method of dilute gas property calculation.

(III) Other properties that are not used in this work.

## **1.4. RES parameters for viscosity**

Location: Supporting Information/Parameters/RES parameters for viscosity/xxx

Contains: RES parameters (*n_λ_*_,f_*_i_*, *n_λ_*_,g_*_i_* and *ξ_λ_*) of viscosity developed for each of the four cubic EOS (PR, SRK, PTV and YFR) and the modified alpha functions (PR-Twu, PTV-Heyen, SRK-Coquelet, SRK-MS, PR-Coquelet and PR-MS).

In each file: the number of experimental data used for parameter fitting (DataN), the group number of this fluid (GroupN), the group-specific parameters (n1_glb and so on), the fluid-specific parameters (n1_ind and so on) and fluid-specific scaling factor (xita) are given for each pure fluid.

# **2. Figures**

## **2.1. Residual viscosity ln(*η*^r+^+1) vs. residual entropy *s*^+^/*ξ_η_***

Location: Supporting Information/Figures/s_eta_fitted_data

Contains: Individual fit for ln($\eta_{\text{res}}^{+}$+1) vs. *s*^+^/*ξ_η_* for the pure fluids where applicable, and global for ln($\eta_{\text{res}}^{+}$+1) vs. *s*^+^ for the pure fluids. Fitting results for the four cubic EOS (PR, SRK, PTV and YFR) and the modified alpha functions (PR-Twu, PTV-Heyen, SRK-Coquelet, SRK-MS, PR-Coquelet and PR-MS) are provided based on the filtered literature data. Performance of the group-specific parameter fittings for each model is also provided in the subfolder group_plot.

**2.2. The relative deviation of viscosity experimental data to the RES and ECS models**

Location: Supporting Information/Figures/Deviation plots

Contains: Relative deviations of the analysable experimental data from the RES model and the models in REFPROP 10.0 for each of the pure fluids. Deviation results for the four cubic EOS (PR, SRK, PTV and YFR) and the modified alpha functions (PR-Twu, PTV-HEYEN, SRK-Coquelet, SRK-MS, PR-Coquelet and PR-MS) are provided where applicable. The legend format is structured as: publication year–first three letters of the first author's last name–first three letters of the second author's last name–phase number. If there is no second author, "Xxx" is used in place of the second author's name. The phase numbers are defined as follows:

1: single phase data with (*T*, *p*) reported.

2: saturated gas phase data in equilibrium with liquid.

3: saturated liquid phase data in equilibrium with gas.

4: single phase data with (*T*, ρ) reported.

**2.3. Summary for the viscosity deviations and calculation successful rate of all the models investigated in this work for each component**

Location: Supporting Information/Figures/Bar_chart_summary

Contains: This part includes bar charts summarizing the viscosity average absolute deviations (AAD), BIAS, and calculation success rates for all models evaluated in this work across all the components. The success rate is calculated as the ratio of analysable data to the total available data for each model and substance.

**2.4. Relative deviations of ten cubic model residual entropy calculations from reference EOS implemented in REFPROP 10.0 for each component**

Location: Supporting Information/Figures/Residual_entropy_deviation_models

Contains: This section presents illustrations of the relative deviations in residual entropy calculations using ten cubic EOS models when compared to the reference EOS implemented in REFPROP 10.0 for each component at 300 K.

**3. Reference list**

Location: Supporting Information/Reference list.docx

Contains: The references for the literature data analysed in this work.

**References**

(1) Lemmon, E. W.; Bell, I. H.; Huber, M. L.; McLinden, M. O. NIST Standard Reference Database 23: Reference Fluid Thermodynamic and Transport Properties-REFPROP, Version 10.0; National Institute of Standards and Technology. [*https://www.nist.gov/srd/ref*](https://www.nist.gov/srd/ref) **2018**.
