# Supplementary material for: Evaluating Cubic Equations of State with Various α Functions for Viscosity Predictions of 124 Industrial Important Fluids Based on Residual Entropy Scaling
Source: ACS Omega. 2025 Jun 27;10(27):29021–36. doi: 10.1021/acsomega.5c01157 (PMC12268422; doi:10.1021/acsomega.5c01157)
Supplement: Supplementary file 1 [file ao5c01157_si_001.zip › Supporting Information package 1/Reference list.docx]

(1)	Edwards, D. A.; Bonilla, C. F. Viscosity of Liquid Styrene and Butadiene. Ind. Eng. Chem. 1944, 36, 1038–1040. https://doi.org/10.1021/ie50419a016.

(2)	Neduzhii, I. A.; Khmara, Yu. I. Experimental Investigations of the Liquid - Viscosity of Propylene, Isobutylene, Butadiene-1,3, Toluol and Cyclohexane. Thermophys. Prop. Gases Liquids 1968, 1, 158–160.

(3)	Senftleben, H.; Gladisch. A Method of Determining the Specific Heats, the Viscosities and the Thermal Conductivities of Gases (in German). Z. Phys. 1949, 125, 653.

(4)	Dunlop, P. J. Viscosities of a Series of Gaseous Fluorocarbons at 25 .Degree.C. J. Chem. Phys. 1994, 100, 3149–3151. https://doi.org/10.1063/1.466405.

(5)	Liessmann, G.; Schmidt, W.; Reiffarth, S. Recommended Thermophysical Data. Data compilation of the Saechsische Olefinwerke Boehlen Germany 1995, 1.

(6)	Vargaftik, N. B. Dictionary of Thermophysical Properties of Gases and Liquids. 1972, 178–321.

(7)	Scholz, A.; Kley, G. Collection of Transport Property Data. 1981.

(8)	Golubev, I. F. Viscosity of Gases and Gas Mixtures; 1959.

(9)	Titani, T. The Viscosity of Vapors of Organic Compounds. Part II. Bull. Chem. Soc. Jpn. 1930, 5, 98.

(10)	Smutny, E. J.; Bondi, A. A. Di-Tert-Butyl Ether: Strain Energy and Physical Properties. J. Phys. Chem. 1961, 65, 546–550.

(11)	Brazier, D. W.; Freeman, G. R. The Effect of Pressure on the Density, Dielectric Constant, and Viscosity of Several Hydrocarbons and Other Organic Liquids. Can. J. Chem. 1969, 47, 893–899. https://doi.org/10.1139/v69-147.

(12)	Lambert, J. D.; Cotton, K. J.; Pailthorpe, M. W.; Robinson, A. M.; Scrivins, J.; Vale, W. R. F.; Young, R. M. Transport Properties of Gaseous Hydrocarbons. Proc. R. Soc. London, Ser. A 1955, 231, 280–290. https://doi.org/10.1098/rspa.1955.0173.

(13)	Chavanne, G.; Van Risseghem, H. Bull. Soc. Chim. Belg. 1922, 31, 87.

(14)	Aucejo, A.; CruzBurguet, M.; Munoz, R.; Marques, J. L. Densities, Viscosities, and Refractive Indices of the Binary Liquid Systems n-Alkanes + Isomers of Hexane at 298.15 K. J. Chem. Eng. Data 1995, 40, 871–874. https://doi.org/10.1021/je00020a029.

(15)	Kuss, E.; Pollmann, P. The Pressure Dependence of the Viscosity and the Degree of Branching of Liquid Alkanes. Z. Phys. Chem. (Munich) 1969, 68, 205–227.

(16)	Diaz Pena, M.; Esteban, F.; Sanchez, M. A. Viscosity of Hydrocarbons. I. Hexane and Isomers. An. R. Soc. Esp. Fis. Quim. Ser. A 1967, 63, 103–108.

(17)	McCall, D. W.; Douglass, D. C.; Anderson, E. W. Self-Diffusion in Liquids: Paraffin Hydrocarbons. Phys. Fluids (1958-1988) 1959, 2, 87–91. https://doi.org/10.1063/1.1724398.

(18)	Touazi, A. A.; Khellili, E.; Didaoui, S.; Khimeche, K.; Benziane, M. Measurement and Correlation of Isobaric Densities and Viscosities for Endo-Dicyclopentadiene with Hydrocarbons (C6-C8) at Different Temperatures. J. Therm. Anal. Calorim 2018, 134, 1223–1242. https://doi.org/10.1007/s10973-018-7583-2.

(19)	Neumann, S. Unpublished Data from Thesis. Unpublished Data 1995.

(20)	Gorbachev, M. Y.; Bersuker, I. B.; Dimoglo, A. S. The Dependence of the Viscosity of Organic Nonassociated Liquids on the Structural and Conformational Characteristics of Two Molecules. Teor. Eksp. Khim. 1989, 25, 563.

(21)	Eicher, L. D.; Zwolinski, B. J. Molecular Structure and Shear Viscosity. Isomeric Hexanes. J. Phys. Chem. 1972, 76, 3295.

(22)	Ling, A. C.; Willard, J. E. Viscosities of Some Organic Glasses Used as Trapping Matrices. J. Phys. Chem. 1968, 72, 1918.

(23)	Gramajo de Doz, M. B.; Cases, A. M.; Bonatti, C. M.; Solimo, H. N. Influence of Temperature on the (Liquid + Liquid) Equilibria of (3-Methyl Pentane + Cyclopentane + Methanol) Ternary System at T = (293.15, 297.15, and 299.15) K. J. Chem. Thermodyn. 2009, 41, 1279–1283. https://doi.org/10.1016/j.jct.2009.05.016.

(24)	Tu, C.-H.; Ku, H.-C.; Wang, W.-F.; Chou, Y.-T. Volumetric and Viscometric Properties of Methanol, Ethanol, Propan-2-Ol, and 2-Methylpropan-2-Ol with a Synthetic C6+ Mixture from 298.15 K to 318.15 K. J. Chem. Eng. Data 2001, 46, 317–321. https://doi.org/10.1021/je0002888.

(25)	Bouzas, A.; Burguet, M. C.; Monton, J. B.; Munoz, R. Densities, Viscosities, and Refractive Indices of the Binary Systems Methyl Tert-Butyl Ether + 2-Methylpentane, + 3-Methylpentane, + 2,3-Dimethylpentane, and + 2,2,4-Trimethylpentane at 298.15 K. J. Chem. Eng. Data 2000, 45, 331–333. https://doi.org/10.1021/je9902793.

(26)	Wen, C.; Meng, X.; Wei, K.; Wu, J. Compressed Liquid Viscosity of 2-Methylpentane, 3-Methylpentane, and 2,3-Dimethylbutane at Temperatures from (273 to 343) K and Pressures up to 40 MPa. J. Chem. Eng. Data 2017, 62, 1146–1152. https://doi.org/10.1021/acs.jced.6b01024.

(27)	Mohammed, G. A.; Oudah, M. Kh. Density, Viscosity and Refractive Index of the Ternary System (Ethanol + N-Hexane + 3-Methyl Pentane) at 20, 30, 40 C and 101.325 Kpa. Iraqi J. Chem. Petrol. Eng. 2015, 16, 51–58.

(28)	Thorpe, T. E.; Rodger, J. W. Philos. Trans. R. Soc. London, Ser. A 1894, 185, 522.

(29)	Batschinski, A. J. Investigations of the Internal Friction of Fluids. Z. Phys. Chem. 1913, 84, 643–706.

(30)	Bagdasaryan, S. S. Theory of Nuclei of a New Phase and the Limits of the Liquid State. Zh. Fiz. Khim. 1964, 38, 1816–1820.

(31)	Dunstan, A. E. LXXXIII. The Viscosity of Liquid Mixtures. Part. J. Chem. Soc. 1904, 85, 817–827.

(32)	Dunstan, A. E.; Hilditch, T. P. Determination of Viscosity and Other Physical Properties. Part II. Influence of Association of Unsaturted Groups. Ber. Bunsen-Ges. Phys. Chem. 1912, 18, 185.

(33)	Faust, O. The Viscosity of Liquid Mixtures, Their Dependence on Temperature and the Relation between the Viscosity of Liquids with Their Vapor Pressures. Z. Phys. Chem., Stoechiom. Verwandschaftsl. 1912, 79, 97.

(34)	Weissenberger, G.; Piatti, L. Uber Die Molekulberbindungen Der Phenole II. Das Verhalten Der Kresole Gegen Aromatische Kohlenwasserstoffe. Monatsh. Chem. 1924, 45, 281–285.

(35)	Weissenberger, G.; Schuster, F. Molecualr Compounds of Phenols III Behavior of Binary Systems with Phenols and Phenol Ethers. Monatsh. Chem. 1924, 45, 413.

(36)	Lewis, J. R. The Viscosity of Liquids Containing Dissolved Gases. J. Am. Chem. Soc. 1925, 47, 626–640. https://doi.org/10.1021/ja01680a007.

(37)	Timmermans, J.; Martin, F. Study of the Physical Constants of Twenty Organic Compounds of the Physical Constants of Twenty Organic Compounds. J. Chim. Phys. Phys.-Chim. Biol. 1928, 25, 411–451.

(38)	Bingham, E. C.; Brown, D. F. The Mixture Law. J. Rheol. 1932, 3, 95–112.

(39)	Hughes, O. L.; Hartley, H. Effect of Small Water Additions upon the Conductivity of Electrolytes in Non-Aqueous Solvents. Philos. Mag. 1933, 15, 610.

(40)	Reynolds, M. B.; Kraus, C. A. J. Am. Chem. Soc. 1948, 70, 1709–1713. https://doi.org/10.1021/ja01185a012.

(41)	Karr, A. E.; Bowes, W. M.; Scheibel, E. G. Analysis of Binary and Ternary Mixtures System Acetone + Chloroform + Methyl Isobutyl Ketone. Anal. Chem. 1951, 23, 459–463.

(42)	Jullander, I. Solubility Properties of Nonionic Water-Soluble Cellulose Ethers in Mixtures of Water and Alcohol. Acta Chem. Scand. 1955, 9, 1620.

(43)	Ling, T. D.; Van Winkle, M. Interfacial Tension at the Boiling Point and Vapor Viscosity Near the Dew Point of Binary Mixtures. Ind. Eng. Chem. Chem. Eng. Data Ser. 1958, 3, 82–88.

(44)	Blank, M. G. Miscibility in Ternary Liquid Systems Containing Formamide and Acetone. Zh. Fiz. Khim. 1968, 42, 3056–3062.

(45)	Konobeev, B. I.; Lyapin, V. V. Density, Viscosity, and Surface Tension Data on Certain Binary Systems. Zh. Prikl. Khim. 1970, 43, 803–810.

(46)	Campbell, R. H.; Kartzmark, E. M. Thermodynamic and Other Properties of Methanol + Acetone, Carbon Disulphide + Acetone, Carbon Disulphide + Methanol, and Carbon Disulphide + Methanol + Acetone. J. Chem. Thermodyn. 1973, 5, 163–172. https://doi.org/10.1016/s0021-9614(73)80076-x.

(47)	Terent’eva, A. A.; Krumgal’z, B. S.; Gerzhberg, Yu. I. Density and Viscosity of Acetone and Methyl Ethyl Ketone at Temp. below 0. Zh. Prikl. Khim. 1973, 46, 1143.

(48)	Atoyan, V. A.; Mamedov, I. A. Complex Study of Acoustic and Viscous Properties of Dimethyl Ketone in a Wide Range of Temperatures and Pressures. Zh. Fiz. Khim. 1976, 50, 2976–2978.

(49)	Hafez, M.; Hartland, S. Densities and Viscosities of Binary Systems Toluene-Acetone and 4-Methyl-2-Pentanone-Acetic Acid at 20, 25, 35, and 45 Deg C. J. Chem. Eng. Data 1976, 21, 179–182. https://doi.org/10.1021/je60069a011.

(50)	Kerchove, F. V.; De Vijlder, M. Some Physicochemical Properties of the Binary Mixtures Heptane-Propanone and Heptane-Ethyl Acetate. J. Chem. Eng. Data 1977, 22, 333–337. https://doi.org/10.1021/je60074a005.

(51)	Yadava, R. R.; Yadava, S. S. Viscosmetric Study of Molecular Interactions in Binary Liquid Mixtures of Polar and Nonpolar Solvents. Indian J. Chem., Sect. A: Inorg., Phys., Theor. Anal. 1981, 20, 221–224.

(52)	Noda, K.; Ohashi, M.; Ishida, K. Viscosities and Densities at 298.15 K for Mixtures of Methanol, Acetone and Water. J. Chem. Eng. Data 1982, 27, 326–328. https://doi.org/10.1021/je00029a028.

(53)	Prakash, S.; Srivastava, S.; Singh, R. Molecular Interaction of Benzyl Alcohol with Dioxan and Acetone at 30deg C, 35deg C and 40deg C. Acustica 1990, 71, 1990.

(54)	Schumpe, A.; Luehring, P. Oxygen Diffusivities in Organic Liquids at 293.15 K. J. Chem. Eng. Data 1990, 35, 24–25. https://doi.org/10.1021/je00059a007.

(55)	Das, A. K.; Jha, B. L. Molecular Interactions in Binary Mixture of DMSO-Aromatic/Aliphatic Hydrocarbons. J. Mol. Liq. 1991, 50, 155–186.

(56)	Zhang, J.; Liu, H. Determination of Viscosity under Pressure for Organic Substances and Its Correlation with Density under High Pressure. Huagong Xuebao 1991, 42, 269–277.

(57)	Liu, H.; Luo, Z. Experimental Determination of the Density and Viscosity for Liquid Mixtures. Huaxue Gongcheng 1992, 20, 65–68.

(58)	Lorenzi, L. D.; Fermeglia, M.; Torriano, G. Densities and Viscosities of 1,1,1-Trichloroethane with 13 Different Solvents at 298.15 K. J. Chem. Eng. Data 1995, 40, 1172–1177. https://doi.org/10.1021/je00022a003.

(59)	Tsierkezos, N. G.; Molinou, I. E. Densities and Viscosities of Ethylene Glycol Binary Mixtures at 293.15 K. J. Chem. Eng. Data 1999, 44, 955–958. https://doi.org/10.1021/je990059p.

(60)	Kinart, C. M.; Kinart, W. J.; Cwiklinska, A. Density and Viscosity at Various Temperatures for 2-Methoxyethanol + Acetone Mixtures. J. Chem. Eng. Data 2002, 47, 76–78. https://doi.org/10.1021/je010213j.

(61)	Peng, I.-H.; Tu, C.-H. Densities and Viscosities of Acetone, Diisopropyl Ether, Ethanol, and Methyl Ethyl Ketone with a Five-Component Hydrocarbon Mixture from 288.15 K to 308.15 K. J. Chem. Eng. Data 2002, 47, 1457–1461. https://doi.org/10.1021/je020077y.

(62)	Govindarajan, S.; Kannappan, V.; Naresh, M. D.; Venkataboopathy, K.; Lokanadam, B. Ultrasonic Studies on the Molecular Interaction of Gallic Acid in Aqueous Methanol and Acetone Solutions and the Role of Gallic Acid as Viscosity Reducer. J. Mol. Liq. 2003, 107, 289–316.

(63)	Agarwal, D.; Singh, M. Densities and Viscosities of Binary Liquid Mixtures of Trichloroethylene and Tetrachloroethylene with Some Polar and Nonpolar Solvents. J. Chem. Eng. Data 2004, 49, 1218–1224. https://doi.org/10.1021/je034203p.

(64)	Jain, P.; Singh, M. Density, Viscosity, and Excess Properties of Binary Liquid Mixtures of Propylene Carbonate with Polar and Nonpolar Solvents. J. Chem. Eng. Data 2004, 49, 1214–1217. https://doi.org/10.1021/je034204h.

(65)	Chen, S.; Lei, Q.; Fang, W. Viscosities and Densities for Binary Mixtures of N-Methylpiperazine with Methanol, Ethanol, n-Propanol, Iso-Propanol, n-Butanol and Iso-Butanol at 293.15, 298.15 and 303.15K. Fluid Phase Equilib. 2005, 234, 22–33. https://doi.org/10.1016/j.fluid.2005.05.012.

(66)	Gonzalez, B.; Dominguez, A.; Tojo, J.; Esteves, M. J. C.; Cardoso, M. J. E. d. M.; Barcia, O. E. Dynamic Viscosities of KI or NH4I in Methanol and NH4I in Ethanol at Several Temperatures and 0.1 MPa. J. Chem. Eng. Data 2005, 50, 109–112. https://doi.org/10.1021/je049808f.

(67)	Wang, J.; Zhu, A.; Zhuo, K. Excess Molar Volumes and Excess Logarithm Viscosities for Binary Mixtures of the Ionic Liquid 1-Butyl-3-Methylimidazolium Hexaflurophosphate with Some Organic Compounds. J. Solution Chem. 2005, 34, 585–596.

(68)	Gonzalez, E. J.; Alonso, L.; Dominguez, A. Physical Properties of Binary Mixtures of the Ionic Liquid 1-Methyl-3-Octylimidazolium Chloride with Methanol, Ethanol, and 1-Propanol at T = (298.15, 313.15, and 328.15) K and at P ) 0.1 MPa. J. Chem. Eng. Data 2006, 51, 1446–1452. https://doi.org/10.1021/je060123k.

(69)	Ren, Y.; Xia, S.; Ma, P. Measurement of Densities and Viscosities of Crotonic Acid - Solvent Binary Systems. Shiyou Huagong 2007, 36, 267.

(70)	Sih, R.; Armenti, M.; Mammucari, R.; Dehghani, F.; Foster, N. R. Viscosity Measurements on Saturated Gas-Expanded Liquid Systems Ethanol and Carbon Dioxide. J. Supercrit. Fluids 2008, 43, 460–468.

(71)	Tian, Y.; Wang, X.; Wang, J. Densities and Viscosities of 1-Butyl-3-Methylimidazolium Tetrafluoroborate + Molecular Solvent Binary Mixtures. J. Chem. Eng. Data 2008, 53, 2056–2059. https://doi.org/10.1021/je8001128.

(72)	Rajagopal, K.; Chenthilnath, S. Molecular Interaction Studies and Theoretical Estimation of Ultrasonic Speeds Using Scaled Particle Theory in Binary Mixtures of Toluene with Homologous Nitriles at Different Temperatures. Thermochim. Acta 2010, 498, 45–53. https://doi.org/10.1016/j.tca.2009.10.001.

(73)	Rajagopal, K.; Chenthilnath, S. Density and Viscosity of Ketones with Toluene at Different Temperatures and at Atmospheric Pressure. J. Chem. Eng. Data 2010, 55, 1060–1063. https://doi.org/10.1021/je900523k.

(74)	Yang, L.; Luo, T.-L.; Lian, H.-L.; Liu, G.-J. Density and Viscosity of (2,2-Dichloro-N,N-Di-2-Propenylacetamide + Acetone) and (2,2-Dichloro-N,N-Di-2-Propenylacetamide + Ethanol) at T = (278.15 to 313.15) K. J. Chem. Eng. Data 2010, 55, 1364–1367. https://doi.org/10.1021/je900535d.

(75)	Titani, T. The Viscosity of Vapors of Organic Compounds. Part III. Bull. Chem. Soc. Jpn. 1933, 8, 255–276.

(76)	Craven, P. M.; Lambert, J. D. The Viscosities of Organic Vapors. Proc. R. Soc. (London) 1951, 205, 439–449.

(77)	Atoyan, V. A.; Mamedov, I. A. Study of Acoustic and Viscous Properties of Certain Ketones in the Liquid Phase at V = Const and Dependence on Molecular Weight. Zh. Fiz. Khim. 1980, 54, 856–860.

(78)	Kiyama, R.; Makita, T. The Viscosity of Carbon Dioxide, Ammonia, Acetylene, Argon and Oxygen under High Pressures. Rev. Phys. Chem. Jpn. 1952, 22, 49.

(79)	Wobser, R.; Mueller, F. The Viscosity of Gases and Vapors and Their Mixtures in the Hoeppler Viscosimeter (in German). Kolloid-Beih. 1941, 52, 165–276.

(80)	Vogel, H. Uber Die Viskosital Einiger Gase Und Ihre Temperaturabhangigkeit Bei Tiefen Temperaturen. Ann. Phys. (Leipzig) 1914, 43, 1235.

(81)	Bailey, B. J. The Viscosity of Carbon Dioxide and Acetylene at Atmospheric Pressure. J. Phys. D 1970, 3, 550–562. https://doi.org/10.1088/0022-3727/3/4/312.

(82)	Pal, A. K.; Barua, A. K. Viscosity of Some Quadrupolar Gases and Vapors. J. Chem. Phys. 1968, 48, 872–874. https://doi.org/10.1063/1.1668726.

(83)	Makita, T. Mem. Fac. Ind. Arts, Kyoto Techn. Univ., Sci. Technol. 1955, 4, 19.

(84)	Makhija, R. C.; Stairs, R. A. Viscosity of Associated Liquids. Can. J. Chem. 1970, 48, 1214.

(85)	Laesecke, A.; Perking, R. A.; Nieto de Castro, C. A. Thermal Conductivity of R134a. Fluid Phase Equilib. 1992, 80, 263–274. https://doi.org/10.1016/0378-3812(92)87073-v.

(86)	Trautz, M.; Heberling, R. Viscosity of Ammonia & Its Mixture with Hydrogen, Nitrogen, Ethylene. Ann. Phys. (Berlin) 1931, 10, 155.

(87)	Van Cleave, A. B.; Maass, O. The Molecular Diameter of Deuterium as Determined by Viscosity Measurements. Can. J. Res. 1935, 12, 57–62.

(88)	Burch, L. G.; Raw, C. J. G. Transport Properties of Polar-Gas Mixtures. I. Viscosities of Ammonia- Methylamine Mixtures. J. Chem. Phys. 1967, 47, 2798–2801.

(89)	Carmichael, L. T.; Reamer, H. H.; Sage, B. H. Viscosity of Ammonia at High Pressures. J. Chem. Eng. Data 1963, 8, 400–404. https://doi.org/10.1021/je60018a032.

(90)	Sun, S. B. K.; Storvick, T. S. Viscosity of Ammonia at High Temperature and Pressure. J. Chem. Eng. Data 1979, 24, 88–91. https://doi.org/10.1021/je60081a009.

(91)	Hellemans, J.; Zink, H.; Van Paemel, O. The Viscosity of Liquid Nitrogen and Liguid Oxigen along Isoterms as a Function of Pressure. Physica (Amsterdam) 1970, 47, 45.

(92)	vanderGulik, P. S.; Trappeniers, N. J. The Viscosity of Argon at High Densities. Physica A (Amsterdam) 1986, 135, 1–20.

(93)	Andreev, I. I.; Tsderberg, N. V.; Popov, V. N. Experimental Study of the Viscosity of Argon. Teploenergetika 1966, 78–81.

(94)	Timrot, D. L.; Serednitskaya, M. A.; Traktueva, S. A. Viscosity of Gases as Studied by the Oscillating Disk Method. Teploenergetika 1969, 16, 83–85.

(95)	Kalelkar, A. S.; Kestin, J. Viscosity of He-Ar and He-Kr Binary Gaseous Mixtures in the Temperature Range 25-720 C. J. Chem. Phys. 1970, 52, 4248.

(96)	Kestin, J.; Wakeham, W.; Watanabe, K. Viscosity, Thermal Conductivity, and Diffusion Coefficient of Ar-Ne and Ar- Kr Gaseous Mixtures in the Temperature Range 25-700 C. J. Chem. Phys. 1970, 53, 3773–3780.

(97)	Kestin, J.; Ro, S. T.; Wakeham, W. An Extended Law of Corresponding States for the Equilibrium and Transport Properties of the Noble Gases. Phys. Norv. 1972, 58, 165–211.

(98)	Borisov, S. F.; Kalinin, B. A.; Porodnov, B. T.; Suetin, P. E. Measurements of the Viscosity Coefficients of Gases by the Method of Non Steady-State Flow. Inzh.-Fiz. Zh. 1973, 24, 67–69.

(99)	Kurin, V. I.; Golubev, I. F. Viscosity of Argon, Air, and Carbon Dioxide at Pressures to 400 at and Different Temperatures. Teploenergetika 1974, 21, 125.

(100)	Timrot, D. L.; Serednitskaya, M. A.; Traktueva, S. A. Investigation of Viscosity of Air at Temperatures 300-700 K and Pressures 0 .1 - 12 MPa by Oscillating Disk Method. Teploenergetika 1975, 22, 84–87.

(101)	Lyusternik, V. E.; Lavushev, A. V. Study of the Viscosity of Argon up to 2000 K with the Method of Flow through the Porous Media. Teplofiz. Vys. Temp. 1976, 14, 970–977.

(102)	Kestin, J.; Wakeham, W. A. The Viscosity of Three Polar Gases. Ber. Bunsen-Ges. Phys. Chem. 1979, 83, 573.

(103)	Hobley, A.; Matthews, G. P.; Townsend, A. The Use of a Novel Capillary Flow Viscometer for the Study of the Argon/ Carbon Dioxide System. Int. J. Thermophys. 1989, 10, 1165–1179. https://doi.org/10.1007/bf00500569.

(104)	Evers, C.; Loesch, H. W.; Wagner, W. An Absolute Viscometer-Densimeter and Measurements of the Viscosity of Nitrogen, Methane, Helium, Neon, Argon, and Krypton over a Wide Range of Density and Temperature. Int. J. Thermophys. 2002, 23, 1411–1439. https://doi.org/10.1023/a:1020784330515.

(105)	Hurly, J. J.; Gillis, K. A.; Mehl, J. B.; Moldover, M. R. The Viscosity of Seven Gases Measured with a Greenspan Viscometer. Int. J. Thermophys. 2003, 24, 1441–1474. https://doi.org/10.1023/b:ijot.0000004088.04964.4c.

(106)	Wang, X.; Wu, J.; Liu, Z. Viscosity of Gaseous HFC245fa. J. Chem. Eng. Data 2010, 55, 496–499. https://doi.org/10.1021/je900279z.

(107)	Wilhelm, J.; Vogel, E. Viscosity Measurements of Gaseous Argon, Krypton, and Propane. Int. J. Thermophys. 2000, 21, 301–318. https://doi.org/10.1023/a:1006667125801.

(108)	Vogel, E. Reference Viscosity of Argon at Low Density in the Temperature Range from 290 K to 680 K. Int. J. Thermophys. 2010, 31, 447–461. https://doi.org/10.1007/s10765-010-0760-6.

(109)	Carey, C.; Bradshaw, J.; Lin, E.; Carnevale, E. H. Experimental Determination of Gas Properties at High Temperatures and/or Pressures; AEDC-TR-74-33; Panametrics Inc. Experimetnal Determination of Gas Properties at High Temperatures and/or Pressures: Waltham, MA, 1974.

(110)	Goodwin, A. R. H.; Fitt, A. D.; Ronaldson, K. A.; Wakeham, W. A. A Vibrating Plate Fabricated by the Methods of Microelectromechanical Systems (MEMS) for the Simultaneous Measurement of Density and Viscosity: Results for Argon at Temperatures Between 323 and 423K at Pressures up to 68MPa. Int. J. Thermophys. 2006, 27, 1650–1676. https://doi.org/10.1007/s10765-006-0114-6.

(111)	Heydweiller, A. The Viscosity of Liquids above Their Boiling Points. Wied. Ann. 1896, 59, 193.

(112)	Khalilov, Kh. M. Viscosity of Liquids and Saturated Vapors at High Temperatures & Pressures. Zh. Eksp. Teor. Fiz. 1939, 9, 335–345.

(113)	Hammond, B. R.; Stokes, R. H. Diffusion in Binary Liquid Mixtures. Part 2 The Diffusion of Carbon Tetrachloride in Some Organic Solvents at 25 Deg. Trans. Faraday Soc. 1955, 51, 1641.

(114)	Kuss, E. High Pressure Measurements III. The Viscosity and Compressibility of Fluids. Z. Angew. Phys. 1955, 7, 372–378.

(115)	Heiks, J. R.; Orban, E. Liquid Viscosities at Elevated Tempaeratures and Pressures: Viscosity of Benzene from 90 Deg to Its Critical Temperature. J. Phys. Chem. 1956, 60, 1025.

(116)	Teller, A. J.; Walsh, T. J. Viscosities of Benzene-Methyl Ethyl Ketone Systems. J. Chem. Eng. Data 1959, 4, 279–283. https://doi.org/10.1021/je60003a023.

(117)	Bulanov, N. V.; Nikitin, E. D.; Skripov, V. P. Thermal Conductivbity of Liquids in the Metastable State. Inzh.-Fiz. Zh. 1974, 26, 204–207.

(118)	Gartenmeister, R. The Viscosity of Liquid Carbon Compounds and Its Relationship to Chemical Consitution. Z. Phys. Chem., Stoechiom. Verwandschaftsl. 1890, 6, 524.

(119)	Dunstan, A. E.; Thole, B. T.; Hunt, J. S. The Relation between Viscosity and Vhemical Constitution. Part I. The Viscosity of Pyridine Solutions. J. Chem. Soc. 1907, 91, 1728–1736.

(120)	Dunstan, A. E.; Stubbs, J. A. The Relation between Viscosity and Chemical Constitution. Part III. the Enol-Ketonic Tautomerism. J. Chem. Soc. Trans. 1908, 93, 1919.

(121)	Finney, R. J.; Fury, M.; Jonas, J. Density and Temperature Dependence of Self-Diffusion and Shear Viscosity of Perfluorocyclobutane in the Dense Fluid Region. J. Chem. Phys. 1977, 66, 760–765.

(122)	Dunstan, A. E.; Hilditch, T. P.; Thole, F. B. The Relation between Viscosity and Chemical Constitution: Vii the Effect of the Relative Position of Two Unsaturated Groups on Viscosity. J. Chem. Soc. Trans. 1913, 103, 133.

(123)	Muchin, G. Measurement of Fluidity on Solutions. Z. Elektrochem. Angew. Phys. Chem. 1913, 19, 819.

(124)	Bingham, E. C.; Sarver, L. A. Fluidities and Specific Volumes of Mixtures of Benzyl Benzoate and Benzene XXIX. J. Am. Chem. Soc. 1920, 42, 2011–2022. https://doi.org/10.1021/ja01455a007.

(125)	Meyer, J.; Mylius, B. Knowledge of Viscosity of Binary Liquid Mixtures. Z. Phys. Chem., Stoechiom. Verwandschaftsl. 1920, 95, 349.

(126)	Schaaf, F. Higher Alcohols from Petroleum Olefins. Z. Phys. Chem. (Leipzig) 1923, 126, 237.

(127)	Miller, C. C. The Stokes-Einstein Law for Diffusion in Solution. Proc. R. Soc. London, Ser. A 1924, 106, 724.

(128)	Kolossowsky, N. D. Bull. Soc. Chim. Belg. 1925, 34, 221.

(129)	Martin, F. Bull. Soc. Chim. Belg. 1925, 34, 82.

(130)	Dessart, A. Theory of Concentrated Solutions: III. Physical Constants of Mixtures of m-Nitrotoluene and m-Toluidine with Some Hydrocarbons. Bull. Soc. Chim. Belg. 1926, 35, 9.

(131)	Timmermans, J.; Martin, F. ? J. Chim. Phys. Phys.-Chim. Biol. 1926, 23, 747.

(132)	Madge, E. W. J. Phys. Chem. 1930, 34, 1599.

(133)	Tausz, J.; Staab, A. Lubricating Oils. Petroleum Z. 1930, 26, 1129–1140.

(134)	Kireev, V. A.; Skvortsova, A. A. Equilibrium in Liquid Mixtures and Solutions: Vii Elasticity, Vapor- Tension, Compositon and Some Other Properties of Binary Mixtures Benzene + Dichloroethane. Zh. Fiz. Khim. 1936, 7, 63–70.

(135)	Shalberov, N. A.; Ostroumov, V. V. Use of Gas Viscometers Opf the Rankin Type for Measuring Viscosity of Liquids. Zh. Fiz. Khim. 1936, 7, 552.

(136)	Lemonde, H. Diffusion and Viscosity in Binary Liquid Mixtures. Ann. Phys. (Berlin) 1938, 9, 539–644.

(137)	Salceanu, C. New Method for the Determination of the Viscosity of Liquids. C. R. Hebd. Seances Acad. Sci. 1939, 208, 1797–1799.

(138)	Udovenko, V. V.; Toropov, A. P. Zh. Obshch. Khim. 1940, 10, 11–16.

(139)	Linke, R. The Viscosity of Binary Liquid Mixtures. Z. Phys. Chem. Abt. A 1941, 188, 17.

(140)	Teitel’baum, B. Ya.; Gortalova, T. A.; Ganelina, S. G. Viscosity of Systems Formed by Benzenes and Its Homologs with the Lower Alcohols. Zh. Obshch. Khim. 1950, 20, 1422.

(141)	Subnis, M. V. Rheochor Part II. (Application of Hammick and Andrews Equation to Binary Mixtures). Univ. Rajputana Studies, Phys. Sci. 1952, 121–127.

(142)	Dixon, J. A.; Schiessler, R. W. J. Phys. Chem. 1954, 58, 430.

(143)	Katti, P. K.; Chaudhri, M. M.; Prakash, O. Viscosities of Binary Mixtures Involving Benzene, Carbon Tetrachloride and Cyclohexane. J. Chem. Eng. Data 1966, 11, 593–594. https://doi.org/10.1021/je60031a044.

(144)	Ridgway, K.; Butler, P. A. Some Physical Properties of the Ternary System Benzene-Cyclohexane-n-Hexane. J. Chem. Eng. Data 1967, 12, 509–515. https://doi.org/10.1021/je60035a012.

(145)	Collings, A. F.; McLaughlin, E. Torsional Crystal Technique for the Measurement of Viscosities of Liquids at High Pressure. Trans. Faraday Soc. 1971, 67, 340.

(146)	Mamedov, A. M.; Akhundov, T. S.; Ismail-Zade, Sh. M.; Tairov, A. D. Viscosity of Benzene. Izv. Vyssh. Uchebn. Zaved., Neft Gaz 1971, 14, 74–76.

(147)	Alms, G. R.; Bauer, D. R.; Brauman, J. I.; Pecora, R. Depolarized Rayleigh Scattering and Orientational Relaxation of Molecules in Solution. I. Benzene, Toluene, and p-Xylene. J. Chem. Phys. 1973, 58, 5570–5578.

(148)	Parkhurst, H. J.; Jonas, J. Dense Liquids. II. The Effect of Density and Temperature on Viscosity of Tetramethylsilane and Benzene. J. Chem. Phys. 1975, 63, 2705–2709.

(149)	Dhillon, M. S.; Chugh, H. S. Viscosities and Activation Energies of Flow of Mixtures Containing 1,2- Dibromoethane. Thermochim. Acta 1976, 16, 345–353. https://doi.org/10.1016/0040-6031(76)80027-5.

(150)	Asfour, A. A.; Dullien, F. A. L. Viscosities and Densities of Four Binary Liquid Systems at 25.00 Deg C. J. Chem. Eng. Data 1981, 26, 312–316. https://doi.org/10.1021/je00025a028.

(151)	Dymond, J. H.; Robertson, J.; Isdale, J. D. Transport Properties of Nonelectrolyte Liquid Mixtures. III. Viscosity Coefficients for n-Octane, n-Dodecane, and Equimolar Mixtures of n-Octane + n-Dodecane from 25 to 100.Degree. C at Pressures Up. Int. J. Thermophys. 1981, 2, 133–154. https://doi.org/10.1007/bf00503937.

(152)	Kashiwagi, H.; Makita, T. Viscosity of Twelve Hydrocarbon Liquids in the Temperature Range 298-348 K at Pressures Up To 110 MPa. Int. J. Thermophys. 1982, 3, 289–305. https://doi.org/10.1007/bf00502346.

(153)	Abdullaev, F. G.; Akhundov, R. T. Experimental Study of the Viscosity of Ethylbenzene and O-, m-, and p-Xylene in Gaseous Phase. Izv. Vyssh. Uchebn. Zaved., Neft Gaz 1983, 28.

(154)	Albright, J. G.; Edge, A. V. J.; Mills, R. Relations Between Intradiffusion and Viscosity Coefficients Albright Equation. J. Chem. Soc., Faraday Trans. 1 1983, 79, 1327.

(155)	Oswal, S.; Rathnam, M. V. Viscosity Data of Binary Mixtures: Ethyl Acetate + Cyclohexane, + Benzene, + Toluene, + Ethylbenzene + Carbon Tetrachloride, and + Chloroform at 303.1 5 K. Can. J. Chem. 1984, 62, 2851–2853. https://doi.org/10.1139/v84-482.

(156)	Awwad, A. M.; Al-Azzawi, S. F.; Salman, M. A. Volumes and Viscosities of Benzene + N-Alkane Mixtures. Fluid Phase Equilib. 1986, 31, 171–182. https://doi.org/10.1016/0378-3812(86)90011-7.

(157)	Oswal, S. L.; Rao, A. V. Studies on Viscosity & Volume of Mixing of Binary Liquid Mixtures of Tertiary Amines with Cyclohexane & Benzene. Indian J. Chem., Sect. A: Inorg., Phys., Theor. Anal. 1985, 24, 1026.

(158)	Fischer, J.; Weiss, A. Transport Properties of Liquids. V. Self-Diffusion, Viscosity, and Mass Density of Ellipsoidal Shaped Molecules in the Pure Liquid Phase. Ber. Bunsen-Ges. Phys. Chem. 1986, 90, 896.

(159)	Manjeshwar, L. S.; Aminabhavi, T. M. Densities and Viscosities of Binary Liquid Mixtures at 45 Deg. C. J. Chem. Eng. Data 1987, 32, 409–412. https://doi.org/10.1021/je00050a006.

(160)	Ramanjaneyulu, K.; Chandrasekhar, A. C. H.; Venkateswarlu, P.; Krishnaiah, A. A Study of Interactions in Binary Mixtures from Viscosity Measurements. Phys. Chem. Liq. 1987, 16, 217–223.

(161)	Chandrasekhar, A. C. H.; Surendra Nath, K. N.; Krishnaiah, A. Volumetric and Transport Properties of Volumetric and Transport Properties of 1,1,1-Trichloroethane with Some Aliphatic, Alicyclic and Aromatic Hydrocarbons at 303.15 K. Chem. Scr. 1988, 28, 421–425.

(162)	Rao, K. P. C.; Reddy, K. S. Viscosities of Binary Liquid Mixtures of Cyclohexanone with Alkanes, Benzene, Toluene, and Tetrachloromethane at 298.15 K. Can. J. Chem. Eng. 1988, 66, 474.

(163)	Rathnam, M. V. Viscosities and Densities of SOme Binary Liquid Mixtures of Esters at 303.15 and 313.15 K. J. Chem. Eng. Data 1988, 33, 509–511. https://doi.org/10.1021/je00054a034.

(164)	Knapstad, B.; Skjolsvik, P. A.; Oye, H. A. Viscosity of Pure Hydrocarbons. J. Chem. Eng. Data 1989, 34, 37–43. https://doi.org/10.1021/je00055a013.

(165)	Kouris, S.; Panayioton, C. Dynamic Viscosity of Mixtures of Benzene, Ethanol, and n-Heptane. J. Chem. Eng. Data 1989, 34, 200–203. https://doi.org/10.1021/je00056a016.

(166)	Ruiz, E.; Urieta, J. S.; Santafe, J.; Gutierrez Losa, C. Viscosities of Benzene or Cyclohexane + 2,4-Lutidine, + 2,6-Lutidine, + Collidine, + Mesitylene, + m-Xylene, and + p-Xylene at 303.15, 313.15 and 323.15 K. Bull. Soc. Chim. Fr. 1989, 304.

(167)	Schrodt, J. T.; Akel, R. M. Binary Liquid Viscosities and Their Estimation from Classical Solution Thermodynamics. J. Chem. Eng. Data 1989, 34, 8–13. https://doi.org/10.1021/je00055a003.

(168)	Joshi, S. S.; Aminabhavi, T. M.; Balundgi, R. H.; Shukla, S. S. Densities and Viscosities of Binary Liquid Mixtures of Nitrobenzene with Cyclohexane and N,N-Dimethylformamide. J. Chem. Eng. Data 1990, 35, 185–187. https://doi.org/10.1021/je00060a027.

(169)	Sekar, P. R.; Venkateswarlu, R.; Reddy, K. S. Excess Volumes, Isentropic Compressibilities, and Viscosities of Binary Mixtures Containing Cyclohexene. Can. J. Chem. 1990, 68, 363–368. https://doi.org/10.1139/v90-054.

(170)	Sivaramprasad, G.; Rao, M. V.; Prasad, D. H. L. Density and Viscosity of Ethanol + 1,2-Dichloroethane, Ethanol + 1,1,1- Trichloroethane, and Ethanol + 1,1,2,2-Tetrachloroethane Binary Mixtures. J. Chem. Eng. Data 1990, 35, 122–124. https://doi.org/10.1021/je00060a006.

(171)	Aminabhavi, T. M.; Aminabhavi, V. A.; Joshi, S. S.; Balundgi, R. H. Excess Properties of Some Binary Liquid Mixtures in the Temperature Range 298.15-313.15 K. Indian J. Technol. 1991, 29, 545–557.

(172)	Assael, M. J.; Papadaki, M.; Wakeham, W. A. Measurements of the Viscosity of Benzene, Toluene, and m-Xylene at Pressure up to 80 MPa. Int. J. Thermophys. 1991, 12, 449–457. https://doi.org/10.1007/bf00502361.

(173)	Papaioannou, D.; Evangelou, T.; Panayioton, C. Dynamic Viscosity of Multicomponent Liquid Mixtures. J. Chem. Eng. Data 1991, 36, 43–46. https://doi.org/10.1021/je00001a013.

(174)	Ramakanth, C.; Mukherjee, A. K.; Das, T. R. Diffusion Coeffcients for Selected Binary Liquid Systems: Cyclohexanes and n-Alkyl Alcohols. J. Chem. Eng. Data 1991, 36, 384–387. https://doi.org/10.1021/je00004a012.

(175)	Aralaguppi, M. I.; Aminabhavi, T. M.; Harogoppad, S. B.; Balundgi, R. H. Thermodynamic Interactions in Binary Mixtures of Dimethyl Sulfoxide with Benzene, Toluene, 1,3-Dimethylbenzene, 1,3,5-Trimethylbenzene, and Methoxybenzene from 298.15 to 308.15 K. J. Chem. Eng. Data 1992, 37, 298–303. https://doi.org/10.1021/je00007a006.

(176)	Klofutar, C.; Paljk, S.; Golc-Teger, S. Thermodynamic Functions of Activation for Viscous Flow of Cholesterol in Some Nonaqueous Solutions. Thermochim. Acta 1992, 206, 19–32. https://doi.org/10.1016/0040-6031(92)85280-9.

(177)	Oswal, S. L.; Patel, A. T. Viscosity of Binary Mixtures. II. n-Butyl, n-Hexyl, n-Octyl, n-Decyl, and n-Dodecylamine with Benzene and n-Hexyl, n-Decyl, and n-Dodecylamine with Cyclohexane. Int. J. Thermophys. 1992, 13, 629–641. https://doi.org/10.1007/bf00501945.

(178)	Tasioula-Margari, M.; Demetropoulos, I. N. Viscosities of Dilute Solutions of the Triglycerides Triolein, Trilinolein, Tristearin, Tripalmitin, and Trimyristin in Benzene and p-Xylene. J. Chem. Eng. Data 1992, 37, 77–79. https://doi.org/10.1021/je00005a023.

(179)	Shastri, S. S.; Mukherjee, A. K.; Das, T. R. Surface Tension of Some Binary Organic Solutions of Alkylbenzenes and 1-Alkanols. J. Chem. Eng. Data 1993, 38, 399–400. https://doi.org/10.1021/je00011a017.

(180)	Lafuente, C.; Lopez, M. C.; Santafe, J.; Royo, F. M.; Urieta, J. S. Excess Volumes and Excess Viscosities of Benzene with Picolines. Thermochim. Acta 1994, 237, 35–41. https://doi.org/10.1016/0040-6031(94)85181-6.

(181)	Exarchos, N. C.; Tasioula-Margari, M.; Demetropoulos, I. N. Viscosities and Densities of Dilute Solutions of Glycerol Trioleate + Octane, + p-Xylene, + Toluene, and + Chloroform. J. Chem. Eng. Data 1995, 40, 567–571. https://doi.org/10.1021/je00019a005.

(182)	Krishnan, K. M.; Rambabu, K.; Venkateswarlu, P.; Raman, G. K. A Study on Mixing Properties of Binary Mixtures of 2-Methoxyethanol with Aromatic Hydrocarbons. J. Chem. Eng. Data 1995, 40, 132–135. https://doi.org/10.1021/je00017a030.

(183)	Rived, F.; Roses, M.; Bosch, E. Densities, Refractive Indices, Absolute Viscosities, and Static Dielectric Constants of 2-Methylpropan-2-Ol + Hexane, + Benzene, + Propan-2-Ol, + Methanol, + Ethanol, and + Water at 303.2 K. J. Chem. Eng. Data 1995, 40, 1111–1114. https://doi.org/10.1021/je00021a017.

(184)	Swain, N.; Panda, D.; Singh, S. K.; Chakravortty, V. Viscosity and Density of Tri-n-Butyl Phosphate + Benzene + o-Xylene from 30 0C to 450C. J. Chem. Eng. Data 1997, 42, 1235–1237. https://doi.org/10.1021/je970129x.

(185)	Viera dos Santos, F. J.; Nieto de Castro, C. A. Viscosity of Toluene and Benzene under High Pressure. Int. J. Thermophys. 1997, 18, 367–378. https://doi.org/10.1007/bf02575167.

(186)	Casad, H.; Segade, L.; Franjo, C.; Jimenez Cuesta, E.; Paz Andrade, M. I. Excess Properties for Propyl Propanoate + Hexane + Benzene at 298.15 K. J. Chem. Eng. Data 1998, 43, 756–762. https://doi.org/10.1021/je9800609.

(187)	Swain, N.; Panda, D.; Singh, S. K.; Chakrovortty, V. VISCOSITIES AND DENSITIES OF BINARY LIQUID MIXTURES OF TBP WITH BENZENE, TOLUENE AND ORTHO-XYLENE. J. Mol. Liq. 1998, 75, 211–218.

(188)	Aminabhavi, T. M.; Banerjee, K. Density, Viscosity, Refractive Index, and Speed of Sound in Binary Mixtures of 1-Chloronaphthalene with Benzene, Methylbenzene, 1,4-Dimethylbenzene, 1,3,5-Trimethylbenzene, and Methoxybenzene at (298.15, 303.15, and 308.15) K. J. Chem. Eng. Data 1999, 44, 547–552. https://doi.org/10.1021/je980310l.

(189)	Aralaguppi, M. I.; Jadar, C. V.; Aminabhavi, T. M. Density, Refractive Index, Viscosity, and Speed of Sound in Binary Mixtures of Cyclohexanone with Hexane, Heptane, Octane, Nonane, Decane, Dodecane, and 2,2,4-Trimethylpentane. J. Chem. Eng. Data 1999, 44, 435–440. https://doi.org/10.1021/je9802266.

(190)	Guzman, C.; Lafuente, C.; Santafe, J.; Royo, F. M.; Urieta, J. S. Thermodynamic and Transport Properties of Binary Mixtures Containing 1,3-Dioxolane. Int. J. Thermophys. 1999, 20, 1435–1448. https://doi.org/10.1023/a:1021489005178.

(191)	Sastry, N. V.; Jain, N. J.; George, A.; Bahadur, P. Viscosities, Speeds of Sound and Excess Isentropic Compressibilities of Binary Mixtures of Alkyl Alkanoate-Hydrocarbons at 308.15 K and 318.15 K. Fluid Phase Equilib. 1999, 163, 275–289. https://doi.org/10.1016/s0378-3812(99)00232-0.

(192)	Swain, N.; Panda, D.; Singh, S. K.; Chakravortty, V. Viscosity and Density of Tri-n-Butyl Phosphate + Benzene + Toluene from 30 Deg C to 45 Deg C. J. Chem. Eng. Data 1999, 44, 32–34. https://doi.org/10.1021/je980143p.

(193)	Mariano, A.; Postigo, M.; Artigas, H.; Pardo, J.; Royo, F. M. Densities and Viscosities of the Ternary Mixture (Benzene 1-Propanol Ethyl Acetate) at 298.15 K. Phys. Chem. Liq. 2000, 38, 567–581.

(194)	Swain, N.; Singh, S. K.; Panda, D.; Chakravortty, V. Viscosities and Densities of Ternary Liquid Mixtures of DEHPA, Benzene and Ortho-Xylene. J. Mol. Liq. 2000, 85, 321–330.

(195)	Tsierkezos, N. G.; Palaiologou, M. M.; Molinou, I. E. Densities and Viscosities of 1-Pentanol Binary Mixtures at 293.15 K. J. Chem. Eng. Data 2000, 45, 272–275. https://doi.org/10.1021/je9902138.

(196)	Casad, H.; Segade, L.; Cabeza, O.; Franjo, C.; Jimenez Cuesta, E. Densities, Viscosities, and Refractive Indexes for Propyl Propanoate + 1-Hexanol + Benzene at 298.15 K. J. Chem. Eng. Data 2001, 46, 651–656. https://doi.org/10.1021/je0003593.

(197)	Chowdhury, M. A.; Majid, M. A.; Saleh, M. A. Volumetric and Viscometric Behaviour of Binary Systems: (1-Hexanol + Hydrocarbons). J. Chem. Thermodyn. 2001, 33, 347–360. https://doi.org/10.1006/jcht.2000.0751.

(198)	Kapadi, U. R.; Hundiwale, D. G.; Patil, N. B.; Lande, M. K.; Patil, P. R. Studies of Viscosity and Excess Molar Volume of Binary Mixtures of Propane-1,2 Diol with Water at Various Temperatures. Fluid Phase Equilib. 2001, 192, 63–70. https://doi.org/10.1016/s0378-3812(01)00621-5.

(199)	Pandey, J. D.; Dubey, N.; Dwivedi, D. C.; Dey, R. PREDICTION OF HEAT OF MIXING FROM INTERNAL PRESSURE DATA. Phys. Chem. Liq. 2001, 39, 781–790.

(200)	Swain, N.; Singh, S. K.; Panda, D.; Chakravortty, V. Viscosities, Densities and Excess Properties of Binary Mixtures of Di-(2-Ethyl Hexyl) Phosphoric Acid (DEHPA) with Benzene, Toluene and Ortho-Xylene. J. Mol. Liq. 2001, 94, 233–248.

(201)	Nayak, J. N.; Aralaguppi, M. I.; Aminabhavi, T. M. Density, Viscosity, Refractive Index, and Speed of Sound in the Binary Mixtures of Ethyl Chloroacetate with Aromatic Liquids at 298.15, 303.15, and 308.15 K. J. Chem. Eng. Data 2002, 47, 964–969. https://doi.org/10.1021/je0200158.

(202)	George, J.; Sastry, N. V. Thermophysical Properties of Binary Mixtures of Methyl Methacrylate+Di-Ethers (Ethyl, Isopropyl, and Butyl) at 298.15 and 308.15 K. Int. J. Thermophys. 2003, 24, 1697–1719. https://doi.org/10.1023/b:ijot.0000004100.28834.0a.

(203)	Lark, B. S.; Mehra, M.; Oswal, S. L.; Ghael, N. Y. Viscosity of Some Binary Mixtures of Arenes. Int. J. Thermophys. 2003, 24, 1475–1494. https://doi.org/10.1023/b:ijot.0000004089.27285.6c.

(204)	Nikam, P. S.; Kharat, S. J. Densities, Viscosities, and Thermodynamic Properties of (N,N-Dimethylformamide + Benzene + Chlorobenzene) Ternary Mixtures at (298.15, 303.15, 308.15, and 313.15) K. J. Chem. Eng. Data 2003, 48, 1202–1207. https://doi.org/10.1021/je030130y.

(205)	Oswal, S. L.; Maisuria, M. M.; Gardas, R. L. Viscosity of Binary Mixtures of Cycloalkane with Cycloalkane, Alkane and Aromatic Hydrocarbon at 303.15 K. J. Mol. Liq. 2003, 108, 199–215.

(206)	Ali, A.; Nain, A. K.; Lal, B.; Chand, D. Densities, Viscosities, and Refractive Indices of Binary Mixtures of Benzene with Isomeric Butanols at 30C. Int. J. Thermophys. 2004, 25, 1835–1847. https://doi.org/10.1007/s10765-004-7738-1.

(207)	Ma, P. S.; Zhou, Q.; Yang, C.; Xia, S. MEASUREMENT OF VISCOSITIES AND DENSITIES OF L IQUIDS BY VIBRATIN G-WIRE METHOD. Huagong Xuebao 2004, 55, 1608.

(208)	Oswal, S. L.; Desai, J. S.; Ijardar, S. P. Studies of Viscosities of Dilute Solutions of Alkylamine in Non-Electrolyte Solvents I. Aliphatic and Aromatic Hydrocarbons. Thermochim. Acta 2004, 423, 29–41. https://doi.org/10.1016/j.tca.2004.04.015.

(209)	Yang, C.; Xu, W.; Ma, P. Thermodynamic Properties of Binary Mixtures of P-Xylene with Cyclohexane, Heptane, Octane, and N-Methyl-2-Pyrrolidone at Several Temperatures. J. Chem. Eng. Data 2004, 49, 1794–1801. https://doi.org/10.1021/je049776w.

(210)	Oswal, S. L.; Gardas, R. L.; Phalak, R. P. Densities, Speeds of Sound, Isentropic Compressibilities, Refractive Indices and Viscosities of Binary Mixtures of Tetrahydrofuran with Hydrocarbons at 303.15 K. J. Mol. Liq. 2005, 116, 109–118.

(211)	Ali, A.; Nain, A. K.; Chand, D.; Ahmad, R. Volumetric, Ultrasonic, Viscometric and Refractive Index Behavior of Binary Mixtures of 2,2,4-Trimethylpentane with Aromatic Hydrocarbons: An Experimental and Theoretical Study. J. Mol. Liq. 2006, 128, 32–41.

(212)	Ali, A.; Tariq, M. Thermodynamic and Transport Behaviour of Binary Liquid Mixtures of Benzyl Alcohol with Monocyclic Aromatics at 303.15 K. J. Mol. Liq. 2006, 128, 50–55.

(213)	Baragi, J. G.; Aralaguppi, M. I.; Kariduraganavar, M. Y.; Kulkarni, S. S.; Kittur, A.; Aminabhavi, T. M. Excess Properties of the Binary Mixtures of Methylcyclohexane + Alkanes (C6 to C12) at T = 298.15 K to T = 308.15 K. J. Chem. Thermodyn. 2006, 38, 75–83. https://doi.org/10.1016/j.jct.2005.03.024.

(214)	Han, K. J.; Oh, J.-H.; Park, S.-J. Densities and Viscosities for the Ternary Systems of Methyl Tert-Butyl Ether + Methanol + Benzene and Methyl Tert-Butyl Ether + Methanol + Toluene and Their Sub-Binary Systems at 298.15 K. J. Chem. Eng. Data 2006, 51, 1339–1344. https://doi.org/10.1021/je0600799.

(215)	Suresh Reddy, K. V. N.; Sankara Reddy, G.; Krishnaiah, A. Excess Volumes, Densities, Speeds of Sound and Viscosities for the Binary Systems of Diisopropyl Ether with Hydrocarbons at 303.15K. Thermochim. Acta 2006, 440, 43–50. https://doi.org/10.1016/j.tca.2005.10.008.

(216)	Varshney, S.; Singh, M. Densities, Viscosities, and Excess Molar Volumes of Ternary Liquid Mixtures of Bromobenzene + 1,4-Dioxane + (Benzene or + Toluene or + Carbon Tetrachloride) and Some Associated Binary Liquid Mixtures. J. Chem. Eng. Data 2006, 51, 1136–1140. https://doi.org/10.1021/je0600303.

(217)	Hernandez-Galvan, M. A.; Garcia-Sanchez, F.; Macias-Salinas, R. Liquid Viscosities of Benzene, n-Tetradecane, and Benzene + n-Tetradecane from 313 to 393K and Pressures up to 60MPa: Experiment and Modeling. Fluid Phase Equilib. 2007, 262, 51–60. https://doi.org/10.1016/j.fluid.2007.08.010.

(218)	Kharat, S. J.; Nikam, P. S. Density and Viscosity Studies of Binary Mixtures of Aniline+benzene and Ternary Mixtures of (Aniline+benzene+N,N-Dimethylformamide) at 298.15, 303.15, 308.15, and 313.15 K. J. Mol. Liq. 2007, 131–132, 81–86.

(219)	Nain, A. K. Ultrasonic and Viscometric Studies of Molecular Interactions in Binary Mixtures of Tetrahydrofuran with Some Aromatic Hydrocarbons at Temperatures from 288.15 to 318.15 K. Phys. Chem. Liq. 2007, 45, 371–388.

(220)	Reddy, K. V. N. S.; Rao, P. S.; Krishnaiah, A. Experimental and Theoretical Values of Sound Speeds and Viscosities for the Binary Systems of MTBE with Hydrocarbons. J. Mol. Liq. 2007, 135, 14–20.

(221)	Song, S.; Xia, S.; Ma, P.; Di, Z. Densities and Viscosities of N-Formylmorpholine + Benzene, + Toluene at Different Temperatures and Atmospheric Pressures. J. Chem. Eng. Data 2007, 52, 591–595. https://doi.org/10.1021/je060475r.

(222)	Yang, C.; Liu, Z.; Lai, H.; Ma, P. Thermodynamic Properties of Binary Mixtures of N-Methyl-2-Pyrrolidinone with Cyclohexane, Benzene, Toluene at (303.15 to 353.15) K and Atmospheric Pressure. J. Chem. Thermodyn. 2007, 39, 28–38. https://doi.org/10.1016/j.jct.2006.06.005.

(223)	Awwad, A. M.; Abu-Daabes, M. A. Densities, Viscosities, and Excess Properties of (N-Methylmorpholine + Cyclohexane, + Benzene, and + Toluene) at T = (298.15, 303.15, 313.15, 323.15) K. J. Chem. Thermodyn. 2008, 40, 645–652. https://doi.org/10.1016/j.jct.2007.11.002.

(224)	Shukla, R. K.; Shukla, S. K.; Pandey, V. K.; Awasthi, P. Excess Internal Pressure, Excess Energy of Vaporization and Excess Pseudo-Gruneisen Parameter of Binary, Ternary and Quaternary Liquid Mixtures. J. Mol. Liq. 2008, 137, 104–109.

(225)	Song, C.-Y.; Shen, H.-Z.; Zhao, J.-H.; Wang, L.-C.; Wang, F.-A. Densities and Viscosities of Binary Mixtures of Vitamin K3 with Benzene, Toluene, Ethylbenzene, o-Xylene, m-Xylene, and p-Xylene from (303.15 to 333.15) K. J. Chem. Eng. Data 2008, 53, 1110–1115. https://doi.org/10.1021/je7006549.

(226)	Wankhede, D. S.; Wankhede, N.; Lande, M.; Arbad, B. Densities and Viscosities of Propylene Carbonate with Aromatic Hydrocarbons (Benzene, 1,4-Dimethylbenzene and Ethylbenzene) at 288.15, 298.15 and 308.15?K. Phys. Chem. Liq. 2008, 46, 319–327.

(227)	Ali, A.; Nabi, F.; Tariq, M. Volumetric, Viscometric, Ultrasonic, and Refractive Index Properties of Liquid Mixtures of Benzene with Industrially Important Monomers at Different Temperatures. Int. J. Thermophys. 2009, 30, 464–474. https://doi.org/10.1007/s10765-009-0562-x.

(228)	Baylaucq, A.; Watson, G.; Zeberg-Mikkelsen, C.; Bazile, J.-P.; Boned, C. Dynamic Viscosity of the Binary System 1-Propanol + Toluene as a Function of Temperature and Pressure. J. Chem. Eng. Data 2009, 54, 2715–2721. https://doi.org/10.1021/je9002477.

(229)	Nain, A. K.; Chand, D.; Chandra, P.; Pandey, J. D. Excess Internal Pressures, Excess Free Volumes and Excess Thermodynamic Parameters of Some Non-Aqueous Binary Mixtures from Ultrasonic Speed, Density and Viscosity Data. Phys. Chem. Liq. 2009, 47, 195–209. https://doi.org/10.1080/00319100802072664.

(230)	Oswal, S. L.; Ghael, N. Y.; Gardas, R. L. Volumetric and Transport Properties of Ternary Mixtures Containing 1-Propanol + Ethyl Ethanoate + Cyclohexane or Benzene at 303.15 K: Experimental Data, Correlation and Prediction by ERAS Model. Thermochim. Acta 2009, 484, 11–21. https://doi.org/10.1016/j.tca.2008.11.007.

(231)	Sastry, N. V.; Thakor, R. R.; Patel, M. C. Excess Molar Volumes, Viscosity Deviations, Excess Isentropic Compressibilities and Deviations in Relative Permittivities of (Alkyl Acetates (Methyl, Ethyl, Butyl and Isoamyl) + n-Hexane, + Benzene, + Toluene, + (o-, m-, p-) Xylenes, + (Chloro-, Bromo-, Nitro-) Benzene at Temperatures from 298.15 to 313.15 K. J. Mol. Liq. 2009, 144, 13–22.

(232)	Yan, J.-H.; Dai, L.-Y.; Wang, X.-Z.; Chen, Y.-Q. Densities and Viscosities of Binary Mixtures of Cyclopropanecarboxylic Acid with Methanol, Ethanol, Propan-1-Ol, and Butan-1-Ol at Different Temperatures. J. Chem. Eng. Data 2009, 54, 1147–1152. https://doi.org/10.1021/je800930v.

(233)	Ikeuchi, H.; Kanakubo, M.; Okuno, S.; Sato, R.; Fujita, K.; Hamada, M.; Shoda, N.; Fukui, K.; Okada, K.; Kanazawa, H.; Iimori, A.; Miyake, D.; Takeda, T.; Sato, G. P. Densities and Viscosities of Tris(Acetylacetonato)Cobalt(III) Complex Solutions in Various Solvents. J. Solution Chem. 2010, 39, 1428–1453. https://doi.org/10.1007/s10953-010-9598-6.

(234)	Rathnam, M. V.; Jain, K.; Kumar, M. S. Physical Properties of Binary Mixtures of Ethyl Formate with Benzene, Isopropyl Benzene, Isobutyl Benzene, and Butylbenzene at (303.15, 308.15, and 313.15) K. J. Chem. Eng. Data 2010, 55, 1722–1726. https://doi.org/10.1021/je900689m.

(235)	Mariano, A.; Canzonieri, S.; Camacho, A.; Mainar, A.; Postigo, M. Viscometric and Volumetric Properties of Benzene + Methyl Acetate, or + Methyl Propanoate, or + Methyl Butanoate Binary Systems at 283.15, 298.15 and 313.15 K. Phys. Chem. Liq. 2011, 49, 720–728.

(236)	Pradhan, P.; Roy, M. N. Viscous Synergy and Antagonism, Excess Molar Volume, Isoentropic Compressibility and Excess Molar Refraction of Ternary Mixtures Containing Tetrahydrofuran, Methanol and Six Membered Cyclic Compounds at 298.15K. Phys. Chem. Liq. 2011, 49, 286–301. https://doi.org/10.1080/00319100903147886.

(237)	Shukla, R. K.; Awasthi, N.; Kumar, A.; Shukla, A.; Pandey, V. K. Prediction of Associational Behaviour of Binary Liquid Mixtures from Viscosity Data at 298.15, 303.15, 308.15 and 313.15 K. J. Mol. Liq. 2011, 2011, 131–138. https://doi.org/10.1016/j.molliq.2010.11.006.

(238)	Yadava, S. S.; Singh, S.; Bhan, R.; Yadav, N. Viscosities of Binary Mixtures of Ethanenitrile with Benzene and Several Substituted Benzenes. Korean J. Chem. Eng. 2011, 28, 256–260. https://doi.org/10.1007/s11814-010-0319-z.

(239)	Sarkar, B. K.; Choudhury, A.; Sinha, B. Excess Molar Volumes, Excess Viscosities and Ultrasonic Speeds of Sound of Binary Mixtures of 1,2-Dimethoxyethane with Some Aromatic Liquids at 298.15 K. J. Solution Chem. 2012, 41, 53–74. https://doi.org/10.1007/s10953-011-9780-5.

(240)	Yadava, S. S.; Bhan, R.; Yadav, N. Viscosities of Binary Liquid Mixtures of 2-Methylpropane Nitrile and Some Aromatic Hydrocarbons at 303.15 K and 308.15 K. J. Solution Chem. 2012, 41, 926–936. https://doi.org/10.1007/s10953-012-9850-3.

(241)	Bhatia, S. C.; Sangwan, J.; Rani, R.; Bhatia, R. Densities, Viscosities, Speeds of Sound, and Refractive Indices of Binary Mixtures of 2-Ethyl-1-Hexanol with Benzene and Halobenzenes. Int. J. Thermophys. 2013, 34, 2076–2088. https://doi.org/10.1007/s10765-013-1526-8.

(242)	Bhalodia, J.; Sharma, S. Viscometric, Acoustical and Spectroscopic Investigation of b-Pinene with Benzene, Toluene, m-Xylene and Mesitylene at 303.15, 308.15 and 313.15 K and Atmospheric Pressure. J. Solution Chem. 2013, 42, 1794–1815. https://doi.org/10.1007/s10953-013-0073-z.

(243)	Lowry, T. M.; Nasini, A. G. Proc. R. Soc. London, Ser. A 1929, 123, 686.

(244)	Adzumi, H. Studies on the Flow of Gaseous Mixtures through Capillaries. III. The Flow of Gaseous Mixtures at Medium Pressures. Bull. Chem. Soc. Jpn. 1937, 12, 292–303.

(245)	Maczek, A. O. S.; Edwards, C. J. C. The Viscosity and Binary Diffusion Coefficients of Some Gaseous Hydrocarbons, Fluorocarbons and Siloxanes. In Symp. Transp. Prop. Fluids Fluid Mixtures: Their Meas. Estim. Correl. Use; 1979; p 3.5.

(246)	Vogel, E.; Bich, E.; Nimz, R. The Initial Density Dependence of the Viscosity of Organic Vapors: Benzene and Methanol. Physica A (Amsterdam) 1986, 139, 188–207.

(247)	Kuenen, J. P.; Visser, S. W. The Virial Coefficient B for Normal Butane. Versl. Gewone Vergad. Afd. Natuurk-d., K. Ned. Akad. Wet. 1913, 22, 330.

(248)	Sage, B. H.; Yale, W. D.; Lacey, W. N. Effect of Pressure on Viscosity of Butane and Isobutane. Ind. Eng. Chem. 1939, 31, 223.

(249)	Goldhahn, H. Crotyl Bromide. Chem. Tech. (Leipzig) 1955, 7, 16.

(250)	Ratkovics, F.; Salamon, T.; Domonkos, L. Properties of Alcohol-Amine Mixtures, VI A Method for the Determination of the Average Degree of Association. Acta Chim. Acad. Sci. Hung. 1974, 83, 71.

(251)	Wobser, R.; Muller, F. Die Innere Reibung von Gasen Und Dampfen Und Ihre Messung. Kolloidchem. Beih. 1941, 52, 165.

(252)	Dolan, J. P.; Starling, K. E.; Lee, A. L.; Eakin, B. E.; Ellington, R. T. Liquid, Gas and Dense Fluid Viscosity of n-Butane. J. Chem. Eng. Data 1963, 8, 396. https://doi.org/10.1021/je60018a031.

(253)	Golubev, I. F.; Agaev, N. A. Viscosity of Saturated Hydrocarbons; 1964.

(254)	Kestin, J.; Ro, S. T.; Wakeham, W. A. Reference Values of the Viscosity of Twelve Gases at 25 Degrees C. Trans. Faraday Soc. 1971, 67, 2308–2313.

(255)	Diaz Pena, M.; Cheda, J. A. R. Viscosidad De Mezclas De N-Alcanos. An. Quim. 1975, 71, 257–261.

(256)	Kestin, J.; Khalifa, H. E.; Ro, S. T.; Wakeham, W. A. Preliminary Data on the Pressure Effect on the Viscosity of Sodium Chloride-Water Solutions in the Range 10-40 OC. J. Chem. Eng. Data 1977, 22, 207–214. https://doi.org/10.1021/je60073a008.

(257)	Abe, J.; Kestin, J.; Khalifa, H. E.; Wakeham, W. A. The Viscosity and Diffusion Coefficients of the Mixtures of Four Light Hydrocarbon Gases. Physica A (Amsterdam) 1978, 93, 155–170.

(258)	Abe, J.; Kestin, J.; Khalifa, H. E.; Wakeham, W. A. The Viscosity of Normal Butane, Isobutane, and Their Mixtures. Physica A (Amsterdam) 1979, 97, 296.

(259)	Lipkin, M. R.; Davison, J. A.; Kurtz, S. S. Viscosity of Propane, Butane, and Isobutane. Ind. Eng. Chem. 1942, 34, 976.

(260)	Carmichael, L. T.; Sage, B. H. Viscosity of Ethane at High Pressures. J. Chem. Eng. Data 1963, 8, 94–98. https://doi.org/10.1021/je60016a028.

(261)	Golubev, I. F.; Agaev, N. A. Generalization of the Viscosity Data for Saturated Hydrocarbons at Various Temperatures and Pressures. Dokl. Akad. Nauk SSSR 1963, 151, 875–878.

(262)	Diller, D. E.; Van Poolen, L. J. Measurements of the Viscosities of Saturated and Compressed Liquid Normal Butane and Isobutane. Int. J. Thermophys. 1985, 6, 43–62. https://doi.org/10.1007/bf00505791.

(263)	Kiran, E.; Sen, Y. L. High-Pressure Viscosity and Density of n-Alkanes. Int. J. Thermophys. 1992, 13, 411–442. https://doi.org/10.1007/bf00503880.

(264)	Kumagai, A.; Tomida, D.; Yokoyama, C. Measurements of the Liquid Viscosities of Mixtures of N-Butane, n-Hexane, and n-Octane with Squalane to 30MPa. Int. J. Thermophys. 2006, 27, 376–393. https://doi.org/10.1007/s10765-006-0053-2.

(265)	Bingham, E. C.; Fornwalt, H. J. Chemical Consitution and Association. J. Rheol. 1930, 1, 372–417.

(266)	Shepard, A. F.; Henne, A. L.; Midgley, T. Physical Properties of the Normal Paraffin Hydrocarbons Pentane to Dodecane. J. Am. Chem. Soc. 1931, 53, 1948–1958. https://doi.org/10.1021/ja01356a052.

(267)	Doolittle, A. K.; Peterson, R. H. Preparation of Physical Properties of a Series of N-Alkanes. J. Am. Chem. Soc. 1951, 73, 2145–2151. https://doi.org/10.1021/ja01149a069.

(268)	Rastorguev, Yu. L.; Keramidi, A. S. Experimental Study of the Viscosity of N-Undecane at High Pressures. Izv. Vyssh. Uchebn. Zaved., Neft Gaz 1971, 59–63.

(269)	Guseinov, S. O.; Naziev, Y. M. Viscosity of N-Undecane and n-Heptadecane at High Pressures. Izv. Vyssh. Uchebn. Zaved., Neft Gaz 1973, 16, 61–63.

(270)	Ratkovics, F.; Salamon, T.; Domonkos, L. Properties of Mixtures Containing Amines and Alcohols. IV. Viscosities and Average Degrees of Association in n-Butylamine - 1-Butanol Mixtures. Magy. Kem. Foly. 1974, 80, 155–159.

(271)	Iwahashi, M.; Yamaguchi, Y.; Ogura, Y.; Suzuki, M. Dynamical Structures of Normal Alkanes, Alcohols. and Fatty Acids in the Liquid State as Determined by Viscosity, Self-Diffusion Coefficient, Infrared Spectra, and C NMR Spin-Lattice Relaxation Time Measurements. Bull. Chem. Soc. Jpn. 1990, 63, 2154–2158.

(272)	Assael, M. J.; Papadaki, M. Measurements of the Viscosity of N-Heptane, n-Nonane, and n-Undecane at Pressures up to 70 MPa. Int. J. Thermophys. 1991, 12, 801–810. https://doi.org/10.1007/bf00502407.

(273)	Zhang, L.; Guo, Y.; Wei, H.; Yang, F.; Fang, W.; Lin, R. Densities and Viscosities of Binary Mixtures of Exo-Tetrahydrodicyclopentadiene with N-Undecane or N-Tetradecane at T = (293.15 to 313.15) K. J. Chem. Eng. Data 2010, 55, 4108–4113. https://doi.org/10.1021/je100313a.

(274)	Chi, H.; Li, G.; Guo, Y.; Xu, L.; Fang, W. Excess Molar Volume along with Viscosity, Flash Point, and Refractive Index for Binary Mixtures of Cis-Decalin or Trans-Decalin with C9 to C11 n-Alkanes. J. Chem. Eng. Data 2013, 58, 2224–2232. https://doi.org/10.1021/je400250u.

(275)	Dymond, J. H.; Young, K. J. Transport Properties of Nonelectrolyte Liquid Mixtures I. Viscosity Coefficients for n-Alkane Mixtures at Saturation Pressure from 283 to 378 K. Int. J. Thermophys. 1980, 1, 331–344. https://doi.org/10.1007/bf00516562.

(276)	Evans, E. B. The Viscosity of Hydrocarbons. Parts IV-VI. J. Inst. Pet. 1938, 24, 321–327.

(277)	Cutler, W. G. A Study of the Compressions of Several High Molecular Weight Hydrocarbons. PhD Thesis, Pennsylvania State University, 1955.

(278)	Hogenboon, D. L.; Webb, W.; Dixon, J. A. Viscosity of Several Liquid Hydrocarbons as a Function of Temperature, Pressure, and Free Volume. J. Chem. Phys. 1967, 46, 2586–2597.

(279)	Anonymous, R. Properties of Hydrocarbon of High Molecular Weight; Univ.; Am. Pet. Inst. Res. Proj. 42, 1968.

(280)	Keramidi, A. S.; Rastorguev, Yu. L. Study of the Viscosity of N-Dodecane at High Pressures. Izv. Vyssh. Uchebn. Zaved., Neft Gaz 1970, 108–114.

(281)	Jambon, C.; Delmas, G. Viscosities of Mixtures of SnR4 Compounds and of Some Other Large Globular Molecules. Effect of Size Difference. Can. J. Chem. 1977, 55, 1360–1366. https://doi.org/10.1139/v77-188.

(282)	Awwad, A. M.; Allos, E. I. Thermodynamic Properties of Binary Mixtures of Isooctane and N-Alkane at 298.15 K. Fluid Phase Equilib. 1985, 22, 353–366. https://doi.org/10.1016/0378-3812(85)87031-x.

(283)	Aucejo, A.; Part, E.; Medina, P.; Sancho-Tello, M. Viscosity of Some N-Alkane/1-Chloroalkane Binary Liquid Mixtures. J. Chem. Eng. Data 1986, 31, 143–145. https://doi.org/10.1021/je00044a003.

(284)	Awwad, A. M.; Salman, M. A. Excess Molar Volumes and Viscosities of Binary Mixtures of Cyclohexane and N-Alkane at 298.15 K. Fluid Phase Equilib. 1986, 25, 195–208. https://doi.org/10.1016/0378-3812(86)80015-2.

(285)	Ducoulombier, D.; Zhou, H.; Boned, C.; Peyrelasse, J.; Saint-Guirons, H.; Xans, P. Pressure (1-1000 Bars) and Temperature (20-100 C) Dependence of the Viscosity of Liquid Hydrocarbons. J. Phys. Chem. 1986, 90, 1692.

(286)	Wakefield, D. L.; Marsh, K. N. Viscosities of Nonelectrolyte Liquid Mixt. I. n-Hexadecane + n-Octane. Int. J. Thermophys. 1987, 8, 649–662. https://doi.org/10.1007/bf00500785.

(287)	Wakefield, D. L. Viscosities of Nonelectrolyte Liquid Mixtures. III. Selected Binar and Quaternary Mixtures. Int. J. Thermophys. 1988, 9, 365–381. https://doi.org/10.1007/bf00513077.

(288)	Knapstad, B.; Skjolsvik, P. A.; Oye, H. A. Viscosity of Three Binary Hydrocarbon Mixtures. J. Chem. Eng. Data 1991, 36, 84–88. https://doi.org/10.1021/je00001a025.

(289)	Aminabhavi, T. M.; Gopalkrishna, B. Densities, Viscosities, Refractive Indices, and Speeds of Sound of the Binary Mixtures of Bis(2-Methoxyethyl) Ether with Nonane,Decane, Dodecane, Tetradecane, and Hexadecane at 298.15, 308.15, and 31. J. Chem. Eng. Data 1994, 39, 529–534. https://doi.org/10.1021/je00015a029.

(290)	Aminabhavi, T. M.; Gopalakrishna, B. Density, Viscosity, Refractive Index, and Speed of Sound in Aqueous Mixtures of N,N-Dimethylformamide, Dimethyl Sulfoxide, N,N- Dimethylacetamide, Acetonitrile, Ethylene Glycol, Diethylene Glycol, 1,. J. Chem. Eng. Data 1995, 40, 856–861. https://doi.org/10.1021/je00020a026.

(291)	Aminabhavi, T. M.; Patil, V. B. Density, Refractive Index, Viscosity, and Speed of Sound in Binary Mixtures of Ethenylbenzene with Hexane, Heptane, Octane, Nonane, Decane, and Dodecane. J. Chem. Eng. Data 1997, 42, 641–646. https://doi.org/10.1021/je960382h.

(292)	Nayak, J. N.; Aralaguppi, M. I.; Aminabhavi, T. M. Density, Viscosity, Refractive Index, and Speed of Sound for the Binary Mixtures of Ethyl Chloroacetate with n-Alkanes (C6 to C12) at (298.15, 303.15, and 308.15) K. J. Chem. Eng. Data 2001, 46, 891–896. https://doi.org/10.1021/je010020w.

(293)	Garcia, B.; Alcalde, R.; Aparicio, S.; Leal, J. M. Thermophysical Behavior of Methylbenzoate + N-Alkanes Mixed Solvents. Application of Cubic Equations of State and Viscosity Models. Ind. Eng. Chem. Res. 2002, 41, 4399–4408. https://doi.org/10.1021/ie020008c.

(294)	Gonzalez, B.; Dominguez, A.; Tojo, J. Viscosities, Densities and Speeds of Sound of the Binary Systems: 2-Propanol with Octane, or Decane, or Dodecane at T=(293.15, 298.15, and 303.15) K. J. Chem. Thermodyn. 2003, 35, 939–953. https://doi.org/10.1016/s0021-9614(03)00047-8.

(295)	Moreiras, A. F.; Garcia, J.; Lugo, L.; Comunas, M. J. P.; Lopez, E. R.; Fernandez, J. Experimental Densities and Dynamic Viscosities of Organic Carbonate + N-Alkane or p-Xylene Systems at 298.15 K. Fluid Phase Equilib. 2003, 204, 233–243. https://doi.org/10.1016/s0378-3812(02)00261-3.

(296)	Caudwell, D. R. Viscosity of Dense Fluid Mixtures. PhD Thesis, Imperial College, 2004.

(297)	Caudwell, D.; Goodwin, A. R. H.; Trusler, J. P. M. A Robust Vibrating Wire Viscometer for Reservoir Fluids: Results for Toluene and n-Decane. J. Pet. Sci. Eng. 2004, 44, 333–340. https://doi.org/10.1016/j.petrol.2004.02.019.

(298)	Gonzalez, B.; Dominguez, A.; Tojo, J. Dynamic Viscosities, Densities, and Speed of Sound and Derived Properties of the Binary Systems Acetic Acid with Water, Methanol, Ethanol, Ethyl Acetate and Methyl Acetate at T = (293.15, 298.15, and 303.15) K at Atmospheric Pressure. J. Chem. Eng. Data 2004, 49, 1590–1596. https://doi.org/10.1021/je0342825.

(299)	Gonzalez, B.; Dominguez, A.; Tojo, J. Dynamic Viscosities of 2-Butanol with Alkanes (C8, C10, and C12) at Several Temperatures. J. Chem. Thermodyn. 2004, 36, 267–275. https://doi.org/10.1016/j.jct.2003.12.005.

(300)	Aljimaz, A. S.; Al-Kandary, J. A.; Abdul-Latif, A.-H. M.; Al-Zanki, A. M. Physical Properties of (Anisole + n-Alkanes) at Temperatures between (293.15 and 303.15) K. J. Chem. Thermodyn. 2005, 37, 631–642. https://doi.org/10.1016/j.jct.2004.09.021.

(301)	Goodwin, A. R. H.; Donzier, E. P.; Fitt, A. D.; Ronaldson, K. A.; Wakeham, W. A.; Manrique de Lara, M.; Marty, F.; Mercier, B. A Vibrating Edge Supported Plate, Fabricated by the Methods of Micro Electro Mechanical System for the Simultaneous Measurement of Density and Viscosity: Results for Methylbenzene and Octane at Temperatures between (323 and 423) K and Pressures in the Range (0.1 to 68) MPa. J. Chem. Eng. Data 2006, 51, 190–208. https://doi.org/10.1021/je0503296.

(302)	Tian, Q.; Liu, H. Densities and Viscosities of Binary Mixtures of Tributyl Phosphate with Hexane and Dodecane from (298.15 to 328.15) K. J. Chem. Eng. Data 2007, 52, 892–897. https://doi.org/10.1021/je060491o.

(303)	Zhang, L.; Yongsheng, G.; Xiao, J.; Gong, X.; Fang, W. Density, Refractive Index, Viscosity, and Surface Tension of Binary Mixtures of Exo-Tetrahydrodicyclopentadiene with Some n-Alkanes from (293.15 to 313.15) K. J. Chem. Eng. Data 2011, 56, 4268–4273. https://doi.org/10.1021/je200757a.

(304)	Mahajan, A. R. R. ].; Mirgane, S. R. R. ]. Study of Thermodynamic and Transport Properties of Binary Liquid Mixtures of N-Decane with Hexan-2-Ol, Heptan-2-Ol and Octan-2-Ol at T = 298.15 K. Experimental Results and Application of the Prigogine-Flory-Patterson Theory. J. Chem. Thermodyn. 2013, 64, 159–166. https://doi.org/10.1016/j.jct.2013.05.014.

(305)	Blahusiak, M.; Schlosser, S. Physical Properties of Phosphonium Ionic Liquid and Its Mixtures with Dodecane and Water. J. Chem. Thermodyn. 2014, 72, 54–64. https://doi.org/10.1016/j.jct.2013.12.022.

(306)	Lago, S.; Rodriguez-Cabo, B.; Arce, A.; Soto, A. Water/Oil/[P6,6,6,14][NTf2] Phase Equilibria. J. Chem. Thermodyn. 2014, 75, 63–68. https://doi.org/10.1016/j.jct.2014.02.012.

(307)	Liu, H.; Zhu, L. Excess Molar Volumes and Viscosities of Binary Systems of Butylcyclohexane with N-Alkanes (C7 to C14) at T = 293.15 K to 313.15 K. J. Chem. Eng. Data 2014, 59, 369–375. https://doi.org/10.1021/je400835u.

(308)	Prak, D. J. L.; Alexandre, S. M.; Cowart, J. S.; Trulove, P. C. Density, Viscosity, Speed of Sound, Bulk Modulus, Surface Tension, and Flash Point of Binary Mixtures of n-Dodecane with 2,2,4,6,6-Pentamethylheptane or 2,2,4,4,6,8,8-Heptamethylnonane. J. Chem. Eng. Data 2014, 59, 1334–1346. https://doi.org/10.1021/je5000132.

(309)	Singh, M. L.; Tripathi, S.; Lokhande, M.; Gandhi, P.; Gaikar, V. Density, Viscosity, and Interfacial Tension of Binary Mixture of Tri-Iso-Amyl Phosphate (TiAP) and n-Dodecane: Effect of Compositions and Gamma Absorbed Doses. J. Chem. Eng. Data 2014, 59, 1130–1139. https://doi.org/10.1021/je400493x.

(310)	Slusar’, V. P.; Rudenko, N. S.; Tret’yakov, V. M. Experimental Study of the Viscosity of Simple Substances along the Saturation Curve and under Pressure. Ukr. Fiz. Zh. 1973, 18, 190–194.

(311)	Coursey, B. M.; Heric, E. L. Viscosity of Some Binary Systems of Hexadecane and Normal Choroalkanes. J. Chem. Eng. Data 1969, 14, 426–430. https://doi.org/10.1021/je60043a015.

(312)	Rastorguev, Yu. L.; Keramidi, A. S. Viscosity of Hydrocarbons. Izv. Vyssh. Uchebn. Zaved., Neft Gaz 1972, 15, 61–66.

(313)	Matthews, M. A.; Rodden, J. B.; Akgerman, A. High-Temperature Diffusion, Viscosity, and Density Measurements in n-Hexadecane. J. Chem. Eng. Data 1987, 32, 317–319. https://doi.org/10.1021/je00049a011.

(314)	Dandekar, A. Y.; Andersen, S. I.; Stenby, E. H. Measurement of Viscosity of Hydrocarbon Liquids Using a Microviscometer. J. Chem. Eng. Data 1998, 43, 551–554. https://doi.org/10.1021/je9702807.

(315)	Fermeglia, M.; Torriano, G. Density, Viscosity, and Refractive Index for Binary Systems of n-C16 and Four Nonlinear Alkanes at 298.15 K. J. Chem. Eng. Data 1999, 44, 965–969. https://doi.org/10.1021/je9900171.

(316)	Queimada, A. J.; Quinones-Cisneros, S. E.; Marrucho, I. M.; Coutinho, J. A. P.; Stendy, E. H. Viscosity and Liquid Density of Asymmetric Hydrocarbon Mixtures. Int. J. Thermophys. 2003, 24, 1221–1239. https://doi.org/10.1023/a:1026142932619.

(317)	Tripathi, N. Densities, Viscosities, and Refractive Indices of Mixtures of Hexane with Cyclohexane, Decane, Hexadecane, and Squalane at 298.15K. Int. J. Thermophys. 2005, 26, 693–703. https://doi.org/10.1007/s10765-005-5572-8.

(318)	Aguila-Hernandez, J.; Trejo, A.; Garcia-Flores, B. E.; Molnar, R. Viscometric and Volumetric Behaviour of Binary Mixtures of Sulfolane and N-Methylpyrrolidone with Monoethanolamine and Diethanolamine in the Range 303 373 K. Fluid Phase Equilib. 2008, 267, 172–180. https://doi.org/10.1016/j.fluid.2008.02.023.

(319)	Dubey, G. P.; Sharma, M. Temperature and Composition Dependence of the Densities, Viscosities, and Speeds of Sound of Binary Liquid Mixtures of 1-Butanol with Hexadecane and Squalane. J. Chem. Eng. Data 2008, 53, 1032–1038. https://doi.org/10.1021/je7007654.

(320)	Dubey, G. P.; Sharma, M. Studies of Mixing Properties of Binary Systems of 2-Propanol with Hexadecane and Squalane at T = (298.15, 303.15, and 308.15) K. J. Chem. Thermodyn. 2009, 41, 115–122. https://doi.org/10.1016/j.jct.2008.07.010.

(321)	Rajagopal, K.; Andrade, L. L. P. R.; Paredes, M. L. L. High-Pressure Viscosity Measurements for the Binary System Cyclohexane + n-Hexadecane in the Temperature Range of (318.15 to 413.15) K. J. Chem. Eng. Data 2009, 54, 2967–2970. https://doi.org/10.1021/je9000262.

(322)	Prak, D. J. L.; Morris, R. E.; Cowart, J. S.; Hamilton, L. J.; Trulove, P. C. Density, Viscosity, Speed of Sound, Bulk Modulus, Surface Tension, and Flash Point of Direct Sugar to Hydrocarbon Diesel (DSH-76) and Binary Mixtures of N-Hexadecane and 2,2,4,6,6-Pentamethylheptane. J. Chem. Eng. Data 2013, 58, 3536–3544. https://doi.org/10.1021/je400839x.

(323)	Baled, H. O.; Xing, D.; Katz, H.; Tapriyal, D.; Gamwo, I. K.; Soong, Y.; Bamgbade, B. A.; Wu, Y.; McHugh, M. A.; Enick, R. M. Viscosity of N-Hexadecane, n-Octadecane and n-Eicosane at Pressures up to 243 MPa and Temperatures up to 534 K. J. Chem. Thermodyn. 2014, 72, 108–116. https://doi.org/10.1016/j.jct.2014.01.008.

(324)	Vorlander, D.; Walter, R. Z. Phys. Chem. (Leipzig) 1925, 118, 1.

(325)	Godchot, M.; Cauquil, G. On the Viscosities, Surface Tensions and Parachors of Some Cyclic Hydrocarbons (in French). C. R. Hebd. Seances Acad. Sci. 1931, 192, 1560.

(326)	Hugel, G. Relation Between the Viscosity and Constitution of Hydrocarbons of High Molecular Weight. Chim. Ind. (Paris) 1931, 26, 1282–1288.

(327)	Friend, J. N.; Hargreaves, W. D. VII. Viscosities at the Boiling Point of Some Primary Amines, Cyclohexane and Some of Its Derivatives. Philos. Mag. Ser. 7 1944, 35, 57–64.

(328)	Jonas, J.; Hasha, D.; Huang, S. G. Self-Diffusion and Viscosity of Methylcyclohexane in the Dense Liquid Region. J. Chem. Phys. 1979, 71, 3996.

(329)	Et-Tahir, A.; Boned, C.; Lagourette, B.; Xans, P. Determination of the Viscosity of Various Hydrocarbons and Mixtures of Hydrocarbons versus Temperature and Pressure. Int. J. Thermophys. 1995, 16, 1309–1334. https://doi.org/10.1007/bf02083543.

(330)	Oswal, S. L.; Oswal, P.; Phalak, R. P. Viscosity of Nonelectrolyte Liquid Mixtures. IV. Binary Mixtures Containing p-Dioxane. Int. J. Thermophys. 1996, 17, 1255–1267. https://doi.org/10.1007/bf01438668.

(331)	Baylaucq, A.; Dauge, P.; Boned, C. Viscosity and Density of the Ternary Mixture Heptane + Methylcyclohexane + 1 -Methylnaphthalene. Int. J. Thermophys. 1997, 18, 1089–1107. https://doi.org/10.1007/bf02575251.

(332)	Zeberg-Mikkelsen, C. K.; Barrouhou, M.; Boned, C. Viscosity and Density Measurements of Binary Mixtures Composed of Methylcyclohexane + Cis-Decalin Versus Temperature and Pressure. Int. J. Thermophys. 2003, 24, 361–374. https://doi.org/10.1023/a:1022911703225.

(333)	Pereiro, A. B.; Rodriguez, A.; Canosa, J.; Tojo, J. Density, Viscosity, and Speed of Sound of Dialkyl Carbonates with Cyclopentane and Methyl Cyclohexane at Several Temperatures. J. Chem. Eng. Data 2004, 49, 1392–1399. https://doi.org/10.1021/je049895b.

(334)	Oswal, S. L.; Prajapati, K. D.; Oswal, P.; Ghael, N. Y.; Ijardar, S. P. Viscosity of Binary Mixtures of 1-Alkanol+cyclohexane, 2-Alkanol+cyclohexane and 1-Alkanol+methylcyclohexane at 303.15 K. J. Mol. Liq. 2005, 116, 73–82.

(335)	Ye, J.-D.; Tu, C.-H. Densities and Viscosities for Binary and Ternary Mixtures of Diisopropyl Ether, Ethanol, and Methylcyclohexane. J. Chem. Eng. Data 2005, 50, 1060–1067. https://doi.org/10.1021/je050031f.

(336)	Aralaguppi, M. I.; Baragi, J. G. Physico-Chemical and Excess Properties of the Binary Mixtures of Methylcyclohexane + Ethanol, + Propan-1-Ol, + Propan-2-Ol, + Butan-1-Ol, + 2-Methyl-1-Propanol, or 3-Methyl-1-Butanol at T = (298.15, 303.15, and 308.15) K. J. Chem. Thermodyn. 2006, 38, 434–442. https://doi.org/10.1016/j.jct.2005.06.011.

(337)	Baragi, J. G.; Aralaguppi, M. I. Excess and Deviation Properties for the Binary Mixtures of Methylcyclohexane with Benzene, Toluene, p-Xylene, Mesitylene, and Anisole at T = (298.15, 303.15, and 308.15) K. J. Chem. Thermodyn. 2006, 38, 1717–1724. https://doi.org/10.1016/j.jct.2005.12.005.

(338)	Davies, M. K.; Archer, A. L.; Maczek, A. O. S.; Manzanares-Papayanopoulos, E.; McLure, I. A. Shear Viscosities of Methycyclohexane, Perfluoromethylcyclohexane, and Their Mixtures in the Vicinity of the Upper Critical Mixing Temperature. 1. Critical Isopleth and Coexistence Curve. J. Chem. Eng. Data 2006, 51, 1502–1508. https://doi.org/10.1021/je0502593.

(339)	Iloukhani, H.; Rezaei-Sameti, M.; Basiri-Parsa, J.; Azizian, S. Studies of Dynamic Viscosity and Gibbs Energy of Activation of Binary Mixtures of Methylcyclohexane with N-Alkanes (C5 C10) at Various Temperatures. J. Mol. Liq. 2006, 126, 117–123.

(340)	Iloukhani, H.; Samiey, B. Excess Molar Volumes, Viscosities, and Speeds of Sound of the Ternary Mixture (1-Heptanol (1) + Trichloroethylene (2) + Methylcyclohexane (3)) at T = 298.15 K. J. Chem. Thermodyn. 2007, 39, 206–217. https://doi.org/10.1016/j.jct.2006.07.015.

(341)	Yang, X.; Xing, Y.; Li, D.; Guo, Y.; Fang, W. Volumetric and Viscous Properties at Several Temperatures for Binary Mixtures of N-Methylpiperazine with Methylcyclohexane or n-Heptane. J. Chem. Eng. Data 2010, 55, 2914–2916. https://doi.org/10.1021/je900969u.

(342)	Ranjbar, S.; Momenian, S. H. Densities and Viscosities of Binary and Ternary Mixtures of (Nitrobenzene + 1-Bromobutane), (1-Bromobutane + Methylcyclohexane), (Nitrobenzene + Methylcyclohexane), and (Methylcyclohexane + Nitrobenzene + 1-Bromobutane) from (293.15 to 308.15) K. J. Chem. Eng. Data 2011, 56, 3949–3954. https://doi.org/10.1021/je200350a.

(343)	Xing, Y.; Yang, X.; Fang, W.; Guo, Y.; Lin, R. Measurement on Vapor Pressure, Density and Viscosity for Binary Mixtures of JP-10 and Methylcyclohexane. Fluid Phase Equilib. 2011, 305, 192–196. https://doi.org/10.1016/j.fluid.2011.03.030.

(344)	Li, G.; Chi, H.; Fang, W.; Hu, S. Excess Molar Volume along with Viscosity and Refractive Index for Binary Systems of Tricyclo[5.2.1.0(2.6)]Decane with Five Cycloalkanes. J. Chem. Eng. Data 2013, 58, 3078–3086. https://doi.org/10.1021/je400529k.

(345)	Jiang, X.; He, G.; Wu, X.; Guo, Y.; Fang, W.; Xu, L. Density, Viscosity, Refractive Index, and Freezing Point for Binary Mixtures of 1,1’-Bicyclohexyl with Alkylcyclohexane. J. Chem. Eng. Data 2014, 59, 2499–2504. https://doi.org/10.1021/je500275j.

(346)	Keramidi, A. S.; Rastorguev, Yu. L.; Grigor’ev, B. A. Experimental Investigation of the Dynamic Viscosity Coefficient of N-Hydrocarbons. Teplofiz. Svoist. Zhidk. 1976, 90–96.

(347)	Queimada, A. J.; Marrucho, I. M.; Coutinho, J. A. P.; Stenby, E. H. Viscosity and Liquid Density of Asymmetric N-Alkane Mixtures: Measurement and Modeling. Int. J. Thermophys. 2005, 26, 47–61. https://doi.org/10.1007/s10765-005-2352-4.

(348)	Trautz, M.; Husseini, I. Die Reibung, Warmeleitung Und Diffusion Im Gasmischungen. Ann. Phys. (Berlin) 1934, 20, 121.

(349)	Sargent, J. W.; Seffl, R. J. Properties of Perfluorinated Liquids. Fed. Proc. 1970, 29, 1699–1703.

(350)	Morgado, P. J. R. Semifluorinated Alkanes - Structure - Properties Relations. Thesis 2011, 1–196.

(351)	Cochran, M. A.; North, A. M.; Pethrick, R. A. Ultrasonic Studies of Perfluoro-n-Alkanes. J. Chem. Soc., Faraday Trans. 1974, 70, 1274.

(352)	Freire, M. G.; Ferreira, A. G. M.; Fonseca, I. M. A.; Marrucho, I. M.; Coutinho, J. A. P. Viscosities of Liquid Fluorocompounds. J. Chem. Eng. Data 2008, 53, 538–542. https://doi.org/10.1021/je700632z.

(353)	Morgado, P.; Black, J.; Lewis, J. B.; Iacovella, C. R.; McCabe, C.; Martins, L. F. G.; Filipe, E. J. M. Viscosity of Liquid Systems Involving Hydrogenated and Fluorinated Substances: Liquid Mixtures of (Hexane + Perfluorohexane). Fluid Phase Equilib. 2013, 358, 161–165. https://doi.org/10.1016/j.fluid.2013.07.060.

(354)	Duan, Y.-Y.; Shi, L.; Zhu, M.-S.; Han, L.-Z. Critical Parameters and Saturated Density of Trifluoroiodomethane (CF3I). J. Chem. Eng. Data 1999, 44, 501–504. https://doi.org/10.1021/je980251b.

(355)	Trautz, M.; Winterkorn, H. The Viscosity, Thermal Conductivity and Diffusion in Gases - XVIII. Measurements of Viscosity on Aggressive Gases (Cl2, HI). Ann. Phys. (Leipzig) 1931, 10, 511–528. https://doi.org/10.1002/andp.19314020406.

(356)	Braune, H.; Linke, R. The Internal Friction Some Gases and Vapors. III. Influence of the Dipole Moment on the Size of the Sutherland Constant. Z. Phys. Chem. Abt. A 1930, 148, 195–215.

(357)	Steacie, E. W. R.; Johnson, F. M. G. The Viscosities of the Liquid Halogens. J. Am. Chem. Soc. 1925, 47, 754–762. https://doi.org/10.1021/ja01680a022.

(358)	van Aubel, E. Sur La Viscosite Du Chlore Liquide. Bull. Cl. Sci. Acad. R. Belg. 1926, 12, 369–373.

(359)	Trautz, M.; Ruf, F. Viscosity, Thermal Conductivity and Diffusion in Gas Mixtures. XXVII. Internal Viscosity of Chlorine and of Hydrogen Iodide. A Test of Methods of Viscosity Measurements on Corrosive Gases. Ann. Phys. (Berlin) 1934, 20, 127.

(360)	Heston, W. M.; Hennelly, E. J.; Smyth, C. P. Dielectric Constants, Viscosities, Densities, Refractive Indices and Dipole Moment Calculations for Some Organic Halides. J. Am. Chem. Soc. 1950, 72, 2071–2075. https://doi.org/10.1021/ja01161a054.

(361)	Mumford, S. A.; Phillips, J. W. C. 19. The Physical Properties of Some Aliphatic Compounds. J. Chem. Soc. 1950, 75–84.

(362)	Fort, R. J.; Moore, W. R. Viscosities of Binary Mixtures of Benzyl Acetate with Dioxane. Trans. Faraday Soc. 1966, 62, 1112.

(363)	Nigam, R. K.; Singh, P. P. Viscosity and Molecular Interactions in Binary Mixtures of Benzene and Toluene with Chloro- and Bromobenzenes. Indian J. Chem. 1971, 9, 691–695.

(364)	Abdullaev, F. G.; Djafarova, N. I. Experimental Study of the Dynamic Viscosity Coefficient of Chlorbenzene. Izv. Vyssh. Uchebn. Zaved., Neft Gaz 1980, 23, 89–90.

(365)	Singh, R. P.; Sinha, C. P. Dielectric Behavior of the Binary Mixtures of N-Hexane with Toluene, Chlorobenzene, and 1-Hexanol. J. Chem. Eng. Data 1982, 27, 283–287. https://doi.org/10.1021/je00029a016.

(366)	Koshechko, V. G.; Krylov, V. A. Thermodynamic Equilibrium of the Ionic Pairs of the Kation -Radical Salts in Acetonitrile and Chlorobenzene. Zh. Fiz. Khim. 1984, 58, 1334–1339.

(367)	Singh, R. P.; Sinha, C. P. Viscosities and Activation Energies of Viscous Flow of the Binary Mixtures of N-Hexane with Toluene, Chlorobenzene, and 1-Hexanol. J. Chem. Eng. Data 1984, 29, 132–135. https://doi.org/10.1021/je00036a010.

(368)	Singh, R. P.; Sinha, C. P. Viscosities and Acyivation Energies of Viscous Flow of Ternary Mixtures of Toluene, Chlorobenzene, 1-Hexanol and Benzyl Alcohol. J. Chem. Eng. Data 1985, 30, 470–474. https://doi.org/10.1021/je00042a031.

(369)	Gokavi, G. S.; Raju, J. R.; Aminabhavi, T. M.; Balundgi, R. H.; Muddapur, M. V. Viscosities and Densities of Binary Liquid Mixtures of Dimethyl Sulfoxide with Chlorobenzene, Pyridine, and Methyl Ethyl Ketone at 25 35, 45, and 55 Deg C. J. Chem. Eng. Data 1986, 31, 15–18. https://doi.org/10.1021/je00043a004.

(370)	Singh, R. P.; Sinha, C. P.; Singh, B. N. Viscosities, Densities, and Activation Energies of Viscous Flow of the Ternary Systems n-Hexane-Benzyle Alcohol-TOluene, n-Hexane-Benzyl Alcohol-Chlorobenzene, and n-Hexane-Benzyl Alcohol-1-Hexanol a. J. Chem. Eng. Data 1986, 31, 107–111. https://doi.org/10.1021/je00043a031.

(371)	Ramadevi, R. S.; Venkatesu, P.; Venkata, M.; Rao, P. Viscosities of Binary Liquid Mixtures of N,N-Dimethylformamide with Substituted Benzenes at 303.15 and 313.15 K. J. Chem. Eng. Data 1996, 41, 479–481. https://doi.org/10.1021/je950187n.

(372)	Artigas, H.; Cea, P.; Mainar, A. M.; Pardo, J.; Royo, F. M. EXCESS VOLUMES AND EXCESS VISCOSITIES OF MIXTURES HALOHYDROCARBON + 2-BUTANOL AT 298.15 AND 313.15 K. Phys. Chem. Liq. 1997, 35, 105–119.

(373)	Artigas, H.; Dominguez, M.; Mainar, A. M.; Lopez, M. C.; Royo, F. M. Densities and Viscosities of Binary Mixtures of Some Halohydrocarbons with 2-Methyl-1-Propanol at 298.15 and 313.15 K. J. Chem. Eng. Data 1998, 43, 580–584. https://doi.org/10.1021/je970268e.

(374)	Nayak, J. N.; Aralaguppi, M. I.; Aminabhavi, T. M. Density, Viscosity, Refractive Index, and Speed of Sound in the Binary Mixtures of Ethyl Chloroacetate + Cyclohexanone, + Chlorobenzene, + Bromobenzene, or + Benzyl Alcohol at (298.15, 303.15, and 308.15) K. J. Chem. Eng. Data 2003, 48, 628–631. https://doi.org/10.1021/je0201828.

(375)	Li, N.; Ma, P.; Jin, H.; Yang, C. Densities and Viscosities for Correlative System of Dimethyl Carbonate at Different Temperatures. Gaoxiao Huaxue Gongcheng Xuebao 2004, 18, 7;766.

(376)	Oswal, S. L.; Desai, J. S.; Ijardar, S. P.; Malek, N. I. Studies of Viscosities of Dilute Solutions of Alkylamine in Non-Electrolyte Solvents. II. Haloalkanes and Other Polar Solvents. Thermochim. Acta 2005, 427, 51–60. https://doi.org/10.1016/j.tca.2004.08.013.

(377)	Bhuiyan, M. M. H.; Ferdaush, J.; Uddin, M. H. Densities and Viscosities of Binary Mixtures of (Dimethylsulfoxide + Aliphatic Lower Alkanols (C1 C3)) at Temperatures from T = 303.15 K to T = 323.15 K. J. Chem. Thermodyn. 2007, 39, 675–683. https://doi.org/10.1016/j.jct.2006.10.016.

(378)	Ali, A.; Nabi, F.; Itoo, F. A.; Tasneem, S. Volumetric, Ultrasonic, Viscometric and Refractive Index Behaviour of Binary Mixtures of Styrene with Ethylalcohol and Chlorobenzene at Different Temperatures (T=298.15 K to 313.15 K). J. Mol. Liq. 2008, 143, 141–146.

(379)	Baskaran, R.; Kubendran, T. R. Thermo Physical Properties of 4-Hydroxy 4-Methyl Pentanone with Nitrobenzene or Ethyl Benzene at Temperatures of (303.15, 313.15, and 323.15) K and a Pressure of 0.1 MPa. J. Chem. Eng. Data 2008, 53, 1956–1961. https://doi.org/10.1021/je8002699.

(380)	Sastry, N. V.; Thakor, R. R.; Patel, M. C. Thermophysical Properties For Diethylene Glycol + Nitrobenzene and Triethylene Glycol + (Chloro-, Bromo-, Nitro-) Benzene Systems at Different Temperatures. Int. J. Thermophys. 2008, 29, 610–618. https://doi.org/10.1007/s10765-008-0399-8.

(381)	Shukla, D.; Singh, S.; Parveen, S.; Gupta, M.; Shukla, J. P. Thermophysical Properties of Binary Mixtures of Methanol with Chlorobenzene and Bromobenzene from 293K to 313K. Int. J. Thermophys. 2008, 29, 1376–1384. https://doi.org/10.1007/s10765-008-0444-7.

(382)	Ni, B.; Su, L.; Wang, H.; Qiu, H. Thermophysical Properties of the Binary Mixtures of 1,2-Dichloroethane with Chlorobenzene and Bromobenzene from 298.15 to 313.15 K. J. Chem. Eng. Data 2010, 55, 4541–4545. https://doi.org/10.1021/je100552a.

(383)	Hamzehlouia, S.; Asfour, A. A. Densities and Viscosities of Ten Binary and Ten Ternary Regular Solution Systems at 308.15 and 313.15 K. J. Mol. Liq. 2012, 174, 143–152. https://doi.org/10.1016/j.molliq.2012.06.020.

(384)	Kumar, D. B. K.; Reddy, K. R.; Rao, G. S.; Rao, G. V. R.; Rambabu, C. Thermodynamic and Spectroscopic Study of Molecular Interactions in the Binary Liquid Mixtures of N-Methyl-2-Pyrrolidone and Some Substituted Benzenes at Different Temperatures. J. Mol. Liq. 2012, 174, 100–111. https://doi.org/10.1016/j.molliq.2012.07.019.

(385)	Rathnam, M. V.; Bhanushali, K. R.; Sayed, R. T.; Kumar, M. S. S. Acoustic, Thermodynamic and Transport Properties of Binary Liquid Mixtures of Isopentyl Acetate with Aryl Halides at 303.15 and 313.15 K. J. Mol. Liq. 2012, 173, 35–41. https://doi.org/10.1016/j.molliq.2012.05.022.

(386)	Lavanya, T. G.; Saravanakumar, K.; Baskaran, R.; Kubendran, T. R. Density, Viscosity, Sound Speed, and Thermoacoustical Parameters of Benzaldehyde with Chlorobenzene or Nitrobenzene at 303.15 K, 308.15 K, and 313.15 K. Int. J. Thermophys. 2013, 34, 1280–1287. https://doi.org/10.1007/s10765-013-1492-1.

(387)	Panchenkov, G. M.; Maksareva, T. S.; Erchenkov, V. V. ? Zh. Fiz. Khim. 1958, 32, 2787–2791.

(388)	Akhundov, T. S.; Abdullaev, F. G.; Ishkhanov, Yu. B. Experimental Study of the Viscosity of Chlorobenzene in the Gaseous Phase and Critical Region. Izv. Vyssh. Uchebn. Zaved., Neft Gaz 1984, 86, 93.

(389)	Vogel, E. Towards Reference Viscosities of Carbon Monoxide and Nitrogen at Low Density Using Measurements between 290K and 680K as Well as Theoretically Calculated Viscosities. Int. J. Thermophys. 2012, 33, 741–757. https://doi.org/10.1007/s10765-012-1185-1.

(390)	Rabe, D. Experimental Determination of Dynamic Viscosity and Density of Binary Liquid Mixtures of N-Heptane with CO2, CH4, C2H6, C3H8 and of Toluene with CH4 and C3H8. PhD Thesis, Technical University Berlin, 1981.

(391)	Diller, D. E.; Ball, M. J. Shear Viscosity Coefficients of Compressed Gaseous and Liquid Carbon Dioxide at Temperatures between 220 and 320 K and at Pressures to 30 MPa. Int. J. Thermophys. 1985, 6, 619. https://doi.org/10.1007/bf00500334.

(392)	Gururaja, G. J.; Tirunarayanan, M. A.; Ramachandran, A. Dynamic Viscosity of Gas Mixtures. J. Chem. Eng. Data 1967, 12, 562–567. https://doi.org/10.1021/je60035a024.

(393)	Kestin, J.; Khalifa, H. E.; Wakeham, W. A. Viscosity of Multicomponent Mixtures of Four Complex Gases. J. Chem. Phys. 1976, 65, 5186–5188.

(394)	Harris, E. J. R.; Hope, C. G.; Gough, D. W.; Smith, E. B. Viscosity of Gaseous Carbon Dioxide, Sulfur Hexafluoride, and Nitrous Oxide at Low Temperatures. J. Chem. Soc., Faraday Trans. 1 1979, 75, 892–897.

(395)	Lusternik, V. E.; Kuznetsov, V. I. Measurements of the Viscosity of CO2 up to 1870 Kat 1 Atm. Teplofiz. Vys. Temp. 1986, 24, 169–170.

(396)	Hunter, I. N.; Marsh, G.; Matthews, G. P.; Smith, E. B. Argon + Carbon Dioxide Gaseous Mixture Viscosities and Anisotropic Pair Potential Energy Functions. Int. J. Thermophys. 1993, 14, 819–833. https://doi.org/10.1007/bf00502110.

(397)	Ulybin, S. A.; Makarushkin, V. I. Viscosity of CO2 at 220-1300 k up to 300 MPa. Teploenergetika 1976, 23, 65.

(398)	Padua, A.; Wakeham, W. A.; Wilhelm, J. The Viscosity of Liquid Carbon Dioxide. Int. J. Thermophys. 1994, 15, 767–777. https://doi.org/10.1007/bf01447093.

(399)	Maltsev, V. A.; Nerushev, O. A.; Novopashin, S. A.; Radchenko, V. V.; Licht, W.; Miller, E. J.; Parekh, V. S. Viscosity of H2-CO2 Mixtures at (500, 800, and 1100) K. J. Chem. Eng. Data 2004, 49, 684–687. https://doi.org/10.1021/je0342419.

(400)	Isdale, J. D.; Dymond, J. H.; Brawn, T. A. Viscosity and Density of N-Hexane-Cyclohexane Mixtures between 25 and 100 C up to 500 MPa. High Temp. - High Pressures 1979, 11, 571–580.

(401)	Lavrent’ev, I. P.; Yakovlev, V. F. The Velocity of Ultrasound in Cyclohexane and Its Shear Viscosity along the Saturation Line. Zh. Fiz. Khim. 1985, 59, 2893.

(402)	Drapier, P. Research on the Viscosity of Binary Liquid Mixtures in the Vicinity of the Critical Temperature. Bull. Cl. Sci. Acad. R. Belg. 1911, 1, 621–640.

(403)	Vorlander, D.; Fischer, J. Chem. Ber. 1932, 65, 1756.

(404)	Pownall, H. J.; Smith, L. C. Viscosity of the Hydrocarbon Region of Micelles. Measurement of Excimer Fluorescence. J. Am. Chem. Soc. 1973, 95, 3136–3140. https://doi.org/10.1021/ja00791a009.

(405)	Jonas, J.; Hasha, D.; Huang, S. G. Density Effects of Transport Properties in Liquid Cyclohexane. J. Phys. Chem. 1980, 84, 109.

(406)	Mishra, R. Interaction Studies in Binary Liquid Mixtures from Viscosity Measurements. Acta Chim. Acad. Sci. Hung. 1981, 108, 103–110.

(407)	Durov, V. A.; Artykov, A. Acoustic Spectroscope of Liquid Cyclohexane. Zh. Fiz. Khim. 1985, 59, 2550–2554.

(408)	Manjeshwar, L. S.; Aminabhavi, T. M. Densities and Viscosities of Binary Liquid Mixtures Containing Bromoform at 45 Deg C. J. Chem. Eng. Data 1988, 33, 184–185. https://doi.org/10.1021/je00052a036.

(409)	Cueto, M. J. M.; Vallejo, M. G.; Luque, V. F. Physiochemical Studies Concerning Miscibility of Vegetable Oils. XIV. Molar Volumes, Molar Refractions, and Viscosities of Solutions of Methyl Stearate in Cyclohexane, Hexane, Tetrachloroethylene, and Trichloroethylene. Grasas Aceites 1991, 42, 14–21.

(410)	Papanastasiou, G. E.; Ziogas, I. I. Physical Behavior of Some Reaction Media. Density, Viscosity, Dielectric Constant, and Refractive Index Changes of Ethanol-Cyclohexane Mixtures at Several Temperatures. J. Chem. Eng. Data 1991, 36, 46–51. https://doi.org/10.1021/je00001a014.

(411)	Nath, J.; Chaudhari, S. K. Excess Volumes, Dielectric Constants, Refractive Indexes, and Viscosities for Anisole + Methylene Chloride, 1,2-Dichloroethane, Trichloroethene, Tetrachloroethene, and Cyclohexane. J. Chem. Eng. Data 1992, 37, 387–390. https://doi.org/10.1021/je00008a003.

(412)	Blasco, D.; Lafuente, C.; Santafe, J.; Royo, F. M.; Urieta, J. S. Excess Volumes and Excess Viscosities of Binary Mixtures of Cyclohexane + Picoline. Thermochim. Acta 1993, 230, 55–63. https://doi.org/10.1016/0040-6031(93)80346-c.

(413)	Munoz, M. J.; Rodriguez, M.; Martin, R.; Galan, M. A Study of the Viscosity of Methyl Myristate Solutions in Organic Solvents. An. Quim. 1994, 90, 177–180.

(414)	Pandey, J. D.; Jain, P.; Vyas, V. Speed of Sound, Viscosity, and Refractive Index of Multicomponent Systems: Analysis of Experimental Data from the Bertrand-Acree- Burchfield Equation. Can. J. Chem. 1994, 72, 2486–2492. https://doi.org/10.1139/v94-315.

(415)	Aminabhavi, T. M.; Gopalakrishna, B. Density, Viscosity, Refractive Index, and Speed of Sound in Binary Mixtures of 2-Ethoxyethanol with n-Alkanes (C6 to C12), 2,2,4- Trimethylpentane, and Cyclohexane in the Temperature Interval 298.15-. J. Chem. Eng. Data 1995, 40, 632–641. https://doi.org/10.1021/je00019a022.

(416)	Artigas, H.; Sanz, M. L.; Mainar, A. M.; Royo, F. M.; Urieta, J. S. EXCESS VOLUMES AND EXCESS VISCOSITIES OF BINARY MIXTURES OF CYCLOHEXANE + AN ISOMER OF BUTANOL AT SEVERAL TEMPERATURES. Phys. Chem. Liq. 1995, 30, 17–28.

(417)	Aminabhavi, T. M.; Patil, V. B.; Aralaguppi, M. I.; Phayde, H. T. S. Density, Viscosity, and Refractive Index of the Binary Mixtures of Cyclohexane with Hexane, Heptane, Octane, Nonane, and Decane at (298.15, 30 3.15, and 308.15) K. J. Chem. Eng. Data 1996, 41, 521–525. https://doi.org/10.1021/je950279c.

(418)	Padua, A. A. H.; Fareleira, J. M. N. A.; Calado, J. C. G.; Wakeham, W. A. Validation of an Accurate Vibrating-Wire Densimeter: Density and Viscosity of Liquids over Wide Ranges of Temperature and Pressure. Int. J. Thermophys. 1996, 17, 781–802. https://doi.org/10.1007/bf01439190.

(419)	Aminabhavi, T. M.; Banerjee, K. Density, Viscosity, Refractive Index, and Speed of Sound in Binary Mixtures of Methyl Acetate + Ethylene Glycol or + Poly(Ethylene Glycol) in the Temperature Interval (298.15-308.15) K. J. Chem. Eng. Data 1998, 43, 852–855. https://doi.org/10.1021/je9800757.

(420)	Gascon, I.; Lafuente, C.; Cea, P.; Royo, F. M.; Urieta, J. S. Viscosities of the Ternary Mixture (Cyclohexane + Tetrahydrofuran + Chiorocyclohexane) at 298.15 and 313.15 K. Fluid Phase Equilib. 1999, 164, 143–155. https://doi.org/10.1016/s0378-3812(99)00257-5.

(421)	Gascon, I.; Lopez, M. C.; Dominguez, M. M.; Royo, F. M.; Urieta, J. S. Viscosities and Viscosity Predictions of the Ternary Mixture Cyclohexane + 1-3-Dioxolane + 1-Butanol at 298.15 and 313.15 K. J. Chem. Eng. Jpn. 2000, 33, 740–746.

(422)	Gascon, I.; Mainar, A. M.; Royo, F. M.; Urieta, J. S.; Alvarez-Cerdeirina, L. Experimental Viscosities and Viscosity Predictions of the Ternary Mixture (Cyclohexane + 1,3-Dioxolane + 2-Butanol) at 298.15 and 313.15 K. J. Chem. Eng. Data 2000, 45, 751–755. https://doi.org/10.1021/je000081e.

(423)	Gomez-Diaz, D.; Mejuto, J. C.; Navaza, J. M. Physicochemical Properties of Liquid Mixtures. 1. Viscosity, Density, Surface Tension and Refractive Index of Cyclohexane + 2,2,4-Trimethylpentane Binary Liquid Systems from 25 C to 50 C. J. Chem. Eng. Data 2001, 46, 720–724. https://doi.org/10.1021/je000310x.

(424)	Gomez-Diaz, D.; Mejuto, J. C.; Navaza, J. M.; Rodriguez-Alvarez, A. Viscosities, Densities, Surface Tensions, and Refractive Indexes of 2,2,4-Trimethylpentane + Cyclohexane + Decane Ternary Liquid Systems at 298.15 K. J. Chem. Eng. Data 2002, 47, 872–875. https://doi.org/10.1021/je010288n.

(425)	Ali, A.; Abida; Hyder, S. STUDY OF INTERMOLECULAR INTERACTIONS IN BINARY MIXTURES OF CYCLOHEXANE WITH 1-ALKANOLS AT 308K. Phys. Chem. Liq. 2004, 42, 411–422.

(426)	Haro, M.; Rodriguez, V.; Cea, P.; Lopez, M. C.; Lafuente, C. Viscosimetric Study of Multicomponent Liquid Mixtures Containing Oxygenated Compounds. Int. J. Thermophys. 2004, 25, 669–678. https://doi.org/10.1023/b:ijot.0000034231.98922.7e.

(427)	Oswal, S. L.; Ghael, N. Y.; Prajapati, K. D. Speeds of Sound, Isentropic Compressibilities, Viscosities and Excessmolar Volumes of Cyclohexanol + Cyclohexane at 303.15 K. Thermochim. Acta 2004, 419, 59–65. https://doi.org/10.1016/j.tca.2004.01.034.

(428)	Iloukhani, H.; Rezaei-Sameti, M.; Zarei, H. A. Volumetric and Viscometric Studies of Molecular Interaction of the Ternary System Toluene (1) + Cyclohexane (2) + n-Hexane (3) at 298.15K. Thermochim. Acta 2005, 438, 9–15. https://doi.org/10.1016/j.tca.2005.08.007.

(429)	Singh, S.; Sethi, B. P. S.; Katyal, R. C.; Rattan, V. K. Viscosities, Densities, and Speeds of Sound of Binary Mixtures of o-Xylene, m-Xylene, p-Xylene, and Isopropylbenzene with 2-Butanone at 298.15 K. J. Chem. Eng. Data 2005, 50, 125–127. https://doi.org/10.1021/je049793l.

(430)	Gonzalez, E. J.; Gonzales, B.; Calvar, N.; Dominguez, A. Physical Properties of Binary Mixtures of the Ionic Liquid 1-Ethyl-3-Methylimidazolium Ethyl Sulfate with Several Alcohols at T = (298.15, 313.15, and 328.15) K and Atmospheric Pressure. J. Chem. Eng. Data 2007, 52, 1641–1648. https://doi.org/10.1021/je700029q.

(431)	Fan, W.; Zhou, Q.; Zhang, S.; Yan, R. Excess Molar Volume and Viscosity Deviation for the Methanol + Methyl Methacrylate Binary System at T) (283.15 to 333.15) K. J. Chem. Eng. Data 2008, 53, 1836–1840. https://doi.org/10.1021/je800164w.

(432)	Gardas, R. L.; Oswal, S. L. Volumetric and Transport Properties of Ternary Mixtures Containing 1-Butanol or 1-Pentanol, Triethylamine and Cyclohexane at 303.15 K: Experimental Data, Correlation and Prediction by the ERAS Model. J. Solution Chem. 2008, 37, 1449–1470.

(433)	Ghael, N. Y.; Gardas, R. L.; Oswal, S. L. Volumetric and Transport Properties of Ternary Mixtures Containing 1-Alkanol + Ethyl Ethanoate + Cyclohexane at 303.15 K: Experimental Data, Correlation and Prediction by ERAS Model. Thermochim. Acta 2009, 491, 44–57. https://doi.org/10.1016/j.tca.2009.02.022.

(434)	Hernandez-Galvan, M. A.; Garcia-Sanchez, F.; Garcia-Flores, B. E.; Castro-Arellano, J. Liquid Viscosities of Cyclohexane, Cyclohexane + Tetradecane, and Cyclohexane + Benzene from (313 to 393) K and Pressures Up to 60 MPa. J. Chem. Eng. Data 2009, 54, 2831–2838. https://doi.org/10.1021/je800882f.

(435)	Roy, M. N.; Sarkar, B. K.; Sinha, B. Densities and Viscosities of Ternary Mixtures of Cyclohexane + Cyclohexanone + Some Alkyl Acetates at 298.15 K. J. Chem. Eng. Data 2009, 54, 1076–1083. https://doi.org/10.1021/je8007217.

(436)	Silva, A.; Reis, R. A.; Paredes, M. L. L. Density and Viscosity of Decalin, Cyclohexane, and Toluene Binary Mixtures at (283.15, 293.15, 303.15, 313.15, and 323.15) K. J. Chem. Eng. Data 2009, 54, 2067–2072. https://doi.org/10.1021/je800940j.

(437)	Rathnam, M. V.; Mankumare, S.; Kumar, M. S. S. Density, Viscosity, and Speed of Sound of (Methyl Benzoate + Cyclohexane), (Methyl Benzoate + n-Hexane), (Methyl Benzoate + Heptane), and (Methyl Benzoate + Octane) at Temperatures of (303.15, 308.15, and 313.15) K. J. Chem. Eng. Data 2010, 55, 1354–1358. https://doi.org/10.1021/je9006597.

(438)	Yadava, S. S.; Yadav, N. Densities, Viscosities and Ultrasound Velocities for Binary Mixtures of Isopropylethanoate and Non-Polar Hydrocarbon Solvents. J. Mol. Liq. 2010, 157, 6–12. https://doi.org/10.1016/j.molliq.2010.07.010.

(439)	Rathnam, M. V.; Mankumare, S.; Jain, K.; Kumar, M. S. S. Densities, Viscosities and Speeds of Sound of Binary Mixtures of Ethyl Benzoate + Hydrocarbons at (303.15, 308.15 and 313.15) K. J. Solution Chem. 2012, 41, 475–490. https://doi.org/10.1007/s10953-012-9802-y.

(440)	Sastry, N. V.; Patel, S. R.; Soni, S. S. Excess Molar Volumes, Excess Isentropic Compressibilities, Excess Viscosities, Relative Permittivity and Molar Polarization Deviations for Methyl Acetate+, Ethyl Acetate+, Butyl Acetate+, Isoamyl Acetate+, Methyl Propionate+, Ethyl Propionate+, Ethyl Butyrate+, Methyl Methacrylate+, Ethyl Methacrylate+, and Butyl Methacrylate + Cyclohexane at T = 298.15 and 303.15 K. J. Mol. Liq. 2013, 183, 102–112. https://doi.org/10.1016/j.molliq.2013.04.015.

(441)	Hammami, N. E.; Bouanz, M.; Toumi, A. Thermodynamic Properties of Cyclohexane Methanol Liquid Mixture from Shear Viscosity Measurements. Fluid Phase Equilib. 2014, 384, 25–35. https://doi.org/10.1016/j.fluid.2014.10.013.

(442)	Torin-Ollarves, G. A.; Martin, M. C.; Chamorro, C. R.; Segovia, J. J. Densities, Viscosities, and Isobaric Heat Capacities of the System (1-Butanol + Cyclohexane) at High Pressures. J. Chem. Thermodyn. 2014, 74, 153–160. https://doi.org/10.1016/j.jct.2014.01.020.

(443)	Ma, R.-F.; Shi, L.; Duan, Y.-Y.; Han, L.-Z.; Liu, N.-X. Saturated Liquid Viscosity of Cyclopentane and Isopentane. J. Chem. Eng. Data 2003, 48, 1418–1421. https://doi.org/10.1021/je0202174.

(444)	Timmermans, J.; Hennaut-Roland, M. Works from International Bureau at Physical-Chemical Standards. VIII. Physical Constants of 20 Organic Compounds. J. Chim. Phys. Phys.-Chim. Biol. 1937, 34, 693.

(445)	Assael, M. J.; Dalouti, N. K.; Dymond, J. H. The Viscosity of Toluene in the Temperature Range 210 to 370 K. Int. J. Thermophys. 2000, 21, 291. https://doi.org/10.1023/a:1006628008963.

(446)	Harris, K. R.; Newitt, P. J.; Woolf, L. A. Temperature and Density Dependence of the Viscosity of Cyclopentane. J. Chem. Eng. Data 2004, 49, 138–142. https://doi.org/10.1021/je034142v.

(447)	Gonzalez, B.; Dominguez, D.; Tojo, J. Physical Properties of the Binary Systems Methylcyclopentane with Ketones (Acetone, Butanone and 2-Pentanone) at T = (293.15, 298.15, and 303.15) K. New UNIFAC-VISCO Interaction Parameters. J. Chem. Thermodyn. 2006, 38, 707–716. https://doi.org/10.1016/j.jct.2005.08.002.

(448)	Kurihara, K.; Kandil, M. E.; Marsh, K. N.; Goodwin, A. R. H. Measurement of the Viscosity of Liquid Cyclopentane Obtained with a Vibrating Wire Viscometer at Temperatures between (273 and 353) K and Pressures below 45 MPa. J. Chem. Eng. Data 2007, 52, 803–807. https://doi.org/10.1021/je060416d.

(449)	Kumar, H.; Singla, M.; khosla, A.; Gaba, R. Viscometric Studies of Binary Liquid Mixtures of Cyclopentane (1) +2-Propanol (2), +1-Butanol (2), and +2-Butanol (2) at T=(298.15 and 308.15) K. J. Mol. Liq. 2011, 158, 182–186. https://doi.org/10.1016/j.molliq.2010.12.002.

(450)	Kumar, H.; Gulati, N.; Kundi, V. Viscometric Studies of Binary Liquid Mixtures of Cyclopentane (1)+branched Alkanols (2) at T=(298.15 and 308.15) K. J. Mol. Liq. 2012, 174, 63–69. https://doi.org/10.1016/j.molliq.2012.07.020.

(451)	Dunlop, P. J. Viscosities of a Series of Gaseous Fluorocarbons at 25 .Degree.C. J. Chem. Phys. 1994, 100, 3149–3151. https://doi.org/10.1063/1.466405.

(452)	Lukin, V. I.; Ivakin, B. A.; Suetin, P. E. Temperature Dependence of the Viscosity Coefficients of Some Gases. Sov. Phys. - Tech. Phys. 1983, 28, 597.

(453)	Van Itterbeek, A.; Paemel, van. Measurements on the Viscosity of Neon, Hydrogen, Deuterium and Helium as a Function of the Temperature, between Room Temperature and Liquid Hydrogen Temperatures. Physica (Amsterdam) 1940, 7, 265.

(454)	Coremans, J. M. J.; van Itterbeek, A.; Beenakker, J. J. M.; Knaap, H. F. P.; Zandbergen, P. The Viscosity of Gaseous He, Ne, H2 and D2 below 80 K. Physica Amsterdam 1958, 24, 557–576. https://doi.org/10.1016/S0031-8914(58)80070-1.

(455)	Rudenko, N. S.; Konareva, V. G. Viscosity of Liquid Hydrogen and Deuterium. Russ. J. Phys. Chem. 1963, 37, 1493–1494.

(456)	Becker, E. N.; Misenta, R. Viscosity of HD and Helium-3 between 14 and 20 K. Z. Phys. 1955, 140, 535–539. https://doi.org/10.1007/BF01337559.

(457)	Van Itterbeek, A.; Claes, A. Viscosity of Light Hydrogen Gas and Deuterium between 293 Degrees K. and 14 Degrees K. Nature (London) 1938, 142(3600), 793–794.

(458)	Michels, A.; Schipper, A. C. J.; Rintoul, W. H. The Viscosity of Hydrogen and Deuterium at Pressures up to 2000 Atmospheres. Physica (Amsterdam) 1953, 19, 1011–1028.

(459)	Menabde, N. E. Viscosity Coefficient of Hydrogen (H(2), D(2)), Neon (Ne(20), Ne(22)) and Helium (He(3)) Isotopes in the Temperature Range -195 to +25 Degrees C. Sov. At. Energy (Engl. Transl.) 1965, 19, 1421–1422.

(460)	Van Itterbeek, A.; Van Paemel, O. Determination of Internal Friction of Liquid Hydrogen and Deuterium. Physica (Amsterdam) 1941, 8, 133–143.

(461)	Rietveld, A., A. O. ,. van Itterbeek; Velds, C. A. Viscosity of Binary Mixtures of Hydrogen Isotopes and Mixtures of He and Ne. Comm. Phys. Lab. Univ. Leiden 1959, 1–12.

(462)	Barua, A. K.; Afzal, M.; Flynn, G. P.; Ross, J. Viscosity of Hydrogen, Deuterium, Methane, and Carbon Monoxide from 50 to 150 C below 200 Atmospheres. J. Chem. Phys. 1964, 41, 374.

(463)	Lukin, V. I.; Ivakin, B. A.; Suetin, P. E. Viscosity of Some Gases Measured by the Non-Steady Flow Method. Zh. Tekhn. Fiz. 1981, 51, 860–862.

(464)	Lukin, V. I.; Ivakin, B. A.; Suetin, P. E. Temperature Dependence of Then Viscosity Coefficients of Some Gases. Zh. Tekhn. Fiz. 1983, 53, 931–932.

(465)	Konareva, V. G.; Rudenko, N. S. Measurements of Viscosity of the Hydrogen Isotops along the Liquid-Gas Coexistence Curve. Zh. Fiz. Khim. 1967, 41, 2387–2388.

(466)	Rudenko, N. P.; Slyusar, V. P. Density of Liquid Deiterium. Zh. Fiz. Khim. 1968, 42, 242–243.

(467)	Kestin, J.; Leidenfrost, W. Effect of Pressure on the Viscosity of N2-CO2 Mixtures. Physica (Amsterdam) 1959, 25, 525–536.

(468)	Rudenko, N. S.; Konareva, V. G. Viscosity of Liquid PH2 and OH2. Zh. Eksp. Teor. Fiz. 1965, 1965, 769–770.

(469)	De Troyer, A.; Van Itterbeek, A.; Van den Berg, G. J. Measurements on the Thermal Diffusion of Ne - H2, Ne - D2 and Ne - He Mixtures at Liquid Hydrogen Temperatures. Physica Amsterdam 1950, 16, 669–677. https://doi.org/10.1016/0031-8914(50)90035-8.

(470)	Kestin, J.; Nagashima, A. Viscosity of Neon-Helium and Neon-Argon Mixtures at 20 and 30 C. J. Chem. Phys. 1964, 40, 3648–3654.

(471)	Maitland, G. C.; Mustapha, M.; Wakeham, W. A. Thermal Conductivity of Polyatomic Gases at Low Density. J. Chem. Soc. Faraday Trans. I 1983, 79, 163–172. https://doi.org/10.1039/f19837900163.

(472)	Rivkin, S. L.; Levin, A. Ya.; Izrailevskii, L. B.; Kharitonov, K. G. Experimental Study of Viscosity of the Heavy Water in Liquid Phase at Pressures up to 500 Bar. Teploenergetika 1974, 69–71.

(473)	Kellomaki, A. Finn. Chem. Lett. 1975, 2, 51.

(474)	Selwood, P. W.; Frost, A. A. Some Properties of Heavy Water. J. Am. Chem. Soc. 1933, 55, 4335–4336. https://doi.org/10.1021/ja01337a508.

(475)	Taylor, H. S.; Selwood, P. W. Some Properties of Heavy Water. J. Am. Chem. Soc. 1934, 56, 998–999. https://doi.org/10.1021/ja01319a509.

(476)	Timrot, D. L.; Shuiskaya, K. F. Experimental Study of the Viscosity of Heavy Water. At. Energ. 1959, 7, 459–462.

(477)	Tourky, A. R.; Rizk, H. A.; Girgis, Y. M. The Dielectric Properties of Heavy Water in Dioxane. Z. Phys. Chem. (Munich) 1962, 32, 102–109.

(478)	Agaev, N. A.; Usibova, A. D. Viscosity of Ordinary and Heavy Water at High Pressures in the Temperature Range from 0 to 150 C. Dokl. Akad. Nauk Az. SSR 1968, 180, 334–337.

(479)	Rivkin, S. L.; Levin, A. Ya.; Izrailevskii, L. B.; Kharitonov, K. G.; Gromova, I. A. Dependence between the Dynamic Viscosity Coefficient of Heavy and Ordinary Water in the Liquid Phase. Teploenergetika 1976, 79–82.

(480)	Timrot, D. L.; Serednitskaya, M. A.; Traktueva, C. A. Viscosity of Heavy Water Vapor at 100-500.Deg. and 0.08-1.3 Bars. Teploenergetika 1974, 83–84.

(481)	Rivkin, S. L.; Levin, A. Ya.; Izrailevskii, L. B.; Kharitonov, K. G.; Ptitsyna, V. N. Experimental Study of the Dynamic Viscosity Coefficient of Heavy Water at Supercrtical Parameters of State. Teploenergetika 1977, 65–67.

(482)	Dickinson, E. Pressure Dependence of Shear Viscosity in N-Alkane + Dimethylsiloxane Mixtures. J. Phys. Chem. 1977, 81, 2108–2113.

(483)	Abbas, R.; Ihmels, E. C.; Enders, S.; Gmehling, J. Measurement of Transport Properties for Selected Siloxanes and Their Mixtures Used as Working Fluids for Organic Rankine Cycles. Ind. Eng. Chem. Res. 2011, 50, 8756–8763. https://doi.org/10.1021/ie2002632.

(484)	Fischer, J. D. Transport Properties of Pure Fluids and Binary Mixtures with Different Interaction Parameters. Thesis 1986, 1–290.

(485)	Markgraf, J. H. 1-Chloro-2,3-Dimethylbutane. J. Chem. Eng. Data 1968, 13, 447–448. https://doi.org/10.1021/je60038a047.

(486)	Fischer, J.; Weiss, A. Transport Properties of Liquids. VII. Viscosity, Excess Volumes, and Self-Diffusion of Binary Mixtures of Donor-Acceptor and Carbon Tetrachloride-Carboxylic Acid Systems. Ber. Bunsen-Ges. Phys. Chem. 1986, 90, 1141. https://doi.org/10.1002/bbpc.19860901205.

(487)	Palczewska-Tulinska, M.; Oracz, P. Selected Physicochemical Properties of Hexamethylcyclotrisiloxane, Octamethylcyclotetrasiloxane, and Decamethylcyclopentasiloxane. J. Chem. Eng. Data 2005, 50, 1711–1719. https://doi.org/10.1021/je050173+.

(488)	Whorton, R.; Amis, E. S. The Equivalent Conductance of Electrolytes in Mixed Solvents: IV Tetraethylammonium Picrate in the Methanol + Ethanol System. Z. Phys. Chem. (Munich) 1956, 8, 9–19.

(489)	DiGuilio, R. M.; Lee, R. J.; Schaeffer, S. T.; Brasher, L. L.; Teja, A. S. Densities and Viscosities of the Ethanolamines. J. Chem. Eng. Data 1992, 37, 239–242. https://doi.org/10.1021/je00006a028.

(490)	Teng, T. T.; Maham, Y.; Hepler, L. G.; Mather, A. E. Viscosity of Aqueous Solutions of N-Methyldiethanolamine and of Diethanolamine. J. Chem. Eng. Data 1994, 39, 290–293. https://doi.org/10.1021/je00014a021.

(491)	Blanco, A.; Garcia-Abuin, A.; Gomez-Diaz, D.; Navaza, J.; Villaverde, O. Density, Speed of Sound, Viscosity, Surface Tension, and Excess Volume of N-Ethyl-2-Pyrrolidone + Ethanolamine (or Diethanolamine or Triethanolamine) from T = (293.15 to 323.15) K. J. Chem. Eng. Data 2013, 58, 653–659. https://doi.org/10.1021/je301123j.

(492)	Lopez, A. B.; Garcia-Abuin, A.; Gomez-Diaz, D.; La Rubia, M. D.; Navaza, J. M. Density, Speed of Sound, Viscosity, Refractive Index and Surface Tension of N-Methyl-2-Pyrrolidone + Diethanolamine (or Triethanolamine) from T = (293.15 to 323.15) K. J. Chem. Thermodyn. 2013, 61, 1–6. https://doi.org/10.1016/j.jct.2013.01.020.

(493)	Spasojevic, V. D.; Serbanovic, S. P.; Djordjevic, B. D.; Kijeveanin, M. L. Densities, Viscosities, and Refractive Indices of Aqueous Alkanolamine Solutions as Potential Carbon Dioxide Removal Reagents. J. Chem. Eng. Data 2013, 58, 84–92. https://doi.org/10.1021/je300938w.

(494)	Haghtalab, A.; Shojaeian, A. Volumetric and Viscometric Behaviour of the Binary Systems of N-Methyldiethanolamine and Diethanolamine with 1-Butyl-3-Methylimidazolium Acetate at Various Temperatures. J. Chem. Thermodyn. 2014, 68, 128–137. https://doi.org/10.1016/j.jct.2013.09.001.

(495)	Sage, B. H.; Lavender, H. M.; Lacey, W. N. Phase Equilibria in Hydrocarbon Systems: Methane - Decane System. Ind. Eng. Chem. 1940, 32, 743–747.

(496)	Lee, A. L.; Ellington, R. T. Viscosity of N-Decane in the Liquid Phase. J. Chem. Eng. Data 1965, 10, 346–348. https://doi.org/10.1021/je60027a013.

(497)	Carmichael, L. T.; Berry, V. M.; Sage, B. H. Viscosity of Hydrocarbons. n-Decane. J. Chem. Eng. Data 1969, 14, 27–31. https://doi.org/10.1021/je60040a009.

(498)	Moore, J. W.; Wellek, R. M. Diffusion Coefficients of N-Heptane and n-Decane in n-Alkanes and n-Alcohols at Several Temperatures. J. Chem. Eng. Data 1974, 19, 136–140. https://doi.org/10.1021/je60061a023.

(499)	Atrops, H.; Kalali, H. E.; Kohler, F. Melting Curves, Densities, Viscosities and Thermodymamic Excess Properties of 1,2,4-Trichlorobenzene + n-Decane and + n-Tetradecane. Ber. Bunsen-Ges. Phys. Chem. 1982, 86, 26–31. https://doi.org/10.1002/bbpc.19820860106.

(500)	Wakefield, D. L.; Marsh, K. N.; Zwolinski, B. J. Viscosities of Nonelectrolyte Liquid Mixtures. II. Binary and Quaternary Systems of Some n-Alkanes. Int. J. Thermophys. 1988, 9, 47. https://doi.org/10.1007/bf00503999.

(501)	Assael, M. J.; Dymond, J. H.; Papadaki, M.; Patterson, P. M. Correlation and Prediction of Dense Fluid Transport Coefficients. I. n-Alkanes. Int. J. Thermophys. 1992, 13 (2), 269–281.

(502)	Oliveira, C. M. B. P.; Wakeham, W. A. The Viscosity of Five Liquid Hydrocarbons at Pressures up to 250 MPa. Int. J. Thermophys. 1992, 13, 773–790. https://doi.org/10.1007/bf00503906.

(503)	Papaioannou, D.; Bridakis, M.; Panayiotou, C. G. Excess Dynamic Viscosity and Excess Volume of N-Butylamine + 1-Alkanol Mixtures at Moderately High Pressures. J. Chem. Eng. Data 1993, 38, 370–378. https://doi.org/10.1021/je00011a010.

(504)	Estrada-Baltazar, A.; Alvarado, J. F. J.; Iglesias-Silva, G. A.; Barrufet, M. A. Experimental Liquid Viscosities of Decane and Octane + Decane from 298.15 K to 373.15 K and Up to 25 MPa. J. Chem. Eng. Data 1998, 43, 441–446. https://doi.org/10.1021/je970233e.

(505)	Franjo, C.; Segade, L.; Menaut, C. P.; Pico, J. M.; Jimenez, E. Viscosities and Densities of Solutions of N-Decane, or n-Tetradecane with Several Esters at 25 C. J. Solution Chem. 2001, 30, 995–1005.

(506)	Segade, L.; Casad, H.; Cabeza, O.; Prego, M.; Garcia-Garabal, S.; Franjo, C.; Jimenez Cuesta, E. Viscosities of Di-n-Butyl Ether + 1-Propanol + n-Decane Mixture at 308.15 K. Fluid Phase Equilib. 2001, 182, 353–364. https://doi.org/10.1016/s0378-3812(01)00393-4.

(507)	Tohidi, B.; Burgass, R. W.; Danesh, A.; Todd, A. C. Viscosity and Density of Methane + Methylcyclohexane from (323 to 423) K and Pressures to 140 MPa. J. Chem. Eng. Data 2001, 46, 385–390. https://doi.org/10.1021/je000191l.

(508)	Audonnet, F.; Padua, A. A. H. Viscosity and Density of Mixtures of Methane and N-Decane from 298 to 393 K and up to 75 MPa. Fluid Phase Equilib. 2004, 216, 235–244. https://doi.org/10.1016/j.fluid.2003.10.017.

(509)	Kapoor, S.; Rattan, V. K. Isobaric Vapor-Liquid Equilibria at 97.3 KPa and Excess Properties at (303.15, 308.15, and 313.15) K of Binary Mixture of p-Xylene + Decane. J. Chem. Eng. Data 2005, 50, 1891–1896. https://doi.org/10.1021/je0501585.

(510)	Dubey, G. P.; Sharma, M. Thermophysical Properties of Binary Mixtures of 2-Methyl-1-Propanol with Hexane, Octane, and Decane at 298.15 K. J. Chem. Eng. Data 2007, 52, 449–453. https://doi.org/10.1021/je060389r.

(511)	Fang, Z.; Qiao, Y.; Di, Z.; Huo, Y.; Ma, P.; Xia, S. Viscosities of P-Xylene, Acetic Acid, and p-Xylene + Acetic Acid at (313.15 to 473.15) K and (0.10 to 3.20) MPa: Determined by the Rolling-Ball Method. J. Chem. Eng. Data 2008, 53, 2787–2792. https://doi.org/10.1021/je800635g.

(512)	Yang, F.; Guo, Y.; Xing, Y.; Li, D.; Fang, W.; Lin, R. Densities and Viscosities of Binary Mixtures of JP-10 with n-Octane or n-Decane at Several Temperatures. J. Chem. Eng. Data 2008, 53, 2237–2240. https://doi.org/10.1021/je800348s.

(513)	Caudwell, D. R.; Trusler, J. P. M.; Vesovic, V.; Wakeham, W. A. Viscosity and Density of Five Hydrocarbon Liquids at Pressures up to 200 MPa and Temperatures up to 473 K. J. Chem. Eng. Data 2009, 54, 359–366. https://doi.org/10.1021/je800417q.

(514)	Feitosa, F. X.; Caetano, A. C. R.; Cidade, T. B.; de Sant Ana, H. B. Viscosity and Density of Binary Mixtures of Ethyl Alcohol with N-Alkanes (C6, C8, and C10). J. Chem. Eng. Data 2009, 54, 2957–2963. https://doi.org/10.1021/je800925v.

(515)	Sato, Y.; Yoshioka, H.; Aikawa, S.; Smith Jr., R. L. A Digital Variable-Angle Rolling-Ball Viscometer for Measurement of Viscosity, Density, and Bubble-Point Pressure of CO2 and Organic Liquid Mixtures. Int. J. Thermophys. 2010, 31, 1896–1903. https://doi.org/10.1007/s10765-008-0542-6.

(516)	Zivkovic, E. M.; Kijevcanin, M. L.; Radovic, I. R.; Serbanovic, S. P.; Djordjevic, B. D. Viscosity of the Binary Systems 2-Methyl-2-Propanol with n-Alkanes at T = (303.15, 308.15, 313.15, 318.15 and 323.15) K: Prediction and Correlation - New UNIFAC-VISCO Interaction Parameters. Fluid Phase Equilib. 2010, 299, 191–197. https://doi.org/10.1016/j.fluid.2010.10.009.

(517)	Gong, X.; Guo, Y.; Xiao, J.; Yang, Y.; Fang, W. Densities, Viscosities, and Refractive Indices of Binary Mixtures of 1,2,3,4-Tetrahydronaphthalene with Some n-Alkanes at T = (293.15 to 313.15) K. J. Chem. Eng. Data 2012, 57, 3278–3282. https://doi.org/10.1021/je300899n.

(518)	Fang, S.; Zuo, X.-B.; Xu, X.-J.; Ren, D.-H. Density, Viscosity and Excess Molar Volume of Binary Mixtures of Tri-n-Octylamine + Diluents (n-Heptane, n-Octane, n-Nonane, and n-Decane) at Various Temperatures. J. Chem. Thermodyn. 2014, 68, 281–287. https://doi.org/10.1016/j.jct.2013.09.017.

(519)	Qin, X.; Cao, X.; Guo, Y.; Xu, L.; Hu, S.; Fang, W. Density, Viscosity, Surface Tension, and Refractive Index for Binary Mixtures of 1,3-Dimethyladamantane with Four C10 Alkanes. J. Chem. Eng. Data 2014, 59, 775–783. https://doi.org/10.1021/je4008926.

(520)	Kendall, J.; Wright, A. H. The Viscosity of Liquids. IV. Ideal Mixtures of the Types Ether-Ether and Ester-Ester. J. Am. Chem. Soc. 1920, 42, 1776–1779.

(521)	Meng, X.; Zheng, P.; Wu, J.; Liu, Z. Density and Viscosity Measurements of Diethyl Adipate from (303 to 373) K and up to 20 MPa. J. Chem. Eng. Data 2008, 53, 1474–1478. https://doi.org/10.1021/je700740e.

(522)	Sah, R. S.; Sinha, B.; Roy, M. N. Study of the Solution Properties of Ternary Mixtures of 1,3-Dioxolane (1), Diethyl Ether (2), and n-Amyl Alcohol (3) and the Corresponding Binary Mixtures by Density, Viscosity, Refractivity, and Ultrasonic Speed Measurements at 298.15 K. J. Chem. Eng. Data 2010, 55, 4536–4540. https://doi.org/10.1021/je100594u.

(523)	Sah, R. S.; Roy, M. N. Volumetric, Viscometric, Interferrometric and Refractometric Properties of 2-MethoxyethanolYdiethyletherYdichloromethane Ternary System and Its Corresponding Binaries at 298.15K. Phys. Chem. Liq. 2011, 49, 133–144. https://doi.org/10.1080/00319100903012395.

(524)	Dash, S. K.; Pradhan, S. K.; Dalai, B.; Moharana, L.; Swain, B. B. Studies on Molecular Interaction in Binary Mixtures of Diethyl Ether with Some Alkanols an Acoustic Approach. Phys. Chem. Liq. 2012, 50, 735–749. https://doi.org/10.1080/00319104.2012.713554.

(525)	Bi, S.; Wu, J.; Liu, Z.; Meng, X. Experimental Investigation of the Viscosity of Saturated Liquid Dimethyl Carbonate. Xi’an Jiaotong Daxue Xuebao 2003, 37, 903.

(526)	Wright, F. J. Influence of Temperature on Viscosity of Nonassociated Liquids. J. Chem. Eng. Data 1961, 6, 454–456. https://doi.org/10.1021/je00103a035.

(527)	Naejus, R.; Lemordant, D.; Coudert, R.; Willmann, P. Excess Thermodynamic Properties of Binary Mixtures Containing Linear or Cyclic Carbonates as Solvents at the Temperatures 298.15 K and 315.15 K. J. Chem. Thermodyn. 1997, 29, 1503–1515. https://doi.org/10.1006/jcht.1997.0260.

(528)	Pal, A.; Dass, G.; Kumar, A. Excess Molar Volumes, Viscosities, and Refractive Indices of Triethylene Glycol Dimethyl Ether with Dimethyl Carbonate, Diethyl Carbonate, and Propylene Carbonate at 298.15 K. J. Chem. Eng. Data 1998, 43, 738–741. https://doi.org/10.1021/je980016t.

(529)	Pal, A.; Kumar, H.; Kumar, A.; Dass, G. Excess Molar Volumes and Viscosities of Mixtures of Some N-Alkoxyethanols with Dialkyl Carbonates at 298.15 K. Fluid Phase Equilib. 1999, 166, 245–258. https://doi.org/10.1016/s0378-3812(99)00282-4.

(530)	Comunas, M. J. P.; Baylaucq, A.; Boned, C.; Fernandez, J. High-Pressure Measurements of the Viscosity and Density of Two Polyethers and Two Dialkyl Carbonates. Int. J. Thermophys. 2001, 22, 749–768. https://doi.org/10.1023/a:1010770831215.

(531)	Pal, A.; Bhardwaj, R. K.; Dass, G. Excess Molar Volumes and Viscosities of Mixtures of Diethylene Glycol Dibutyl Ether with Dialkyl Carbonates at 298.15, 308.15, and 318.15 K. Int. J. Thermophys. 2001, 22, 769–787. https://doi.org/10.1023/a:1010722915285.

(532)	Mialkowski, C.; Chagnes, A.; Carre, B.; Lemordant, D.; Willmann, P. Excess Thermodynamic Properties of Binary Liquid Mixtures Containing Dimethylcarbonate and -Butyrolactone. J. Chem. Thermodyn. 2002, 34, 1847–1856. https://doi.org/10.1016/s0021-9614(02)00232-x.

(533)	Rodriguez, A.; Canosa, J.; Dominguez, A.; Tojo, J. Dynamic Viscosities of Diethyl Carbonate with Linear and Secondary Alcohols at Several Temperatures. J. Chem. Eng. Data 2004, 49, 157–162. https://doi.org/10.1021/je0341413.

(534)	Arbad, B. R. R.; Lande, M. K. K.; Wankhede, N.; Wankhede, D. S. Viscosities, Ultrasonic Velocities at (288.15 and 298.15) K, and Refractive Indices at (298.15) K of Binary Mixtures of 2,4,6-Trimethyl-1,3,5-Trioxane with Dimethyl Carbonate, Diethyl Carbonate, and Propylene Carbonate. J. Chem. Eng. Data 2006, 51, 68–72. https://doi.org/10.1021/je050183a.

(535)	Comelli, F.; Francesconi, R.; Bigi, A.; Rubini, K. Excess Molar Enthalpies, Molar Heat Capacities, Densities, Viscosities, and Refractive Indices of Dimethyl Sulfoxide + Esters of Carbonic Acid at 308.15 K and Atmospheric Pressure. J. Chem. Eng. Data 2006, 51, 665–670. https://doi.org/10.1021/je050444g.

(536)	Rodriguez, A.; Pereiro, A. B.; Canosa, J.; Tojo, J. Dynamic Viscosities of the Ternary Liquid Mixtures (Dimethyl Carbonate + Methanol + Ethanol) and (Dimethyl Carbonate + Methanol + Hexane) at Several Temperatures. J. Chem. Thermodyn. 2006, 38, 505–519. https://doi.org/10.1016/j.jct.2005.07.008.

(537)	Iloukhani, H.; Zarei, H. A.; Behroozi, M. Thermodynamic Studies of Ternary Mixtures of Diethylcarbonate (1)+dimethylcarbonate (2)+N,N-Dimethylacetamide (3) at 298.15 K. J. Mol. Liq. 2007, 135, 141–145.

(538)	Meng, X.; Zheng, P.; Wu, J.; Liu, Z.-G. Viscosity and Density Measurements of Dimethyl Carbonate. J. Eng. Thermophys. 2009, 30, 26–30.

(539)	Xing, Y.; Shao, D.; Fang, W.; Guo, Y.; Lin, R. Vapor Pressures and Flash Points for Binary Mixtures of Tricyclo [5.2.1.0^(2.6)] Decane and Dimethyl Carbonate. Fluid Phase Equilib. 2009, 284, 14–18. https://doi.org/10.1016/j.fluid.2009.06.002.

(540)	Comelli, F.; Bigi, A.; Vitalini, D.; Rubini, K. Densities, Viscosities, Refractive Indices, and Heat Capacities of Poly(Ethylene Glycol-Ran-Propylene Glycol) + Esters of Carbonic Acid at (293.15 and 313.15) K and at Atmospheric Pressure. J. Chem. Eng. Data 2010, 55, 205–210. https://doi.org/10.1021/je900307z.

(541)	Litaiem, Y.; Dhahbi, M. Measurements and Correlations of Viscosity, Conductivity and Density of an Hydrophobic Ionic Liquid (Aliquat 336) Mixtures with a Non-Associated Dipolar Aprotic Solvent (DMC). J. Mol. Liq. 2012, 169, 54–62. https://doi.org/10.1016/j.molliq.2012.02.019.

(542)	Meng, X.; Zhang, J.; Wu, J. T.; Liu, Z.-G. Experimental Measurement and Modeling of the Viscosity of Dimethyl Ether. J. Chem. Eng. Data 2012, 57, 988–993. https://doi.org/10.1021/je201297j.

(543)	Tomida, D.; Nagasaka, T.; Hongo, M.; Yokoyama, C. Viscosity of Gaseous Mixtures of Methoxymethane + Nitrogen. J. Chem. Eng. Data 2009, 54, 1343–1347. https://doi.org/10.1021/je8009545.

(544)	Wang, X. -p.; Song, B.; Wu, J. -t.; Liu, Z.-G. Gaseous Viscosity of Dimethyl Ether in the Low Density Region. J. Eng. Thermophys. (China) 2011, 32, 910–912.

(545)	Cousins, D. S.; Laesecke, A. Sealed Gravitational Capillary Viscometry of Dimethyl Ether and Two Next-Generation Alternative Refrigerants. J. Res. Natl. Inst. Stand. Technol. 2012, 117, 231–255. https://doi.org/10.6028/jres.117.014.

(546)	Thorpe, T. E.; Rodger, J. W. On the Relations between the Viscosity (Internal Friction) of Liquids and Their Chemical Nature. Part II. Philos. Trans. R. Soc. London, Ser. A 1897, 189, 71.

(547)	Schmidt, A. W.; Hopp, G.; Schoeller, V. Preparation and Physical Data for Monoalkyl Benzenes. Ber. Dtsch. Chem. Ges., B 1939, 72, 1893–1897.

(548)	Panchenkov, G. M.; Erchenkov, V. V. Temperature Variation of the Coordination Number and Diffusion Coefficient in a Liquid. Russ. J. Phys. Chem. 1962, 36, 455–457.

(549)	Barlow, A. J.; Lamb, J. D.; Matheson, A. J. Proc. R. Soc. London, Ser. A 1966, 292, 322.

(550)	Akhundov, T. S. Viscosity of Ethylbenzene. Izv. Vyssh. Uchebn. Zaved., Neft Gaz 1973, 16, 46;74.

(551)	Shikhaliev, Y. A. Relationship between Viscosity and Activation Energy for Binary Liquid Solutions. Zh. Fiz. Khim. 1977, 51, 961–963.

(552)	Abdullaev, F. G.; Akhundov, T. S. Experimental Study of the Viscosity of Ethylbenzene. Izv. Vyssh. Uchebn. Zaved., Neft Gaz 1985, 28, 52.

(553)	Fermeglia, M.; Lapasin, R. Excess Volumes and Viscosities of Binary Mixtures of Organics. J. Chem. Eng. Data 1988, 33, 415–417. https://doi.org/10.1021/je00054a008.

(554)	Singh, R. P.; Sinha, C. P.; Das, J. C.; Ghosh, P. Viscosity and Density of Ternary Mixtures for Toluene, Ethylbenzene, Bromobenzene, and 1-Hexanol. J. Chem. Eng. Data 1989, 34, 335–338. https://doi.org/10.1021/je00057a022.

(555)	Krahn, U. G.; Luft, G. Viscosity of Several Liquid Hydrocarbons in the Temperature Range 298-453 K at Pressures up to 200 MPa. J. Chem. Eng. Data 1994, 39, 670–672. https://doi.org/10.1021/je00016a006.

(556)	Katyal, R. C.; Singh, S.; Rattan, V. K.; Kanda, P.; Acharya, S. Viscosities, Densities, and Ultrasonic Velocities of 3-Pentanone + Ethylbenzene and 3-Pentanone + o-Xylene at (293.15, 303.15, and 313.15) K. J. Chem. Eng. Data 2003, 48, 1262–1265. https://doi.org/10.1021/je030151s.

(557)	Rattan, V. K.; Singh, S.; Sethi, B. P. S. Viscosities, Densities, and Ultrasonic Velocities of Binary Mixtures of Ethylbenzene with Ethanol, 1-Propanol, and 1-Butanol at (298.15 and 308.15)K. J. Chem. Eng. Data 2004, 49, 1074–1077. https://doi.org/10.1021/je049912x.

(558)	Rathnam, M. V.; Mohite, S.; Kumar, M. S. S. Viscosity, Density, and Refractive Index of Some (Ester + Hydrocarbon) Binary Mixtures at 303.15 K and 313.15 K. J. Chem. Eng. Data 2005, 50, 325–329. https://doi.org/10.1021/je0400052.

(559)	Yang, C.; Yu, W.; Ma, P. Densities and Viscosities of Binary Mixtures of Ethylbenzene + N-Methyl-2-Pyrrolidone, Ethylbenzene + Sulfolane, and Styrene + Octane from (303.15 to 353.15) K and Atmospheric Pressure. J. Chem. Eng. Data 2005, 50, 1197–1203. https://doi.org/10.1021/je049572f.

(560)	Dominguez-Perez, M.; Franjo, C.; Pico, J.; Segade, L.; Cabeza, O.; Jimenez, E. Experimental Study of the Dynamic Viscosity Deviations in the Binary Systems: Hexane + Ethylbenzene, + o-Xylene, + m-Xylene, + p-Xylene at 298.15 K. Int. J. Thermophys. 2009, 30, 1197–1201. https://doi.org/10.1007/s10765-009-0622-2.

(561)	Rathnam, M. V.; Mohite, S.; Kumar, M. S. Thermophysical Properties of Isoamyl Acetate or Methyl Benzoate + Hydrocarbon Binary Mixtures, at (303.15 and 313.15) K. J. Chem. Eng. Data 2009, 54, 305–309. https://doi.org/10.1021/je800325d.

(562)	Rathnam, M. V.; Mohite, S.; Kumar, M. S. S. Density, Excess Volume, and Viscosity of Vinyl Acetate or Benzyl Acetate with (o-, m-, p-)Xylenes and Ethylbenzene at T = (303.15 and 313.15) K. J. Solution Chem. 2010, 39, 1735–1748. https://doi.org/10.1007/s10953-010-9610-1.

(563)	Bhatia, S. C.; Rani, R.; Bhatia, R. Viscosities, Densities, Speeds of Sound and Refractive Indices of Binary Mixtures of o-Xylene, m-Xylene, p-Xylene, Ethylbenzene and Mesitylene with 1-Decanol at 298.15 and 308.15 K. J. Mol. Liq. 2011, 159, 132–141. https://doi.org/10.1016/j.molliq.2010.12.011.

(564)	Rathnam, M. V.; Mohite, S.; Kumar, M. S. S. Density and Viscosity of Alkyl Acetates + Aromatic Hydrocarbons at (303.15 and 313.15) K. J. Solution Chem. 2011, 40, 390–402.

(565)	Saravanakumar, K.; Lavanya, T. G.; Baskaran, R.; Kubendran, T. R. Thermophysical and Thermoacoustical Properties of Benzaldehyde Mixtures with Ethylbenzene at 303.15, 308.15, and 313.15 K and a Pressure of 0.1 MPa. Zh. Fiz. Khim. 2012, 86, 647–655.

(566)	Barega, E. W.; Zondervan, E.; Haan, A. B. d. Experimental Density, Viscosity, Interfacial Tension and Water Solubility of Ethyl Benzene - a-Methyl Benzyl Alcohol - Water System. J. Chem. Thermodyn. 2013, 63, 31–37. https://doi.org/10.1016/j.jct.2013.03.023.

(567)	Zhang, J.; Zhang, P.; Han, F.; Chen, G.; Deng, R.; Wei, X.-H. Gas-Liquid Equilibrium Data for a Mixture Gas of Sulfur Dioxide + Nitrogen with Ethylene Glycol Aqueous Solutions at 298.15 K and 123.15 KPa. J. Chem. Eng. Data 2008, 53, 2372–2374. https://doi.org/10.1021/je800271e.

(568)	Taylor, C. A.; Rinkenbach, W. H. Ethylene Glycol an Evaluation of Available Information on the Physical Properties of This Compound. Ind. Eng. Chem. 1926, 18, 676–678.

(569)	Rinkenbach, W. H. Properties of Diethylene Glycol. Ind. Eng. Chem. 1927, 19, 475–476.

(570)	Timmermans, J.; Hennaut-Roland, M. Physical Constants of 20 Organic Compounds. VII. J. Chim. Phys. Phys.-Chim. Biol. 1935, 32, 501.

(571)	Mellan, I. Polyhydric Alcohols; 1962.

(572)	Davids, E. L.; Findlay, T. J. V. Phase Diagram for Ethylene Glycol and Triethylamine. Aust. J. Chem. 1967, 20, 1343.

(573)	Jerome, F. S.; Tseng, J. T.; Fan, L. T. Viscosities of Aqueous Glycerol Solutions. J. Chem. Eng. Data 1968, 13, 496–496. https://doi.org/10.1021/je60039a010.

(574)	Oyevaar, M. H.; Morssinkhof, R. W. J.; Westerterp, K. R. Density, Viscosity, Solubility, and Diffusivity of Carbon Dioxide an Nitrous Oxide in Solutions of Diethanolamine in Aqueous Ethylene Glycol at 298 K. J. Chem. Eng. Data 1989, 34, 77–82. https://doi.org/10.1021/je00055a022.

(575)	Kumagai, A.; Mochida, H.; Takahashi, S. Liquid Viscosities and Densities of HFC-134a (1,1,1,2- Tetrafluoroethane) + Glycol Mixtures. Int. J. Thermophys. 1993, 14, 45–53. https://doi.org/10.1007/bf00522660.

(576)	Reddy, V. K.; Reddy, K. S.; Krishnaiah, A. Excess Volumes, Speeds of Sound, and Viscosities for Mixtures of 1,2- Ethanediol and Alkoxy Alcohols with Water at 308.15 K. J. Chem. Eng. Data 1994, 39, 615–617. https://doi.org/10.1021/je00015a051.

(577)	Pal, A.; Singh, W. Speeds of Sound and Viscosities in Aqueous Poly(Ethylene Glycol) Solutions at 303.15 and 308.15 K. J. Chem. Eng. Data 1997, 42, 234–237. https://doi.org/10.1021/je960173x.

(578)	Pal, A.; Sharma, S. Excess Molar Volumes and Viscosities of 1-Propanol + Ethylene Glycol, + Ethylene Glycol Monomethyl, + Ethylene Glycol Dimethyl, + Diethylene Glycol Dimethyl, + Triethylene Glycol Dimethyl, + Diethylene Glycol Diethyl, and + Diethylene Glycol Dibutyl Ethers at 298.15 K. J. Chem. Eng. Data 1998, 43, 532–536. https://doi.org/10.1021/je9702955.

(579)	Tsierkezos, N. G.; Molinou, I. E. Thermodynamic Properties of Water + Ethylene Glycol at 283.15, 293.15, 303.15, 313.15. J. Chem. Eng. Data 1998, 43, 989–993. https://doi.org/10.1021/je9800914.

(580)	Saleh, M.; Begum, S.; Begum, S. K.; Begum, B. A. VISCOSITY OF DILUTE AQUEOUS SOLUTIONS OF SOME DIOLS. Phys. Chem. Liq. 1999, 37, 785–801.

(581)	Lech, T.; Czechowski, G.; Jadzyn, J. Viscosity of the Series of 1,n-Alkanediols. J. Chem. Eng. Data 2001, 46, 725–727. https://doi.org/10.1021/je0003750.

(582)	Vijaya Kumar Naidu, B.; Chowdoji Rao, K.; Subha, M. C. S. Densities and Viscosities of Mixtures of Some Glycols and Polyglycols in Dimethyl Sulfoxide at 308.15 K. J. Chem. Eng. Data 2002, 47, 379–382. https://doi.org/10.1021/je0101395.

(583)	Naidu, B. V. K.; Rao, K. C.; Subha, M. C. S. Densities, Viscosities, and Excess Properties for Binary Mixtures of Some Glycols and Polyglycols in N-Methylacetamide at 308.15 K. J. Chem. Eng. Data 2003, 48, 625–627. https://doi.org/10.1021/je020178w.

(584)	Sastry, N. V.; Patel, M. C. Densities, Excess Molar Volumes, Viscosities, Speeds of Sound, Excess Isentropic Compressibilities, and Relative Permittivities for Alkyl (Methyl, Ethyl, Butyl, and Isoamyl) Acetates + Glycols at Different Temperatures. J. Chem. Eng. Data 2003, 48, 1019–1027. https://doi.org/10.1021/je0340248.

(585)	Sun, T.; Teja, A. S. Density, Viscosity and Thermal Conductivity of Aqueous Ethylene, Diethylene and Triethylene Glycol Mixtures between 290 K and 450 K. J. Chem. Eng. Data 2003, 48, 198–202. https://doi.org/10.1021/je025610o.

(586)	Yang, C.; Ma, P.; Jing, F.; Tang, D. Excess Molar Volumes, Viscosities, and Heat Capacities for the Mixtures of Ethylene Glycol + Water from 273.15 K to 353.15 K. J. Chem. Eng. Data 2003, 48, 836–840. https://doi.org/10.1021/je020140j.

(587)	Sinha, A.; Roy, M. N. Densities, Viscosities, and Sound Speeds of Some Acetate Salts in Binary Mixtures of Tetrahydrofuran and Methanol at (303.15, 313.15, and 323.15) K. J. Chem. Eng. Data 2006, 51, 1415–1423. https://doi.org/10.1021/je060113j.

(588)	Yang, C.; Liu, Z.; Lai, H.; Ma, P. Excess Molar Volumes and Viscosities of Binary Mixtures of P-Cresol with Ethylene Glycol and Methanol at Different Temperature and Atmospheric Pressure. J. Chem. Eng. Data 2006, 51, 457–461. https://doi.org/10.1021/je050376y.

(589)	Yang, C.; Yu, W.; Tang, D. Densities and Viscosities of Binary Mixtures of M-Cresol with Ethylene Glycol or Methanol over Several Temperatures. J. Chem. Eng. Data 2006, 51, 935–939. https://doi.org/10.1021/je0504156.

(590)	Gurung, B. B.; Roy, M. N. Study of Densities, Viscosity Deviations, and Isentropic Compressibilities of Ternary Liquid Mixtures of Water and Ethane-1,2-Diol with Some Monoalcohols at Various Temperatures. Phys. Chem. Liq. 2007, 45, 331–343.

(591)	Mehta, S. K.; Ram, G.; Kumar, V.; Bhasin, K. K. Structural and Interactional Studies of Homologous Series of a,x-Alkanediols in N,N-Dimethylformamide. J. Chem. Thermodyn. 2007, 39, 781–790. https://doi.org/10.1016/j.jct.2006.10.004.

(592)	Song, X.; Luo, Y.; Wang, J.; Yu, J. Determination and Correlation of the Densities and Viscosities of Methanol-Glycol Binary Mixtures. Huadong Huagong Xueyuan Xuebao 2007, 33, 4;750.

(593)	Wu, C.; Liu, D.; Ma, Z.; Liu, X. Determination and Correlation of Viscosities for Ethylene Glycol and Diethylene Glycol with a Catalyst. Nanjing Huagong Daxue Xuebao 2007, 29, 4;51.

(594)	Mehta, S. K.; Ram, G.; Bhasin, K. K. Molecular Interactions of a,x-Alkanediols in Pyrrolidin-2-One: Thermophysical and Spectroscopic Measurements. J. Chem. Thermodyn. 2008, 40, 498–508. https://doi.org/10.1016/j.jct.2007.08.005.

(595)	Nain, A. K. Ultrasonic and Viscometric Studies of Molecular Interactions in Binary Mixtures of Formamide with Ethanol, 1-Propanol, 1,2-Ethanediol and 1,2-Propanediol at Different Temperatures. J. Mol. Liq. 2008, 140, 108–116.

(596)	Shi, D.; Gao, G.; Li, D.; Dong, J.; Wang, L. New Device for Fast Measuring Surface Tension, Density and Viscosity of Liquids. Fluid Phase Equilib. 2008, 273, 87–91. https://doi.org/10.1016/j.fluid.2008.08.015.

(597)	Zorebski, E.; Lubowiecka-Kostka, B. Thermodynamic and Transport Properties of (1,2-Ethanediol + 1-Nonanol) at Temperatures from (298.15 to 313.15) K. J. Chem. Thermodyn. 2009, 41, 197–204. https://doi.org/10.1016/j.jct.2008.09.018.

(598)	Doghaeei, A.; Rostami, A.; Omrani, A. Densities, Viscosities, and Volumetric Properties of Binary Mixtures of 1,2-Propanediol + 1-Heptanol or 1-Hexanol and 1,2-Ethanediol + 2-Butanol or 2-Propanol at T = (298.15, 303.15, and 308.15) K. J. Chem. Eng. Data 2010, 55, 2894–2899. https://doi.org/10.1021/je900954y.

(599)	Ge, M.-L.; Ma, J.-L.; Chu, B. Densities and Viscosities of Propane-1,2,3-Triol + Ethane-1,2-Diol at T = (298.15 to 338.15) K. J. Chem. Eng. Data 2010, 55, 2649–2651. https://doi.org/10.1021/je900938b.

(600)	Li, H.; Chen, X.; Zhang, Z. Measurement and Correlation of the Densities and Viscosities of 2-Bromopropane + Ethanol Binary Mixtures at Temperatures from (298.15 to 318.15) K. J. Chem. Eng. Data 2010, 55, 3441–3444. https://doi.org/10.1021/je100159m.

(601)	Kinart, C. M.; Klimczak, M.; Kinart, W. Viscosimetric and Structural Properties of Binary Mixtures of 2-Butoxyethanol with Ethylene Glycol, Diethylene Glycol, Triethylene Glycol and Tetraethylene Glycol Systems at T^293.15, 298.15 and 303.15K. Phys. Chem. Liq. 2011, 49, 181–191. https://doi.org/10.1080/00319100903191405.

(602)	Kinart, C. M.; Maj, M. Viscosities of Ternary Mixtures 2-Alkoxyethanol + Sulfolane + Ethylene Glycols at T^303.15K. Phys. Chem. Liq. 2011, 49, 508–518.

(603)	Sagdeev, D. I.; Fomina, M. G.; Mukhamedzyanov, G. K.; Abdulagatov, I. M. Experimental Study of the Density and Viscosity of Polyethylene Glycols and Their Mixtures at Temperatures from 293 K to 473 K and at Atmospheric Pressure. J. Chem. Thermodyn. 2011, 43, 1824–1843. https://doi.org/10.1016/j.jct.2011.06.013.

(604)	Sagdeev, D. I.; Fomina, M. G.; Mukhamedzyanov, G. K.; Abdulagatov, I. M. Experimental Study of the Density and Viscosity of Polyethylene Glycols and Their Mixtures at Temperatures from 293 K to 465 K and at High Pressures up to 245 MPa. Fluid Phase Equilib. 2012, 315, 64–76. https://doi.org/10.1016/j.fluid.2011.11.022.

(605)	Gao, F.; Niu, Y.; Zhang, J.; Sun, S.; Wei, X. Solubility for Dilute Sulfur Dioxide in Binary Mixtures of N,N-Dimethylformamide + Ethylene Glycol at T = 308.15 Kand p = 122.66 KPa. J. Chem. Thermodyn. 2013, 62, 8–16. https://doi.org/10.1016/j.jct.2013.02.010.

(606)	Moosavi, M.; Motahari, A.; Omrani, A.; Rostami, A. A. Investigation on Some Thermophysical Properties of Poly(Ethylene Glycol) Binary Mixtures at Different Temperatures. J. Chem. Thermodyn. 2013, 58, 340–350. https://doi.org/10.1016/j.jct.2012.11.016.

(607)	Quijada-Maldonado, E.; Meindersma, G. W.; De Haan, A. B. Viscosity and Density Data for the Ternary System Water(1) Ethanol(2) Ethylene Glycol(3) between 298.15 K and 328.15 K. J. Chem. Thermodyn. 2013, 57, 500–505. https://doi.org/10.1016/j.jct.2012.08.024.

(608)	Kondaiah, M.; Sreekanth, K.; Kumar, D. S.; Nayeem, S. M.; Rao, D. K. Densities, Viscosities, and Excess Properties for Binary Mixtures of Ethylene Glycol with Amides at 308.15 K. J. Therm. Anal. Calorim. 2014, 118, 475–483. https://doi.org/10.1007/s10973-014-4019-5.

(609)	Li, Y.; Zheng, D.; Dong, L.; Nie, N.; Xiong, B. Solubilities of CO2 in, and Densities and Viscosities of, the Piperazine + 1-Ethyl-3-Methyl-Imidazolium Acetate + H2O System. J. Chem. Eng. Data 2014, 59, 618–625. https://doi.org/10.1021/je400491p.

(610)	Pastoriza-Gallego, M. J.; Lugo, L.; Cabaleiro, D.; Legido, J. L.; Pineiro, M. M. Thermophysical Profile of Ethylene Glycol-Based ZnO Nanofluids. J. Chem. Thermodyn. 2014, 73, 23–30. https://doi.org/10.1016/j.jct.2013.07.002.

(611)	Zhang, Z.; Zhou, Q.; Lu, X.; Qiao, C.; Zhang, S. Densities and Viscosities of Binary Mixtures Containing 1,3-Dimethylimidazolium Dimethylphosphate and Alcohols. J. Chem. Eng. Data 2014, 59, 2377–2388. https://doi.org/10.1021/je401124t.

(612)	Sun, S.; Niu, Y.; Gao, F.; Shen, J.; Wei, X. Solubility Properties and Spectral Characterization of Dilute SO2 in Binary Mixtures of Urea + Ethylene Glycol. J. Chem. Eng. Data 2015, 60, 161–170. https://doi.org/10.1021/je5009119.

(613)	Zhang, H.; Gong, M.; Li, H.; Guo, H.; Dong, X.; Wu, J. Gaseous PVTx Measurements for (HFC134a+HC290) by a Compact Single-Sinker Densimeter. J. Chem. Thermodyn. 2015, 81, 60–65. https://doi.org/10.1016/j.jct.2014.10.001.

(614)	Diller, D. E.; Saber, J. M. Measurements of the Viscosity of Compressed Gaseous and Liquid Ethane. Physica A (Amsterdam) 1981, 108, 143–152.

(615)	Gerf, S. F.; Galkov, G. I. Viscosity of Liquified Pure Gases and Their Mixtures. Zh. Tekh. Fiz. 1940, 10, 725–732.

(616)	Galkov, G. I.; Gerf, S. F. Viscosity of Liquefied Pure Gases and Their Mixtures. Zh. Tekh. Fiz. 1941, 11, 613–615.

(617)	Ishida, Y. Determination of Viscosities and of the Stokes-Millikan Law Constant by the Oil-Drop Method. Phys. Rev. 1923, 21, 550.

(618)	Trautz, M.; Sorg, K. G. Viscosity, Heat Conductivity and Diffusion in Gas Mixtures. XVI. The Viscosity of u Hydrogen, Methane, Ethane, Propane and Their Binary Mixtures. Ann. Phys. (Berlin) 1931, 10, 81–96.

(619)	De Rocco, A.; Halford, I. Intermolecular Potentials of Argon, Methane and Ethane. J. Chem. Phys. 1958, 28, 1152.

(620)	Baron, J. D.; Roof, J. G.; Wells, F. W. Viscosity of Nitrogen, Methane, Ethane, and Propane at Elevated Temperature and Pressure. J. Chem. Eng. Data 1959, 4, 283–288. https://doi.org/10.1021/je60003a024.

(621)	Iwasaki, H.; Takahashi, M. Viscosity of Carbon Dioxide and Ethane. J. Chem. Phys. 1981, 74, 1930.

(622)	Wilhelm, J.; Seibt, D.; Vogel, E.; Buttig, D.; Hassel, E. Viscosity Measurements on Gaseous Ethane. J. Chem. Eng. Data 2006, 51, 136–144. https://doi.org/10.1021/je050287l.

(623)	Smith, A. S.; Brown, G. G. Correlating Fluid Viscosity. Ind. Eng. Chem. 1943, 35, 705.

(624)	Eakin, B. E.; Starling, K. E.; Dolan, J. P.; Ellington, R. T. Liquid, Gas, and Dense Fluid Viscosity of Ethane. J. Chem. Eng. Data 1962, 7, 33–36. https://doi.org/10.1021/je60012a010.

(625)	Seibt, D.; Voss, K.; Herrmann, S.; Vogel, E.; Hassel, E. Simultaneous Viscosity-Density Measurements on Ethane and Propane over a Wide Range of Temperature and Pressure Including the Near-Critical Region. J. Chem. Eng. Data 2011, 56, 1476–1493. https://doi.org/10.1021/je101178u.

(626)	Hendl, S.; Vogel, E. The Viscosity of Gaseous Ethane and Its Initial Density Dependence. Fluid Phase Equilib. 1992, 76, 259–272. https://doi.org/10.1016/0378-3812(92)85093-n.

(627)	Mizushima, S. Bull. Chem. Soc. Jpn. 1926, 1, 143.

(628)	Hatem, S. Study of the Mixtures Ethanol-Pyridine, Ethanol-Quinoline. Bull. Soc. Chim. Fr. 1949, 599.

(629)	Khalimonova. Ukr. Fiz. Zh. 1962, 7, 483.

(630)	Brunson, R. R.; Byers, C. H. Viscosities of Alcohol-Hydrocarbon Systems in the Critical Region. A Dynamic Laser Light Scattering Approach. J. Chem. Eng. Data 1989, 34, 46–52. https://doi.org/10.1021/je00055a015.

(631)	Ali, A.; Nain, A. K.; Kamil, M. Physical-Chemical Studies of Nonaqueous Binary Liquid Mixtures at Various Temperatures. Thermochim. Acta 1996, 274, 209–221. https://doi.org/10.1016/0040-6031(95)02719-x.

(632)	Thole, F. B. CCLXVI. Viscosity and Association Part I. Association of the Phenols. J. Chem. Soc. 1910, 97, 2596–2606.

(633)	Bingham, E. C.; White, G. F.; Thomas, A.; Cadwell, J. L. Fluidity and Hydration Theory. II. Z. Phys. Chem. (Leipzig) 1913, 83, 641–673.

(634)	Dolian, F. E.; Briscoe, H. T. The Viscosities of Solutions of Chlorides in Certain Solvents. J. Phys. Chem. 1937, 41, 1129.

(635)	Vierk, A.-L. Experimental Investigation of the Two-Component Systems: Water-Aceto- Nitrole, Water-Dioxane, Ethanol-Acetonitrile and Cyclohexane-Dioxane. Z. Anorg. Chem. 1950, 261, 283–296. https://doi.org/10.1002/zaac.19502610504.

(636)	Misra, B. N.; Varshni, Y. P. Viscosity Temperature Relation for Solutions. J. Chem. Eng. Data 1961, 6, 194–196. https://doi.org/10.1021/je60010a007.

(637)	Bamelis, P.; Huyskens, P.; Meeussen, E. No.18 Influence of the Association of Alcohols on The Viscosity of Solutions. J. Chim. Phys. Phys.-Chim. Biol. 1965, 62, 158–170.

(638)	Komarenko, V. G.; Manzhelii, V. G.; Radtsig, A. V. Density and Viscosity of Normal Alcohols at Low Temperatures. Ukr. Fiz. Zh. 1967, 12, 676.

(639)	Tommila, E.; Lindell, E.; Virtalaine, M.-L.; Laakso, R. Densities, Viscosities, Surface Tensions, Dielectric Constants, Vapor Pressures, Activities, and Heats of Mixing of Sulpholane + Water, Sulpholane + Methanol, and Sulpholane + Ethanol Mixtures. Suom. Kemistil. B 1969, 42, 95–104.

(640)	Phillips, T. W.; Murphy, K. P. Liquid Viscosity in Halogenated Refrigerants. ASHRAE Trans. 1970, 76, 146–156.

(641)	Shikhaliev, Y. A.; Mamedov, A. A.; Aleskerov, M. A.; Khalilov, S. K. Temperature and Concentration Variation of the Viscosities of Toluene- Ethanol and Heptane-Ethanol Mixtures. Zh. Fiz. Khim. 1974, 48, 1696.

(642)	Lee, F.-M.; Lahti, L. E.; Stoops, C. E. Solution Properties of Urea + Alcohol + Water Mixtures. J. Chem. Eng. Data 1976, 21, 36–40. https://doi.org/10.1021/je60068a003.

(643)	Tanaka, Y.; Yamamoto, T.; Satomi, Y.; Kubota, H.; Makita, T. Specific Volume and Viscosity of Ethanol-Water Mixtures under High Pressure. Rev. Phys. Chem. Jpn. 1977, 47, 12–24.

(644)	Baykut, S.; Tanrikulu, M. Investigations of Certain Properties of N-Acids and n-Alcohols and Some Mathematical Relationships between These Properties and Their Carbon Numbers. Chim. Acta Turc. 1980, 8, 69–76.

(645)	Pikkarainen, L. Density & Viscosity of Binary Solvent Mixtures of N-Methylmethane Sulfonamide with Ethanol, Pyridine & N,N-Dimethylacetamide. Finn. Chem. Lett. 1980, 185.

(646)	Kumar, A.; Prakash, O.; Prakash, S. Ultrasonic Velocities, Densities, and Viscosities of Trimethylamine in Methanol, Ethanol, and 1-Propanol. J. Chem. Eng. Data 1981, 26, 64–67. https://doi.org/10.1021/je00023a021.

(647)	Pikkarainen, L. Densities and Viscosities of Binary Mixtures of N,N-Dimethylacetamide with Aliphatic Alcohols. J. Chem. Eng. Data 1983, 28, 344–347. https://doi.org/10.1021/je00033a019.

(648)	Pikkarainen, L. Densities and Viscosities of Binary Solvent Mixtures of N,N-Dimethyl- Mathanesulfonamide with Aliphatic Alcohols. Finn. Chem. Lett. 1983, 63.

(649)	Rauf, M. A.; Stewart, G. H.; Farhat-Aziz. Viscosities and Densities of Binary Mixtures of 1-Alkanols from 15 to 55 C. J. Chem. Eng. Data 1983, 28, 324–328. https://doi.org/10.1021/je00033a012.

(650)	Papanastasiou, G. E.; Papoutsis, A. D.; Kokkinidis, I. Physical Behavior of Some Reaction Media. Density, Viscosity, Dielectric Constant, and Refractive Index Changes of Ethano-Dioxane Mixtures at Several Temperatures. J. Chem. Eng. Data 1987, 32, 377–381. https://doi.org/10.1021/je00049a028.

(651)	Tanaka, Y.; Matsuda, Y.; Fujiwara, H.; Kubota, H.; Makita, T. Viscosity of (Water + Alcohol) Mixtures under High Pressure. Int. J. Thermophys. 1987, 8, 147–163. https://doi.org/10.1007/bf00515199.

(652)	Crabtree, A. M.; O’Brien, J. F. Excess Viscosities of Binary Mixtures of Chloroform and Alcohols. J. Chem. Eng. Data 1991, 36, 140–142. https://doi.org/10.1021/je00002a003.

(653)	Garcia, B.; Herrera, C.; Leal, J. M. Shear Viscosities of Binary Liquid Mixtures: 2-Pyrrolidinone with 1- Alkanols. J. Chem. Eng. Data 1991, 36, 269–274. https://doi.org/10.1021/je00003a004.

(654)	Pikkarainen, L. Thermodynamic and Physicochemical Properties of Binary Solvent Mixtures of Hexamethylphosphoric Triamide with Aliphatic Alcohols. A Viscometric Study. Thermochim. Acta 1992, 197, 463–468. https://doi.org/10.1016/0040-6031(92)85047-y.

(655)	Aminabhavi, T. M.; Aralaguppi, M. I.; Harogoppad, S. B.; Balundgi, R. H. Densities, Viscosities, Refractive Indices, and Speeds of Sound for Methyl Acetoacetate + Aliphatic Alcohols (C1-C8). J. Chem. Eng. Data 1993, 38, 31–39. https://doi.org/10.1021/je00009a008.

(656)	Assael, M. J.; Dymond, J. H.; Polimatidou, S. K. Correlation and Prediction of Dense Fluid Transport Coefficients. VI. n-Alcohols. Int. J. Thermophys. 1994, 15 (2), 189–201.

(657)	Papaioannou, D.; Panayioton, C. Viscosity of Alkanol + Alkane Mixtures at Moderately High Pressures. J. Chem. Eng. Data 1994, 39, 463–466. https://doi.org/10.1021/je00015a013.

(658)	Rodriguez, M.; Galan, M.; Munoz, M. J.; Martin, R. Viscosity of Triglycerides + Alcohols from 278 to 313 K. J. Chem. Eng. Data 1994, 39, 102–105. https://doi.org/10.1021/je00013a027.

(659)	El-Banna, M. M.; Ramadan, M. Sh. Density and Viscosity of Octyl, Decyl, and Dodecyl Sodium Sulfates in Ethanol. J. Chem. Eng. Data 1995, 40, 367–370. https://doi.org/10.1021/je00018a004.

(660)	Lee, M. J.; Lin, T.-K. Density and Viscosity for Monoethanolamine + Water, + Ethanol, and + 2- Propanol. J. Chem. Eng. Data 1995, 40, 336–339. https://doi.org/10.1021/je00017a074.

(661)	Nikam, P. S.; Jadhav, M. C.; Hasan, M. Density and Viscosity of Mixtures of Nitrobenzene with Methanol, Ethanol, Propan-1-Ol, Propan-2-Ol, Butan-1-Ol, 2-Methylpropan-1-Ol, and 2- Methylpropan-2-Ol at 298.15 and 303.15 K. J. Chem. Eng. Data 1995, 40, 931–934. https://doi.org/10.1021/je00020a044.

(662)	Papaioannou, D.; Panayioton, C. Viscosity of Binary Mixtures of Propylamine with Alkanols at Moderately High Pressures. J. Chem. Eng. Data 1995, 40, 202–209. https://doi.org/10.1021/je00017a042.

(663)	Aucejo, A.; Burguet, M. C.; Munoz, R. Densities, Viscosities, and Refractive Indices of Some Binary Liquid Systems of Ethanol + Isomers of Hexanol at 298.15 K. J. Chem. Eng. Data 1996, 41, 1131–1134. https://doi.org/10.1021/je9601265.

(664)	Aalto, M.; Liukkonen, S. Bubble Point Pressures and Densities for the Binary Systems of Propane with Triacontane, Hexatriacontane, Tetracontane, Pentacontane, and Squalane at 353−373 K and 4.00−7.00 MPa. J. Chem. Eng. Data 1996, 41 (1), 79–83. https://doi.org/10.1021/je9501393.

(665)	Nikam, P. S.; Jadhay, M. C.; Hasan, M. Density and Viscosity of Mixtures of Dimethyl Sulfoxide + Methanol, + Ethanol, +Propan-1-Ol, +Propan-2-Ol, +Butan-1-Ol, +2-Methylpropan-1-Ol, and +2-Methylpropan-2-Ol at 298.15 K and 303.15 K. J. Chem. Eng. Data 1996, 41, 1028–1031. https://doi.org/10.1021/je9600358.

(666)	Nikam, P. S.; Mahale, T. R.; Hasan, M. Density and Viscosity of Binary Mixtures of Ethyl Acetate with Methanol, Ethanol, Propan-1-Ol, Propan-2-Ol, Butan-1-Ol, 2-Methylpropan-1-Ol, and 2- Methylpropan-2-Ol at (298.15, 303.15, and 308.15) K. J. Chem. Eng. Data 1996, 41, 1055–1058. https://doi.org/10.1021/je960090g.

(667)	Orge, B.; Iglesias, M.; Rodriguez, A.; Canosa, J. M.; Tojo, J. Mixing Properties of (Methanol, Ethanol, or 1-Propanol) with (n-Pentane, n-Hexane, n-Heptane and n-Octane) at 298.15 K. Fluid Phase Equilib. 1997, 133, 213–227. https://doi.org/10.1016/s0378-3812(97)00031-9.

(668)	Aminabhavi, T. M.; Patil, V. B. Density, Viscosity, and Speed of Sound in Binary Mixtures of 1-Chloronaphthalene with Methanol, Ethanol, Propan-1-Ol, Butan-1-Ol, Pentan-1-Ol, and Hexan-1-Ol in the Temperature Range (298.15-308.15) K. J. Chem. Eng. Data 1998, 43, 504–508. https://doi.org/10.1021/je980032q.

(669)	Canosa, J.; Rodriguez, A.; Tojo, J. Dynamic Viscosity and Speed of Sound of the Ternary Mixture Methyl Acetate + Methanol + Ethanol at 298.15. J. Chem. Eng. Data 1998, 43, 961–966. https://doi.org/10.1021/je980076z.

(670)	Kumagai, A.; Yokoyama, C. Liquid Viscosity of Binary Mixtures of Methanol with Ethanol and 1-Propanol from 273.15 to 333.15 K. Int. J. Thermophys. 1998, 19, 3–13. https://doi.org/10.1023/a:1021438800094.

(671)	Nikam, P. S.; Shirsat, L. N.; Hasan, M. Density and Viscosity Studies of Binary Mixtures of Acetonitrile with Methanol, Ethanol, Propan-1-Ol, Propan-2-Ol, Butan-1-Ol, 2-Methylpropan-1-Ol, and 2-Methylpropan-2-Ol at (298.15, 303.15, 308.15,. J. Chem. Eng. Data 1998, 43, 732–737. https://doi.org/10.1021/je980028e.

(672)	Oswal, S. L.; Desai, H. S. Studies of Viscosity and Excess Molar Volume of Binary Mixtures. 1. Propylamine + 1 -Alkanol Mixtures at 303.15 and 313.15 K. Fluid Phase Equilib. 1998, 149, 359–376. https://doi.org/10.1016/s0378-3812(98)00318-5.

(673)	Aminabhavi, T. M.; Patil, V. B.; Banerjee, K. Thermodynamic Interactions in Binary Mixtures of Ethenylbenzene with Methanol, Ethanol, Butan-1-Ol, Pentan-1-Ol, and Hexan-1-Ol in the Temperature Range 298.15-308.15 K. J. Chem. Eng. Data 1999, 44, 1291–1297. https://doi.org/10.1021/je990120q.

(674)	Haraschta, P.; Heintz, A.; Lehmann, J. K.; Peters, A. Excess Molar Volumes and Viscosities of Binary Mixtures of 2-Methylpuridine with Methanol, Ethanol, Propan-1-Ol, Propan-2-Ol, Butan-2-Ol, and 2-Methylpropan-2-Ol at 298.15 K and Atmospheric Pressure. J. Chem. Eng. Data 1999, 44, 932–935. https://doi.org/10.1021/je990086f.

(675)	Oswal, S. L.; Desai, H. S. Studies of Viscosity and Excess Molar Volume of Binary Mixtures 2. Butylamine + I -Alkanol Mixtures at 303.15 and 313.15 K. Fluid Phase Equilib. 1999, 161, 191–204. https://doi.org/10.1016/s0378-3812(99)00165-x.

(676)	Prasad, T. E. V.; Chandrika, K.; Haritha, M.; Geetha, N. B.; Prasad, D. H. L. DENSITY AND VISCOSITY OF ETHANOL + 0-XYLENE, ETHANOL + m-XYLENE, ETHANOL +p-XYLENE AND METHANOL + O-XYLENE MIXTURES. Phys. Chem. Liq. 1999, 1999, 429–434.

(677)	Henni, A.; Maham, Y.; Tontiwachwuthlkul, P.; Chakma, A.; Mather, A. E. Densities and Viscosities for Binary Mixtures of N-Methyldiethanolamine + Triethylene Glycol Monomethyl Ether from 25 C to 70 C and N-Methyldiethanolamine + Ethanol Mixtures at 40 C. J. Chem. Eng. Data 2000, 45, 247–253. https://doi.org/10.1021/je9902140.

(678)	Nikam, P. S.; Jagdale, B. S.; Sawant, A. B.; Hasan, M. Densities and Viscosities of Binary Mixtures of Toluene with Methanol, Ethanol, Propan-1-Ol, Butan-1-Ol, Pentan-1-Ol, and 2-Methylpropan-2-Ol at (303.15, 308.15, 313.15) K. J. Chem. Eng. Data 2000, 45, 559–563. https://doi.org/10.1021/je990317i.

(679)	Pan, I.-C.; Tang, M.; Chen, Y. P. Densities and Viscosities of Binary Liquid Mixtures of Vinyl Acetate, Diethyl Oxalate, and Dibutyl Phthalate with Normal Alkanols at 303.15 K. J. Chem. Eng. Data 2000, 45, 1012–1015. https://doi.org/10.1021/je0001328.

(680)	Tu, C.-H.; Liu, C.-Y.; Wang, W.-F.; Chou, Y.-T. Volumetric and Viscometric Properties of Binary Mixtures of Aliphatic Alcohols (C1-C4) with Nitroethane from 293.15 K to 313.15 K. J. Chem. Eng. Data 2000, 45, 450–456. https://doi.org/10.1021/je9902589.

(681)	Ku, S.-C.; Peng, I.-H.; Tu, C.-H. Density and Viscosity of Mixtures of Alkoxypropanols with Ethanol at T = (298.15, 308.15, and 318.15) K. J. Chem. Eng. Data 2001, 46, 1392–1398. https://doi.org/10.1021/je010067h.

(682)	Oswal, S. L.; Desai, H. S. Studies of Viscosity and Excess Molar Volume of Binary Mixtures 3. 1-Alkanol + Di-n-Propylamine, and + Di-n-Butylamine Mixtures at 303.15 and 313.15 K. Fluid Phase Equilib. 2001, 186, 81–102. https://doi.org/10.1016/s0378-3812(01)00504-0.

(683)	Tu, C.-H.; Lee, S.-L.; Peng, I.-H. Excess Volumes and Viscosities of Binary Mixtures of Aliphatic Alcohols (C1-C4) with Nitromethane. J. Chem. Eng. Data 2001, 46, 151–155. https://doi.org/10.1021/je0002080.

(684)	Prasad, T. E. V.; Mythili, R.; Nirmala, G. S.; Prasad, D. H. L. Densities and Viscosities of the Binary Mixtures of Chloroethylenes with Some Aliphatic Alcohols. J. Chem. Eng. Data 2002, 47, 68–71. https://doi.org/10.1021/je010141w.

(685)	Belda, R.; Herraez, J. V.; Diez, O. RHEOLOGICAL STUDY AND THERMODYNAMIC ANALYSIS OF THE BINARY SYSTEM (WATER/ETHANOL): INFLUENCE OF CONCENTRATION. Phys. Chem. Liq. 2004, 42, 467–479.

(686)	Chen, H.-W.; Wen, C.-C.; Tu, C.-H. Excess Molar Volumes, Viscosities, and Refractive Indexes for Binary Mixtures of 1-Chlorobutane with Four Alcohols at T = (288.15, 298.15 and 308.15) K. J. Chem. Eng. Data 2004, 49, 347–351. https://doi.org/10.1021/je030226s.

(687)	Rodriquez, A.; Canosa, J.; Dominguez, A.; Tojo, J. Viscosities of Dimethyl Carbonate with Alcohols at Several Temperatures UNIFAC-VISCO Interaction Parameters (?OCOO?/Alcohol). Fluid Phase Equilib. 2004, 216, 167–174. https://doi.org/10.1016/j.fluid.2003.10.014.

(688)	Saleh, M. A.; Akhtar, S.; Begum, S.; Ahmed, M. S.; Begum, S. K. DENSITY AND VISCOSITY OF 1-ALKANOLS. Phys. Chem. Liq. 2004, 42, 615–623.

(689)	Calvar, N.; Dominguez, A.; Tojo, J. Vapor Liquid Equilibria for the Quaternary Reactive System Ethyl Acetate + Ethanol +water + Acetic Acid and Some of the Constituent Binary Systems at 101.3 KPa. Fluid Phase Equilib. 2005, 235, 215–222. https://doi.org/10.1016/j.fluid.2005.07.010.

(690)	Djojoputro, H.; Ismadji, S. Density and Viscosity for a Binary Mixture of Cis-3-Hexenyl Formate, Butyl Acetate, Trans-2-Hexenyl Acetate, and Cis-3-Hexenyl Acetate with Ethanol at Several Temperatures. J. Chem. Eng. Data 2005, 50, 1009–1013. https://doi.org/10.1021/je0500025.

(691)	Djojoputro, H.; Ismadji, S. Density and Viscosity of Binary Mixtures of Ethyl-2-Methylbutyrate and Ethyl Hexanoate with Methanol, Ethanol, and 1-Propanol at (293.15, 303.15, and 313.15) K. J. Chem. Eng. Data 2005, 50, 1343–1347. https://doi.org/10.1021/je0500633.

(692)	Ku, H.-C.; Tu, C.-H. Densities and Viscosities for Binary and Ternary Mixtures of Ethanol, 2-Butanone, and 2,2,4-Trimethylpentane at T = (298.15, 308.15, and 318.15) K. J. Chem. Eng. Data 2005, 50, 608–615. https://doi.org/10.1021/je049655w.

(693)	Wang, C.-C.; Chen, H.-W.; Tu, C.-H. Densities, Viscosities, and Refractive Indices for Binary and Ternary Mixtures of Ethanol, 2-Methylpropan-2-Ol, and 2,2,4-Trimethylpentane. J. Chem. Eng. Data 2005, 50, 1687–1693. https://doi.org/10.1021/je0501639.

(694)	Wankhede, N.; Lande, M. K. K.; Arbad, B. R. R. Excess Molar Volumes and Viscosity Deviations of Binary Mixtures of 2,4,6-Trimethyl-1,3,5-Trioxane + Ethanol, 1-Propanol, and 1-Butanol at (298.15, 303.15, and 308.15) K. J. Chem. Eng. Data 2005, 50, 969–972. https://doi.org/10.1021/je049556i.

(695)	Zeberg-Mikkelsen, C. K.; Baylaucq, A.; Watson, G.; Boned, C. High-Pressure Viscosity Measurements for the Ethanol + Toluene Binary System. Int. J. Thermophys. 2005, 26, 1289–1302. https://doi.org/10.1007/s10765-005-8089-2.

(696)	Alvarez, E.; Gomez-Diaz, D.; La Rubia, M. D.; Navaza, J. M. Densities and Viscosities of Aqueous Ternary Mixtures of 2-(Methylamino)Ethanol and 2-(Ethylamino)Ethanol with Diethanolamine, Triethanolamine, N-Methyldiethanolamine, or 2-Amino-1-Methyl-1-Propanol from 298.15 to 323.15 K. J. Chem. Eng. Data 2006, 51, 955–962. https://doi.org/10.1021/je050463q.

(697)	Arce, A.; Rodriguez, H.; Soto, A. Effect of Anion Fluorination in 1-Ethyl-3-Methylimidazolium as Solvent for the Liquid Extraction of Ethanol from Ethyl Tert-Butyl Ether. Fluid Phase Equilib. 2006, 242, 164–168. https://doi.org/10.1016/j.fluid.2006.01.008.

(698)	Calvar, N.; Gonzalez, B.; Dominguez, A.; Tojo, J. Physical Properties of the Ternary Mixture Ethanol+Water+1-Butyl-3-Methylimidazolium Chloride at 298.15K. J. Solution Chem. 2006, 35, 1217–1225. https://doi.org/10.1007/s10953-006-9073-6.

(699)	Chen, H.-W.; Tu, C.-H. Densities, Viscosities, and Refractive Indices for Binary and Ternary Mixtures of Diisopropyl Ether, Ethanol, and 2,2,4-Trimethylpentane. J. Chem. Eng. Data 2006, 51, 261–267. https://doi.org/10.1021/je050367p.

(700)	Gomez, E.; Gonzalez, B.; Calvar, N.; Tojo, E.; Dominguez, A. Physical Properties of Pure 1-Ethyl-3-Methylimidazolium Ethylsulfate and Its Binary Mixtures with Ethanol and Water at Several Temperatures. J. Chem. Eng. Data 2006, 51, 2096–2102. https://doi.org/10.1021/je060228n.

(701)	Kadam, U. B.; Hiray, A. P.; Sawant, A. B.; Hasan, M. Densities, Viscosities, and Ultrasonic Velocity Studies of Binary Mixtures of Trichloromethane with Methanol, Ethanol, Propan-1-Ol, and Butan-1-Ol at T=(298.15 and 308.15) K. J. Chem. Thermodyn. 2006, 38, 1675–1683. https://doi.org/10.1016/j.jct.2006.03.010.

(702)	Mutalik, V.; Manjeshwar, L. S.; Sairam, M.; Aminabhavi, T. M. Thermodynamic Properties of (Tetradecane + Benzene, + Toluene, + Chlorobenzene, + Bromobenzene, + Anisole) Binary Mixtures at T = (298.15, 303.15, and 308.15) K. J. Chem. Thermodyn. 2006, 38, 1062–1071. https://doi.org/10.1016/j.jct.2005.10.022.

(703)	Roy, M. N.; Sinha, A. Viscous Synergy and Antagonism and Isentropic Compressibility of Ternary Mixtures Containing 1,3-Dioxolane, Water and Monoalkanols at 303.15K. Fluid Phase Equilib. 2006, 243, 133–141. https://doi.org/10.1016/j.fluid.2006.02.023.

(704)	Sheu, Y.-W.; Tu, C.-H. Densities and Viscosities of Binary Mixtures of Isoamyl Acetate, Ethyl Caproate, Ethyl Benzoate, Isoamyl Butyrate, Ethyl Phenylacetate, and Ethyl Caprylate with Ethanol at T = (288.15, 298.15, 308.15, and 318.15) K. J. Chem. Eng. Data 2006, 51, 496–503. https://doi.org/10.1021/je050389b.

(705)	Zeberg-Mikkelsen, C. K.; Watson, G.; Baylaucq, A.; Galliero, G.; Boned, C. Comparative Experimental and Modeling Studies of the Viscosity Behavior of Ethanol +C7 Hydrocarbon Mixtures versus Pressure and Temperature. Fluid Phase Equilib. 2006, 245, 6–19. https://doi.org/10.1016/j.fluid.2006.01.030.

(706)	Arce, A.; Rodriquez, H.; Soto, A. Use of a Green and Cheap Ionic Liquid to Purify Gasoline Octane Boosters. Green Chem. 2007, 9, 247–253. https://doi.org/10.1039/b611139d.

(707)	Calvar, N.; Gonzalez, B.; Gomez, E.; Dominguez, A. Study of the Behaviour of the Azeotropic Mixture Ethanol Water with Imidazolium-Based Ionic Liquids. Fluid Phase Equilib. 2007, 259, 51–56. https://doi.org/10.1016/j.fluid.2007.03.021.

(708)	Calvar, N.; Gomez, E.; Gonzalez, B.; Dominguez, A. Experimental Determination, Correlation, and Prediction of Physical Properties of the Ternary Mixtures Ethanol + Water with 1-Octyl-3-Methylimidazolium Chloride and 1-Ethyl-3-Methylimidazolium Ethylsulfate. J. Chem. Eng. Data 2007, 52, 2529–2535. https://doi.org/10.1021/je700318e.

(709)	Sun, Y.; Ma, P. Densities and Viscosities for Binary Mixture of the Ethyl Crotonate with Ethanol at Different Temperatures. Gaoxiao Huaxue Gongcheng Xuebao 2007, 21, 511.

(710)	Arce, A.; Soto, A.; Ortega, J.; Sabater, G. Viscosities and Volumetric Properties of Binary and Ternary Mixtures of Tris(2-Hydroxyethyl) Methylammonium Methylsulfate + Water + Ethanol at 298.15 K. J. Chem. Eng. Data 2008, 53, 770–775. https://doi.org/10.1021/je700618y.

(711)	Awwad, A. M.; Alsyouri, H. A.; Jbara, K. A. Viscosities and Densities of Binary Mixtures of (N-Acetylmorpholine + Alkanols) from (293.15 to 323.15) K. J. Chem. Eng. Data 2008, 53, 1655–1659. https://doi.org/10.1021/je8003194.

(712)	Calvar, N.; Gonzalez, B.; Gomez, E.; Dominguez, A. Vapor Liquid Equilibria for the Ternary System Ethanol + Water + 1-Ethyl-3-Methylimidazolium Ethylsulfate and the Corresponding Binary Systems Containing the Ionic Liquid at 101.3 KPa. J. Chem. Eng. Data 2008, 53, 820–825. https://doi.org/10.1021/je700710d.

(713)	Gomez, E.; Gonzalez, B.; Calvar, N.; Dominguez, A. Excess Molar Properties of Ternary System (Ethanol + Water + 1,3-Dimethylimidazolium Methyl Sulfate) and Its Binary Mixtures at Several Temperatures. J. Chem. Thermodyn. 2008, 40, 1208–1216. https://doi.org/10.1016/j.jct.2008.04.006.

(714)	Gonzalez, B.; Calvar, N.; Gomez, E.; Macedo, E. A.; Dominguez, A. Synthesis and Physical Properties of 1-Ethyl 3-Methylpyridinium Ethylsulfate and Its Binary Mixtures with Ethanol and Water at Several Temperatures. J. Chem. Eng. Data 2008, 53, 1824–1828. https://doi.org/10.1021/je800159w.

(715)	Rehman, H.; Ansari, M. S. Density, Viscosity, and Electrical Conductivity Measurements on the Ternary System H2O + C2H5OH + LiCl over the Entire Ranges of Solvent Composition and LiCl Solubility from (-5 to +50) Deg C. J. Chem. Eng. Data 2008, 53, 2072–2088. https://doi.org/10.1021/je800150h.

(716)	Yang, C.; Sun, Y.; He, Y.; Ma, P. Volumetric Properties and Viscosities of Binary Mixtures of N,N-Dimethylformamide with Methanol and Ethanol in the Temperature Range (293.15 to 333.15) K. J. Chem. Eng. Data 2008, 53, 293–297. https://doi.org/10.1021/je700430g.

(717)	Andreatta, A. E.; Arce, A.; Rodil, E.; Soto, A. Physical Properties of Binary and Ternary Mixtures of Ethyl Acetate, Ethanol, and 1-Octyl-3-Methyl-Imidazolium Bis(Trifluoromethylsulfonyl)Imide at 298.15 K. J. Chem. Eng. Data 2009, 54, 1022. https://doi.org/10.1021/je800899w.

(718)	Domanska, U.; Laskowska, M. Temperature and Composition Dependence of the Density and Viscosity of Binary Mixtures of (1-Butyl-3-Methylimidazolium Thiocyanate + 1-Alcohols). J. Chem. Eng. Data 2009, 54, 2113–2119. https://doi.org/10.1021/je8008254.

(719)	Gonzalez, B.; Calvar, N.; Gomez, E.; Dominguez, I.; Dominguez, A. Synthesis and Physical Properties of 1-Ethylpyridinium Ethylsulfate and Its Binary Mixtures with Ethanol and 1-Propanol at Several Temperatures. J. Chem. Eng. Data 2009, 54, 1353–1358. https://doi.org/10.1021/je800981d.

(720)	Mokhtarani, B.; Sharifi, A.; Mortaheb, H. R.; Mirzaei, M.; Mafi, M.; Sadeghian, F. Density and Viscosity of 1-Butyl-3-Methylimidazolium Nitrate with Ethanol, 1-Propanol, or 1-Butanol at Several Temperatures. J. Chem. Thermodyn. 2009, 41, 1432–1438. https://doi.org/10.1016/j.jct.2009.06.023.

(721)	Roy, M. N.; Banerjee, A.; Choudhury, A. Rheological, Thermodynamic and Ultrasonic Study of Binary Mixtures Containing 2-Methoxyethanol and Some Alkan-1-Ol. Phys. Chem. Liq. 2009, 47, 412–436. https://doi.org/10.1080/00319100802087183.

(722)	Tsierkezos, N. G.; Molinou, I. E. An Investigation on Ion Association and Ion Solvation of Potassium Acetate in Alcohol Solutions. Phys. Chem. Liq. 2009, 47, 505-(-514). https://doi.org/10.1080/00319100802307672.

(723)	Wang, Y.; Lian, H.-L.; Luo, T.-L.; Liu, G.-J. Densities and Viscosities of (1,6-Hexanediamine + Ethanol) and (1,6-Hexanediamine + Ethanol + Water) Mixtures at T ) (303.15 to 328.15) K. J. Chem. Eng. Data 2009, 54, 1158–1162. https://doi.org/10.1021/je800942u.

(724)	Zarei, H. A.; Mirhidari, N.; Zangeneh, Z. Densities, Excess Molar Volumes, Viscosity, and Refractive Indices of Binary and Ternary Liquid Mixtures of Methanol (1) + Ethanol (2) + 1,2-Propanediol (3) at P = 81.5 KPa. J. Chem. Eng. Data 2009, 54, 847–854. https://doi.org/10.1021/je800633a.

(725)	Andreatta, A. E.; Arce, A.; Rodil, E.; Soto, A. Physico-Chemical Properties of Binary and Ternary Mixtures of Ethyl Acetate + Ethanol + 1-Butyl-3-Methyl-Imidazolium Bis(Trifluoromethylsulfonyl)Imide at 298.15 K and Atmospheric Pressure. J. Solution Chem. 2010, 39, 371–383.

(726)	Anouti, M.; Jacquemin, J.; Lemordant, D. Volumetric Properties, Viscosities, and Isobaric Heat Capacities of Imidazolium Octanoate Protic Ionic Liquid in Molecular Solvents. J. Chem. Eng. Data 2010, 55, 5719–5728. https://doi.org/10.1021/je100671v.

(727)	Bhattacharjee, A.; Roy, M. N. Study on Molecular Interactions by Antagonism, Synergy and Excess Functions of Ternary Mixtures of Tetrahydrofuran with Dimethylsulphoxide and Monoalkanols. Phys. Chem. Liq. 2010, 48, 618–637. https://doi.org/10.1080/00319100903350167.

(728)	Calvar, N.; Gomez, E.; Gonzalez, B.; Dominguez, A. Experimental Vapor-Liquid Equilibria for the Ternary System Ethanol + Water + 1-Ethyl-3-Methylpyridinium Ethylsulfate and the Corresponding Binary Systems at 101.3 KPa: Study of the Effect of the Cation. J. Chem. Eng. Data 2010, 55, 2786–2791. https://doi.org/10.1021/je900998f.

(729)	Goncalves, F. A. M. M.; Trindade, A. R.; Costa, C. S. M. F.; Bernardo, J. C. S.; Johnson, I.; Fonseca, I. M. A.; Ferreira, A. G. M. PVT, Viscosity, and Surface Tension of Ethanol: New Measurements and Literature Data Evaluation. J. Chem. Thermodyn. 2010, 42, 1039–1049. https://doi.org/10.1016/j.jct.2010.03.022.

(730)	Hou, C.; Jiang, Z.; Ren, B. Density and Viscosity of Clopidogrel Hydrogen Sulfate + Methanol and Clopidogrel Hydrogen Sulfate + Ethanol from (278.15 to 313.15) K. J. Chem. Eng. Data 2010, 55, 4943–4945. https://doi.org/10.1021/je1000796.

(731)	Rilo, E.; Vila, J.; Garcia, M.; Varela, L. M.; Cabeza, O. Viscosity and Electrical Conductivity of Binary Mixtures of CnMIM-BF4 with Ethanol at 288 K, 298 K, 308 K, and 318 K. J. Chem. Eng. Data 2010, 55, 5156–5163. https://doi.org/10.1021/je100687x.

(732)	Garcia-Abuin, A.; Gomez-Diaz, D.; La Rubia, M. D.; Navaza, J. M. Density, Speed of Sound, Viscosity, Refractive Index, and Excess Volume of N-Methyl-2-Pyrrolidone + Ethanol (or Water or Ethanolamine) from T = (293.15 to 323.15) K. J. Chem. Eng. Data 2011, 56, 646–651. https://doi.org/10.1021/je100967k.

(733)	Hasan, M.; Hiray, A. P.; Kadam, U. B.; Shirude, D. F.; Kurhe, K. J.; Sawant, A. B. Densities, Viscosities, Speeds of Sound, FT-IR and 1H-NMR Studies of Binary Mixtures of n-Butyl Acetate with Ethanol, Propan-1-Ol, Butan-1-Ol and Pentan-1-Ol at 298.15, 303.15, 308.15 and 313.15 K. J. Solution Chem. 2011, 40, 415–429.

(734)	Kurnia, K. A.; Mutalib, M. I. A. Densities and Viscosities of Binary Mixture of the Ionic Liquid Bis(2-Hydroxyethyl)Ammonium Propionate with Methanol, Ethanol, and 1-Propanol at T = (293.15, 303.15, 313.15, and 323.15) K and at P = 0.1 MPa. J. Chem. Eng. Data 2011, 56, 79–83. https://doi.org/10.1021/je100848r.

(735)	Liu, J.; Zhu, C.; Ma, Y. Densities and Viscosities of Binary Solutions of Benzene-1,3-Diol + Water, Ethanol, Propan-1-Ol, and Butan-1-Ol at T = (293.15 to 333.15) K. J. Chem. Eng. Data 2011, 56, 2095–2099. https://doi.org/10.1021/je101173w.

(736)	Trenzado, J. L.; Romano, E.; Segade, L.; Caro, M. N.; Gonzalez, E.; Galvan, S. Densities and Viscosities of Four Binary Diethyl Carbonate + 1-Alcohol Systems from (288.15 to 313.15) K. J. Chem. Eng. Data 2011, 56, 2841–2848. https://doi.org/10.1021/je1013476.

(737)	Blanco, A.; Garcia-Abuin, A.; Gomez-Diaz, D.; Navaza, J. M. Density, Speed of Sound, Viscosity, Refractive Index, and Excess Volume of N-Methyl-2-Pyrrolidone (NMP) + Water + Ethanol from T = (293.15 to 323.15) K. J. Chem. Eng. Data 2012, 57, 1009–1014. https://doi.org/10.1021/je201152j.

(738)	Chen, K.-D.; Lin, Y.-F.; Tu, C.-H. Densities, Viscosities, Refractive Indexes, and Surface Tensions for Mixtures of Ethanol, Benzyl Acetate, and Benzyl Alcohol. J. Chem. Eng. Data 2012, 57, 1118–1127. https://doi.org/10.1021/je201009c.

(739)	Garcia-Mardones, M.; Gascon, I.; Lopez, M. C.; Royo, F. M.; Lafuente, C. Viscosimetric Study of Binary Mixtures Containing Pyridinium-Based Ionic Liquids and Alkanols. J. Chem. Eng. Data 2012, 57, 3549–3556. https://doi.org/10.1021/je300557g.

(740)	Gong, Y. -h.; Shen, C.; Lu, Y.-Z.; Meng, H.; Li, C.-X. Viscosity and Density Measurement for Six Binary Mixtures of Water (Methanol or Ethanol) with an Ionic Liquid [BMIM][DMP] or [EMIM][DMP] at Atmospheric Pressure in the Temperature Range of (293.15 to 333.15) K. J. Chem. Eng. Data 2012, 57, 33–39. https://doi.org/10.1021/je200600p.

(741)	Han, S.; Zhu, C.; Ma, Y. Densities and Viscosities of Naphthalen-1-Ol, Naphthalen-2-Ol, and 1-Aminonaphthalene in the Solvents of Ethanol, Propan-1-Ol, and Butan-1-Ol. J. Chem. Eng. Data 2012, 57, 1396–1403. https://doi.org/10.1021/je201222n.

(742)	Quijada, E.; Boogaart, S. v. d.; Lijbers, J. H.; Meindersma, G. W.; Haan, A. B. d. Experimental Densities, Dynamic Viscosities and Surface Tensions of the Ionic Liquids Series 1-Ethyl-3-Methylimizazolium Acetate and Dicyanamide and Their Binary and Ternary Mixtures with Water and Ethanol at T = (298.15 K to 343.15 K). J. Chem. Thermodyn. 2012, 51, 51–58. https://doi.org/10.1016/j.jct.2012.02.027.

(743)	Shao, D.; Lu, X.; Fang, W.; Yongsheng, G.; Xu, L. Densities and Viscosities for Binary Mixtures of the Ionic Liquid N-Ethyl Piperazinium Propionate with n-Alcohols at Several Temperatures. J. Chem. Eng. Data 2012, 57, 937–942. https://doi.org/10.1021/je201116t.

(744)	Sreekanth, K.; Kondaiah, M.; Kumar, D. S.; Rao, D. K. Influence of Temperature on Thermodynamic Properties of Acid Base Liquid Mixtures. J. Therm. Anal. Calorim. 2012, 110, 1341–1352. https://doi.org/10.1007/s10973-011-2131-3.

(745)	Xu, Y. J.; Yao, J.; Wang, C.; Li, H. Density, Viscosity, and Refractive Index Properties for the Binary Mixtures of n-Butylammonium Acetate Ionic Liquid + Alkanols at Several Temperatures. J. Chem. Eng. Data 2012, 57, 298–308. https://doi.org/10.1021/je200707b.

(746)	Yao, H.; Zhang, S.; wang, J.; Zhou, Q.; Dong, H.; Zhang, X. Densities and Viscosities of the Binary Mixtures of 1-Ethyl-3-Methylimidazolium Bis(Trifluoromethylsulfonyl)Imide with N-Methyl-2-Pyrrolidone or Ethanol at T = (293.15 to 323.15) K. J. Chem. Eng. Data 2012, 57, 875–881. https://doi.org/10.1021/je200922s.

(747)	Caro, M. N.; Trenzado, J. L.; Galvan, S.; Romano, E.; Gonzalez, E.; Alcalde, R.; Aparicio, S. Densities and Viscosities of Three Binary Monoglyme + 1-Alcohol Systems from (283.15 to 313.15) K. J. Chem. Eng. Data 2013, 58, 909–914. https://doi.org/10.1021/je301095j.

(748)	Faria, M. A. F.; Martins, R. J.; Cardoso, M. J. E. M.; Barcia, O. E. Density and Viscosity of the Binary Systems Ethanol + Butan-1-Ol, + Pentan-1-Ol, + Heptan-1-Ol, + Octan-1-Ol, Nonan-1-Ol, + Decan-1-Ol at 0.1 MPa and Temperatures from 283.15 K to 313.15 K. J. Chem. Eng. Data 2013, 58, 3405–3419. https://doi.org/10.1021/je400630f.

(749)	Kondaiah, M.; Sreekanth, K.; Kumar, D. S.; Rao, D. K. Volumetric and Viscometric Properties of Propanoic Acid in Equimolar Mixtures of N,N-Dimethyl Formamide + Alkanols at T/K 5 303.15, 313.15, and 323.15. J. Solution Chem. 2013, 42, 494–515. https://doi.org/10.1007/s10953-012-9898-0.

(750)	Xu, Y.; Chen, B.; Qian, W.; Li, H. Properties of Pure N-Butylammonium Nitrate Ionic Liquid and Its Binary Mixtures of with Alcohols at T = (293.15 to 313.15) K. J. Chem. Thermodyn. 2013, 58, 449–459. https://doi.org/10.1016/j.jct.2012.10.008.

(751)	Yang, Q.; Yu, K.; Xing, H.; Su, B.; Bao, Z.; Yang, Y.; Ren, Q. The Effect of Molecular Solvents on the Viscosity, Conductivity and Ionicity of Mixtures Containing Chloride Anion-Based Ionic Liquid. J. Ind. Eng. Chem. (Amsterdam, Neth.) 2013, 19, 1708–1714. https://doi.org/10.1016/j.jiec.2013.02.010.

(752)	Castro, M. C.; Rodriguez, H.; Arce, A.; Soto, A. Mixtures of Ethanol and the Ionic Liquid 1-Ethyl-3- Methylimidazolium Acetate for the Fractionated Solubility of Biopolymers of Lignocellulosic Biomass. Ind. Eng. Chem. Res. 2014, 53, 11850–11861. https://doi.org/10.1021/ie501956x.

(753)	Florido, P. M.; Andrade, I. M. G.; Capellini, M. C.; Carvalho, F. H.; Aracava, K. K.; Koshima, C. C.; Rodrigues, C. E. C.; Goncalves, C. B. Viscosities and Densities of Systems Involved in the Deterpenation of Essential Oils by Liquid-Liquid Extraction: New UNIFAC-VISCO Parameters. J. Chem. Thermodyn. 2014, 72, 152–160. https://doi.org/10.1016/j.jct.2013.11.026.

(754)	Goncalves, D.; Koshima, C. C.; Nakamoto, K. T.; Umeda, T. K.; Aracava, K. K.; Goncalves, C. B.; Rodrigues, C. E. d. C. Deterpenation of Eucalyptus Essential Oil by Liquid + Liquid Extraction: Phase Equilibrium and Physical Properties for Model Systems at T = 298.2 K. J. Chem. Thermodyn. 2014, 69, 66–72. https://doi.org/10.1016/j.jct.2013.09.044.

(755)	Moosavi, M.; Omrani, A.; Rostami, A. A.; Motahari, A. Isobaric, Isothermal Theoretical Investigation and Examination of Different Prediction Equations on Some Physicochemical Properties in PEG Liquid Polymer System. J. Chem. Thermodyn. 2014, 68, 205–215. https://doi.org/10.1016/j.jct.2013.09.006.

(756)	Zhu, C.; Han, S.; Liu, J.; Ma, Y. Densities and Viscosities of Diaminotoluene with Water, Ethanol, Propan-1-Ol, and Butan-1-Ol from (293.15 to 333.15) K. J. Chem. Eng. Data 2014, 59, 880–889. https://doi.org/10.1021/je400968v.

(757)	Cao, Q.; Lu, X.; Wu, X.; Guo, Y.; Xu, L.; Fang, W. Density, Viscosity, and Conductivity of Binary Mixtures of the Ionic Liquid N-(2-Hydroxyethyl)Piperazinium Propionate with Water, Methanol, or Ethanol. J. Chem. Eng. Data 2015, 60, 455–463. https://doi.org/10.1021/je500380x.

(758)	Salinas, R.; Pla-Franco, J.; Lladosa, E.; Monton, J. Density, Speed of Sound, Viscosity, and Excess Properties of Binary Mixtures Formed by Ethanol and Bis(Trifluorosulfonyl)Imide-Based Ionic Liquids. J. Chem. Eng. Data 2015, 60, 525–540. https://doi.org/10.1021/je500594z.

(759)	Reid, R. C.; Belenyessy, L. I. Viscosity of Polar Vapor Mixtures. J. Chem. Eng. Data 1960, 5, 150–151. https://doi.org/10.1021/je60006a007.

(760)	Thausen, E. Uber Die Innere Reibung von Gasgemischen. Ann. Phys. (Leipzig) 1911, 36, 815.

(761)	Trautz, M.; Stauf, F. W. Viscosity, Heat Conductivity and Diffusion in Gaseous Mixtures. III. Viscosity of H2-C3H4 Mixtures. Ann. Phys. (Leipzig) 1929, 2, 737.

(762)	Trautz, M.; Zink, R. Viscosity, Thermal Conductivity, and Diffusion in Mas Mixutres. XII. Gas Viscosity at High Temperatures. Ann. Phys. (Berlin) 1930, 7, 427.

(763)	Comings, E. W.; Mayland, B. J.; Egly, R. S. Viscosity of Gases at High Pressures; 42; Bull.-Ill., Univ., Eng. Exp. Stn.: 1, 1944.

(764)	Golubev, I. F.; Petrov, V. A. Viscosity of Gases and Gaseous Mixtures; 1953.

(765)	Misic, D.; Thodos, G. The Thermal Conductivity of Hydrocarbon Gases at Normal Pressures. AIChE J. 1961, 7, 264.

(766)	Vaschenko, D. M.; Voinov, Y.; Voityuk, B. V.; Dregulyas, E. K.; Kolomiets, A. Y.; Labinov, S. D.; Morozov, A. A.; Neduzhil, I. A.; Provotar, V. P.; Soldatenko, Yu. A.; Storozhenko, E. I.; Khmara, Yu. I. Thermodynamic and Transport Properties of Ethylene and Propylene; 1971.

(767)	Maass, O.; Boomer, E. H. Vapor Densities at Low Pressures and over an Extended Temperature Range: I the Properties of Ethylene Oxide Compared to Oxygen Compounds of Similar Molecular Weight. J. Am. Chem. Soc. 1922, 44, 1709–1728. https://doi.org/10.1021/ja01429a013.

(768)	Senftleben, H. Z. Angew. Phys. 1953, 5, 33.

(769)	Zhou, F.; Pan, Y.; Zhu, B. Ethylene Oxide Synthesis in Three-Phase Slurry Reactor under Pressure V. Determination and Correlation of Physical Property Data of Solvent No. 2. Huadong Huagong Xueyuan Xuebao 2000, 26, 581–583.

(770)	Stephan, K.; Heckenberger, T. Thermal Conductivity and Viscosity Data of Fluid Mixtures DECHEMA Chemistry Data Series, Vol. X, Part 1, 1988

(771)	Maczynska, Z.; Maczynski, A. Floppy Book on Viscosity of Liquids, Version 1991.04.13. 1991.

(772)	Hersh, C. K.; Gaynor, A. J. Symposium on Advanced Propellant Chemistry. 149th National Meeting ACS, Vol. 9 No. 1. AIChE-Meeting 1966, 128–135.

(773)	Wayaku, M. Vapor Pressure–Temperature Equation of Hydrocarbon Compounds. Aromatikkusu 1974, 26, 541–553.

(774)	Elverum, G.; Doescher, R. N. Physical Properties of Liquid Fluorine. J. Chem. Phys. 1952, 20, 1834.

(775)	Kanda, E. Studies on Fluorine at Low Temperatures. VIII. Determination of Molecular Heat, Heat of Fusion of Condensed Fluorine and the Entropy of Fluorine. Bull. Chem. Soc. Jpn. 1937, 12, 511.

(776)	Franck, E. U.; Stoeber, W. Viscosity and Molecular Effect Diameter of Fluorine. Z. Naturforsch. Sec. A 1952, 7, 822–823. https://doi.org/10.1515/zna-1952-1210.

(777)	Rankine, A. O.; Smith, J. O. The Viscosities and Molecular Dimensions of Methane, Sulphuretted Hydrogen and Cyanogen. Philos. Mag. 1921, 42, 615.

(778)	Bhattacharyya, P. K. Viscosity of Binary Polar-Gas Mixtures: CH3Cl - H2S and CH3Cl - SO2. J. Chem. Phys. 1970, 53, 893–895. https://doi.org/10.1063/1.1674154.

(779)	Pal, A. K.; Bhattacharyya, P. K. Viscosity of Binary Polar-Gas Mixtures. J. Chem. Phys. 1969, 51, 828–831. https://doi.org/10.1063/1.1672075.

(780)	Bhattacharyya, P. K.; Ghosi, A. K. Viscosity of Polar - Quadrupolar Gas Mixtures. J. Chem. Phys. 1970, 52, 2719–2723.

(781)	Pal, A. K.; Barua, A. K. Viscosity of Hydrogen Nitrogen and Hydrogen Ammonia Gas Mixtures. J. Chem. Phys. 1967, 47, 216–218. https://doi.org/10.1063/1.1711848.

(782)	Monteil, J. M.; Lazarre, F.; Salvinien, J.; Viallet, P. Viscometer for the Study of Relative Viscosity of H2S. J. Chim. Phys. Phys. Chim. Biol. 1969, 66, 1673–1675. https://doi.org/10.1051/jcp/196966s21673.

(783)	Hennel, J. W.; Krynicki, K. Viscosity of Liquid Hydrogen Sulfide. Acta Phys. Pol. 1959, 19, 523–526.

(784)	Jung, G.; Schmick, H. Der Einfluss Der Molekularen Anziehungskraefte Auf Die Innere Reibung von Gasgemischen. Z. Phys. Chem. Abt. B 1930, 7, 130–147.

(785)	Steele, B. D.; McIntosh, D.; Archibald, E. H. The Vapor Pressures, Densities, Surface Energies and Viscosities of Pure Solvents. Z. Phys. Chem., Stoechiom. Verwandschaftsl. 1906, 55, 129–150.

(786)	Runovskaya, I. V.; Zorin, A. D.; Devyatykh, G. G. Viscosity of Condensed Volatile Inorganic Hydrides of Group III- VI Elements. Zh. Neorg. Khim. 1970, 15, 2581.

(787)	Vogel, E. Reference Viscosities of Gaseous Methane and Hydrogen Sulfide at Low Density in the Temperature Range from (292 to 682) K. J. Chem. Eng. Data 2011, 56, 3265–3272. https://doi.org/10.1021/je200371n.

(788)	Harle, H. On the Viscosities of the Hydrogen Halides. Proc. Roy. Soc. London Ser. A 1922, 100, 429–440. https://doi.org/10.1098/rspa.1922.0008.

(789)	Trautz, M.; Narath, A. Die Innere Reibung von Gasgemischen. Ann. Phys. (Leipzig) 1926, 79, 637.

(790)	Krynicki, K.; Hennel, J. W. VISCOSITY OF LIQUID AMMONIA AND HYDROGEN CHLORIDE. Acta Phys. Pol. 1963, 24, 269–271.

(791)	Zinovieva, K. N. Viscosity of Liquid He-3 in the Interval 0.35 - 3.2 K and He-4 above the Lambda-Point. Zh. Eksp. Teor. Fiz. 1958, 34, 609–615.

(792)	Luker, J. A.; Johnson, C. A. Viscosity of Helium, Oxygen, Helium-Oxygen, Helium-Steam, and Oxygen-Steam Mixtures at High Temperatures and Pressures. J. Chem. Eng. Data 1959, 4, 176–182. https://doi.org/10.1021/je60002a019.

(793)	Kao, J. T.; Kobayashi, R. Viscosity of Helium and Nitrogen and Their Mixtures at Low Temperatures and Elevated Pressures. J. Chem. Phys. 1967, 47, 2836–2849.

(794)	Tsederberg, N. V.; Popov, V. N.; Panchenko, S. S. Experimental Study of the Viscosity of Helium at Temperatures from 80 to 273 K and at Pressures to 40 MPa. Teploenergetika 1974, 78–79.

(795)	Tanaka, Y.; Nakajima, M.; Kubota, H.; Makita, T. Viscosity of SF6 + N2 and SF6 + He Gaseous Mixtures. J. Chem. Eng. Jpn. 1980, 13, 155–159.

(796)	Reynes, E. G.; Thodos, G. Viscosity of Helium, Neon, and Nitrogen in the Dense Gaseous Region. J. Chem. Eng. Data 1966, 11, 137–140. https://doi.org/10.1021/je60029a001.

(797)	Rudenko, N. S.; Karnus, A. I. Viscosity of He4 in the Temperature Range from 14 to 31 K and at Pressures to 100 Atm. Ukr. Fiz. Zh. 1968, 13, 1334–1337.

(798)	Seibt, D.; Herrmann, S.; Vogel, E.; Bich, E.; Hassel, E. Simultaneous Measurements on Helium and Nitrogen with a Newly Designed Viscometer-Densimeter over a Wide Range of Temperature and Pressure. J. Chem. Eng. Data 2009, 54, 2626–2637. https://doi.org/10.1021/je900131q.

(799)	Albright, L. F.; Lohrenz, J. Viscosity of Liquids. AIChE J. 1956, 2, 290–295.

(800)	Ledneva, T. M. Bulk Viscosity and Its Temperature Dependence for Some Organc Liquids. Vestn. Mosk. Univ. 1956, 2, 49–60.

(801)	Abdulagatov, I. M.; Rasulov, S. M. Viscosity of Binary N-Pentane (0.5)-n-Heptane (0.5) System and Its Pure Components near the Liquid-Gas Critical Points. Teplofiz. Vys. Temp. 1992, 30, 501–508.

(802)	Abdulagatov, I. M.; Rasulov, S. M. Anomaly of Viscosity of the N-Heptane near the Liquid-Gas Critical Point. Zh. Fiz. Khim. 1996, 70, 2187–2190.

(803)	Smyth, C. P.; Stoops, W. N. The Dielectric Polarization of Liquids III. The Polarization of the Isomers of Heptane. J. Am. Chem. Soc. 1928, 50, 1883–1890. https://doi.org/10.1021/ja01394a008.

(804)	Fawcett, F. S. Boiling Points of Three Isomeric Heptanes. Ind. Eng. Chem. 1946, 38, 338–340. https://doi.org/10.1021/ie50435a026.

(805)	Giller, E. B.; Drickamer, H. G. Viscosity of Normal Paraffins near the Freezing Point. Ind. Eng. Chem. 1949, 41, 2067.

(806)	Koshkin, N. I.; Nozdrev, V. F. Study of Ultrasound Attenuation for a Series of Saturated Hydrocarbons by the Pulse Method. Dokl. Akad. Nauk 1953, 92, 793–796.

(807)	Zhigunov, I. S.; Ampelogova, N. I. Some Properties of Nitrogen-Containing Solvents and Their Binary Mixtures II. Properties of n-Butylamine + Water System. Fiz. Khim. Rastvorov 1972, 181.

(808)	Badalov, Y. A.; Naziev, Y. M.; Guseinov, S. O. Viscosity of Liquids of Binary Mixtures of N-Heptane and Benzene at High Pressures. Izv. Vyssh. Uchebn. Zaved., Neft Gaz 1975, 18, 67–70.

(809)	Kessel’man, P. M.; Porichanskii, E. G.; Karbanov, E. M. Study of Dynamic Viscosity Coefficients for Freons in the Wide Temperature and Pressure Interval. Kholod. Tekh. Tekhnol. 1976, 22, 48–50.

(810)	Gunchuk, B. V.; Karbanov, E. M.; Lapardin, N. I.; Zakharzhevskiy, V. Y. Investigation of the Viscosity of Some Freons Along the Saturated Line. In Teplofiz. Svoistva Veshchestv. Mater.; No. 11, Rabinovich, V. A., Ed., Standards Publ.: Moscow, 1977; pp 39–46.

(811)	Rappon, M.; Kaukinen, J. A. MOLECULAR ASSOCIATION OF PENTANOLS IN N-HEPTANE II : VISCOSITIES AS A FUNCTION OF TEMPERATURE COVERING LOW CONCENTRATION RANGE. J. Mol. Liq. 1988, 38, 107–133. https://doi.org/10.1016/0167-7322(88)80011-4.

(812)	Kanti, M.; Lagourette, B.; Alliez, J.; Boned, C. Viscosity of Binary Heptane-Nonylbenzene as a Function of Pressure and Temperature: Application of Flory’s Theory. Fluid Phase Equilib. 1991, 65, 291–304. https://doi.org/10.1016/0378-3812(91)87031-4.

(813)	Assael, M. J.; Oliveira, C.; Papadaki, M.; Wakeham, W. A. Vibrating-Wire Viscometers for Liquids at High Pressures. Int. J. Thermophys. 1992, 13, 593–615. https://doi.org/10.1007/bf00501943.

(814)	Blanco, A. M.; Ortega, J.; Garcia, B.; Leal, J. M. Studies on Densities and Viscosities of Binary Mixtures of Alkyl Benzoates in N-Heptane. Thermochim. Acta 1993, 222, 127–136. https://doi.org/10.1016/0040-6031(93)80546-m.

(815)	Aminabhavi, T. M.; Aralaguppi, M. I.; Gopalakrishna, B.; Khinnavar, R. S. Densities, Shear Viscosities, Refractive Indices, and Speeds of Sound of Bis(2-Methoxyethyl) Ether with Hexane, Heptane, Octane, and 2,2,4- Trimethylpentane in the Temperature Interval 298.15-318.15. J. Chem. Eng. Data 1994, 39, 522–528. https://doi.org/10.1021/je00015a028.

(816)	Sastry, N. V.; Raj, M. M. Densities, Speeds of Sound, Viscosities, Dielectric Constants, and Refractive Indices for 1-Heptanol + Hexane and +Heptane at 303.15 and 313.1 5 K. J. Chem. Eng. Data 1996, 41, 612–618. https://doi.org/10.1021/je950172p.

(817)	Sastry, N. V.; Valand, M. K. Densities, Speeds of Sound, Viscosities, and Relative Permittivities for 1- Propanol + and 1-Butanol + Heptane at 298.15 K and 308.15 K. J. Chem. Eng. Data 1996, 41, 1421–1425. https://doi.org/10.1021/je960135d.

(818)	Will, S.; Leipertz, A. Viscosity of Liquid N-Heptane by Dynamic Light Scattering. Int. J. Thermophys. 1997, 18, 1339–1354. https://doi.org/10.1007/bf02575338.

(819)	Jimenez, E.; Franjo, C.; Segade, L.; Legido, J. L.; Andrade, M. I. P. Viscosities and Densities for the 1-Propanol + n -Heptane System at Several Temperatures. J. Solution Chem. 1998, 27, 569–579.

(820)	Matos, J. S.; Trenzado, J. L.; Gonzalez, E.; Alcalde, R. Volumetric Properties and Viscosities of the Methyl Butanoate + N-Heptane + n-Octane Ternary System and Its Binary Constituents in the Temperature Range from 283.15 to 313.15 K. Fluid Phase Equilib. 2001, 186, 207–234. https://doi.org/10.1016/s0378-3812(01)00511-8.

(821)	Ma, P.; Jin, H.; Li, N.; Yang, C. Densities and Viscosities for Binary Mixture of the Styrene with N-Octane at Different Temperatures. Tianjin Daxue Xuebao 2005, 38, 5;356.

(822)	Pensado, A. S.; Comunas, M. J. P.; Lugo, L.; Fernandez, J. Experimental Dynamic Viscosities of 2,3-Dimethylpentane up to 60 MPa and from (303.15 to 353.15) K Using a Rolling-Ball Viscometer. J. Chem. Eng. Data 2005, 50, 849–855. https://doi.org/10.1021/je049662k.

(823)	Abdulagatov, I. M.; Zeinalova, A. B.; Azizov, N. D. Viscosity of Aqueous Electrolyte Solutions at High Temperatures and High Pressures. Viscosity B-Coefficient. Sodium Iodide. J. Chem. Eng. Data 2006, 51, 1645–1659. https://doi.org/10.1021/je060124c.

(824)	Landaverde-Cortes, D. C.; Estrada-Baltazar, A.; Iglesias-Silva, G. A.; Hall, K. R. Densities and Viscosities of MTBE + Heptane or Octane at p ) 0.1 MPa from (273.15 to 363.15) K. J. Chem. Eng. Data 2007, 52, 1226–1232. https://doi.org/10.1021/je600554h.

(825)	Wang, Z. F.; Wang, L.-S.; Fan, T.-B. Densities and Viscosities of Ternary Mixtures of Heptane, Octane, Nonane, and Hexyl Benzene from 293.15 K to 313.15 K. J. Chem. Eng. Data 2007, 52, 1866–1871. https://doi.org/10.1021/je700202h.

(826)	El-Sayed, H. E. M.; Asfour, A.-F. A. Viscometric and Volumetric Properties of 10 Regular Binary Systems at 308.15 K and 313.15 K. Int. J. Thermophys. 2009, 30, 1773–1790. https://doi.org/10.1007/s10765-009-0667-2.

(827)	Sagdeev, D.; Fomina, M.; Mukhamedzyanov, G.; Abdulagatov, I. M. Experimental Study of the Density and Viscosity of N-Heptane at Temperatures from 298 K to 470 K and Pressure up to 245 MPa. Int. J. Thermophys. 2013, 34, 1–33. https://doi.org/10.1007/s10765-012-1373-z.

(828)	Luning Prak, D.; Cowart, J.; Trulove, P. Density, Viscosity, Speed of Sound, Bulk Modulus, and Surface Tension of Binary Mixtures of n-Heptane + 2,2,4-Trimethylpentane at (293.15 to 338.15) K and 0.1 MPa. J. Chem. Eng. Data 2014, 59, 3842–3851. https://doi.org/10.1021/je5007532.

(829)	Melaven, R. M.; Mack, E. Jr. The Collision Areas and Shapes of Carbon Chain Molecules in the Gaseous State: Normal-Heptane, Normal-Octane, Normal-Nonane. J. Am. Chem. Soc. 1932, 54, 888–904. https://doi.org/10.1021/ja01342a007.

(830)	Agaev, N. A.; Golubev, I. F. Viscosity of N-Pentane in the Liquid and Gaseous State at High Pressures and Different Temperatures. Gaz. Prom. 1963, 45–50.

(831)	Carmichael, L. T.; Reamer, H. H.; Sage, B. H. Thermal Conductivity of Fluids. Methane. J. Chem. Eng. Data 1966, 11, 52–57. https://doi.org/10.1021/je60028a014.

(832)	Diaz Pena, M.; Cheda, J. A. R. Viscosidad de Hidrocarburos. II. n-Heptano y Sus Isomeros. An. Quim. 1974, 70, 107.

(833)	Vogel, E.; Holdt, B. Temperature and Initial-Density Dependence of the Vapour Phase Viscosity of n-Alkanes (n = 5- 7). High Temp. - High Pressures 1991, 23, 473–483.

(834)	Abdulagatov, I. M.; Rasulov, S. M. Viscosity of N-Pentane, N-Heptane and Their Mixtures within the Temperature Range from 298K up to Critical Points at the Saturation Vapor Pressure. Ber. Bunsen-Ges. Phys. Chem. 1996, 100, 148–154.

(835)	Andrade, E. N. d. C.; Rotherham, L. On the Determination of Viscosity by the Oscillation of a Vessel Enclosing a Fluid: Part 2. Proc. Phys. Soc. 1936, 48, 261–266. https://doi.org/10.1088/0959-5309/48/2/303.

(836)	Grunberg, L. Viscosity of Regular Solutions Systems Involving Carbon Tetrachloride, Benzene, and Cyclohexane. Trans. Faraday Soc. 1954, 50, 1293.

(837)	Parisot, P. E.; Johnson, E. F. Liquid Viscosity above the Normal Boiling Point. J. Chem. Eng. Data 1961, 6, 263–267. https://doi.org/10.1021/je60010a024.

(838)	Naziev, Y. M.; Guseinov, S. O.; Akhmedov, A. K. Experimental Study of the Viscosity of Some Hydrocarbons at Various Pressures and Temperatures. Izv. Vyssh. Uchebn. Zaved., Neft Gaz 1972, 15, 65–68.

(839)	Strumpf, H. J.; Collings, A. F.; Pings, C. J. Viscosity of Xenon and Ethane in the Critical Region. J. Chem. Phys. 1974, 60, 3109–3123.

(840)	Bulanov, N. V.; Skripov, V. P. Measurements of the Viscosity of Overheated Liquids at High Pressures. Inzh.-Fiz. Zh. 1975, 29, 1074–1078.

(841)	Craubner, H. Densitometer for Absolute Meas. of the Temperature Dependence of Den- Sity, Partial Volumes, and Thermal Expansivity of Solids and Liquids. Rev. Sci. Instrum. 1986, 57, 2817.

(842)	Hamelin, J.; Mehl, J. B.; Moldover, M. R. Resonators for Accurate Dielectric Measurements in Conducting Liquids. Rev. Sci. Instrum. 1998, 69 (1), 255–260. https://doi.org/10.1063/1.1148505.

(843)	Grigor’ev, B. A.; Keramidi, A. S.; Rodchenko, S. I.; Grachev, V. K. Viscosity of Normal Hexane at Low Temperature. Izv. Vyssh. Uchebn. Zaved., Neft Gaz 1988, 31, 53.

(844)	Oswal, S. L.; Dave, J. P. Viscosities of Nonelectrolyte Liquid Mixtures. II. Binary Mixtures of n- Hexane with Alkanoates and Bromoalkanoates. Int. J. Thermophys. 1992, 13, 943–955. https://doi.org/10.1007/bf01141208.

(845)	Franjo, C.; Menaut, C. P.; Jimenez Cuesta, E.; Legido, J. L.; Paz Andrade, M. I. Viscosities and Densities of Octane + Butan-1-Ol, Hexan-1-Ol, and Octan-1- Ol at 298.15 K. J. Chem. Eng. Data 1995, 40, 992–994. https://doi.org/10.1021/je00020a058.

(846)	Dominguez, M.; Pardo, J.; Lopez, M. C.; Royo, F. M.; Urieta, J. S. Viscosities of the Ternary Mixture (1-Butanol + n-Hexane + 1-Butylamine) at the Temperatures 298.15 and 313.15 K. Fluid Phase Equilib. 1996, 124, 147–159. https://doi.org/10.1016/s0378-3812(96)03070-1.

(847)	Dominguez, M.; Santafe, J.; Lopez, M. C.; Royo, F. M.; Urieta, J. S. Viscosities of the Ternary Mixture (1-Butanol + n-Hexane + 1-Chiorobutane) at 298.15 K and 313.15 K. Fluid Phase Equilib. 1998, 152, 133–148. https://doi.org/10.1016/s0378-3812(98)00377-x.

(848)	Dominguez, M.; Pardo, J. I.; Gascon, I.; Royo, F. M.; Urieta, J. S. Viscosities of the Ternary Mixture (2-Butanol + n-Hexane + 1-Butylamine) at 298.15 and 313.15 K. Fluid Phase Equilib. 2000, 169, 277–292. https://doi.org/10.1016/s0378-3812(00)00332-0.

(849)	Gascon, I.; Pardo, J.; Santafe, J.; Dominguez, M.; Urieta, J. S. Experimental and Predicted Viscosities of the Binary System (n-Hexane + 1,3-Dioxolane) and for the Ternary System (n-Hexane + 1,2-Dioxolane + 1-Butanol) at 298.15 and 313.15 K. Fluid Phase Equilib. 2001, 180, 211–220. https://doi.org/10.1016/s0378-3812(01)00348-x.

(850)	Comelli, F.; Ottani, S.; Francesconi, R.; Castellari, C. Densities, Viscosities, and Refractive Indices of Binary Mixtures Containing n-Hexane + Components of Pine Resins and Essential Oils at 298.15 K. J. Chem. Eng. Data 2002, 47, 93–97. https://doi.org/10.1021/je010216w.

(851)	Bolotnikov, M. F.; Neruchev, Yu. A. Viscosities and Densities of Binary Mixtures of Hexane with 1-Chlorohexane between 293.15 K and 333.15 K. J. Chem. Eng. Data 2003, 48, 739–741. https://doi.org/10.1021/je034002l.

(852)	Dominguez, M.; Langa, E.; Mainar, A. M.; Santafe, J.; Urieta, J. S. Measurements, Correlations, and Predictions of Viscosities for the Ternary Mixture (2-Butanol + Hexane + 1-Chlorobutane) at 298.15 K and 313.15 K. J. Chem. Eng. Data 2003, 48, 302–307. https://doi.org/10.1021/je020114l.

(853)	Gascon, I.; Villares, A.; Haro, M.; Martin, S.; Artigas, H. Experimental and Predicted Viscosities of the Ternary Mixture (Hexane + 1,3-Dioxolane + 2-Butanol) at 298.15 and 313.15 K. J. Chem. Eng. Data 2005, 50, 722–726. https://doi.org/10.1021/je049576k.

(854)	Modarress, H.; Mohsen-Nia, M. Experimental and Theoretical Studies of Viscosities of Ternary Mixture [2-PropanolQethyl AcetateQn-Hexane] and Its Binary Constituents at 298.15, 308.15 and 313.15K. Phys. Chem. Liq. 2006, 44, 67–76.

(855)	Carrasco, A.; Perez-Navarro, M.; Gascon, I.; Lopez, M. C.; Lafuente, C. Densities and Viscosities of the Ternary Mixtures 2-Methyl-1-Propanol (or 2-Methyl-2-Propanol) + N-Hexane + 1-Chlorobutane at 298.15 K. J. Chem. Eng. Data 2008, 53, 1223–1227. https://doi.org/10.1021/je800048f.

(856)	McCoubrey, J. C.; McCrea, J. N.; Ubbelohde, A. R. The Configuration of Flexible Polymethylene Molecules in the Gas Phase. J. Chem. Soc. 1951, 1961.

(857)	Schuler; Michel. Z. Naturforsch., A: Astrophys. Phys. Phys. Chem. 1956, 11, 403.

(858)	Grachev, V. K.; Grigor’ev, B. A.; Keramidi, A. S. Study of the Viscosity of Normal Hexane at High Teperatures. Izv. Vyssh. Uchebn. Zaved., Neft Gaz 1989, 41–44.

(859)	van Itterbeek, A.; van Paemel, O. Measurements on the Viscosity of Gases for Low Pressures at Room Temperature and at Low Temperatures. Physica Amsterdam 1940, 7, 273–283. https://doi.org/10.1016/S0031-8914(40)90116-1.

(860)	Diller, D. E. Measurements of the Viscosity of Parahydrogen. J. Chem. Phys. 1965, 42, 2089.

(861)	Breitenbach, P. On the Viscosity of Gases and Their Change with Temperature (in German). Ann. Phys. (Leipzig) 1901, 5, 166–169.

(862)	Verschaffelt, J. E.; Nicaise, C. The Viscosity of Liquefied Gases. IX. Preliminary Determination of the Viscosity of Liquid Hydrogen. Comm. Phys. Lab. Univ. Leiden 1917, 66–71.

(863)	Yen, K. An Absolute Determination of the Coefficients of Viscosity of Hydrogen, Nitrogen and Oxygen. Philos. Mag. Ser. 6 1919, 38, 582.

(864)	Klein, S. A.; McLinden, M. O.; Laesecke, A. An Improved Extended Corresponding States Method for Estimation of Viscosity of Pure Refrigerants and Mixtures. Int. J. Refrig. 1997, 20 (3), 208–217. https://doi.org/10.1016/S0140-7007(96)00073-4.

(865)	Trautz, M.; Baumann. Viscosity, Thermal COnductivey and Diffusion in Gas Mixtures II. The Viscosity of H2-N2 and H2-CO Mixtures. Ann. Phys. (Leipzig) 1929, 2, 733.

(866)	Trautz, M.; Ludewigs, W. The Viscosity, Thermal Conductivity and Diffusion in Gas Mixtures. VI. Viscosity Determinations on Pure Gases by Direct Measurement and by the Same Means on Their Mixtures (in German). Ann. Phys. (Leipzig) 1929, 3(5), 409–428.

(867)	Trautz, M.; Kurtz, F. Viscosity, Thermal Conductivity, and Diffusion in Gas Mixtures. Ann. Phys. (Berlin) 1931, 9, 981.

(868)	Sutherland, B. P.; Maass, O. Measurement of the Viscosity of Gases over a Large Temperature Range. Can. J. Res. 1932, 6, 428.

(869)	Trautz, M.; Zimmermann, H. Viscosity, Thermal Conductivity and Diffusion in Gas Mixtures. XXX. Viscosity at Low Temperatures of Hydrogen, Helium and Neon and Their Binary Mixtures Down to 90 Deg Abs. Ann. Phys. (Berlin) 1935, 22, 189–194.

(870)	Johns, H. E. The Viscosity of Liquid Hydrogen. Can. J. Res. A 1939, 17, 221–226.

(871)	Johnston, H. L.; McCloskey, K. E. Viscosities of Several Common Gases Between 90 K and Room Temperature. J. Phys. Chem. 1940, 44, 1038–1058.

(872)	Buddenberg, J. W.; Wilke, C. R. Viscosities of Some Mixed Gases. J. Phys. Colloid Chem. 1951, 55, 1491–1498.

(873)	Kuss, E. Viscosity of Compressed Gases. Z. Angew. Phys. 1952, 4, 203.

(874)	Kompaneets, V. Y. Experimental Investigation of the Viscosity of Gases and Gaseous Mixtures at High Temperatures. Sb. Nauchn. Rab., Leningr. Inst. Mekhan. Selsk. Khoz. 1953, 113.

(875)	Kestin, J.; Pilarczyk, K. Measurement of the Viscosity of Five Gases at Elevated Pressures by the Oscillating-Disk Method. Trans. Am. Soc. Mech. Eng. 1954, 76, 987–999.

(876)	Rietveld, A., A. O. ,. van Itterbeek. Viscosity of Mixtures of H(2) and HD between 300 and 14 Degrees K. Comm. Phys. Lab. Univ. Leiden 1957, 1–5.

(877)	Kestin, J.; Wang, H. E. The Viscosity of Five Gases: A Re-Evaluation. Trans. Am. Soc. Mech. Eng. 1958, 80, 11.

(878)	Golubev, I. F.; Shepeleva, R. I. Viscosity of Hydrogen at Low Temperatures and High Pressures (in Russian). Gaz. Prom. 1966, 11, 54–58.

(879)	Gracki, J. A.; Flynn, G. P.; Ross, J.-J. Viscosity of Nitrogen, Helium, Hydrogen, and Argon from -100 to 25 C up 150 - 200 Atm. J. Chem. Phys. 1969, 51, 3856.

(880)	Guevara, F. A.; McInter, B. B.; Ottesen, D. High-Temperature Viscosity Ratios for Hydrogen, Helium, Argon, and Nitrogen. Phys. Fluids 1969, 12, 2493.

(881)	Chuang, S.-. y; Chappelear, P. S.; Kobayashi, R. Viscosity of Methane, Hydrogen, and Four Mixtures of Methane and Hydrogen from -100 C to 0 C at High Pressures. J. Chem. Eng. Data 1976, 21, 403–411. https://doi.org/10.1021/je60071a010.

(882)	Clifford, A. A.; Kestin, J.; Wakeham, W. A. The Viscosity of Mixtures of Hydrogen with Three Noble Gases. Ber. Bunsen-Ges. Phys. Chem. 1981, 85, 385–388.

(883)	Händel, G.; Kleinrahm, R.; Wagner, W. Measurements of the (Pressure, Density, Temperature) Relation of Methane in the Homogeneous Gas and Liquid Regions in the Temperature Range from 100 K to 260 K and at Pressures up to 8 MPa. J. Chem. Thermodyn. 1992, 24 (7), 685–695. https://doi.org/10.1016/S0021-9614(05)80076-X.

(884)	Nabizadeh, H.; Mayinger, F. Viscosity of Gaseous R404A, R407C, R410A, and R507. Int. J. Thermophys. 1999, 20 (3), 777–790.

(885)	Boyd, J. H. The Viscosity of Compressed Gases. Phys. Rev. 1930, 35, 1284.

(886)	Keesom, W. H.; MacWood, G. E. The Viscosity of Hydrogen Vapour. Comm. Phys. Lab. Univ. Leiden 1938, 1–4.

(887)	Keesom, W. H.; Keesom, P. H. The Viscosity of Hydrogen Vapour. Comm. Phys. Lab. Univ. Leiden 1940, 1–4.

(888)	Tsederberg, N. V.; Popov, V. N.; Andreev, I. I. Experimental Study of the Viscosity of Hydrogen. Teploenergetika 1965, 84–86.

(889)	Yusibani, E.; Nagahama, Y.; Kohno, M.; Takata, Y.; Woodfield, P.; Shinzato, K.; Fujii, M. A Capillary Tube Viscometer Designed for Measurements of Hydrogen Gas Viscosity at High Pressure and High Temperature. Int. J. Thermophys. 2011, 32, 1111–1124. https://doi.org/10.1007/s10765-011-0999-6.

(890)	Garner, J. B.; Adams, L.; Stuchell, R. M. Physical and Chemical Properties of Isobutylene. Pet. Refiner 1945, 24, 99–102.

(891)	Tatevskii, V. M.; Grikina, O. E.; Abramenkov, A. V.; Tkachik, Z. A. Relationship between the Viscosity of Liquids and the Chemical Structure of Their Molecules. Russ. J. Phys. Chem. 1985, 59, 1643–1644.

(892)	Rappenecker, K. On the Viscosity Coefficient of Gases and Their Temperature Dependence. Z. Phys. Chem., Stoechiom. Verwandschaftsl. 1910, 72, 695.

(893)	Bratton, . J., A. C.; Felsing, W. A. A High-Efficiency Method Applicable to Certain Classes of Compounds-. Ind. Eng. Chem. 1936, 28, 424–430.

(894)	Maman, A. Octanes. C. R. Hebd. Seances Acad. Sci. 1938, 207, 1401–1402.

(895)	Johnson, G. C.; Fawcett, F. S. Inter-Polymerization of Isobutane and 2-Methyl-2-Butene Using an Alumina-Silica Catalyst. Compostion of the Hydrogenated Nonanes. J. Am. Chem. Soc. 1946, 68, 1416–1419. https://doi.org/10.1021/ja01212a005.

(896)	Dymond, J. H.; Glen, N. F.; Isdale, J. D. Transport Properties of Nonelectrolyte Liquid Mixtures–VII. Visocity Coeff. for Isooctane and for Equimolar Mixtures of Isooctane + n-Octane and Isooctane + n-Dodecane from 25 to 100 Deg. C at Pr. Int. J. Thermophys. 1985, 6, 233–250. https://doi.org/10.1007/bf00522146.

(897)	Ma, P.; Li, N. Measurement and Correlation of Densities and Viscosities for Correlative Systems of Dimethyl Carbonate. Tianjin Daxue Xuebao 2005, 38, 577.

(898)	Salas, J. A.; Pedrosa, G. C.; Acevedo, I. L.; Arancibia, E. L. Excess Molar Volumes and Excess Viscosities of the 2,2,4-Trimethylpentane (1) + 3-Methyl-1-Butanol (2) and Propan-2-Ol (3) Ternary System at 298.15 K. J. Mol. Liq. 2006, 124, 37–44.

(899)	Ku, H.-C.; Wang, C.-C.; Tu, C.-H. Densities, Viscosities, Refractive Indexes, and Surface Tensions for Binary and Ternary Mixtures of Tetrahydofuran, 2-Propanol, and 2,2,4-Trimethylpentane. J. Chem. Eng. Data 2008, 53, 566–573. https://doi.org/10.1021/je700626v.

(900)	Ku, H.-C.; Wang, C.-C.; Tu, C.-H. Densities, Viscosities, Refractive Indices, and Surface Tensions for the Mixtures of 1,3-Dioxolane + 2-Propanol or + 2,2,4-Trimethylpentane at (288.15, 298.15, and 308.15) K and 1,3-Dioxolane + 2-Propanol + 2,2,4-Trimethylpentane at 298.15 K. J. Chem. Eng. Data 2009, 54, 131–136. https://doi.org/10.1021/je800664z.

(901)	Kao, Y.-C.; Tu, C.-H. Densities, Viscosities, Refractive Indices, and Surface Tensions for Binary and Ternary Mixtures of 2-Propanol, Tetrahydropyran, and 2,2,4-Trimethylpentane. J. Chem. Thermodyn. 2011, 43, 216–226. https://doi.org/10.1016/j.jct.2010.08.019.

(902)	Rafiee, H. R.; Ranjbar, S.; Poursalman, F. Densities and Viscosities of Binary and Ternary Mixtures of Cyclohexanone, 1,4-Dioxane and Isooctane from T = (288.15 to 313.15) K. J. Chem. Thermodyn. 2012, 54, 266–271. https://doi.org/10.1016/j.jct.2012.05.005.

(903)	Yue, L.; Qin, X.; Wu, X.; Xu, L.; Guo, Y.; Fang, W. Excess Molar Volume along with Viscosity, Refractive Index and Relative Permittivity for Binary Mixtures of Exo-Tetrahydrodicyclopentadiene with Four Octane Isomers. J. Chem. Thermodyn. 2015, 81, 26–33. https://doi.org/10.1016/j.jct.2014.09.015.

(904)	Diaz Pena, M.; Cabello, A.; Cheda, J. A. R. Viscosidad Del N-Octano e Isomeros En Fase de Vapor. An. Quim. 1975, 71, 637.

(905)	Lesche, H.; Klemp, D.; Nickel, B. Dichte Und Viskositat Des Isopentans von 115 K Bis 293 K. Z. Phys. Chem. (Leipzig) 1984, 141, 239.

(906)	Bleakney, J. W. Measurement on the Vapor Viscosities of the Two Common Pentanes, Two Pentanes and Carbon Tetrachloride. Physica (The Hague) 1932, 3, 123.

(907)	Day, R. Variation of the Vapor Viscosities of Pentane and Isopentane with Pressure by Rotating Cylynder Method. Phys. Rev. 1932, 40, 281–290.

(908)	McCoubrey, J. C.; Singh, N. M. The Vapor Phase Viscosities of the Pentanes. J. Phys. Chem. 1963, 67, 517.

(909)	Kumagai, A.; Tomida, D.; Yokoyama, C. Measurements of the Liquid Viscosities of Mixtures of Isobutane with Squalane to 30 MPa. Int. J. Thermophys. 2007, 28, 1111–1119. https://doi.org/10.1007/s10765-007-0259-y.

(910)	Gonzalez, M. H.; Lee, A. L. Viscosity of Isobutane. J. Chem. Eng. Data 1966, 11, 357–359. https://doi.org/10.1021/je60030a019.

(911)	Agaev, N. A.; Yusibova, A. D. Viscosity of Isobutane at High Pressures. Gaz. Prom. 1969, 14, 41.

(912)	Kuechenmeister, C.; Vogel, E. The Viscosity of Gaseous Isobutane and Its Initial Density Dependence. Int. J. Thermophys. 2000, 21, 329. https://doi.org/10.1023/a:1006671226710.

(913)	Ulybin, S. A.; Makarushkin, V. I.; Skorodumov, S. V. Viscosity of Krypton at Temperatures from 120-1300 K and at Pressures up to 100 Mpa. Teplofiz. Vys. Temp. 1978, 16, 282–287.

(914)	Golik, O. Z.; Cholpan, P. P. Molecular Structure, Compressibility, Surface Tension, and Viscosity of Some Polysiloxanes. Ukr. Fiz. Zh. 1960, 5, 242–251.

(915)	Sperkach, V. S.; Cholpan, P. F. Study of Acoustic Scattering in Some Siloxanes. Fiz. Zhidk. Sostoyaniya 1979, 104–109.

(916)	Matthews, J. B.; Sumner, J. F.; Moelwyn-Hughes, E. A. The Vapour Pressures of Certain Liquids. Trans. Faraday Soc. 1950, 46, 797–803.

(917)	Anonymous, X. The Physical and Chemical Properties for Devulcanized Solution. Chem. Eng. Oil Gas 1981, 1–24.

(918)	Song, J.-H.; Park, S.-B.; Yoon, J.-H.; Lee, H.; Lee, K.-H. Densities and Viscosities of Monoethanolamine + Ethylene Glycol + Water. J. Chem. Eng. Data 1996, 41, 1152–1154. https://doi.org/10.1021/je9601366.

(919)	Lee, M. J.; Lin, T.-K.; Pai, Y.-H.; Lin, K.-S. Density and Viscosity for Monoethanolamine + 1-Propanol, + 1-Hexanol, and + 1-Octanol. J. Chem. Eng. Data 1997, 42, 854–857. https://doi.org/10.1021/je9700636.

(920)	Maham, Y.; Liew, C. N.; Mather, A. E. Viscosities and Excess Properties of Aqueous Solutions of Ethanolamines from 25 to 80deg C. J. Solution Chem. 2002, 31, 743–756.

(921)	Islam, M. N.; Islam, M. M.; Yeasmin, M. N. Viscosity of Aqueous Solutions of 2-Methoxyethanol, 2-Ethoxyethanol, and Ethanolamine. J. Chem. Thermodyn. 2004, 36, 889–893. https://doi.org/10.1016/j.jct.2004.06.004.

(922)	Amundsen, T. G.; Oi, L. E.; Eimer, D. A. Density and Viscosity of Monoethanolamine + Water + Carbon Dioxide from (25 to 80) Deg C. J. Chem. Eng. Data 2009, 54, 3096–3100. https://doi.org/10.1021/je900188m.

(923)	Song, H.-J.; Lee, M.-G.; Kim, H.; Gaur, A.; Park, J.-W. Density, Viscosity, Heat Capacity, Surface Tension, and Solubility of CO2 in Aqueous Solutions of Potassium Serinate. J. Chem. Eng. Data 2011, 56, 1371–1377. https://doi.org/10.1021/je101144k.

(924)	Tian, S.; Ren, S.; Hou, Y.; Wu, W.; Peng, W. Densities, Viscosities and Excess Properties of Binary Mixtures of 1,1,3,3-Tetramethylguanidinium Lactate + Water at T = (303.15 to 328.15) K. J. Chem. Eng. Data 2013, 58, 1885–1892. https://doi.org/10.1021/je3009073.

(925)	Shaikh, M. S.; Shariff, A. M.; Bustam, M. A.; Murshid, G. Physicochemical Properties of Aqueous Solutions of Sodium L-Prolinate as an Absorbent for CO2 Removal. J. Chem. Eng. Data 2014, 59, 362–368. https://doi.org/10.1021/je400830w.

(926)	Xu, F.; Gao, H.; Dong, H.; Wang, Z.; Zhang, X.; Ren, B.; Zhang, S. Solubility of CO2 in Aqueous Mixtures of Monoethanolamine and Dicyanamide-Based Ionic Liquids. Fluid Phase Equilib. 2014, 365, 80–87. https://doi.org/10.1016/j.fluid.2013.12.020.

(927)	Shen, S.; Yang, Y. -n.; Wang, Y.; Ren, S.; Han, J.; Chen, A. CO2 Absorption into Aqueous Potassium Salts of Lysine and Proline: Density, Viscosity and Solubility of CO2. Fluid Phase Equilib. 2015, 399, 40–49. https://doi.org/10.1016/j.fluid.2015.04.021.

(928)	Rudenko, N. S.; Shubnikov, L. V. Variation in the Viscosity of Liquid Methane and Ethylene with Temperature. Phys. Z. Sowjetunion 1935, 8, 179.

(929)	Rudenko, N. S. Viscosity of the Liquids O(2), N(2), CH(4), C(2)H(4) and Air (in Russian). Zh. Eksp. Teor. Fiz. 1939, 9, 1078.

(930)	Bresler, S. E.; Landerman, A. Viscosity of Liquid Methane and Deuteriomethane. Zh. Eksp. Teor. Fiz. 1940, 10, 250.

(931)	Gerf, S. F.; Galkov, G. I. Viscosity of Liquid Pure Gases and Their Mixtures. Zh. Tekh. Fiz. 1941, 11, 801–808.

(932)	Mescheriakov, N. V.; Golubev, I. F. Viazkost’ Uglevodorodnykh Gazov Pri Vysokikh Davleniakh,; 1954.

(933)	Ross, I. F.; Brown, G. M. Viscosities of Gases at High Pressures. Ind. Eng. Chem. 1957, 49, 2026.

(934)	Pavlovich, N. V. Study of the Thermal Conductivity of Natural Gases and Methane. Gaz. Prom. 1959, 45–49.

(935)	Iwasaki, M.; Takahashi, H. The Viscosity of Methane at High Pressures. Kogyo Kagaku Zasshi 1959, 62, 918.

(936)	Carmichael, L. T.; Berry, V. M.; Sage, B. H. Viscosity of Hydrocarbons. Methane. J. Chem. Eng. Data 1965, 10, 57–61. https://doi.org/10.1021/je60024a020.

(937)	Huang, E. T. S.; Swift, G. W.; Kurata, F. Viscosities of Methane and Propane at Low Temperatures and High Pressures. AIChE J. 1966, 12, 932–936.

(938)	Hellemans, J. M.; Kestin, J.; Ro, S. T. Viscosity of Methane, Carbon Tetrafluoride and Sulfur Hexafluoride over a Range of Temperatures. Physica (Amsterdam) 1973, 65, 376.

(939)	Swift, G. W.; Christy, J. A.; Kurata, F. Liquid Viscosities of Methane and Propane. AIChE J. 1959, 5, 98–102.

(940)	Swift, G. W.; Lohrenz, J.; Kurata, F. Liquid Viscosities above the Normal Boiling Point for Methane, Ethane, Propane, and n-Butane. AIChE J. 1960, 6, 415–419.

(941)	Giddings, J. G.; Kao, J. T.; Kobayashi, R. Development of a High Pressure Capillary-Tube Viscometer and Its Application to Methane, Propane, and Their Mixtures in the Gaseous and Liquid Regions. J. Chem. Phys. 1966, 45, 578.

(942)	Hongo, M.; Yokoyama, C.; Takahashi, S. Viscosity of Methane-Chlorodiflouromethane (R22) Gaseous Mixtures in the Temperature Range from 298.15 to 373.15 K and at Pressures up to 5 MPa. J. Chem. Eng. Jpn. 1988, 21, 632–638.

(943)	vanderGulik, P. S.; Mostert, R.; vandenBerg, H. R. The Viscosity of Methane at 273 K up to 1 GPa. Fluid Phase Equilib. 1992, 79, 301–311. https://doi.org/10.1016/0378-3812(92)85139-y.

(944)	Jones, G.; Fornwalt, H. J. The Influence of Surface Tension on the Measurement of Viscosity The Viscosity of Methanol. J. Am. Chem. Soc. 1938, 60, 1683–1693. https://doi.org/10.1021/ja01274a043.

(945)	Sadek, H.; Fuoss, R. M. Electrolyte-Solvent Interaction I. Tetrabutylammonium Bromide in Methanol- Nitrobenzene Mixtures. J. Am. Chem. Soc. 1950, 72, 301–306. https://doi.org/10.1021/ja01157a080.

(946)	Mikhail, S. Z.; Kimel, W. R. Densities and Viscosities of Methanol-Water Mixtures. J. Chem. Eng. Data 1961, 6, 533–537. https://doi.org/10.1021/je60011a015.

(947)	Isakova, N. P.; Oshueva, L. A. Viscosity of Liquid Metyhanol at High Pressures. Zh. Fiz. Khim. 1966, 40, 1130–1131.

(948)	Kubota, H.; Tsuda, S.; Murata, M.; Yamamoto, T.; Tanaka, Y.; Makita, T. Specific Volume and Viscosity of Methanol-Water Mixtures under High Pressure. Rev. Phys. Chem. Jpn. 1979, 49, 59–69.

(949)	Singh, R. P.; Haque, M. M. Viscosity Variations in Associated Liquid Systems Showing Partial Miscibility: Part I-Kinematic Viscosities of Methanol-n-Butanol-Water Ternary System of Different Compositions. Indian J. Chem., Sect. A: Inorg., Phys., Theor. Anal. 1981, 20, 874–878.

(950)	Isdale, J. D.; Easteal, A. J.; Woolf, L. A. Shear Viscosity of Methanol and Methanol + Water Mixtures Under Pressure. Int. J. Thermophys. 1985, 6, 439–450. https://doi.org/10.1007/bf00508889.

(951)	Liu, H.; Yu, D.; Feng, G.; Yang, X.; Nie, Q. Measurement and Correlation of the Viscosities of Water-Methanol-Lithium Chloride System. Tianranqi Huagong 1994, 19, 52–56.

(952)	Reddy, V. K.; Rambabu, K.; Devarajulu, T.; Krishnaiah, A. Volume of Mixing, Speed of Sound, and Viscosity of Methyl Cellosolve with Aliphatic Alcohols at 308.15 K. J. Chem. Eng. Data 1995, 40, 124–127. https://doi.org/10.1021/je00017a028.

(953)	Saha, N.; Das, B.; Hazra, D. K. Viscosities and Excess Molar Volumes for Acetonitrile + Methanol at 298.15, 308.15, and 318.15 K. J. Chem. Eng. Data 1995, 40, 1264–1266. https://doi.org/10.1021/je00022a026.

(954)	Sastry, N. V.; Raj, M. M. Excess Molar Volumes and Isentropic Compressibilities of Binary Mixtures Containing Methyl Methacrylate-n-Alcohols at 303.15 and 313.15 K. Thermochim. Acta 1995, 257, 39–50. https://doi.org/10.1016/0040-6031(94)02207-5.

(955)	Muhuri, P. K.; Das, B.; Hazra, D. K. Apparent Molar Volumes and Apparent Molar Adiabatic Compressibilities of Some Symmetrical Tetraalkylammonium Bromides in 1,2-Dimethoxyethane. Indian J. Chem., Sect. A: Inorg., Phys., Theor. Anal. 1996, 35, 288–293.

(956)	Bakshi, M. S.; Kaur, G. Thermodynamic Behavior of Mixtures. 4. Mixtures of Methanol with Pyridine and N,N-Dimethylformamide at 25 C. J. Chem. Eng. Data 1997, 42, 298–300. https://doi.org/10.1021/je960300p.

(957)	George, M. A.; Young, K. M.; Robertson III, E. A.; Beck, S. E.; Voloshin, G. Vapor Pressure and Viscosity of 1,1,1,5,5,5-Hexafluoro-2,4-Pentanedione. J. Chem. Eng. Data 1998, 43, 60–64. https://doi.org/10.1021/je9700941.

(958)	Rodriguez, A.; Canosa, J.; Tojo, J. Dynamic Viscosities of the Ternary Liquid Mixture Involving CH3COOCH3, CH3OH, and CH3CH2CH2OH at T = 298.15 K. J. Chem. Thermodyn. 1998, 30, 1307–1318. https://doi.org/10.1006/jcht.1998.0396.

(959)	Sastry, N. V.; Valand, M. K. Densities, Viscosities, and Relative Permittivities for Pentane + 1-Alcohols (C1 to C12) at 298.15 K. J. Chem. Eng. Data 1998, 43, 152–157. https://doi.org/10.1021/je9701801.

(960)	Canosa, J.; Rodriguez, A.; Tojo, J. Dynamic Viscosities of the Binary Mixtures (Methyl Acetate or Methanol + 2-Methyl-2-Butanol) and the Ternary Mixtures (Methyl Acetate + Methanol + 2-Propanol, or 2-Butanol, or 2-Methyl-2-Butanol) at T=298.15 K. J. Chem. Thermodyn. 2000, 32, 551–565. https://doi.org/10.1006/jcht.2000.0628.

(961)	Pal, A.; Dass, G. Excess Molar Volumes and Viscosities for Binary Liquid Mixtures of 2-Propoxyethanol and of 2-Isopropoxyethanol with Methanol, 1-Propanol, 2-Propanol, and 1-Pentanol at 298.15 K. J. Chem. Eng. Data 2000, 45, 693–698. https://doi.org/10.1021/je0000120.

(962)	Prasad, T. E. V.; Agrawal, S. B.; Bajaj, A. B.; Prasad, D. H. L. DENSITY AND VISCOSITY OF METHANOL + TRICHLOROETHYLENE, n-PROP ANOL + TRICHLOROETHYLENE AND n-BUTANOL + TRICHLOROETHYLENE MIXTURES. Phys. Chem. Liq. 2000, 38, 433–438.

(963)	Prasad, T. E. V.; Vallabhaneni, G. K.; Sameera, M. K.; Bose, V. P.; Prasad, D. H. L. Density and Viscosity of the Binary Mixtures Formed by P-Xylene with Methanol, n-Propanol and n-Butanol. Phys. Chem. Liq. 2000, 38, 475–480.

(964)	Saha, N.; Das, B. Apparent Molar Volumes of Some Symmetrical Tetraalkylammonium Bromides in Methanol + Acetonitrile Mixtures at 298.15 K. J. Mol. Liq. 2000, 89, 169–174.

(965)	Visak, Z. P.; Ferreira, A. G. M.; Fonseca, I. M. A. Densities and Viscosities of the Ternary Mixtures Water + Butyl Acetate + Methanol and Water + Ethyl Propionate + Methanol at 303.15 K. J. Chem. Eng. Data 2000, 45, 296–931. https://doi.org/10.1021/je000085j.

(966)	George, J.; Sastry, N. V.; Patel, S. R.; Valand, M. K. Densities, Viscosities, Speeds of Sound, and Relative Permittivities for Methyl Acrylate + 1-Alcohols (C1-C6) at T = (308.15 and 318.15) K. J. Chem. Eng. Data 2002, 47, 262–269. https://doi.org/10.1021/je010268l.

(967)	Heintz, A.; Klasen, D.; Lehmann, J. K. Excess Molar Volumes and Viscosities of Binary Mixtures of Methanol and the Ionic Liquid 4-Methyl-N-Butylpyridinium Tetrafluoroborate at 25, 40, and 50 Deg C. J. Solution Chem. 2002, 31, 467–476.

(968)	Giro, F.; Goncalves, M.; Ferreira, A. G. M.; Fonseca, I. M. A. Viscosity and Density Data of the System Water + N-Pentyl Acetate + Methanol Calculations with a Modified Redlich-Kwong-Soave Equation of State. Fluid Phase Equilib. 2003, 204, 217–232. https://doi.org/10.1016/s0378-3812(02)00262-5.

(969)	Kijevcanin, M. L.; Ribeiro, I. S. A.; Ferreira, A. G. M.; Fonseca, I. M. A. Densities, Viscosities, and Surface and Interfacial Tensions of the Ternary Mixture Water + Ethyl Butyrate + Methanol at 303.15 K. J. Chem. Eng. Data 2003, 48, 1266–1270. https://doi.org/10.1021/je0301500.

(970)	Chatterjee, A.; Das, B. Electrical Conductances of Tetrabutylammonium Bromide, Sodium Tetraphenylborate, and Sodium Bromide in Methanol (1) + Water (2) Mixtures at (298.15, 308.15, and 318.15) K. J. Chem. Eng. Data 2006, 51, 1352–1355. https://doi.org/10.1021/je0600810.

(971)	Zafarani-Moattar, M. T.; Majdan-Cegincara, R. Viscosity, Density, Speed of Sound, and Refractive Index of Binary Mixtures of Organic Solvent + Ionic Liquid, 1-Butyl-3-Methylimidazolium Hexafluorophosphate at 298.15 K. J. Chem. Eng. Data 2007, 52, 2359–2364. https://doi.org/10.1021/je700338t.

(972)	Pal, A.; Gaba, R. Volumetric, Acoustic, and Viscometric Studies of Molecular Interactions in Binary Mixtures of Dipropylene Glycol Dimethyl Ether with 1-Alkanols at 298.15 K. J. Chem. Thermodyn. 2008, 40, 818–828. https://doi.org/10.1016/j.jct.2008.01.008.

(973)	Davila, M. J.; Aparicio, S.; Alcalde, R. Thermophysical Properties of Binary and Ternary Mixtures Containing Lactams and Methanol. Ind. Eng. Chem. Res. 2009, 48, 10065–10076. https://doi.org/10.1021/ie900822e.

(974)	Jain, D. M.; Shah, V.; Rabadiya, S.; Oswal, S. Viscosity and Excess Molar Volume of Binary Mixtures of Methanol with N-Butylamine and Di-n-Butylamine at 303.15, 313.15 and 323.15 K. Characterization in Terms of ERAS Model. J. Mol. Liq. 2009, 144, 65–70.

(975)	Roy, M. N.; Dewan, R. K.; Sarkar, L. Ion-Solvent Interactions of Some Halides of Common Cations with Organic Solvent Mixtures by Conductometric, Volumetric, Viscometric, and Refractometric Techniques. J. Chem. Eng. Data 2010, 55, 1347–1353. https://doi.org/10.1021/je900656c.

(976)	Shekaari, H.; Mousavi, S. S. Volumetric Properties of Ionic Liquid 1,3-Dimethylimidazolium Methyl Sulfate + Molecular Solvents at T = (298.15 - 328.15) K. Fluid Phase Equilib. 2010, 291, 201–207. https://doi.org/10.1016/j.fluid.2010.01.002.

(977)	Pal, A.; Gaba, R.; Kumar, H. Acoustic, Viscometric, and Spectroscopic Studies of Dipropylene Glycol Monopropyl Ether with n-Alkanols at Temperatures of 288.15, 298.15, and 308.15 K. J. Solution Chem. 2011, 40, 786–802. https://doi.org/10.1007/s10953-011-9688-0.

(978)	Wang, L.-S.; Tian, N.-N. Densities and Viscosities of Binary Mixtures of Methanol with Dimethyl Methylphosphonate and Dimethyl Phosphite from (293.15 to 333.15) K. Int. J. Thermophys. 2011, 32, 1175–1187. https://doi.org/10.1007/s10765-011-0989-8.

(979)	Warrier, P.; Teja, A. S. Density, Viscosity, and Thermal Conductivity of Mixtures of 1-Ethoxy-1,1,2,2,3,3,4,4,4-Nonafluorobutane (HFE 7200) with Methanol and 1-Ethoxybutane. J. Chem. Eng. Data 2011, 56, 4291–4294. https://doi.org/10.1021/je1011828.

(980)	Yu, Z.; Gao, H.; Wang, H.; Chen, L. Densities, Viscosities, and Refractive Properties of the Binary Mixtures of the Amino Acid Ionic Liquid [Bmim][Ala] with Methanol or Benzylalcohol at T = (298.15 to 313.15) K. J. Chem. Eng. Data 2011, 56, 2877–2883. https://doi.org/10.1021/je2000606.

(981)	Marczak, W.; Adamczyk, N.; Lezniak, M. Viscosity of Associated Mixtures Approximated by the Grunberg-Nissan Model. Int. J. Thermophys. 2012, 33, 680–691. https://doi.org/10.1007/s10765-011-1100-1.

(982)	Qian, W.; Xu, Y. J.; Zhu, H.; Yu, C. Properties of Pure 1-Methylimidazolium Acetate Ionic Liquid and Its Binary Mixtures with Alcohols. J. Chem. Thermodyn. 2012, 49, 87–94. https://doi.org/10.1016/j.jct.2012.01.013.

(983)	Singh, S.; Yasmin, M.; Shukla, D.; Parveen, S.; Gupta, M. Thermoacoustical Study of Molecular Interactions in Binary Mixtures of Pentane with Methanol and Tert-Butanol. Phys. Chem. Liq. 2012, 50, 210–221. https://doi.org/10.1080/00319104.2011.597030.

(984)	Yu, Z.; Gao, H.; Wang, H.; Chen, L. Thermodynamic Properties of Binary Mixtures of the Amino Acid Ionic Liquids [Bmim][Glu] or [Bmim][Gly] with Methanol at T = 298.15 to 313.15 K. J. Solution Chem. 2012, 41, 173–186. https://doi.org/10.1007/s10953-011-9788-x.

(985)	Brahman, D.; Sinha, B. Effect of Tetrabutylammonium Bromide on Solution Behavior of Salicylaldehyde Anil Zinc (II) in Methanol at T = (298.15, 308.15 and 318.15) K. J. Chem. Thermodyn. 2013, 67, 13–20. https://doi.org/10.1016/j.jct.2013.07.010.

(986)	Gupta, S.; Chattejee, A.; Das, S.; Basu, B. Electrical Conductances of 1-Butyl-3-Propylimidazolium Bromide and 1-Butyl-3-Propylbenzimidazolium Bromide in Water, Methanol, and Acetonitrile at (308, 313, and 318) K at 0.1 MPa. J. Chem. Eng. Data 2013, 58, 1–6. https://doi.org/10.1021/je300339q.

(987)	Jain, P.; Sharma, S.; Shukla, R. K. Density and Viscosity of Tetrabutyl Ammonium Hydrogen Sulphate and Tetrabutyl Ammonium Chloride Salts in Aqueous and Methanolic Solution at 303 K. Phys. Chem. Liq. 2013, 51, 547–566. https://doi.org/10.1080/00319104.2012.760084.

(988)	Brahman, D.; Sinha, B. Partial Molar Volumes and Viscosity B-Coefficients for N,N’-Ethylenebis(Salicylideneiminato)-Diaquochromium(III) Chloride in Methanolic Solutions of 1-Butyl-2,3-Dimethylimidazolium Tetrafluoroborate at T = (298.15, 308.15, and 318.15) K. J. Chem. Thermodyn. 2014, 68, 260–269. https://doi.org/10.1016/j.jct.2013.09.018.

(989)	Garcia-Mardones, M.; Martin, S.; Gascon, I.; Lafuente, C. Thermophysical Properties of the Binary Mixture 1-Propylpyridinium Tetrafluoroborate with Methanol. J. Chem. Eng. Data 2014, 59, 1564–1573. https://doi.org/10.1021/je401120a.

(990)	Wu, J.-Y.; Chen, Y.-P.; Su, C.-S. Density and Viscosity of Ionic Liquid Binary Mixtures of 1-n-Butyl-3-Methylimidazolium Tetrafluoroborate with Acetonitrile, N,N-Dimethylacetamide, Methanol, and N-Methyl-2-Pyrrolidone. J. Solution Chem. 2015, 44, 395–412. https://doi.org/10.1007/s10953-014-0273-1.

(991)	Teske, V.; Vogel, E. Viscosity Measurements on Methanol Vapor and Their Evaluation. J. Chem. Eng. Data 2006, 51, 628–635. https://doi.org/10.1021/je050429b.

(992)	Ceriani, R.; Goncalves, C. B.; Rabelo, J.; Caruso, M.; Cunha, A. C. C.; Cavaleri, F. W.; Batista, E. A. C.; Meirelles, A. J. A. Group Contribution Model for Predicting Viscosity of Fatty Compounds. J. Chem. Eng. Data 2007, 52, 965–972. https://doi.org/10.1021/je600552b.

(993)	Pratas, M. J.; Freitas, S.; Oliveira, M.; Monteiro, S.; Lima, A. S.; Coutinho, J. A. P. Densities and Viscosities of Fatty Acid Methyl and Ethyl Esters. J. Chem. Eng. Data 2010, 55, 3983–3990. https://doi.org/10.1021/je100042c.

(994)	Pratas, M. J.; Freitas, S.; Oliveira, M. B.; Monteiro, S. C.; Lima, A. S.; Coutinho, J. A. P. Densities and Viscosities of Minority Fatty Acid Methyl and Ethyl Esters Present in Biodiesel. J. Chem. Eng. Data 2011, 56, 2175–2180. https://doi.org/10.1021/je1012235.

(995)	Treibs, W. Ber. Dtsch. Chem. Ges. B 1942, 75, 331–335. https://doi.org/10.1002/cber.19420750405.

(996)	Allen, C. A. W.; Watts, K. C.; Ackmann, R. G.; Pegg, M. J. Predicting the Viscosity of Biodiesel Fuels from Their Fatty Acid Ester Composition. Fuel 1999, 78, 1319–1326. https://doi.org/10.1016/S0016-2361(99)00059-9.

(997)	Gubareva, A. I. Temperature Depencence of Physico-Chemical Properties of Hexamethyldisiloxane and Diphenylmethylsilane; 874khp-D83; ONIITeKhim (Cherkassy UA), 1983.

(998)	Keffler, L. J. P.; MacLean, J. H. Homology in Long-Chain Compounds - Oleic Acid and the n-Alkyl Oleates. J. Soc. Chem. Ind. London Trans. Commun. 1935, 54, 178–185.

(999)	Albert, O. Viscosity Measurements on Homologous Ester Series with Special Regard to the Relations of Thorpe and Roger. Z. Phys. Chem. Abt. A 1938, 182, 421–429.

(1000)	Menzel; Berger; Nikuradse. Chem. Ber. 1949, 82, 418.

(1001)	YAMAWAKI, H. Pressure Dependence of Viscosity for Methyl Oleate and Methyl Linoleate up to 400 MPa. Int. J. Thermophys. 2020, 41, 112. https://doi.org/10.1007/s10765-020-02693-w.

(1002)	Keffler, L.; McLean, J. H. Homology in Long-Chain Compounds. I. Oleic Acid and the n-Alkyl Oleates. J. Soc. Chem. Ind. (London) 1935, 54, 178–185.

(1003)	Bhuyan, S.; Sundararajan, S.; Yao, L.; Hammond, E. G.; Wang, T. Boundary Lubrication Properties of Lipid-Based Compounds Evaluated Using Microtribological Methods. Tribol. Lett. 2006, 22, 167–172.

(1004)	Yao, L.; Hammond, E.; Wang, T. Melting Points and Viscosities of Fatty Acid Esters That Are Potential Targets for Engineered Oilseed. J. Am. Oil Chem. Soc. 2008, 85, 77–82. https://doi.org/10.1007/s11746-007-1159-9.

(1005)	Gaikwad, B. R.; Subrahmanyam, V. V. R. Physical Properties of N-Saturated Higher Fatty Alcohols in Liquid State. J. Indian Chem. Soc. 1988, 65, 266–268.

(1006)	Gros, A. T.; Feuge, R. O. Surface and Interfacial Tensions, Viscosities, and Other Physical Properties of Some n-Aliphatic Acids and Their Methyl and Ethyl Esters. J. Am. Oil Chem. Soc. 1952, 29, 313–317.

(1007)	Timmermans, J.; Hennaut-Roland, M. The Work of the International Bureau of Physical-Chemical Standards IV Study of the Physical Constants of Twenty Organic Compounds. J. Chim. Phys. Phys.-Chim. Biol. 1930, 27, 401–438.

(1008)	Al-Madfai, S. F.; Awwad, A. M.; Jbara, K. A. Some Properties of Binary Mixtures of N-Formylmorpholine and an Aromatic Hydrocarbon. Thermochim. Acta 1985, 84, 33–40. https://doi.org/10.1016/0040-6031(85)85372-7.

(1009)	Oshmyansky, Y.; Hanley, H. J. M.; Ely, J. F.; Kidnay, A. J. The Viscosities and Densities of Selected Organic Compounds and Mixtures of Interest in Coal Liquefaction Studies. Int. J. Thermophys. 1986, 7, 599. https://doi.org/10.1007/bf00502393.

(1010)	Goud, B. B.; Venkateswarlu, P.; Rao, M. V. P. Excess Volumes and Viscosities of Isomeric Xylenes with O-Nitrotoluene or m-Nitrotoluene at 308.15 K. J. Chem. Eng. Data 1999, 44, 731–734. https://doi.org/10.1021/je9900023.

(1011)	Moumouzias, G.; Ritzoulis, G. Viscosities and Densities for Propylene Carbonate + Toluene at 15, 20, 25, 30, and 35.Degree.C. J. Chem. Eng. Data 1992, 37, 482–483. https://doi.org/10.1021/je00008a024.

(1012)	Singh, S.; Sethi, B. P. S.; Katyal, R. C.; Rattan, V. K. Viscosities, Densities, and Speeds of Sound of Binary Mixtures of o-Xylene, m-Xylene, p-Xylene, and Isopropylbenzene with 4-Methylpentan-2-One at 298.15 K. J. Chem. Eng. Data 2004, 49, 1373–1375. https://doi.org/10.1021/je049899g.

(1013)	Al-Kandary, J. A.; Aljimaz, A. S.; Abdul-Latif, A.-H. M. Densities, Viscosities, and Refractive Indices of Binary Mixtures of Anisole with Benzene, Methylbenzene, Ethylbenzene, Propylbenzene, and Butylbenzene at (293.15 and 303.15) K. J. Chem. Eng. Data 2006, 51, 99–103. https://doi.org/10.1021/je0502546.

(1014)	Yang, T.; Xia, S.; Song, S.; Fu, X.; Ma, P. Densities and Viscosities of N-Formylmorpholine (NFM) + p-Xylene, + o-Xylene, + m-Xylene at Different Temperatures and Atmospheric Pressure. J. Chem. Eng. Data 2007, 52, 2062–2066. https://doi.org/10.1021/je7002513.

(1015)	Das, K.; Habibullah, M.; Rahman, I. M. M.; Hasegawa, H.; Uddin, K. S.; Saifuddin, K. Thermodynamic Properties of the Binary Mixture of Hexan-1-Ol with m-Xylene at T = (303.15, 313.15, and 323.15) K. J. Chem. Eng. Data 2009, 54, 3300–3302. https://doi.org/10.1021/je900219w.

(1016)	Habibullah, M.; Das, K. N.; Rahman, I. M. M.; Hasegawa, H.; Uddin, M. A.; Saifuddin, K. Volumetric and Viscometric Behavior of the Binary System: (Hexan-1-Ol + p-Xylene). J. Chem. Eng. Data 2010, 55, 5311–5313. https://doi.org/10.1021/je100390b.

(1017)	Zarei, H.; Salami, Z. Densities, Excess Molar Volumes, Viscosity, and Refractive Indices of Binary Mixtures of Ethanoic Acid and Trichloroethylene with Dimethylbenzenes at Different Temperatures. J. Chem. Eng. Data 2012, 57, 620–625. https://doi.org/10.1021/je201211f.

(1018)	Dikio, E. Z. D.; Vilakazi, G.; Ngoy, P. Density, Dynamic Viscosity and Derived Properties of Binary Mixtures of m-Xylene, o-Xylene, and p-Xylene, with Pyridine at T=293.15, 303.15, 313.15 and 323.15 K. J. Mol. Liq. 2013, 177, 190–197. https://doi.org/10.1016/j.molliq.2012.10.021.

(1019)	Yokoyama, C.; Takahashi, M.; Takahashi, S. Viscosity of Nitrous Oxide in the Critical Region. Int. J. Thermophys. 1994, 15, 603–626. https://doi.org/10.1007/bf01563790.

(1020)	Hurly, J. J. Viscosity and Speed of Sound of Gaseous Nitrous Oxide and Nitrogen Trifluoride Measured with a Greenspan Viscometer. Int. J. Thermophys. 2004, 25, 625–641. https://doi.org/10.1023/b:ijot.0000034229.03525.aa.

(1021)	Takahashi, M.; Shibasaki-Kitakawa, N.; Yokoyama, C.; Takahashi, S. Viscosity of Gaseous Nitrous Oxide from 298.15 K to 398.15 K at Pressures up to 25 MPa. J. Chem. Eng. Data 1996, 41, 1495–1498. https://doi.org/10.1021/je960060d.

(1022)	Phibbs, M. K. Viscosity of Neopentane. J. Chem. Phys. 1951, 19, 1420.

(1023)	Gonzalez, M. H.; Lee, A. L. Viscosity of 2,2-Dimethylpropane. J. Chem. Eng. Data 1968, 13, 66–69. https://doi.org/10.1021/je60036a021.

(1024)	Diaz Pena, M.; Esteban, F. Viscosity of Quasi-Spherical Molecules in the Vapor Phase. An. R. Soc. Esp. Fis. Quim., A 1966, 62, 347–357.

(1025)	Michels, A.; Botzen, A. The Thermal Conductivity of Nitrogen at Pressures up to 2500 Atm. Physica (Amsterdam) 1953, 19, 585.

(1026)	Ellis, C. P.; Raw, C. J. G. High-Temperature Gas Viscosities. II. Nitrogen, Nitric Oxide, Boron Trifluoride, Silicon Tetrafluoride, and Sulfur Hexafluoride. J. Chem. Phys. 1959, 30, 574–576.

(1027)	Flynn, G. P.; Hanks, R. V.; Lemaire, N. A.; Ross, J. Viscosity of Nitrogen, Helium, Neon, and Argon from -78.5 Degrees to 100 Degrees C below 200 Atmospheres. J. Chem. Phys. 1963, 38, 154.

(1028)	Goldman, K. Viscosity of Nitrogen at Low Temperatures and High Pressures. Physica (Amsterdam) 1963, 29, 499.

(1029)	Iwasaki, H.; Kestin, J. The Viscosity of Argon-Helium Mixtures. Physica (Amsterdam) 1963, 29, 1345–1372. https://doi.org/10.1016/s0031-8914(63)80243-8.

(1030)	Kestin, J.; Whitelaw, J. H. A Relative Determination of the Viscosity of Several Gases by the Oscillating Disk Method. Physica (Amsterdam) 1963, 29, 335.

(1031)	DiPippo, R.; Kestin, J.; Whitelaw, J. H. Physica (Amsterdam) 1966, 32, 2064.

(1032)	Clarke, A. G.; Smith, E. B. Low-Temperature Viscosities of Argon, Krypton and Xenon. J. Chem. Phys. 1968, 48, 3988.

(1033)	Clarke, A. G.; Smith, E. B. Low Temperature Viscosities and Intermolecular Forces of Simple Gases. J. Chem. Phys. 1969, 51, 4156–4161.

(1034)	Vermesse, J. Messure Du Coefficient de Viscosite de l’azote a Haute Pression. Ann. Phys. (Paris) 1969, 4, 245.

(1035)	Dawe, R. A.; Smith, E. B. Viscosities of the Inert Gases at High Temperatures. J. Chem. Phys. 1970, 52, 693.

(1036)	Kestin, J.; Paykoc, E.; Sengers, J. V. On the Density Expansion for Viscosity in Gases. Physica (Amsterdam) 1971, 54, 1.

(1037)	Golubev, I. F.; Kurin, V. I. Measurement of the Viscosity of Gases at Pressures to 4,000 Kgf/Cm2 and Different Temperatures. Teploenergetika 1974, 83–85.

(1038)	Maitland, G. C.; Smith, E. B. Viscosity of Binary Gas Mixtures at High Temperatures. J. Chem. Soc., Faraday Trans. 1 1974, 70, 1191–1211.

(1039)	Zozulya, V. N.; Blagoi, Yu. P. Viscosity of Nitrogen Nearby Critical Point Liquid-Gas. Zh. Eksp. Teor. Fiz. 1974, 66, 212.

(1040)	Hoogland, J. H. B.; Van Den Berg, H. R.; Trappeniers, N. J. Measurements of the Viscosity of Sulfur Hexafluoride up to 100 Bar by a Capillary-Flow Viscometer. Phys. A (Amsterdam, Neth.) 1985, 134, 169–192.

(1041)	Audonnet, F.; Padua, A. A. H. Simultaneous Measurement of Density and Viscosity of N-Pentane from 298 to 383 K and up to 100 MPa Using a Vibrating-Wire Instrument. Fluid Phase Equilib. 2001, 181, 147–161. https://doi.org/10.1016/s0378-3812(01)00487-3.

(1042)	Lv, S.; Zhao, X.; Yao, C.; Wang, W.; Guo, Z. Viscosity of Gaseous Ethyl Fluoride (HFC-161). Fluid Phase Equilib. 2014, 384, 100–105. https://doi.org/10.1016/j.fluid.2014.10.023.

(1043)	Van Itterbeek, A.; Zink, H.; Hellemans, J. Viscosity of Liquified Gases at Pressures above One Atmosphere. Physica (Amsterdam) 1966, 32, 489.

(1044)	Grevendonk, W.; Herreman, W.; DeBock, A. Measurement of the Viscosity of Liquid Nitrogen. Physica (Amsterdam) 1970, 46, 600.

(1045)	Rutherford, W. M. Viscosity of Liquid-Phase Methyl Chloride. J. Chem. Eng. Data 1984, 29, 163–165. https://doi.org/10.1021/je00036a019.

(1046)	Seibt, D.; Vogel, E.; Bich, E.; Buttig, D.; Hassel, E. Viscosity Measurements on Nitrogen. J. Chem. Eng. Data 2006, 51, 526–533. https://doi.org/10.1021/je050399c.

(1047)	Keramidi, A. S.; Rastorguev, Yu. L. Viscosity of Hydrocarbons. n-Nonane. Izv. Vyssh. Uchebn. Zaved., Neft Gaz 1972, 15, 65–68.

(1048)	Landaverde-Cortes, D. C.; Iglesias-Silva, G. A.; Ramos-Estrada, M.; Hall, K. R. Densities and Viscosities of MTBE + Nonane or Decane at p = 0.1 MPa from (273.15 to 363.15) K. J. Chem. Eng. Data 2008, 53, 288–292. https://doi.org/10.1021/je700428f.

(1049)	Islam, N.; Waris, B. Viscosities o Organic Liquids and the Free Volume Model. Indian J. Chem., Sect. A: Inorg., Phys., Theor. Anal. 1976, 14, 30.

(1050)	Badalyan, A. G.; Rodchenko, S. I. Viscosity of N-Octane. Izv. Vyssh. Uchebn. Zaved., Neft Gaz 1986, 29, 61.

(1051)	Harris, K. R.; Malhotra, R.; Woolf, L. A. Temperature and Density Dependence of the Viscosity of Octane and Toluene. J. Chem. Eng. Data 1997, 42, 1254–1260. https://doi.org/10.1021/je970105q.

(1052)	Barrufet, M. A.; Hall, K. R.; Estrada-Baltazar, A.; Iglesias-Silva, G. A. Liquid Viscosity of Octane and Pentane + Octane Mixtures from 298.15 K to 373.15 K up to 25 MPa. J. Chem. Eng. Data 1999, 44, 1310–1314. https://doi.org/10.1021/je990043z.

(1053)	Trenzado, J. L.; Matos, J. S.; Gonzalez, E.; Romano, E.; Caro, M. N. A Study on Properties Derived from Densities and Viscosities for the Ternary Systems (Methyl Pentanoate or Methyl Heptanoate) + n-Octane + 1-Hexanol and Their Binary Subsystems at Various Temperatures. J. Chem. Eng. Data 2003, 48, 1004–1014. https://doi.org/10.1021/je034017j.

(1054)	Abdulagatov, I. M.; Azizov, N. D. (P,Rho,T,x) and Viscosity Measurements of (X1 n-Heptane + (1 - X1) n-Octane) Mixtures at High Temperatures and High Pressures. J. Chem. Thermodyn. 2006, 38, 1402–1415. https://doi.org/10.1016/j.jct.2006.01.012.

(1055)	Estrada-Baltazar, A.; Iglesias-Silva, G.; Caballero-Ceron, C. Volumetric and Transport Properties of Binary Mixtures of N-Octane + Ethanol, + 1-Propanol, + 1-Butanol, and + 1-Pentanol from (293.15 to 323.15) K at Atmospheric Pressure. J. Chem. Eng. Data 2013, 58, 3351–3363. https://doi.org/10.1021/je4004806.

(1056)	Dumitrescu, V.; Budeanu, M. M.; Radu, S.; Camenita, A. D. Densities and Viscosities of Binary Mixtures of 2-Methoxy-2-Methylpropane with n-Octane. Phys. Chem. Liq. 2015, 53, 242–251. https://doi.org/10.1080/00319104.2014.972554.

(1057)	Lavushev, A. V.; Lyusternik, V. E. Measurements of the Viscosity of Oxygen at Atmospheric Pressure up to 2000 K. Zh. Fiz. Khim. 1976, 50, 3009–3311.

(1058)	Timmermans, J.; Hennaut-Roland, M. Work of the International Bureau of Physical-Chemical Standards. IX. The Physical Constants of Twenty Organic Compounds. J. Chim. Phys. Phys.-Chim. Biol. 1955, 52, 223.

(1059)	Mamedov, A. M.; Akhundov, T. S.; Tairov, A. D. Experimental Study of O-Xylene Viscosity at Temperatures up to 275 c and Pressures up to 400 Bars. Izv. Vyssh. Uchebn. Zaved., Neft Gaz 1967, 10, 72–74.

(1060)	Ramachandran, D.; Rambabu, K.; Krishnan, K. M.; Venkateswarlu, P.; Raman, G. K. Volume of Mixing, Speed of Sound, and Viscosity of Butyl Acetate with Xylenes at 303.15 K. J. Chem. Eng. Data 1995, 40, 815–817. https://doi.org/10.1021/je00020a017.

(1061)	Moumouzias, G.; Ritzoulis, C.; Ritzoulis, G. A Study in Mixtures of -Butyrolactone with o-Xylene and m-Xylene: Densities and Viscosities. J. Chem. Eng. Data 1999, 44, 1187–1191. https://doi.org/10.1021/je990094w.

(1062)	Das, K. N.; Habibullah, M.; Uddin, K. S.; Barua, P. The Thermodynamic Excess Properties of Binary Mixtures: I. Hexanol-1 + o-Xylene. J. Solution Chem. 2006, 35, 1017–1028.

(1063)	Sage, B. H.; Lacey, W. N. Viscosity of Hydrocarbon Solutions. Viscosity of Liquid and Gaseous Propane. Ind. Eng. Chem. 1938, 30, 829–834.

(1064)	Hubbard, R. M.; Brown, G. G. Viscosity of N-Pentane. Ind. Eng. Chem. 1943, 35, 1276–1280.

(1065)	Reamer, H. H.; Cokelet, G.; Sage, B. H. Viscosity of Fluids at High Pressures. Rotating Cylinder Viscometer and the Viscosity of Pentane. Anal. Chem. 1959, 31, 1422.

(1066)	Belinskii, B. A.; Ikramov, S. K. COMPREHENSIVE INVESTIGATION OF THE ACOUSTICAL PARAMETERS, VISCOSITY, AND DENSITY OF n-PENTANE OVER A WIDE PRESSURE INTER VAL. Sov. Phys. - Acoust. 1973, 18, 300–303.

(1067)	Postigo, M. A.; Zurita, J. L.; De Soria, M. L. G.; Katz, M. Excess Thermodynamic Properties of the N-Pentanedichloromethane System at 298.15 K. Can. J. Chem. 1986, 64, 1966–1968. https://doi.org/10.1139/v86-325.

(1068)	De Soria, M. L. G.; Zurita, J. L.; Postigo, M. A.; Katz, M. Excess Thermodynamic Properties of the N-Pentane + Methylacetate System at 298.15 K. Thermochim. Acta 1988, 130, 249–258. https://doi.org/10.1016/0040-6031(88)87071-0.

(1069)	Acevedo, I. L.; Pedrosa, G. C.; Katz, M. Dynamic Viscosities of Non-Electrolyte Mixtures. Phys. Chem. Liq. 1993, 26, 99–106.

(1070)	Abdulagatov, I. M.; Rasulov, S. M.; Abdurakhmanov, I. M. Pentane Dynamics near the Liquid-Gas Critical Point. Zh. Fiz. Khim. 1991, 65, 1306–1311.

(1071)	Diller, D. E. Measurements of the Viscosity of Saturated and Compressed Liquid Propane. J. Chem. Eng. Data 1982, 27, 240–243. https://doi.org/10.1021/je00029a003.

(1072)	Klemenc-Wien, A.; Remi, W. Experimental Investigation of the Viscosity of Nitric Oxide, Propane, and Their Mixtures with Hydrogen. Monatsh. Chem. 1924, 44, 307.

(1073)	Starling, K. E.; Eakin, B. E.; Ellington, R. T. Liquid, Gas, and Dense-Fluid Viscosity of Propane. AIChE J. 1960, 6, 438–442.

(1074)	Carmichael, L. T.; Berry, V. M.; Sage, B. H. Viscosity of Hydrocarbons. Propane. J. Chem. Eng. Data 1964, 9, 411–415. https://doi.org/10.1021/je60022a038.

(1075)	Golubev, I. F.; Gnezdilov, N. E. Viazkost’ Gazovykh Smesei; 1971.

(1076)	Nagaoka, K.; Yamashita, Y.; Tanaka, Y.; Kubota, H.; Makita, T. Viscosity of Binary Gaseous Mixtures of Fluorocarbons. J. Chem. Eng. Jpn. 1986, 19, 263.

(1077)	Vogel, E. The Viscosity of Gaseous Propane and Its Initial Density Dependence. Int. J. Thermophys. 1995, 16, 1335–1351. https://doi.org/10.1007/bf02083544.

(1078)	Wilhelm, J.; Vogel, E. Viscosity Measurements on Gaseous Propane. J. Chem. Eng. Data 2001, 46, 1467–1471. https://doi.org/10.1021/je010062k.

(1079)	Haepp, H. J. Messung Der Viscositat von Kohlendioxid Und Propylen. Warme- Stoffubertrag. 1976, 9, 281.

(1080)	Zimakov, P. V.; Sokolava, A. V. Physical Properties of Propylene Oxide. Zh. Fiz. Khim. 1953, 27, 1079.

(1081)	Yang, C.; Ma, P.; Tang, D.; Yin, Q.-X.; Zhao, C. Excess Molar Volume,Viscosity and Heat Capacity for the Binary Mixture of p-Xylene and Acetic Acid at Different Temperatute. Chin. J. Chem. Eng. 2002, 10, 604.

(1082)	Feng, L.; Ma, P.; Xiang, Z.; Yang, C. Determination of Densities and Viscosities for Binary Mixture of Diethyl Carbonate and P-Xylene at Atmospheric Pressure and Different Temperatures. Shiyou Huagong 2005, 34, 965.

(1083)	Di, Z.; Ma, P.; Xia, S.; Yang, T. Determination of Densities and Viscosities of P-Xylene-Acetic Acid-Water Ternary System. Shiyou Huagong 2006, 35, 851.

(1084)	Vogel, E.; Hendl, S. Vapor Phase Viscosity of Toluene and P-Xylene. Fluid Phase Equilib. 1992, 79, 313–326. https://doi.org/10.1016/0378-3812(92)85140-4.

(1085)	Lavochnik, A. I.; Solovey, R. L. Viscosity of Freons and Their Mixtures in Liquid State at Atmospheric Pressure. Kholod. Tekhn. 1980, 32–34.

(1086)	Kumagai, A.; Takahashi, S. Viscosity of Saturated Liquid Fluorocarbon Refrigerants from 273 to 353 K. Int. J. Thermophys. 1991, 12, 105–117. https://doi.org/10.1007/bf00506125.

(1087)	Benning, A. F.; Markwood, W. H. The Viscosities of “Freon” Refrigerants. ASHRAE J. 1939, 37, 243–247.

(1088)	Rivkin, S. L.; Levin, A. Ya.; Izrailevskij, L. B. Teplofiz. Svoistva Vesch. Mater.; 1971.

(1089)	Ivanchenko, S. I. Dynamic Viscosity Investigation of Freons of Methane and Ethane Rows. PhD Thesis, Odessa Technological Institute of Food Industry, 1974.

(1090)	Geller, V. Z.; Ivanchenko, S. I.; Kronberg, A. V. Study of Dynamic Viscosity Coefficient of Methane Type Freons. In Teplofiz. Svoistva Veshchestv. Mater.; Collect. Vol. No., V. A. Rabinovich, Ed., Standards Publ.: Moscow, 1975; pp 148–161.

(1091)	Geller, V. Z.; Karabanov, E. M.; Gunchuk, B. V.; Zakharzhevskiy, V. Y.; Lapardin, N. I. The Investigation of the Viscosity Coefficient of Some of the Liquid Gases About the Saturated Line. Gaz. Prom. 1976, 32–2.

(1092)	Karbanov, E. M. Investigation of the Dynamic Viscosity of Some Freons of Ethane Type and of the Bromide Freons. PhD Thesis, Grozny Petroleum Institute, 1978.

(1093)	Hovorka, F.; Geiger, F. E. Thermodynamic Prop. of Trifluorotrichloroethane and Difluorotetrachlo- Ethane. J. Am. Chem. Soc. 1933, 55, 4759–4761. https://doi.org/10.1021/ja01339a004.

(1094)	Ivanchenko, S. I. Viscosity of Triflourotricloroethane. Izv. Vyssh. Uchebn. Zaved., Neft Gaz 1972, 15, 82.

(1095)	Rasskazov, D. C.; Babikov, Yu. M.; Filatov, N. Ya. Teplofiz. Svoistva Veshch. Mater.; 1980.

(1096)	Geller, V. Z. Investigation of the Viscosity of Freons of the Methane, Ethane, and Propane Types. Summary of Experimental Data. In Teplofiz. Svoistva Veshchestv. Mater.; No. 15, Sychev, V. V., Ed., Standards Publ.: Moscow, 1980; pp 89–114.

(1097)	Arnemann, M.; Kruse, H. Liquid Viscosities of the Non-Azeotropic Binary Refrigerant Mixtures R22/R1 14, R22/R152A, R22/R142B. Measurements and Calculations. In Actes Congr. Int. Froid, 18th; Vol. 2, Int. Congr. Refrig., Saint-Hyacinthe: Que, 1991; pp 379–383.

(1098)	Bondar’, G. E. The Viscosity of Freons at Low Temperature. PhD Thesis, Odessa Technological Institute of Chemical Industry, 1984.

(1099)	Kronberg, A. V. Experimental and Theoretical Investigation of the Viscosity of Methane and Ethane Row’s Refrigerants. PhD Thesis, Azerbaijan Institute of Petroleum Industry, 1979.

(1100)	Awbery, J. H.; Griffiths, E. The Viscosities of Some Liquid Refrigerants. Proc. Phys. Soc. 1936, 48, 372–380.

(1101)	Zaloudik, P. Contribution to Viscosity Measurements with Aid of Hoppler’s Viscometer. Chem. Prum. 1962, 12, 81–83.

(1102)	Srichand, M.; Tirunarayanan, M. A.; Ramachandran, A. Studies of the Viscosity of Mixtures of R12 and R22 Vapors. ASHRAE J. 1970, 12, 61–66.

(1103)	Chaikovskii, V. F.; Geller, V.; Ivanchenko, S. I. Viscosity of Diflorodichloromethane. Izv. Vyssh. Uchebn. Zaved., Neft Gaz 1973, 16, 111–112.

(1104)	Alam, M. J.; Yamaguchi, K.; Hori, Y.; Kariya, K.; Miyara, A. Measurement of Thermal Conductivity and Viscosity of Cis-1-Chloro-2, 3, 3, 3-Tetrafluoropropene (R-1224yd (Z)). Int. J. Refrig. 2019, 104, 221–228.

(1105)	Matsuo, S.; Tanaka, Y.; Kubota, H.; Makita, T.; Kashiwagi, H. Thermophysical Properties of R123 (PVT Properties, Viscosity, and Thermal Conductivity). In Proc. Symp. Thermophys. Prop. 9th; Japan, 1988; p 231.

(1106)	Diller, D. E.; Aragon, A. S.; Laesecke, A. Measurements of the Viscosities of Saturated and Compressed Liquid Chlorodifluoromethane (R22). Int. J. Refrig. 1993, 16, 19–22.

(1107)	Nabizadeh, H.; Mayinger, F. Viscosity of Gaseous R123, R134a and R142b. In Proc. European Conference on Thermophysical Properties, 12th; Vienna, Austria, 1990.

(1108)	Nabizadeh, H.; Mayinger, F. Viscosity of Gaseous R123, R134a, and R142b. High Temp. - High Pressures 1992, 24, 221–230.

(1109)	Dowdell, D. C.; Matthews, G. P. Gas Viscosities and Intermolecular Interactions of Replacement Refrigerants HCFC 123 (2,2-Dichloro-1,1,1-Trifluoroethane), HCFC 124 (2- Chloro-1,1,1,2-Tetrafluoroethane) and HFC 134a (1,1,1,2-Tetrafl. J. Chem. Soc., Faraday Trans. 1993, 89, 3545–3552.

(1110)	Yokoyama, C.; Nishino, T.; Takahashi, M. Viscosity of Gaseous Mixtures of HFC-125 (Pentafluoroethane)+ HFC-134a (1, 1, 1, 2-Tetrafluoroethane) under Pressure. Fluid Phase Equilib. 2000, 174 (1–2), 231–240.

(1111)	Nakazawa, N.; Kawamura, M.; Sekiya, A.; Ootake, K.; Tamai, R.; Kurokawa, Y.; Murata, J. Liquid Viscosities of Fluorinated Dialkylethers. Nippon Reito Kyokai Ronbunshu 2002, 19, 33–41.

(1112)	Meng, X.; Wen, C.; Wu, J. Measurement and Correlation of the Liquid Viscosity of Trans-1-Chloro-3, 3, 3-Trifluoropropene (R1233zd (E)). J. Chem. Thermodyn. 2018, 123, 140–145.

(1113)	Hulse, R.; Singh, R.; Pham, H. Physical Properties of HFO-1234yf (Paper No. 178). In 3rd Int. Inst. Refrigeration Conference on Thermophysical Properties and Transfer Processes of Refrigerants; Boulder (CO), June, 2009; pp 1–7.

(1114)	Meng, X.; Qiu, G.; Wu, J.; Abdulagatov, I. M. Viscosity Measurements for 2,3,3,3-Tetrafluoroprop-1-Ene (R1234yf) and Trans-1,3,3,3-Tetrafluoropropene (R1234ze(E)). J. Chem. Thermodyn. 2013, 63, 24–30. https://doi.org/10.1016/j.jct.2013.03.013.

(1115)	Grebenkov, A. J.; Hulse, R.; Pham, H.; Singh, R. Physical Properties and Equation of State for Trans-1,3,3,3-Tetrafluoropropene. In 3rd Int. Inst. Refrigerantion Conference on Thermophysical Properties and Transfer Processes of Refrigerants; Boulder, CO, pp Paper No. 191 Pages 1-19, 2009.

(1116)	Diller, D. E.; Peterson, S. M. Measurements of the Viscosities of Saturated and Compressed Fluid 1-Chloro- 1,2,2,2-Tetrafluoroethane (R124) and Pentafluoroethane (R125) at Temperatures between 120 and 420 K. Int. J. Thermophys. 1993, 14, 55–66. https://doi.org/10.1007/bf00522661.

(1117)	Ripple, D.; Matar, O. Viscosity of the Saturated Liquid Phase of Six Halogenated Compounds and Three Mixtures. J. Chem. Eng. Data 1993, 38 (4), 560–564.

(1118)	Assael, M. J.; Polimatidou, S. K. Measurements of the Viscosity of Refrigerants in the Vapor Phase. Int. J. Thermophys. 1997, 18, 353–366. https://doi.org/10.1007/bf02575166.

(1119)	Oliveira, C. M. B. P.; Wakeham, W. A. The Viscosity of Liquid R134a. Int. J. Thermophys. 1993, 14, 33–44. https://doi.org/10.1007/bf00522659.

(1120)	Heide, R.; Schenk, J. Betimmung Der Transportgroben von HFKW; 708; Forschungsrat Kaltetechnik E.V. Betimmung der Transportgroben von HFKW: Dresden, 1996.

(1121)	Wilson, L. C.; Wilding, W. V.; Wilson, G. M.; Rowley, R. L.; Felix, V. M.; Chisolm-Carter, T. Thermophysical Properties of HFC-125. Fluid Phase Equilib. 1992, 80, 167–177. https://doi.org/10.1016/0378-3812(92)87065-u.

(1122)	Ripple, D.; Defibaugh, D. Viscosity of the Saturated Liquid Phase of Three Fluorinated Ethanes: R152a, R143a, and R125. J. Chem. Eng. Data 1997, 42, 360–364. https://doi.org/10.1021/je960284w.

(1123)	Avelino, H. M. T.; Fareleira, J. M. N. A.; Oliveira, C. M. B. P. Viscosity of Compressed Liquid 1,1,1-Trifluoroethane (HFC-143a) and Pentafluoroethane (HFC-125). J. Chem. Eng. Data 2006, 51, 1672–1677. https://doi.org/10.1021/je0601356.

(1124)	Oliveira, C. M. B. P.; Wakeham, W. A. Viscosity of R134a, R32, and R125 at Saturation. Int. J. Thermophys. 1999, 20, 365–373. https://doi.org/10.1023/a:1022640617694.

(1125)	Takahashi, M.; Shibasaki-Kitakawa, N.; Yokoyama, C. Viscosity of Gaseous HFC-125 (Pentafluoroethane) under High Pressures. Int. J. Thermophys. 1999, 20, 445–453. https://doi.org/10.1023/a:1022648819511.

(1126)	Reed, J. F.; Rabinovitch, B. S. Viscosities of Fluorinated Methyl Bromides and Chlorides. Ind. Eng. Chem. Chem. Eng. Data Ser. 1957, 2, 75. https://doi.org/10.1021/i460002a021.

(1127)	Ivanchenko, S. I.; Bondar’, G. E.; Geller, V. Z. Experimental Investigation of Viscosity of Freon-13 and 14. In Kholod. Tekh. Tekhnol., Collect. Vol.; No. 19, Tekhnika: Kiev, 1974; pp 98–102.

(1128)	Takahashi, M.; Takahashi, S.; Iwasaki, H. Viscosity of Gaseous Chlorotrifluoromethane (R 13) under Pressure. J. Chem. Eng. Data 1985, 30, 10–14. https://doi.org/10.1021/je00039a004.

(1129)	Chajkovskij, V. F.; Geller, V. Z.; Bondar, E. G. The Viscosity of R13 at Low Temperatures. Kholod. Tekh. Tekhnol. 1977, 66–72.

(1130)	Diller, D. E.; Van Poolen, L. J. Measurement of Viscosities of Saturated and Compressed Fluid Chlorotrifluoromethane (R13). Cryogenics 1989, 29, 1063–1066.

(1131)	Alam, M. J.; Islam, M. A.; Kariya, K.; Miyara, A. Measurement of Thermal Conductivity and Correlations at Saturated State of Refrigerant Trans-1‑chloro‑3, 3, 3-Trifluoropropene (R-1233zd (E)). Int. J. Refrig. 2018, 90, 174–180.

(1132)	Han, L.-Z.; Zhu, M.-S.; Li, X.-Y.; Luo, D. Viscosity of Saturated Liquid for 1,1,1,2-Tetrofluoroethane. J. Chem. Eng. Data 1995, 40, 650–652. https://doi.org/10.1021/je00019a026.

(1133)	Huber, M. L.; Laesecke, A.; Perkins, R. A. Model for the Viscosity and Thermal Conductivity of Refrigerants, Including a New Correlation for the Viscosity of R134a. Ind. Eng. Chem. Res. 2003, 42 (13), 3163–3178. https://doi.org/10.1021/ie0300880.

(1134)	Pasekov, M. F.; Ustjushanin, E. E. Viscosity of R134a in Gas Phase. Teplofiz. Vys. Temp. 1994, 32, 630–633.

(1135)	Shibasaki-Kitakawa, N.; Takahashi, M.; Yokoyama, C. Viscosity of Gaseous HFC-134a (l,l,l,2-Tetrafluoroethane) Under High Pressures. Int. J. Thermophys. 1998, 19, 1285–1295. https://doi.org/10.1023/a:1021919215793.

(1136)	Ruvinskii, G. Y.; Lavrenchenko, G. K.; Il’yushenko, S. V. Thermophysical Properties of R-134a. Kholod. Tekhn. 1990, 67, 20–26.

(1137)	Lavrenchenko, G. K.; Ruvinskii, G. Y.; Iljushenko, S. V.; Kanaev, V. V. Thermophysical Properties of Refrigerant R134a. Int. J. Refrig. 1992, 15, 386–392.

(1138)	Laesecke, A.; Bair, S. High-Pressure Viscosity Measurements of 1,1,1,2-Tetrafluoroethane. Int. J. Thermophys. 2011, 32, 925–941. https://doi.org/10.1007/s10765-011-0978-y.

(1139)	Comunas, M. J. P.; Baylaucq, A.; Quinones-Cisneros, S. E.; Zeberg-Mikkelsen, C. K.; Boned, C.; Fernandez, J. Viscosity Measurements and Correlations for 1,1,1,2-Tetrafluoroethane (HFC-134a) up to 140 MPa. Fluid Phase Equilib. 2003, 210, 21–32. https://doi.org/10.1016/s0378-3812(03)00158-4.

(1140)	Meng, X.; Zhang, J.; Wu, J. Compressed Liquid Viscosity of 1,1,1,3,3-Pentafluoropropane (R245fa) and 1,1,1,3,3,3-Hexafluoropropane (R236fa). J. Chem. Eng. Data 2011, 56, 4956–4964. https://doi.org/10.1021/je200854k.

(1141)	Wilhelm, J.; Vogel, E. Gas-Phase Viscosity of the Alternative Refrigerant R134a at Low Densities. Fluid Phase Equilib. 1996, 125, 257–266. https://doi.org/10.1016/s0378-3812(96)03082-8.

(1142)	Raw, C. J. G.; Tang, H. Viscosity and Diffusion Coefficients of Gaseous Sulfer Hexafluoride-Carbon Tetrafluoride Mixtures. J. Chem. Phys. 1963, 39, 2616–2618.

(1143)	Genot, B. Thermal Effusion and Viscosity of Gaseous Tetrafluoromethane. J. Chim. Phys. Phys.-Chim. Biol. 1970, 67, 1322–1331.

(1144)	Filatov, N. Ya. Experimental Investigation of Viscosity of Refrigerants-14, 21, 23. PhD Thesis, Moscow Energy Institute, 1975.

(1145)	Kochubey, V. F.; Moin, F. B. Determination of Gas-Kinetic Diameters of Fluoromethane Molecules. Zh. Fiz. Khim. 1978, 52, 15–17.

(1146)	Rasskazov, D. S.; Babikov, Yu. M.; Filatov, N. Ya. Experimental Investigation of the Viscosity of Freon 23. In Teplofiz. Svoistva Veshchestv Mater.; Collect. Vol. No. 8, V. A. Rabinovich, Ed., Standards Publ.: Moscos, 1975; pp 142–147.

(1147)	Kumagai, A.; Takahashi, S. Saturated Liquid Viscosities of Environmentally Acceptable Fluorocarbons. In Proc. Symp. Thermophys. Prop., 11th; Tokyl, Japan, 1990; pp 119–122.

(1148)	Kumagai, A.; Takahashi, S. Saturated Liquid Viscosities and Densities of Environmentally Acceptable Hydrochlorofluorocarbons (HCGCs). Int. J. Thermophys. 1993, 14, 339–342. https://doi.org/10.1007/bf00507818.

(1149)	Mears, W. H.; Stahl, R. F.; Orfeo, S. R.; Shair, R. C.; Kells, L. F.; Thompson, W.; McCann, H. Thermodynamic Properties of Halogenated Ethanes and Ethylenes. Ind. Eng. Chem. 1955, 47, 1449.

(1150)	Konrad, S. I. Viscosity of Binary and Multicomponent Refrigerant Mixtures. PhD Thesis, Odessa Technological Institute of Food Industry, 1985.

(1151)	Takahashi, M.; Yokoyama, C.; Takahashi, S. Viscosities of Gaseous R13B1, R142b, and R152a. J. Chem. Eng. Data 1987, 32, 98–103. https://doi.org/10.1021/je00047a027.

(1152)	Wang, X.; Wu, J.; Liu, Z.-G. Viscosity of R143a in Gaseous Phase. J. Eng. Thermophys. 2009, 30, 4;1455-1458.

(1153)	Stern, K. H.; Martell, A. E. Thermodynamics of Ion Pair Formation in Some Non-Aqueous Solvents. J. Am. Chem. Soc. 1955, 77, 1983–1986. https://doi.org/10.1021/ja01612a083.

(1154)	Alluraiah, G.; Ramanjaneyulu, K.; Krishnaiah, A. A Study of Interactions in Binary Mixtures of 1-Heptanol with Some Chlorinated Ethanes and Ethenes from Viscosity Measurements at 303.15 K. Phys. Chem. Liq. 1989, 19, 181.

(1155)	Malhotra, R.; Price, W. E.; Woolf, L. A.; Easteal, A. J. Thermodynamic and Transport Properties of 1,2-Dichloroethane. Int. J. Thermophys. 1990, 11, 835–861. https://doi.org/10.1007/bf00503578.

(1156)	Krishnaiah, A.; Surendranath, K. N. Densities, Speeds of Sound, and Viscosities of Mixtures of Oxolane with Chloroethanes and Chloroethenes. J. Chem. Eng. Data 1996, 41, 1012–1014. https://doi.org/10.1021/je950304e.

(1157)	Zhang, H.-L.; Han, S.-J. Viscosmetric and Volumetric Studies on Binary Mixtures of 1,2-Dichloroethane and Chlorinated Methanes or Their Binary Equimolar Mixtures at 303.15 K. Fluid Phase Equilib. 1997, 140, 233–244. https://doi.org/10.1016/s0378-3812(97)00193-3.

(1158)	Mussari, L.; Postigo, M.; Lafuente, C.; Royo, F. M.; Urieta, J. S. Viscosity Measurements for the Binary Mixtures of 1,2-Dichloroethane or 1,2-Dibromoethane with Isomeric Butanols. J. Chem. Eng. Data 2000, 45, 86–91. https://doi.org/10.1021/je990211n.

(1159)	Oswal, S. L.; Patel, I. N.; Modi, P. S.; Barad, S. A. Viscosities and Excess Molar Volumes of Binary Mixtures of Alkyl Acetates with Di-, Tri-, and Tetrachloroethane. Int. J. Thermophys. 2000, 21, 681. https://doi.org/10.1023/a:1006689418706.

(1160)	Aznarez, S.; Katz, M.; Arancibia, E. L. Thermophysical Properties of the Ternary System 2-Methoxyethanol + Acetonitrile + 1,2- Dichloroethane, and the Binary Systems at 25deg C. J. Solution Chem. 2002, 31, 639–651.

(1161)	Sathyanarayana, B.; Ranjithkumar, B.; Jyostna, T. S.; Satyanarayana, N. Densities and Viscosities of Binary Liquid Mixtures of N-Methylacetamide with Some Chloroethanes and Chloroethenes at T = 308.15 K. J. Chem. Thermodyn. 2007, 39, 16–21. https://doi.org/10.1016/j.jct.2006.06.009.

(1162)	Bhatia, S. C.; Bhatia, R.; Dubey, G. P. Studies on Transport and Thermodynamic Properties of Binary Mixtures of Hexan-1-Ol with Halogenated Compounds at 293.15 K. J. Chem. Eng. Data 2009, 54, 3303–3306. https://doi.org/10.1021/je900278v.

(1163)	Ranjbar, S.; Fakhri, K.; Ghasemi, J. B. Densities and Viscosities of (1-Propanol + 1,2-Dichloroethane), (1-Propanol + Benzaldehyde), (Benzaldehyde + 1,2-Dichloroethane), and (1-Propanol + 1,2-Dichloroethane + Benzaldehyde) Mixtures from T = 288.15 K to 313.15 K. J. Chem. Eng. Data 2009, 54, 3284–3290. https://doi.org/10.1021/je900212d.

(1164)	Syeda, A. B.; Koppula, A. J.; Boodida, S.; Nallani, S. Excess Molar Volumes and Sound Speed in (Phenylacetonitrile + 1,2-Dichloroethane), (Phenylacetonitrile + 1,1,2-Trichloroethane), (Phenylacetonitrile + 1,1,2,2-Tetrachloroethane), (Phenylacetonitrile + Trichloroethene), and (Phenylacetonitrile + Tetrachloroethene) at Temperatures of (303.15, 308.15, and 313.15) K. J. Chem. Eng. Data 2010, 55, 1405–1410. https://doi.org/10.1021/je900570e.

(1165)	vanderGulik, P. S. Viscosity of Saturated R152a Measured with a Vibrating Wire Viscometer. Int. J. Thermophys. 1995, 16, 867–876. https://doi.org/10.1007/bf02093469.

(1166)	Lapardin, N. I. Viscosity of Difluooroethane. Izv. Vyssh. Uchebn. Zaved., Neft Gaz 1982, 25, 24–63.

(1167)	Lapardin, N. I. Investigation of the Viscosity of Freons R 152A and R 218. PhD Thesis, Odessa Technological Institute of Chemical Industry, 1983.

(1168)	Wang, W.; Li, G.; He, Z.; Liu, Z. Experimental Study for Gaseous Viscosities with Oscillating-Disk Viscometer. Xi’an Jiaotong Daxue Xuebao 1997, 31, 10–14.

(1169)	Geller, V. Z.; Ivanchenko, S. I.; Peredriy, V. G. Experimental Investigation of Dynamic Viscosity and Thermal Conductivity Coeficients of Difluorudichloromethane. Izv. Vyssh. Uchebn. Zaved., Neft Gaz 1973, 61–65.

(1170)	Butyrskaya, S. T. Investigation of the Dynamic Viscosity Coefficient of Freon-22. In Teplofiz. Svoistva Zhidkostey; Collect. Vol., Nauka: Moscow, 1970; pp 73–76.

(1171)	Takahashi, M.; Takahashi, S.; Iwasaki, H. Viscosity of Gaseous Chlorodifluoromethane(R-22). Kagaku Kogaku Ronbunshu 1983, 9, 482–484.

(1172)	Zhang, Z.; Liu, Z.-G.; Wang, W. Gaseous Viscosity Measurement for Several Refrigerant Mixtures. J. Chem. Eng. Data 2002, 47, 383–387. https://doi.org/10.1021/je015502m.

(1173)	Chaikovskiy, V. F.; Geller, V. Z.; Gorykin, S. F.; Artamonov, S. D.; Bondar’, G. E.; Ivanchenko, S. I.; Lenskiy, L. R.; Peredriy, V. G. Comprehensive Investigation of the Thermophysical Properties of Most Important and Promising Regrigerants in Liquid and Gaseous Phase. In Teplofiz. Svoistva Zhidkostey, Collect. Vol.; Nauka: Moscow, 1976; pp 108–117.

(1174)	Hongo, M.; Takahashi, S. Viscosity of Chlrodifluoromethane at High Pressure. J. Chem. Eng. Jpn. 1982, 15, 155–158.

(1175)	Laesecke, A.; Hafer, R. F. Viscosity of Fluorinated Propane Isomers. 2. Measurements of Three Compounds and Model Comparisons. J. Chem. Eng. Data 1998, 43, 84–92. https://doi.org/10.1021/je970186q.

(1176)	Liu, X.-J.; Shi, L.; Han, L.-Z.; Zhu, M.-S. Liquid Viscosity of 1,1,1,2,3,3,3-Heptafluoropropane (HFC-227ea) along the Saturation Line. J. Chem. Eng. Data 1999, 44, 688–692. https://doi.org/10.1021/je980098l.

(1177)	Tang, X.; Zhang, J.; Meng, X.; Wu, J. Viscosity and Density Measurements for Liquid 1,1,1,2,3,3,3,-Heptafluoropropane(HFC-227ea). J. Eng. Thermophys. 2012, 33, 191–194.

(1178)	McCoubrey, J. C.; Singh, N. M. Intermolecular Forces in Quasi-Spherical Molecules. Trans. Faraday Soc. 1957, 53, 877–883.

(1179)	Tomassini, F.; Levi, A. C.; Scoles, G. Viscosity and Thermal Conductivity of Polar Gases in an Electric Field. Physica (Amsterdam) 1970, 49, 299–341.

(1180)	Rasskazov, D. C.; Babikov, Yu. M.; Filatov, N. Ya. Experimental Investigation of Viscosity of Refrigerant R-23. Tr. Mosk. Energ. Inst. 1974, 62–69.

(1181)	Sagaidakova, N. G. Experimental Investigation of Viscosity of Freons-12B1, 13B1, and 502 in Wide Region of State Parameters. PhD Thesis, Leningrad Technological Institute of Refrigeration Industry, 1977.

(1182)	Yokoyama, C.; Takahashi, M. Viscosity of CHF3 in the Critical Region. Int. J. Thermophys. 1997, 18, 1369–1385. https://doi.org/10.1007/bf02575340.

(1183)	Laesecke, A.; Defibaugh, D. R. Viscosity of 1,1,1,2,3,3-Hexafluoropropane and 1,1,1,3,3,3- Hexafluoropropane at Saturated-Liquid Conditions from 262 K to 353 K. J. Chem. Eng. Data 1996, 41, 59–62. https://doi.org/10.1021/je950206t.

(1184)	Takahashi, M.; Shibasaki-Kitakawa, N.; Yokoyama, C.; Takahashi, S. Gas Viscosity of Difluoromethane from 298.15 to 423.15 K and up to 10 Mpa. J. Chem. Eng. Data 1995, 40, 900–902. https://doi.org/10.1021/je00020a036.

(1185)	Geller, V. Z.; Paulaitis, M. E.; Bivens, D. B.; Yokozeki, A. Viscosities of HFC-32 and HFC-32/Lubricant Mixtures. Int. J. Thermophys. 1996, 17, 75–83. https://doi.org/10.1007/bf01448211.

(1186)	Badylkes, I. S. 1960.

(1187)	McGovern, E. W. Chlorohydrocarbon Solvents. Ind. Eng. Chem. 1943, 35, 1230–1239.

(1188)	Kessel’man, P. M.; Porichanskii, E. G.; Romanov, V. K.; Ponomareva, O. P.; Svetlichnyi, P. I.; Zhelezny, V. P.; Slyusarev, V. V.; Karbanov, E. M. Complex Study of the Thermophysical Properties of Refrigerant S318. Teplofiz. Svoistva Veshchestv Mater. 1977, 4–10.

(1189)	Sekiya, A.; Misaki, S. The Potential of Hydrofluoroethers to Replace CFCs, HCFCs, and PFCs. J. Fluorine Chem. 2000, 101, 215–221.

(1190)	Hu, X.; Meng, X.; Wei, K.; Li, W.; Wu, J. Compressed Liquid Viscosity Measurements of HFE-7000, HFE-7100, HFE-7200, and HFE-7500 at Temperatures from (253 to 373) K and Pressures up to 30 MPa. J. Chem. Eng. Data 2015, 60, 3562–3570. https://doi.org/10.1021/acs.jced.5b00499.

(1191)	Ulybin, S. A.; Makarushkin, V. I. Viscosity of Xenon at Temperatures from 170 to 1300 K and at Pressures up to 1000 Bar. Teplofiz. Vys. Temp. 1977, 15, 509–514.

(1192)	Ueda, K.; Kigoshi, K. The Viscosity of the Gaseous Heaxfluorides of Sulfur, Selenium, and Tellurium. J. Inorg. Nucl. Chem. 1974, 36, 989–992.

(1193)	Grigor’ev, V. A.; Keramidi, A. S.; Grachev, V. K.; Rastorguev, Yu. L. Experimental Investigation of Viscosity of Sulfur-Hexafluroide. Therm. Eng. 1978, 24, 66–68.

(1194)	Hafer, R. F. Torsional Crystal Viscometry: Wide Ranging Resonance Measurements and Feasibility Study of Transient-Decay Measurements. Master’s Thesis, University of Colorado, 1999.

(1195)	Wilhelm, J.; Seibt, D.; Bich, E.; Vogel, E.; Hassel, E. Viscosity Measurements on Gaseous Sulphur Hexafluoride. J. Chem. Eng. Data 2005, 50, 896–906. https://doi.org/10.1021/je049612f.

(1196)	Grigor’ev, B. A.; Keramidi, A. S.; Grachev, V. K.; Rastorguev, Yu. L. Experimental Study of the Viscosity of Sulfur Hexafluoride. Teploenergetika 1977, 85–87.

(1197)	Trautz, M.; Weizel, W. Determination of Viscosity of Sulphur Dioxide and Its Mixtures with Hydrogen. Ann. Phys. (Leipzig) 1925, 78, 305–369.

(1198)	Smith, C. J. The Viscosity and Molecular Dimensions of Sulfur Dioxide. Philos. Mag. Ser. 6 1922, 44, 508.

(1199)	Shimotake, H.; Thodos, G. Viscosity of Sulfur Dioxide at 200OC. for Pressures Up to 3500 P.S.I. J. Chem. Eng. Data 1963, 8, 88–90. https://doi.org/10.1021/je60016a026.

(1200)	Chang, K. C.; Hesse, R. J.; Raw, C. J. G. Transport Properties of Polar Gas Mixtures SO2+ SO2F2 Mixtures. Trans. Faraday Soc. 1970, 66, 590–596. https://doi.org/10.1039/tf9706600590.

(1201)	Chakraborti, P. K.; Gray, P. Viscosities of Gaseous Mixtures Containing Polar Gases: More than One Polar Constituent. Trans. Faraday Soc. 1966, 62, 1769–1775. https://doi.org/10.1039/tf9666201769.

(1202)	Chakraborti, P. K.; Gray, P. Viscosities of Gaseous Mixtures Containing Polar Gases Polar Constituent. Trans. Faraday Soc. 1965, 61, 2422.

(1203)	Stakelbeck, H. Z. Gesamte Kalte-Ind. 1933, 40, 33.

(1204)	Lucatu, E. Relation between Vapor Pressure, Viscosity and Molecular Association. C. R. Hebd. Seances Acad. Sci. 1938, 207, 1403–1405.

(1205)	Adams, H. E.; Rogers, H. E. The Viscosity of Solutions of Sulfur Dioxide in Organic Liquids. J. Am. Chem. Soc. 1939, 61, 112–115. https://doi.org/10.1021/ja01870a038.

(1206)	Cupp, S. B.; Rogers, H. E. The Viscosities of Solutions of Surfur Dioxide in Organic Liquids. J. Am. Chem. Soc. 1939, 61, 3353–3354. https://doi.org/10.1021/ja01267a035.

(1207)	Scheuer, O. Anz. Akad. Wiss. Math.-Natur. Kl. 1911, 48, 304–308.

(1208)	Clifford, A. A.; Gray, P.; Johns, A. I.; Scott, A. C.; Watson, J. T. R. Thermal Conductivity of Argon, Nitrogen, and Hydrogen between 300 and 400 K and up to 25 MPa. J. Chem. Soc., Faraday Trans. 1 1981, 77, 2679–2691. https://doi.org/10.1039/f19817702679.

(1209)	Salomon, M. Electrolyte Solvation in Aprotic Solvents. J. Power Src. 1989, 26, 9–21. https://doi.org/10.1016/0378-7753(89)80010-2.

(1210)	Iwasaki, H.; Takahashi, M. The Viscosity of Gaseous Sulfur Dioxide. Bull. Chem. Soc. Jpn. 1975, 48, 988–992. https://doi.org/10.1246/bcsj.48.988.

(1211)	Litkenhous, E. E.; Van Arsdale, J. D.; Hutchison, I. W. The Physical Properties of the Ternary System Butyl Alcohol-Ethyl Acetate- Toluene. J. Phys. Chem. 1940, 44, 377–388.

(1212)	Xu, Z.; Meng, X.; Wu, J.; Liu, Z. Development of a Vibrating-Wire Viscometer and Measurement of Saturated Liquid Viscosity of Methyl Tert-Butyl Ether. J. Eng. Thermophys. 2007, 28, 4;189.

(1213)	Il’in; Ivanov, V. N. Measurement of Absolute Internal-Friction Coefrficients by the Pendulum Viscometer Method. Kolloid Z. 1935, 71, 267.

(1214)	Zeidler, O.; Schuster. Farben-Ztg. 1942, 47, 318.

(1215)	Akhundov, T. S.; Ismail-Zade, Sh. M.; Tairov, A. D. Experimental Investigation of the Viscosity of Toluene at High Pressure and Temperature. Izv. Vyssh. Uchebn. Zaved., Neft Gaz 1970, 79.

(1216)	Katz, M.; Lobo, P. W.; Minano, A. S.; Solimo, H. Viscosities, Densities, and Refractive Indices of Binary Liquid Mixt. Can. J. Chem. 1971, 49, 2605–2609. https://doi.org/10.1139/v71-429.

(1217)	Pugachevich, N. N.; Martirosyan, A. L.; Kamalyan, R. M.; Lavygin, I. A. Viscosity and Density of Solution of Polydimethylsiloksane in Toluene. Zh. Fiz. Khim. 1975, 49, 130–133.

(1218)	Akhundov, T. S.; Abdullaev, F. G.; Ramazanzade, M. G. Experimental Study of the Viscosity of Toluene in Gaseous Phase and in the Critical Region. Izv. Vyssh. Uchebn. Zaved., Neft Gaz 1983, 53–54.

(1219)	Ritzoulis, G.; Papadopoulos, N.; Jannakoudakis, D. Densities, Viscosities, and Dielectric Constatns of Acetonitrile + Toluene at 15, 25, and 35 Deg. C. J. Chem. Eng. Data 1986, 31, 146–148. https://doi.org/10.1021/je00044a004.

(1220)	Byers, C. H.; Williams, D. F. Visosities of Pure Polyatomic Hydrocarbons. J. Chem. Eng. Data 1987, 32, 344–348. https://doi.org/10.1021/je00049a018.

(1221)	Singh, R. P.; Sinha, C. P.; Das, J. C.; Ghosh, P. Viscosity and Density of Ternary Mixtures of Toluene, Bromobenzene, 1-Hexanol and 1-Octanol. J. Chem. Eng. Data 1990, 35, 93–97. https://doi.org/10.1021/je00059a025.

(1222)	Krall, A. H.; Sengers, J. V.; Kestin, J. Viscosity of Liquid Toluene at Temperatures from 25 to 150.Degree.C and at Pressures up to 30 MPa. J. Chem. Eng. Data 1992, 37, 349–355. https://doi.org/10.1021/je00007a021.

(1223)	Dymond, J. H.; Glen, N. F.; Isdale, J. D.; Pyda, M. The Viscosity of Liquid Toluene at Elevated Pressures. Int. J. Thermophys. 1995, 16, 877–882. https://doi.org/10.1007/bf02093470.

(1224)	Assael, M. J.; Dalouti, N. K.; Polimatidou, S. The Viscosity of Toluene in the Temperature Range from 210 to 370 K at Pressures up to 30 Mpa. Int. J. Thermophys. 1999, 20, 1367–1377. https://doi.org/10.1023/a:1021428819290.

(1225)	Harris, K. R. Temperature and Density Dependence of the Viscosity of Toluene. J. Chem. Eng. Data 2000, 45, 893–897. https://doi.org/10.1021/je000024l.

(1226)	Roos, M.; Bart, H.-J. Extraction of Acetic Acid with Tri-n-Octylamine: Physical Properties and Phase Equilibria. J. Chem. Eng. Data 2001, 46, 1198–1202. https://doi.org/10.1021/je000313+.

(1227)	Akhtar, S.; Hossain, K. M. S.; Saleh, M. A. VISCOSITY AND THERMODYNAMICS OF VISCOUS FLOW FOR THE SYSTEMS OF ISOMERIC PENTANOLS WITH TOLUENE. Phys. Chem. Liq. 2002, 40, 435–448.

(1228)	Cruz, R. d. C.; Martins, R. J.; Cardoso, M. E.; Barcia, O. E. Measurement of the Viscosity of Binary and Ternary Systems Containing Benzaldehyde, Toluene, and Benzyl Alcohol at 293.15 K and 0.1 MPa. J. Chem. Eng. Data 2002, 47, 927–931. https://doi.org/10.1021/je010327v.

(1229)	Rattan, V. K.; Kapoor, S.; Tochigi, K. Viscosities and Densities of Binary Mixtures of Toluene with Acetic Acid and Propionic Acid at (293.15, 303.15, 313.15, and 323.15) K. J. Chem. Eng. Data 2002, 47, 1182–1184. https://doi.org/10.1021/je020018k.

(1230)	Caetano, F. J. P.; Correia da Mata, J. L.; Fareleira, J. M. N. A.; Oliveira, C. M. B. P.; Wakeham, W. A. Viscosity Measurements of Liquid Toluene at Low Temperatures Using a Dual Vibrating-Wire Technique. Int. J. Thermophys. 2004, 25, 1–11. https://doi.org/10.1023/b:ijot.0000022325.10161.39.

(1231)	Kandil, M. E.; Marsh, K. N.; Goodwin, A. R. H. Vibrating Wire Viscometer with Wire Diameters of (0.05 and 0.15) Mm: Results for Methylbenzene and Two Fluids with Nominal Viscosities at T = 298 K and p = 0.01 MPa of (14 and 232) MPa*s at Temperatures between (298 and 373) K and Pressures below 40 MPa. J. Chem. Eng. Data 2005, 50, 647–655. https://doi.org/10.1021/je049636m.

(1232)	Nikam, P. S.; Kharat, S. J. Density and Viscosity Studies of Binary Mixtures of N,N-Dimethylformamide with Toluene and Methyl Benzoate at (298.15, 303.15, 308.15, and 313.15) K. J. Chem. Eng. Data 2005, 50, 455–459. https://doi.org/10.1021/je040012q.

(1233)	Hasan, M.; Shirude, D. F.; Hiray, A. P.; Kadam, U. B.; Sawant, A. B. Densities, Viscosities, and Speed of Sound Studies of Binary Mixtures of Methylbenzene with Heptan-1-Ol, Octan-1-Ol, and Decan-1-Ol at (303.15 and 313.15) K. J. Chem. Eng. Data 2006, 51, 1922–1926. https://doi.org/10.1021/je0602519.

(1234)	Tsierkezos, N. G.; Filippou, A. C. Thermodynamic Investigation of N,N-Dimethylformamide/Toluene Binary Mixtures in the Temperature Range from 278.15 to 293.15 K. J. Chem. Thermodyn. 2006, 38, 952–961. https://doi.org/10.1016/j.jct.2005.10.008.

(1235)	Hasan, M.; Shirude, D. F.; Hiray, A. P.; Kadam, U. B.; Sawant, A. B. Densities, Viscosities and Ultrasonic Velocity Studies of Binary Mixtures of Toluene with Heptan-1-Ol, Octan-1-Ol and Decan-1-Ol at 298.15 and 308.15 K. J. Mol. Liq. 2007, 135, 32–37. https://doi.org/10.1016/j.molliq.2006.10.012.

(1236)	Alonso, L.; Arce, A.; Francisco, M.; Soto, A. Liquid-Liquid Equilibria for [C8mim][NTf2] + Thiophene + 2,2,4-Trimethylpentane or + Toluene. J. Chem. Eng. Data 2008, 53, 1750–1755. https://doi.org/10.1021/je800071q.

(1237)	Narendra, K.; Srinivasu, C.; Kalpana, C.; Narayanamurthy, P. Excess Thermo Dynamical Parameters of Binary Mixtures of Toluene and Mesitylene with Anisaldehyde Using Ultrasonic Technique at Different Temperatures. J. Therm. Anal. Calorim. 2012, 107, 25–30. https://doi.org/10.1007/s10973-011-1521-x.

(1238)	Assael, M. J.; Mylona, S. A Novel Vibrating-Wire Viscometer for High-Viscosity Liquids at Moderate Pressures. J. Chem. Eng. Data 2013, 58, 993–1000. https://doi.org/10.1021/je301306e.

(1239)	Vijayalakshmi, D.; Rao, C. N.; Gowrisankar, M.; Sivakumar, K.; Venkateswarlu, P. Densities, Viscosities and Speeds of Sound of Binary Mixtures of Ethyl Benzoate with Toluene, and Isomeric Chlorotoluenes at Different Temperatures. J. Mol. Liq. 2014, 197, 272–286. https://doi.org/10.1016/j.molliq.2014.05.015.

(1240)	Bajic, D. M.; Zivkovic, E. M.; Jovanovic, J.; Serbanovic, S. P.; Kijevcanin, M. L. Experimental Measurements and Modelling of Volumetric Properties, Refractive Index and Viscosity of Binary Systems of Ethyl Lactate with Methyl Ethyl Ketone, Toluene and n-Methyl-2-Pirrolidone at 288.15 323.15 K and Atmospheric Pressure. New UNIFAC VISCO and ASOG VISCO Interaction Parameters. Fluid Phase Equilib. 2015, 399, 50–65. https://doi.org/10.1016/j.fluid.2015.04.017.

(1241)	Kerimov, A. M.; Agaev, N. A.; Abaszade, A. Experimental Study of the Viscosity of Water at Temperatures from 100 to 275 C and at High Pressures. Teploenergetika 1969, 87–90.

(1242)	Rivkin, S. L.; Levin, A. Ya.; Izrailevskii, L. B. Experimental Study of the Viscosity of Water at Temperatures from 250 to 375 and at Pressures up to 50 MPa. Teploenergetika 1970, 79–81.

(1243)	Kingham, D. J.; Adams, W. A.; McGuire, M. J. Viscosity Measurements of Water in Regioon of Its Maximum Density. J. Chem. Eng. Data 1974, 19, 1–3. https://doi.org/10.1021/je60060a028.

(1244)	Assael, M. J.; Polimatidou, S.; Wakeham, W. A. The Viscosity of Liquid Water at Pressures up to 32 MPa. Int. J. Thermophys. 1993, 14, 795–803. https://doi.org/10.1007/bf00502108.

(1245)	Jones, H. C.; Davis, P. B. The Freezing-Point Lowering, Conductivity, and Viscosity of Solutions of Certain Electrolytes in Water, Methyl Alcohol, Ethyl Alcohol, Acetone, and Glycerol, and in Mixtures of These Solvents with On; 180; Carnegie Inst. Monograph: Washington, 179-1, 1913.

(1246)	Bingham; Jackson. Bur. Stand. (U. S.), Bull. 1918, 14, 59.

(1247)	Bhagwat, W. V.; Mandloi, K. Application of Mixture Law to Rheochor. J. Indian Chem. Soc. 1946, 23, 349–352.

(1248)	Babak, S. F. Viscosity and Density of Binary Mixtures of Nicotine I. Zh. Obshch. Khim. 1949, 19, 1604.

(1249)	LeBel, R. G.; Goring, D. A. I. Density, Viscosity, Refractive Index, and Hygroscopicity of Mixtures of Water and Dimethyl Sulfoxide. J. Chem. Eng. Data 1962, 7, 100–101. https://doi.org/10.1021/je60012a032.

(1250)	Wallace, W. J.; Mathews, A. L. Densities, Refractive Indeices, Molar Refractions, and Viscosities of Ethylene Glycol Dimethyl Ether-Water Solutions at 25 C. J. Chem. Eng. Data 1963, 8, 496–498. https://doi.org/10.1021/je60019a006.

(1251)	Wallace, W. J.; Mathews, A. L. Density, Refractive Indices, Molar Refractions, and Viscosities of Diethylene Glycol Dimethyl Ether-Water Solutions at 25 C. J. Chem. Eng. Data 1964, 9, 267–268. https://doi.org/10.1021/je60021a044.

(1252)	Blumenshine, R. L.; Sears, P. G. Several Properties of the 2-Pyrrolidone-Water System as Functions of Composition and Temperature. J. Chem. Eng. Data 1966, 11, 141–143. https://doi.org/10.1021/je60029a002.

(1253)	Rivkin, S. L.; Levin, A. Ya. Experimental Study of the Viscosity of Water and Vapor. Teploenergetika 1966, 79–82.

(1254)	Wallace, W. J.; Shephard, C. S.; Underwood, C. Densities, Refractive Indicies, Molar Refractions, Viscosities and Dielectric Constants of Triethylene Glycol Dimethyl Ether-Water Solutions at 25C. J. Chem. Eng. Data 1968, 13, 11–13. https://doi.org/10.1021/je60036a004.

(1255)	Hayduk, W.; Laudie, H.; Smith, O. H. Viscosity, Freezing Point, Vapor-Liquid Equilibria, and Other Properties of Aqueous-Tetrahydrofuran Solutions. J. Chem. Eng. Data 1973, 18, 373–376. https://doi.org/10.1021/je60059a027.

(1256)	Deckwer, W. Density, Viscosity, Vapor Pressure, and Hydrogen Solubility of Aqueous MnSO4 Solutionos. J. Chem. Eng. Data 1980, 25, 75–76. https://doi.org/10.1021/je60084a022.

(1257)	Sacco, A.; Petrella, G.; Castagnolo, M.; Dell’Atti, A. Excess Volumes and Viscosity of Water-Sulfolane Mixtures at 30, 40 and 50. Degree.C. Thermochim. Acta 1981, 44, 59–66. https://doi.org/10.1016/0040-6031(81)80271-7.

(1258)	Collings, A. F.; Bajenov, N. A High Precision Capillary Viscometer and Further Relative Results for the Viscosity of Water. Metrologia 1983, 19, 61.

(1259)	James, C. J.; Mulcahy, D. E.; Steel, B. J. Viscometer Calibration Standards: Viscosities of Water Between 0 and 60 Deg C and of Selected Aqueous Sucrose Sulutions at 15 Deg C from Measurements with A Flared Capillary Viscometer. J. Phys. D: Appl. Phys. 1984, 17, 225–230. https://doi.org/10.1088/0022-3727/17/2/006.

(1260)	Guarino, G.; Ortona, O.; sartorio, R.; Vitagliano, V. Diffusion, Viscosity, and Refractivity Data of the Systems Dimethyl- Formamide-Water and N-Methylpyrrolidone-Water at 5 C. J. Chem. Eng. Data 1985, 30, 366–368. https://doi.org/10.1021/je00041a039.

(1261)	Al-Ghawas, H. A.; Hagewiesche, D. P.; Ruiz-Ibanez, G.; Sandall, O. C. Physicochemical Properties Important for Carbon Dioxide Absorption in Aqueous Methyldiethanolamine. J. Chem. Eng. Data 1989, 34, 385–391. https://doi.org/10.1021/je00058a004.

(1262)	Palepu, R.; Clarke, J. Viscosities and Densities of 2,2,2-Trifluoroethanol + Water at Various Temperatures. Thermochim. Acta 1989, 156, 359–363. https://doi.org/10.1016/0040-6031(89)87202-8.

(1263)	Pandey, J. D.; Shukla, A.; Rai, R. D.; Misra, K. Ultrasonic, Volumetric, and Viscometric Studies of Tetracycline and Its Allied Compound. J. Chem. Eng. Data 1989, 34, 29–31. https://doi.org/10.1021/je00055a010.

(1264)	Laachach, A.; Ferriol, M.; Cohen-Adad, M. T.; Huck, J.; Jorat, L.; Noyel, G.; Getzen, F. W.; Bureau, J. C. Densities, Molar Volumes and Viscosities in the Systems Water-Hydrazine, Water-Methylhydrazine and Water-1,1- Dimethylhydrazine. Fluid Phase Equilib. 1992, 71, 301–312. https://doi.org/10.1016/0378-3812(92)80005-t.

(1265)	Liu, W.; Wang, J.; Wang, C.; Lu, J. Viscosity of Amino Acids in Methanol - Water Mixtures. Wuli Huaxue Xuebao 1992, 8, 742–748.

(1266)	Das, B.; Hazra, D. K. Viscosities and Excess Molar Volumes for 2-Methoxy-1-Ethanol + Water at Different Temperatures. J. Chem. Eng. Data 1993, 38, 361–363. https://doi.org/10.1021/je00011a007.

(1267)	Lee, M. J.; Hwang, S. M.; Kuo, Y. C. Densities and Viscosities of Binary Solutions Containing Butylamine, Benzylamine, and Water. J. Chem. Eng. Data 1993, 38, 577–579. https://doi.org/10.1021/je00012a026.

(1268)	Solimo, H. N.; Gramajo de Doz, M. B. Liquid-Liquid Equilibrium, Densities, Viscosities, Refractive Indices, and Excess Properties of the Ternary System Water + 4-Hydroxy-4-Methyl-2- Pentanone + Benzyl Alcohol at 298.15 K. J. Chem. Eng. Data 1995, 40, 563–566. https://doi.org/10.1021/je00019a004.

(1269)	Pal, A.; Singh, Y. P. Excess Molar Volumes and Viscosities for Glycol Ether-Water Solutions at the Temperature 308.15 K: Ethylene Glycol Monomethyl, Diethylene Glycol Monomethyl, and Triethylene Glycol Monomethyl Ethers. J. Chem. Eng. Data 1996, 41, 425–427. https://doi.org/10.1021/je950198g.

(1270)	Moha-Ouchane, M.; Boned, C.; Allal, A.; Benseddik, M. Viscosity and Excess Volume at High Pressures in Associative Binaries. Int. J. Thermophys. 1998, 19, 161–189. https://doi.org/10.1023/a:1021455203728.

(1271)	Henni, A.; Tontiwachwuthlkul, P.; Chakma, A.; Mather, A. E. Densities and Viscosities of Triethylene Glycol Monomethyl Ether +Water Solutions in the Temperature Interval 25 Deg C - 80 Deg C. J. Chem. Eng. Data 1999, 44, 101–107. https://doi.org/10.1021/je980127s.

(1272)	Pai, Y.-H.; Chen, L.-J. Liquid-Liquid Equilibria of Two Binary Systems: Water + 1-Pentanol and Water + 2-Methyl-2-Butanol and Two Ternary Systems: Water + 1 -Pentanol + 2-Butyloxyethanol and Water + 2-Methyl-2-Butanol + 2-Butyloxyethanol. Fluid Phase Equilib. 1999, 155, 95–105. https://doi.org/10.1016/s0378-3812(98)00456-7.

(1273)	Romero, C. M.; Rojas, J. L. Apparent Molar Volumes and Viscosities of DL-Alpha-Alanine in Water-Ethanol Mixtures. Phys. Chem. Chem. Phys. 2000, 38, 237–243.

(1274)	Akhtar, S.; Faruk, A. N. M. O.; Saleh, M. A. Viscosity of Aqueous Solutions of Formamide, N-Methylformamide, and N,N-Dimethylformamide. Phys. Chem. Liq. 2001, 39, 383–399.

(1275)	Henni, A.; Tontiwachwuthlkul, P.; Chakma, A.; Mather, A. E. Volumetric Properties and Viscosities for Aqueous Diglycolamine Solutions from 25 Deg C to 70 Deg C. J. Chem. Eng. Data 2001, 46, 56–62. https://doi.org/10.1021/je000136c.

(1276)	Saleh, M. A.; Akhtar, S.; Ahmed, M. S.; Uddin, M. H. Viscosities of Aqueous Solutions of Dimethylsulfoxide, 1,4-Dioxane, and Tetrahydrofuran. Phys. Chem. Liq. 2001, 39, 551–563.

(1277)	Maham, Y.; Lebrette, L.; Mather, A. E. Viscosities and Excess Properties of Aqueous Solutions of Mono-and Diethylethanolamines at Temperatures between 298.15 and 353.15 K. J. Chem. Eng. Data 2002, 47, 550–553. https://doi.org/10.1021/je015528d.

(1278)	Palaiologou, M. M.; Molinou, I. E.; Tsierkezos, N. G. Viscosity Studies on Lithium Bromide in Water + Dimethyl Sulfoxide Mixtures at 278.15 K and 293.15 K. J. Chem. Eng. Data 2002, 47, 1285–1289. https://doi.org/10.1021/je020063s.

(1279)	Saleh, M. A.; Ahmed, M. S.; Islam, M. S. VISCOSITY AND EXCESS VISCOSITY OF DILUTE AQUEOUS SOLUTIONS OF ETHYLENEDIAMINE, TRIMETHYLENEDIAMINE AND N,NDIMETHYLTRIMETHYLENEDIAMINE. Phys. Chem. Liq. 2002, 40, 477–490.

(1280)	Abdulagatov, I. M.; Azizov, N. D. Viscosity for Aqueous Li2SO4 Solutions at Temperatures from 298 to 575 K and at Pressures up to 30 MPa. J. Chem. Eng. Data 2003, 48, 1549–1556. https://doi.org/10.1021/je030177j.

(1281)	Henni, A.; Hromek, J.; Tontiwachwuthlkul, P.; Chakma, A. Volumetric Properties and Viscosities for Aqueous AMP Solutions from 25 0C to 70 0C. J. Chem. Eng. Data 2003, 48, 551–556. https://doi.org/10.1021/je0201119.

(1282)	Kapadi, U. R.; Hundiwale, D. G.; Patil, N. B.; Lande, M. K. Effect of Temperature on Excess Molar Volumes and Viscosities of Binary Mixtures of Ethylenediamine and Water. Fluid Phase Equilib. 2003, 205, 267–274. https://doi.org/10.1016/s0378-3812(02)00293-5.

(1283)	Uchida, T.; Ohmura, R.; Nagao, J.; Takeya, S.; Ebinuma, T.; Narita, H. Viscosity of Aqueous CO2 Solutions Measured by Dynamic Light Scattering. J. Chem. Eng. Data 2003, 48, 1225–1229. https://doi.org/10.1021/je034041x.

(1284)	Henni, A.; Tontiwachwuthikul, P.; Chakma, A. Volumetric Properties and Viscosities for Aqueous NFM Solutions from 25 Deg C to 70 Deg C. J. Chem. Eng. Data 2004, 49, 1724–1726. https://doi.org/10.1021/je0498208.

(1285)	Li, X.-X.; Liu, Y.-X.; Wei, X.-H. Physical-Chemical Properties and Liquid-Liquid Equilibria for Binary Mixture of Diethylene Glycol Diethyl Ether with Water. J. Chem. Eng. Data 2004, 49, 1043–1045. https://doi.org/10.1021/je049932z.

(1286)	Abdulagatov, I. M.; Zeinalova, A.; Azizov, N. D. Viscosity of Aqueous Na2SO4 Solutions at Temperatures from 298 to 573K and at Pressures up to 40MPa. Fluid Phase Equilib. 2005, 227, 57–70. https://doi.org/10.1016/j.fluid.2004.10.028.

(1287)	Bernal-Garcia, J. M.; Hall, K. R.; Estrada-Baltazar, A.; Iglesias-Silva, G. A. Density and Viscosity of Aqueous Solutions of N,N-Dimethylethanolamine at p = 0.1 MPa from T = (293.15 to 363.15) K. J. Chem. Thermodyn. 2005, 37, 762–767. https://doi.org/10.1016/j.jct.2004.11.016.

(1288)	Liu, W.; Zhao, T.; Zhang, Y.; Wang, H.; Yu, M. The Physical Properties of Aqueous Solutions of the Ionic Liquid [BMIM][BF4]. J. Solution Chem. 2006, 35, 1337–1346.

(1289)	Pang, F.-M.; Seng, C.-E.; Teng, T.-T.; Ibrahim, M. H. Densities and Viscosities of Aqueous Solutions of 1-Propanol and 2-Propanol at Temperatures from 293.15 K to 333.15 K. J. Mol. Liq. 2007, 136, 71–78. https://doi.org/10.1016/j.molliq.2007.01.003.

(1290)	Abdulagatov, I.; Azizov, N. Experimental Study of the Effect of Temperature, Pressure, and Concentration on the Viscosity of Aqueous KBr Solutions. J. Solution Chem. 2008, 37, 3–26.

(1291)	Abu-Daabes, M. A.; Awwad, A. M. Volumetric and Viscometric Properties of Aqueous Solutions of N-(2-Hydroxyethyl)Morpholine at T = (293.15, 303.15, 313.15, 323.15, 333.15) K. J. Chem. Thermodyn. 2008, 40, 874–878. https://doi.org/10.1016/j.jct.2008.01.007.

(1292)	Nikam, P. S.; Aher, J. S.; Kharat, S. J. Viscosities of Ammonium Sulfate, Potassium Sulfate, and Aluminum Sulfate in Water and Water + N,N-Dimethylformamide Mixtures at Different Temperatures. J. Chem. Eng. Data 2008, 53, 2469–2472. https://doi.org/10.1021/je800330d.

(1293)	Besbes, R.; Ouerfelli, N.; Latrous, H. Density, Dynamic Viscosity, and Derived Properties of Binary Mixtures of 1,4 Dioxane with Water at T=298.15 K. J. Mol. Liq. 2009, 145, 1–4. https://doi.org/10.1016/j.molliq.2008.09.009.

(1294)	Hartono, A.; Svendsen, H. F. Density, Viscosity, and Excess Properties of Aqueous Solution of Diethylenetriamine (DETA). J. Chem. Thermodyn. 2009, 41, 973–979. https://doi.org/10.1016/j.jct.2008.11.012.

(1295)	Palani, R.; Geetha, A. Acoustical and Excess Thermodynamic Studies of Molecular Interaction in Aqueous Mixed Solvent Systems at 303, 308 and 313K. Phys. Chem. Liq. 2009, 47, 542–552. https://doi.org/10.1080/00319100802562862.

(1296)	Sadeghi, R.; Goodarzi, B.; Karami, K. Effect of Potassium Citrate Salts on the Transport Behavior of L-Alanine in Aqueous Solutions at T = (293.15 to 308.15) K. J. Chem. Eng. Data 2009, 54, 791–794. https://doi.org/10.1021/je800559d.

(1297)	Wang, L.-C.; Ding, H.; Zhao, J.-H.; Song, C.-Y.; Wang, J.-S. Density and Viscosity of (4-Picoline + Water) Binary Mixtures from T ) (298.15 to 338.15) K. J. Chem. Eng. Data 2009, 54, 1000–1003. https://doi.org/10.1021/je8008182.

(1298)	Blanco, A.; Garcia-Abuin, A.; Gomez-Diaz, D.; Navaza, J. M.; Vidal-Tato, I. Influence of Temperature and Composition upon Density, Viscosity, Speed of Sound, and Refractive Index of Aqueous Solutions of 1-Ethyl-2-Pyrrolidinone. J. Chem. Eng. Data 2010, 55, 962–965. https://doi.org/10.1021/je9003959.

(1299)	Qiao, Y.; Di, Z.; Ma, P. S.; Ma, Y.; Xia, S. Viscosities of Pure Water, Acetic Acid + Water, and p-Xylene + Acetic Acid + Water at Different Temperature and Pressure*. Chin. J. Chem. Eng. 2010, 18, 446–454.

(1300)	Dhondge, S. S.; Pandhurnekar, C.; Garade, S.; Dadure, K. Volumetric and Transport Behavior of Different Carbohydrates in Aqueous and Aqueous Urea Mixtures at Different Temperatures. J. Chem. Eng. Data 2011, 56, 3484–3491. https://doi.org/10.1021/je2000099.

(1301)	Goodwin, A. R. H.; Marsh, K. M. An Absolute Viscometer for Liquids: Measurement of the Viscosity of Water at T = 298.15 K and p = 0.1 MPa. J. Chem. Eng. Data 2011, 56, 167–170. https://doi.org/10.1021/je100990n.

(1302)	Nain, A. K.; Lather, M.; Sharma, R. K. Volumetric, Ultrasonic and Viscometric Behavior of l-Methionine in Aqueous-Glucose Solutions at Different Temperatures. J. Mol. Liq. 2011, 159, 180–188.

(1303)	Rajagopal, K.; Gladson, S. E. THERMODYNAMIC INTERACTIONS OF POTASSIUM FLUORIDE IN AQUEOUS DIMETHYL SULFOXIDE SOLUTIONS AT DIFFERENT TEMPERATURES. Thermochim. Acta 2011, 525, 197–205. https://doi.org/10.1016/j.tca.2011.08.013.

(1304)	Lu, J.-G.; Hua, A.-C.; Xu, Z.-W.; Fan, F.; Cheng, L.; Lin, F.-Y. Measurement and Prediction of Densities, Viscosities, and Surface Tensions for Aqueous Solutions of Potassium Citrate. Fluid Phase Equilib. 2012, 327, 9–13. https://doi.org/10.1016/j.fluid.2012.05.007.

(1305)	Ouerfelli, N.; Barhoumi, Z.; Iulian, O. Viscosity Arrhenius Activation Energy and Derived Partial Molar Properties in 1,4-Dioxane +Water Binary Mixtures from 293.15 to 323.15 K. J. Solution Chem. 2012, 41, 458–474. https://doi.org/10.1007/s10953-012-9812-9.

(1306)	Ren, N.; Wu, Y.-T.; Ma, C.-F. Experimental Study of Viscosity of New Kind of Molten Salt With Low Melting Point. J. Eng. Thermophys. 2012, 33, 497–500.

(1307)	Bald, A.; Kinart, Z.; Wypych-Stasiewicz, A.; Tomas, R. Conductance Studies of NaCl, KCl, NaBr, KBr, Bu4NBr, and NaBPh4 in Water + 2-Methoxyethanol Mixtures at 298.15 K. J. Mol. Liq. 2013, 182, 14–24. https://doi.org/10.1016/j.molliq.2013.02.017.

(1308)	Banipal, T. S.; Singh, H.; Banipal, P. K.; Singh, V. Volumetric and Viscometric Studies on L-Ascorbic Acid, Nicotinic Acid, Thiamine Hydrochloride and Pyridoxine Hydrochloride in Water at Temperatures (288.15 to 318.15) K and at Atmospheric Pressure. Thermochim. Acta 2013, 553, 31–39. https://doi.org/10.1016/j.tca.2012.10.017.

(1309)	Chandra, A.; Patidar, V.; Singh, M.; Kale, R. K. Physicochemical and Friccohesity Study of Glycine, L-Alanine and L-Phenylalanine with Aqueous Methyltrioctylammonium and Cetylpyridinium Chloride from T = (293.15 to 308.15) K. J. Chem. Thermodyn. 2013, 65, 18–28. https://doi.org/10.1016/j.jct.2013.05.037.

(1310)	Dhondge, S. S.; Deshmukh, D. W.; Paliwal, L. J.; Dahasahasra, P. N. The Study of Molecular Interactions of Ascorbic Acid and Sodium Ascorbate with Water at Temperatures (278.15, 288.15 and 298.15) K. J. Chem. Thermodyn. 2013, 67, 217–226. https://doi.org/10.1016/j.jct.2013.08.016.

(1311)	Jozwiak, M.; Tyczynska, M.; Bald, A. Viscosity of Urea in the Mixture of N,N-Dimethylformamide and Water. J. Chem. Eng. Data 2013, 58, 217–224. https://doi.org/10.1021/je300608v.

(1312)	Siongco, K. R.; Leron, R. B.; Li, M.-H. Densities, Refractive Indices, and Viscosities of N,N-Diethylethanol Ammonium Chloride - Glycerol or - Ethylene Glycol Deep Eutectic Solvents and Their Aqueous Solutions. J. Chem. Thermodyn. 2013, 65, 65–72. https://doi.org/10.1016/j.jct.2013.05.041.

(1313)	Andanson, J.-M.; Traikia, M.; Husson, P. Ionic Association and Interactions in Aqueous Methylsulfate Alkyl-Imidazolium-Based Ionic Liquids. J. Chem. Thermodyn. 2014, 77, 214–221. https://doi.org/10.1016/j.jct.2014.01.031.

(1314)	Dhondge, S. S.; Dahasahasra, P. N.; Paliwal, L. J.; Deshmukh, D. W. Density and Viscosity Study of Nicotinic Acid and Nicotinamide in Dilute Aqueous Solutions at and around the Temperature of the Maximum Density of Water. J. Chem. Thermodyn. 2014, 76, 16–23. https://doi.org/10.1016/j.jct.2014.02.024.

(1315)	He, X.; Shao, Q.; Kong, W.; Yu, L.; Zhang, X.; Deng, Y. A Simple Method for Estimating Mutual Diffusion Coefficients of Ionic Liquids-Water Based on an Optofluidic Chip. Fluid Phase Equilib. 2014, 366, 9–15. https://doi.org/10.1016/j.fluid.2014.01.003.

(1316)	Jiang, X.; Zhu, C.; Ma, Y. Volumetric and Viscometric Studies of Amino Acids in L-Ascorbic Acid Aqueous Solutions at T = (293.15 to 323.15) K. J. Chem. Thermodyn. 2014, 71, 50–63. https://doi.org/10.1016/j.jct.2013.11.002.

(1317)	Krolikowska, M.; Lipinski, P.; Maik, D. Density, Viscosity and Phase Equilibria Study of (Ethylsulfate - Based Ionic Liquid + Water) Binary Systems as a Function of Temperature and Composition. Thermochim. Acta 2014, 582, 1–9. https://doi.org/10.1016/j.tca.2014.02.019.

(1318)	Kumar, H.; Singla, M.; Katal, A. Effect of N-Acetylglycine on Volumetric, Acoustic and Viscometric Behaviour of Aqueous Amoxicillin Solutions. Thermochim. Acta 2014, 583, 49–58. https://doi.org/10.1016/j.tca.2014.03.013.

(1319)	Mehrdad, A.; Shekaari, H.; Niknam, Z. Viscometric Studies of Interactions between Ionic Liquid 1-Octyl-3-Methyl-Imidazolium Bromide and Polyvinyl Pyrrolidone in Aqueous Solutions. J. Chem. Thermodyn. 2014, 79, 1–7. https://doi.org/10.1016/j.jct.2014.07.005.

(1320)	Navarro, S. S.; Leron, R. B.; Soriano, A. N.; Li, M.-H. Thermophysical Property Characterization of Aqueous Amino Acid Salt Solution Containing Serine. J. Chem. Thermodyn. 2014, 78, 23–31. https://doi.org/10.1016/j.jct.2014.05.019.

(1321)	Zawadzki, M.; Krolikowska, M.; Lipinski, P. Physicochemical and Thermodynamic Characterization of N-Alkyl-N-Methylpyrrolidinium Bromides and Its Aqueous Solutions. Thermochim. Acta 2014, 589, 148–157. https://doi.org/10.1016/j.tca.2014.05.028.

(1322)	Garcia, A. A.; Leron, R. B.; Soriano, A. N.; Li, M.-H. Thermophysical Property Characterization of Aqueous Amino Acid Salt Solutions Containing .Alpha.-Aminobutyric Acid. J. Chem. Thermodyn. 2015, 81, 136–142. https://doi.org/10.1016/j.jct.2014.10.005.

(1323)	Kumar, K.; Chauhan, S. Volumetric, Compressibility and Viscometric Studies on Sodium Cholate/Sodium Deoxycholate Amino Acid Interactions in Aqueous Medium. Thermochim. Acta 2015, 606, 12–24. https://doi.org/10.1016/j.tca.2015.02.014.

(1324)	Raman, M. S.; Kesavan, M.; Senthilkumar, K.; Ponnuswamy, V. Ultrasonic, DFT and FT-IR Studies on Hydrogen Bonding Interactions in Aqueous Solutions of Diethylene Glycol. J. Mol. Liq. 2015, 202, 115–124. https://doi.org/10.1016/j.molliq.2014.12.014.

(1325)	Xu, Q.; Sun, S.; Lan, G.; Xiao, J.; Zhang, J.; Wei, X. Excess Properties and Spectral Investigation for the Binary System Diethylene Glycol Dimethyl Ether + Water at T = (293.15, 298.15, 303.15, 308.15 and 313.15) K. J. Chem. Eng. Data 2015, 60, 2–10. https://doi.org/10.1021/je500389t.

(1326)	Shifrin, A. S. Viscosity of Water Steam at Atmospheric Pressure. Teploenergetika 1959, 22–27.

(1327)	Rivkin, S. L.; Levin, A. Ya.; Izrailevskii, L. B. Experimental Study of Viscosity of Water at Temperatures to 450 C and at Pressures up to 350 Bar. Teploenergetika 1968, 74–78.

(1328)	Zhdanov, A. G.; Lusternik, V. E.; Timrot, D. L. Capillary Viscometer for the Viscosity Determination of Vapor by Closed Scheme. Inzh.-Fiz. Zh. 1968, 15, 1120–1123.

(1329)	Timrot, D. L.; Serednitskaya, M. A.; Bespalov, M. S. Experimental Study of the Viscosity of Water Vapor at Temperatures from 50 to 500 C and at Pressures from 0.06 to 1.5 Bar. Teploenergetika 1973, 78–80.

(1330)	Nagashima, A.; Tanishita, I.; Mural, Y. Measurement of Pressure Effect on Viscosity of Steam. J. Chem. Eng. Data 1974, 19, 212–213. https://doi.org/10.1021/je60062a021.

(1331)	Timrot, D. L. Par Vysokih Parametrov v Energetike (Steam of High Parameters in Energetics); 1950.

(1332)	Mineev, V. N.; Zaidel, R. M. Viscosity of Water and Mercury under Shok Load. Zh. Eksp. Teor. Fiz. 1968, 54, 1633–1639.

(1333)	Rivkin, S. L.; Levin, A. Ya.; Izrailevskii, L. B.; Kharitonov, K. G. Experimental Study of the Viscosity of Water in the Supercrtical Region of Parameters State at Pressures up to 500 Bar and at Temperatures to 500 C. Teploenergetika 1973, 11–12.

(1334)	Liu, X. Y.; He, M. G.; Zhang, Y. Viscosity of Water in the Region around the Critical Point. J. Supercrit. Fluids 2012, 63, 150–154. https://doi.org/10.1016/j.supflu.2011.11.021.

(1335)	Teske, V.; Vogel, E.; Bich, E. Viscosity Measurements on Water Vapor and Their Evaluation. J. Chem. Eng. Data 2005, 50, 2082–2087. https://doi.org/10.1021/je050288d.

(1336)	Vogel, E. Precise Measurements of the Viscosity Coefficients of Nitrogen and Rare Gases between Room Temperature and 650 K. Ber. Bunsen-Ges. Phys. Chem. 1984, 88, 997–1002.

(1337)	Trautz, M.; Heberling, R. The Friction, Thermal Conduction and Diffusion in Gas Mixtures. XXV. The Internal Friction of Xenon and Its Mixtures with Hydrogen and Helium. Ann. Phys. (Leipzig) 1934, 412, 118–120. https://doi.org/10.1002/andp.19344120203.

(1338)	Trappeniers, N. J.; Botzen, A.; Van Oosten, J.; Van den Berg, H. R. THE VISCOSITY OF KRYPTON BETWEEN 25deg AND 75deg C AND AT PRESSURES UP TO 2000 ATM. Phys. Norv. 1965, 31, 945–952. https://doi.org/10.1016/0031-8914(65)90024-8.

(1339)	Reynes, E. G.; Thodos, G. The Viscosities of Argon, Krypton, and Xenon in the Dense Gaseous Region. Physica (Amsterdam) 1964, 30, 1529–1542. https://doi.org/10.1016/0031-8914(64)90177-6.

(1340)	Rankine, A. O. On the Viscosities of the Gases of the Argon Group. Proc. Roy. Soc. London Ser. A 1910, 83, 516–525. https://doi.org/10.1098/rspa.1910.0040.

(1341)	Nasini, A. G.; Rossi, C. Viscosita Di Gas Rari. Gazz. Chim. Ital. 1928, 58, 433–442.

(1342)	Boon, J. P.; Legros, J. C.; Thomaes, G. ON THE PRINCIPLE OF CORRESPONDING STATES FOR THE VISCOSITY OF SIMPLE LIQUIDS. Physica (Amsterdam) 1967, 33, 547–557.

(1343)	Slyusar, V. P.; Rudenko, N. S.; Tretyakov, V. M. Eksperimentalnoe Issledovanie Vyazkosti Prostykh Veshchestv Na Linii Nasyshcheniya i Pod Davleniem. Ukr. Fiz. Zh. Russ. Ed. 1972, 17, 1257–1263.

(1344)	Roy, C. S.; Rakshit, A. B.; Barua, A. K. Viscosity of Gas Mixtures and Polar-Nonpolar Molecular Interactions. Physica (Amsterdam) 1973, 66, 442–454. https://doi.org/10.1016/0031-8914(73)90293-0.

(1345)	Thomas, L. H. Viscosity and Molecular Association. III. Association of Phenols and Amides. J. Chem. Soc. 1960, 4906–4914. https://doi.org/10.1039/jr9600004906.

(1346)	Rigby, M.; Smith, E. B. Viscosities of the Inert Gases. Trans. Faraday Soc. 1966, 62, 54–58. https://doi.org/10.1039/tf9666200054.

(1347)	Maitland, G. C.; Smith, E. B. Critical Reassessment of Viscosities of 11 Common Gases. J. Chem. Eng. Data 1972, 17, 150–156. https://doi.org/10.1021/je60053a015.

(1348)	Goldblatt, M.; Wageman, W. E. High Temperature Viscosity Ratios for Xenon. Phys. Fluids 1971, 14, 1024–1025. https://doi.org/10.1063/1.1693533.

(1349)	May, E. F.; Berg, R. F.; Moldover, M. R. Reference Viscosities of H_2_, CH_4_, Ar, and Xe at Low Densities. Int. J. Thermophys. 2007, 28, 1085–1110. https://doi.org/10.1007/s10765-007-0198-7.

(1350)	Vogel, E.; Bastubbe, E.; Rohde, S. Calibration of an Oscillating Quartz Disc Viscometer for Precision Measurements of Gases and Organic Vapors. Wiss. Z. Univ. Rostock math. naturw. Reihe 1984, 33, 34–42.

(1351)	Rakshit, A. B.; Roy, C. S. Viscosity and Polar-Nonpolar Interactions in Mixtures of Inert Gases with Ammonia. Phys. Norv. 1974, 78, 153–164. https://doi.org/10.1016/0031-8914(74)90316-4.

(1352)	Kestin, J.; Khalifa, H. E.; Wakeham, W. A. The Viscosity and Diffusion Coefficients of the Binary Mixtures of Xenon with the Other Noble Gases. Physica A Amsterdam 1978, 90, 215–228. https://doi.org/10.1016/0378-4371(78)90110-3.

(1353)	Vogel, E. The Viscosities of Dilute Kr, Xe, and CO2 Revisited: New Experimental Reference Data at Temperatures from 295 K to 690 K. Int. J. Thermophys. 2016, 37, 63. https://doi.org/10.1007/s10765-016-2068-7.

(1354)	Lin, H.; Che, J.; Zhang, J. T.; Feng, X. J. Measurements of the Viscosities of Kr and Xe by the Two-Capillary Viscometry. Fluid Phase Equilib. 2016, 418, 198–203. https://doi.org/10.1016/j.fluid.2016.01.038.

(1355)	Golovicher, L. E.; Kolenchits, O. A.; Nesterov, N. A. DYNAMIC VISCOSITY OF GASES IN A WIDE RANGE OF TEMPERATURE. I. Inzh. Fiz. Zh. 1989, 56, 982–987. https://doi.org/10.1007/BF00870442.

(1356)	Barr, I. A.; Matthews, G. P.; Smith, E. B.; Tindell, A. R. Intermolecular Forces and the Gaseous Viscosities of Argon-Xenon Mixtures. J. Phys. Chem. 1981, 85, 3342–3347. https://doi.org/10.1021/j150622a027.

(1357)	Sevast’yanov, R. M.; Zykov, N. A. Transport Coefficients of the Monatomic Gases in the Temperature Range from 100 to 3000 K. Inzh. Fiz. Zh. 1978, 34, 118–125.

(1358) Timrot, D. L.; Traktueva, S. A. Dependence of the Viscosity of CO_2_ and SF_6_ on Temperature at moderate Densities. Teploenergetika 1975, 22, 81-83.

(1359) Baylaucq, A.; Boned, C.; Canet, X.; Zeberg-Mikkelsen, C. K. High-Pressure (up to 140 MPa) Dynamic Viscosity of the Methane and Toluene System: Measurements and Comparative Study of Some Representative Models. Int. J. Thermophys. 2003, 24, 621–638. https://doi.org/10.1023/a:1024023913165.

(1360) Baylaucq, A.; Boned, C.; Dauge, P.; Lagourette, B. Measurements of the Viscosity and Density of Three Hydrocarbons and the Three Associated Binary Mixtures Versus Pressure and Temperature. Int. J. Thermophys. 1997, 18, 3–23. https://doi.org/10.1007/bf02575198.
